# Clustered somatic mutations are frequent in transcription factor binding motifs within proximal promoter regions in melanoma and other cutaneous malignancies

**DOI:** 10.18632/oncotarget.11892

**Published:** 2016-09-07

**Authors:** Andrew J. Colebatch, Leon Di Stefano, Stephen Q. Wong, Ross D. Hannan, Paul M. Waring, Alexander Dobrovic, Grant A. McArthur, Anthony T. Papenfuss

**Affiliations:** ^1^ Research Division, Peter MacCallum Cancer Centre, Victorian Comprehensive Cancer Centre, Victoria, Australia; ^2^ Department of Pathology, University of Melbourne, Victoria, Australia; ^3^ Bioinformatics Division, The Walter and Eliza Hall Institute of Medical Research, Parkville, Victoria, Australia; ^4^ ACRF Department of Cancer Biology and Therapeutics, John Curtin School of Medical Research, The Australian National University, Australian Capital Territory, Australia; ^5^ Translational Genomics and Epigenomics Laboratory, Olivia Newton-John Cancer Research Institute, Victoria, Australia; ^6^ School of Cancer Medicine, La Trobe University, Bundoora, Victoria, Australia; ^7^ Sir Peter MacCallum Department of Oncology, University of Melbourne, Victoria, Australia; ^8^ Department of Medical Biology, University of Melbourne, Victoria, Australia

**Keywords:** melanoma, gene promoter, non-coding mutations, transcription factors, ultraviolet radiation

## Abstract

Most cancer DNA sequencing studies have prioritized recurrent non-synonymous coding mutations in order to identify novel cancer-related mutations. Although attention is increasingly being paid to mutations in non-coding regions, standard approaches to identifying significant mutations may not be appropriate and there has been limited analysis of mutational clusters in functionally annotated non-coding regions. We sought to identify clustered somatic mutations (hotspot regions across samples) in functionally annotated regions in melanoma and other cutaneous malignancies (cutaneous squamous cell carcinoma, basal cell carcinoma and Merkel cell carcinoma). Sliding window analyses revealed numerous recurrent clustered hotspot mutations in proximal promoters, with some specific clusters present in up to 25% of cases. Mutations in melanoma were clustered within ETS and Sp1 transcription factor binding motifs, had a UV signature and were identified in other cutaneous malignancies. Clinicopathologic correlation and mutation analysis support a causal role for chronic UV irradiation generating somatic mutations in transcription factor binding motifs of proximal promoters.

## INTRODUCTION

Most DNA sequencing studies in cancer have focused primarily on the analysis of coding regions of the genome [1–6]. Indeed, the statistical approaches used to infer significance of mutations are largely motivated by the idea of single nucleotide or amino acid changes, which may not be appropriate for changes to non-coding features [4, 7–9]. Moreover, there is an increasing appreciation of the biological importance of somatic mutations in non-coding regions of the genome, including promoters, enhancers, insulators (together constituting cis-regulatory regions) [10, 11], as well as non-coding RNAs [12], to malignant transformation.

The first and most significant example of an oncogenic non-coding mutation is within the proximal promoter of the telomerase reverse transcriptase (*TERT*) gene, first demonstrated as frequently mutated in melanoma [13, 14], and subsequently in a variety of other malignancies [15–19]. The *TERT* promoter mutation occurs in up to 80% of melanomas [20, 21], establishing it as the commonest somatic mutation in this malignancy. The *TERT* promoter mutation occurs recurrently within one or other of two specific sites (known as C228T and C250T), and has functional consequences by maintaining expression of TERT at critical junctures of cell development [22] through the creation of novel ETS binding sites. The identification of *TERT* promoter mutations has led to several systematic searches of non-coding somatic mutations across various cancers. These efforts have uncovered significantly mutated non-coding regions adjacent to various genes, such as *SDHD*, *PLEKHS1, WDR74* [23], *DPH3* [24] and *NDUFB9* [25]. The mutations proximal to *SDHD* and *DPH3* occurred in ETS transcription factor binding motifs, while the *NDUFB9* promoter mutation occured in between a Sp1/KLF-like site and an ETS motif. Unlike the *TERT* promoter, these non-coding mutations did not create but rather ablated predicted ETS binding sites by altering the core GGAA sequence or a nucleotide flanking the canonical ETS DNA-binding motif. Of note is that all these above mutations were described in melanoma samples.

Given the importance of ETS-related mutations in melanoma, as demonstrated by the high frequency of *TERT* promoter mutations and the emerging literature on other non-coding promoter mutations related to ETS binding sites, we hypothesized that there would be somatic mutations present in other ETS transcription factor binding sites across the genome, particularly in regulatory regions. In order to find somatic mutations which might occur within the binding sites of ETS or other transcription factors, we systematically analyzed melanoma sequencing data for mutational clusters in transcription factor binding motif-sized windows across samples (henceforth referred to as ‘clustered mutations’), both within regulatory sequences and across the entire melanoma genome; having determined these clusters, we sought the same mutations in other cutaneous malignancies to determine the effect of cell-of-origin on clustered mutation presence and frequency.

## RESULTS

### Clustered promoter mutations are common in cutaneous melanoma

To identify recurrent mutations, we first systematically screened whole exome sequencing data from a previously published set of primary cutaneous melanoma samples [1] for clustered mutations, utilizing a heuristic sliding window approach with a threshold of 4 mutations present when evaluated across all samples in a 5 basepair (bp) window (a flowchart of the overall approach is in Figure S1). We used this data as a discovery set due to the extensive clinicopathologic annotation available and the common origin of samples from primary cutaneous melanomas. Whole exome data contains non-coding DNA due to capture of genomic regions adjacent to exons, including proximal promoter sequence adjacent to the first exon. The 5bp window size was chosen to approximate the width of a transcription factor binding site. This yielded 98 windows across the exome fitting these criteria, including canonical non-clustered *BRAF* and *NRAS* (BRAF V600 and NRAS Q61 mutations) mutation hotspot regions. Interestingly, approximately half of the recurrent mutations were in annotated promoter regions (Table 1). Fourteen of these promoters were bidirectional (the genomic start position of both genes being within 1 kb, Table S1).

**Table 1:** The number of mutation clusters from 34 cutaneous melanoma exomes in different categories of annotated genomic feature

Splice Site	Intron	5′UTR	3′UTR	Coding	Promoter
0	33	18	3	18	73

**Figure 1:**
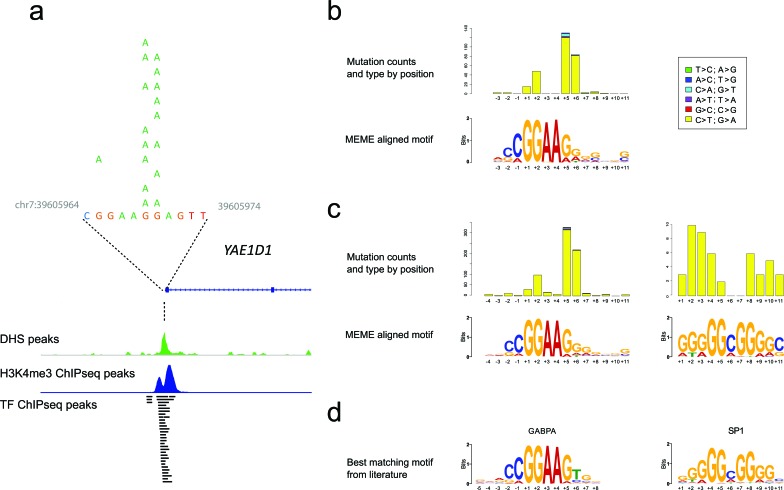
The relationship of clustered promoter mutations to transcription factor binding motifs **a.** Typical features of the clustered proximal promoter mutations (in *YAE1D1*). Here, the SNVs come from 12 samples, which were positive by both HRM and Sanger sequencing. These sites typically are near the TSS with peaks for DHS, H3K4me3 and multiple transcription factors (from ENCODE data). **b.** The motif predicted using MEME from the mutation clusters in the 34 melanoma exomes along with the position, type and frequency of SNVs in relation to the motif. **c.** The same information for the clusters from the 40 melanoma whole genomes. An Sp1-like signature was detected only in the whole genome data with frequencies substantially lower than the ETS motif. The C>T mutations occurring within the adenosines at positions +3 and +4 of the ETS motif from the whole genomes is due to SNVs creating an ETS site from the *TERT* promoter. **d.** The closest matching transcription factor binding sites identified using TOMTOM.

Because of the risk of false positive mutation calls in these sites, many of which were at the edge of the sequence adjacent to the captured first exon with consequently lower read depths, we sought to validate their presence using multiple orthogonal approaches. First, we evaluated a distinct set of 93 clinical melanoma samples for *YAE1D1* promoter mutations with high resolution melting (HRM) analysis followed by Sanger sequencing (Figure 1a; Figure S2). The *YAE1D1* promoter was selected due to the ease of interpretability of the HRM plots and the limited number of single nucleotide variant (SNV) sites compared to other windows. This demonstrated that 12 of the 93 (12.9%) melanoma samples contained *YAE1D1* promoter mutations in identical positions to those in the 34 exomes, being a combination of mononucleotide and dinucleotide mutations at G and GG sites.

Having confirmed a single promoter mutation position, we designed a custom multiplex sequencing panel to interrogate multiple clustered mutation sites in a larger clinical dataset. After exclusion of regions that failed primer design due to either to low primer binding specificity or to extreme primer GC% (GC% >80% or <20%), 77 regions were evaluated. We utilized an independent set of primary melanoma samples with extensive clinicopathologic data (*n* = 170) for this multiplexed assay. Additionally, sequencing data from 93 normal samples was available (72 from matched samples and 21 from unmatched samples). Examination of positions that were only mutated within the 34 whole exome samples revealed that many of the promoter regions in the clinical samples frequently also contained clusters of mutations, with up to 27.1% of samples possessing at least one mutation in these regions, excluding the *TERT* promoter (see Table 2 for locations with >10% incidence; Table S4 for the complete data). Four regions derived from the analysis of the 34 melanoma exomes lacked any mutations in the clinical samples, three from introns and one from a coding region.

We noted that all regions mutated in more than 10% of samples, apart from *TERT*, *BRAF* and *NRAS*, were distinguished by occurring within certain ENCODE data tracks, including DNase I hypersensitivity peaks, histone H3K4me3 peaks and multiple transcription factor ChIP-seq peaks, consistent with origin from active proximal promoters (see Figure 1a). No mutations in these regions were found within the 93 samples from normal tissue, confirming that these mutations were somatic in origin.

As a second validation, we next evaluated 40 whole genome sequences (WGS) from melanoma samples obtained from The Cancer Genome Atlas (TCGA) for clustered mutations in order to confirm our findings from whole exomes and capture a comprehensive landscape of these non-coding mutations. Initially, we used the same threshold of 4 mutations in a window across all sequences, but expanded the window to 15bp in size to capture the possibility of adjacent transcription factor binding sites leading to a wider motif.

The threshold of 4 mutations was chosen by simulating variants across 40 melanoma genomes using a Poisson binomial distribution. This demonstrated that the chance of finding a window with 4 or more mutations given the mutation rates of the melanoma genomes was *p* < 10^−6^ (p_adjusted_ < 0.005 after Bonferroni correction). Given the features identified in the whole exome data and in order to explore possible functional relevance, we focused at first on those clusters within annotated DNaseI hypersensitivity sites (DHS) sites and within transcription factor binding site regions, which were recorded as within the 5′-untranslated region (5′-UTR) or promoter of an annotated gene. This constituted a heuristic definition of a promoter, and is referred to as the ‘heuristic promoter’ approach.

Several DHS tracks are publically available with some variation between different sets. We initially screened using the DNase I Hypersensitivity Clusters in 125 cell types from ENCODE (V3) (wgEncodeRegDnaseClusteredV3) track from UCSC (derived from multiple cell lines), after which we compared this to the DHS track from the Melano ENCODE cell line (derived from a normal melanocyte). Using the former, we generated 182 windows which were annotated to 213 nearby genes (see Table S3), while using the Melano DHS data the list comprised 180 windows within the promoter and 5′UTR regions of 211 genes; these 180 windows were a subset of the 182 from the UCSC DHS analysis. Since the *TERT* promoter was one of the 2 windows lost and the latter was a superset of the former, we elected to utilize the wgEncodeRegDnaseClusteredV3 track in order to capture *TERT* promoter mutations in our analysis.

Comparison of the overlap between the 98 cluster windows from the 34 melanoma exomes and the 182 from the whole melanoma genomes revealed that there were only 22 windows within the intersection of the two sets. Fifty three of the cluster windows from the 34 melanoma exomes contain at least one SNV from the melanoma whole genome data corresponding to a SNV position from the 34 melanoma exomes.

### Clustered mutations are located within ETS and Sp1 motifs

We performed *de novo* motif discovery on the sequences from the cluster windows of both the whole exomes and the whole genomes using MEME. Remarkably, the whole exome cluster windows revealed a 14bp motif that strongly resembled an ETS transcription factor binding site (Figure 1b) in 50 windows (E-value 7.4 × 10^−56^); of these, 44 windows overlapped DHS sites. Mapping the location of mutations within this motif indicated that variants, either single nucleotide or dinucleotide, occurred most frequently at the location of the guanosines at positions +5 and +6 just 3′ to the core ‘GGAA’ motif, or within the guanosines of the core at positions +1 and +2. (Figures 1b, 1c).

Motif analysis of mutation clusters in the whole genome data unexpectedly revealed two enriched motifs, one matching an ETS binding site similar to the exome data, while the novel, second motif resembled a Sp1 binding site (Figure 1c); analysis using TOMTOM confirmed the similarity of these motifs to ETS (GABPA) and Sp1 transcription factor binding sites respectively (Figure 1d). After alignment of mutations to these motifs, the ETS sites showed the same positional mutation distribution as shown in the 34 whole exomes, while the Sp1 sites were mutated more evenly across the guanosines at a markedly lower frequency.

### Unbiased genome-wide statistical testing for significant clustering of SNVs

In our initial scan of the whole genome data, we fixed a window size (15bp) and minimum number of SNVs (4) above which a window was recorded for further analysis. There are at least three potential confounders of this approach. The first is the heterogeneity of background mutation rates across the genome [7]: by chance alone, we would expect to see more and larger clusters in regions with high background rates, and fewer and smaller clusters may be of interest if they occur in regions of low mutation density. The second is the choice of fixed thresholds. The third is variation in baseline mutation rates between patients, for example due to differences in UV exposure; the initial straightforward scan may have confounded intra-sample and cross-sample hotspot recurrence.

**Figure 2:**
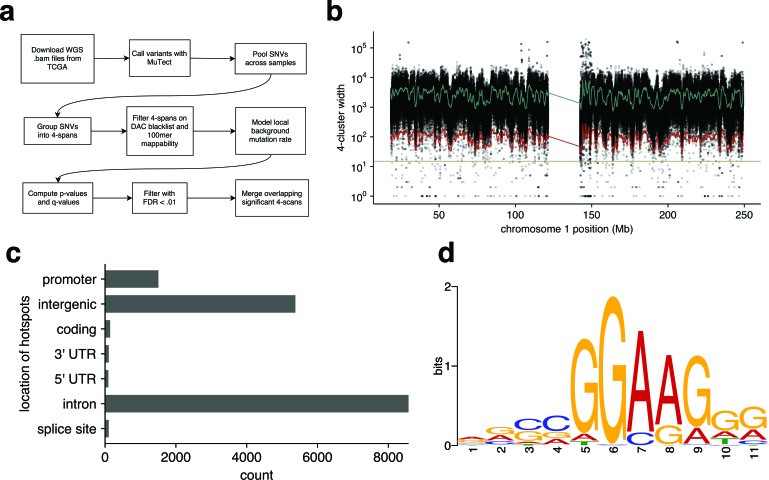
Unbiased, background-corrected, whole genome search of melanoma TCGA data **a.** Flowchart of the search method. **b.** Model fit to the distribution of 4-scan lengths along chromosome 1. The green line indicates estimated local average 4-scan width; the red line indicates the threshold below which our model deems 4-scans significant (q < 0.01); and the tan-colored line represents the fixed threshold of 15bp used in the initial search. **c.** Annotated locations of those 4-scans deemed significant by the model. Overlapping significant 4-scans were merged into hotspots. **d.** Motif discovered by MEME in a proximal promoter hotspots.

To overcome these shortcomings, we developed an unbiased, background-corrected statistical test for runs of 4 SNVs (4-scans) in the sample-merged TCGA whole genome SNV data that are significantly smaller than the estimated local background rate would predict (Figure 2a). This search was consistently less conservative than the initial 15bp sliding window heuristic promoter method (Figure 2b), and identified 13,434 significant 4-scans. After merging overlapping 4-scans, this identified 8,434 hotspots with an average width of 32.6bp (Figure S6, Table S6). Most of the hotspots were annotated as occurring in intronic or intergenic regions; approximately 1% fell in coding regions, and 9% were in promoters (Figure 2c). Sixty one percent (61%) were located in annotated repeats and may represent false positives. Around 1% were located in putative enhancers (Table S5). Of those detected with our earlier heuristic promoter method, 73% were also detected by the unbiased approach. The mean number of variants per hotspot was 4.5, and the average number of distinct samples contributing to each hotspot was 3.6 (Figures S7 and S8).

To confirm that this method could identify regions known to be biologically important in melanoma, we examined those hotspots with high numbers of mutations and a high proportion of samples; pleasingly this identified *TERT* and *BRAF* SNVs as striking outliers (Figure S9). Forming a restricted, proximal promoter-enriched subset of these hotspots (*n* = 456) using the same criteria as the heuristic promoter method and using MEME to search for recurrent motifs again identified an ETS binding site motif (Figure 2d). To evaluate whether motifs were present for hotspots in other genomic regions with different chromatin states (e.g. enhancers), we applied *de novo* motif discovery to those clusters arising in each different state (table S5) as determined from normal human melanocytes. The ETS motif was present only in the ‘Active TSS’ state, which corresponds to the promoter regions of actively transcribed genes.

### Clustered mutations show no local effect on gene expression

We next tested for changes in gene expression resulting from mutation clusters in immediately adjacent promoters. For this we utilized the 40 melanomas that had undergone whole genome sequencing and had gene expression data available for analysis from TCGA.

Prior to correction for multiple testing, 8 of 196 tested genes (*DPH3*, *G2E3*, *ALG10*, *ARHGAP21*, *SNHG6*, *CCDC174*, *RPS27*, *YIPF1*) showed a significant difference of expression for the gene product between cases with wild-type and mutated promoters. After correction, no genes achieved statistical significance. In addition, there was no evidence of differential expression for *TERT*; although differential expression of *TERT* with promoter mutations has been shown in multiple cancers, our negative result is consistent with an independent analysis of *TERT* expression in the same melanoma samples [24].

### Clustered mutations are associated with clinicopathologic features of UV exposure

Using a separate set of 170 primary cutaneous melanomas, we evaluated a series of clinicopathologic parameters that are known to be important in prognosis [26], including ulceration, age at diagnosis, stage and Breslow thickness (the depth of invasion of the primary melanoma), as well as those associated with chronic UV exposure, including solar elastosis (a histopathological manifestation of chronic UV skin exposure) and anatomical location. The basic clinicopathologic characteristics of the 170 clinical samples are presented in Table 3. For this analysis, we summed all valid SNVs (see Methods for inclusion criteria) per samples, and this sumn is referred to as the ‘promoter mutation load’.

**Figure 3:**
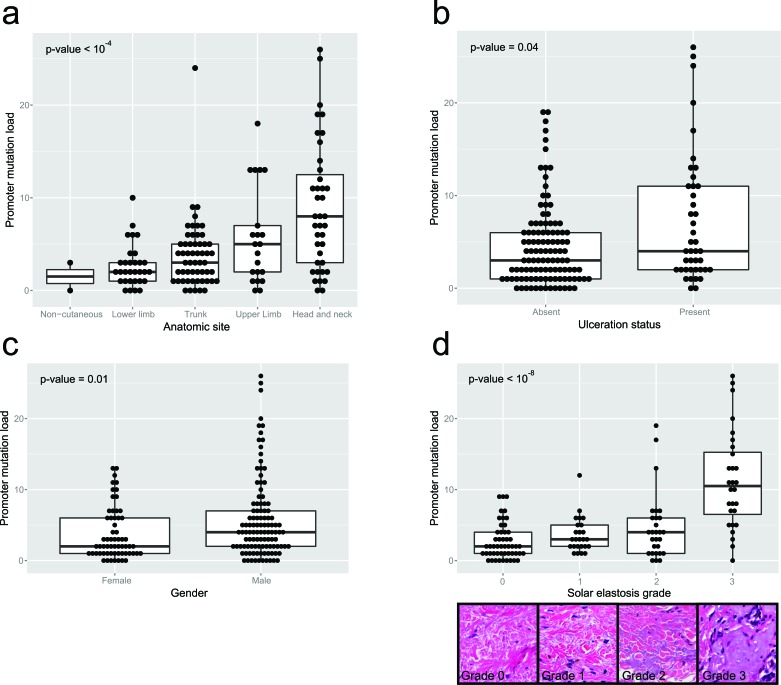
Clinicopathologic parameters compared to promoter mutation load for the 170 clinical melanoma samples, using the multiplex PCR assay **a.** relates the mutation load to the total number of high stringency SNVs to the anatomic site of the primary, while **b.** relates to ulceration status, **c.** to gender and **d.** to grade of solar elastosis. The inset picture shows examples of different grades of solar elastosis.

There was an increase in promoter mutation load with increasing solar elastosis scores (*p* < 10^−8^, Kruskal Wallis test; Figure 3d), and promoter mutation loads were higher in head and neck, and upper limb sites than trunk and lower limb sites (*p* < 10^−4^ Kruskal Wallis test; Figure 3a). Moreover there was a positive correlation between promoter mutation load and age at diagnosis (rho = 0.24, *p*-value = 0.002; Figure S3d). Promoter mutation loads were significantly higher in males (*p* = 0.012 Mann-Whitney U test; Figure 3c) and in ulcerated melanomas (*p* = 0.038 Mann-Whitney U test; Figure 3b). Finally, promoter mutation load varied significantly across different melanoma histological subtypes (*p* < 0.01 Kruskal Wallis test; Figure S3c), with the highest loads in desmoplastic melanomas followed by lentigo maligna melanomas. There was no significant association between promoter mutation load and tumor stage at diagnosis (Figure S3a) or their Breslow thickness (Figure S3b).

**Table 2:** Cluster mutation locations with >10% incidence across validation cohort of 170 melanoma clinical cases

Gene	Location	Incidence (%)
*TERT*	Promoter	76.8
*BRAF*	Codon 600	36.5
*DPH3*	Promoter	27.1
*RPS27*	5′UTR	25.9
*C16orf59*	Promoter	21.2
*RPL18A*	Promoter	18.8
*RPS3A*	Promoter	17.6
*KIAA0907*	Promoter	17.1
*CCDC94*	Promoter	17.1
*SLC30A6*	Promoter	15.9
*UBXN8*	Promoter	14.7
*MRPS31*	5′UTR	14.7
*NRAS*	Codon 61	13.5
*PSMC6*	Promoter	12.9
*CHCHD2*	5′UTR	12.9
*RBM22*	5′UTR	12.9
*SYF2*	Promoter	12.4
*YAE1D1*	Promoter	12.4
*ERGIC3*	5′UTR	12.4
*PSMD11*	Promoter	11.8
*NDUFB9*	5′UTR	11.8
*CDC37*	5′UTR	10.6
*POLDIP3*	Promoter	10.6
*NFIC*	Intron	10
*INO80B*	5′UTR	10

5′UTR - 5 prime untranslated region

**Table 3:** Clinicopathologic parameters of 170 melanoma samples

**Gender**	
Male	109
Female	61
**Anatomic location**	
Head and neck	39
Upper limb	21
Trunk	55
Lower limb	31
Non cutaneous	2
Not stated	22
**TERT promoter status**	
Wildtype	39
All mutations	129
228C>T	56
242CC>TT	9
250C>T	59
228C>T 250C>T	2
242CC>TT 250C>T	1
243C>T 250C>T	1
228C>T 242CC>TT	1
Not recorded	2
**Oncogene status**	
BRAF^1^	67
NRAS	26
Wildtype	129
**Histologic subtype**	
Superficial spreading	85
Nodular	46
Lentigo maligna melanoma	9
Melanoma NOS^2^	7
Desmoplastic	4
Acral lentinginous	2
MelTUMP^3^	2
Arising from blue nevus	1
Nevoid	1
Mucosal	1
Not recorded	12
**Solar elastosis grade**	
Grade 0	50
Grade 1	25
Grade 2	28
Grade 3	28
Not recorded	39
**Ulceration**	
Present	42
Absent	111
Not recorded	17
**Stage at diagnosis**	
I	60
II	62
III	36
IV	8
Not recorded	4
**Breslow thickness**	
0.01-1.00mm	40
1.01-2.00mm	36
2.01-4.00mm	55
>4.00mm	26
Not recorded	13

[Table Legend: 1 - canonical BRAF V600 mutations, 2 - Not otherwise specified, 3 - Melanocytic Tumor of Uncertain Malignant Potential]

### Analysis of the whole genome mutation clusters

Analysis of the mutation spectra of the clustered promoter mutations in the whole genome datasets demonstrated that 97% of all mutations were C→T or G→A transitions at dipyrimidine sites, with 12% of these being CC→TT or GG→AA dinucleotide transitions. This is consistent with a UV signature according to the criteria of Brash in his recent meta-analysis of UV signature mutations [27].

**Figure 4:**
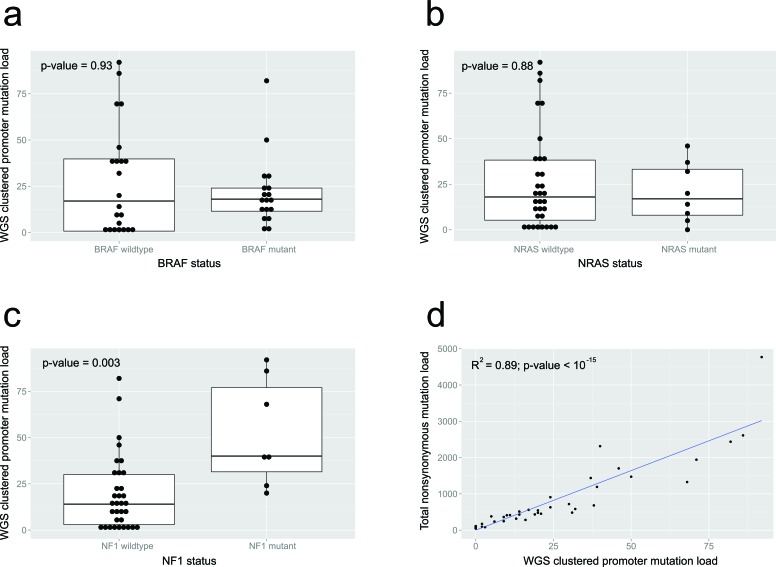
Clinicopathologic parameters from the 40 melanoma whole genome samples The difference in promoter (cluster) mutation load between mutant and wildtype cases for **a.** BRAF, **b.** NRAS and **c**. NF1 mutations are shown. **d**. shows the relationship between promoter (cluster) mutation load and nonsynonymous mutation load.

Linear regression demonstrates that there was significant linear correlation between WGS clustered promoter mutation load (the sum of all SNVs within regions from the heuristic promoter method per WGS sample) and overall non-synonymous mutation load (R^2^ = 0.89, *p*-value < 10^−15^; Figure 4d). There was no difference in WGS clustered promoter mutation loads with either *BRAF* (*p* = 0.93, Mann-Whitney U test; Figure 4a) or *NRAS* (*p* = 0.88, Mann-Whitney U test; Figure 4b) status, while tumors with mutated *NF1* had significantly higher loads (*p* = 0.003, Mann-Whitney U test; Figure 4c).

### Clustered mutations are independent of cell of origin

Finally, to assess whether these clustered mutations are melanoma-specific or not, we evaluated other publicly available exome data sets of different cutaneous tumor types for the mutations we identified in cluster windows in the 34 melanoma whole exomes. For this we examined 11 basal cell carcinoma (BCC) exomes from a study of vismodegib resistant BCCs [28]. Fifty one of the 77 promoter regions identified in the 34 melanoma exomes and validated by our multiplex panel had sufficient read depth for mutation calling.

**Figure 5:**
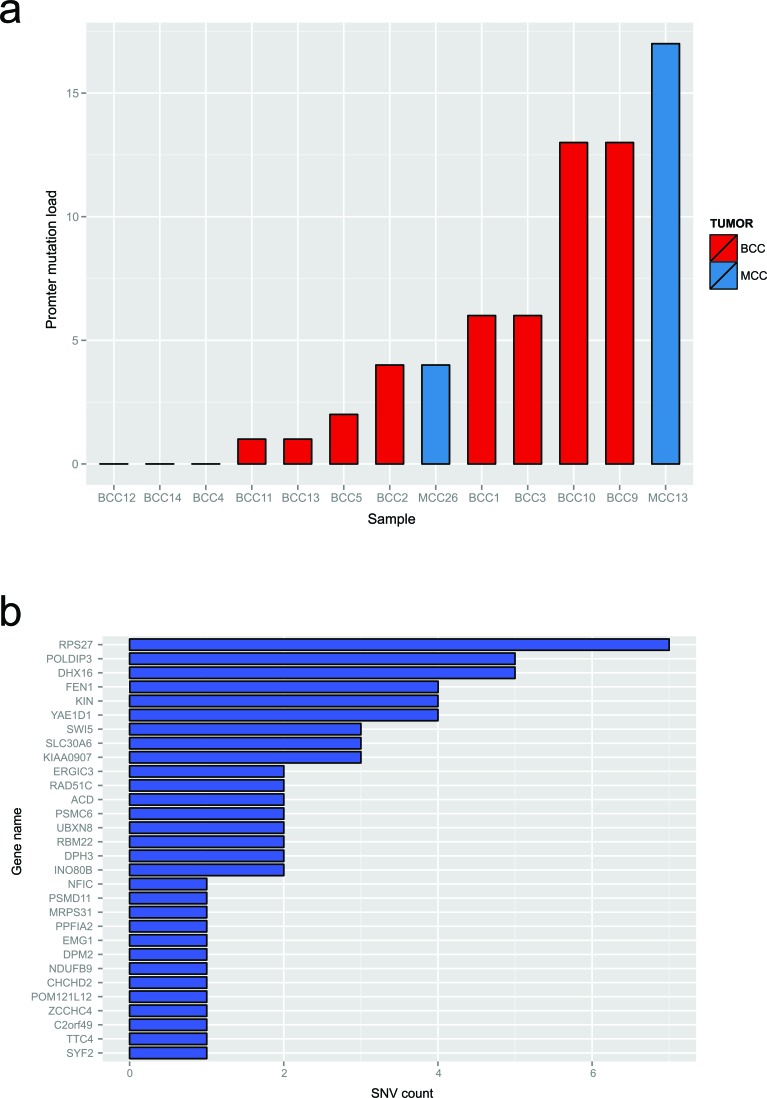
Merkel cell carcinoma and basal cell carcinomas possess the same type of mutation clusters as melanoma **a.** The total number of high stringency SNVs across BCC and MCC samples. **b.** The total number of SNVs called at annotated regions included in the multiplex PCR validation assay

Additionally, we evaluated two Merkel cell carcinoma cell lines, MCC13 and MCC26, with SNV data from COSMIC cell lines project. BCC and MCC samples had from 0 to 15 promoters with at least one previously called SNV, with 2 BCCs having a total of 13 mutated promoters and a single MCC cell line having fifteen mutated promoters (Figure 5). Examining the individual promoters involved showed that the most commonly mutated promoter was *RPS27*, with 7 cases having at least 1 mutation in this promoter. Given the lower read depth and reduced number of promoters available for analysis it is likely that we underestimate the number of mutated promoters; in spite of these limitations, some BCC and MCC samples demonstrate the same phenomenon as cutaneous melanoma.

Analysis of cutaneous squamous cell carcinoma (cSCC) data for the validated promoter mutations was limited given the lack of publically available data at the time of writing. However, evaluation of the recently described *NFKBIE* promoter [29] in cSCC was possible using the supplemental data of Pickering et al. [30], which revealed overlap of SNVs in an ETS motif and in a Sp1 motif with the previously published SNVs (Figure S4). Additionally the functional synonymous mutation described in *BCL2L12* (located at chr19:50169131) [31] is present within the 34 melanoma exomes, 170 clinical melanoma samples and within the cSCC data set (Figure S5). Of note these occur within an ETS-like motif, within a DHS peak, within a site with multiple transcription factor ChIP-seq peaks and occur as a dinucleotide change in two samples; moreover the site is annotated within the 5′UTR of another gene, *IRF3*. Therefore cSCC displays the same phenomenon as demonstrated in the above cutaneous malignancies in at least two sites. However further studies are warranted to confirm this.

## DISCUSSION

In this study, we demonstrate strong support for the role of chronic ultraviolet radiation as the etiologic agent of the mutation clusters that we observed. Firstly, the spectrum of mutation is typical of a UV signature with elevated C→T and especially CC→TT transitions at dipyrimidines. Secondly, melanomas arising in sun-exposed anatomical regions have higher numbers of these mutations. This is supported by the higher number of mutations in melanomas with increasingly severe solar elastosis scores in adjacent dermis. Thirdly, the same clustered mutations are present in at least two other cutaneous malignancies, both with strong evidence for a UV etiology. We therefore claim that ultraviolet radiation is the critical mutagen in the genesis of these proximal promoter mutations.

Using orthogonal methods, we discovered frequent recurrent cluster mutations in cutaneous melanoma and other cutaneous malignancies located within proximal promoter regions. Mutations were defined by positions within and flanking transcription factor motifs for ETS and Sp1, rather than recurrent single base positions. With the clustering of mutations around ETS motifs, we can categorize previously disparate observations under a single conceptual framework as recurrent mutations identified previously in *DPH3* [24], *SDHD* [23], *NDUFB9* [25], *RPS27* [32] and *MRPS31* [33] all conform broadly to this pattern. Moreover, the oncogenic synonymous mutation reported in *BCL2L12* [31] may actually be mutations within the ETS sequence of the proximal promoter of the neighboring gene, *IRF3* (Figure S5, Table S1); indeed *BCL2L12*/*IRF3* mutations show the same features as identified in other mutation clusters, i.e. presence within a DHS peak and over multiple transcription factor ChIP-seq peaks. Furthermore, the *NFKBIE* promoter region analyzed by Shain et al. [29] is of a very similar structure to the regions identified in this paper. Of note, the mutations cluster in the ETS motif at a higher frequency than in the Sp1-like motif adjacent to it (Figure S4). This is consistent with our demonstration that Sp1 motifs contain clusters of somatic mutations, albeit at overall lower rates than ETS motifs, and with a different pattern across positions within the motif (Figure 1c).

The identification of the ETS motif within clustered mutation regions is intriguing given that this is the same motif implicated in the *TERT* promoter mutations; however, while it is recognized that *TERT* promoter mutations create an ETS binding site, the majority of mutations within clusters identified in this study would be predicted to mainly either ablate the motif by mutating the guanosine at positions +1 or +2, or potentially modulate it by mutating a flanking base. The lack of association of promoter mutation with changes in local gene expression, the fact these mutation occur at lower frequencies and across several base positions, and the fact that these alter pre-existing ETS sequence suggest that *TERT* promoter mutations may be a fundamentally different mutational class from the other ETS and Sp1 clustered mutations that we have uncovered in this study.

Our analysis of gene expression data failed to demonstrate any difference in local gene expression between promoter-mutated samples and promoter-wildtype samples for a given gene, including *TERT*, which is consistent with the study of Fredriksson et al. [24] for the set of melanoma samples. Given many of these mutations occur within defined positions of canonical transcription factor motifs within annotated proximal promoters, the absence of any evidence of gene expression differences is surprising and weighs against a role for these mutations as oncogenic drivers and instead supports a passenger role for most of these mutations. Our study, however, does not exclude the existence of context dependent, or transient changes in gene expression caused by these mutations—indeed, the study of Chiba et al. [22] demonstrated that the effects of *TERT* promoter mutations were most critical during cell differentiation, and it is possible that the mutation clusters identified in this study may similarly only manifest their effects at critical junctures of carcinogenesis thereby increasing the likelihood of survival of the tumor cells.

As demonstration of the validity of our approach to finding clusters, several of the regions detected by our hotspot method were present within the supplementary data for Fredriksson et al. [24] and Weinhold et al. [23], and only within the cutaneous i.e sun-exposed melanoma samples they obtained from the TCGA. By expanding our analysis to cancer sets outside of the TCGA to include exome data from basal cell carcinoma samples and Merkel Cell Carcinoma cell lines, we demonstrated that many of these hotspot mutations are found in other UV-related cutaneous malignancies; consistent with this observation, a recent study evaluating *DPH3* promoter mutations in non-melanoma skin cancer found a high frequency in both BCC and squamous cell carcinoma [34].

As these mutations (apart from *TERT* promoter) do not apparently alter local gene expression, and that these mutations do not show any cell lineage specificity, it is possible that they represent passenger mutations acquired during the pre-malignant life of the cell of origin under chronic UV exposure. This would be consistent with the high burden of oncogenic mutations in grossly unremarkable skin obtained from facial tissue specimens [35]. The recent study of the genetic evolution of melanocytic lesions [36] demonstrated the *TERT* promoter mutations are acquired early in tumor evolution, and we would predict other clustered promoter mutations would also be acquired prior to invasive transformation.

Somatic mutations are a result of an imbalance between a mutagenic process and the DNA repair machinery activated to eliminate it. We have demonstrated that the most likely mutagenic agent of these clustered mutations is UV. In humans, which like other placental mammals lack a photolyase enzyme, the bulky DNA adducts (CPDs and (6-4)PPs) from UV damage are repaired exclusively by nucleotide excision repair (NER), and therefore this pathway may also be involved in this phenomenon. Indeed Polak et al. have ascribed a role for NER within promoters whereby repair leads to a reduced somatic mutation load in gene promoters in melanoma and other cancers [37].

Previous studies have demonstrated that NER efficiency is reduced at the proximal promoter of several specific genes [38–41], most likely due to the direct physical association of transcription factors at these sites. Meier et al. demonstrated that UV damage repair within rRNA promoters of yeast was inhibited by the assembled transcriptional machinery, and that the repair defect was more profound with increasing gene expression [42]; however given that yeast utilizes a photolyase repair pathway, this finding may not be applicable to human cells [43]. Recently two studies have demonstrated increased mutational frequency within promoter regions, and by using high throughput data from a method that quantified NER activity called XR-seq [44], they both revealed that NER was inhibited within transcription factor binding sites [45, 46]. Our study independently confirms the findings of both these studies, and expands on them by showing the specific motifs which are hypermutated in gene promoters. The observation of genome-wide NER deficiency in gene promoters, along with data demonstrating that UV footprinting occurs preferentially at specific sites of bound transcription factors [47, 48], suggest that the direct physical association of specific transcription factors, such as members of the ETS and Sp1 families, with these sites in the setting of chronic UV initiated mutagenesis result in the formation of these mutations.

The reason why these mutations occur most frequently within the ETS motif in the proximal promoter remains unclear, especially given that several other transcription factor motifs contain more guanosines which could be somatically mutated by UV. It is tantalizing that the ETS protein that is likely responsible for the functional effects of *TERT* promoter mutations is GABPA [49], which is the transcription factor with the most similar motif to the one detected in the whole genome mutation cluster windows. Future studies will be required to assess whether GABPA is responsible for promoter hypermutation across the genome and its functional role during chronic UV irradiation.

In conclusion, for the first time we have identified recurrent clustered somatic mutations in the proximal promoters of a number of genes, with preferential mutation of specific bases in ETS and Sp1 binding motifs. We demonstrated that these mutations show features consistent with origin from chronic ultraviolet irradiation, and consistent with this, are present in multiple types of cutaneous malignancies arising from distinct cells of origin. These mutation clusters represent a novel signature of ultraviolet mutagenesis, and implicate specific transcription factor families playing a role in a chronic UV irradiation response. Their frequency and recurrence suggest either a selective mechanism was active at some point during tumor development, or these were acquired very early in tumor development. Future investigations should shed light on the nature of the transcriptional response to chronic ultraviolet irradiation and the timing of the acquisition of these promoter mutations.

## MATERIALS AND METHODS

### Sample selection for melanoma clinical samples

Clinical melanoma tumor samples were provided by the Melbourne Melanoma Project from stored DNA in the Peter MacCallum Cancer Centre. Matched normal blood was obtained from the Victorian Cancer Biobank (*n* = 93). All patients gave informed consent and ethics approval was obtained from the Peter MacCallum Cancer Centre Ethics Committee for all human tissues and clinicopathologic data used in this study. Samples were all primary cutaneous melanomas and DNA was obtained from a representative formalin fixed paraffin embedded block by scraping pathologist-identified tumor regions from methyl green stained sections. Tumor and normal DNA were extracted using the Qiagen QIAamp DNA Blood Mini Kit. 233 samples were chosen for sequencing and 170 were suitable for analysis by our criteria (see below).

### Melanoma whole genome and exome data

Whole exome data for the 34 primary cutaneous melanomas were obtained from [1] in BAM format. As previously described, reads were previously aligned to the reference human genome (hg19) using the Burrows-Wheeler Aligner. Single nucleotide variant calling was performed using MuTect 1.1.5 [50] (Broad Institute, Cambridge, MA) with default settings, and all calls marked as PASS were included in analysis.

Whole genome data for 40 melanomas in BAM format was downloaded from the TCGA via CGhub (The Cancer Genome Atlas Research Network, National Cancer Institute and National Human Genome Research Institute, Bethesda, MD, USA. dbGaP study accession: phs000178.v9.p8) [51]. For the two patients with both primary and metastatic samples sequenced, we used the data from the primary lesion. Single nucleotide variant calling was performed using MuTect with default settings, and all calls marked as PASS were included in analysis.

Basal cell carcinoma data was downloaded from the Gene Expression Omnibus (project GSE58374) (from [28]) and converted from SRA format to fastq format. Reads were aligned and variants called as above for the melanoma whole exome samples.

Merkel cell line data from MCC13 and MCC26 were downloaded from the COSMIC cell line project (http://grch37-cancer.sanger.ac.uk/cell_lines).

Cutaneous squamous cell carcinoma [30] and additional melanoma [29, 31] SNV data were obtained from the supplementary tables of the respective published articles.

DNase I hypersensitivity (DHS) and transcription factor binding site .bed files were downloaded from UCSC table browser (accessed on 13 June 2015). DHS data was obtained for the Melano cell line, as well as the composite track using multiple cell lines wgEncodeRegDnaseClusteredV3. Transcription factor binding site data was taken from TxnFacChIPV2. Gene expression data for genes with clustered promoter mutations in the melanoma whole genome set were obtained from cBioportal using the *CDGS-R* package. Differential expression between promoter mutant and promoter wild type cases was evaluated using the nonparametric Mann-Whitney U test. Correction for multiple testing was performed using the Benjamini-Hochberg method as utilized in R. A q-value less than 0.05 was considered significant.

Annotation of transcripts was performed using the TxDb.Hsapiens.UCSC.hg19.knownGene R package, which utilizes RefSeq annotation data.

All analyses were performed using hg19 as the reference genome.

### Initial cluster analysis of mutation data

For whole exome data, a sliding window approach was applied with a window of width 5 base pairs (bp). The coordinates of all windows containing 4 or more SNVs (i.e. approximately 10% of samples) across all samples were recorded. In order to reduce false positives from misaligned reads, we filtered all mutation calls using the ENCODE Duke Mapability 20bp Uniqueness track (wgEncodeDukeMapabilityUniqueness20bp from the UCSC table browser; https://genome.ucsc.edu/cgi-bin/hgTables [52]). Only those mutations in regions with score equal to one were included.

For whole genome data, we set a scanning window of 15 bp (the size of two adjacent motifs) and counted all those with 4 or more SNVs, this threshold having been determined by using a Poisson binomial test as implemented in the *poibin* package in R. The mutation rate was estimated for each sample by dividing the total number of SNVs by 3×10^9^ (approximate length of the human genome) and multiplying by the window size of 15bp. The probability of having 4 or more mutations was less than 10^−6^.

We used the reduce function from the GRanges package to join overlapping windows. These windows were subsetted by using the wgEncodeRegDnaseClusteredV3 coordinates with 50bp flanks, followed by subsetting by regions covered by at least three ENCODE ChIP-seq peaks from the TxnFacChIPV2 data.

Analysis of mutation clusters was performed using R/Bioconductor using the *GenomicRanges* and *VariantAnnotation* packages. Statistical testing was performed in R.

### Motif prediction

The coordinates of the identified hotspot windows in the 34 exomes and 40 whole genomes were used for sequence extraction after adding 10bp flanks. Sequence alignment and motif analysis was performed using the MEME suite (MEME version 4.10.2; http://meme-suite.org) [53]. The motif matching was performed using TOMTOM to the HOCOMOCO v9 database of human transcription factor binding site motifs [54, 55].

### Unbiased, background-corrected genome-wide cluster analysis

To overcome potential sources of bias in the initial cluster analysis of the whole genome data, we developed a novel approach to find statistically significant clusters of SNVs of the merged TCGA whole genome data (Figure 2a). We merged SNV data between patients and scanned each chromosome, computing the distance spanned by every group of four adjacent SNVs. We call such groups of SNVs “4-scans”, and the number of nucleotides each 4-scan spans its “width”. Four-scans are not disjoint, but may overlap. To correct for the heterogeneity in background mutation rate across the genome, we computed the average number of SNVs across all samples in sliding 1Mb windows. We filtered out 4-scans in DAC blacklisted regions and that were not uniquely-mappable with a 100-mer. We then regressed the 0.5-offset log-transformed 4-scan widths against the log-transformed SNV count per megabase and its square, plus an intercept term. The resulting residuals have an approximate normal distribution (Figure S10), from which we calculated p-values, and q-values using Benjamini-Hochberg FDR correction for each 4-scan. Overlapping 4-scans with q < 0.01 were merged into hotspots. Analysis was performed in R.

Hotspots were compared with a restricted, proximal promoter-enriched subset obtained by applying the same filters as in the heuristic promoter definition applied to the whole genome samples: each hotspot in the restricted set was required to overlap a uniquely mappable region from the ENCODE Duke Mapability 20bp Uniqueness track; to be within 50bp of a wgEncodeRegDnaseClusteredV3 site; and to be covered by at least three ENCODE ChIP-seq peaks from the TxnFacChIPV2 data. We looked for recurrent motifs in the restricted subset of hotspots using MEME.

To find motifs in other annotated regions, we used the core 15 state model from the Roadmap Epigenomics Project (http://www.roadmapepigenomics.org/) from the two melanocyte cell lines (E059 and E061) to annotate the different chromatin states in the whole genomes. We applied the MEME motif finding algorithm on those clusters present in each annotated region (Table S5).

### Confirmation of mutations by HRM and Sanger sequencing

High resolution melting analysis of 93 melanoma samples for *YAE1D1* mutation was performed using the technique published in Hondow et al. [56]. HRM was performed using a Roche Lightcycler 480 with proprietary software. The primers used were:

Forward: AGCCTCCACTCGCCGTCTTC

Reverse: ACATCACCGAGGCAATTACGG

Sanger sequencing was performed as per the method in Mar et al. [1] using the reverse primer above.

### Custom mutation panel screening and base calling

A custom mutation panel was designed which utilized the Fluidigm Access Array platform for multiplex PCR followed by next generation sequencing on an Illumina MiSeq. The 98 cluster windows from the 34 whole melanoma exomes were used for designing amplicons of approximately 150bp width. We used Primer3 for primer design (version 0.4.0, http://bioinfo.ut.ee/primer3-0.4.0/) [57, 58] and excluded any windows in which unique primers could not be created. Primer sequences for the 77 amplicons are given in Table S2. After PCR, sequencing barcodes were attached to products to permit MiSeq sequencing. Alignment was performed using an in-house custom algorithm for amplicons as described in [59].

Cases were initially selected by i) the availability of adequate DNA volume (12 μL) and ii) DNA concentration greater than 1ng/μL, as measured by Qubit analysis (ThermoFisher Scientific, Massachusetts, USA) using standard protocol. This resulted in 233 samples proceeding to analysis. Samples were run in duplicate with 4μL loaded for each. After alignment, samples were excluded if more than 30 amplicons had less than 100 reads in either of the duplicates, or both duplicates had more than 20 amplicons with less than 100 reads. After exclusion 170 cases were suitable for analysis.

Mutation calls were only made for SNVs if all three of the following were satisfied: 1) Read depth was greater than 50; 2) SNV was present in both duplicates or in single sample with allelic fraction greater than 0.1; 3) SNV was present in the mutation data from the 34 exomes.

### Clinical data and histopathology

Patient information such as age, gender, date of surgery, date of birth, tumor stage and site of melanoma as well as histologic variables including subtype, Breslow thickness and ulceration were collected. Melanoma subtype was based on the World Health Organisation (WHO) criteria [60]. Solar elastosis was evaluated for all samples with available slides and adjacent dermis (131/170) using the method of Weyers et al. [61]. Solar elastosis measurement was performed by a pathologist (AC) blinded to case ID number. Analysis of clinicopathologic variables was performed using a Mann-Whitney U test for two groups (mutation status, ulceration, gender) and a Kruskal-Wallis test for more than two groups (Breslow thickness, stage, anatomical location, subtype). Spearman correlation was used to analyze association between promoter mutation load and continuous variables such as age and non-synonymous mutation load. Linear regression of non-synonymous mutation load and cluster mutation load was performed in R.

### Availability of data and materials

The melanoma whole genome, melanoma whole exome, BCC, cSCC and MCC datasets supporting the conclusions of this article are publically available and are described above. The remaining datasets supporting the conclusions of this article are included within the article and supplementary material.

## SUPPLEMENTARY MATERIAL FIGURES AND TABLES














