# Supplementary material for: Clustered somatic mutations are frequent in transcription factor binding motifs within proximal promoter regions in melanoma and other cutaneous malignancies
Source: Oncotarget. 2016 Sep 7;7(41):66569–85. doi: 10.18632/oncotarget.11892 (PMC5341821; doi:10.18632/oncotarget.11892)
Supplement: Supplementary file 1 [file oncotarget-07-66569-s001.pdf]

Clustered somatic mutations are frequent in transcription factor binding motifs within proximal promoter regions in melanoma and other cutaneous malignancies

Supplementary Material

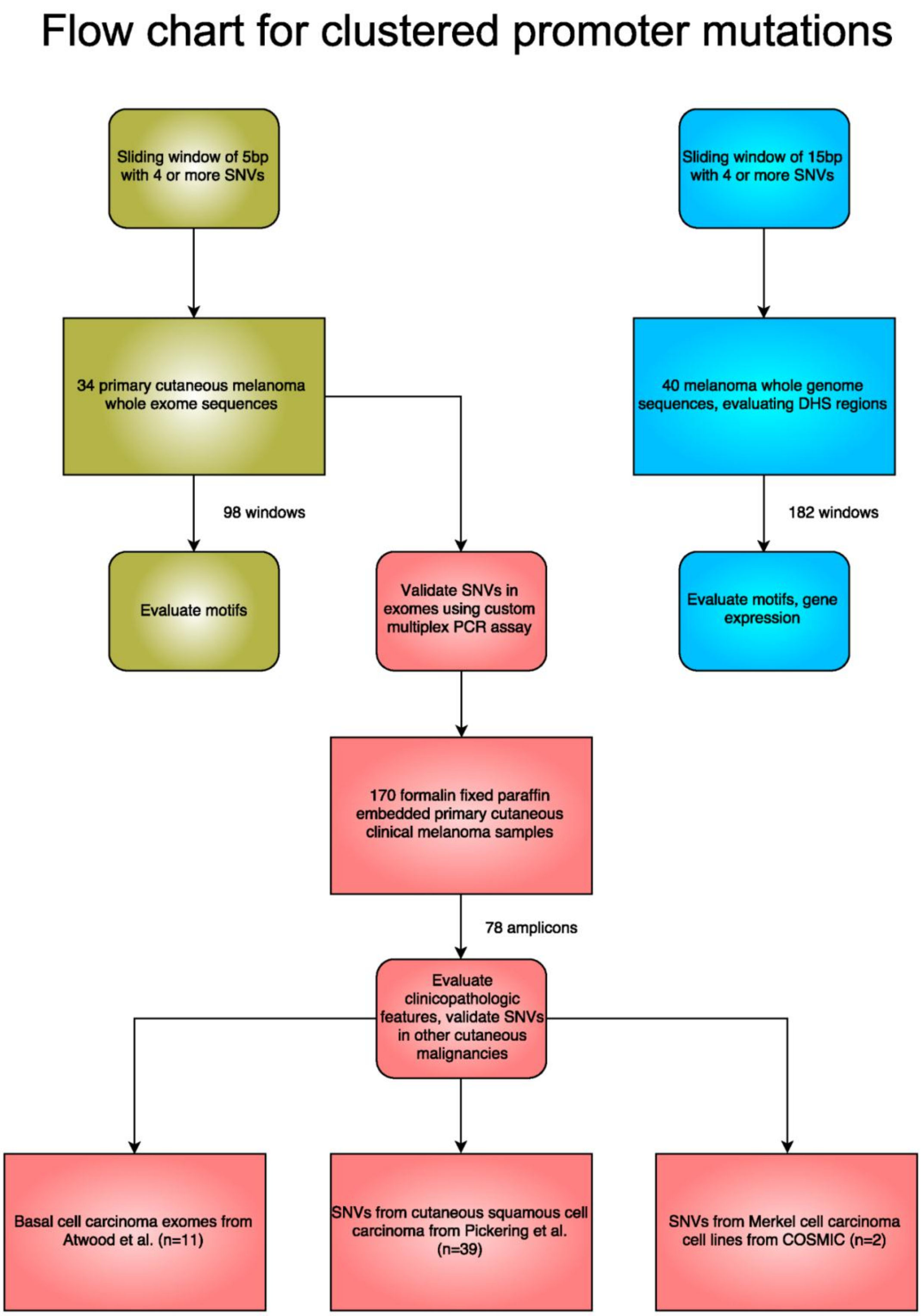

Figure S1: Flowchart of the initial heuristic discovery, validation and analysis of proximal promoter SNV clusters.

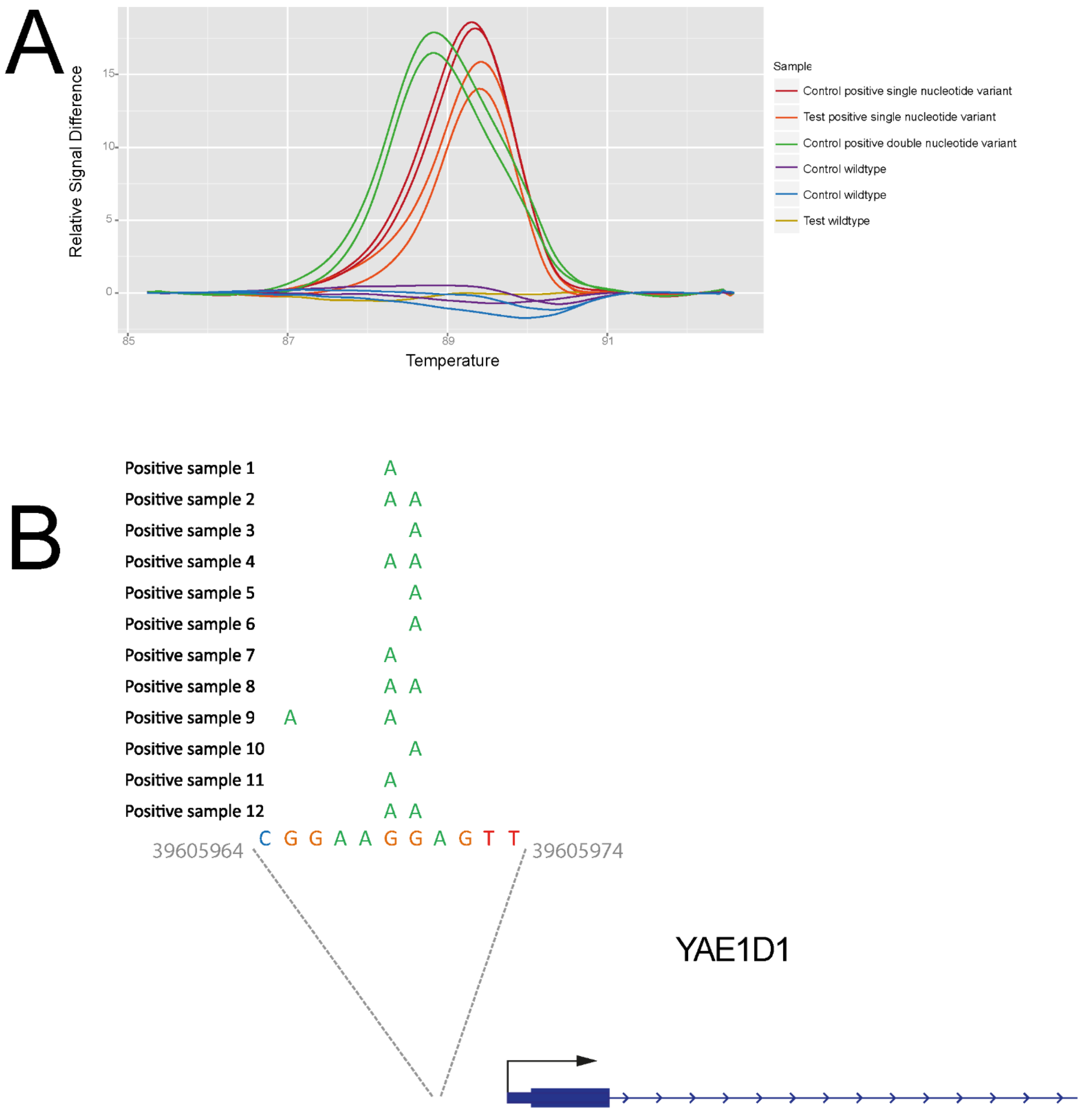

Figure S2: High resolution melt analysis and sequencing of the *YAE1D1* promoter. **a.** Example of the high resolution melt (HRM) curves for *YAE1D1* promoter variants. Shown are the melt curves for two known mutation positive samples, two known wildtype samples (all from the 34 whole exome sequenced melanoma series), one unknown FFPE sample which appeared mutated and one unknown FFPE sample which appeared wildtype, both confirmed on subsequent Sanger sequencing. Temperature is in degrees Celsius. **b.** SNVs present across 12 melanoma samples in the *YAE1D1* promoter.

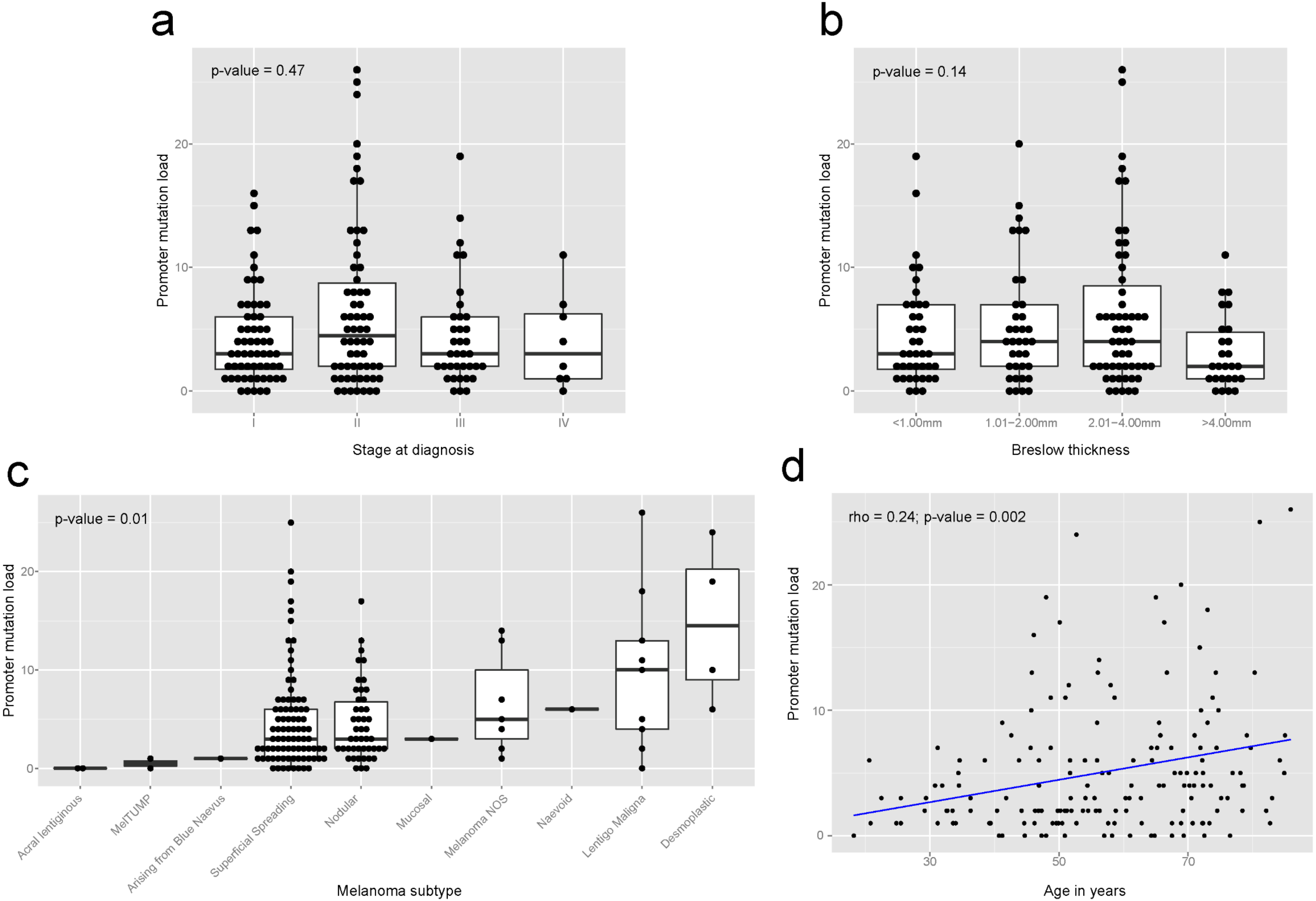

Figure S3: Further clinicopathologic correlates of promoter mutation load (defined from all high stringency SNVs from the multiplex PCR validation assay within a sample) to a) clinical stage at diagnosis, b) Breslow thickness, c) WHO melanoma subtype and d) age at time of diagnosis in days.

NFKBIE

chr6: 44,233,345 - 44,233,463

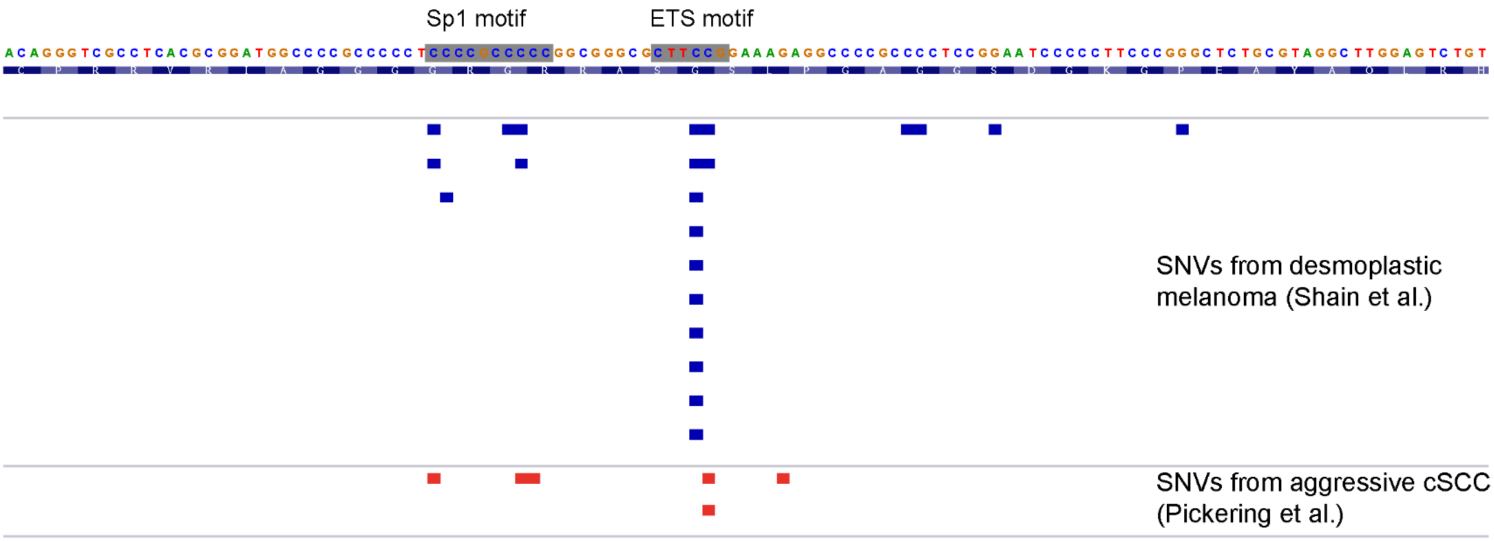

Figure S4: The proximal promoter of *NFKBIE* identified as frequently mutated in desmoplastic melanoma [29] is also mutated in the same positions across a set of aggressive cSCCs [30] within motifs (marked in grey) that match those identified in the clustered mutation regions of the 40 melanoma whole genome samples. The motif to the left matches the Sp1 signature while the one on the right matches the ETS signature.

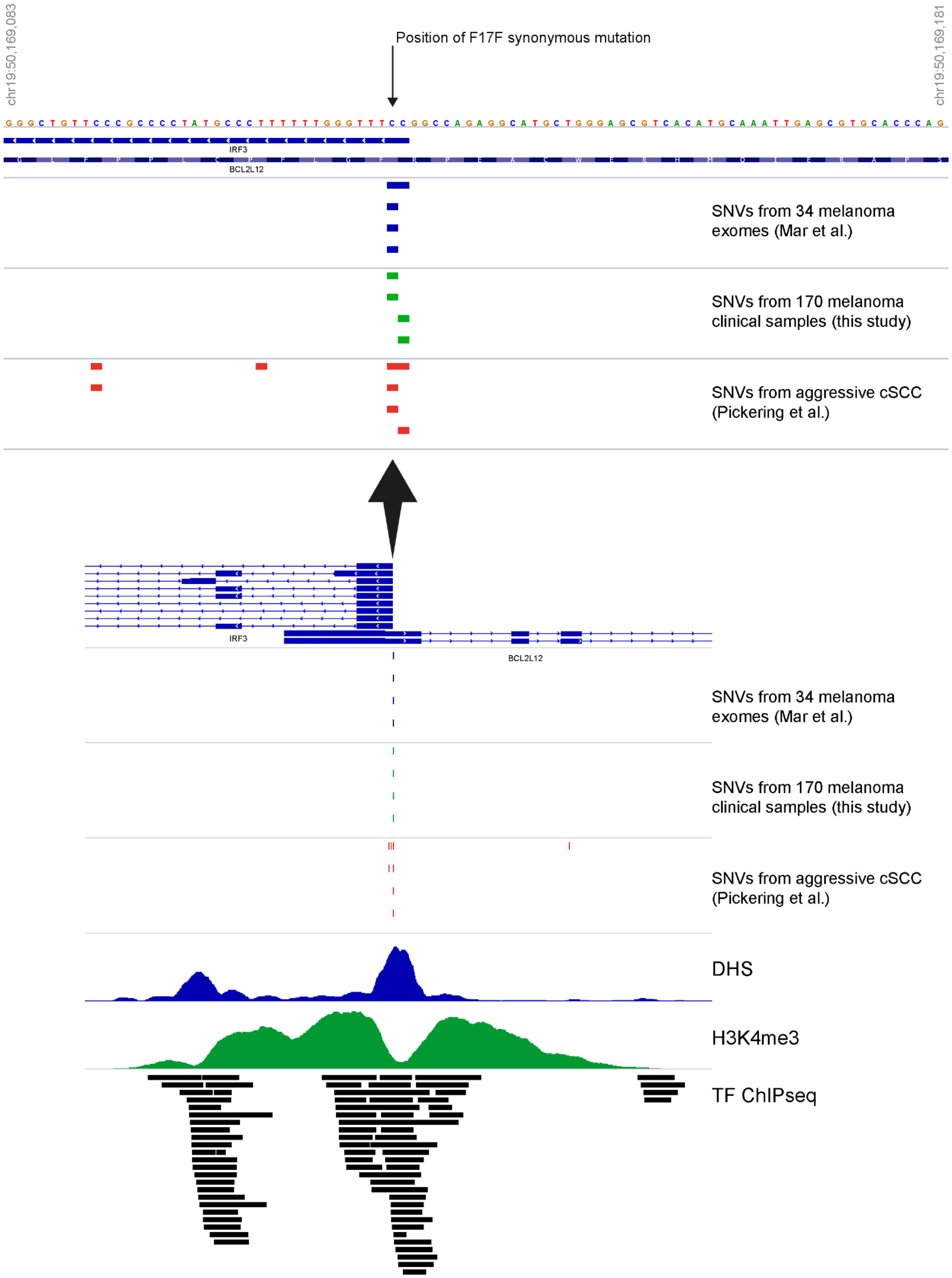

Figure S5: The proximal promoter of BCL2L12/IRF3 is shown in the region of the previously described synonymous mutation F17F [31], with SNVs detected in the whole exome sequences from 34 primary melanoma samples [1], the multiplex amplicon sequences from 170 clinical melanoma cases and the whole exome sequences of a set of aggressive cSCCs [30]. The SNVs at this region closely resemble the pattern found at other typical clustered promoter mutation windows.

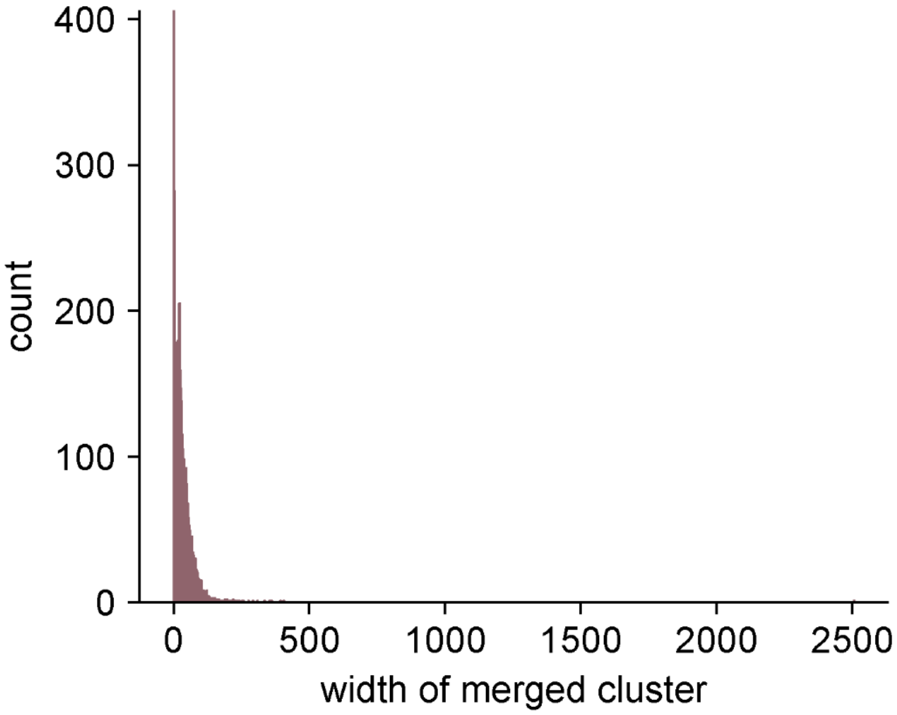

Figure S6: Histogram of hotspot widths following the merging of significant 4-scans in the unbiased, background-corrected search.

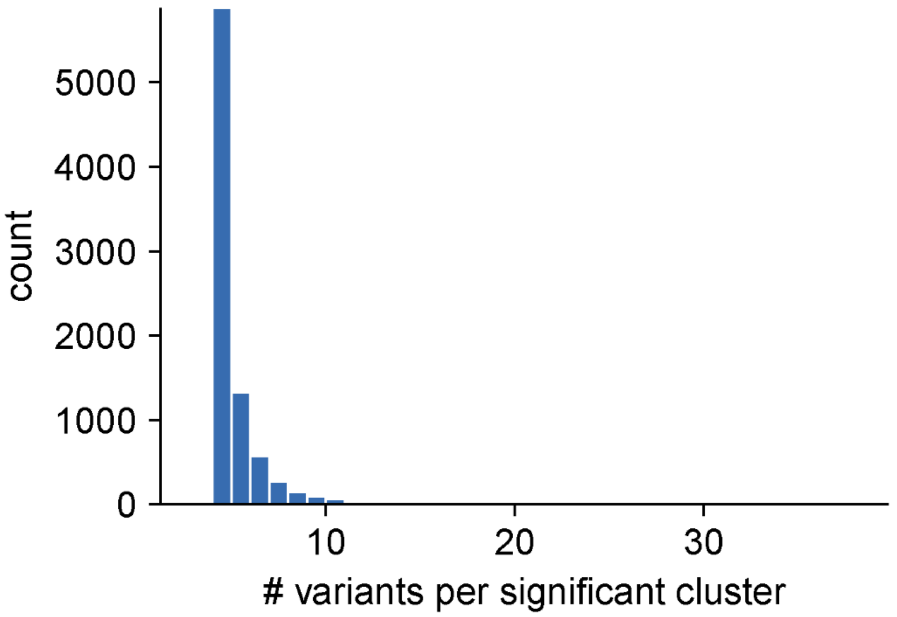

Figure S7: Histogram of variants per hotspot in the background-corrected search.

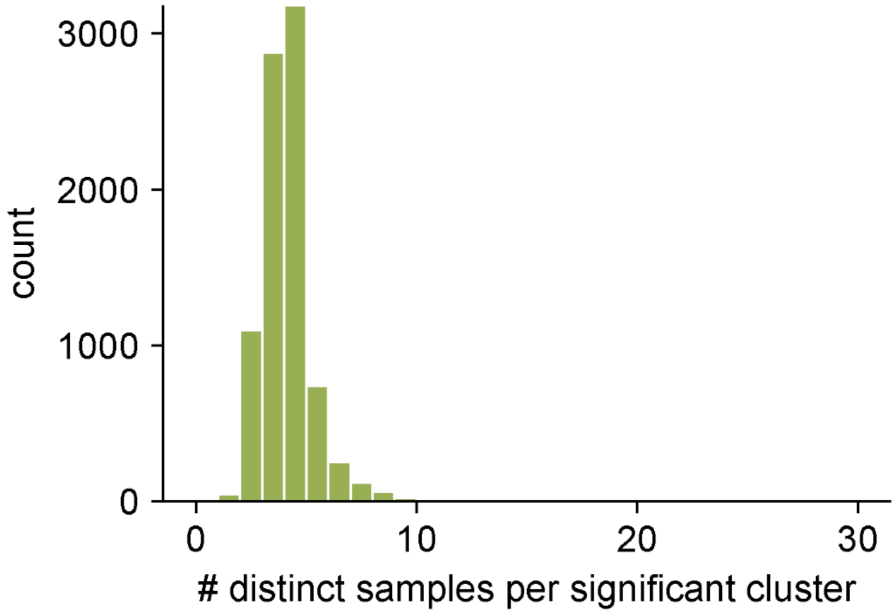

Figure S8: Histogram of the number of distinct samples represented in each hotspot in the background-corrected search.

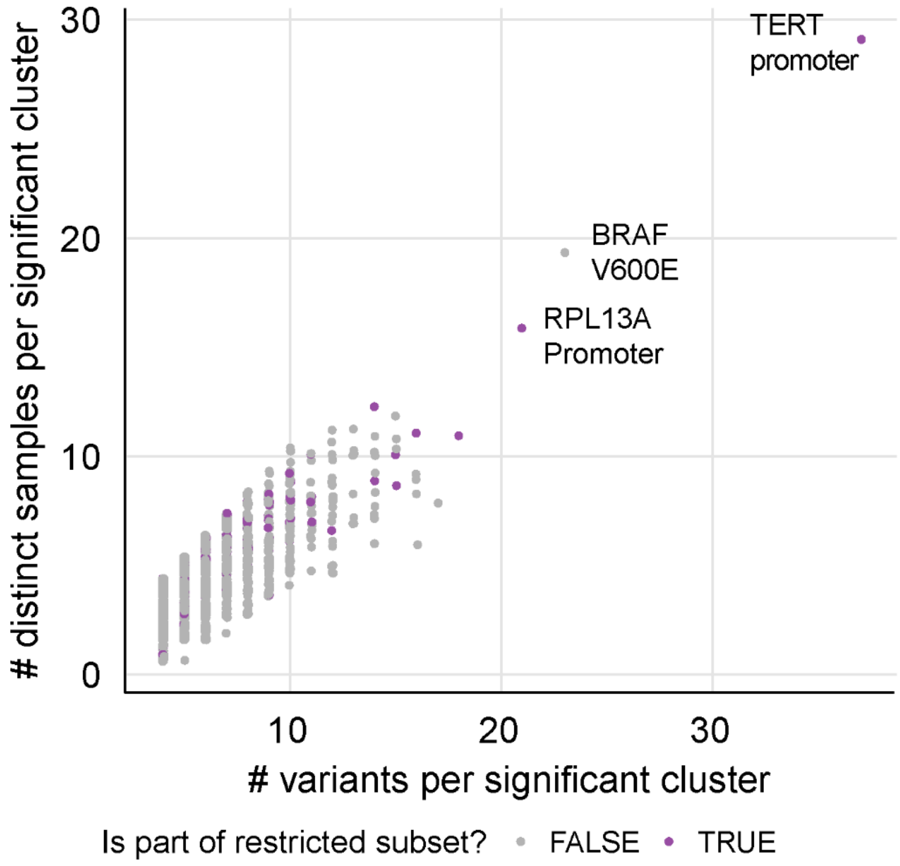

Figure S9: Scatter of the number of variants versus the number of distinct samples per hotspot in the unbiased search. Purple points represent the restricted, promoter-enriched subset of hotspots corresponding to the filtering in the initial search.

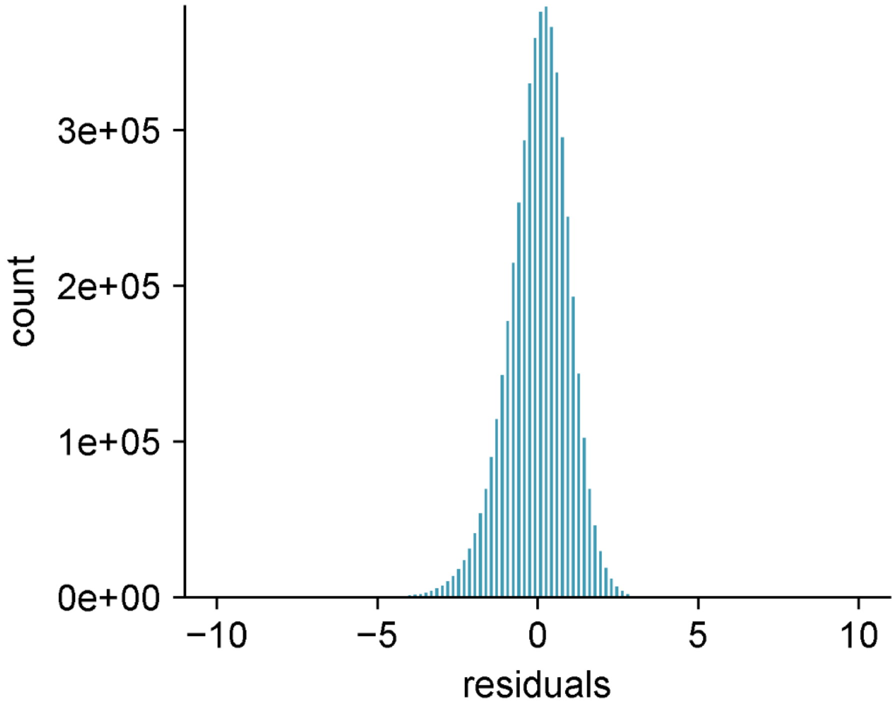

Figure S10: Histogram of regression residuals. The distribution is approximately normal, though with an apparent left-skew. Since the left tail of the empirical distribution is fatter than that of the theoretical, our method may be more liberal than the nominal FDR threshold suggests.

Supplemental Tables

Table S1. Annotated mutation clusters from the 34 whole exome samples.

Table S2. Primer sequences from the custom gene sequencing panel.

Table S3. 182 cluster window regions from WGS.

Table S4. SNV data from the validation set of 170 clinical melanoma samples.

Table S5. The frequency and percentage of hotspots in each of the putative chromatin states, for both melanocyte foreskin cell lines (E059 and E061), with enhancer states merged.

Table S6. Table of all hotspots detected by the unbiased, background-corrected genome-wide cluster analysis

For Table S1-S6, please see the attached word files.
