# Supplementary material for: Clustered somatic mutations are frequent in transcription factor binding motifs within proximal promoter regions in melanoma and other cutaneous malignancies
Source: Oncotarget. 2016 Sep 7;7(41):66569–85. doi: 10.18632/oncotarget.11892 (PMC5341821; doi:10.18632/oncotarget.11892)
Supplement: Supplementary file 2 [file oncotarget-07-66569-s002.docx]

**Table S1. Annotated mutation clusters from the 34 whole exome samples.**

| CHROM | START | END | LOCATION | GENE SYMBOL |
| --- | --- | --- | --- | --- |
| chr1 | 894682 | 894687 | promoter | NOC2L |
| chr1 | 894682 | 894687 | promoter | KLHL17 |
| chr1 | 25559063 | 25559068 | promoter | SYF2 |
| chr1 | 55181528 | 55181533 | intron | MROH7-TTC4 |
| chr1 | 55181528 | 55181533 | fiveUTR | TTC4 |
| chr1 | 55181528 | 55181533 | promoter | TTC4 |
| chr1 | 115256529 | 115256534 | coding | NRAS |
| chr1 | 153276253 | 153276258 | intron | PGLYRP3 |
| chr1 | 153963222 | 153963227 | promoter | RPS27 |
| chr1 | 153963239 | 153963244 | fiveUTR | RPS27 |
| chr1 | 153963239 | 153963244 | promoter | RPS27 |
| chr1 | 155904246 | 155904251 | promoter | KIAA0907 |
| chr2 | 10830114 | 10830119 | promoter | NOL10 |
| chr2 | 32390904 | 32390909 | promoter | SLC30A6 |
| chr2 | 74682175 | 74682180 | fiveUTR | INO80B |
| chr2 | 74682175 | 74682180 | promoter | INO80B |
| chr2 | 74682175 | 74682180 | promoter | INO80B-WBP1 |
| chr2 | 105953996 | 105954001 | promoter | C2orf49 |
| chr2 | 168098326 | 168098331 | coding | XIRP2 |
| chr3 | 16306504 | 16306509 | promoter | OXNAD1 |
| chr3 | 16306504 | 16306509 | promoter | DPH3 |
| chr3 | 48481602 | 48481607 | promoter | TMA7 |
| chr3 | 48481602 | 48481607 | promoter | CCDC51 |
| chr3 | 52542194 | 52542199 | intron | STAB1 |
| chr3 | 67048644 | 67048649 | promoter | KBTBD8 |
| chr3 | 68802215 | 68802220 | intron | FAM19A4 |
| chr3 | 124449234 | 124449239 | fiveUTR | UMPS |
| chr3 | 124449234 | 124449239 | promoter | UMPS |
| chr4 | 25314329 | 25314334 | promoter | ZCCHC4 |
| chr4 | 53611041 | 53611046 | coding | ERVMER34-1 |
| chr4 | 69093838 | 69093843 | intron | TMPRSS11B |
| chr4 | 152020700 | 152020705 | promoter | RPS3A |
| chr4 | 190878494 | 190878499 | intron | FRG1 |
| chr5 | 26885698 | 26885703 | intron | CDH9 |
| chr5 | 145826780 | 145826785 | promoter | TCERG1 |
| chr5 | 150080667 | 150080672 | promoter | RBM22 |
| chr6 | 397027 | 397032 | intron | IRF4 |
| chr6 | 397027 | 397032 | promoter | IRF4 |
| chr6 | 30640795 | 30640800 | fiveUTR | DHX16 |
| chr6 | 30640795 | 30640800 | promoter | DHX16 |
| chr6 | 56047428 | 56047433 | fiveUTR | COL21A1 |
| chr7 | 39605965 | 39605970 | promoter | YAE1D1 |
| chr7 | 53103781 | 53103786 | coding | POM121L12 |
| chr7 | 56174180 | 56174185 | intron | PSPH |
| chr7 | 56174180 | 56174185 | fiveUTR | CHCHD2 |
| chr7 | 56174180 | 56174185 | promoter | CHCHD2 |
| chr7 | 72419616 | 72419621 | threeUTR | POM121 |
| chr7 | 72419616 | 72419621 | intron | NSUN5P2 |
| chr7 | 72419616 | 72419621 | coding | NSUN5P2 |
| chr7 | 76142329 | 76142334 | intron | UPK3B |
| chr7 | 140453136 | 140453141 | coding | BRAF |
| chr7 | 152497540 | 152497545 | intron | ACTR3B |
| chr8 | 30601668 | 30601673 | promoter | UBXN8 |
| chr8 | 40389560 | 40389565 | threeUTR | ZMAT4 |
| chr8 | 68931943 | 68931948 | intron | PREX2 |
| chr8 | 125551344 | 125551349 | fiveUTR | NDUFB9 |
| chr8 | 125551344 | 125551349 | promoter | NDUFB9 |
| chr8 | 125551344 | 125551349 | promoter | TATDN1 |
| chr10 | 7830002 | 7830007 | promoter | ATP5C1 |
| chr10 | 7830002 | 7830007 | promoter | KIN |
| chr10 | 127512080 | 127512085 | promoter | BCCIP |
| chr10 | 127512080 | 127512085 | promoter | UROS |
| chr11 | 1093364 | 1093369 | coding | MUC2 |
| chr11 | 8704334 | 8704339 | fiveUTR | RPL27A |
| chr11 | 8704334 | 8704339 | promoter | SNORA45A |
| chr11 | 46958261 | 46958266 | fiveUTR | C11orf49 |
| chr11 | 46958261 | 46958266 | promoter | C11orf49 |
| chr11 | 61560107 | 61560112 | promoter | FEN1 |
| chr11 | 61560107 | 61560112 | promoter | TMEM258 |
| chr11 | 61560107 | 61560112 | promoter | MIR611 |
| chr11 | 62414149 | 62414154 | fiveUTR | GANAB |
| chr11 | 62414149 | 62414154 | promoter | GANAB |
| chr11 | 65063342 | 65063347 | coding | POLA2 |
| chr11 | 99932099 | 99932104 | coding | CNTN5 |
| chr12 | 7080016 | 7080021 | promoter | EMG1 |
| chr12 | 7080016 | 7080021 | fiveUTR | EMG1 |
| chr12 | 7080016 | 7080021 | promoter | PHB2 |
| chr12 | 34175405 | 34175410 | fiveUTR | ALG10 |
| chr12 | 34175405 | 34175410 | promoter | ALG10 |
| chr12 | 53473215 | 53473220 | promoter | SPRYD3 |
| chr12 | 81693129 | 81693134 | coding | PPFIA2 |
| chr12 | 81693129 | 81693134 | threeUTR | PPFIA2 |
| chr12 | 81693129 | 81693134 | intron | PPFIA2 |
| chr12 | 117923348 | 117923353 | intron | KSR2 |
| chr12 | 132537869 | 132537874 | intron | EP400 |
| chr13 | 41345346 | 41345351 | promoter | MRPS31 |
| chr14 | 53173817 | 53173822 | promoter | PSMC6 |
| chr16 | 836281 | 836286 | coding | RPUSD1 |
| chr16 | 2510095 | 2510100 | promoter | C16orf59 |
| chr16 | 29802021 | 29802026 | promoter | KIF22 |
| chr16 | 29802021 | 29802026 | intron | BOLA2 |
| chr16 | 67440234 | 67440239 | coding | ZDHHC1 |
| chr16 | 67694194 | 67694199 | promoter | PARD6A |
| chr16 | 67694194 | 67694199 | coding | ACD |
| chr16 | 83841525 | 83841530 | fiveUTR | HSBP1 |
| chr16 | 83841525 | 83841530 | promoter | HSBP1 |
| chr17 | 30771480 | 30771485 | promoter | PSMD11 |
| chr17 | 37356486 | 37356491 | promoter | RPL19 |
| chr17 | 56769917 | 56769922 | promoter | RAD51C |
| chr17 | 56769917 | 56769922 | promoter | TEX14 |
| chr17 | 72346721 | 72346726 | intron | KIF19 |
| chr19 | 3435234 | 3435239 | intron | NFIC |
| chr19 | 4247060 | 4247065 | promoter | CCDC94 |
| chr19 | 4247072 | 4247077 | promoter | CCDC94 |
| chr19 | 10514238 | 10514243 | fiveUTR | CDC37 |
| chr19 | 10514238 | 10514243 | promoter | CDC37 |
| chr19 | 10514238 | 10514243 | promoter | MIR1181 |
| chr19 | 10514260 | 10514265 | fiveUTR | CDC37 |
| chr19 | 10514260 | 10514265 | promoter | CDC37 |
| chr19 | 10514260 | 10514265 | promoter | MIR1181 |
| chr19 | 13885240 | 13885245 | promoter | C19orf53 |
| chr19 | 15768976 | 15768981 | intron | CYP4F3 |
| chr19 | 17970682 | 17970687 | promoter | RPL18A |
| chr19 | 22836890 | 22836895 | intron | ZNF492 |
| chr19 | 38202422 | 38202427 | intron | ZNF607 |
| chr19 | 44901332 | 44901337 | intron | ZNF112 |
| chr19 | 44901332 | 44901337 | intron | ZNF285 |
| chr19 | 44901332 | 44901337 | promoter | ZNF285 |
| chr19 | 50169131 | 50169136 | coding | BCL2L12 |
| chr19 | 50169131 | 50169136 | promoter | IRF3 |
| chr19 | 51501258 | 51501263 | intron | KLK8 |
| chr19 | 51501258 | 51501263 | intron | KLK9 |
| chr19 | 54197615 | 54197620 | promoter | MIR526B |
| chr19 | 54197615 | 54197620 | promoter | MIR519B |
| chr19 | 54778707 | 54778712 | intron | LILRA6 |
| chr19 | 54778707 | 54778712 | intron | LILRB2 |
| chr19 | 56736012 | 56736017 | intron | ZSCAN5A |
| chr20 | 29623955 | 29623960 | intron | FRG1B |
| chr20 | 29623955 | 29623960 | promoter | FRG1B |
| chr20 | 34129792 | 34129797 | fiveUTR | ERGIC3 |
| chr20 | 34129792 | 34129797 | promoter | ERGIC3 |
| chr22 | 25016277 | 25016282 | intron | GGT1 |
| chr22 | 25016296 | 25016301 | coding | GGT1 |
| chr22 | 43011001 | 43011006 | promoter | RNU12 |
| chr22 | 43011001 | 43011006 | promoter | POLDIP3 |
| chr9 | 130700157 | 130700162 | fiveUTR | DPM2 |
| chr9 | 131038409 | 131038412 | promoter | SWI5 |
| chr9 | 131038409 | 131038412 | promoter | GOLGA2 |
| chrX | 34150221 | 34150226 | coding | FAM47A |
| chrX | 48830732 | 48830737 | intron | GRIPAP1 |
| chrX | 51075901 | 51075906 | coding | NUDT10 |
| chrX | 71351817 | 71351822 | intron | NHSL2 |
| chrX | 71351817 | 71351822 | promoter | NHSL2 |
| chrX | 71351817 | 71351822 | promoter | RGAG4 |
| chrX | 117043419 | 117043424 | coding | KLHL13 |
