# Supplementary material for: Clustered somatic mutations are frequent in transcription factor binding motifs within proximal promoter regions in melanoma and other cutaneous malignancies
Source: Oncotarget. 2016 Sep 7;7(41):66569–85. doi: 10.18632/oncotarget.11892 (PMC5341821; doi:10.18632/oncotarget.11892)
Supplement: Supplementary file 3 [file oncotarget-07-66569-s003.docx]

**Table S2. Primer sequences from the custom gene sequencing panel.**

| Name | Forward | Reverse |
| --- | --- | --- |
| SYF2_Promoter | CTTCCCACTTCCGGCAACAAG | CGCAAGACCCTACGCAATCC |
| TTC4_5'UTR | GCTGCCGGGAGGATTGAGG | GTCGTCTGAGGTGGGATCCTG |
| PGLYRP3_Intron | GGCCAGGAAGATGCCCTCTC | TCACCCTCCTCCTCGTCACC |
| RPS27_5'UTR | GTTCCAAACATGGTGGCACAAG | ATCTCCGCCGAAACCTGGAC |
| KIAA0907_Promoter | CCCGCCAACTATCCGACTACC | CTGCCTCAGAAACGCCAGATG |
| KIN_Promoter | TACTCAGCCAGCCGGAAACG | CCCCTCCCACCTTGCTCTC |
| CNTN5_Coding | CAGGCGGTGCTGGAAATACC | AATTTCCACTTTCAGTTTGACTTTTGG |
| EMG1_5'UTR | GAAGTCCCGCCCCTTTCTGG | GAACAGCGCGGCTTCTAATCAC |
| ALG10_5'UTR | GAAACAGCGACCCGATCTTTCC | GAGCCTGGAAACCCGAGCTG |
| PSMC6_Promoter | TGGAGGCCTCTTTCAGTGTATGC | GGCGAAACCCTGCCTGGAG |
| C16orf59_Promoter | CCGCAAGGAGGGAGAGAAAG | GCGGCGGCGTCTCCTTGC |
| HSBP1_5'UTR | GAGGGGCTATGCGAAAGAAGG | GCTTCTCGCGACGAGACTGG |
| PSMD11_Promoter | CCCGGCGGAGAGACTACAAC | GCCGCCATCTTACCGCTCTC |
| NFIC_Intron | GATTACTACACTTCGCCCAGCTC | GTGGCTGCCGGAAGACGTAG |
| CCDC94_Promoter | CTTGATAGGCGGGGATTGGAAC | TCACCTCGCAAGCACCTGAG |
| CYP4F3_Intron | TCCATGGGTCTATTTTCTTGTTTCTG | CAATCCTTATCAAGGGAGCAAAGAC |
| RPL18A_Promoter | GGGCAGAGAAGCCAATGGAC | GTGCCCGAGGCCTTCATGG |
| BCL2L12_Coding | TAACAGACCCAAAAGCCGATGG | AACCGGCCCAGCGTAGAAAG |
| MIR526B_Promoter | AAACATGCATACAGGGTCAATAAATG | TTCCCTCAAGAGGGTCACAGC |
| SLC30A6_Promoter | TCCTCCCACGCTTTCCTCAC | CGAAACCCTCACCCCAACAG |
| DPH3_Promoter | CTGGGCTCGGCATCATCAGG | CCGCCCCTAGTTCAGCCATC |
| KBTBD8_Promoter | GAGCCGGGGCAATCAAACTAC | CTTCTCTAGCTCCCGGGTTCC |
| FAM19A4_Intron | GGCGACCACCTCACAGGTC | GAAAATGTCATCATAGCATCATTTTGG |
| UMPS_5'UTR | GGGAAACCCACGGGTGCAG | ACGAGCGACCGCCATTGTCG |
| ZCCHC4_Promoter | GGGGCCGCTTAGGTGCAG | CCCGAAAGCCTCAGTACGAAAC |
| ERVMER34-1_Coding | GAAGGCCTTTGGTTTGGTTTTGG | GCACAAACCGGACCTGGAAC |
| TMPRSS11B_Intron | GGAATCAGGGTAAGCTAGTGGTC | GAATTGGTTTCAGTGCCCAGGAG |
| RPS3A_Promoter | CCACACACGATGGCGATGTC | AGTCGCGCGAGAACTTAGGC |
| DHX16_5'UTR | CGGCTGGAGCCTCAGCTTC | GGTATGGTTCCGCGAGGTTTG |
| YAE1D1_Promoter | CAAAATCACCGCAAGCGAAAGG | GACATCACCGAGGCAATTACGG |
| CHCHD2_5'UTR | GCTAGGCCTCCGGACGTG | CTTCAGGCCTGCGGAGACG |
| UBXN8_Promoter | CACGGCCGTCAGTATTTACCAC | CCCCACGTGAAGCCATGGTG |
| NDUFB9_5'UTR | GACCTCCCCAGCGGAAGC | CATTACGGCGCTGACCTTCC |
| BRAF_ex7_Coding | GAAAAATAGCCTCAATTCTTACCATCC | TCATGAAGACCTCACAGTAAAAATAGG |
| NRAS_ex3_Coding | GAGGAAGCCTTCGCCTGTCC | TGCCCCCTTACCCTCCACAC |
| INO80B_5'UTR | CGTTTCTCAGGGACGATTTTGG | TGAGGTCCTGCGAGGAAAGG |
| SWI5_Promoter | GCGATACTGGAGCGCAGAATG | GTGCGCAGCTTTTGTTGTGACG |
| C11orf49_5'UTR | CAGCTCCACTTTGCGACACG | CCTTTGACCGGGGTGGCTTC |
| RPL19_Promoter | TGGAGGCTATGAGTGAGTTTAGTG | CGCAGCAGCGAAAGGAAAGAG |
| NOC2L_Promoter | CAGCTGCCATGACACCAACC | TGCGGTCGGAGAACCATAGAG |
| CDC37_5'UTR | TGGTCCCACACGCTGTAGTCC | GCTGCCGCGGTTACTAGGG |
| c19orf53_Promoter | CCCGTGAGTTCTTGCGTTTTTC | CGGGTTTGTGCGCCTGAAAC |
| CCDC51_Promoter | CGACCAATCGTCTGCCACTC | ACCGGAAACGGAGCGTCTGC |
| POLDIP3_Promoter | CCGCTAGGGAGCGACGAAC | AATGTGCCGAACTTCCGTATGC |
| NOL10_Promoter | CCTTTCCCACCAGCGTGCTC | CCGGATTCAGCCACCTTCCC |
| C2orf49_Promoter | GCTTTTGCAAGCGGGACCAG | GAGACCCCAGTCGGCTACC |
| XIRP2_Coding | AACCCTGGTGTGTTTTTCTGTTTG | GCTTGGCTGGGGTTGTCATC |
| CDH9_Intron | GAAAATTGTTATTCAGTGCACCATGC | GGGAGCTCTCGTTGCGATTC |
| TCERG1_Promoter | CCCCCGAGTCACCAATCAAC | CCAACGAGAGAGGCGGAGAC |
| RBM22_5'UTR | AGAGAGGACCGCCACAATCC | ACCAGGACCCCAATTGTTAAGC |
| POM121L12_Coding | GGGCGAGACCTCTCCTGTG | TCTCCAGGGGCTCTCCTGAC |
| PREX2_Intron | GGAAGAATGCTTACACCCCGAAC | CACACATACCACTATGCCAAGAGC |
| BCCIP_Promoter | ACTCAGCGCCGAGCTTCTTC | CACGCCGCTTAGACCTGGAC |
| RPL27A_5'UTR | GCAATCCCGCGAGACCAGG | GGAATACAGCTTAGGGGTCTCC |
| FEN1_5'UTR | CCCCGCGAAGGCTAATCCG | CCCCTGCTCCCTCTACACC |
| GANAB_Promoter | TTGCTCCTTGACCCCAAACCTC | CCCCCTGCGATAATTTGGAGTG |
| SPRYD3_Promoter | CTCCGCACACAAGCTCTTCG | GCCTTCCGATTGGCTCCTGC |
| PPFIA2_Coding | CCTCTAGCCATGCGACCACAG | TCACAGCCATATTTTTCCATATACCTG |
| KSR2_Intron | GGTAGTCTGAAATCCAACCATGAATC | CAACAAAACCAGGCAGATTGCTC |
| EP400_Intron | TGGGCCCTTCTGCTTGAGTG | GGCTGCTGTGCCTTCTGCTG |
| MRPS31_5'UTR | GGAAACATCGCCGAGACACG | AGTTACCCGCCCCTGTAGCC |
| RPUSD1_Coding | ATCCAGGGAGGGCAGGAAGG | GTGGTGGGCGACCTGACC |
| KIF22_Promoter | GCTGGAAGGCGGGATAGTGG | CCATTCCACTCCCTCCTTGG |
| ZDHHC1_Coding | GATCCCAAAGCCGATCACAGC | GACGGCCCCTGAGAAGAGTG |
| ACD_Coding | GGCTACACCCAGCGGATGC | ACACAGGCCCCCGAGCAG |
| RAD51C_Promoter | AGACTGCGCAAAGCTGCAAGG | CGCAGGCTCACCTGCTAACC |
| ZNF607_Intron | CGAGGATGCTGGGTGATGTG | CTCGCCATGTGGAGGTTTGTG |
| KLK8_Intron | CCCTGAGAGAGCAGCCCTTTG | TGAGAAGTGATGTCCCCTGTGC |
| ERGIC3_5'UTR | GCCTGCGTGAGGAAAAAGAGG | TGGGGTAGGCATCGAACTGC |
| DPM2_5'UTR | GCACACGGTGCCAATCTCAC | AGCAGGCCTGGGGACTACG |
| GRIPAP1_Intron | CTTGCTGAGCCGCACAATTTCC | TCATCCCAGGCCAGACACAG |
| NHSL2_Intron | CTGCGGCAGGATCTGTCGAG | AGAGGCGCAGGCGAGCTG |
| INO80B_5'UTR | CGTTTCTCAGGGACGATTTTGG | TGAGGTCCTGCGAGGAAAGG |
| STAB1_Intron | AGTGGGCTCCCTCCCTCAG | GAGGCCATCTGGGTCCATGC |
| POLA2_Coding | CCCCCGGTCAGCTAGTGC | CGGGATGATGAGGACATCTGG |
| C17orf67_5'UTR | GAAAGTCTCCCAGCCCATGC | CCTGGAGCACTGGCTGAACC |
| KIF19_Intron | GCCTGCTGGAGCTGGAGAAC | AGCCACAGGTCGTGCTCCTC |
