# Supplementary material for: Clustered somatic mutations are frequent in transcription factor binding motifs within proximal promoter regions in melanoma and other cutaneous malignancies
Source: Oncotarget. 2016 Sep 7;7(41):66569–85. doi: 10.18632/oncotarget.11892 (PMC5341821; doi:10.18632/oncotarget.11892)
Supplement: Supplementary file 4 [file oncotarget-07-66569-s004.docx]

**Table S3. 182 cluster window regions from WGS.**

| CHROM | START | END | WIDTH | MUTATIONS | GENES |
| --- | --- | --- | --- | --- | --- |
| chr1 | 6845278 | 6845297 | 20 | 5 | CAMTA1 |
| chr1 | 25559063 | 25559078 | 16 | 7 | SYF2 |
| chr1 | 28585913 | 28585929 | 17 | 7 | SESN2 |
| chr1 | 38478322 | 38478338 | 17 | 6 | UTP11L |
| chr1 | 43824528 | 43824543 | 16 | 9 | CDC20 |
| chr1 | 54355506 | 54355520 | 15 | 4 | YIPF1 |
| chr1 | 63833263 | 63833277 | 15 | 4 | ALG6 |
| chr1 | 75197976 | 75197990 | 15 | 5 | TYW3 |
| chr1 | 100598553 | 100598567 | 15 | 8 | SASS6, TRMT13 |
| chr1 | 153963222 | 153963242 | 21 | 8 | RPS27 |
| chr1 | 226250305 | 226250319 | 15 | 4 | H3F3A, H3F3AP4 |
| chr1 | 231114784 | 231114798 | 15 | 4 | ARV1, TTC13 |
| chr1 | 231473732 | 231473746 | 15 | 5 | EXOC8, SPRTN |
| chr1 | 242011461 | 242011475 | 15 | 4 | EXO1 |
| chr2 | 26101488 | 26101503 | 16 | 7 | ASXL2 |
| chr2 | 32390897 | 32390911 | 15 | 4 | SLC30A6 |
| chr2 | 70056751 | 70056765 | 15 | 7 | GMCL1 |
| chr2 | 128615743 | 128615758 | 16 | 10 | POLR2D |
| chr2 | 234763235 | 234763249 | 15 | 5 | HJURP |
| chr3 | 12883297 | 12883311 | 15 | 4 | RPL32 |
| chr3 | 14693050 | 14693064 | 15 | 4 | CCDC174 |
| chr3 | 16306504 | 16306519 | 16 | 8 | DPH3, OXNAD1 |
| chr3 | 101280670 | 101280685 | 16 | 11 | TRMT10C |
| chr3 | 125314438 | 125314453 | 16 | 6 | OSBPL11 |
| chr4 | 1283556 | 1283570 | 15 | 4 | MAEA |
| chr4 | 2936615 | 2936642 | 28 | 6 | MFSD10, NOP14-AS1 |
| chr4 | 6576888 | 6576902 | 15 | 4 | MAN2B2 |
| chr4 | 25915723 | 25915737 | 15 | 4 | SMIM20 |
| chr4 | 48343328 | 48343342 | 15 | 4 | SLAIN2 |
| chr4 | 109541729 | 109541749 | 21 | 7 | RPL34-AS1, RPL34 |
| chr5 | 1295228 | 1295264 | 37 | 37 | TERT |
| chr5 | 32585650 | 32585664 | 15 | 4 | SUB1 |
| chr5 | 125936587 | 125936601 | 15 | 4 | PHAX |
| chr5 | 133702909 | 133702923 | 15 | 7 | CDKL3 |
| chr5 | 137800768 | 137800782 | 15 | 5 | EGR1 |
| chr5 | 137878970 | 137878984 | 15 | 5 | ETF1 |
| chr5 | 145562355 | 145562369 | 15 | 5 | LARS |
| chr5 | 171433908 | 171433922 | 15 | 4 | FBXW11 |
| chr6 | 3259136 | 3259150 | 15 | 4 | PSMG4 |
| chr6 | 26521056 | 26521070 | 15 | 4 | HCG11 |
| chr6 | 28048714 | 28048728 | 15 | 4 | ZNF165 |
| chr6 | 30585200 | 30585214 | 15 | 4 | MRPS18B, PPP1R10 |
| chr6 | 30640795 | 30640810 | 16 | 8 | DHX16 |
| chr6 | 34855824 | 34855838 | 15 | 4 | ANKS1A, TAF11 |
| chr6 | 44095263 | 44095289 | 27 | 5 | TMEM63B, MRPL14 |
| chr6 | 74230336 | 74230350 | 15 | 4 | EEF1A1 |
| chr6 | 149867285 | 149867299 | 15 | 4 | PPIL4 |
| chr6 | 170893742 | 170893756 | 15 | 4 | PDCD2 |
| chr7 | 1544063 | 1544077 | 15 | 8 | INTS1 |
| chr7 | 2671434 | 2671448 | 15 | 5 | TTYH3 |
| chr7 | 27779715 | 27779729 | 15 | 4 | TAX1BP1 |
| chr7 | 39605966 | 39605983 | 18 | 6 | YAE1D1 |
| chr7 | 56174180 | 56174194 | 15 | 4 | CHCHD2 |
| chr7 | 66461698 | 66461712 | 15 | 4 | SBDS, TYW1 |
| chr7 | 72298915 | 72298935 | 21 | 8 | SBDSP1, TYW1B |
| chr7 | 72298980 | 72298994 | 15 | 4 | SBDSP1, TYW1B |
| chr7 | 99756351 | 99756365 | 15 | 5 | C7orf43 |
| chr7 | 100210060 | 100210074 | 15 | 4 | MOSPD3 |
| chr7 | 102105380 | 102105394 | 15 | 6 | MIR5090, LRWD1, ALKBH4 |
| chr7 | 102213040 | 102213054 | 15 | 6 | POLR2J3 |
| chr7 | 111846605 | 111846619 | 15 | 4 | ZNF277, DOCK4 |
| chr7 | 120590716 | 120590730 | 15 | 4 | ING3 |
| chr7 | 138144802 | 138144816 | 15 | 4 | TRIM24 |
| chr7 | 140714739 | 140714766 | 28 | 8 | MRPS33 |
| chr8 | 30601668 | 30601682 | 15 | 8 | UBXN8 |
| chr8 | 37594117 | 37594131 | 15 | 4 | ERLIN2 |
| chr8 | 56987134 | 56987155 | 22 | 6 | SNORD54, RPS20 |
| chr8 | 67837824 | 67837840 | 17 | 6 | SNHG6 |
| chr8 | 75262590 | 75262605 | 16 | 7 | GDAP1 |
| chr8 | 124054557 | 124054572 | 16 | 9 | DERL1 |
| chr8 | 125551344 | 125551358 | 15 | 4 | NDUFB9, TATDN1 |
| chr9 | 3526039 | 3526053 | 15 | 4 | RFX3 |
| chr9 | 19103000 | 19103014 | 15 | 4 | HAUS6 |
| chr9 | 88555858 | 88555872 | 15 | 4 | NAA35 |
| chr9 | 91933357 | 91933371 | 15 | 5 | SECISBP2 |
| chr9 | 130954114 | 130954128 | 15 | 4 | CIZ1 |
| chr9 | 131038409 | 131038427 | 19 | 8 | GOLGA2, SWI5 |
| chr9 | 133454937 | 133454952 | 16 | 5 | LOC100272217, FUBP3 |
| chr10 | 7830002 | 7830016 | 15 | 5 | KIN, ATP5C1 |
| chr10 | 25013719 | 25013733 | 15 | 4 | ARHGAP21 |
| chr10 | 27443328 | 27443342 | 15 | 6 | YME1L1, MASTL |
| chr10 | 105156316 | 105156330 | 15 | 6 | PDCD11, USMG5 |
| chr10 | 126490350 | 126490364 | 15 | 4 | FAM175B |
| chr11 | 1330906 | 1330931 | 26 | 6 | TOLLIP-AS1, TOLLIP |
| chr11 | 30344546 | 30344560 | 15 | 5 | ARL14EP |
| chr11 | 45939434 | 45939449 | 16 | 5 | PEX16 |
| chr11 | 46958261 | 46958275 | 15 | 4 | C11orf49 |
| chr11 | 47448144 | 47448163 | 20 | 7 | PSMC3 |
| chr11 | 47870405 | 47870419 | 15 | 4 | NUP160 |
| chr11 | 61735191 | 61735206 | 16 | 11 | FTH1 |
| chr11 | 64018909 | 64018923 | 15 | 4 | PLCB3 |
| chr11 | 64863681 | 64863695 | 15 | 4 | VPS51 |
| chr11 | 82868069 | 82868089 | 21 | 6 | PCF11 |
| chr11 | 96123088 | 96123103 | 16 | 6 | CCDC82, JRKL |
| chr11 | 111797697 | 111797712 | 16 | 5 | DIXDC1 |
| chr11 | 111957541 | 111957558 | 18 | 6 | TIMM8B, SDHD |
| chr12 | 498776 | 498790 | 15 | 5 | KDM5A |
| chr12 | 34175266 | 34175281 | 16 | 5 | ALG10 |
| chr12 | 40499938 | 40499952 | 15 | 4 | SLC2A13 |
| chr12 | 49412648 | 49412663 | 16 | 8 | PRKAG1 |
| chr12 | 54582863 | 54582877 | 15 | 4 | SMUG1 |
| chr12 | 54582886 | 54582904 | 19 | 10 | SMUG1 |
| chr12 | 110434226 | 110434240 | 15 | 4 | GIT2 |
| chr13 | 37633863 | 37633877 | 15 | 5 | SUPT20H |
| chr13 | 41345346 | 41345360 | 15 | 6 | MRPS31 |
| chr13 | 41837721 | 41837747 | 27 | 5 | MTRF1 |
| chr13 | 52586460 | 52586474 | 15 | 4 | ALG11, ATP7B, UTP14C |
| chr13 | 60738141 | 60738155 | 15 | 4 | DIAPH3 |
| chr13 | 60738196 | 60738210 | 15 | 4 | DIAPH3 |
| chr13 | 115047496 | 115047510 | 15 | 4 | UPF3A |
| chr14 | 31028336 | 31028350 | 15 | 5 | G2E3 |
| chr14 | 55658346 | 55658365 | 20 | 5 | DLGAP5 |
| chr14 | 59655179 | 59655204 | 26 | 6 | DAAM1 |
| chr15 | 75628395 | 75628409 | 15 | 4 | COMMD4 |
| chr15 | 90931383 | 90931397 | 15 | 5 | IQGAP1 |
| chr15 | 100273816 | 100273831 | 16 | 6 | LYSMD4 |
| chr16 | 1470877 | 1470896 | 20 | 5 | C16orf91 |
| chr16 | 2510095 | 2510110 | 16 | 12 | C16orf59 |
| chr16 | 4666451 | 4666465 | 15 | 4 | UBALD1 |
| chr16 | 23568707 | 23568721 | 15 | 4 | EARS2, UBFD1 |
| chr16 | 25042922 | 25042937 | 16 | 6 | LOC554206 |
| chr16 | 67260923 | 67260937 | 15 | 4 | LRRC29, TMEM208 |
| chr16 | 67260974 | 67260997 | 24 | 7 | LRRC29, TMEM208 |
| chr16 | 68119138 | 68119152 | 15 | 4 | NFATC3 |
| chr16 | 83841525 | 83841540 | 16 | 7 | HSBP1 |
| chr16 | 88636744 | 88636758 | 15 | 4 | ZC3H18 |
| chr16 | 88923346 | 88923360 | 15 | 4 | GALNS, TRAPPC2L |
| chr17 | 1588263 | 1588277 | 15 | 4 | PRPF8 |
| chr17 | 6554934 | 6554948 | 15 | 5 | MED31 |
| chr17 | 7338572 | 7338593 | 22 | 6 | TMEM102 |
| chr17 | 30771480 | 30771494 | 15 | 4 | PSMD11 |
| chr17 | 38574320 | 38574334 | 15 | 4 | TOP2A |
| chr17 | 43394559 | 43394573 | 15 | 4 | MAP3K14 |
| chr17 | 45216135 | 45216149 | 15 | 4 | CDC27 |
| chr17 | 45973311 | 45973325 | 15 | 4 | SP2 |
| chr17 | 62207552 | 62207566 | 15 | 5 | ERN1 |
| chr17 | 79008738 | 79008752 | 15 | 5 | BAIAP2, BAIAP2-AS1 |
| chr17 | 79849512 | 79849527 | 16 | 9 | ALYREF, ANAPC11 |
| chr17 | 80231587 | 80231601 | 15 | 5 | CSNK1D |
| chr18 | 3262100 | 3262114 | 15 | 4 | MYL12B |
| chr19 | 1490861 | 1490875 | 15 | 4 | PCSK4, REEP6 |
| chr19 | 1490904 | 1490918 | 15 | 4 | PCSK4, REEP6 |
| chr19 | 1653018 | 1653037 | 20 | 5 | TCF3 |
| chr19 | 2151781 | 2151807 | 27 | 9 | AP3D1 |
| chr19 | 4639315 | 4639329 | 15 | 4 | TNFAIP8L1 |
| chr19 | 7459933 | 7459970 | 38 | 17 | ARHGEF18 |
| chr19 | 11373223 | 11373237 | 15 | 4 | DOCK6 |
| chr19 | 13049346 | 13049360 | 15 | 4 | CALR |
| chr19 | 13885240 | 13885254 | 15 | 4 | C19orf53 |
| chr19 | 17830241 | 17830256 | 16 | 6 | MAP1S |
| chr19 | 17970682 | 17970696 | 15 | 5 | RPL18A |
| chr19 | 19887356 | 19887370 | 15 | 4 | LINC00663 |
| chr19 | 47552109 | 47552123 | 15 | 4 | TMEM160 |
| chr19 | 48248737 | 48248754 | 18 | 6 | GLTSCR2 |
| chr19 | 48867537 | 48867556 | 20 | 5 | SYNGR4, TMEM143 |
| chr19 | 49990691 | 49990708 | 18 | 13 | RPL13A |
| chr19 | 49990743 | 49990757 | 15 | 4 | RPL13A |
| chr19 | 50269887 | 50269901 | 15 | 4 | AP2A1 |
| chr19 | 50269931 | 50269946 | 16 | 5 | AP2A1 |
| chr19 | 50270044 | 50270066 | 23 | 5 | AP2A1 |
| chr19 | 53606741 | 53606755 | 15 | 4 | ZNF160 |
| chr19 | 54641318 | 54641332 | 15 | 5 | CNOT3 |
| chr19 | 56135981 | 56135995 | 15 | 4 | ZNF784 |
| chr19 | 58978368 | 58978382 | 15 | 5 | ZNF324 |
| chr19 | 59010861 | 59010875 | 15 | 4 | SLC27A5 |
| chr20 | 2489815 | 2489829 | 15 | 4 | ZNF343 |
| chr20 | 24973457 | 24973471 | 15 | 4 | APMAP |
| chr20 | 30945967 | 30945981 | 15 | 4 | ASXL1 |
| chr20 | 32580916 | 32580941 | 26 | 8 | RALY |
| chr20 | 33464195 | 33464209 | 15 | 4 | ACSS2 |
| chr20 | 34129792 | 34129806 | 15 | 4 | ERGIC3 |
| chr20 | 45142211 | 45142225 | 15 | 4 | ZNF334 |
| chr20 | 57607411 | 57607425 | 15 | 4 | ATP5E |
| chr21 | 34100374 | 34100388 | 15 | 5 | PAXBP1-AS1, SYNJ1 |
| chr22 | 30988175 | 30988193 | 19 | 10 | PES1 |
| chr22 | 31556121 | 31556135 | 15 | 6 | MIR3928, RNF185 |
| chr22 | 34317157 | 34317171 | 15 | 4 | LARGE |
| chr22 | 35795975 | 35795989 | 15 | 4 | MCM5 |
| chr22 | 36877731 | 36877748 | 18 | 5 | TXN2 |
| chr22 | 43011002 | 43011016 | 15 | 4 | RNU12, POLDIP3 |
| chr22 | 45098012 | 45098032 | 21 | 6 | PRR5-ARHGAP8, PRR5 |
| chrX | 153059915 | 153059929 | 15 | 5 | IDH3G, SSR4 |
