# Supplementary material for: Clustered somatic mutations are frequent in transcription factor binding motifs within proximal promoter regions in melanoma and other cutaneous malignancies
Source: Oncotarget. 2016 Sep 7;7(41):66569–85. doi: 10.18632/oncotarget.11892 (PMC5341821; doi:10.18632/oncotarget.11892)
Supplement: Supplementary file 5 [file oncotarget-07-66569-s005.docx]

**Table S4. SNV data from the validation set of 170 clinical melanoma samples.**

| Sample | Chrom | Start | End | Ref allele | Alt allele | Read depth phred>20 | Alt allele frequency |
| --- | --- | --- | --- | --- | --- | --- | --- |
| Mel.1.Rep.1 | chr1 | 155904250 | 155904250 | C | T | 8190 | 6.79% |
| Mel.1.Rep.1 | chr3 | 16306504 | 16306504 | C | T | 6877 | 16.85% |
| Mel.1.Rep.2 | chr1 | 155904250 | 155904250 | C | T | 8929 | 15.58% |
| Mel.1.Rep.2 | chr3 | 16306505 | 16306505 | C | T | 6716 | 15.71% |
| Mel.1.Rep.2 | chr7 | 39605969 | 39605969 | G | A | 229 | 48.47% |
| Mel.1.Rep.2 | chr14 | 53173817 | 53173817 | G | A | 716 | 5.17% |
| Mel.1.Rep.2 | chr19 | 48248753 | 48248753 | C | T | 6036 | 11.20% |
| Mel.2.Rep.1 | chr5 | 150080667 | 150080667 | C | T | 3637 | 5.20% |
| Mel.2.Rep.2 | chr2 | 32390904 | 32390904 | C | T | 2858 | 3.67% |
| Mel.2.Rep.2 | chr5 | 150080667 | 150080667 | C | T | 3923 | 12.13% |
| Mel.2.Rep.2 | chr7 | 53103782 | 53103782 | G | A | 2455 | 5.34% |
| Mel.3.Rep.1 | chr1 | 153963239 | 153963239 | C | T | 5667 | 24.49% |
| Mel.3.Rep.1 | chr16 | 2510095 | 2510095 | G | A | 5564 | 99.51% |
| Mel.3.Rep.2 | chr2 | 32390904 | 32390904 | C | T | 3737 | 34.06% |
| Mel.3.Rep.2 | chr2 | 32390905 | 32390905 | C | T | 3756 | 4.10% |
| Mel.3.Rep.2 | chr3 | 68802177 | 68802177 | G | A | 4800 | 4.75% |
| Mel.3.Rep.2 | chr14 | 53173818 | 53173818 | G | A | 676 | 28.11% |
| Mel.4.Rep.1 | chr1 | 894682 | 894682 | C | T | 130 | 87.69% |
| Mel.4.Rep.1 | chr5 | 150080667 | 150080667 | C | T | 2558 | 42.40% |
| Mel.4.Rep.2 | chr1 | 153963222 | 153963222 | G | A | 6200 | 5.05% |
| Mel.4.Rep.2 | chr8 | 30601668 | 30601668 | G | A | 4828 | 4.81% |
| Mel.5.Rep.1 | chr1 | 153963222 | 153963222 | G | A | 5527 | 5.68% |
| Mel.5.Rep.1 | chr12 | 34175405 | 34175405 | C | T | 4479 | 5.20% |
| Mel.5.Rep.2 | chr3 | 48481605 | 48481605 | G | A | 4751 | 16.88% |
| Mel.6.Rep.1 | chr1 | 115256529 | 115256529 | T | C | 6428 | 49.08% |
| Mel.6.Rep.1 | chr7 | 39605969 | 39605969 | G | A | 5213 | 59.42% |
| Mel.6.Rep.1 | chr7 | 39605970 | 39605970 | G | A | 5207 | 59.59% |
| Mel.6.Rep.2 | chr1 | 894650 | 894650 | C | T | 300 | 70.67% |
| Mel.6.Rep.2 | chr1 | 115256529 | 115256529 | T | C | 2334 | 31.53% |
| Mel.6.Rep.2 | chr7 | 39605969 | 39605969 | G | A | 1457 | 27.87% |
| Mel.6.Rep.2 | chr7 | 39605970 | 39605970 | G | A | 1453 | 27.94% |
| Mel.6.Rep.2 | chr11 | 8704335 | 8704335 | C | T | 4342 | 3.04% |
| Mel.7.Rep.1 | chr2 | 10830114 | 10830114 | C | T | 11081 | 44.77% |
| Mel.7.Rep.1 | chr4 | 152020703 | 152020703 | G | A | 8151 | 10.32% |
| Mel.7.Rep.1 | chr7 | 140453136 | 140453136 | A | T | 2121 | 57.66% |
| Mel.7.Rep.1 | chr8 | 30601668 | 30601668 | G | A | 5650 | 17.10% |
| Mel.7.Rep.1 | chr8 | 30601669 | 30601669 | G | A | 5634 | 17.15% |
| Mel.7.Rep.1 | chr12 | 117923349 | 117923349 | G | A | 1665 | 24.55% |
| Mel.7.Rep.1 | chr17 | 30771480 | 30771480 | C | T | 3388 | 24.59% |
| Mel.7.Rep.1 | chr19 | 17970682 | 17970682 | C | T | 9151 | 46.60% |
| Mel.7.Rep.1 | chr19 | 48248748 | 48248748 | G | A | 5066 | 9.44% |
| Mel.7.Rep.2 | chr2 | 10830114 | 10830114 | C | T | 6011 | 35.09% |
| Mel.7.Rep.2 | chr7 | 140453136 | 140453136 | A | T | 573 | 5.41% |
| Mel.7.Rep.2 | chr8 | 30601668 | 30601668 | G | A | 5152 | 15.39% |
| Mel.7.Rep.2 | chr8 | 30601669 | 30601669 | G | A | 5140 | 15.23% |
| Mel.7.Rep.2 | chr12 | 117923349 | 117923349 | G | A | 3496 | 42.83% |
| Mel.7.Rep.2 | chr17 | 30771480 | 30771480 | C | T | 2633 | 99.35% |
| Mel.8.Rep.1 | chr1 | 25559063 | 25559063 | C | T | 6473 | 44.05% |
| Mel.8.Rep.1 | chr1 | 55181508 | 55181508 | G | A | 2513 | 68.68% |
| Mel.8.Rep.1 | chr1 | 55181509 | 55181509 | G | A | 2511 | 68.02% |
| Mel.8.Rep.1 | chr1 | 153963239 | 153963239 | C | T | 5976 | 99.18% |
| Mel.8.Rep.1 | chr1 | 155904250 | 155904250 | C | T | 2157 | 42.93% |
| Mel.8.Rep.1 | chr2 | 32390905 | 32390905 | C | T | 10330 | 56.36% |
| Mel.8.Rep.1 | chr7 | 140453145 | 140453145 | A | G | 1183 | 23.67% |
| Mel.8.Rep.1 | chr12 | 7080016 | 7080016 | G | A | 4058 | 39.77% |
| Mel.8.Rep.1 | chr14 | 53173818 | 53173818 | G | A | 1293 | 99.69% |
| Mel.8.Rep.1 | chr16 | 83841525 | 83841525 | C | T | 2595 | 7.24% |
| Mel.8.Rep.1 | chr16 | 83841526 | 83841526 | C | T | 2606 | 7.37% |
| Mel.8.Rep.1 | chr17 | 37356487 | 37356487 | C | T | 166 | 48.80% |
| Mel.8.Rep.2 | chr2 | 32390905 | 32390905 | C | T | 5327 | 24.52% |
| Mel.8.Rep.2 | chr4 | 152020703 | 152020703 | G | A | 6372 | 32.08% |
| Mel.8.Rep.2 | chr7 | 56174183 | 56174183 | G | A | 2174 | 8.56% |
| Mel.8.Rep.2 | chr9 | 131038414 | 131038414 | G | A | 1515 | 9.31% |
| Mel.8.Rep.2 | chr16 | 83841525 | 83841525 | C | T | 3606 | 70.44% |
| Mel.8.Rep.2 | chr16 | 83841526 | 83841526 | C | T | 3627 | 70.69% |
| Mel.8.Rep.2 | chr17 | 37356487 | 37356487 | C | T | 1154 | 12.31% |
| Mel.8.Rep.2 | chr19 | 10514260 | 10514260 | G | A | 554 | 96.57% |
| Mel.9.Rep.1 | chr4 | 152020700 | 152020700 | G | A | 9516 | 73.32% |
| Mel.9.Rep.1 | chr7 | 140453136 | 140453136 | A | T | 2432 | 8.51% |
| Mel.9.Rep.2 | chr1 | 153963227 | 153963227 | C | T | 2003 | 15.28% |
| Mel.9.Rep.2 | chr3 | 124449214 | 124449214 | T | C | 3133 | 3.19% |
| Mel.9.Rep.2 | chr4 | 152020700 | 152020700 | G | A | 6129 | 21.60% |
| Mel.9.Rep.2 | chr7 | 140453136 | 140453136 | A | T | 618 | 60.52% |
| Mel.10.Rep.1 | chr1 | 115256529 | 115256529 | T | C | 6950 | 17.57% |
| Mel.10.Rep.1 | chr1 | 153963207 | 153963207 | C | T | 8401 | 4.30% |
| Mel.10.Rep.1 | chr1 | 153963227 | 153963227 | C | T | 8407 | 6.98% |
| Mel.10.Rep.1 | chr13 | 41345355 | 41345355 | C | T | 10147 | 18.28% |
| Mel.10.Rep.1 | chr19 | 4247060 | 4247060 | G | A | 816 | 6.13% |
| Mel.10.Rep.1 | chr19 | 15768976 | 15768976 | C | T | 3146 | 3.18% |
| Mel.10.Rep.1 | chr19 | 17970682 | 17970682 | C | T | 13055 | 31.80% |
| Mel.10.Rep.1 | chr22 | 43011001 | 43011001 | G | A | 15623 | 15.81% |
| Mel.10.Rep.2 | chr1 | 115256529 | 115256529 | T | C | 810 | 11.85% |
| Mel.10.Rep.2 | chr3 | 16306504 | 16306504 | C | T | 3258 | 20.93% |
| Mel.10.Rep.2 | chr3 | 16306505 | 16306505 | C | T | 3268 | 4.90% |
| Mel.10.Rep.2 | chr8 | 30601669 | 30601669 | G | A | 3356 | 14.36% |
| Mel.10.Rep.2 | chr13 | 41345349 | 41345349 | C | T | 1583 | 5.69% |
| Mel.10.Rep.2 | chr16 | 2510095 | 2510095 | G | A | 1794 | 12.65% |
| Mel.10.Rep.2 | chr19 | 15768976 | 15768976 | C | T | 185 | 13.51% |
| Mel.10.Rep.2 | chr19 | 17970682 | 17970682 | C | T | 2054 | 36.08% |
| Mel.11.Rep.1 | chr1 | 155904250 | 155904250 | C | T | 6882 | 21.37% |
| Mel.11.Rep.1 | chr3 | 16306504 | 16306504 | C | T | 5862 | 24.76% |
| Mel.11.Rep.1 | chr3 | 16306505 | 16306505 | C | T | 5866 | 24.83% |
| Mel.11.Rep.1 | chr3 | 52542219 | 52542219 | G | A | 7640 | 3.09% |
| Mel.11.Rep.1 | chr13 | 41345355 | 41345355 | C | T | 9811 | 48.24% |
| Mel.11.Rep.1 | chr19 | 4247041 | 4247041 | G | A | 3673 | 51.59% |
| Mel.11.Rep.1 | chr19 | 10514228 | 10514228 | G | A | 1088 | 14.43% |
| Mel.11.Rep.1 | chr20 | 34129795 | 34129795 | G | A | 5925 | 20.30% |
| Mel.11.Rep.2 | chr1 | 155904250 | 155904250 | C | T | 6222 | 20.75% |
| Mel.11.Rep.2 | chr3 | 16306504 | 16306504 | C | T | 4485 | 11.62% |
| Mel.11.Rep.2 | chr3 | 16306505 | 16306505 | C | T | 4497 | 11.70% |
| Mel.11.Rep.2 | chr13 | 41345355 | 41345355 | C | T | 2128 | 50.52% |
| Mel.11.Rep.2 | chr19 | 4247041 | 4247041 | G | A | 1685 | 60.24% |
| Mel.11.Rep.2 | chr20 | 34129795 | 34129795 | G | A | 1675 | 29.77% |
| Mel.12.Rep.1 | chr7 | 140453136 | 140453136 | A | T | 3119 | 25.49% |
| Mel.12.Rep.1 | chr19 | 10514260 | 10514260 | G | A | 1364 | 14.30% |
| Mel.12.Rep.2 | chr7 | 140453136 | 140453136 | A | T | 408 | 26.23% |
| Mel.13.Rep.1 | chr1 | 55181529 | 55181529 | G | A | 4462 | 7.96% |
| Mel.13.Rep.1 | chr1 | 153963181 | 153963181 | C | T | 8285 | 10.72% |
| Mel.13.Rep.1 | chr2 | 32390905 | 32390905 | C | T | 13715 | 4.51% |
| Mel.13.Rep.1 | chr3 | 16306504 | 16306504 | C | T | 4960 | 9.27% |
| Mel.13.Rep.1 | chr3 | 16306505 | 16306505 | C | T | 4964 | 31.53% |
| Mel.13.Rep.1 | chr4 | 152020703 | 152020703 | G | A | 9068 | 9.70% |
| Mel.13.Rep.1 | chr7 | 140453136 | 140453136 | A | T | 1254 | 15% |
| Mel.13.Rep.1 | chr7 | 140453137 | 140453137 | C | T | 1256 | 14.97% |
| Mel.13.Rep.1 | chr8 | 30601669 | 30601669 | G | A | 3905 | 39.28% |
| Mel.13.Rep.1 | chr9 | 131038414 | 131038414 | G | A | 4389 | 9.73% |
| Mel.13.Rep.1 | chr19 | 3435234 | 3435234 | C | T | 4445 | 14.99% |
| Mel.13.Rep.1 | chr19 | 3435235 | 3435235 | C | T | 4434 | 15.11% |
| Mel.13.Rep.1 | chr22 | 43011002 | 43011002 | G | A | 16221 | 22.80% |
| Mel.13.Rep.2 | chr7 | 39605970 | 39605970 | G | A | 1450 | 21.38% |
| Mel.13.Rep.2 | chr7 | 53103759 | 53103759 | G | A | 617 | 3.57% |
| Mel.13.Rep.2 | chr8 | 30601669 | 30601669 | G | A | 2301 | 5.78% |
| Mel.13.Rep.2 | chr11 | 99932099 | 99932099 | C | T | 1451 | 4.07% |
| Mel.13.Rep.2 | chr16 | 836281 | 836281 | G | T | 901 | 5.77% |
| Mel.13.Rep.2 | chr19 | 3435234 | 3435234 | C | T | 440 | 15.91% |
| Mel.13.Rep.2 | chr19 | 3435235 | 3435235 | C | T | 438 | 15.75% |
| Mel.13.Rep.2 | chr19 | 4247060 | 4247060 | G | A | 234 | 15.81% |
| Mel.14.Rep.1 | chr7 | 140453136 | 140453136 | A | T | 4058 | 50.67% |
| Mel.14.Rep.1 | chr16 | 2510096 | 2510096 | G | A | 7953 | 31.71% |
| Mel.14.Rep.1 | chr17 | 30771480 | 30771480 | C | T | 6545 | 66.20% |
| Mel.14.Rep.1 | chr19 | 4247072 | 4247072 | C | T | 3307 | 3.21% |
| Mel.14.Rep.2 | chr7 | 140453136 | 140453136 | A | T | 2563 | 51.23% |
| Mel.14.Rep.2 | chr16 | 2510096 | 2510096 | G | A | 6237 | 21.28% |
| Mel.14.Rep.2 | chr17 | 30771480 | 30771480 | C | T | 4468 | 9.33% |
| Mel.15.Rep.1 | chr1 | 115256530 | 115256530 | G | T | 6857 | 57.90% |
| Mel.15.Rep.1 | chr7 | 56174183 | 56174183 | G | A | 5825 | 23.99% |
| Mel.15.Rep.1 | chr9 | 130700157 | 130700157 | C | T | 2437 | 26.30% |
| Mel.15.Rep.2 | chr1 | 115256530 | 115256530 | G | T | 6248 | 62.86% |
| Mel.15.Rep.2 | chr7 | 56174183 | 56174183 | G | A | 4876 | 37.35% |
| Mel.15.Rep.2 | chr9 | 130700157 | 130700157 | C | T | 2551 | 47.35% |
| Mel.16.Rep.1 | chr1 | 55181528 | 55181528 | G | A | 5184 | 5.88% |
| Mel.16.Rep.1 | chr7 | 56174204 | 56174204 | C | T | 6392 | 16.41% |
| Mel.16.Rep.1 | chr7 | 140453136 | 140453136 | A | T | 2101 | 48.79% |
| Mel.16.Rep.1 | chr11 | 8704338 | 8704338 | C | T | 15311 | 31.28% |
| Mel.16.Rep.1 | chr12 | 81693129 | 81693129 | C | T | 11343 | 5.14% |
| Mel.16.Rep.1 | chr16 | 2510095 | 2510095 | G | T | 8369 | 4.34% |
| Mel.16.Rep.1 | chr19 | 17970682 | 17970682 | C | T | 13053 | 60.18% |
| Mel.16.Rep.1 | chr19 | 50169132 | 50169132 | C | T | 6010 | 8.97% |
| Mel.16.Rep.2 | chr7 | 140453136 | 140453136 | A | T | 2280 | 88.90% |
| Mel.16.Rep.2 | chr19 | 17970682 | 17970682 | C | T | 2716 | 50.87% |
| Mel.17.Rep.1 | chr1 | 55181529 | 55181529 | G | T | 4942 | 7.75% |
| Mel.17.Rep.1 | chr3 | 48481581 | 48481581 | C | T | 7448 | 9.22% |
| Mel.17.Rep.1 | chr19 | 10514253 | 10514253 | G | A | 587 | 21.12% |
| Mel.17.Rep.2 | chr11 | 99932099 | 99932099 | C | T | 5931 | 4.69% |
| Mel.18.Rep.1 | chr1 | 153963227 | 153963227 | C | T | 8895 | 12.64% |
| Mel.18.Rep.1 | chr7 | 140453136 | 140453136 | A | T | 2702 | 3.52% |
| Mel.18.Rep.1 | chr14 | 53173818 | 53173818 | G | A | 2872 | 9.30% |
| Mel.18.Rep.2 | chr1 | 153963227 | 153963227 | C | T | 1892 | 10.73% |
| Mel.18.Rep.2 | chr7 | 140453136 | 140453136 | A | T | 857 | 19.25% |
| Mel.18.Rep.2 | chr8 | 30601668 | 30601668 | G | A | 4554 | 4.50% |
| Mel.19.Rep.1 | chr7 | 140453136 | 140453136 | A | T | 1990 | 13.62% |
| Mel.19.Rep.2 | chr7 | 140453136 | 140453136 | A | T | 2397 | 37.51% |
| Mel.20.Rep.1 | chr7 | 56174183 | 56174183 | G | A | 5970 | 3.25% |
| Mel.20.Rep.1 | chr7 | 140453136 | 140453136 | A | T | 275 | 73.09% |
| Mel.20.Rep.1 | chr9 | 131038409 | 131038409 | G | A | 1242 | 7.49% |
| Mel.20.Rep.2 | chr7 | 140453136 | 140453136 | A | T | 214 | 31.78% |
| Mel.20.Rep.2 | chr16 | 67694198 | 67694198 | G | A | 1230 | 3.58% |
| Mel.21.Rep.1 | chr2 | 168098327 | 168098327 | G | A | 10759 | 3.14% |
| Mel.21.Rep.1 | chr7 | 140453136 | 140453136 | A | T | 3355 | 11.42% |
| Mel.21.Rep.1 | chr14 | 53173808 | 53173808 | C | T | 2456 | 3.34% |
| Mel.21.Rep.1 | chr17 | 56769917 | 56769917 | C | T | 10123 | 13.62% |
| Mel.21.Rep.1 | chr19 | 50169132 | 50169132 | C | T | 6302 | 7.84% |
| Mel.21.Rep.2 | chr7 | 140453136 | 140453136 | A | T | 2419 | 5.83% |
| Mel.21.Rep.2 | chr17 | 56769917 | 56769917 | C | T | 1784 | 9.53% |
| Mel.21.Rep.2 | chr19 | 50169132 | 50169132 | C | T | 247 | 14.17% |
| Mel.22.Rep.1 | chr1 | 25559063 | 25559063 | C | T | 7538 | 65.48% |
| Mel.22.Rep.1 | chr19 | 17970682 | 17970682 | C | T | 5996 | 73.34% |
| Mel.22.Rep.2 | chr2 | 32390904 | 32390904 | C | T | 312 | 44.87% |
| Mel.22.Rep.2 | chr3 | 68802177 | 68802177 | G | T | 1342 | 3.65% |
| Mel.22.Rep.2 | chr9 | 130700157 | 130700157 | C | T | 497 | 84.10% |
| Mel.22.Rep.2 | chr16 | 2510096 | 2510096 | G | A | 413 | 40.44% |
| Mel.23.Rep.1 | chr8 | 68931946 | 68931946 | C | T | 9438 | 9.82% |
| Mel.23.Rep.1 | chr17 | 54872420 | 54872420 | T | A | 7015 | 10.65% |
| Mel.24.Rep.1 | chr2 | 74682176 | 74682176 | C | T | 4598 | 25.51% |
| Mel.24.Rep.1 | chr7 | 140453136 | 140453136 | A | T | 2796 | 33.44% |
| Mel.24.Rep.1 | chr19 | 13885240 | 13885240 | C | T | 5475 | 9.04% |
| Mel.24.Rep.2 | chr2 | 74682176 | 74682176 | C | T | 3176 | 25.88% |
| Mel.24.Rep.2 | chr3 | 67048644 | 67048644 | C | T | 3469 | 3.23% |
| Mel.24.Rep.2 | chr7 | 140453136 | 140453136 | A | T | 2111 | 14.92% |
| Mel.24.Rep.2 | chr19 | 51501258 | 51501258 | G | A | 4768 | 3.52% |
| Mel.25.Rep.1 | chr1 | 115256529 | 115256529 | T | A | 6814 | 9.97% |
| Mel.25.Rep.1 | chr1 | 153963227 | 153963227 | C | T | 7824 | 18.42% |
| Mel.25.Rep.1 | chr5 | 150080667 | 150080667 | C | T | 13817 | 37.68% |
| Mel.25.Rep.1 | chr14 | 53173817 | 53173817 | G | A | 2112 | 34.80% |
| Mel.25.Rep.1 | chr14 | 53173818 | 53173818 | G | A | 2114 | 34.63% |
| Mel.25.Rep.1 | chr19 | 3435235 | 3435235 | C | T | 4676 | 45.34% |
| Mel.25.Rep.1 | chr19 | 4247045 | 4247045 | G | A | 3439 | 83.16% |
| Mel.25.Rep.2 | chr1 | 115256529 | 115256529 | T | A | 1647 | 39.71% |
| Mel.25.Rep.2 | chr1 | 153963227 | 153963227 | C | T | 1756 | 37.81% |
| Mel.25.Rep.2 | chr2 | 74682176 | 74682176 | C | T | 5326 | 5.14% |
| Mel.25.Rep.2 | chr5 | 150080667 | 150080667 | C | T | 1483 | 32.34% |
| Mel.25.Rep.2 | chr19 | 3435235 | 3435235 | C | T | 649 | 44.07% |
| Mel.25.Rep.2 | chr19 | 4247045 | 4247045 | G | A | 2190 | 31.78% |
| Mel.26.Rep.1 | chr1 | 55181508 | 55181508 | G | A | 4520 | 8.96% |
| Mel.26.Rep.1 | chr7 | 140453136 | 140453136 | A | T | 3255 | 27.50% |
| Mel.26.Rep.1 | chr14 | 53173818 | 53173818 | G | A | 2796 | 13.63% |
| Mel.26.Rep.2 | chr1 | 55181508 | 55181508 | G | A | 4602 | 6.56% |
| Mel.26.Rep.2 | chr7 | 140453136 | 140453136 | A | T | 3809 | 16.46% |
| Mel.26.Rep.2 | chr14 | 53173817 | 53173817 | G | A | 2976 | 10.79% |
| Mel.27.Rep.1 | chr1 | 894683 | 894683 | C | T | 230 | 42.61% |
| Mel.27.Rep.1 | chr1 | 894688 | 894688 | C | T | 230 | 94.78% |
| Mel.27.Rep.2 | chr10 | 127512084 | 127512084 | G | A | 1883 | 8.18% |
| Mel.27.Rep.2 | chr17 | 30771480 | 30771480 | C | T | 3085 | 5.61% |
| Mel.28.Rep.1 | chr2 | 32390905 | 32390905 | C | T | 14790 | 15.56% |
| Mel.28.Rep.1 | chr9 | 131038409 | 131038409 | G | A | 4789 | 7.50% |
| Mel.28.Rep.1 | chr14 | 53173818 | 53173818 | G | A | 2253 | 25.52% |
| Mel.28.Rep.1 | chr17 | 30771480 | 30771480 | C | T | 3902 | 13.69% |
| Mel.29.Rep.1 | chr1 | 153963227 | 153963227 | C | T | 5333 | 5.03% |
| Mel.29.Rep.1 | chr1 | 153963239 | 153963239 | C | T | 5324 | 10.28% |
| Mel.29.Rep.1 | chr1 | 155904250 | 155904250 | C | T | 6011 | 21.05% |
| Mel.29.Rep.1 | chr2 | 32390904 | 32390904 | C | T | 3229 | 5.05% |
| Mel.29.Rep.1 | chr2 | 32390905 | 32390905 | C | T | 3210 | 46.35% |
| Mel.29.Rep.1 | chr2 | 74682175 | 74682175 | C | T | 5228 | 16.12% |
| Mel.29.Rep.1 | chr2 | 74682176 | 74682176 | C | T | 5207 | 38.44% |
| Mel.29.Rep.1 | chr3 | 16306504 | 16306504 | C | T | 4118 | 4.78% |
| Mel.29.Rep.1 | chr3 | 48481602 | 48481602 | C | T | 4065 | 46.40% |
| Mel.29.Rep.1 | chr3 | 124449234 | 124449234 | G | A | 4428 | 14.10% |
| Mel.29.Rep.1 | chr4 | 152020703 | 152020703 | G | A | 4910 | 17.59% |
| Mel.29.Rep.1 | chr6 | 30640795 | 30640795 | G | A | 4992 | 52.84% |
| Mel.29.Rep.1 | chr8 | 30601669 | 30601669 | G | A | 4769 | 28.85% |
| Mel.29.Rep.1 | chr8 | 125551345 | 125551345 | C | T | 2939 | 52.40% |
| Mel.29.Rep.1 | chr12 | 53473215 | 53473215 | C | T | 2715 | 29.06% |
| Mel.29.Rep.1 | chr12 | 117923348 | 117923348 | G | A | 3281 | 25.43% |
| Mel.29.Rep.1 | chr16 | 29802021 | 29802021 | G | A | 2797 | 17.29% |
| Mel.29.Rep.1 | chr16 | 29802047 | 29802047 | C | T | 2820 | 3.01% |
| Mel.29.Rep.1 | chr16 | 67694198 | 67694198 | G | A | 356 | 50.56% |
| Mel.29.Rep.1 | chr17 | 30771480 | 30771480 | C | T | 2971 | 71.85% |
| Mel.29.Rep.1 | chr17 | 30771481 | 30771481 | T | G | 3060 | 72.90% |
| Mel.29.Rep.1 | chr17 | 37356494 | 37356494 | G | A | 1499 | 24.48% |
| Mel.29.Rep.1 | chr19 | 3435235 | 3435235 | C | T | 3710 | 6.28% |
| Mel.29.Rep.1 | chr19 | 4247045 | 4247045 | G | A | 3062 | 10.09% |
| Mel.29.Rep.1 | chr22 | 43011002 | 43011002 | G | A | 5761 | 9.30% |
| Mel.29.Rep.2 | chr1 | 155904250 | 155904250 | C | T | 6826 | 35.46% |
| Mel.29.Rep.2 | chr2 | 32390905 | 32390905 | C | T | 4490 | 31.42% |
| Mel.29.Rep.2 | chr2 | 74682122 | 74682122 | G | A | 6584 | 10.48% |
| Mel.29.Rep.2 | chr2 | 74682175 | 74682175 | C | T | 6590 | 28.51% |
| Mel.29.Rep.2 | chr2 | 74682176 | 74682176 | C | T | 6595 | 5.95% |
| Mel.29.Rep.2 | chr3 | 16306504 | 16306504 | C | T | 5634 | 24.33% |
| Mel.29.Rep.2 | chr3 | 16306505 | 16306505 | C | T | 5646 | 4.27% |
| Mel.29.Rep.2 | chr3 | 48481602 | 48481602 | C | T | 3817 | 16.90% |
| Mel.29.Rep.2 | chr3 | 124449246 | 124449246 | C | T | 5176 | 17.33% |
| Mel.29.Rep.2 | chr4 | 152020703 | 152020703 | G | A | 5816 | 20.36% |
| Mel.29.Rep.2 | chr6 | 30640795 | 30640795 | G | A | 4575 | 58.71% |
| Mel.29.Rep.2 | chr8 | 125551345 | 125551345 | C | T | 3445 | 30.69% |
| Mel.29.Rep.2 | chr11 | 61560107 | 61560107 | G | A | 4178 | 13.14% |
| Mel.29.Rep.2 | chr16 | 67694198 | 67694198 | G | A | 1927 | 43.85% |
| Mel.29.Rep.2 | chr17 | 30771480 | 30771480 | C | T | 5356 | 42.55% |
| Mel.29.Rep.2 | chr17 | 30771481 | 30771481 | T | G | 5449 | 44.04% |
| Mel.29.Rep.2 | chr17 | 37356494 | 37356494 | G | A | 1451 | 16.68% |
| Mel.29.Rep.2 | chr19 | 3435235 | 3435235 | C | T | 5069 | 26.28% |
| Mel.29.Rep.2 | chr19 | 10514238 | 10514238 | C | T | 286 | 77.89% |
| Mel.29.Rep.2 | chr19 | 10514239 | 10514239 | C | T | 286 | 77.27% |
| Mel.29.Rep.2 | chr19 | 38202422 | 38202422 | G | A | 9411 | 9.84% |
| Mel.29.Rep.2 | chr20 | 34129792 | 34129792 | G | A | 3810 | 10.68% |
| Mel.30.Rep.1 | chr1 | 115256530 | 115256530 | G | T | 6952 | 37.85% |
| Mel.30.Rep.1 | chr12 | 53473217 | 53473217 | C | T | 5206 | 10.64% |
| Mel.30.Rep.1 | chr16 | 67694198 | 67694198 | G | A | 3291 | 7.93% |
| Mel.30.Rep.2 | chr1 | 115256530 | 115256530 | G | T | 6759 | 51.88% |
| Mel.30.Rep.2 | chr2 | 74682122 | 74682122 | G | A | 4968 | 12% |
| Mel.30.Rep.2 | chr9 | 131038413 | 131038413 | G | A | 5481 | 3.74% |
| Mel.31.Rep.1 | chr1 | 25559063 | 25559063 | C | T | 8103 | 36.12% |
| Mel.31.Rep.1 | chr1 | 115256529 | 115256529 | T | C | 6671 | 3.16% |
| Mel.31.Rep.1 | chr1 | 153963181 | 153963181 | C | T | 7779 | 19.50% |
| Mel.31.Rep.1 | chr1 | 155904250 | 155904250 | C | T | 5760 | 21.75% |
| Mel.31.Rep.1 | chr1 | 231114784 | 231114784 | C | T | 896 | 8.15% |
| Mel.31.Rep.1 | chr2 | 10830118 | 10830118 | G | A | 9718 | 19.40% |
| Mel.31.Rep.1 | chr2 | 32390904 | 32390904 | C | T | 13202 | 56.66% |
| Mel.31.Rep.1 | chr2 | 32390905 | 32390905 | C | T | 13212 | 56.97% |
| Mel.31.Rep.1 | chr3 | 16306504 | 16306504 | C | T | 5570 | 44.06% |
| Mel.31.Rep.1 | chr3 | 16306505 | 16306505 | C | T | 5577 | 50.80% |
| Mel.31.Rep.1 | chr3 | 67048644 | 67048644 | C | T | 7695 | 40.25% |
| Mel.31.Rep.1 | chr4 | 25314329 | 25314329 | C | T | 440 | 88.64% |
| Mel.31.Rep.1 | chr7 | 39605970 | 39605970 | G | A | 1954 | 61.09% |
| Mel.31.Rep.1 | chr8 | 125551345 | 125551345 | C | T | 3370 | 41.13% |
| Mel.31.Rep.1 | chr9 | 131038414 | 131038414 | G | A | 4549 | 16.07% |
| Mel.31.Rep.1 | chr10 | 7830002 | 7830002 | G | A | 3732 | 29.96% |
| Mel.31.Rep.1 | chr11 | 46958261 | 46958261 | C | T | 6749 | 44.21% |
| Mel.31.Rep.1 | chr11 | 46958262 | 46958262 | C | T | 6745 | 44.27% |
| Mel.31.Rep.1 | chr14 | 53173818 | 53173818 | G | A | 2362 | 14.61% |
| Mel.31.Rep.1 | chr16 | 2510095 | 2510095 | G | A | 4555 | 38.92% |
| Mel.31.Rep.1 | chr16 | 2510096 | 2510096 | G | A | 4543 | 39.07% |
| Mel.31.Rep.1 | chr16 | 83841526 | 83841526 | C | T | 3795 | 85.74% |
| Mel.31.Rep.1 | chr19 | 4247045 | 4247045 | G | A | 3568 | 9.45% |
| Mel.31.Rep.1 | chr19 | 10514238 | 10514238 | C | T | 213 | 13.62% |
| Mel.31.Rep.1 | chr19 | 10514239 | 10514239 | C | T | 214 | 13.08% |
| Mel.31.Rep.1 | chr20 | 34129795 | 34129795 | G | A | 4548 | 48.12% |
| Mel.31.Rep.2 | chr1 | 25559063 | 25559063 | C | T | 1583 | 40.81% |
| Mel.31.Rep.2 | chr1 | 153963181 | 153963181 | C | T | 2088 | 22.84% |
| Mel.31.Rep.2 | chr1 | 155904250 | 155904250 | C | T | 1973 | 14.85% |
| Mel.31.Rep.2 | chr1 | 231114784 | 231114784 | C | T | 1490 | 42.01% |
| Mel.31.Rep.2 | chr2 | 10830118 | 10830118 | G | A | 4234 | 28.50% |
| Mel.31.Rep.2 | chr2 | 32390904 | 32390904 | C | T | 4357 | 70.74% |
| Mel.31.Rep.2 | chr2 | 32390905 | 32390905 | C | T | 4344 | 70.88% |
| Mel.31.Rep.2 | chr3 | 16306504 | 16306504 | C | T | 4508 | 24.84% |
| Mel.31.Rep.2 | chr3 | 16306505 | 16306505 | C | T | 4508 | 37.02% |
| Mel.31.Rep.2 | chr3 | 67048644 | 67048644 | C | T | 1988 | 26.36% |
| Mel.31.Rep.2 | chr4 | 25314329 | 25314329 | C | T | 214 | 64.95% |
| Mel.31.Rep.2 | chr7 | 140453136 | 140453136 | A | G | 630 | 3.33% |
| Mel.31.Rep.2 | chr8 | 125551345 | 125551345 | C | T | 2687 | 31.50% |
| Mel.31.Rep.2 | chr11 | 46958261 | 46958261 | C | T | 2999 | 37.89% |
| Mel.31.Rep.2 | chr11 | 46958262 | 46958262 | C | T | 3008 | 37.99% |
| Mel.31.Rep.2 | chr16 | 2510095 | 2510095 | G | A | 3486 | 74.07% |
| Mel.31.Rep.2 | chr16 | 2510096 | 2510096 | G | A | 3467 | 67.61% |
| Mel.31.Rep.2 | chr16 | 83841526 | 83841526 | C | T | 3006 | 24.14% |
| Mel.31.Rep.2 | chr19 | 3435234 | 3435234 | C | T | 2052 | 9.41% |
| Mel.31.Rep.2 | chr19 | 3435235 | 3435235 | C | T | 2052 | 9.60% |
| Mel.31.Rep.2 | chr20 | 34129795 | 34129795 | G | A | 2840 | 7.57% |
| Mel.32.Rep.1 | chr1 | 55181508 | 55181508 | G | A | 3147 | 29.87% |
| Mel.32.Rep.1 | chr1 | 55181509 | 55181509 | G | A | 3140 | 57.68% |
| Mel.32.Rep.1 | chr1 | 155904250 | 155904250 | C | T | 851 | 92.12% |
| Mel.32.Rep.1 | chr2 | 32390904 | 32390904 | C | T | 6760 | 66.45% |
| Mel.32.Rep.1 | chr2 | 32390905 | 32390905 | C | T | 6755 | 66.72% |
| Mel.32.Rep.1 | chr7 | 39605969 | 39605969 | G | A | 144 | 13.89% |
| Mel.32.Rep.1 | chr7 | 56174183 | 56174183 | G | A | 3074 | 9.63% |
| Mel.32.Rep.1 | chr9 | 130700157 | 130700157 | C | T | 1257 | 4.85% |
| Mel.32.Rep.1 | chr16 | 2510095 | 2510095 | G | A | 144 | 93.75% |
| Mel.32.Rep.1 | chr19 | 3435234 | 3435234 | C | T | 3008 | 9.91% |
| Mel.32.Rep.2 | chr1 | 55181508 | 55181508 | G | A | 524 | 95.23% |
| Mel.32.Rep.2 | chr1 | 55181509 | 55181509 | G | A | 519 | 95.18% |
| Mel.32.Rep.2 | chr1 | 155904250 | 155904250 | C | T | 2052 | 59.04% |
| Mel.32.Rep.2 | chr6 | 30640795 | 30640795 | G | A | 5669 | 80.75% |
| Mel.33.Rep.1 | chr1 | 55181528 | 55181528 | G | A | 5359 | 3.49% |
| Mel.33.Rep.1 | chr6 | 30640795 | 30640795 | G | A | 4814 | 3.18% |
| Mel.33.Rep.1 | chr6 | 30640796 | 30640796 | G | A | 4794 | 3.19% |
| Mel.33.Rep.1 | chr7 | 140453136 | 140453136 | A | T | 4222 | 23.12% |
| Mel.33.Rep.1 | chr19 | 4247045 | 4247045 | G | A | 3269 | 21.99% |
| Mel.33.Rep.2 | chr1 | 231114782 | 231114782 | G | A | 1260 | 7.14% |
| Mel.33.Rep.2 | chr7 | 140453136 | 140453136 | A | T | 6260 | 9.94% |
| Mel.33.Rep.2 | chr17 | 56769917 | 56769917 | C | T | 3326 | 41.49% |
| Mel.33.Rep.2 | chr19 | 4247045 | 4247045 | G | A | 4362 | 15.15% |
| Mel.34.Rep.1 | chr3 | 16306505 | 16306505 | C | T | 4849 | 99.15% |
| Mel.34.Rep.1 | chr19 | 51501258 | 51501259 | GG | G | 8157 | 3.77% |
| Mel.34.Rep.2 | chr11 | 46958261 | 46958261 | C | T | 3819 | 21.55% |
| Mel.35.Rep.1 | chr19 | 3435234 | 3435234 | C | T | 4110 | 35.50% |
| Mel.35.Rep.2 | chr7 | 53103782 | 53103782 | G | A | 1558 | 20.60% |
| Mel.35.Rep.2 | chr8 | 30601669 | 30601669 | G | A | 1899 | 98.79% |
| Mel.35.Rep.2 | chr19 | 50169132 | 50169132 | C | T | 1963 | 4.64% |
| Mel.36.Rep.1 | chr1 | 55181528 | 55181528 | G | A | 6696 | 3.99% |
| Mel.36.Rep.1 | chr1 | 115256530 | 115256530 | G | T | 7393 | 66.55% |
| Mel.36.Rep.1 | chr8 | 125551345 | 125551345 | C | A | 2900 | 28.83% |
| Mel.36.Rep.2 | chr1 | 115256530 | 115256530 | G | T | 6169 | 66.90% |
| Mel.36.Rep.2 | chr7 | 53103783 | 53103783 | G | A | 1677 | 23.43% |
| Mel.36.Rep.2 | chr8 | 125551345 | 125551345 | C | A | 1119 | 75.69% |
| Mel.36.Rep.2 | chr22 | 43011001 | 43011001 | G | A | 5453 | 15.24% |
| Mel.37.Rep.1 | chr3 | 16306504 | 16306504 | C | T | 7842 | 9.68% |
| Mel.37.Rep.2 | chr2 | 10830117 | 10830117 | G | A | 5966 | 3% |
| Mel.37.Rep.2 | chr2 | 10830118 | 10830118 | G | A | 5897 | 25.29% |
| Mel.37.Rep.2 | chr3 | 16306504 | 16306504 | C | T | 7000 | 18.45% |
| Mel.37.Rep.2 | chr3 | 16306505 | 16306505 | C | T | 7019 | 18.46% |
| Mel.37.Rep.2 | chr12 | 81693129 | 81693129 | C | A | 5883 | 4.61% |
| Mel.38.Rep.2 | chr11 | 46958261 | 46958261 | C | T | 7546 | 11.49% |
| Mel.39.Rep.1 | chr3 | 48481602 | 48481602 | C | T | 6402 | 9.53% |
| Mel.39.Rep.1 | chr5 | 145826780 | 145826780 | C | T | 1839 | 8.75% |
| Mel.39.Rep.2 | chr1 | 115256530 | 115256530 | G | T | 8601 | 3.43% |
| Mel.39.Rep.2 | chr12 | 7080017 | 7080017 | G | A | 7155 | 4.54% |
| Mel.40.Rep.1 | chr7 | 39605969 | 39605969 | G | A | 7296 | 3.71% |
| Mel.40.Rep.1 | chr7 | 39605970 | 39605970 | G | A | 7230 | 3.87% |
| Mel.41.Rep.1 | chr1 | 25559064 | 25559064 | C | T | 8308 | 16.43% |
| Mel.41.Rep.1 | chr1 | 153963239 | 153963239 | C | T | 8415 | 21.15% |
| Mel.41.Rep.1 | chr4 | 69093838 | 69093838 | C | T | 3666 | 6.41% |
| Mel.41.Rep.1 | chr8 | 125551345 | 125551345 | C | T | 4276 | 7.02% |
| Mel.41.Rep.1 | chr12 | 7080017 | 7080017 | G | A | 6678 | 7.23% |
| Mel.41.Rep.1 | chr12 | 53473218 | 53473219 | CC | C | 2557 | 11.48% |
| Mel.41.Rep.1 | chr19 | 3435234 | 3435234 | C | T | 6388 | 42.03% |
| Mel.41.Rep.1 | chr19 | 13885240 | 13885240 | C | T | 1626 | 52.28% |
| Mel.41.Rep.1 | chr20 | 34129795 | 34129795 | G | A | 3548 | 8.74% |
| Mel.41.Rep.2 | chr4 | 69093838 | 69093838 | C | T | 5675 | 17.29% |
| Mel.41.Rep.2 | chr4 | 152020703 | 152020703 | G | A | 8115 | 35.19% |
| Mel.41.Rep.2 | chr4 | 152020712 | 152020712 | G | A | 8082 | 18.04% |
| Mel.41.Rep.2 | chr8 | 30601669 | 30601669 | G | A | 9196 | 3.81% |
| Mel.41.Rep.2 | chr11 | 62414149 | 62414149 | C | T | 246 | 8.54% |
| Mel.41.Rep.2 | chr19 | 4247060 | 4247060 | G | T | 6000 | 5.07% |
| Mel.41.Rep.2 | chrX | 48830734 | 48830734 | G | T | 10040 | 7.51% |
| Mel.42.Rep.1 | chr7 | 140453136 | 140453136 | A | T | 5562 | 45.16% |
| Mel.42.Rep.2 | chr7 | 140453136 | 140453136 | A | T | 6291 | 47.66% |
| Mel.42.Rep.2 | chr16 | 83841525 | 83841525 | C | T | 3420 | 4.18% |
| Mel.43.Rep.1 | chr1 | 153963239 | 153963239 | C | T | 6683 | 4.67% |
| Mel.43.Rep.1 | chr2 | 32390905 | 32390905 | C | T | 3167 | 6.95% |
| Mel.43.Rep.1 | chr3 | 16306504 | 16306504 | C | T | 5374 | 31.17% |
| Mel.43.Rep.1 | chr8 | 125551344 | 125551344 | C | T | 159 | 88.05% |
| Mel.43.Rep.1 | chr11 | 46958262 | 46958262 | C | T | 4978 | 13.32% |
| Mel.43.Rep.1 | chr12 | 7080017 | 7080017 | G | A | 5245 | 19.32% |
| Mel.43.Rep.1 | chr12 | 34175405 | 34175405 | C | T | 4604 | 32.04% |
| Mel.43.Rep.1 | chr14 | 53173818 | 53173818 | G | A | 684 | 35.23% |
| Mel.43.Rep.1 | chr16 | 83841525 | 83841525 | C | T | 2246 | 34.46% |
| Mel.43.Rep.1 | chr19 | 13885240 | 13885240 | C | T | 4770 | 24.21% |
| Mel.43.Rep.1 | chr19 | 13885241 | 13885241 | C | T | 4799 | 23.94% |
| Mel.43.Rep.1 | chr19 | 48248748 | 48248748 | G | A | 4958 | 8.86% |
| Mel.43.Rep.2 | chr1 | 153963239 | 153963239 | C | T | 7713 | 5.72% |
| Mel.43.Rep.2 | chr1 | 155904250 | 155904250 | C | T | 7567 | 3.25% |
| Mel.43.Rep.2 | chr2 | 32390905 | 32390905 | C | T | 4537 | 36.01% |
| Mel.43.Rep.2 | chr3 | 16306504 | 16306504 | C | T | 6044 | 62.44% |
| Mel.43.Rep.2 | chr3 | 124449234 | 124449234 | G | A | 5183 | 32.52% |
| Mel.43.Rep.2 | chr10 | 7830002 | 7830002 | G | A | 2770 | 3.50% |
| Mel.43.Rep.2 | chr11 | 99932099 | 99932099 | C | T | 7832 | 8.53% |
| Mel.43.Rep.2 | chr12 | 7080017 | 7080017 | G | A | 5516 | 13.22% |
| Mel.43.Rep.2 | chr12 | 34175405 | 34175405 | C | T | 5471 | 17.79% |
| Mel.43.Rep.2 | chr14 | 53173818 | 53173818 | G | A | 658 | 64.44% |
| Mel.43.Rep.2 | chr19 | 13885240 | 13885240 | C | T | 5274 | 13.69% |
| Mel.43.Rep.2 | chr19 | 13885241 | 13885241 | C | T | 5284 | 19.11% |
| Mel.44.Rep.1 | chr7 | 140453136 | 140453136 | A | T | 3524 | 82.89% |
| Mel.44.Rep.2 | chr3 | 16306504 | 16306504 | C | T | 4605 | 42.48% |
| Mel.44.Rep.2 | chr3 | 16306505 | 16306505 | C | T | 4605 | 42.63% |
| Mel.45.Rep.1 | chr2 | 10830114 | 10830114 | C | T | 4156 | 9.50% |
| Mel.45.Rep.1 | chr5 | 145826780 | 145826780 | C | T | 1480 | 14.93% |
| Mel.45.Rep.1 | chr7 | 39605969 | 39605969 | G | A | 4574 | 4.15% |
| Mel.45.Rep.1 | chr7 | 39605970 | 39605970 | G | A | 4526 | 4.15% |
| Mel.45.Rep.1 | chr7 | 56174183 | 56174183 | G | A | 5396 | 41.07% |
| Mel.45.Rep.1 | chr13 | 41345346 | 41345346 | C | T | 3763 | 27.32% |
| Mel.45.Rep.1 | chr19 | 4247060 | 4247060 | G | A | 2789 | 5.20% |
| Mel.45.Rep.1 | chr19 | 13885241 | 13885241 | C | T | 4260 | 6.22% |
| Mel.45.Rep.1 | chr22 | 43011001 | 43011001 | G | A | 4055 | 27.40% |
| Mel.45.Rep.1 | chr22 | 43011002 | 43011002 | G | A | 4052 | 27.12% |
| Mel.45.Rep.2 | chr1 | 55181528 | 55181528 | G | A | 7142 | 7.01% |
| Mel.45.Rep.2 | chr4 | 152020703 | 152020703 | G | A | 6461 | 11.64% |
| Mel.45.Rep.2 | chr5 | 145826780 | 145826780 | C | T | 1188 | 81.80% |
| Mel.45.Rep.2 | chr19 | 10514261 | 10514261 | G | A | 964 | 6.22% |
| Mel.45.Rep.2 | chr19 | 17970682 | 17970682 | C | T | 6025 | 7.83% |
| Mel.46.Rep.1 | chr5 | 150080667 | 150080667 | C | T | 3200 | 33.60% |
| Mel.46.Rep.2 | chr2 | 32390905 | 32390905 | C | T | 7010 | 10.39% |
| Mel.46.Rep.2 | chr5 | 150080667 | 150080667 | C | T | 7148 | 54.45% |
| Mel.47.Rep.1 | chr7 | 39605969 | 39605969 | G | A | 2109 | 13.56% |
| Mel.47.Rep.1 | chr19 | 3435234 | 3435234 | C | T | 3603 | 12.07% |
| Mel.47.Rep.2 | chr19 | 10514253 | 10514253 | G | A | 3735 | 41.31% |
| Mel.47.Rep.2 | chr19 | 10514261 | 10514261 | G | A | 3610 | 34.16% |
| Mel.48.Rep.1 | chr1 | 25559064 | 25559064 | C | T | 3349 | 58.45% |
| Mel.48.Rep.1 | chr2 | 74682175 | 74682175 | C | T | 3991 | 38.34% |
| Mel.48.Rep.1 | chr3 | 16306504 | 16306504 | C | T | 4471 | 81.25% |
| Mel.48.Rep.1 | chr3 | 16306505 | 16306505 | C | T | 4472 | 81.60% |
| Mel.48.Rep.1 | chr7 | 39605969 | 39605969 | G | A | 3732 | 63.40% |
| Mel.48.Rep.1 | chr7 | 56174183 | 56174183 | G | A | 5336 | 61.35% |
| Mel.48.Rep.1 | chr8 | 30601668 | 30601668 | G | A | 3515 | 36.33% |
| Mel.48.Rep.1 | chr8 | 30601669 | 30601669 | G | A | 3498 | 37.54% |
| Mel.48.Rep.1 | chr10 | 7830002 | 7830002 | G | A | 4403 | 79.32% |
| Mel.48.Rep.1 | chr19 | 4247072 | 4247072 | C | T | 1242 | 62.88% |
| Mel.48.Rep.2 | chr1 | 25559064 | 25559064 | C | T | 5634 | 71.23% |
| Mel.48.Rep.2 | chr1 | 153963227 | 153963227 | C | T | 7202 | 74.19% |
| Mel.48.Rep.2 | chr2 | 74682175 | 74682175 | C | T | 2055 | 60.29% |
| Mel.48.Rep.2 | chr3 | 16306504 | 16306504 | C | T | 4890 | 56.44% |
| Mel.48.Rep.2 | chr3 | 16306505 | 16306505 | C | T | 4872 | 56.69% |
| Mel.48.Rep.2 | chr7 | 39605969 | 39605969 | G | A | 4609 | 15.75% |
| Mel.48.Rep.2 | chr8 | 30601668 | 30601668 | G | A | 3376 | 59% |
| Mel.48.Rep.2 | chr8 | 30601669 | 30601669 | G | A | 3351 | 66.34% |
| Mel.48.Rep.2 | chr10 | 7830002 | 7830002 | G | A | 5563 | 50.32% |
| Mel.48.Rep.2 | chr12 | 81693130 | 81693130 | C | T | 1577 | 11.67% |
| Mel.48.Rep.2 | chr19 | 4247072 | 4247072 | C | T | 1163 | 95.53% |
| Mel.48.Rep.2 | chr19 | 10514261 | 10514261 | G | A | 3556 | 63.79% |
| Mel.49.Rep.1 | chr1 | 153963227 | 153963227 | C | T | 4115 | 20.19% |
| Mel.49.Rep.1 | chr14 | 53173818 | 53173818 | G | A | 1276 | 58.86% |
| Mel.49.Rep.1 | chr16 | 83841525 | 83841525 | C | A | 262 | 98.47% |
| Mel.49.Rep.1 | chr17 | 30771480 | 30771480 | C | T | 1637 | 10.87% |
| Mel.49.Rep.1 | chr19 | 48248748 | 48248748 | G | A | 3064 | 35.17% |
| Mel.49.Rep.2 | chr1 | 153963227 | 153963227 | C | T | 7807 | 20.21% |
| Mel.49.Rep.2 | chr13 | 41345346 | 41345346 | C | T | 7172 | 82.43% |
| Mel.49.Rep.2 | chr14 | 53173818 | 53173818 | G | A | 2422 | 22.42% |
| Mel.49.Rep.2 | chr16 | 83841525 | 83841525 | C | A | 3865 | 33.76% |
| Mel.49.Rep.2 | chr17 | 30771480 | 30771480 | C | T | 6532 | 12.97% |
| Mel.50.Rep.1 | chr1 | 894683 | 894683 | C | T | 495 | 70.51% |
| Mel.50.Rep.1 | chr4 | 69093838 | 69093838 | C | T | 3535 | 19.60% |
| Mel.50.Rep.1 | chr4 | 152020712 | 152020712 | G | A | 3661 | 8.58% |
| Mel.50.Rep.1 | chr7 | 140453136 | 140453136 | A | T | 3354 | 12.52% |
| Mel.50.Rep.1 | chr7 | 140453137 | 140453137 | C | T | 3356 | 12.57% |
| Mel.50.Rep.1 | chr12 | 53473217 | 53473217 | C | T | 812 | 3.82% |
| Mel.50.Rep.1 | chr19 | 17970682 | 17970682 | C | T | 3740 | 13.93% |
| Mel.50.Rep.2 | chr1 | 153963228 | 153963228 | C | T | 5783 | 3.01% |
| Mel.50.Rep.2 | chr3 | 16306504 | 16306504 | C | T | 3228 | 5.27% |
| Mel.50.Rep.2 | chr3 | 16306505 | 16306505 | C | T | 3233 | 5.26% |
| Mel.50.Rep.2 | chr4 | 69093838 | 69093838 | C | T | 3643 | 17.90% |
| Mel.50.Rep.2 | chr7 | 140453136 | 140453136 | A | T | 2526 | 63.14% |
| Mel.50.Rep.2 | chr7 | 140453137 | 140453137 | C | T | 2518 | 63.07% |
| Mel.50.Rep.2 | chr8 | 68931944 | 68931944 | C | T | 3176 | 13.60% |
| Mel.51.Rep.1 | chr16 | 29802021 | 29802021 | G | A | 2460 | 61.63% |
| Mel.51.Rep.1 | chr19 | 17970682 | 17970682 | C | T | 4053 | 80.49% |
| Mel.51.Rep.1 | chr19 | 51501259 | 51501259 | G | A | 6678 | 21.79% |
| Mel.51.Rep.1 | chr22 | 43010992 | 43010992 | G | A | 1110 | 66.22% |
| Mel.52.Rep.1 | chr1 | 115256529 | 115256529 | T | C | 3389 | 51.96% |
| Mel.52.Rep.1 | chr3 | 16306505 | 16306505 | C | T | 1614 | 34.39% |
| Mel.52.Rep.2 | chr1 | 115256529 | 115256529 | T | C | 4438 | 48.31% |
| Mel.52.Rep.2 | chr3 | 16306505 | 16306505 | C | T | 2351 | 29.69% |
| Mel.52.Rep.2 | chr16 | 67440234 | 67440234 | G | A | 3289 | 5.38% |
| Mel.53.Rep.1 | chr1 | 115256530 | 115256530 | G | T | 4391 | 8.59% |
| Mel.53.Rep.1 | chr1 | 153963227 | 153963227 | C | T | 3994 | 19.85% |
| Mel.53.Rep.1 | chr1 | 155904250 | 155904250 | C | T | 3795 | 26.53% |
| Mel.53.Rep.1 | chr8 | 30601668 | 30601668 | G | A | 3085 | 32.90% |
| Mel.53.Rep.1 | chr19 | 17970682 | 17970682 | C | T | 2958 | 19.61% |
| Mel.53.Rep.1 | chr22 | 43011001 | 43011001 | G | A | 3128 | 20.24% |
| Mel.53.Rep.1 | chr22 | 43011002 | 43011002 | G | A | 3109 | 20.36% |
| Mel.53.Rep.2 | chr1 | 115256530 | 115256530 | G | T | 5816 | 45.64% |
| Mel.53.Rep.2 | chr1 | 153963227 | 153963227 | C | T | 5675 | 46.66% |
| Mel.53.Rep.2 | chr8 | 30601668 | 30601668 | G | A | 4387 | 10.60% |
| Mel.53.Rep.2 | chr16 | 29802026 | 29802026 | G | A | 1172 | 20.99% |
| Mel.53.Rep.2 | chr22 | 43011001 | 43011001 | G | A | 4088 | 29.82% |
| Mel.53.Rep.2 | chr22 | 43011002 | 43011002 | G | A | 4072 | 29.76% |
| Mel.54.Rep.1 | chr1 | 115256529 | 115256529 | T | C | 3854 | 94.37% |
| Mel.54.Rep.1 | chr3 | 16306505 | 16306505 | C | T | 3143 | 13.24% |
| Mel.54.Rep.1 | chr4 | 152020703 | 152020703 | G | A | 3350 | 26.76% |
| Mel.54.Rep.1 | chr5 | 145826780 | 145826780 | C | T | 725 | 3.59% |
| Mel.54.Rep.1 | chr5 | 150080667 | 150080667 | C | T | 3975 | 17.41% |
| Mel.54.Rep.1 | chr9 | 131038409 | 131038409 | G | A | 2373 | 94.18% |
| Mel.54.Rep.1 | chr10 | 7830002 | 7830002 | G | A | 3108 | 77.80% |
| Mel.54.Rep.1 | chr17 | 30771480 | 30771480 | C | T | 1216 | 5.26% |
| Mel.54.Rep.1 | chr19 | 4247045 | 4247045 | G | A | 1714 | 12.49% |
| Mel.54.Rep.1 | chr19 | 17970682 | 17970682 | C | T | 2406 | 85.24% |
| Mel.54.Rep.1 | chr22 | 43011001 | 43011001 | G | A | 2764 | 13.60% |
| Mel.54.Rep.2 | chr1 | 115256529 | 115256529 | T | C | 4761 | 33.19% |
| Mel.54.Rep.2 | chr3 | 16306505 | 16306505 | C | T | 3150 | 41.14% |
| Mel.54.Rep.2 | chr5 | 150080667 | 150080667 | C | T | 3844 | 10.09% |
| Mel.54.Rep.2 | chr8 | 125551345 | 125551345 | C | T | 2121 | 40.10% |
| Mel.54.Rep.2 | chr9 | 131038409 | 131038409 | G | A | 2224 | 85.57% |
| Mel.54.Rep.2 | chr10 | 7830002 | 7830002 | G | A | 190 | 94.74% |
| Mel.54.Rep.2 | chr12 | 7080017 | 7080017 | G | A | 3718 | 20.55% |
| Mel.54.Rep.2 | chr17 | 30771480 | 30771480 | C | T | 2153 | 75.31% |
| Mel.54.Rep.2 | chr19 | 4247045 | 4247045 | G | A | 1747 | 3.32% |
| Mel.54.Rep.2 | chr22 | 43011001 | 43011001 | G | A | 3611 | 22.85% |
| Mel.55.Rep.1 | chr3 | 16306504 | 16306504 | C | T | 3390 | 26.19% |
| Mel.55.Rep.1 | chr7 | 56174183 | 56174183 | G | A | 3679 | 11.96% |
| Mel.55.Rep.1 | chr8 | 125551344 | 125551344 | C | T | 1304 | 60.66% |
| Mel.55.Rep.2 | chr3 | 16306504 | 16306504 | C | T | 3208 | 30.02% |
| Mel.55.Rep.2 | chr7 | 56174183 | 56174183 | G | A | 3801 | 67.65% |
| Mel.55.Rep.2 | chr7 | 140453136 | 140453136 | A | T | 2093 | 50.69% |
| Mel.55.Rep.2 | chr12 | 7080016 | 7080016 | G | A | 4226 | 36.85% |
| Mel.57.Rep.1 | chr2 | 32390904 | 32390904 | C | T | 2048 | 9.18% |
| Mel.57.Rep.1 | chr2 | 32390905 | 32390905 | C | T | 2049 | 8.98% |
| Mel.57.Rep.1 | chr3 | 16306504 | 16306504 | C | T | 2154 | 10.82% |
| Mel.57.Rep.1 | chr3 | 16306505 | 16306505 | C | T | 2170 | 10.83% |
| Mel.57.Rep.1 | chr4 | 152020703 | 152020703 | G | A | 2509 | 14.95% |
| Mel.57.Rep.2 | chr4 | 152020703 | 152020703 | G | A | 3947 | 6.11% |
| Mel.57.Rep.2 | chr7 | 140453136 | 140453136 | A | T | 3206 | 15.85% |
| Mel.57.Rep.2 | chr9 | 131038409 | 131038409 | G | A | 3495 | 26.02% |
| Mel.57.Rep.2 | chr19 | 4247041 | 4247041 | G | A | 1550 | 10.71% |
| Mel.58.Rep.1 | chr4 | 152020712 | 152020712 | G | A | 3076 | 3.15% |
| Mel.58.Rep.1 | chr7 | 140453136 | 140453136 | A | T | 1856 | 32.49% |
| Mel.58.Rep.2 | chr1 | 894682 | 894682 | C | T | 673 | 98.37% |
| Mel.58.Rep.2 | chr4 | 152020703 | 152020703 | G | A | 3489 | 10.81% |
| Mel.58.Rep.2 | chr13 | 41345350 | 41345350 | C | A | 3533 | 4.50% |
| Mel.59.Rep.1 | chr2 | 32390904 | 32390904 | C | T | 2839 | 37.34% |
| Mel.59.Rep.1 | chr3 | 16306504 | 16306504 | C | T | 2585 | 44.53% |
| Mel.59.Rep.2 | chr3 | 48481605 | 48481605 | G | A | 2880 | 6.22% |
| Mel.59.Rep.2 | chr19 | 4247045 | 4247045 | G | A | 1764 | 19.10% |
| Mel.60.Rep.1 | chr3 | 16306504 | 16306504 | C | T | 12320 | 23.08% |
| Mel.60.Rep.1 | chr12 | 53473218 | 53473219 | CC | C | 2270 | 18.17% |
| Mel.60.Rep.1 | chr12 | 117923349 | 117923349 | G | GG | 1842 | 4.92% |
| Mel.60.Rep.2 | chr1 | 115256529 | 115256529 | T | A | 8696 | 14.33% |
| Mel.60.Rep.2 | chr3 | 16306504 | 16306504 | C | T | 10517 | 66.95% |
| Mel.60.Rep.2 | chr3 | 16306505 | 16306505 | C | T | 10508 | 31.40% |
| Mel.61.Rep.1 | chr3 | 16306504 | 16306504 | C | T | 12685 | 25.06% |
| Mel.61.Rep.1 | chr3 | 16306505 | 16306505 | C | T | 12701 | 25.12% |
| Mel.61.Rep.1 | chr19 | 48248748 | 48248748 | G | A | 7861 | 9.14% |
| Mel.61.Rep.2 | chr1 | 55181508 | 55181508 | G | A | 16710 | 3.48% |
| Mel.61.Rep.2 | chr2 | 105953996 | 105953996 | C | T | 4803 | 6.68% |
| Mel.61.Rep.2 | chr3 | 16306504 | 16306504 | C | T | 28412 | 22.98% |
| Mel.61.Rep.2 | chr3 | 16306505 | 16306505 | C | T | 28435 | 22.95% |
| Mel.61.Rep.2 | chr19 | 48248748 | 48248748 | G | A | 9530 | 38.61% |
| Mel.62.Rep.1 | chr5 | 150080667 | 150080667 | C | T | 7438 | 78.39% |
| Mel.62.Rep.2 | chr4 | 152020703 | 152020703 | G | A | 11243 | 3.28% |
| Mel.62.Rep.2 | chr5 | 150080667 | 150080667 | C | T | 8113 | 41.71% |
| Mel.63.Rep.1 | chr1 | 153963239 | 153963239 | C | T | 15858 | 15.31% |
| Mel.63.Rep.1 | chr1 | 155904250 | 155904250 | C | T | 10704 | 87.48% |
| Mel.63.Rep.1 | chr2 | 32390905 | 32390905 | C | T | 7268 | 15.58% |
| Mel.63.Rep.1 | chr2 | 74682175 | 74682175 | C | T | 8106 | 16.36% |
| Mel.63.Rep.1 | chr3 | 16306504 | 16306504 | C | T | 12670 | 3.81% |
| Mel.63.Rep.1 | chr3 | 16306505 | 16306505 | C | T | 12699 | 3.81% |
| Mel.63.Rep.1 | chr4 | 69093838 | 69093838 | C | T | 30018 | 74.14% |
| Mel.63.Rep.1 | chr7 | 56174183 | 56174183 | G | A | 10549 | 77.27% |
| Mel.63.Rep.1 | chr8 | 30601668 | 30601668 | G | C | 8377 | 74.51% |
| Mel.63.Rep.1 | chr8 | 30601669 | 30601669 | G | A | 8383 | 74.34% |
| Mel.63.Rep.1 | chr9 | 130700157 | 130700157 | C | T | 1859 | 98.76% |
| Mel.63.Rep.1 | chr12 | 7080016 | 7080016 | G | A | 12173 | 39.63% |
| Mel.63.Rep.1 | chr13 | 41345346 | 41345346 | C | T | 20103 | 43.01% |
| Mel.63.Rep.1 | chr14 | 53173818 | 53173818 | G | A | 1562 | 6.34% |
| Mel.63.Rep.1 | chr16 | 83841526 | 83841526 | C | T | 3313 | 28.20% |
| Mel.63.Rep.1 | chr17 | 56769917 | 56769917 | C | T | 22068 | 61.93% |
| Mel.63.Rep.2 | chr1 | 894682 | 894682 | C | T | 191 | 28.27% |
| Mel.63.Rep.2 | chr1 | 153963239 | 153963239 | C | T | 7844 | 64.14% |
| Mel.63.Rep.2 | chr2 | 32390905 | 32390905 | C | T | 12228 | 15.69% |
| Mel.63.Rep.2 | chr3 | 16306504 | 16306504 | C | T | 9460 | 15.92% |
| Mel.63.Rep.2 | chr3 | 16306505 | 16306505 | C | T | 9461 | 15.88% |
| Mel.63.Rep.2 | chr4 | 69093838 | 69093838 | C | T | 30505 | 21.85% |
| Mel.63.Rep.2 | chr4 | 152020703 | 152020703 | G | A | 5898 | 58.93% |
| Mel.63.Rep.2 | chr7 | 56174183 | 56174183 | G | A | 10793 | 44.94% |
| Mel.63.Rep.2 | chr8 | 30601668 | 30601668 | G | C | 5043 | 54.67% |
| Mel.63.Rep.2 | chr8 | 30601669 | 30601669 | G | A | 5041 | 54.71% |
| Mel.63.Rep.2 | chr8 | 125551345 | 125551345 | C | T | 340 | 38.24% |
| Mel.63.Rep.2 | chr12 | 7080016 | 7080016 | G | A | 9991 | 66.83% |
| Mel.63.Rep.2 | chr13 | 41345346 | 41345346 | C | T | 18670 | 42.11% |
| Mel.63.Rep.2 | chr14 | 53173818 | 53173818 | G | A | 1024 | 15.92% |
| Mel.63.Rep.2 | chr16 | 2510095 | 2510095 | G | A | 5893 | 97.56% |
| Mel.63.Rep.2 | chr16 | 2510096 | 2510096 | G | A | 5918 | 9.09% |
| Mel.63.Rep.2 | chr16 | 83841526 | 83841526 | C | T | 3622 | 63.09% |
| Mel.63.Rep.2 | chr17 | 30771480 | 30771480 | C | T | 3793 | 94.67% |
| Mel.63.Rep.2 | chr17 | 56769917 | 56769917 | C | T | 24249 | 28.49% |
| Mel.64.Rep.1 | chr3 | 16306505 | 16306505 | C | T | 12528 | 64.88% |
| Mel.64.Rep.1 | chr3 | 68802215 | 68802215 | G | A | 3999 | 23.66% |
| Mel.64.Rep.1 | chr13 | 41345355 | 41345355 | C | T | 19677 | 19.64% |
| Mel.64.Rep.1 | chr16 | 83841526 | 83841526 | C | T | 1962 | 67.28% |
| Mel.64.Rep.1 | chr19 | 48248737 | 48248737 | G | A | 1632 | 54.08% |
| Mel.64.Rep.2 | chr3 | 16306505 | 16306505 | C | T | 8707 | 4.01% |
| Mel.64.Rep.2 | chr3 | 68802215 | 68802215 | G | A | 2934 | 73.94% |
| Mel.64.Rep.2 | chr13 | 41345355 | 41345355 | C | T | 12352 | 45.11% |
| Mel.64.Rep.2 | chr16 | 83841526 | 83841526 | C | T | 2298 | 32.38% |
| Mel.64.Rep.2 | chr19 | 4247060 | 4247060 | G | A | 297 | 99.33% |
| Mel.65.Rep.1 | chr1 | 115256529 | 115256529 | T | C | 6843 | 4.50% |
| Mel.65.Rep.1 | chr2 | 10830114 | 10830114 | C | T | 19048 | 40.64% |
| Mel.65.Rep.1 | chr2 | 10830118 | 10830118 | G | A | 18990 | 8.49% |
| Mel.65.Rep.1 | chr7 | 56174183 | 56174183 | G | A | 5659 | 3.62% |
| Mel.65.Rep.1 | chr9 | 131038409 | 131038409 | G | A | 6168 | 33.67% |
| Mel.65.Rep.2 | chr2 | 10830114 | 10830114 | C | T | 24047 | 39.17% |
| Mel.65.Rep.2 | chr17 | 30771480 | 30771480 | C | T | 7204 | 5.43% |
| Mel.65.Rep.2 | chr20 | 34129795 | 34129795 | G | A | 11304 | 10.02% |
| Mel.66.Rep.1 | chr12 | 7080016 | 7080016 | G | A | 8983 | 14.04% |
| Mel.67.Rep.1 | chr3 | 16306505 | 16306505 | C | T | 8622 | 39.35% |
| Mel.67.Rep.1 | chr4 | 152020703 | 152020703 | G | A | 10464 | 12.64% |
| Mel.67.Rep.1 | chr19 | 17970682 | 17970682 | C | T | 14119 | 37.47% |
| Mel.67.Rep.1 | chr22 | 43011001 | 43011001 | G | A | 8261 | 13.21% |
| Mel.67.Rep.2 | chr1 | 115256529 | 115256529 | T | C | 10354 | 19.64% |
| Mel.67.Rep.2 | chr1 | 153963181 | 153963181 | C | T | 10848 | 10.22% |
| Mel.67.Rep.2 | chr3 | 16306505 | 16306505 | C | T | 8361 | 46.79% |
| Mel.67.Rep.2 | chr3 | 67048644 | 67048644 | C | T | 9763 | 7.29% |
| Mel.67.Rep.2 | chr7 | 39605969 | 39605969 | G | A | 158 | 55.70% |
| Mel.67.Rep.2 | chr7 | 39605970 | 39605970 | G | A | 156 | 56.41% |
| Mel.67.Rep.2 | chr19 | 17970682 | 17970682 | C | T | 14239 | 22.64% |
| Mel.68.Rep.1 | chr1 | 153276253 | 153276253 | G | A | 13496 | 25.99% |
| Mel.68.Rep.1 | chr1 | 153963239 | 153963239 | C | T | 11385 | 28.65% |
| Mel.68.Rep.1 | chr1 | 155904250 | 155904250 | C | T | 9222 | 70.52% |
| Mel.68.Rep.1 | chr1 | 155904276 | 155904276 | C | T | 9200 | 49.61% |
| Mel.68.Rep.1 | chr6 | 30640795 | 30640795 | G | A | 6855 | 32.59% |
| Mel.68.Rep.1 | chr6 | 30640796 | 30640796 | G | A | 6846 | 11.34% |
| Mel.68.Rep.1 | chr8 | 30601669 | 30601669 | G | A | 7735 | 14.34% |
| Mel.68.Rep.1 | chr12 | 132537871 | 132537871 | C | T | 1324 | 4.23% |
| Mel.68.Rep.1 | chr16 | 2510096 | 2510096 | G | A | 1665 | 26.31% |
| Mel.68.Rep.1 | chr19 | 4247045 | 4247045 | G | A | 4257 | 14.85% |
| Mel.68.Rep.1 | chr19 | 4247072 | 4247072 | C | T | 4238 | 18.43% |
| Mel.68.Rep.1 | chr19 | 48248748 | 48248748 | G | A | 5255 | 4.59% |
| Mel.68.Rep.2 | chr1 | 153276253 | 153276253 | G | A | 11239 | 41.52% |
| Mel.68.Rep.2 | chr1 | 153963239 | 153963239 | C | T | 9876 | 14.39% |
| Mel.68.Rep.2 | chr1 | 155904250 | 155904250 | C | T | 7500 | 75.21% |
| Mel.68.Rep.2 | chr1 | 155904276 | 155904276 | C | T | 7499 | 37.40% |
| Mel.68.Rep.2 | chr6 | 30640795 | 30640795 | G | A | 6143 | 5.29% |
| Mel.68.Rep.2 | chr6 | 30640796 | 30640796 | G | A | 6115 | 14.39% |
| Mel.68.Rep.2 | chr9 | 131038409 | 131038409 | G | A | 6833 | 9.21% |
| Mel.68.Rep.2 | chr16 | 2510096 | 2510096 | G | A | 4066 | 23.18% |
| Mel.68.Rep.2 | chr19 | 4247045 | 4247045 | G | A | 3657 | 26.39% |
| Mel.68.Rep.2 | chr19 | 4247072 | 4247072 | C | T | 3633 | 58.35% |
| Mel.68.Rep.2 | chr19 | 48248748 | 48248748 | G | A | 6001 | 59.21% |
| Mel.69.Rep.1 | chr2 | 32390904 | 32390904 | C | T | 7918 | 4.96% |
| Mel.69.Rep.1 | chr2 | 32390905 | 32390905 | C | T | 7944 | 5.02% |
| Mel.69.Rep.1 | chr5 | 145826780 | 145826780 | C | T | 632 | 15.51% |
| Mel.69.Rep.1 | chr7 | 140453136 | 140453136 | A | T | 670 | 5.22% |
| Mel.69.Rep.1 | chr11 | 46958262 | 46958262 | C | T | 5856 | 10.95% |
| Mel.69.Rep.1 | chr17 | 37356494 | 37356494 | G | A | 2364 | 42.30% |
| Mel.69.Rep.1 | chr19 | 3435234 | 3435234 | C | T | 6396 | 37.35% |
| Mel.69.Rep.1 | chr19 | 3435235 | 3435235 | C | T | 6403 | 7.20% |
| Mel.69.Rep.2 | chr2 | 74682176 | 74682176 | C | T | 4325 | 5.32% |
| Mel.69.Rep.2 | chr5 | 145826780 | 145826780 | C | T | 1016 | 27.07% |
| Mel.69.Rep.2 | chr7 | 140453136 | 140453136 | A | T | 2231 | 29.18% |
| Mel.69.Rep.2 | chr9 | 130700157 | 130700157 | C | T | 3967 | 23.37% |
| Mel.69.Rep.2 | chr11 | 46958262 | 46958262 | C | T | 6136 | 28.61% |
| Mel.69.Rep.2 | chr17 | 37356494 | 37356494 | G | A | 550 | 10.18% |
| Mel.69.Rep.2 | chr19 | 3435234 | 3435234 | C | T | 7225 | 64.19% |
| Mel.70.Rep.1 | chr3 | 16306504 | 16306504 | C | T | 4149 | 62.11% |
| Mel.70.Rep.1 | chr3 | 16306505 | 16306505 | C | T | 4140 | 62.39% |
| Mel.70.Rep.1 | chr19 | 4247072 | 4247072 | C | T | 179 | 99.44% |
| Mel.70.Rep.1 | chr19 | 38202422 | 38202422 | G | A | 17860 | 11.92% |
| Mel.71.Rep.1 | chr4 | 152020703 | 152020703 | G | A | 6594 | 12.13% |
| Mel.71.Rep.1 | chr5 | 145826780 | 145826780 | C | T | 2145 | 10.77% |
| Mel.71.Rep.1 | chr7 | 56174183 | 56174183 | G | A | 6634 | 66.05% |
| Mel.71.Rep.1 | chr7 | 140453136 | 140453136 | A | T | 4948 | 15.18% |
| Mel.71.Rep.1 | chr7 | 140453137 | 140453137 | C | T | 4971 | 14.99% |
| Mel.71.Rep.1 | chr13 | 41345346 | 41345346 | C | T | 6927 | 12.66% |
| Mel.71.Rep.1 | chr14 | 53173818 | 53173818 | G | A | 809 | 14.46% |
| Mel.71.Rep.2 | chr5 | 145826780 | 145826780 | C | T | 1145 | 59.88% |
| Mel.71.Rep.2 | chr7 | 56174183 | 56174183 | G | A | 3813 | 27.23% |
| Mel.71.Rep.2 | chr7 | 140453136 | 140453136 | A | T | 1536 | 83.92% |
| Mel.71.Rep.2 | chr7 | 140453137 | 140453137 | C | T | 1529 | 83.91% |
| Mel.71.Rep.2 | chr13 | 41345346 | 41345346 | C | T | 4486 | 17.08% |
| Mel.71.Rep.2 | chr16 | 2510095 | 2510095 | G | A | 115 | 93.91% |
| Mel.71.Rep.2 | chr16 | 2510096 | 2510096 | G | A | 114 | 94.74% |
| Mel.71.Rep.2 | chr19 | 3435235 | 3435235 | C | T | 3219 | 12.05% |
| Mel.71.Rep.2 | chr19 | 38202425 | 38202425 | G | A | 9135 | 14.38% |
| Mel.72.Rep.2 | chr1 | 25559064 | 25559064 | C | T | 1297 | 98.38% |
| Mel.72.Rep.2 | chr13 | 41345346 | 41345346 | C | T | 11599 | 99.63% |
| Mel.73.Rep.1 | chr1 | 25559063 | 25559063 | C | T | 8590 | 6.78% |
| Mel.73.Rep.1 | chr1 | 115256530 | 115256530 | G | A | 7618 | 4.99% |
| Mel.73.Rep.1 | chr1 | 153963227 | 153963227 | C | T | 7846 | 3.71% |
| Mel.73.Rep.1 | chr2 | 10830118 | 10830118 | G | A | 4761 | 9.39% |
| Mel.73.Rep.1 | chr2 | 74682175 | 74682175 | C | T | 6920 | 13.44% |
| Mel.73.Rep.1 | chr7 | 140453136 | 140453136 | A | T | 4875 | 25.48% |
| Mel.73.Rep.2 | chr1 | 25559063 | 25559063 | C | T | 6379 | 7.89% |
| Mel.73.Rep.2 | chr1 | 55181528 | 55181528 | G | A | 5487 | 10.44% |
| Mel.73.Rep.2 | chr5 | 26885701 | 26885701 | C | T | 3215 | 3.73% |
| Mel.73.Rep.2 | chr7 | 140453136 | 140453136 | A | T | 4073 | 11.51% |
| Mel.73.Rep.2 | chr11 | 46958262 | 46958262 | C | T | 4159 | 44.06% |
| Mel.73.Rep.2 | chr17 | 30771480 | 30771480 | C | T | 3102 | 23.98% |
| Mel.74.Rep.1 | chr7 | 140453136 | 140453136 | A | T | 2052 | 57.36% |
| Mel.74.Rep.1 | chr13 | 41345346 | 41345346 | C | T | 4268 | 39.51% |
| Mel.74.Rep.2 | chr7 | 53103781 | 53103781 | C | A | 1925 | 3.38% |
| Mel.74.Rep.2 | chr7 | 140453136 | 140453136 | A | T | 2170 | 65.62% |
| Mel.74.Rep.2 | chr11 | 8704338 | 8704338 | C | T | 4551 | 33.68% |
| Mel.74.Rep.2 | chr13 | 41345346 | 41345346 | C | T | 4476 | 83.17% |
| Mel.75.Rep.1 | chr1 | 153276253 | 153276253 | G | A | 9859 | 51.22% |
| Mel.75.Rep.1 | chr2 | 32390904 | 32390904 | C | T | 8495 | 16.44% |
| Mel.75.Rep.1 | chr7 | 56174183 | 56174183 | G | A | 4875 | 84.16% |
| Mel.75.Rep.1 | chr8 | 125551345 | 125551345 | C | T | 4077 | 12.14% |
| Mel.75.Rep.1 | chr16 | 2510095 | 2510095 | G | A | 4780 | 38.67% |
| Mel.75.Rep.1 | chr20 | 34129795 | 34129795 | G | A | 3707 | 77.26% |
| Mel.75.Rep.2 | chr1 | 153276253 | 153276253 | G | A | 5149 | 46.02% |
| Mel.75.Rep.2 | chr1 | 155904250 | 155904250 | C | T | 2031 | 87.78% |
| Mel.75.Rep.2 | chr2 | 74682176 | 74682176 | C | T | 4367 | 4.01% |
| Mel.75.Rep.2 | chr3 | 16306504 | 16306504 | C | T | 2467 | 20.59% |
| Mel.75.Rep.2 | chr3 | 16306505 | 16306505 | C | T | 2473 | 20.70% |
| Mel.75.Rep.2 | chr7 | 56174183 | 56174183 | G | A | 3041 | 40.81% |
| Mel.75.Rep.2 | chr8 | 125551345 | 125551345 | C | T | 3508 | 80.42% |
| Mel.75.Rep.2 | chr12 | 132537871 | 132537871 | C | T | 265 | 8.68% |
| Mel.75.Rep.2 | chr20 | 34129795 | 34129795 | G | A | 2743 | 3.97% |
| Mel.76.Rep.1 | chr1 | 115256529 | 115256529 | T | A | 5185 | 29.46% |
| Mel.76.Rep.1 | chr1 | 153963222 | 153963222 | G | A | 6053 | 3.78% |
| Mel.76.Rep.1 | chr1 | 153963239 | 153963239 | C | T | 6028 | 22.69% |
| Mel.76.Rep.1 | chr6 | 30640795 | 30640795 | G | A | 3911 | 26.18% |
| Mel.76.Rep.1 | chr7 | 56174183 | 56174183 | G | A | 4492 | 15.05% |
| Mel.76.Rep.1 | chr7 | 56174204 | 56174204 | C | T | 4461 | 19.91% |
| Mel.76.Rep.1 | chr13 | 41345355 | 41345355 | C | T | 5822 | 5.08% |
| Mel.76.Rep.1 | chr16 | 67694211 | 67694211 | C | T | 130 | 35.38% |
| Mel.76.Rep.1 | chr19 | 4247078 | 4247078 | G | A | 2675 | 4.53% |
| Mel.76.Rep.1 | chr19 | 17970682 | 17970682 | C | T | 4820 | 12.37% |
| Mel.76.Rep.1 | chr20 | 34129792 | 34129792 | G | A | 4114 | 9.77% |
| Mel.76.Rep.2 | chr1 | 25559064 | 25559064 | C | T | 5023 | 26% |
| Mel.76.Rep.2 | chr1 | 115256529 | 115256529 | T | A | 4734 | 15.12% |
| Mel.76.Rep.2 | chr1 | 153963239 | 153963239 | C | T | 5913 | 26.57% |
| Mel.76.Rep.2 | chr1 | 231114784 | 231114784 | C | T | 1002 | 10.58% |
| Mel.76.Rep.2 | chr2 | 10830117 | 10830117 | G | A | 2316 | 6.17% |
| Mel.76.Rep.2 | chr3 | 68802177 | 68802177 | G | A | 3665 | 17.11% |
| Mel.76.Rep.2 | chr8 | 125551321 | 125551321 | G | A | 334 | 5.99% |
| Mel.76.Rep.2 | chr16 | 2510096 | 2510096 | G | A | 1818 | 16.56% |
| Mel.76.Rep.2 | chr17 | 30771480 | 30771480 | C | T | 3807 | 12.24% |
| Mel.76.Rep.2 | chr19 | 4247060 | 4247060 | G | A | 2628 | 21.58% |
| Mel.76.Rep.2 | chr19 | 17970682 | 17970682 | C | T | 4753 | 21.01% |
| Mel.76.Rep.2 | chr19 | 51501258 | 51501258 | G | A | 4504 | 5.35% |
| Mel.76.Rep.2 | chr20 | 34129795 | 34129795 | G | A | 2801 | 5.68% |
| Mel.76.Rep.2 | chr22 | 43010992 | 43010992 | G | A | 4993 | 8.09% |
| Mel.77.Rep.1 | chr1 | 115256530 | 115256530 | G | A | 6284 | 10.18% |
| Mel.77.Rep.1 | chr1 | 153963227 | 153963227 | C | T | 7436 | 36.54% |
| Mel.77.Rep.1 | chr8 | 125551321 | 125551321 | G | A | 5208 | 3.34% |
| Mel.77.Rep.1 | chr8 | 125551345 | 125551345 | C | T | 5227 | 59.33% |
| Mel.77.Rep.1 | chr17 | 37356487 | 37356487 | C | T | 1887 | 3.39% |
| Mel.77.Rep.1 | chr19 | 17970682 | 17970682 | C | T | 6829 | 19.60% |
| Mel.77.Rep.2 | chr1 | 153963227 | 153963227 | C | T | 6013 | 39% |
| Mel.77.Rep.2 | chr3 | 68802220 | 68802220 | G | A | 4149 | 5.52% |
| Mel.77.Rep.2 | chr8 | 125551345 | 125551345 | C | T | 3557 | 76.10% |
| Mel.77.Rep.2 | chr19 | 17970682 | 17970682 | C | T | 5655 | 29.50% |
| Mel.78.Rep.2 | chr4 | 152020712 | 152020712 | G | A | 1705 | 40.59% |
| Mel.79.Rep.1 | chr3 | 16306504 | 16306504 | C | T | 5742 | 42.72% |
| Mel.79.Rep.1 | chr7 | 39605965 | 39605965 | G | A | 4845 | 8.79% |
| Mel.79.Rep.1 | chr7 | 140453136 | 140453136 | A | T | 999 | 30.33% |
| Mel.79.Rep.1 | chr16 | 2510095 | 2510095 | G | A | 3258 | 96.44% |
| Mel.79.Rep.1 | chr16 | 2510096 | 2510096 | G | A | 3266 | 96.08% |
| Mel.79.Rep.1 | chr16 | 67440238 | 67440238 | G | A | 6846 | 8.06% |
| Mel.79.Rep.2 | chr3 | 16306504 | 16306504 | C | T | 5629 | 48.48% |
| Mel.79.Rep.2 | chr7 | 140453136 | 140453136 | A | T | 4109 | 39.79% |
| Mel.79.Rep.2 | chr12 | 53473215 | 53473215 | C | T | 1575 | 5.78% |
| Mel.79.Rep.2 | chr16 | 2510095 | 2510095 | G | A | 4609 | 76.48% |
| Mel.79.Rep.2 | chr16 | 2510096 | 2510096 | G | A | 4600 | 76.85% |
| Mel.79.Rep.2 | chr19 | 38202422 | 38202422 | G | A | 14877 | 8.29% |
| Mel.80.Rep.1 | chr19 | 4247072 | 4247072 | C | T | 1343 | 48.03% |
| Mel.80.Rep.2 | chr1 | 153963239 | 153963239 | C | T | 7359 | 48.50% |
| Mel.80.Rep.2 | chr5 | 150080667 | 150080667 | C | T | 8186 | 35.51% |
| Mel.80.Rep.2 | chr8 | 68931889 | 68931889 | A | G | 4063 | 5.59% |
| Mel.81.Rep.1 | chr1 | 153963227 | 153963227 | C | T | 6934 | 35.02% |
| Mel.81.Rep.1 | chr7 | 140453136 | 140453136 | A | T | 4700 | 48.15% |
| Mel.81.Rep.1 | chr7 | 140453137 | 140453137 | C | T | 4689 | 48.16% |
| Mel.81.Rep.1 | chr8 | 30601668 | 30601668 | G | A | 6387 | 11.34% |
| Mel.81.Rep.1 | chr8 | 30601669 | 30601669 | G | A | 6364 | 11.33% |
| Mel.81.Rep.1 | chr12 | 7080016 | 7080016 | G | A | 4335 | 29.02% |
| Mel.81.Rep.1 | chr19 | 10514238 | 10514238 | C | T | 230 | 44.35% |
| Mel.81.Rep.1 | chr19 | 17970682 | 17970682 | C | T | 6174 | 3.48% |
| Mel.81.Rep.2 | chr1 | 153963227 | 153963227 | C | T | 5806 | 41.11% |
| Mel.81.Rep.2 | chr3 | 16306505 | 16306505 | C | T | 3664 | 3.93% |
| Mel.81.Rep.2 | chr7 | 140453136 | 140453136 | A | T | 3377 | 36.54% |
| Mel.81.Rep.2 | chr7 | 140453137 | 140453137 | C | T | 3375 | 36.56% |
| Mel.81.Rep.2 | chr8 | 30601668 | 30601668 | G | A | 5079 | 10.89% |
| Mel.81.Rep.2 | chr8 | 30601669 | 30601669 | G | A | 5037 | 10.82% |
| Mel.81.Rep.2 | chr12 | 7080016 | 7080016 | G | A | 3500 | 12.49% |
| Mel.81.Rep.2 | chr16 | 67694195 | 67694195 | G | A | 2068 | 7.06% |
| Mel.82.Rep.2 | chr4 | 152020703 | 152020703 | G | A | 3983 | 26.62% |
| Mel.82.Rep.2 | chr7 | 140453136 | 140453136 | A | T | 3752 | 27.77% |
| Mel.83.Rep.1 | chr7 | 39605965 | 39605965 | G | A | 516 | 16.67% |
| Mel.83.Rep.1 | chr7 | 56174180 | 56174180 | G | A | 3469 | 12.91% |
| Mel.83.Rep.1 | chr8 | 30601669 | 30601669 | G | A | 2774 | 53.68% |
| Mel.83.Rep.1 | chr19 | 17970682 | 17970682 | C | T | 4826 | 10.94% |
| Mel.83.Rep.2 | chr1 | 25559063 | 25559063 | C | T | 1974 | 42.86% |
| Mel.83.Rep.2 | chr17 | 56769918 | 56769918 | C | T | 256 | 43.14% |
| Mel.84.Rep.1 | chr1 | 153963270 | 153963270 | A | G | 5250 | 3.54% |
| Mel.84.Rep.1 | chr7 | 39605970 | 39605970 | G | A | 3139 | 7.84% |
| Mel.84.Rep.1 | chr7 | 140453136 | 140453136 | A | T | 1096 | 89.23% |
| Mel.84.Rep.1 | chr7 | 140453137 | 140453137 | C | T | 1088 | 88.88% |
| Mel.84.Rep.1 | chr12 | 53473201 | 53473201 | G | A | 463 | 12.10% |
| Mel.84.Rep.1 | chr14 | 53173817 | 53173817 | G | A | 255 | 9.02% |
| Mel.84.Rep.1 | chr14 | 53173818 | 53173818 | G | A | 254 | 9.06% |
| Mel.84.Rep.1 | chr19 | 3435234 | 3435234 | C | T | 1917 | 44.29% |
| Mel.84.Rep.2 | chr1 | 153963228 | 153963228 | C | T | 6391 | 13.83% |
| Mel.84.Rep.2 | chr7 | 140453136 | 140453136 | A | T | 659 | 24.58% |
| Mel.84.Rep.2 | chr7 | 140453137 | 140453137 | C | T | 661 | 65.81% |
| Mel.84.Rep.2 | chr9 | 131038413 | 131038413 | G | A | 2506 | 29.57% |
| Mel.84.Rep.2 | chr11 | 8704335 | 8704335 | C | T | 3237 | 41.47% |
| Mel.84.Rep.2 | chr12 | 53473201 | 53473201 | G | A | 324 | 11.11% |
| Mel.84.Rep.2 | chr14 | 53173817 | 53173817 | G | A | 626 | 18.85% |
| Mel.84.Rep.2 | chr14 | 53173818 | 53173818 | G | A | 626 | 19.49% |
| Mel.84.Rep.2 | chr16 | 2510095 | 2510095 | G | A | 4823 | 17.46% |
| Mel.84.Rep.2 | chr16 | 2510096 | 2510096 | G | A | 4745 | 17.68% |
| Mel.84.Rep.2 | chr19 | 3435234 | 3435234 | C | T | 4004 | 38.01% |
| Mel.85.Rep.1 | chr5 | 150080667 | 150080667 | C | T | 5163 | 5.56% |
| Mel.85.Rep.2 | chr1 | 153963239 | 153963239 | C | T | 5099 | 4.16% |
| Mel.85.Rep.2 | chr2 | 32390905 | 32390905 | C | T | 2706 | 98.85% |
| Mel.85.Rep.2 | chr11 | 61560113 | 61560113 | G | A | 1488 | 10.35% |
| Mel.85.Rep.2 | chr19 | 17970682 | 17970682 | C | T | 1415 | 68.06% |
| Mel.86.Rep.1 | chr1 | 25559063 | 25559063 | C | T | 5417 | 40.14% |
| Mel.86.Rep.1 | chr1 | 25559064 | 25559064 | C | T | 5421 | 41.30% |
| Mel.86.Rep.1 | chr1 | 153963239 | 153963239 | C | T | 5870 | 52.93% |
| Mel.86.Rep.1 | chr1 | 155904250 | 155904250 | C | T | 6016 | 89.10% |
| Mel.86.Rep.1 | chr2 | 10830114 | 10830114 | C | T | 3349 | 54.09% |
| Mel.86.Rep.1 | chr2 | 32390904 | 32390904 | C | T | 7581 | 36.50% |
| Mel.86.Rep.1 | chr2 | 32390905 | 32390905 | C | T | 7591 | 36.39% |
| Mel.86.Rep.1 | chr2 | 74682104 | 74682104 | G | A | 4730 | 5.05% |
| Mel.86.Rep.1 | chr2 | 74682176 | 74682176 | C | T | 4733 | 43.86% |
| Mel.86.Rep.1 | chr3 | 16306504 | 16306504 | C | T | 4734 | 59.19% |
| Mel.86.Rep.1 | chr3 | 16306505 | 16306505 | C | T | 4725 | 92.28% |
| Mel.86.Rep.1 | chr5 | 150080667 | 150080667 | C | T | 9104 | 46.73% |
| Mel.86.Rep.1 | chr7 | 56174183 | 56174183 | G | A | 5349 | 48.67% |
| Mel.86.Rep.1 | chr8 | 30601668 | 30601668 | G | A | 6409 | 86.88% |
| Mel.86.Rep.1 | chr8 | 30601669 | 30601669 | G | A | 6375 | 85.80% |
| Mel.86.Rep.1 | chr8 | 125551344 | 125551344 | C | T | 6152 | 43.46% |
| Mel.86.Rep.1 | chr8 | 125551345 | 125551345 | C | T | 6104 | 87.09% |
| Mel.86.Rep.1 | chr9 | 131038413 | 131038413 | G | A | 5320 | 38.35% |
| Mel.86.Rep.1 | chr10 | 7830002 | 7830002 | G | A | 3320 | 60.90% |
| Mel.86.Rep.1 | chr10 | 7830003 | 7830003 | G | A | 3316 | 61.22% |
| Mel.86.Rep.1 | chr10 | 127512084 | 127512084 | G | A | 3314 | 25.23% |
| Mel.86.Rep.1 | chr11 | 46958262 | 46958262 | C | T | 3937 | 84.70% |
| Mel.86.Rep.1 | chr12 | 7080016 | 7080016 | G | A | 3753 | 85.82% |
| Mel.86.Rep.1 | chr13 | 41345346 | 41345346 | C | T | 5436 | 90.81% |
| Mel.86.Rep.1 | chr16 | 2510095 | 2510095 | G | A | 3839 | 47.10% |
| Mel.86.Rep.1 | chr16 | 2510096 | 2510096 | G | A | 3824 | 36.66% |
| Mel.86.Rep.1 | chr16 | 83841526 | 83841526 | C | T | 2578 | 74.27% |
| Mel.86.Rep.1 | chr17 | 30771480 | 30771480 | C | T | 4538 | 46.27% |
| Mel.86.Rep.1 | chr17 | 56769917 | 56769917 | C | T | 2507 | 40.13% |
| Mel.86.Rep.1 | chr17 | 56769918 | 56769918 | C | A | 2513 | 34.62% |
| Mel.86.Rep.1 | chr19 | 3435234 | 3435234 | C | T | 3624 | 28.89% |
| Mel.86.Rep.1 | chr19 | 10514253 | 10514253 | G | A | 1187 | 71.27% |
| Mel.86.Rep.1 | chr19 | 10514260 | 10514260 | G | A | 1169 | 71.43% |
| Mel.86.Rep.1 | chr19 | 17970682 | 17970682 | C | T | 5689 | 54.99% |
| Mel.86.Rep.1 | chr22 | 43011001 | 43011001 | G | A | 5801 | 90.59% |
| Mel.86.Rep.2 | chr1 | 25559063 | 25559063 | C | T | 4860 | 26.75% |
| Mel.86.Rep.2 | chr1 | 25559064 | 25559064 | C | T | 4863 | 26.57% |
| Mel.86.Rep.2 | chr1 | 153963239 | 153963239 | C | T | 5125 | 47.63% |
| Mel.86.Rep.2 | chr1 | 155904250 | 155904250 | C | T | 4859 | 91.55% |
| Mel.86.Rep.2 | chr2 | 10830114 | 10830114 | C | T | 2557 | 56.20% |
| Mel.86.Rep.2 | chr2 | 32390904 | 32390904 | C | T | 5490 | 40.58% |
| Mel.86.Rep.2 | chr2 | 32390905 | 32390905 | C | T | 5459 | 40.63% |
| Mel.86.Rep.2 | chr2 | 74682176 | 74682176 | C | T | 4539 | 44.28% |
| Mel.86.Rep.2 | chr3 | 16306504 | 16306504 | C | T | 3865 | 52.56% |
| Mel.86.Rep.2 | chr3 | 16306505 | 16306505 | C | T | 3867 | 88.85% |
| Mel.86.Rep.2 | chr5 | 150080667 | 150080667 | C | T | 6969 | 49.47% |
| Mel.86.Rep.2 | chr7 | 56174183 | 56174183 | G | A | 3883 | 46.21% |
| Mel.86.Rep.2 | chr8 | 30601668 | 30601668 | G | A | 5146 | 91.27% |
| Mel.86.Rep.2 | chr8 | 30601669 | 30601669 | G | A | 5124 | 91.04% |
| Mel.86.Rep.2 | chr8 | 125551344 | 125551344 | C | T | 4545 | 55.54% |
| Mel.86.Rep.2 | chr8 | 125551345 | 125551345 | C | T | 4529 | 84.70% |
| Mel.86.Rep.2 | chr9 | 131038413 | 131038413 | G | A | 4770 | 28.70% |
| Mel.86.Rep.2 | chr10 | 7830002 | 7830002 | G | A | 2926 | 43.06% |
| Mel.86.Rep.2 | chr10 | 7830003 | 7830003 | G | A | 2916 | 43.53% |
| Mel.86.Rep.2 | chr10 | 127512084 | 127512084 | G | A | 2127 | 5.97% |
| Mel.86.Rep.2 | chr11 | 46958262 | 46958262 | C | T | 3145 | 84.38% |
| Mel.86.Rep.2 | chr12 | 7080016 | 7080016 | G | A | 3332 | 83.79% |
| Mel.86.Rep.2 | chr13 | 41345346 | 41345346 | C | T | 4531 | 89.85% |
| Mel.86.Rep.2 | chr16 | 2510095 | 2510095 | G | A | 3689 | 30.96% |
| Mel.86.Rep.2 | chr16 | 2510096 | 2510096 | G | A | 3645 | 57.50% |
| Mel.86.Rep.2 | chr16 | 83841526 | 83841526 | C | T | 2348 | 86.54% |
| Mel.86.Rep.2 | chr17 | 30771480 | 30771480 | C | T | 3909 | 45.35% |
| Mel.86.Rep.2 | chr17 | 56769917 | 56769917 | C | T | 2272 | 54.36% |
| Mel.86.Rep.2 | chr17 | 56769918 | 56769918 | C | A | 2280 | 45.44% |
| Mel.86.Rep.2 | chr19 | 3435234 | 3435234 | C | T | 2922 | 35.52% |
| Mel.86.Rep.2 | chr19 | 10514253 | 10514253 | G | A | 905 | 46.08% |
| Mel.86.Rep.2 | chr19 | 10514260 | 10514260 | G | A | 892 | 46.36% |
| Mel.86.Rep.2 | chr19 | 17970682 | 17970682 | C | T | 5383 | 25.50% |
| Mel.86.Rep.2 | chr22 | 43011001 | 43011001 | G | A | 5338 | 94.17% |
| Mel.87.Rep.1 | chr1 | 155904276 | 155904276 | C | T | 477 | 26.62% |
| Mel.87.Rep.1 | chr3 | 124449234 | 124449234 | G | A | 230 | 33.48% |
| Mel.87.Rep.1 | chr16 | 67440235 | 67440235 | G | A | 1784 | 3.59% |
| Mel.87.Rep.2 | chr7 | 140453145 | 140453145 | A | C | 187 | 82.35% |
| Mel.88.Rep.1 | chr1 | 153963239 | 153963239 | C | T | 4451 | 27.34% |
| Mel.88.Rep.1 | chr2 | 168098326 | 168098326 | C | T | 5964 | 3.30% |
| Mel.88.Rep.1 | chr7 | 56174183 | 56174183 | G | A | 3671 | 30.73% |
| Mel.88.Rep.1 | chr8 | 125551344 | 125551344 | C | T | 3977 | 7.37% |
| Mel.88.Rep.1 | chr8 | 125551345 | 125551345 | C | T | 3994 | 7.31% |
| Mel.88.Rep.1 | chr13 | 41345346 | 41345346 | C | T | 4024 | 16.93% |
| Mel.88.Rep.1 | chr16 | 67694228 | 67694228 | G | A | 1792 | 60.88% |
| Mel.88.Rep.1 | chr19 | 4247060 | 4247060 | G | A | 2172 | 26.47% |
| Mel.88.Rep.1 | chr19 | 10514253 | 10514253 | G | A | 318 | 10.38% |
| Mel.88.Rep.1 | chr19 | 17970682 | 17970682 | C | T | 4366 | 13.57% |
| Mel.88.Rep.1 | chr19 | 38202425 | 38202425 | G | A | 8796 | 9.37% |
| Mel.88.Rep.1 | chr20 | 34129795 | 34129795 | G | A | 2673 | 13.73% |
| Mel.88.Rep.2 | chr1 | 153963239 | 153963239 | C | T | 7973 | 23.58% |
| Mel.88.Rep.2 | chr1 | 153963254 | 153963254 | G | A | 7955 | 4.17% |
| Mel.88.Rep.2 | chr7 | 56174183 | 56174183 | G | A | 5729 | 36.85% |
| Mel.88.Rep.2 | chr8 | 125551344 | 125551344 | C | T | 6172 | 18.31% |
| Mel.88.Rep.2 | chr8 | 125551345 | 125551345 | C | T | 6175 | 18.41% |
| Mel.88.Rep.2 | chr13 | 41345346 | 41345346 | C | T | 6886 | 13.13% |
| Mel.88.Rep.2 | chr16 | 2510095 | 2510095 | G | A | 4665 | 34.58% |
| Mel.88.Rep.2 | chr16 | 67694228 | 67694228 | G | A | 2281 | 17.10% |
| Mel.88.Rep.2 | chr19 | 4247060 | 4247060 | G | A | 3482 | 29.91% |
| Mel.88.Rep.2 | chr19 | 15768976 | 15768976 | C | T | 4276 | 10.36% |
| Mel.88.Rep.2 | chr19 | 17970682 | 17970682 | C | T | 7834 | 30.88% |
| Mel.88.Rep.2 | chr19 | 38202425 | 38202425 | G | A | 16155 | 5.50% |
| Mel.88.Rep.2 | chr19 | 48248737 | 48248737 | G | A | 5720 | 6.73% |
| Mel.88.Rep.2 | chr20 | 34129795 | 34129795 | G | A | 4611 | 10.74% |
| Mel.89.Rep.1 | chr8 | 125551345 | 125551345 | C | T | 4251 | 51.41% |
| Mel.89.Rep.1 | chr11 | 8704334 | 8704334 | C | T | 884 | 3.05% |
| Mel.89.Rep.1 | chr12 | 53473219 | 53473219 | C | T | 2649 | 3.10% |
| Mel.89.Rep.1 | chr16 | 2510095 | 2510095 | G | A | 2524 | 48.65% |
| Mel.89.Rep.1 | chr19 | 48248748 | 48248748 | G | A | 2679 | 72.38% |
| Mel.89.Rep.2 | chr1 | 115256529 | 115256529 | T | C | 6465 | 41.39% |
| Mel.89.Rep.2 | chr3 | 16306505 | 16306505 | C | T | 8854 | 4.57% |
| Mel.89.Rep.2 | chr5 | 150080667 | 150080667 | C | T | 8083 | 18.27% |
| Mel.89.Rep.2 | chr8 | 125551345 | 125551345 | C | T | 3289 | 13.68% |
| Mel.89.Rep.2 | chr16 | 2510095 | 2510095 | G | A | 6365 | 34.32% |
| Mel.89.Rep.2 | chr16 | 67694211 | 67694211 | C | T | 4664 | 9.11% |
| Mel.89.Rep.2 | chr19 | 4247060 | 4247060 | G | A | 2691 | 4.20% |
| Mel.89.Rep.2 | chr19 | 17970682 | 17970682 | C | T | 7798 | 41.87% |
| Mel.89.Rep.2 | chr19 | 48248748 | 48248748 | G | A | 4507 | 41.74% |
| Mel.90.Rep.1 | chr3 | 124449234 | 124449234 | G | A | 2625 | 29.66% |
| Mel.90.Rep.1 | chr5 | 150080670 | 150080670 | C | T | 2995 | 41.07% |
| Mel.90.Rep.1 | chr7 | 53103782 | 53103782 | G | A | 1952 | 4.35% |
| Mel.90.Rep.1 | chr7 | 140453136 | 140453136 | A | T | 540 | 10.93% |
| Mel.90.Rep.1 | chr19 | 17970682 | 17970682 | C | T | 1670 | 24.51% |
| Mel.90.Rep.2 | chr5 | 150080670 | 150080670 | C | T | 4042 | 62.84% |
| Mel.90.Rep.2 | chr19 | 17970682 | 17970682 | C | T | 1307 | 58.45% |
| Mel.91.Rep.1 | chr4 | 152020713 | 152020713 | G | A | 10014 | 20.16% |
| Mel.91.Rep.1 | chr7 | 140453136 | 140453136 | A | T | 3262 | 28.11% |
| Mel.91.Rep.1 | chr14 | 53173818 | 53173818 | G | A | 581 | 10.67% |
| Mel.91.Rep.2 | chr3 | 52542219 | 52542219 | G | A | 781 | 6.91% |
| Mel.91.Rep.2 | chr7 | 140453136 | 140453136 | A | T | 2869 | 23.14% |
| Mel.92.Rep.1 | chr1 | 155904276 | 155904276 | C | T | 5025 | 7.26% |
| Mel.92.Rep.1 | chr1 | 231114784 | 231114784 | C | T | 985 | 30.76% |
| Mel.92.Rep.1 | chr2 | 32390904 | 32390904 | C | T | 2431 | 30.32% |
| Mel.92.Rep.1 | chr7 | 140453136 | 140453136 | A | T | 3919 | 35.72% |
| Mel.92.Rep.1 | chr7 | 140453137 | 140453137 | C | T | 3906 | 35.56% |
| Mel.92.Rep.1 | chr11 | 8704277 | 8704277 | G | A | 2010 | 3.68% |
| Mel.92.Rep.1 | chr14 | 53173817 | 53173817 | G | A | 607 | 38.71% |
| Mel.92.Rep.1 | chr16 | 2510095 | 2510095 | G | A | 4906 | 33.10% |
| Mel.92.Rep.1 | chr19 | 50169131 | 50169131 | C | T | 1788 | 31.10% |
| Mel.92.Rep.1 | chr20 | 34129830 | 34129830 | C | T | 1546 | 4.66% |
| Mel.92.Rep.2 | chr1 | 231114784 | 231114784 | C | T | 975 | 4% |
| Mel.92.Rep.2 | chr2 | 32390904 | 32390904 | C | T | 2675 | 30.02% |
| Mel.92.Rep.2 | chr4 | 152020703 | 152020703 | G | A | 6856 | 5.92% |
| Mel.92.Rep.2 | chr7 | 140453136 | 140453136 | A | T | 3871 | 34.75% |
| Mel.92.Rep.2 | chr7 | 140453137 | 140453137 | C | T | 3868 | 34.77% |
| Mel.92.Rep.2 | chr11 | 8704277 | 8704277 | G | A | 2263 | 10.91% |
| Mel.92.Rep.2 | chr14 | 53173817 | 53173817 | G | A | 476 | 5.46% |
| Mel.92.Rep.2 | chr19 | 10514239 | 10514239 | C | T | 309 | 71.75% |
| Mel.93.Rep.1 | chr1 | 155904250 | 155904250 | C | T | 5705 | 10.13% |
| Mel.93.Rep.1 | chr2 | 32390904 | 32390904 | C | T | 3028 | 4.52% |
| Mel.93.Rep.1 | chr5 | 150080667 | 150080667 | C | T | 3645 | 5.08% |
| Mel.93.Rep.1 | chr6 | 30640795 | 30640795 | G | A | 7216 | 4.46% |
| Mel.93.Rep.1 | chr7 | 140453136 | 140453136 | A | T | 4192 | 20.75% |
| Mel.93.Rep.1 | chr11 | 46958261 | 46958261 | C | T | 4692 | 5.14% |
| Mel.93.Rep.1 | chr11 | 61560107 | 61560107 | G | A | 4427 | 3.32% |
| Mel.93.Rep.1 | chr16 | 2510096 | 2510096 | G | A | 6270 | 21.23% |
| Mel.93.Rep.1 | chr17 | 54872420 | 54872420 | T | C | 2006 | 5.13% |
| Mel.93.Rep.2 | chr1 | 155904250 | 155904250 | C | T | 4400 | 8.73% |
| Mel.93.Rep.2 | chr4 | 152020703 | 152020703 | G | A | 6624 | 4.51% |
| Mel.93.Rep.2 | chr7 | 140453136 | 140453136 | A | T | 3391 | 28.93% |
| Mel.93.Rep.2 | chr9 | 131038413 | 131038413 | G | A | 4492 | 4.23% |
| Mel.93.Rep.2 | chr9 | 131038414 | 131038414 | G | A | 4445 | 4.23% |
| Mel.93.Rep.2 | chr16 | 2510096 | 2510096 | G | A | 4892 | 19.69% |
| Mel.93.Rep.2 | chr16 | 67694211 | 67694211 | C | T | 2777 | 7.42% |
| Mel.94.Rep.1 | chr3 | 16306505 | 16306505 | C | T | 3474 | 62.80% |
| Mel.94.Rep.1 | chr7 | 140453136 | 140453136 | A | T | 2859 | 39.66% |
| Mel.94.Rep.1 | chr16 | 2510096 | 2510096 | G | A | 3559 | 20.06% |
| Mel.94.Rep.2 | chr3 | 16306505 | 16306505 | C | T | 4432 | 62.59% |
| Mel.94.Rep.2 | chr7 | 140453136 | 140453136 | A | T | 3471 | 43.85% |
| Mel.94.Rep.2 | chr16 | 2510096 | 2510096 | G | A | 3874 | 55.76% |
| Mel.94.Rep.2 | chr16 | 67694211 | 67694211 | C | A | 1665 | 10.75% |
| Mel.96.Rep.1 | chr1 | 153963227 | 153963227 | C | T | 4366 | 34.91% |
| Mel.96.Rep.1 | chr2 | 32390904 | 32390904 | C | T | 2384 | 4.40% |
| Mel.96.Rep.1 | chr2 | 74682104 | 74682104 | G | A | 4416 | 6.25% |
| Mel.96.Rep.1 | chr3 | 16306504 | 16306504 | C | T | 5142 | 42.45% |
| Mel.96.Rep.1 | chr3 | 16306505 | 16306505 | C | T | 5135 | 59.82% |
| Mel.96.Rep.1 | chr4 | 152020703 | 152020703 | G | A | 7223 | 8.57% |
| Mel.96.Rep.1 | chr7 | 39605969 | 39605969 | G | A | 3111 | 80.41% |
| Mel.96.Rep.1 | chr7 | 39605970 | 39605970 | G | A | 3117 | 80.62% |
| Mel.96.Rep.1 | chr7 | 140453136 | 140453136 | A | T | 3068 | 63.23% |
| Mel.96.Rep.1 | chr11 | 8704256 | 8704256 | G | A | 1087 | 17.39% |
| Mel.96.Rep.1 | chr12 | 117923349 | 117923349 | G | A | 2005 | 22.22% |
| Mel.96.Rep.1 | chr14 | 53173817 | 53173817 | G | A | 467 | 17.13% |
| Mel.96.Rep.1 | chr16 | 29802047 | 29802047 | C | T | 1303 | 13.35% |
| Mel.96.Rep.1 | chr16 | 83841526 | 83841526 | C | T | 986 | 46.25% |
| Mel.96.Rep.1 | chr16 | 83841529 | 83841529 | C | T | 989 | 46.11% |
| Mel.96.Rep.1 | chr19 | 4247041 | 4247041 | G | A | 3828 | 10.95% |
| Mel.96.Rep.1 | chr19 | 10514239 | 10514239 | C | T | 1643 | 75.46% |
| Mel.96.Rep.1 | chr19 | 13885240 | 13885240 | C | A | 4213 | 4.63% |
| Mel.96.Rep.1 | chr19 | 54197615 | 54197615 | G | A | 2237 | 10.65% |
| Mel.96.Rep.2 | chr1 | 153963227 | 153963227 | C | T | 3225 | 30.45% |
| Mel.96.Rep.2 | chr3 | 16306504 | 16306504 | C | T | 5127 | 38.02% |
| Mel.96.Rep.2 | chr3 | 16306505 | 16306505 | C | T | 5117 | 69.81% |
| Mel.96.Rep.2 | chr5 | 150080667 | 150080667 | C | T | 2532 | 8.45% |
| Mel.96.Rep.2 | chr7 | 39605969 | 39605969 | G | A | 3655 | 70.90% |
| Mel.96.Rep.2 | chr7 | 39605970 | 39605970 | G | A | 3670 | 71.09% |
| Mel.96.Rep.2 | chr7 | 140453136 | 140453136 | A | T | 2444 | 44.64% |
| Mel.96.Rep.2 | chr8 | 125551345 | 125551345 | C | T | 2476 | 38.09% |
| Mel.96.Rep.2 | chr11 | 8704256 | 8704256 | G | A | 412 | 55.10% |
| Mel.96.Rep.2 | chr11 | 46958262 | 46958262 | C | T | 3487 | 7.58% |
| Mel.96.Rep.2 | chr12 | 7080017 | 7080017 | G | A | 3978 | 5.81% |
| Mel.96.Rep.2 | chr12 | 53473217 | 53473217 | C | T | 3028 | 4.76% |
| Mel.96.Rep.2 | chr12 | 117923349 | 117923349 | G | A | 1577 | 41.27% |
| Mel.96.Rep.2 | chr13 | 41345306 | 41345306 | G | A | 3025 | 4.53% |
| Mel.96.Rep.2 | chr14 | 53173817 | 53173817 | G | A | 457 | 36.76% |
| Mel.96.Rep.2 | chr16 | 83841526 | 83841526 | C | T | 1299 | 27.64% |
| Mel.96.Rep.2 | chr16 | 83841529 | 83841529 | C | T | 1292 | 27.71% |
| Mel.96.Rep.2 | chr19 | 4247041 | 4247041 | G | A | 3393 | 4.10% |
| Mel.96.Rep.2 | chr19 | 4247072 | 4247072 | C | T | 3336 | 8.84% |
| Mel.96.Rep.2 | chr19 | 10514239 | 10514239 | C | T | 966 | 66.53% |
| Mel.96.Rep.2 | chr19 | 54197615 | 54197615 | G | A | 1009 | 58.98% |
| Mel.97.Rep.1 | chr1 | 155904250 | 155904250 | C | T | 5444 | 49.90% |
| Mel.97.Rep.1 | chr2 | 10830118 | 10830118 | G | A | 7112 | 4.99% |
| Mel.97.Rep.1 | chr3 | 16306504 | 16306504 | C | T | 5395 | 13.14% |
| Mel.97.Rep.1 | chr3 | 48481603 | 48481603 | C | T | 4376 | 23.35% |
| Mel.97.Rep.1 | chr4 | 152020703 | 152020703 | G | A | 8277 | 4.69% |
| Mel.97.Rep.1 | chr4 | 152020713 | 152020713 | G | A | 8264 | 23.91% |
| Mel.97.Rep.1 | chr5 | 150080667 | 150080667 | C | T | 3812 | 3.78% |
| Mel.97.Rep.1 | chr8 | 30601669 | 30601669 | G | A | 5335 | 26.35% |
| Mel.97.Rep.1 | chr9 | 131038413 | 131038413 | G | A | 4937 | 36.93% |
| Mel.97.Rep.1 | chr11 | 65063342 | 65063342 | C | T | 1180 | 9.49% |
| Mel.97.Rep.1 | chr16 | 2510095 | 2510095 | G | A | 5017 | 15.85% |
| Mel.97.Rep.2 | chr1 | 155904250 | 155904250 | C | T | 3782 | 58.19% |
| Mel.97.Rep.2 | chr3 | 48481603 | 48481603 | C | T | 3894 | 22.52% |
| Mel.97.Rep.2 | chr4 | 152020713 | 152020713 | G | A | 6484 | 39.99% |
| Mel.98.Rep.1 | chr2 | 32390904 | 32390904 | C | G | 4860 | 3.31% |
| Mel.98.Rep.1 | chr2 | 32390905 | 32390905 | C | T | 4875 | 3.26% |
| Mel.98.Rep.1 | chr4 | 152020703 | 152020703 | G | A | 7545 | 11.68% |
| Mel.98.Rep.1 | chr5 | 150080667 | 150080667 | C | T | 5605 | 19.40% |
| Mel.98.Rep.1 | chr7 | 140453136 | 140453136 | A | T | 5593 | 9.24% |
| Mel.98.Rep.1 | chr20 | 34129795 | 34129795 | G | A | 4154 | 9.37% |
| Mel.98.Rep.2 | chr7 | 140453136 | 140453136 | A | T | 5572 | 4.02% |
| Mel.98.Rep.2 | chr19 | 10514232 | 10514232 | C | T | 815 | 56.07% |
| Mel.98.Rep.2 | chr19 | 13885240 | 13885240 | C | T | 4788 | 6.39% |
| Mel.98.Rep.2 | chr19 | 13885241 | 13885241 | C | T | 4811 | 6.34% |
| Mel.99.Rep.1 | chr5 | 145826780 | 145826780 | C | T | 226 | 84.07% |
| Mel.99.Rep.1 | chr5 | 150080667 | 150080667 | C | T | 260 | 56.54% |
| Mel.99.Rep.1 | chr16 | 2510096 | 2510096 | G | A | 483 | 65.63% |
| Mel.99.Rep.2 | chr1 | 55181529 | 55181529 | G | A | 2082 | 68.49% |
| Mel.100.Rep.1 | chr2 | 32390905 | 32390905 | C | T | 2094 | 5.59% |
| Mel.100.Rep.1 | chr2 | 105954000 | 105954000 | C | T | 3759 | 15.89% |
| Mel.100.Rep.1 | chr3 | 16306504 | 16306504 | C | T | 4098 | 26.57% |
| Mel.100.Rep.1 | chr5 | 150080667 | 150080667 | C | T | 2404 | 5.16% |
| Mel.100.Rep.1 | chr8 | 30601669 | 30601669 | G | A | 3549 | 21.56% |
| Mel.100.Rep.1 | chr11 | 8704338 | 8704338 | C | T | 1364 | 17.67% |
| Mel.100.Rep.1 | chr11 | 46958261 | 46958261 | C | T | 3479 | 5.32% |
| Mel.100.Rep.1 | chr11 | 46958262 | 46958262 | C | T | 3476 | 5.45% |
| Mel.100.Rep.1 | chr16 | 2510095 | 2510095 | G | A | 3498 | 10.75% |
| Mel.100.Rep.1 | chr19 | 10514238 | 10514238 | C | T | 875 | 51.43% |
| Mel.100.Rep.2 | chr2 | 105954000 | 105954000 | C | T | 3551 | 4.76% |
| Mel.100.Rep.2 | chr3 | 16306504 | 16306504 | C | T | 3971 | 8.51% |
| Mel.100.Rep.2 | chr5 | 145826780 | 145826780 | C | T | 2269 | 31.69% |
| Mel.100.Rep.2 | chr5 | 150080667 | 150080667 | C | T | 2853 | 4.94% |
| Mel.100.Rep.2 | chr8 | 30601669 | 30601669 | G | A | 4491 | 21.53% |
| Mel.100.Rep.2 | chr11 | 8704338 | 8704338 | C | T | 1242 | 26.17% |
| Mel.100.Rep.2 | chr11 | 46958261 | 46958261 | C | T | 2990 | 36.78% |
| Mel.100.Rep.2 | chr11 | 46958262 | 46958262 | C | T | 2972 | 36.96% |
| Mel.100.Rep.2 | chr14 | 53173818 | 53173818 | G | A | 289 | 13.49% |
| Mel.100.Rep.2 | chr19 | 10514238 | 10514238 | C | T | 906 | 26.16% |
| Mel.100.Rep.2 | chr19 | 48248748 | 48248748 | G | A | 3322 | 32.28% |
| Mel.101.Rep.1 | chr1 | 894683 | 894683 | C | T | 218 | 85.32% |
| Mel.101.Rep.1 | chr1 | 25559063 | 25559063 | C | T | 5790 | 29.14% |
| Mel.101.Rep.1 | chr1 | 25559064 | 25559064 | C | T | 5816 | 15.36% |
| Mel.101.Rep.1 | chr1 | 153963181 | 153963181 | C | G | 3813 | 4.41% |
| Mel.101.Rep.1 | chr2 | 32390904 | 32390904 | C | T | 2326 | 3.44% |
| Mel.101.Rep.1 | chr2 | 32390905 | 32390905 | C | T | 2353 | 3.27% |
| Mel.101.Rep.1 | chr2 | 74682104 | 74682104 | G | A | 4436 | 14.74% |
| Mel.101.Rep.1 | chr2 | 74682140 | 74682140 | G | T | 4431 | 7.88% |
| Mel.101.Rep.1 | chr3 | 16306504 | 16306504 | C | T | 4453 | 22.30% |
| Mel.101.Rep.1 | chr3 | 16306505 | 16306505 | C | T | 4457 | 49.73% |
| Mel.101.Rep.1 | chr5 | 150080667 | 150080667 | C | T | 3074 | 36.61% |
| Mel.101.Rep.1 | chr7 | 140453136 | 140453136 | A | T | 3023 | 10.92% |
| Mel.101.Rep.1 | chr9 | 130700157 | 130700157 | C | T | 2387 | 6.83% |
| Mel.101.Rep.1 | chr16 | 2510096 | 2510096 | G | T | 4266 | 25.69% |
| Mel.101.Rep.1 | chr19 | 3435234 | 3435234 | C | T | 2654 | 21.33% |
| Mel.101.Rep.1 | chr19 | 4247060 | 4247060 | G | A | 3591 | 22.28% |
| Mel.101.Rep.1 | chr19 | 15768976 | 15768976 | C | T | 5317 | 25.45% |
| Mel.101.Rep.1 | chr19 | 17970682 | 17970682 | C | T | 2290 | 21.22% |
| Mel.101.Rep.1 | chr20 | 34129792 | 34129792 | G | A | 1172 | 45.90% |
| Mel.101.Rep.1 | chr20 | 34129830 | 34129830 | C | T | 1179 | 15.79% |
| Mel.101.Rep.2 | chr1 | 25559063 | 25559063 | C | T | 5252 | 18.79% |
| Mel.101.Rep.2 | chr2 | 74682104 | 74682104 | G | A | 3332 | 4.35% |
| Mel.101.Rep.2 | chr3 | 16306504 | 16306504 | C | T | 3974 | 26.32% |
| Mel.101.Rep.2 | chr3 | 16306505 | 16306505 | C | T | 3982 | 43.55% |
| Mel.101.Rep.2 | chr4 | 152020703 | 152020703 | G | A | 5493 | 10.96% |
| Mel.101.Rep.2 | chr5 | 150080667 | 150080667 | C | T | 2975 | 28.94% |
| Mel.101.Rep.2 | chr7 | 140453136 | 140453136 | A | T | 2652 | 8.48% |
| Mel.101.Rep.2 | chr10 | 7830002 | 7830002 | G | A | 3021 | 27.41% |
| Mel.101.Rep.2 | chr14 | 53173817 | 53173817 | G | A | 504 | 6.35% |
| Mel.101.Rep.2 | chr16 | 2510095 | 2510095 | G | A | 3882 | 7.65% |
| Mel.101.Rep.2 | chr19 | 3435234 | 3435234 | C | T | 2485 | 31.63% |
| Mel.101.Rep.2 | chr19 | 4247060 | 4247060 | G | A | 3575 | 10.04% |
| Mel.101.Rep.2 | chr19 | 10514238 | 10514238 | C | T | 315 | 26.98% |
| Mel.101.Rep.2 | chr19 | 10514239 | 10514239 | C | T | 317 | 27.13% |
| Mel.101.Rep.2 | chr19 | 15768976 | 15768976 | C | T | 4307 | 24.13% |
| Mel.101.Rep.2 | chr19 | 17970682 | 17970682 | C | T | 1521 | 21.99% |
| Mel.101.Rep.2 | chr20 | 34129792 | 34129792 | G | A | 996 | 41.57% |
| Mel.101.Rep.2 | chr20 | 34129830 | 34129830 | C | T | 995 | 14.87% |
| Mel.102.Rep.1 | chr2 | 168098327 | 168098327 | G | A | 2331 | 52.21% |
| Mel.102.Rep.1 | chr7 | 53103783 | 53103783 | G | A | 1112 | 14.57% |
| Mel.102.Rep.1 | chr7 | 140453136 | 140453136 | A | T | 2350 | 17.15% |
| Mel.102.Rep.1 | chr8 | 68931944 | 68931944 | C | T | 2212 | 30.61% |
| Mel.102.Rep.1 | chr19 | 3435234 | 3435234 | C | T | 2201 | 9.13% |
| Mel.102.Rep.1 | chr19 | 17970723 | 17970723 | G | C | 1689 | 72.65% |
| Mel.102.Rep.1 | chr19 | 48248737 | 48248737 | G | A | 1861 | 41.88% |
| Mel.102.Rep.2 | chr1 | 153963227 | 153963227 | C | T | 3677 | 11.42% |
| Mel.102.Rep.2 | chr7 | 56174204 | 56174204 | C | T | 2832 | 21.54% |
| Mel.102.Rep.2 | chr7 | 140453136 | 140453136 | A | T | 2666 | 20.86% |
| Mel.102.Rep.2 | chr8 | 68931945 | 68931946 | CC | C | 2763 | 24.68% |
| Mel.102.Rep.2 | chr13 | 41345350 | 41345350 | C | T | 2637 | 11.83% |
| Mel.102.Rep.2 | chr19 | 3435234 | 3435234 | C | T | 2326 | 7.78% |
| Mel.102.Rep.2 | chr19 | 17970723 | 17970723 | G | C | 1532 | 14.03% |
| Mel.102.Rep.2 | chr19 | 48248737 | 48248737 | G | A | 2013 | 34.82% |
| Mel.103.Rep.1 | chr3 | 48481602 | 48481602 | C | T | 1755 | 50.31% |
| Mel.105.Rep.1 | chr1 | 231114784 | 231114784 | C | T | 795 | 4.53% |
| Mel.105.Rep.1 | chr11 | 8704335 | 8704335 | C | T | 1592 | 20.16% |
| Mel.105.Rep.2 | chr19 | 38202422 | 38202422 | G | A | 5775 | 14.96% |
| Mel.106.Rep.1 | chr3 | 16306504 | 16306504 | C | T | 4128 | 5.94% |
| Mel.106.Rep.1 | chr3 | 16306505 | 16306505 | C | T | 4132 | 5.81% |
| Mel.106.Rep.1 | chr5 | 150080667 | 150080667 | C | T | 2562 | 12.10% |
| Mel.106.Rep.1 | chr11 | 46958261 | 46958261 | C | T | 3145 | 15.59% |
| Mel.106.Rep.1 | chr11 | 46958262 | 46958262 | C | T | 3150 | 15.70% |
| Mel.106.Rep.1 | chr16 | 67440235 | 67440235 | G | A | 4268 | 11.25% |
| Mel.106.Rep.1 | chr19 | 4247045 | 4247045 | G | A | 2052 | 8.92% |
| Mel.106.Rep.2 | chr1 | 153963222 | 153963222 | G | A | 8008 | 12.46% |
| Mel.106.Rep.2 | chr10 | 127512083 | 127512083 | G | A | 6142 | 24.26% |
| Mel.106.Rep.2 | chr11 | 46958261 | 46958261 | C | T | 7516 | 13.53% |
| Mel.106.Rep.2 | chr11 | 46958262 | 46958262 | C | T | 7492 | 13.65% |
| Mel.106.Rep.2 | chr13 | 41345355 | 41345355 | C | A | 5556 | 3.37% |
| Mel.106.Rep.2 | chr19 | 4247045 | 4247045 | G | A | 6532 | 14.94% |
| Mel.107.Rep.1 | chr1 | 153963239 | 153963239 | C | T | 3463 | 89.19% |
| Mel.107.Rep.1 | chr1 | 155904250 | 155904250 | C | T | 2674 | 93.87% |
| Mel.107.Rep.1 | chr2 | 10830114 | 10830114 | C | T | 4804 | 14.63% |
| Mel.107.Rep.1 | chr2 | 32390904 | 32390904 | C | T | 1951 | 25.42% |
| Mel.107.Rep.1 | chr7 | 39605969 | 39605969 | G | A | 2967 | 13.45% |
| Mel.107.Rep.1 | chr9 | 131038409 | 131038409 | G | A | 2721 | 70.38% |
| Mel.107.Rep.1 | chr10 | 7830003 | 7830003 | G | A | 1680 | 62.74% |
| Mel.107.Rep.1 | chr11 | 65063343 | 65063343 | C | T | 380 | 15.53% |
| Mel.107.Rep.1 | chr11 | 99932099 | 99932099 | C | T | 5704 | 85.79% |
| Mel.107.Rep.1 | chr16 | 2510095 | 2510095 | G | A | 2222 | 53.76% |
| Mel.107.Rep.1 | chr16 | 67694198 | 67694198 | G | T | 2156 | 55.61% |
| Mel.107.Rep.1 | chr19 | 10514239 | 10514239 | C | T | 663 | 100% |
| Mel.107.Rep.1 | chr19 | 10514260 | 10514260 | G | A | 662 | 99.70% |
| Mel.107.Rep.1 | chr22 | 43011001 | 43011001 | G | A | 2065 | 29.15% |
| Mel.107.Rep.2 | chr1 | 153963227 | 153963227 | C | T | 5482 | 6.07% |
| Mel.107.Rep.2 | chr1 | 153963239 | 153963239 | C | T | 5463 | 73% |
| Mel.107.Rep.2 | chr1 | 155904250 | 155904250 | C | T | 4858 | 93.47% |
| Mel.107.Rep.2 | chr4 | 152020713 | 152020713 | G | A | 8701 | 10.08% |
| Mel.107.Rep.2 | chr7 | 39605969 | 39605969 | G | A | 3251 | 28.11% |
| Mel.107.Rep.2 | chr9 | 130700157 | 130700157 | C | T | 1664 | 32.15% |
| Mel.107.Rep.2 | chr9 | 131038409 | 131038409 | G | A | 3491 | 82.18% |
| Mel.107.Rep.2 | chr10 | 7830003 | 7830003 | G | A | 1656 | 16.30% |
| Mel.107.Rep.2 | chr11 | 99932099 | 99932099 | C | T | 9544 | 97.16% |
| Mel.107.Rep.2 | chr12 | 7080016 | 7080016 | G | C | 5317 | 3.76% |
| Mel.107.Rep.2 | chr14 | 53173817 | 53173817 | G | A | 586 | 29.52% |
| Mel.107.Rep.2 | chr14 | 53173818 | 53173818 | G | A | 585 | 29.23% |
| Mel.107.Rep.2 | chr16 | 2510095 | 2510095 | G | A | 3103 | 66.15% |
| Mel.107.Rep.2 | chr16 | 2510096 | 2510096 | G | A | 3108 | 32.38% |
| Mel.107.Rep.2 | chr16 | 67694198 | 67694198 | G | A | 3349 | 57.28% |
| Mel.107.Rep.2 | chr19 | 10514239 | 10514239 | C | T | 1022 | 42.66% |
| Mel.107.Rep.2 | chr19 | 10514260 | 10514260 | G | A | 1013 | 43.44% |
| Mel.107.Rep.2 | chr20 | 34129792 | 34129792 | G | A | 1113 | 37.20% |
| Mel.107.Rep.2 | chr22 | 43011001 | 43011001 | G | A | 2553 | 13.36% |
| Mel.108.Rep.1 | chr4 | 53611041 | 53611041 | C | T | 4170 | 14.51% |
| Mel.108.Rep.1 | chr4 | 152020703 | 152020703 | G | A | 4801 | 15.25% |
| Mel.108.Rep.1 | chr12 | 7080017 | 7080017 | G | A | 2858 | 12.49% |
| Mel.108.Rep.1 | chr12 | 117923349 | 117923349 | G | A | 2258 | 43% |
| Mel.108.Rep.1 | chr13 | 41345346 | 41345346 | C | T | 1986 | 28.60% |
| Mel.108.Rep.1 | chr14 | 53173817 | 53173817 | G | A | 388 | 20.36% |
| Mel.108.Rep.1 | chr19 | 3435235 | 3435235 | C | T | 2482 | 6.61% |
| Mel.108.Rep.1 | chr19 | 13885241 | 13885241 | C | T | 2889 | 22.34% |
| Mel.108.Rep.1 | chr20 | 34129795 | 34129795 | G | A | 810 | 11.12% |
| Mel.108.Rep.2 | chr4 | 152020703 | 152020703 | G | A | 8245 | 14% |
| Mel.108.Rep.2 | chr8 | 30601668 | 30601668 | G | A | 5205 | 6.72% |
| Mel.108.Rep.2 | chr8 | 30601669 | 30601669 | G | A | 5184 | 6.77% |
| Mel.108.Rep.2 | chr11 | 8704256 | 8704256 | G | A | 1375 | 12.95% |
| Mel.108.Rep.2 | chr12 | 7080017 | 7080017 | G | A | 4767 | 21.40% |
| Mel.108.Rep.2 | chr12 | 117923349 | 117923349 | G | A | 2848 | 19.03% |
| Mel.108.Rep.2 | chr13 | 41345346 | 41345346 | C | T | 3301 | 27.24% |
| Mel.108.Rep.2 | chr14 | 53173817 | 53173817 | G | A | 425 | 11.06% |
| Mel.108.Rep.2 | chr19 | 13885241 | 13885241 | C | T | 4588 | 7.24% |
| Mel.108.Rep.2 | chr19 | 48248748 | 48248748 | G | A | 4279 | 5.21% |
| Mel.108.Rep.2 | chr20 | 34129795 | 34129795 | G | A | 1784 | 11.77% |
| Mel.109.Rep.1 | chr1 | 25559063 | 25559063 | C | T | 5538 | 15.93% |
| Mel.109.Rep.1 | chr1 | 55181528 | 55181528 | G | A | 5543 | 48.39% |
| Mel.109.Rep.1 | chr1 | 153963222 | 153963222 | G | A | 5374 | 38.07% |
| Mel.109.Rep.1 | chr1 | 155904250 | 155904250 | C | T | 5196 | 52.87% |
| Mel.109.Rep.1 | chr2 | 32390905 | 32390905 | C | T | 2578 | 56.72% |
| Mel.109.Rep.1 | chr2 | 74682140 | 74682140 | G | A | 4532 | 32.66% |
| Mel.109.Rep.1 | chr3 | 67048644 | 67048644 | C | T | 5325 | 46.22% |
| Mel.109.Rep.1 | chr5 | 145826781 | 145826781 | G | A | 254 | 35.04% |
| Mel.109.Rep.1 | chr5 | 150080667 | 150080667 | C | T | 3511 | 71.14% |
| Mel.109.Rep.1 | chr6 | 30640796 | 30640796 | G | A | 2380 | 28.78% |
| Mel.109.Rep.1 | chr7 | 56174183 | 56174183 | G | A | 4083 | 44.51% |
| Mel.109.Rep.1 | chr7 | 140453136 | 140453136 | A | T | 2203 | 36.77% |
| Mel.109.Rep.1 | chr8 | 30601668 | 30601668 | G | A | 4517 | 28.69% |
| Mel.109.Rep.1 | chr8 | 30601669 | 30601669 | G | A | 4488 | 45.81% |
| Mel.109.Rep.1 | chr9 | 130700157 | 130700157 | C | T | 735 | 22.99% |
| Mel.109.Rep.1 | chr9 | 131038409 | 131038409 | G | A | 3375 | 21.91% |
| Mel.109.Rep.1 | chr9 | 131038413 | 131038413 | G | A | 3372 | 10.14% |
| Mel.109.Rep.1 | chr11 | 8704277 | 8704277 | G | A | 4114 | 18.42% |
| Mel.109.Rep.1 | chr11 | 61560113 | 61560113 | G | A | 2740 | 57.30% |
| Mel.109.Rep.1 | chr13 | 41345346 | 41345346 | C | T | 3586 | 53.96% |
| Mel.109.Rep.1 | chr14 | 53173818 | 53173818 | G | A | 522 | 37.55% |
| Mel.109.Rep.1 | chr16 | 2510095 | 2510095 | G | A | 3612 | 71.07% |
| Mel.109.Rep.1 | chr16 | 2510096 | 2510096 | G | A | 3615 | 51.37% |
| Mel.109.Rep.1 | chr17 | 30771480 | 30771480 | C | T | 2594 | 3.12% |
| Mel.109.Rep.1 | chr17 | 56769918 | 56769918 | C | T | 1748 | 19.39% |
| Mel.109.Rep.1 | chr20 | 34129795 | 34129795 | G | A | 2445 | 3.80% |
| Mel.109.Rep.1 | chr22 | 43010992 | 43010992 | G | A | 3359 | 37.76% |
| Mel.109.Rep.2 | chr1 | 25559063 | 25559063 | C | T | 7152 | 16.41% |
| Mel.109.Rep.2 | chr1 | 55181528 | 55181528 | G | A | 6509 | 12.06% |
| Mel.109.Rep.2 | chr1 | 153963222 | 153963222 | G | A | 6430 | 31.73% |
| Mel.109.Rep.2 | chr1 | 155904250 | 155904250 | C | T | 6777 | 28.82% |
| Mel.109.Rep.2 | chr2 | 32390905 | 32390905 | C | T | 3888 | 37.78% |
| Mel.109.Rep.2 | chr2 | 74682140 | 74682140 | G | A | 5830 | 32.54% |
| Mel.109.Rep.2 | chr3 | 67048644 | 67048644 | C | T | 6743 | 82.79% |
| Mel.109.Rep.2 | chr5 | 145826781 | 145826781 | G | A | 1161 | 22.05% |
| Mel.109.Rep.2 | chr5 | 150080667 | 150080667 | C | T | 4913 | 54.03% |
| Mel.109.Rep.2 | chr6 | 30640796 | 30640796 | G | A | 5120 | 4.94% |
| Mel.109.Rep.2 | chr7 | 39605969 | 39605969 | G | A | 5651 | 13.10% |
| Mel.109.Rep.2 | chr7 | 39605970 | 39605970 | G | A | 5635 | 13.52% |
| Mel.109.Rep.2 | chr7 | 56174183 | 56174183 | G | A | 5397 | 32.31% |
| Mel.109.Rep.2 | chr7 | 140453136 | 140453136 | A | T | 4051 | 42.71% |
| Mel.109.Rep.2 | chr8 | 30601668 | 30601668 | G | A | 5194 | 11.05% |
| Mel.109.Rep.2 | chr8 | 30601669 | 30601669 | G | A | 5170 | 36.38% |
| Mel.109.Rep.2 | chr9 | 130700157 | 130700157 | C | T | 1435 | 61.46% |
| Mel.109.Rep.2 | chr9 | 131038409 | 131038409 | G | A | 6082 | 52.55% |
| Mel.109.Rep.2 | chr11 | 8704277 | 8704277 | G | A | 5114 | 81.20% |
| Mel.109.Rep.2 | chr11 | 61560113 | 61560113 | G | A | 5224 | 57.71% |
| Mel.109.Rep.2 | chr12 | 81693130 | 81693130 | C | T | 5476 | 4.33% |
| Mel.109.Rep.2 | chr13 | 41345346 | 41345346 | C | T | 4728 | 41.12% |
| Mel.109.Rep.2 | chr16 | 2510095 | 2510095 | G | A | 5707 | 66.39% |
| Mel.109.Rep.2 | chr16 | 2510096 | 2510096 | G | A | 5751 | 10.05% |
| Mel.109.Rep.2 | chr17 | 30771480 | 30771480 | C | T | 3518 | 67.34% |
| Mel.109.Rep.2 | chr20 | 34129795 | 34129795 | G | A | 3305 | 4.18% |
| Mel.109.Rep.2 | chr22 | 43010992 | 43010992 | G | A | 5904 | 55.36% |
| Mel.111.Rep.1 | chr1 | 25559064 | 25559064 | C | T | 2660 | 38.06% |
| Mel.111.Rep.1 | chr1 | 155904250 | 155904250 | C | T | 7589 | 42.59% |
| Mel.111.Rep.1 | chr2 | 10830118 | 10830118 | G | A | 1867 | 32.83% |
| Mel.111.Rep.1 | chr2 | 32390904 | 32390904 | C | T | 700 | 50% |
| Mel.111.Rep.1 | chr2 | 32390905 | 32390905 | C | T | 704 | 49.72% |
| Mel.111.Rep.1 | chr2 | 74682122 | 74682122 | G | A | 3830 | 12.53% |
| Mel.111.Rep.1 | chr2 | 105953996 | 105953996 | C | T | 4345 | 14.75% |
| Mel.111.Rep.1 | chr3 | 16306504 | 16306504 | C | T | 1929 | 14.42% |
| Mel.111.Rep.1 | chr3 | 16306505 | 16306505 | C | T | 1933 | 61.02% |
| Mel.111.Rep.1 | chr5 | 145826780 | 145826780 | C | T | 2774 | 33.72% |
| Mel.111.Rep.1 | chr6 | 30640795 | 30640795 | G | A | 3216 | 52.15% |
| Mel.111.Rep.1 | chr6 | 30640796 | 30640796 | G | A | 3289 | 5.20% |
| Mel.111.Rep.1 | chr9 | 130700157 | 130700157 | C | T | 1378 | 44.34% |
| Mel.111.Rep.1 | chrX | 48830734 | 48830734 | G | A | 3203 | 5.43% |
| Mel.111.Rep.2 | chr1 | 25559064 | 25559064 | C | T | 2254 | 41.44% |
| Mel.111.Rep.2 | chr1 | 155904250 | 155904250 | C | T | 6913 | 20.14% |
| Mel.111.Rep.2 | chr2 | 10830118 | 10830118 | G | A | 1346 | 18.28% |
| Mel.111.Rep.2 | chr2 | 32390904 | 32390904 | C | T | 550 | 47.64% |
| Mel.111.Rep.2 | chr2 | 32390905 | 32390905 | C | T | 553 | 47.74% |
| Mel.111.Rep.2 | chr2 | 74682122 | 74682122 | G | A | 3295 | 22.61% |
| Mel.111.Rep.2 | chr2 | 105953996 | 105953996 | C | T | 4462 | 35.88% |
| Mel.111.Rep.2 | chr3 | 16306504 | 16306504 | C | T | 1718 | 67% |
| Mel.111.Rep.2 | chr3 | 16306505 | 16306505 | C | T | 1723 | 11.09% |
| Mel.111.Rep.2 | chr5 | 145826780 | 145826780 | C | T | 2341 | 49.19% |
| Mel.111.Rep.2 | chr5 | 150080667 | 150080667 | C | T | 1078 | 33.67% |
| Mel.111.Rep.2 | chr6 | 30640795 | 30640795 | G | A | 2662 | 30.09% |
| Mel.111.Rep.2 | chr6 | 30640796 | 30640796 | G | A | 2669 | 6.11% |
| Mel.111.Rep.2 | chr7 | 56174183 | 56174183 | G | A | 1409 | 50.64% |
| Mel.111.Rep.2 | chr9 | 130700157 | 130700157 | C | T | 1056 | 21.40% |
| Mel.111.Rep.2 | chr19 | 15768976 | 15768976 | C | T | 513 | 30.86% |
| Mel.112.Rep.1 | chr1 | 25559063 | 25559063 | C | T | 5087 | 37.47% |
| Mel.112.Rep.1 | chr4 | 152020703 | 152020703 | G | A | 5580 | 33.56% |
| Mel.112.Rep.1 | chr5 | 150080667 | 150080667 | C | T | 2618 | 31.32% |
| Mel.112.Rep.1 | chr14 | 53173817 | 53173817 | G | A | 357 | 83.19% |
| Mel.112.Rep.1 | chr17 | 30771480 | 30771480 | C | T | 3815 | 96.75% |
| Mel.112.Rep.2 | chr1 | 25559063 | 25559063 | C | T | 4853 | 13.62% |
| Mel.112.Rep.2 | chr1 | 153963228 | 153963228 | C | A | 3986 | 3.06% |
| Mel.112.Rep.2 | chr4 | 152020703 | 152020703 | G | A | 5620 | 15.89% |
| Mel.112.Rep.2 | chr5 | 150080667 | 150080667 | C | T | 2948 | 26.94% |
| Mel.112.Rep.2 | chr10 | 7830003 | 7830003 | G | A | 2661 | 29.43% |
| Mel.112.Rep.2 | chr14 | 53173817 | 53173817 | G | A | 440 | 45.91% |
| Mel.112.Rep.2 | chr17 | 30771480 | 30771480 | C | T | 3421 | 99.56% |
| Mel.113.Rep.1 | chr1 | 894682 | 894682 | C | T | 201 | 23.38% |
| Mel.113.Rep.1 | chr1 | 55181508 | 55181508 | G | A | 5164 | 38.36% |
| Mel.113.Rep.1 | chr1 | 153963207 | 153963207 | C | T | 1080 | 5.46% |
| Mel.113.Rep.1 | chr1 | 153963239 | 153963239 | C | T | 1057 | 6.53% |
| Mel.113.Rep.1 | chr1 | 155904250 | 155904250 | C | T | 8543 | 35.93% |
| Mel.113.Rep.1 | chr2 | 32390905 | 32390905 | C | T | 749 | 24.03% |
| Mel.113.Rep.1 | chr3 | 16306505 | 16306505 | C | T | 2090 | 66.35% |
| Mel.113.Rep.1 | chr3 | 48481605 | 48481605 | G | A | 5286 | 31.07% |
| Mel.113.Rep.1 | chr4 | 152020703 | 152020703 | G | A | 3051 | 5.41% |
| Mel.113.Rep.1 | chr7 | 39605969 | 39605969 | G | A | 5931 | 26.83% |
| Mel.113.Rep.1 | chr7 | 39605970 | 39605970 | G | A | 5890 | 26.93% |
| Mel.113.Rep.1 | chr7 | 56174183 | 56174183 | G | A | 1952 | 24.39% |
| Mel.113.Rep.1 | chr11 | 99932099 | 99932099 | C | T | 5887 | 31.66% |
| Mel.113.Rep.1 | chr13 | 41345346 | 41345346 | C | A | 1687 | 21% |
| Mel.113.Rep.1 | chr16 | 29802047 | 29802047 | C | T | 1106 | 5.42% |
| Mel.113.Rep.1 | chr17 | 30771481 | 30771481 | T | C | 4047 | 43.19% |
| Mel.113.Rep.1 | chr17 | 56769917 | 56769917 | C | T | 572 | 26.22% |
| Mel.113.Rep.1 | chr17 | 56769918 | 56769918 | C | T | 575 | 25.96% |
| Mel.113.Rep.1 | chr19 | 13885240 | 13885240 | C | T | 2226 | 30.77% |
| Mel.113.Rep.1 | chr19 | 13885241 | 13885241 | C | T | 2210 | 45.57% |
| Mel.113.Rep.1 | chr19 | 48248748 | 48248748 | G | A | 2459 | 61.64% |
| Mel.113.Rep.1 | chr20 | 34129795 | 34129795 | G | A | 822 | 19.95% |
| Mel.113.Rep.1 | chr22 | 43011001 | 43011001 | G | A | 5733 | 28.83% |
| Mel.113.Rep.1 | chr22 | 43011002 | 43011002 | G | A | 5720 | 28.92% |
| Mel.113.Rep.2 | chr1 | 55181508 | 55181508 | G | A | 4715 | 39.43% |
| Mel.113.Rep.2 | chr1 | 153963239 | 153963239 | C | T | 1183 | 4.65% |
| Mel.113.Rep.2 | chr1 | 155904250 | 155904250 | C | T | 7244 | 21.91% |
| Mel.113.Rep.2 | chr2 | 32390905 | 32390905 | C | T | 832 | 43.75% |
| Mel.113.Rep.2 | chr3 | 16306505 | 16306505 | C | T | 2018 | 56.54% |
| Mel.113.Rep.2 | chr3 | 48481605 | 48481605 | G | A | 5703 | 46.04% |
| Mel.113.Rep.2 | chr3 | 124449246 | 124449246 | C | T | 3529 | 3.77% |
| Mel.113.Rep.2 | chr4 | 152020703 | 152020703 | G | A | 2753 | 28.51% |
| Mel.113.Rep.2 | chr7 | 39605969 | 39605969 | G | A | 6288 | 17.70% |
| Mel.113.Rep.2 | chr7 | 39605970 | 39605970 | G | A | 6306 | 17.84% |
| Mel.113.Rep.2 | chr7 | 56174183 | 56174183 | G | A | 2077 | 47.93% |
| Mel.113.Rep.2 | chr11 | 99932099 | 99932099 | C | T | 6104 | 27.08% |
| Mel.113.Rep.2 | chr13 | 41345346 | 41345346 | C | A | 1933 | 40.20% |
| Mel.113.Rep.2 | chr17 | 30771481 | 30771481 | T | C | 3708 | 17.83% |
| Mel.113.Rep.2 | chr17 | 56769917 | 56769917 | C | T | 1020 | 35.69% |
| Mel.113.Rep.2 | chr17 | 56769918 | 56769918 | C | T | 1027 | 35.87% |
| Mel.113.Rep.2 | chr19 | 13885240 | 13885240 | C | T | 1800 | 48.03% |
| Mel.113.Rep.2 | chr19 | 13885241 | 13885241 | C | T | 1801 | 48.25% |
| Mel.113.Rep.2 | chr19 | 48248748 | 48248748 | G | A | 3046 | 78.23% |
| Mel.113.Rep.2 | chr20 | 34129795 | 34129795 | G | A | 909 | 28.71% |
| Mel.113.Rep.2 | chr22 | 43011001 | 43011001 | G | A | 5680 | 75.79% |
| Mel.113.Rep.2 | chr22 | 43011002 | 43011002 | G | A | 5649 | 75.39% |
| Mel.114.Rep.1 | chr2 | 32390904 | 32390904 | C | T | 709 | 45.56% |
| Mel.114.Rep.1 | chr3 | 48481581 | 48481581 | C | T | 2988 | 35.21% |
| Mel.114.Rep.1 | chr7 | 140453136 | 140453136 | A | T | 2647 | 34.95% |
| Mel.114.Rep.1 | chr12 | 7080016 | 7080016 | G | A | 2149 | 18.61% |
| Mel.114.Rep.2 | chr7 | 140453136 | 140453136 | A | T | 795 | 7.92% |
| Mel.115.Rep.1 | chr1 | 25559063 | 25559063 | C | T | 10823 | 11.48% |
| Mel.115.Rep.1 | chr1 | 155904250 | 155904250 | C | T | 10552 | 13.93% |
| Mel.115.Rep.1 | chr2 | 74682104 | 74682104 | G | A | 9914 | 14.94% |
| Mel.115.Rep.1 | chr3 | 16306505 | 16306505 | C | T | 9120 | 3.87% |
| Mel.115.Rep.1 | chr4 | 53611041 | 53611041 | C | T | 18315 | 4.21% |
| Mel.115.Rep.1 | chr5 | 150080667 | 150080667 | C | T | 9358 | 13.01% |
| Mel.115.Rep.1 | chr6 | 30640796 | 30640796 | G | A | 9251 | 47.55% |
| Mel.115.Rep.1 | chr7 | 39605969 | 39605969 | G | A | 3570 | 38.40% |
| Mel.115.Rep.1 | chr9 | 130700157 | 130700157 | C | T | 6305 | 26.17% |
| Mel.115.Rep.1 | chr13 | 41345346 | 41345346 | C | T | 13148 | 36.21% |
| Mel.115.Rep.1 | chr16 | 2510095 | 2510095 | G | A | 8304 | 13.99% |
| Mel.115.Rep.1 | chr16 | 2510096 | 2510096 | G | A | 8258 | 13.99% |
| Mel.115.Rep.1 | chr16 | 67694198 | 67694198 | G | A | 3451 | 3.30% |
| Mel.115.Rep.1 | chr17 | 30771480 | 30771480 | C | T | 8907 | 8.78% |
| Mel.115.Rep.1 | chr17 | 56769918 | 56769918 | C | T | 18245 | 12.03% |
| Mel.115.Rep.1 | chr19 | 3435235 | 3435235 | C | T | 9162 | 24.98% |
| Mel.115.Rep.1 | chr20 | 34129795 | 34129795 | G | A | 13780 | 17.08% |
| Mel.115.Rep.1 | chr22 | 43011001 | 43011001 | G | A | 10096 | 4.15% |
| Mel.115.Rep.2 | chr1 | 25559063 | 25559063 | C | T | 10369 | 18.05% |
| Mel.115.Rep.2 | chr1 | 153963222 | 153963222 | G | A | 11085 | 3.58% |
| Mel.115.Rep.2 | chr1 | 155904250 | 155904250 | C | T | 9716 | 12.46% |
| Mel.115.Rep.2 | chr2 | 74682104 | 74682104 | G | A | 9776 | 10.96% |
| Mel.115.Rep.2 | chr5 | 150080667 | 150080667 | C | T | 9572 | 19.83% |
| Mel.115.Rep.2 | chr6 | 30640796 | 30640796 | G | A | 9151 | 79.08% |
| Mel.115.Rep.2 | chr7 | 39605969 | 39605969 | G | A | 2377 | 67.02% |
| Mel.115.Rep.2 | chr9 | 130700157 | 130700157 | C | T | 6118 | 11.85% |
| Mel.115.Rep.2 | chr13 | 41345346 | 41345346 | C | T | 12601 | 42.56% |
| Mel.115.Rep.2 | chr16 | 2510095 | 2510095 | G | A | 8737 | 55.73% |
| Mel.115.Rep.2 | chr16 | 2510096 | 2510096 | G | A | 8733 | 55.58% |
| Mel.115.Rep.2 | chr17 | 30771480 | 30771480 | C | T | 7336 | 16.93% |
| Mel.115.Rep.2 | chr17 | 56769918 | 56769918 | C | T | 17038 | 20% |
| Mel.115.Rep.2 | chr19 | 3435235 | 3435235 | C | T | 8815 | 13.25% |
| Mel.115.Rep.2 | chr20 | 34129795 | 34129795 | G | A | 13414 | 23.56% |
| Mel.116.Rep.1 | chr1 | 25559064 | 25559064 | C | T | 5738 | 31.36% |
| Mel.116.Rep.1 | chr1 | 55181528 | 55181528 | G | A | 6849 | 15.03% |
| Mel.116.Rep.1 | chr1 | 55181529 | 55181529 | G | A | 6845 | 15.05% |
| Mel.116.Rep.1 | chr3 | 16306504 | 16306504 | C | T | 11728 | 77.85% |
| Mel.116.Rep.1 | chr3 | 124449234 | 124449234 | G | A | 9151 | 90.05% |
| Mel.116.Rep.1 | chr5 | 150080667 | 150080667 | C | T | 10184 | 9.80% |
| Mel.116.Rep.1 | chr7 | 39605970 | 39605970 | G | A | 650 | 7.69% |
| Mel.116.Rep.1 | chr8 | 30601668 | 30601668 | G | A | 11043 | 76.26% |
| Mel.116.Rep.1 | chr8 | 30601669 | 30601669 | G | A | 11051 | 76.07% |
| Mel.116.Rep.1 | chr16 | 2510095 | 2510095 | G | A | 8599 | 95.41% |
| Mel.116.Rep.1 | chr16 | 2510096 | 2510096 | G | A | 8613 | 95% |
| Mel.116.Rep.1 | chr17 | 56769917 | 56769917 | C | T | 15653 | 99.09% |
| Mel.116.Rep.1 | chr17 | 56769918 | 56769918 | C | T | 15626 | 99.24% |
| Mel.116.Rep.2 | chr1 | 25559064 | 25559064 | C | T | 7214 | 14.52% |
| Mel.116.Rep.2 | chr1 | 55181528 | 55181528 | G | A | 7931 | 15.10% |
| Mel.116.Rep.2 | chr1 | 55181529 | 55181529 | G | A | 7922 | 15.15% |
| Mel.116.Rep.2 | chr2 | 32390905 | 32390905 | C | T | 7280 | 53.05% |
| Mel.116.Rep.2 | chr3 | 16306504 | 16306504 | C | T | 7340 | 24.65% |
| Mel.116.Rep.2 | chr3 | 124449234 | 124449234 | G | A | 8211 | 10.35% |
| Mel.116.Rep.2 | chr19 | 17970682 | 17970682 | C | T | 14373 | 4.92% |
| Mel.116.Rep.2 | chr19 | 50169131 | 50169131 | C | T | 4624 | 3.22% |
| Mel.117.Rep.1 | chr3 | 48481581 | 48481581 | C | T | 4118 | 55.15% |
| Mel.117.Rep.1 | chr4 | 152020703 | 152020703 | G | A | 3936 | 43.41% |
| Mel.117.Rep.1 | chr7 | 140453136 | 140453136 | A | T | 2986 | 70.62% |
| Mel.117.Rep.1 | chr7 | 140453137 | 140453137 | C | T | 2974 | 70.44% |
| Mel.117.Rep.1 | chr12 | 81693131 | 81693131 | C | A | 3192 | 5.52% |
| Mel.117.Rep.1 | chr13 | 41345355 | 41345355 | C | T | 2942 | 5.95% |
| Mel.117.Rep.1 | chr16 | 83841526 | 83841526 | C | T | 2006 | 36.49% |
| Mel.117.Rep.1 | chr19 | 17970682 | 17970682 | C | T | 3704 | 37.99% |
| Mel.117.Rep.1 | chr20 | 34129795 | 34129795 | G | A | 2065 | 24.55% |
| Mel.117.Rep.2 | chr3 | 48481581 | 48481581 | C | T | 4136 | 33.85% |
| Mel.117.Rep.2 | chr4 | 152020703 | 152020703 | G | A | 5077 | 28.13% |
| Mel.117.Rep.2 | chr7 | 140453136 | 140453136 | A | T | 4187 | 75.57% |
| Mel.117.Rep.2 | chr7 | 140453137 | 140453137 | C | T | 4182 | 75.16% |
| Mel.117.Rep.2 | chr16 | 83841526 | 83841526 | C | T | 2868 | 41.91% |
| Mel.117.Rep.2 | chr19 | 17970682 | 17970682 | C | T | 4747 | 48.90% |
| Mel.117.Rep.2 | chr20 | 34129795 | 34129795 | G | A | 3047 | 19.30% |
| Mel.118.Rep.1 | chr6 | 30640795 | 30640795 | G | A | 1131 | 89.83% |
| Mel.118.Rep.1 | chr6 | 30640796 | 30640796 | G | A | 1126 | 89.52% |
| Mel.118.Rep.1 | chr7 | 56174183 | 56174183 | G | A | 2184 | 43.79% |
| Mel.118.Rep.1 | chr7 | 140453136 | 140453136 | A | T | 1887 | 30.95% |
| Mel.118.Rep.1 | chr8 | 30601668 | 30601668 | G | A | 5150 | 67.44% |
| Mel.118.Rep.1 | chr19 | 17970682 | 17970682 | C | T | 1434 | 4.95% |
| Mel.118.Rep.1 | chr22 | 43011001 | 43011001 | G | A | 4522 | 43.85% |
| Mel.118.Rep.2 | chr6 | 30640795 | 30640795 | G | A | 2385 | 7.25% |
| Mel.118.Rep.2 | chr6 | 30640796 | 30640796 | G | A | 2371 | 7% |
| Mel.118.Rep.2 | chr7 | 140453136 | 140453136 | A | T | 3548 | 31.93% |
| Mel.118.Rep.2 | chr19 | 17970682 | 17970682 | C | T | 1234 | 99.35% |
| Mel.119.Rep.1 | chr3 | 16306508 | 16306508 | C | T | 2452 | 5.59% |
| Mel.119.Rep.1 | chr4 | 152020703 | 152020703 | G | A | 2284 | 31.71% |
| Mel.119.Rep.1 | chr7 | 140453136 | 140453136 | A | T | 1685 | 38.69% |
| Mel.119.Rep.2 | chr3 | 16306508 | 16306508 | C | T | 1942 | 10.40% |
| Mel.119.Rep.2 | chr3 | 52542195 | 52542195 | C | T | 224 | 34.82% |
| Mel.119.Rep.2 | chr4 | 152020703 | 152020703 | G | A | 1576 | 20.94% |
| Mel.119.Rep.2 | chr7 | 140453136 | 140453136 | A | T | 1812 | 77.87% |
| Mel.120.Rep.1 | chr3 | 16306504 | 16306504 | C | T | 2656 | 12.84% |
| Mel.120.Rep.1 | chr3 | 16306505 | 16306505 | C | T | 2652 | 12.22% |
| Mel.120.Rep.1 | chr6 | 30640795 | 30640795 | G | A | 2437 | 14.85% |
| Mel.120.Rep.1 | chr6 | 30640796 | 30640796 | G | A | 2437 | 14.81% |
| Mel.120.Rep.1 | chr7 | 39605969 | 39605969 | G | A | 3600 | 99.19% |
| Mel.120.Rep.1 | chr7 | 140453136 | 140453136 | A | G | 1911 | 3.72% |
| Mel.120.Rep.1 | chr11 | 46958261 | 46958261 | C | T | 7017 | 57.71% |
| Mel.120.Rep.1 | chr11 | 46958262 | 46958262 | C | T | 7031 | 57.84% |
| Mel.120.Rep.1 | chr13 | 41345346 | 41345346 | C | T | 1667 | 64.38% |
| Mel.120.Rep.1 | chr19 | 13885240 | 13885240 | C | T | 1427 | 56.20% |
| Mel.120.Rep.2 | chr3 | 16306505 | 16306505 | C | T | 2213 | 47.27% |
| Mel.120.Rep.2 | chr6 | 30640795 | 30640795 | G | A | 2153 | 61.77% |
| Mel.120.Rep.2 | chr6 | 30640796 | 30640796 | G | A | 2155 | 61.67% |
| Mel.120.Rep.2 | chr7 | 39605969 | 39605969 | G | A | 2683 | 34.92% |
| Mel.120.Rep.2 | chr11 | 46958261 | 46958261 | C | T | 7055 | 44.13% |
| Mel.120.Rep.2 | chr11 | 46958262 | 46958262 | C | T | 7069 | 44.03% |
| Mel.120.Rep.2 | chr13 | 41345346 | 41345346 | C | T | 1587 | 12.10% |
| Mel.121.Rep.1 | chr19 | 17970682 | 17970682 | C | T | 3758 | 69.15% |
| Mel.121.Rep.2 | chr19 | 17970682 | 17970682 | C | T | 5227 | 6.75% |
| Mel.122.Rep.1 | chr7 | 140453136 | 140453136 | A | T | 2334 | 46.23% |
| Mel.122.Rep.1 | chr8 | 125551321 | 125551321 | G | A | 1699 | 6.30% |
| Mel.122.Rep.1 | chr11 | 8704277 | 8704277 | G | A | 1625 | 13.60% |
| Mel.122.Rep.2 | chr5 | 150080671 | 150080671 | C | T | 1361 | 9.11% |
| Mel.122.Rep.2 | chr7 | 140453136 | 140453136 | A | T | 2435 | 19.43% |
| Mel.122.Rep.2 | chr8 | 30601669 | 30601669 | G | A | 5514 | 5.51% |
| Mel.122.Rep.2 | chr8 | 125551321 | 125551321 | G | A | 1919 | 37.15% |
| Mel.123.Rep.1 | chr3 | 16306504 | 16306504 | C | T | 1769 | 35.44% |
| Mel.123.Rep.1 | chr3 | 124449234 | 124449234 | G | A | 2608 | 31.43% |
| Mel.123.Rep.1 | chr4 | 152020703 | 152020703 | G | A | 1840 | 25.88% |
| Mel.123.Rep.1 | chr4 | 152020713 | 152020713 | G | A | 1846 | 50.81% |
| Mel.123.Rep.1 | chr7 | 140453136 | 140453136 | A | T | 2183 | 66.97% |
| Mel.123.Rep.1 | chr7 | 140453137 | 140453137 | C | T | 2186 | 66.65% |
| Mel.123.Rep.1 | chr13 | 41345346 | 41345346 | C | T | 1567 | 31.61% |
| Mel.123.Rep.1 | chr16 | 2510095 | 2510095 | G | A | 4828 | 34.78% |
| Mel.123.Rep.1 | chr19 | 4247041 | 4247041 | G | A | 1574 | 7.12% |
| Mel.123.Rep.2 | chr1 | 155904276 | 155904276 | C | T | 5030 | 3.58% |
| Mel.123.Rep.2 | chr3 | 16306504 | 16306504 | C | T | 1289 | 28.47% |
| Mel.123.Rep.2 | chr3 | 124449234 | 124449234 | G | A | 2392 | 46.70% |
| Mel.123.Rep.2 | chr4 | 152020703 | 152020703 | G | A | 1441 | 44.41% |
| Mel.123.Rep.2 | chr4 | 152020713 | 152020713 | G | A | 1460 | 30.21% |
| Mel.123.Rep.2 | chr7 | 140453136 | 140453136 | A | T | 1741 | 46.21% |
| Mel.123.Rep.2 | chr7 | 140453137 | 140453137 | C | T | 1739 | 45.89% |
| Mel.123.Rep.2 | chr12 | 7080017 | 7080017 | G | A | 1304 | 3.83% |
| Mel.123.Rep.2 | chr13 | 41345346 | 41345346 | C | T | 1080 | 41.67% |
| Mel.123.Rep.2 | chr16 | 2510095 | 2510095 | G | A | 3163 | 31.90% |
| Mel.124.Rep.1 | chr1 | 115256529 | 115256529 | T | C | 3310 | 47.46% |
| Mel.124.Rep.1 | chr1 | 153963239 | 153963239 | C | T | 972 | 72.94% |
| Mel.124.Rep.1 | chr2 | 10830118 | 10830118 | G | A | 1858 | 36.83% |
| Mel.124.Rep.1 | chr6 | 30640795 | 30640795 | G | A | 3408 | 43.16% |
| Mel.124.Rep.1 | chr8 | 30601669 | 30601669 | G | A | 3592 | 51.48% |
| Mel.124.Rep.1 | chr8 | 125551344 | 125551344 | C | T | 755 | 69.40% |
| Mel.124.Rep.1 | chr11 | 61560113 | 61560113 | G | A | 3934 | 46.05% |
| Mel.124.Rep.1 | chr13 | 41345355 | 41345355 | C | T | 1627 | 3.38% |
| Mel.124.Rep.1 | chr19 | 13885240 | 13885240 | C | T | 2175 | 66.07% |
| Mel.124.Rep.2 | chr1 | 115256529 | 115256529 | T | C | 4722 | 62.18% |
| Mel.124.Rep.2 | chr1 | 153963239 | 153963239 | C | T | 1142 | 57.84% |
| Mel.124.Rep.2 | chr2 | 10830118 | 10830118 | G | A | 2359 | 87.37% |
| Mel.124.Rep.2 | chr6 | 30640795 | 30640795 | G | A | 3517 | 46.03% |
| Mel.124.Rep.2 | chr8 | 30601669 | 30601669 | G | A | 5330 | 77.34% |
| Mel.124.Rep.2 | chr8 | 125551344 | 125551344 | C | T | 1080 | 80.19% |
| Mel.124.Rep.2 | chr11 | 61560113 | 61560113 | G | A | 5635 | 55.63% |
| Mel.124.Rep.2 | chr19 | 13885240 | 13885240 | C | T | 2988 | 73.46% |
| Mel.125.Rep.1 | chr1 | 55181528 | 55181528 | G | A | 2670 | 4.94% |
| Mel.125.Rep.1 | chr3 | 16306505 | 16306505 | C | T | 1160 | 30.09% |
| Mel.125.Rep.1 | chr3 | 48481602 | 48481602 | C | T | 2948 | 15.30% |
| Mel.125.Rep.1 | chr16 | 2510095 | 2510095 | G | A | 2797 | 16.59% |
| Mel.125.Rep.1 | chr19 | 48248748 | 48248748 | G | A | 1194 | 36.43% |
| Mel.125.Rep.1 | chr20 | 34129792 | 34129792 | G | A | 314 | 55.73% |
| Mel.125.Rep.2 | chr1 | 55181528 | 55181528 | G | A | 3419 | 41.42% |
| Mel.125.Rep.2 | chr3 | 16306505 | 16306505 | C | T | 1980 | 53.18% |
| Mel.125.Rep.2 | chr3 | 48481602 | 48481602 | C | T | 3947 | 18.29% |
| Mel.125.Rep.2 | chr8 | 30601668 | 30601668 | G | A | 5111 | 3.87% |
| Mel.125.Rep.2 | chr8 | 30601669 | 30601669 | G | C | 5110 | 3.87% |
| Mel.125.Rep.2 | chr16 | 67694195 | 67694195 | G | A | 959 | 6.05% |
| Mel.125.Rep.2 | chr19 | 48248748 | 48248748 | G | A | 1907 | 41.68% |
| Mel.125.Rep.2 | chr20 | 34129792 | 34129792 | G | A | 572 | 4.20% |
| Mel.126.Rep.1 | chr7 | 53103759 | 53103759 | G | A | 919 | 11.32% |
| Mel.126.Rep.1 | chr8 | 30601668 | 30601668 | G | A | 5401 | 3.89% |
| Mel.126.Rep.1 | chr11 | 99932099 | 99932099 | C | T | 5263 | 16.09% |
| Mel.126.Rep.1 | chr19 | 38202426 | 38202426 | G | A | 7834 | 6.14% |
| Mel.126.Rep.1 | chr20 | 34129795 | 34129795 | G | A | 851 | 23.50% |
| Mel.126.Rep.2 | chr11 | 65063342 | 65063342 | C | T | 4630 | 8.42% |
| Mel.126.Rep.2 | chr12 | 7080016 | 7080016 | G | A | 2266 | 18.31% |
| Mel.126.Rep.2 | chr20 | 34129795 | 34129795 | G | A | 685 | 11.70% |
| Mel.127.Rep.1 | chr1 | 115256530 | 115256530 | G | T | 3472 | 51.83% |
| Mel.127.Rep.1 | chr1 | 155904250 | 155904250 | C | T | 9287 | 3.19% |
| Mel.127.Rep.1 | chr17 | 30771480 | 30771480 | C | T | 447 | 98.21% |
| Mel.127.Rep.1 | chr19 | 3435235 | 3435235 | C | T | 348 | 57.76% |
| Mel.127.Rep.2 | chr1 | 115256530 | 115256530 | G | T | 3241 | 32.53% |
| Mel.127.Rep.2 | chr3 | 48481602 | 48481602 | C | T | 2871 | 27.76% |
| Mel.127.Rep.2 | chr8 | 125551345 | 125551345 | C | T | 1283 | 3.28% |
| Mel.127.Rep.2 | chr16 | 67694211 | 67694211 | C | T | 1585 | 25.55% |
| Mel.127.Rep.2 | chr17 | 30771480 | 30771480 | C | T | 2018 | 9.91% |
| Mel.127.Rep.2 | chr19 | 3435235 | 3435235 | C | T | 404 | 5.20% |
| Mel.128.Rep.1 | chr8 | 30601647 | 30601647 | T | C | 6127 | 4.96% |
| Mel.128.Rep.2 | chr1 | 153963227 | 153963227 | C | T | 9232 | 17.65% |
| Mel.129.Rep.1 | chr1 | 155904250 | 155904250 | C | T | 4667 | 34.93% |
| Mel.129.Rep.1 | chr2 | 32390904 | 32390904 | C | T | 2893 | 29.38% |
| Mel.129.Rep.1 | chr3 | 16306505 | 16306505 | C | T | 4057 | 44.66% |
| Mel.129.Rep.1 | chr3 | 48481605 | 48481605 | G | A | 4352 | 5.03% |
| Mel.129.Rep.1 | chr4 | 152020703 | 152020703 | G | A | 4358 | 12.95% |
| Mel.129.Rep.1 | chr7 | 140453136 | 140453136 | A | T | 1644 | 39.99% |
| Mel.129.Rep.1 | chr7 | 140453137 | 140453137 | C | T | 1646 | 39.79% |
| Mel.129.Rep.1 | chr8 | 30601668 | 30601668 | G | A | 4013 | 18.61% |
| Mel.129.Rep.1 | chr8 | 30601669 | 30601669 | G | A | 3979 | 18.72% |
| Mel.129.Rep.1 | chr11 | 61560122 | 61560122 | C | T | 3373 | 7.29% |
| Mel.129.Rep.1 | chr16 | 2510096 | 2510096 | G | A | 2772 | 79.29% |
| Mel.129.Rep.1 | chr16 | 67694198 | 67694198 | G | A | 1194 | 53.60% |
| Mel.129.Rep.1 | chr16 | 83841525 | 83841525 | C | T | 1681 | 27.31% |
| Mel.129.Rep.1 | chr19 | 4247060 | 4247060 | G | A | 1569 | 33.91% |
| Mel.129.Rep.2 | chr1 | 25559063 | 25559063 | C | T | 5911 | 3.91% |
| Mel.129.Rep.2 | chr1 | 155904250 | 155904250 | C | T | 6727 | 22.57% |
| Mel.129.Rep.2 | chr2 | 10830118 | 10830118 | G | A | 7519 | 5.07% |
| Mel.129.Rep.2 | chr2 | 32390904 | 32390904 | C | T | 3315 | 12.64% |
| Mel.129.Rep.2 | chr2 | 32390905 | 32390905 | C | T | 3310 | 27.92% |
| Mel.129.Rep.2 | chr3 | 16306505 | 16306505 | C | T | 4801 | 37.33% |
| Mel.129.Rep.2 | chr4 | 152020703 | 152020703 | G | A | 4900 | 48.90% |
| Mel.129.Rep.2 | chr6 | 30640795 | 30640795 | G | A | 3132 | 4.18% |
| Mel.129.Rep.2 | chr6 | 30640796 | 30640796 | G | A | 3115 | 4.27% |
| Mel.129.Rep.2 | chr7 | 140453136 | 140453136 | A | T | 2533 | 25.70% |
| Mel.129.Rep.2 | chr7 | 140453137 | 140453137 | C | T | 2544 | 25.47% |
| Mel.129.Rep.2 | chr10 | 127512083 | 127512083 | G | A | 4390 | 34.31% |
| Mel.129.Rep.2 | chr16 | 2510096 | 2510096 | G | A | 3747 | 44.76% |
| Mel.129.Rep.2 | chr16 | 67694198 | 67694198 | G | A | 247 | 56.68% |
| Mel.129.Rep.2 | chr16 | 83841525 | 83841525 | C | T | 1661 | 26.19% |
| Mel.129.Rep.2 | chr19 | 4247073 | 4247073 | C | T | 1372 | 3.94% |
| Mel.130.Rep.1 | chr1 | 25559063 | 25559063 | C | T | 2059 | 6.75% |
| Mel.130.Rep.1 | chr1 | 153963239 | 153963239 | C | T | 796 | 16.96% |
| Mel.130.Rep.1 | chr1 | 155904250 | 155904250 | C | T | 5655 | 26.81% |
| Mel.130.Rep.1 | chr2 | 74682176 | 74682176 | C | T | 2987 | 14.43% |
| Mel.130.Rep.1 | chr7 | 56174183 | 56174183 | G | A | 1566 | 33.29% |
| Mel.130.Rep.1 | chr9 | 130700157 | 130700157 | C | T | 1163 | 20.22% |
| Mel.130.Rep.1 | chr10 | 7830003 | 7830003 | G | A | 177 | 27.12% |
| Mel.130.Rep.1 | chr13 | 41345346 | 41345346 | C | T | 980 | 20.10% |
| Mel.130.Rep.1 | chr16 | 2510095 | 2510095 | G | A | 2557 | 94.60% |
| Mel.130.Rep.1 | chr17 | 30771480 | 30771480 | C | G | 1926 | 23.05% |
| Mel.130.Rep.1 | chr19 | 13885240 | 13885240 | C | T | 1937 | 7.07% |
| Mel.130.Rep.1 | chr19 | 13885241 | 13885241 | C | T | 1937 | 11.77% |
| Mel.130.Rep.1 | chr19 | 17970682 | 17970682 | C | T | 1276 | 14.81% |
| Mel.130.Rep.1 | chr20 | 34129795 | 34129795 | G | A | 617 | 10.37% |
| Mel.130.Rep.1 | chr22 | 43011001 | 43011001 | G | A | 4229 | 20.29% |
| Mel.130.Rep.2 | chr1 | 153963239 | 153963239 | C | T | 296 | 11.82% |
| Mel.130.Rep.2 | chr1 | 155904250 | 155904250 | C | T | 1642 | 18.51% |
| Mel.130.Rep.2 | chr2 | 74682176 | 74682176 | C | T | 1477 | 5.01% |
| Mel.130.Rep.2 | chr3 | 16306504 | 16306504 | C | T | 743 | 3.50% |
| Mel.130.Rep.2 | chr7 | 56174183 | 56174183 | G | A | 679 | 34.32% |
| Mel.130.Rep.2 | chr9 | 130700157 | 130700157 | C | T | 578 | 15.40% |
| Mel.130.Rep.2 | chr13 | 41345346 | 41345346 | C | T | 561 | 24.42% |
| Mel.130.Rep.2 | chr16 | 2510095 | 2510095 | G | A | 1619 | 40.83% |
| Mel.130.Rep.2 | chr17 | 30771480 | 30771480 | C | T | 750 | 4% |
| Mel.130.Rep.2 | chr19 | 4247078 | 4247078 | G | A | 481 | 4.57% |
| Mel.130.Rep.2 | chr19 | 48248748 | 48248748 | G | A | 637 | 3.61% |
| Mel.130.Rep.2 | chr22 | 43011001 | 43011001 | G | A | 1406 | 14.15% |
| Mel.131.Rep.1 | chr7 | 140453136 | 140453136 | A | T | 3860 | 57.41% |
| Mel.131.Rep.1 | chr8 | 125551345 | 125551345 | C | T | 3779 | 3.25% |
| Mel.131.Rep.2 | chr7 | 140453136 | 140453136 | A | T | 3142 | 51.75% |
| Mel.131.Rep.2 | chr12 | 7080017 | 7080017 | G | A | 4944 | 12.18% |
| Mel.131.Rep.2 | chr16 | 836281 | 836281 | G | A | 3251 | 8.74% |
| Mel.131.Rep.2 | chr16 | 83841525 | 83841525 | C | T | 2548 | 4.08% |
| Mel.132.Rep.1 | chr2 | 10830118 | 10830118 | G | A | 1888 | 3.02% |
| Mel.132.Rep.1 | chr7 | 140453136 | 140453136 | A | T | 2535 | 26.63% |
| Mel.132.Rep.1 | chr16 | 2510095 | 2510095 | G | T | 7090 | 3.15% |
| Mel.132.Rep.2 | chr3 | 16306505 | 16306505 | C | T | 3364 | 3.24% |
| Mel.132.Rep.2 | chr7 | 140453136 | 140453136 | A | T | 3712 | 25.65% |
| Mel.133.Rep.1 | chr7 | 39605970 | 39605970 | G | A | 4488 | 18.36% |
| Mel.133.Rep.1 | chr7 | 140453136 | 140453136 | A | T | 2749 | 59.69% |
| Mel.133.Rep.1 | chr7 | 140453137 | 140453137 | C | T | 2732 | 59.11% |
| Mel.133.Rep.2 | chr3 | 16306505 | 16306505 | C | T | 2047 | 25.70% |
| Mel.133.Rep.2 | chr7 | 39605970 | 39605970 | G | A | 3464 | 22.26% |
| Mel.133.Rep.2 | chr7 | 140453136 | 140453136 | A | T | 2730 | 74.35% |
| Mel.133.Rep.2 | chr7 | 140453137 | 140453137 | C | T | 2697 | 74.08% |
| Mel.134.Rep.1 | chr1 | 55181508 | 55181508 | G | A | 5524 | 42.07% |
| Mel.134.Rep.1 | chr3 | 68802215 | 68802215 | G | A | 850 | 37.46% |
| Mel.134.Rep.1 | chr7 | 140453136 | 140453136 | A | T | 2909 | 75.46% |
| Mel.134.Rep.1 | chr7 | 140453137 | 140453137 | C | T | 2889 | 75.18% |
| Mel.134.Rep.1 | chr19 | 48248740 | 48248740 | G | A | 2147 | 78.89% |
| Mel.134.Rep.1 | chr19 | 50169132 | 50169132 | C | T | 127 | 65.35% |
| Mel.134.Rep.1 | chr20 | 34129795 | 34129795 | G | A | 808 | 27.63% |
| Mel.134.Rep.2 | chr1 | 55181508 | 55181508 | G | A | 5327 | 41.34% |
| Mel.134.Rep.2 | chr3 | 48481603 | 48481603 | C | T | 5374 | 3.20% |
| Mel.134.Rep.2 | chr3 | 68802215 | 68802215 | G | A | 1189 | 38.47% |
| Mel.134.Rep.2 | chr7 | 140453136 | 140453136 | A | T | 2681 | 74% |
| Mel.134.Rep.2 | chr7 | 140453137 | 140453137 | C | T | 2676 | 73.58% |
| Mel.134.Rep.2 | chr19 | 48248740 | 48248740 | G | A | 2413 | 62.22% |
| Mel.134.Rep.2 | chr19 | 50169132 | 50169132 | C | T | 175 | 88.57% |
| Mel.134.Rep.2 | chr20 | 34129795 | 34129795 | G | A | 749 | 35.48% |
| Mel.135.Rep.1 | chr1 | 153963239 | 153963239 | C | T | 1070 | 14.67% |
| Mel.135.Rep.1 | chr2 | 32390904 | 32390904 | C | T | 792 | 7.32% |
| Mel.135.Rep.1 | chr2 | 32390905 | 32390905 | C | T | 795 | 7.92% |
| Mel.135.Rep.1 | chr2 | 74682104 | 74682104 | G | A | 2991 | 32.87% |
| Mel.135.Rep.1 | chr7 | 56174183 | 56174183 | G | A | 2066 | 3.73% |
| Mel.135.Rep.1 | chr8 | 30601668 | 30601668 | G | A | 5492 | 4.83% |
| Mel.135.Rep.1 | chr8 | 125551345 | 125551345 | C | T | 1769 | 6.95% |
| Mel.135.Rep.1 | chr11 | 99932099 | 99932099 | C | T | 4715 | 64.76% |
| Mel.135.Rep.1 | chr19 | 17970682 | 17970682 | C | T | 1552 | 6.06% |
| Mel.135.Rep.2 | chr4 | 152020703 | 152020703 | G | A | 2963 | 4.22% |
| Mel.135.Rep.2 | chr5 | 150080667 | 150080667 | C | T | 1586 | 9.91% |
| Mel.135.Rep.2 | chr7 | 56174183 | 56174183 | G | A | 2147 | 5.22% |
| Mel.135.Rep.2 | chr8 | 30601668 | 30601668 | G | A | 6089 | 14.27% |
| Mel.135.Rep.2 | chr11 | 99932099 | 99932099 | C | T | 5728 | 5.45% |
| Mel.135.Rep.2 | chr16 | 2510095 | 2510095 | G | A | 7185 | 9.02% |
| Mel.135.Rep.2 | chr19 | 17970682 | 17970682 | C | T | 2014 | 15.90% |
| Mel.136.Rep.1 | chr1 | 894682 | 894682 | C | T | 1022 | 57.44% |
| Mel.136.Rep.1 | chr1 | 153963227 | 153963227 | C | T | 1293 | 23.43% |
| Mel.136.Rep.1 | chr1 | 153963239 | 153963239 | C | T | 1280 | 6.80% |
| Mel.136.Rep.1 | chr1 | 155904250 | 155904250 | C | T | 11938 | 19.26% |
| Mel.136.Rep.1 | chr2 | 32390905 | 32390905 | C | T | 1033 | 16.36% |
| Mel.136.Rep.1 | chr3 | 124449234 | 124449234 | G | A | 4442 | 13.60% |
| Mel.136.Rep.1 | chr3 | 124449246 | 124449246 | C | T | 4365 | 13.84% |
| Mel.136.Rep.1 | chr4 | 152020703 | 152020703 | G | A | 3705 | 3.54% |
| Mel.136.Rep.1 | chr5 | 145826781 | 145826781 | G | A | 3441 | 4.30% |
| Mel.136.Rep.1 | chr7 | 39605969 | 39605969 | G | A | 7727 | 29.70% |
| Mel.136.Rep.1 | chr7 | 39605970 | 39605970 | G | A | 7683 | 30.21% |
| Mel.136.Rep.1 | chr9 | 130700157 | 130700157 | C | T | 2306 | 21.55% |
| Mel.136.Rep.1 | chr11 | 46958261 | 46958261 | C | T | 9414 | 8.94% |
| Mel.136.Rep.1 | chr11 | 46958262 | 46958262 | C | T | 9507 | 9.08% |
| Mel.136.Rep.1 | chr12 | 7080016 | 7080016 | G | A | 2537 | 18.37% |
| Mel.136.Rep.1 | chr12 | 117923349 | 117923349 | G | A | 919 | 18.23% |
| Mel.136.Rep.1 | chr13 | 41345346 | 41345346 | C | T | 2122 | 19.56% |
| Mel.136.Rep.1 | chr16 | 2510096 | 2510096 | G | A | 10696 | 7.82% |
| Mel.136.Rep.1 | chr16 | 29802047 | 29802047 | C | T | 1376 | 10.83% |
| Mel.136.Rep.1 | chr19 | 3435235 | 3435235 | C | T | 738 | 21.41% |
| Mel.136.Rep.1 | chr19 | 4247060 | 4247060 | G | A | 2316 | 14.77% |
| Mel.136.Rep.1 | chr19 | 17970682 | 17970682 | C | T | 2707 | 3.58% |
| Mel.136.Rep.1 | chr20 | 34129795 | 34129795 | G | A | 1026 | 23.10% |
| Mel.136.Rep.1 | chr22 | 43010992 | 43010992 | G | A | 7749 | 22.47% |
| Mel.136.Rep.2 | chr1 | 894682 | 894682 | C | T | 1054 | 90.42% |
| Mel.136.Rep.2 | chr1 | 55181528 | 55181528 | G | A | 5178 | 4.07% |
| Mel.136.Rep.2 | chr1 | 153963182 | 153963182 | C | T | 1272 | 3.14% |
| Mel.136.Rep.2 | chr1 | 153963227 | 153963227 | C | T | 1323 | 17.99% |
| Mel.136.Rep.2 | chr1 | 153963239 | 153963239 | C | T | 1308 | 15.98% |
| Mel.136.Rep.2 | chr1 | 155904250 | 155904250 | C | T | 10908 | 27.02% |
| Mel.136.Rep.2 | chr2 | 32390905 | 32390905 | C | T | 815 | 18.40% |
| Mel.136.Rep.2 | chr3 | 124449234 | 124449234 | G | A | 4267 | 10.85% |
| Mel.136.Rep.2 | chr3 | 124449246 | 124449246 | C | T | 4192 | 11.12% |
| Mel.136.Rep.2 | chr4 | 53611041 | 53611041 | C | T | 530 | 13.96% |
| Mel.136.Rep.2 | chr4 | 152020703 | 152020703 | G | A | 3307 | 9.22% |
| Mel.136.Rep.2 | chr7 | 39605969 | 39605969 | G | A | 6113 | 14.62% |
| Mel.136.Rep.2 | chr7 | 39605970 | 39605970 | G | A | 6150 | 15.01% |
| Mel.136.Rep.2 | chr8 | 30601668 | 30601668 | G | A | 6991 | 3.19% |
| Mel.136.Rep.2 | chr9 | 130700157 | 130700157 | C | T | 2032 | 4.92% |
| Mel.136.Rep.2 | chr11 | 46958261 | 46958261 | C | T | 8344 | 19.58% |
| Mel.136.Rep.2 | chr11 | 46958262 | 46958262 | C | T | 8417 | 19.64% |
| Mel.136.Rep.2 | chr12 | 7080016 | 7080016 | G | A | 2472 | 9.71% |
| Mel.136.Rep.2 | chr12 | 117923349 | 117923349 | G | A | 904 | 12.29% |
| Mel.136.Rep.2 | chr13 | 41345346 | 41345346 | C | T | 1918 | 25.55% |
| Mel.136.Rep.2 | chr16 | 29802047 | 29802047 | C | T | 1487 | 30.87% |
| Mel.136.Rep.2 | chr19 | 3435235 | 3435235 | C | T | 661 | 28.90% |
| Mel.136.Rep.2 | chr19 | 4247060 | 4247060 | G | A | 1984 | 26.26% |
| Mel.136.Rep.2 | chr20 | 34129795 | 34129795 | G | A | 1091 | 6.14% |
| Mel.136.Rep.2 | chr22 | 43010992 | 43010992 | G | A | 6762 | 20.26% |
| Mel.137.Rep.1 | chr2 | 32390905 | 32390905 | C | T | 673 | 4.01% |
| Mel.137.Rep.1 | chr3 | 124449234 | 124449234 | G | A | 2417 | 21.73% |
| Mel.137.Rep.1 | chr19 | 3435234 | 3435234 | C | T | 333 | 16.52% |
| Mel.137.Rep.2 | chr2 | 32390905 | 32390905 | C | T | 906 | 12.14% |
| Mel.137.Rep.2 | chr3 | 124449234 | 124449234 | G | A | 4106 | 27.53% |
| Mel.137.Rep.2 | chr11 | 61560107 | 61560107 | G | A | 6369 | 15.79% |
| Mel.137.Rep.2 | chr16 | 2510095 | 2510095 | G | A | 7218 | 16.76% |
| Mel.137.Rep.2 | chr16 | 2510096 | 2510096 | G | A | 7179 | 16.83% |
| Mel.138.Rep.1 | chr1 | 231114784 | 231114784 | C | T | 799 | 21.90% |
| Mel.138.Rep.1 | chr2 | 32390904 | 32390904 | C | T | 3684 | 46.09% |
| Mel.138.Rep.1 | chr7 | 140453136 | 140453136 | A | T | 1907 | 7.92% |
| Mel.138.Rep.1 | chr16 | 2510095 | 2510095 | G | A | 5701 | 9.10% |
| Mel.138.Rep.1 | chr16 | 29802047 | 29802047 | C | T | 3197 | 33.22% |
| Mel.138.Rep.1 | chr19 | 4247072 | 4247072 | C | T | 2338 | 7.36% |
| Mel.138.Rep.1 | chr19 | 17970682 | 17970682 | C | T | 4512 | 27.06% |
| Mel.138.Rep.2 | chr1 | 231114784 | 231114784 | C | T | 1026 | 33.33% |
| Mel.138.Rep.2 | chr2 | 32390904 | 32390904 | C | T | 3026 | 51.72% |
| Mel.138.Rep.2 | chr5 | 145826780 | 145826780 | C | T | 1907 | 3.88% |
| Mel.138.Rep.2 | chr7 | 140453136 | 140453136 | A | T | 3363 | 14.09% |
| Mel.138.Rep.2 | chr12 | 81693130 | 81693130 | C | T | 4628 | 8.86% |
| Mel.138.Rep.2 | chr12 | 132537870 | 132537870 | T | C | 229 | 10.04% |
| Mel.138.Rep.2 | chr16 | 29802047 | 29802047 | C | T | 2396 | 11.15% |
| Mel.138.Rep.2 | chr16 | 67694194 | 67694194 | G | A | 1986 | 15.11% |
| Mel.138.Rep.2 | chr17 | 56769918 | 56769918 | C | T | 2351 | 20.38% |
| Mel.138.Rep.2 | chr19 | 4247072 | 4247072 | C | T | 2504 | 36.22% |
| Mel.138.Rep.2 | chr19 | 17970682 | 17970682 | C | T | 4707 | 3.44% |
| Mel.138.Rep.2 | chr20 | 34129795 | 34129795 | G | A | 2921 | 15.89% |
| Mel.139.Rep.1 | chr1 | 115256530 | 115256530 | G | A | 3943 | 8.50% |
| Mel.139.Rep.1 | chr1 | 153963227 | 153963227 | C | T | 1156 | 9.34% |
| Mel.139.Rep.1 | chr1 | 153963239 | 153963239 | C | T | 1128 | 17.20% |
| Mel.139.Rep.1 | chr1 | 155904250 | 155904250 | C | T | 10123 | 9.02% |
| Mel.139.Rep.1 | chr2 | 10830114 | 10830114 | C | T | 1869 | 15.68% |
| Mel.139.Rep.1 | chr2 | 32390905 | 32390905 | C | T | 831 | 8.90% |
| Mel.139.Rep.1 | chr3 | 16306504 | 16306504 | C | T | 2463 | 4.55% |
| Mel.139.Rep.1 | chr3 | 16306505 | 16306505 | C | T | 2458 | 4.60% |
| Mel.139.Rep.1 | chr4 | 53611041 | 53611041 | C | T | 542 | 17.53% |
| Mel.139.Rep.1 | chr4 | 69093838 | 69093838 | C | T | 1699 | 3.82% |
| Mel.139.Rep.1 | chr4 | 152020703 | 152020703 | G | A | 3074 | 5.47% |
| Mel.139.Rep.1 | chr5 | 150080667 | 150080667 | C | T | 1511 | 7.68% |
| Mel.139.Rep.1 | chr8 | 125551344 | 125551344 | C | T | 1912 | 5.18% |
| Mel.139.Rep.1 | chr9 | 131038409 | 131038409 | G | A | 3915 | 19.74% |
| Mel.139.Rep.1 | chr11 | 46958262 | 46958262 | C | T | 7285 | 3.01% |
| Mel.139.Rep.1 | chr16 | 2510095 | 2510095 | G | A | 9458 | 26.65% |
| Mel.139.Rep.1 | chr17 | 30771480 | 30771480 | C | T | 4467 | 36.60% |
| Mel.139.Rep.1 | chr19 | 4247041 | 4247041 | G | A | 2124 | 7.11% |
| Mel.139.Rep.1 | chr19 | 17970682 | 17970682 | C | T | 1835 | 4.74% |
| Mel.139.Rep.1 | chr22 | 43011001 | 43011001 | G | A | 5858 | 6.88% |
| Mel.139.Rep.1 | chr22 | 43011002 | 43011002 | G | A | 5876 | 6.81% |
| Mel.139.Rep.2 | chr1 | 153963227 | 153963227 | C | T | 1048 | 16.70% |
| Mel.139.Rep.2 | chr1 | 153963239 | 153963239 | C | T | 1036 | 16.70% |
| Mel.139.Rep.2 | chr1 | 155904250 | 155904250 | C | T | 9177 | 9.58% |
| Mel.139.Rep.2 | chr3 | 16306504 | 16306504 | C | T | 2156 | 19.06% |
| Mel.139.Rep.2 | chr3 | 16306505 | 16306505 | C | T | 2162 | 18.96% |
| Mel.139.Rep.2 | chr3 | 124449234 | 124449234 | G | A | 3407 | 7.04% |
| Mel.139.Rep.2 | chr4 | 53611041 | 53611041 | C | T | 379 | 16.62% |
| Mel.139.Rep.2 | chr4 | 69093838 | 69093838 | C | T | 1705 | 21.82% |
| Mel.139.Rep.2 | chr4 | 152020703 | 152020703 | G | A | 2915 | 15% |
| Mel.139.Rep.2 | chr5 | 150080667 | 150080667 | C | T | 1380 | 20.94% |
| Mel.139.Rep.2 | chr9 | 131038409 | 131038409 | G | A | 3604 | 3.25% |
| Mel.139.Rep.2 | chr11 | 46958262 | 46958262 | C | T | 6729 | 11.73% |
| Mel.139.Rep.2 | chr11 | 99932099 | 99932099 | C | T | 4405 | 5.36% |
| Mel.139.Rep.2 | chr13 | 41345346 | 41345346 | C | T | 1378 | 3.19% |
| Mel.139.Rep.2 | chr16 | 2510095 | 2510095 | G | A | 7667 | 29.83% |
| Mel.139.Rep.2 | chr16 | 83841525 | 83841525 | C | T | 507 | 29.39% |
| Mel.139.Rep.2 | chr16 | 83841526 | 83841526 | C | T | 523 | 28.11% |
| Mel.139.Rep.2 | chr19 | 4247041 | 4247041 | G | A | 1859 | 10.38% |
| Mel.139.Rep.2 | chr19 | 4247073 | 4247073 | C | T | 1848 | 6.44% |
| Mel.139.Rep.2 | chr19 | 17970682 | 17970682 | C | T | 1554 | 14.93% |
| Mel.140.Rep.1 | chr1 | 153963227 | 153963227 | C | T | 5273 | 4.76% |
| Mel.140.Rep.1 | chr1 | 153963239 | 153963239 | C | T | 5260 | 3.94% |
| Mel.140.Rep.1 | chr1 | 155904250 | 155904250 | C | T | 6147 | 9.57% |
| Mel.140.Rep.1 | chr2 | 32390904 | 32390904 | C | T | 3302 | 4.18% |
| Mel.140.Rep.1 | chr2 | 105953996 | 105953996 | C | T | 4331 | 6.49% |
| Mel.140.Rep.1 | chr3 | 124449234 | 124449234 | G | A | 4382 | 16.86% |
| Mel.140.Rep.1 | chr5 | 150080667 | 150080667 | C | T | 4345 | 36.32% |
| Mel.140.Rep.1 | chr6 | 30640796 | 30640796 | G | A | 5145 | 4.82% |
| Mel.140.Rep.1 | chr7 | 56174183 | 56174183 | G | A | 5217 | 6.82% |
| Mel.140.Rep.1 | chr7 | 140453136 | 140453136 | A | T | 4326 | 8.16% |
| Mel.140.Rep.1 | chr7 | 140453137 | 140453137 | C | T | 4334 | 8.01% |
| Mel.140.Rep.1 | chr8 | 125551344 | 125551344 | C | T | 3530 | 7.93% |
| Mel.140.Rep.1 | chr9 | 131038414 | 131038414 | G | A | 4908 | 3.57% |
| Mel.140.Rep.1 | chr10 | 7830002 | 7830002 | G | A | 4210 | 7.67% |
| Mel.140.Rep.1 | chr13 | 41345346 | 41345346 | C | T | 3713 | 3.07% |
| Mel.140.Rep.1 | chr13 | 41345349 | 41345349 | C | T | 3708 | 13.65% |
| Mel.140.Rep.1 | chr16 | 2510095 | 2510095 | G | A | 5166 | 7.40% |
| Mel.140.Rep.1 | chr19 | 17970682 | 17970682 | C | T | 4873 | 3.57% |
| Mel.140.Rep.2 | chr1 | 894682 | 894682 | C | T | 778 | 5.40% |
| Mel.140.Rep.2 | chr1 | 155904250 | 155904250 | C | T | 5953 | 5.36% |
| Mel.140.Rep.2 | chr1 | 231114784 | 231114784 | C | T | 1080 | 6.85% |
| Mel.140.Rep.2 | chr3 | 124449234 | 124449234 | G | A | 4450 | 9.01% |
| Mel.140.Rep.2 | chr4 | 25314329 | 25314329 | C | T | 623 | 9.95% |
| Mel.140.Rep.2 | chr5 | 150080667 | 150080667 | C | T | 3969 | 20.91% |
| Mel.140.Rep.2 | chr7 | 140453136 | 140453136 | A | T | 4365 | 9.71% |
| Mel.140.Rep.2 | chr7 | 140453137 | 140453137 | C | T | 4364 | 9.69% |
| Mel.140.Rep.2 | chr8 | 125551321 | 125551321 | G | A | 3486 | 4.02% |
| Mel.140.Rep.2 | chr8 | 125551344 | 125551344 | C | T | 3465 | 6.67% |
| Mel.140.Rep.2 | chr13 | 41345349 | 41345349 | C | T | 3877 | 18.08% |
| Mel.140.Rep.2 | chr16 | 2510095 | 2510095 | G | A | 5009 | 42.23% |
| Mel.140.Rep.2 | chr19 | 4247041 | 4247041 | G | A | 2748 | 10.99% |
| Mel.141.Rep.1 | chr1 | 894683 | 894683 | C | T | 914 | 4.38% |
| Mel.143.Rep.1 | chr1 | 25559063 | 25559063 | C | T | 663 | 28.96% |
| Mel.143.Rep.1 | chr1 | 55181509 | 55181509 | G | A | 4809 | 4.66% |
| Mel.143.Rep.1 | chr1 | 153963227 | 153963227 | C | T | 4231 | 82.23% |
| Mel.143.Rep.1 | chr1 | 155904250 | 155904250 | C | T | 2237 | 26.24% |
| Mel.143.Rep.1 | chr3 | 16306505 | 16306505 | C | T | 3064 | 37.61% |
| Mel.143.Rep.1 | chr16 | 67694211 | 67694211 | C | T | 105 | 63.81% |
| Mel.143.Rep.1 | chr19 | 17970682 | 17970682 | C | T | 1268 | 62.12% |
| Mel.143.Rep.1 | chr20 | 34129795 | 34129795 | G | A | 1921 | 56.09% |
| Mel.143.Rep.2 | chr1 | 25559063 | 25559063 | C | T | 155 | 85.16% |
| Mel.143.Rep.2 | chr1 | 153963227 | 153963227 | C | T | 2985 | 15.08% |
| Mel.143.Rep.2 | chr1 | 155904250 | 155904250 | C | T | 1466 | 6.82% |
| Mel.143.Rep.2 | chr3 | 16306505 | 16306505 | C | T | 2411 | 43.63% |
| Mel.143.Rep.2 | chr8 | 30601668 | 30601668 | G | A | 1400 | 52.57% |
| Mel.143.Rep.2 | chr8 | 30601669 | 30601669 | G | A | 1400 | 52.36% |
| Mel.143.Rep.2 | chr19 | 17970682 | 17970682 | C | T | 713 | 81.18% |
| Mel.143.Rep.2 | chr20 | 34129795 | 34129795 | G | A | 1272 | 67.19% |
| Mel.144.Rep.1 | chr1 | 153963227 | 153963227 | C | T | 4987 | 15.54% |
| Mel.144.Rep.1 | chr2 | 105954000 | 105954000 | C | T | 2513 | 3.50% |
| Mel.144.Rep.1 | chr3 | 68802220 | 68802220 | G | A | 3554 | 6.42% |
| Mel.144.Rep.1 | chr11 | 61560122 | 61560122 | C | T | 1654 | 92.32% |
| Mel.144.Rep.1 | chr19 | 4247060 | 4247060 | G | A | 2005 | 9.73% |
| Mel.144.Rep.2 | chr1 | 115256530 | 115256530 | G | T | 6613 | 26.47% |
| Mel.144.Rep.2 | chr12 | 7080016 | 7080016 | G | A | 5471 | 27.56% |
| Mel.144.Rep.2 | chr12 | 7080017 | 7080017 | G | A | 5486 | 27.60% |
| Mel.144.Rep.2 | chr19 | 4247060 | 4247060 | G | A | 3040 | 23.78% |
| Mel.145.Rep.1 | chr2 | 74682104 | 74682104 | G | A | 2856 | 3.19% |
| Mel.145.Rep.1 | chr2 | 105953960 | 105953960 | G | A | 1933 | 22.87% |
| Mel.145.Rep.1 | chr11 | 61560110 | 61560110 | A | G | 2125 | 22.60% |
| Mel.145.Rep.1 | chr14 | 53173817 | 53173817 | G | A | 2090 | 43.30% |
| Mel.145.Rep.1 | chr19 | 4247073 | 4247073 | C | T | 1846 | 17.06% |
| Mel.145.Rep.1 | chr19 | 17970682 | 17970682 | C | T | 2195 | 29.17% |
| Mel.145.Rep.1 | chr19 | 50169131 | 50169131 | C | T | 1598 | 22.97% |
| Mel.145.Rep.2 | chr2 | 105953960 | 105953960 | G | A | 1596 | 37.28% |
| Mel.145.Rep.2 | chr11 | 61560110 | 61560110 | A | G | 2044 | 24.72% |
| Mel.145.Rep.2 | chr14 | 53173817 | 53173817 | G | A | 1725 | 44.52% |
| Mel.145.Rep.2 | chr19 | 4247073 | 4247073 | C | T | 1619 | 12.17% |
| Mel.145.Rep.2 | chr19 | 17970682 | 17970682 | C | T | 2142 | 13.82% |
| Mel.145.Rep.2 | chr19 | 50169131 | 50169131 | C | T | 1640 | 9.15% |
| Mel.146.Rep.1 | chr1 | 894682 | 894682 | C | T | 1825 | 47.07% |
| Mel.146.Rep.1 | chr4 | 152020703 | 152020703 | G | A | 2270 | 37.80% |
| Mel.146.Rep.1 | chr16 | 67694194 | 67694194 | G | A | 547 | 97.81% |
| Mel.146.Rep.2 | chr7 | 140453136 | 140453136 | A | T | 536 | 53.36% |
| Mel.146.Rep.2 | chr8 | 30601668 | 30601668 | G | A | 684 | 64.91% |
| Mel.147.Rep.1 | chr1 | 115256530 | 115256530 | G | T | 4682 | 61.53% |
| Mel.147.Rep.1 | chr7 | 56174183 | 56174183 | G | A | 2664 | 25.26% |
| Mel.147.Rep.1 | chr16 | 2510096 | 2510096 | G | A | 2656 | 35.20% |
| Mel.147.Rep.1 | chr19 | 10514238 | 10514238 | C | T | 1846 | 44.10% |
| Mel.147.Rep.1 | chr22 | 43011002 | 43011002 | G | A | 2724 | 29% |
| Mel.147.Rep.2 | chr1 | 115256530 | 115256530 | G | T | 4494 | 77.25% |
| Mel.147.Rep.2 | chr1 | 153276253 | 153276253 | G | A | 4765 | 4.64% |
| Mel.147.Rep.2 | chr7 | 56174183 | 56174183 | G | A | 2490 | 45.44% |
| Mel.147.Rep.2 | chr16 | 2510096 | 2510096 | G | A | 2171 | 24.09% |
| Mel.147.Rep.2 | chr19 | 10514238 | 10514238 | C | T | 3195 | 32.71% |
| Mel.147.Rep.2 | chr22 | 43011002 | 43011002 | G | A | 3359 | 82.97% |
| Mel.148.Rep.1 | chr1 | 153963228 | 153963228 | C | T | 4065 | 50.06% |
| Mel.148.Rep.1 | chr12 | 7080017 | 7080017 | G | T | 3406 | 8.16% |
| Mel.148.Rep.2 | chr7 | 140453136 | 140453136 | A | T | 3938 | 40.17% |
| Mel.149.Rep.1 | chr1 | 115256530 | 115256530 | G | T | 4730 | 98.90% |
| Mel.149.Rep.1 | chr19 | 48248740 | 48248740 | G | A | 3942 | 38.32% |
| Mel.150.Rep.2 | chr2 | 168098326 | 168098326 | C | T | 2505 | 6.03% |
| Mel.151.Rep.1 | chr7 | 140453136 | 140453136 | A | T | 3318 | 51.96% |
| Mel.151.Rep.1 | chr11 | 61560107 | 61560107 | G | A | 1975 | 18.33% |
| Mel.151.Rep.1 | chr16 | 2510095 | 2510095 | G | A | 2585 | 52.61% |
| Mel.151.Rep.1 | chr16 | 2510096 | 2510096 | G | A | 2583 | 52.42% |
| Mel.151.Rep.1 | chr19 | 17970682 | 17970682 | C | T | 2866 | 8.69% |
| Mel.151.Rep.2 | chr7 | 140453136 | 140453136 | A | T | 3755 | 85.70% |
| Mel.151.Rep.2 | chr11 | 61560107 | 61560107 | G | A | 3578 | 44.20% |
| Mel.151.Rep.2 | chr16 | 2510095 | 2510095 | G | A | 2890 | 96.16% |
| Mel.151.Rep.2 | chr16 | 2510096 | 2510096 | G | A | 2889 | 95.95% |
| Mel.151.Rep.2 | chr16 | 83841526 | 83841526 | C | A | 2578 | 3.92% |
| Mel.152.Rep.1 | chr7 | 39605970 | 39605970 | G | A | 2611 | 58.18% |
| Mel.152.Rep.1 | chr7 | 56174183 | 56174183 | G | A | 4375 | 7.86% |
| Mel.152.Rep.1 | chr7 | 140453136 | 140453136 | A | T | 4372 | 65.35% |
| Mel.152.Rep.1 | chr7 | 140453137 | 140453137 | C | T | 4370 | 65.31% |
| Mel.152.Rep.2 | chr7 | 39605970 | 39605970 | G | A | 793 | 88.02% |
| Mel.152.Rep.2 | chr7 | 140453136 | 140453136 | A | T | 1100 | 76.73% |
| Mel.152.Rep.2 | chr7 | 140453137 | 140453137 | C | T | 1101 | 76.57% |
| Mel.152.Rep.2 | chr9 | 130700157 | 130700157 | C | T | 201 | 60.20% |
| Mel.153.Rep.1 | chr1 | 153963207 | 153963207 | C | T | 1622 | 4.07% |
| Mel.153.Rep.1 | chr1 | 153963239 | 153963239 | C | T | 1595 | 4.08% |
| Mel.153.Rep.1 | chr1 | 155904250 | 155904250 | C | A | 6555 | 38.05% |
| Mel.153.Rep.1 | chr2 | 10830114 | 10830114 | C | T | 1686 | 90.33% |
| Mel.153.Rep.1 | chr2 | 74682104 | 74682104 | G | A | 3671 | 15.25% |
| Mel.153.Rep.1 | chr3 | 16306504 | 16306504 | C | T | 4682 | 58.50% |
| Mel.153.Rep.1 | chr3 | 48481602 | 48481602 | C | T | 2439 | 8.45% |
| Mel.153.Rep.1 | chr10 | 7830002 | 7830002 | G | A | 284 | 52.11% |
| Mel.153.Rep.1 | chr11 | 8704277 | 8704277 | G | A | 2875 | 46.71% |
| Mel.153.Rep.1 | chr13 | 41345346 | 41345346 | C | T | 1952 | 14.86% |
| Mel.153.Rep.1 | chr16 | 29802023 | 29802023 | G | A | 1922 | 76.32% |
| Mel.153.Rep.1 | chr19 | 15768976 | 15768976 | C | T | 332 | 93.37% |
| Mel.153.Rep.2 | chr1 | 55181528 | 55181528 | G | A | 2923 | 4.58% |
| Mel.153.Rep.2 | chr1 | 55181529 | 55181529 | G | A | 2926 | 29.32% |
| Mel.153.Rep.2 | chr1 | 153963207 | 153963207 | C | T | 1367 | 59.15% |
| Mel.153.Rep.2 | chr1 | 155904250 | 155904250 | C | T | 3198 | 36.02% |
| Mel.153.Rep.2 | chr2 | 10830114 | 10830114 | C | T | 1528 | 6.15% |
| Mel.153.Rep.2 | chr2 | 74682104 | 74682104 | G | A | 1939 | 42.55% |
| Mel.153.Rep.2 | chr3 | 16306504 | 16306504 | C | T | 3018 | 29.22% |
| Mel.153.Rep.2 | chr3 | 48481602 | 48481602 | C | T | 1987 | 50.78% |
| Mel.153.Rep.2 | chr9 | 131038414 | 131038414 | G | A | 2136 | 29.73% |
| Mel.153.Rep.2 | chr11 | 8704277 | 8704277 | G | A | 1596 | 91.17% |
| Mel.153.Rep.2 | chr11 | 46958261 | 46958261 | C | T | 3516 | 29.22% |
| Mel.153.Rep.2 | chr11 | 46958262 | 46958262 | C | T | 3516 | 28.97% |
| Mel.153.Rep.2 | chr13 | 41345346 | 41345346 | C | T | 1365 | 18.61% |
| Mel.153.Rep.2 | chr16 | 29802023 | 29802023 | G | A | 1708 | 39.86% |
| Mel.154.Rep.1 | chr1 | 115256530 | 115256530 | G | T | 229 | 96.94% |
| Mel.154.Rep.1 | chr3 | 16306504 | 16306504 | C | T | 3510 | 97.69% |
| Mel.154.Rep.1 | chr9 | 131038414 | 131038414 | G | A | 1851 | 49.54% |
| Mel.154.Rep.1 | chr19 | 4247072 | 4247072 | C | T | 249 | 66.27% |
| Mel.154.Rep.1 | chr20 | 34129830 | 34129830 | C | T | 847 | 69.54% |
| Mel.154.Rep.2 | chr1 | 115256530 | 115256530 | G | T | 658 | 56.99% |
| Mel.154.Rep.2 | chr3 | 16306504 | 16306504 | C | T | 3392 | 35.67% |
| Mel.154.Rep.2 | chr3 | 124449234 | 124449234 | G | A | 770 | 86.74% |
| Mel.154.Rep.2 | chr9 | 131038414 | 131038414 | G | A | 470 | 58.94% |
| Mel.154.Rep.2 | chr19 | 4247072 | 4247072 | C | T | 275 | 61.45% |
| Mel.155.Rep.1 | chr1 | 115256529 | 115256529 | T | A | 1203 | 10.14% |
| Mel.155.Rep.1 | chr1 | 155904250 | 155904250 | C | T | 4540 | 18.15% |
| Mel.155.Rep.1 | chr2 | 74682175 | 74682175 | C | T | 2383 | 48.55% |
| Mel.155.Rep.1 | chr4 | 53611041 | 53611041 | C | T | 601 | 15.47% |
| Mel.155.Rep.1 | chr5 | 145826780 | 145826780 | C | T | 705 | 7.80% |
| Mel.155.Rep.1 | chr19 | 17970682 | 17970682 | C | T | 2469 | 62% |
| Mel.155.Rep.1 | chr19 | 54197615 | 54197615 | G | A | 1143 | 5.34% |
| Mel.155.Rep.2 | chr1 | 155904250 | 155904250 | C | T | 4768 | 8.49% |
| Mel.155.Rep.2 | chr2 | 74682175 | 74682175 | C | T | 1999 | 10.31% |
| Mel.155.Rep.2 | chr3 | 16306504 | 16306504 | C | T | 3805 | 7.05% |
| Mel.155.Rep.2 | chr3 | 16306505 | 16306505 | C | T | 3801 | 7.08% |
| Mel.155.Rep.2 | chr5 | 150080667 | 150080667 | C | T | 919 | 15.14% |
| Mel.155.Rep.2 | chr6 | 30640795 | 30640795 | G | T | 2416 | 31.83% |
| Mel.155.Rep.2 | chr7 | 39605969 | 39605969 | G | A | 1754 | 4.56% |
| Mel.155.Rep.2 | chr7 | 39605970 | 39605970 | G | A | 1746 | 4.47% |
| Mel.155.Rep.2 | chr8 | 125551344 | 125551344 | C | T | 1285 | 24.20% |
| Mel.155.Rep.2 | chr11 | 8704277 | 8704277 | G | A | 3020 | 4.34% |
| Mel.155.Rep.2 | chr19 | 17970682 | 17970682 | C | T | 2854 | 56.40% |
| Mel.155.Rep.2 | chr22 | 43011001 | 43011001 | G | A | 4830 | 4.22% |
| Mel.155.Rep.2 | chr22 | 43011002 | 43011002 | G | A | 4830 | 4.20% |
| Mel.156.Rep.1 | chr3 | 48481605 | 48481605 | G | A | 2806 | 3.49% |
| Mel.156.Rep.1 | chr7 | 140453136 | 140453136 | A | T | 3331 | 58.66% |
| Mel.156.Rep.1 | chr7 | 140453137 | 140453137 | C | T | 3331 | 58.99% |
| Mel.156.Rep.1 | chr19 | 48248748 | 48248748 | G | A | 3662 | 32.44% |
| Mel.156.Rep.2 | chr7 | 140453136 | 140453136 | A | T | 898 | 99.44% |
| Mel.156.Rep.2 | chr7 | 140453137 | 140453137 | C | T | 898 | 99.33% |
| Mel.156.Rep.2 | chr19 | 48248748 | 48248748 | G | A | 2166 | 25.88% |
| Mel.157.Rep.1 | chr7 | 140453136 | 140453136 | A | T | 2430 | 71.77% |
| Mel.157.Rep.1 | chr10 | 7830002 | 7830002 | G | A | 2404 | 19.14% |
| Mel.157.Rep.1 | chr10 | 7830003 | 7830003 | G | A | 2406 | 19.20% |
| Mel.157.Rep.2 | chr7 | 140453136 | 140453136 | A | T | 3303 | 65.97% |
| Mel.157.Rep.2 | chr10 | 7830002 | 7830002 | G | A | 2844 | 65.31% |
| Mel.157.Rep.2 | chr10 | 7830003 | 7830003 | G | A | 2867 | 65.50% |
| Mel.157.Rep.2 | chr19 | 17970682 | 17970682 | C | T | 3225 | 5.95% |
| Mel.158.Rep.2 | chr1 | 153963227 | 153963227 | C | T | 2031 | 16.54% |
| Mel.159.Rep.1 | chr1 | 115256530 | 115256530 | G | T | 6585 | 58.31% |
| Mel.159.Rep.1 | chr7 | 140453137 | 140453137 | C | A | 3710 | 4.29% |
| Mel.159.Rep.1 | chr16 | 83841525 | 83841525 | C | T | 2928 | 8.67% |
| Mel.159.Rep.2 | chr1 | 115256530 | 115256530 | G | T | 6477 | 30.46% |
| Mel.159.Rep.2 | chr2 | 74682176 | 74682176 | C | T | 3531 | 3.48% |
| Mel.159.Rep.2 | chr2 | 105954000 | 105954000 | C | T | 2726 | 3.05% |
| Mel.159.Rep.2 | chr19 | 17970682 | 17970682 | C | T | 3417 | 12.32% |
| Mel.160.Rep.1 | chr1 | 153963239 | 153963239 | C | T | 3450 | 3.01% |
| Mel.160.Rep.1 | chr3 | 16306505 | 16306505 | C | T | 1923 | 3.12% |
| Mel.160.Rep.1 | chr7 | 140453136 | 140453136 | A | T | 2011 | 27.40% |
| Mel.160.Rep.1 | chr20 | 34129795 | 34129795 | G | A | 1856 | 4.26% |
| Mel.160.Rep.2 | chr1 | 153963227 | 153963227 | C | T | 4243 | 4.31% |
| Mel.160.Rep.2 | chr7 | 140453136 | 140453136 | A | T | 1999 | 17.96% |
| Mel.160.Rep.2 | chr14 | 53173818 | 53173818 | G | A | 1655 | 28.22% |
| Mel.161.Rep.1 | chr1 | 153963207 | 153963207 | C | T | 3021 | 25.02% |
| Mel.161.Rep.1 | chr7 | 39605969 | 39605969 | G | A | 1237 | 33.58% |
| Mel.161.Rep.1 | chr7 | 39605970 | 39605970 | G | A | 1244 | 33.68% |
| Mel.162.Rep.1 | chr1 | 153963239 | 153963239 | C | T | 2197 | 57.01% |
| Mel.162.Rep.1 | chr7 | 39605969 | 39605969 | G | A | 2278 | 37.01% |
| Mel.162.Rep.1 | chr8 | 30601669 | 30601669 | G | A | 2420 | 97.27% |
| Mel.162.Rep.1 | chr8 | 125551345 | 125551345 | C | T | 644 | 97.51% |
| Mel.162.Rep.1 | chr9 | 131038414 | 131038414 | G | A | 2670 | 75.92% |
| Mel.162.Rep.1 | chr10 | 7830002 | 7830002 | G | A | 196 | 56.63% |
| Mel.162.Rep.1 | chr12 | 7080016 | 7080016 | G | A | 1515 | 16.41% |
| Mel.162.Rep.1 | chr12 | 7080017 | 7080017 | G | A | 1525 | 16.59% |
| Mel.162.Rep.1 | chr13 | 41345349 | 41345349 | C | T | 1790 | 78.93% |
| Mel.162.Rep.1 | chr13 | 41345350 | 41345350 | C | T | 1813 | 79.04% |
| Mel.162.Rep.1 | chr16 | 83841529 | 83841529 | C | T | 1466 | 74.35% |
| Mel.162.Rep.1 | chr19 | 13885241 | 13885241 | C | T | 2081 | 27.21% |
| Mel.162.Rep.2 | chr1 | 153963239 | 153963239 | C | T | 1856 | 39.39% |
| Mel.162.Rep.2 | chr7 | 39605969 | 39605969 | G | A | 3574 | 74.76% |
| Mel.162.Rep.2 | chr8 | 30601669 | 30601669 | G | A | 1837 | 48.88% |
| Mel.162.Rep.2 | chr8 | 125551345 | 125551345 | C | T | 815 | 85.03% |
| Mel.162.Rep.2 | chr9 | 131038414 | 131038414 | G | A | 2423 | 5.86% |
| Mel.162.Rep.2 | chr10 | 7830002 | 7830002 | G | A | 162 | 91.98% |
| Mel.162.Rep.2 | chr12 | 7080016 | 7080016 | G | A | 2362 | 6.92% |
| Mel.162.Rep.2 | chr12 | 7080017 | 7080017 | G | A | 2352 | 6.97% |
| Mel.162.Rep.2 | chr13 | 41345349 | 41345349 | C | T | 1707 | 55.45% |
| Mel.162.Rep.2 | chr13 | 41345350 | 41345350 | C | T | 1707 | 55.07% |
| Mel.162.Rep.2 | chr16 | 83841529 | 83841529 | C | T | 852 | 74.53% |
| Mel.162.Rep.2 | chr19 | 13885241 | 13885241 | C | T | 999 | 73.77% |
| Mel.162.Rep.2 | chr19 | 48248740 | 48248740 | G | A | 473 | 58.77% |
| Mel.163.Rep.1 | chr5 | 150080671 | 150080671 | C | A | 2334 | 3.56% |
| Mel.163.Rep.1 | chr7 | 140453136 | 140453136 | A | T | 2049 | 10.10% |
| Mel.163.Rep.2 | chr7 | 140453136 | 140453136 | A | T | 2403 | 12.32% |
| Mel.163.Rep.2 | chr19 | 13885240 | 13885240 | C | T | 1320 | 6.21% |
| Mel.164.Rep.1 | chr1 | 25559063 | 25559063 | C | T | 2405 | 64.06% |
| Mel.164.Rep.1 | chr1 | 115256529 | 115256529 | T | C | 914 | 34.57% |
| Mel.164.Rep.2 | chr1 | 25559063 | 25559063 | C | T | 3244 | 3.51% |
| Mel.164.Rep.2 | chr1 | 115256529 | 115256529 | T | C | 914 | 32.28% |
| Mel.165.Rep.1 | chr7 | 140453136 | 140453136 | A | T | 3050 | 69.80% |
| Mel.165.Rep.1 | chr19 | 4247045 | 4247045 | G | A | 2269 | 32.58% |
| Mel.165.Rep.2 | chr7 | 140453136 | 140453136 | A | T | 3180 | 66.32% |
| Mel.165.Rep.2 | chr19 | 4247045 | 4247045 | G | A | 2149 | 45.93% |
| Mel.166.Rep.1 | chr7 | 140453136 | 140453136 | A | T | 1711 | 4.09% |
| Mel.166.Rep.2 | chr10 | 7830003 | 7830003 | G | A | 1484 | 6.33% |
| Mel.167.Rep.1 | chr1 | 25559064 | 25559064 | C | T | 2847 | 11.35% |
| Mel.167.Rep.1 | chr1 | 153963227 | 153963227 | C | T | 3600 | 34.33% |
| Mel.167.Rep.1 | chr2 | 32390904 | 32390904 | C | T | 2212 | 8.41% |
| Mel.167.Rep.1 | chr2 | 74682104 | 74682104 | G | A | 3229 | 17.25% |
| Mel.167.Rep.1 | chr2 | 105954000 | 105954000 | C | T | 1766 | 49.49% |
| Mel.167.Rep.1 | chr3 | 16306504 | 16306504 | C | T | 2581 | 23.79% |
| Mel.167.Rep.1 | chr3 | 16306505 | 16306505 | C | T | 2582 | 36.79% |
| Mel.167.Rep.1 | chr5 | 150080667 | 150080667 | C | T | 2231 | 7.71% |
| Mel.167.Rep.1 | chr8 | 30601668 | 30601668 | G | A | 2052 | 22.12% |
| Mel.167.Rep.1 | chr9 | 131038409 | 131038409 | G | A | 2149 | 23.87% |
| Mel.167.Rep.1 | chr14 | 53173818 | 53173818 | G | A | 1339 | 84.09% |
| Mel.167.Rep.1 | chr16 | 83841525 | 83841525 | C | T | 1749 | 44.83% |
| Mel.167.Rep.1 | chr17 | 30771480 | 30771480 | C | T | 1232 | 90.50% |
| Mel.167.Rep.1 | chr19 | 4247073 | 4247073 | C | T | 1931 | 4.87% |
| Mel.167.Rep.2 | chr1 | 153963227 | 153963227 | C | T | 3577 | 69.72% |
| Mel.167.Rep.2 | chr2 | 32390904 | 32390904 | C | T | 1851 | 35.12% |
| Mel.167.Rep.2 | chr2 | 74682104 | 74682104 | G | A | 1986 | 39.07% |
| Mel.167.Rep.2 | chr2 | 105953985 | 105953985 | C | T | 1786 | 13.55% |
| Mel.167.Rep.2 | chr2 | 105954000 | 105954000 | C | T | 1785 | 60.34% |
| Mel.167.Rep.2 | chr3 | 16306504 | 16306504 | C | T | 2194 | 25.57% |
| Mel.167.Rep.2 | chr3 | 16306505 | 16306505 | C | T | 2196 | 34.79% |
| Mel.167.Rep.2 | chr7 | 53103787 | 53103787 | C | T | 1562 | 13.76% |
| Mel.167.Rep.2 | chr8 | 30601668 | 30601668 | G | A | 1359 | 56.81% |
| Mel.167.Rep.2 | chr8 | 125551345 | 125551345 | C | T | 134 | 29.32% |
| Mel.167.Rep.2 | chr10 | 127512083 | 127512083 | G | A | 1776 | 35.25% |
| Mel.167.Rep.2 | chr14 | 53173818 | 53173818 | G | A | 1224 | 25.98% |
| Mel.167.Rep.2 | chr16 | 83841525 | 83841525 | C | T | 1409 | 52.45% |
| Mel.167.Rep.2 | chr19 | 3435234 | 3435234 | C | T | 2320 | 12.76% |
| Mel.167.Rep.2 | chr19 | 3435235 | 3435235 | C | T | 2321 | 12.75% |
| Mel.167.Rep.2 | chr19 | 4247073 | 4247073 | C | T | 960 | 7.40% |
| Mel.168.Rep.1 | chr1 | 153963227 | 153963227 | C | T | 8432 | 35.82% |
| Mel.168.Rep.1 | chr1 | 155904250 | 155904250 | C | T | 7301 | 30.16% |
| Mel.168.Rep.1 | chr2 | 32390905 | 32390905 | C | T | 14526 | 10.50% |
| Mel.168.Rep.1 | chr2 | 74682104 | 74682104 | G | A | 6636 | 3.89% |
| Mel.168.Rep.1 | chr3 | 16306504 | 16306504 | C | T | 6175 | 16.96% |
| Mel.168.Rep.1 | chr3 | 16306505 | 16306505 | C | T | 6179 | 7.74% |
| Mel.168.Rep.1 | chr5 | 26885701 | 26885701 | C | T | 5608 | 5.44% |
| Mel.168.Rep.1 | chr5 | 150080667 | 150080667 | C | T | 13964 | 5.82% |
| Mel.168.Rep.1 | chr7 | 56174183 | 56174183 | G | A | 6529 | 33.76% |
| Mel.168.Rep.1 | chr10 | 7830002 | 7830002 | G | A | 7518 | 4.27% |
| Mel.168.Rep.1 | chr10 | 127512084 | 127512084 | G | A | 3520 | 9.69% |
| Mel.168.Rep.1 | chr11 | 46958261 | 46958261 | C | T | 8251 | 22.84% |
| Mel.168.Rep.1 | chr11 | 46958262 | 46958262 | C | T | 8259 | 22.91% |
| Mel.168.Rep.1 | chr13 | 41345346 | 41345346 | C | T | 10330 | 8.84% |
| Mel.168.Rep.1 | chr17 | 30771480 | 30771480 | C | T | 5332 | 23.57% |
| Mel.168.Rep.1 | chr19 | 13885241 | 13885241 | C | T | 6558 | 4.42% |
| Mel.168.Rep.1 | chr19 | 17970682 | 17970682 | C | T | 11383 | 8.91% |
| Mel.168.Rep.1 | chr19 | 48248748 | 48248748 | G | A | 5847 | 10.28% |
| Mel.168.Rep.2 | chr1 | 153963227 | 153963227 | C | T | 3994 | 31.05% |
| Mel.168.Rep.2 | chr1 | 155904250 | 155904250 | C | T | 2359 | 31.07% |
| Mel.168.Rep.2 | chr2 | 32390905 | 32390905 | C | T | 3157 | 12.04% |
| Mel.168.Rep.2 | chr3 | 16306504 | 16306504 | C | T | 4714 | 14.81% |
| Mel.168.Rep.2 | chr3 | 16306505 | 16306505 | C | T | 4720 | 14.33% |
| Mel.168.Rep.2 | chr7 | 56174183 | 56174183 | G | A | 2171 | 20.09% |
| Mel.168.Rep.2 | chr10 | 7830002 | 7830002 | G | A | 280 | 16.79% |
| Mel.168.Rep.2 | chr11 | 46958261 | 46958261 | C | T | 3049 | 6.36% |
| Mel.168.Rep.2 | chr11 | 46958262 | 46958262 | C | T | 3056 | 6.19% |
| Mel.168.Rep.2 | chr13 | 41345346 | 41345346 | C | T | 2835 | 9.28% |
| Mel.168.Rep.2 | chr17 | 30771480 | 30771480 | C | T | 1095 | 86.67% |
| Mel.168.Rep.2 | chr19 | 17970682 | 17970682 | C | T | 2430 | 20.12% |
| Mel.168.Rep.2 | chr19 | 48248748 | 48248748 | G | A | 2285 | 14.32% |
| Mel.169.Rep.1 | chr1 | 25559063 | 25559063 | C | T | 2668 | 99.36% |
| Mel.169.Rep.1 | chr1 | 155904250 | 155904250 | C | T | 4116 | 70.26% |
| Mel.169.Rep.1 | chr3 | 16306505 | 16306505 | C | T | 2990 | 90.30% |
| Mel.169.Rep.1 | chr7 | 56174183 | 56174183 | G | A | 3563 | 65.67% |
| Mel.169.Rep.1 | chr9 | 130700157 | 130700157 | C | T | 2209 | 5.12% |
| Mel.169.Rep.1 | chr11 | 62414150 | 62414150 | C | T | 1110 | 98.82% |
| Mel.169.Rep.2 | chr1 | 25559063 | 25559063 | C | T | 5401 | 61.64% |
| Mel.169.Rep.2 | chr1 | 155904250 | 155904250 | C | T | 6952 | 82.61% |
| Mel.169.Rep.2 | chr3 | 16306505 | 16306505 | C | T | 3665 | 47.04% |
| Mel.169.Rep.2 | chr7 | 56174183 | 56174183 | G | A | 5294 | 4.17% |
| Mel.169.Rep.2 | chr9 | 130700157 | 130700157 | C | T | 2288 | 99.65% |
| Mel.169.Rep.2 | chr11 | 62414150 | 62414150 | C | T | 2352 | 99.45% |
| Mel.169.Rep.2 | chr19 | 17970682 | 17970682 | C | T | 2323 | 53.81% |
| Mel.169.Rep.2 | chr19 | 38202422 | 38202422 | G | A | 1905 | 98.16% |
| Mel.169.Rep.2 | chr22 | 43011001 | 43011001 | G | A | 5014 | 90.63% |
| Mel.169.Rep.2 | chr22 | 43011002 | 43011002 | G | A | 4951 | 90.18% |
| Mel.169.Rep.3 | chr1 | 25559063 | 25559063 | C | T | 6714 | 26.75% |
| Mel.169.Rep.3 | chr1 | 155904250 | 155904250 | C | T | 6391 | 67.11% |
| Mel.169.Rep.3 | chr3 | 16306505 | 16306505 | C | T | 4872 | 66.85% |
| Mel.169.Rep.3 | chr7 | 39605969 | 39605969 | G | A | 6904 | 46.55% |
| Mel.169.Rep.3 | chr7 | 39605970 | 39605970 | G | A | 6879 | 46.61% |
| Mel.169.Rep.3 | chr7 | 56174183 | 56174183 | G | A | 5504 | 42.97% |
| Mel.169.Rep.3 | chr9 | 130700157 | 130700157 | C | T | 3592 | 49.08% |
| Mel.169.Rep.3 | chr11 | 62414150 | 62414150 | C | T | 189 | 87.30% |
| Mel.169.Rep.3 | chr13 | 41345346 | 41345346 | C | T | 4305 | 44.96% |
| Mel.169.Rep.3 | chr19 | 4247060 | 4247060 | G | A | 3158 | 3.48% |
| Mel.169.Rep.3 | chr19 | 17970682 | 17970682 | C | T | 4952 | 23.24% |
| Mel.169.Rep.3 | chr19 | 38202422 | 38202422 | G | A | 8588 | 13.90% |
| Mel.169.Rep.3 | chr22 | 43011001 | 43011001 | G | A | 6399 | 48.34% |
| Mel.169.Rep.3 | chr22 | 43011002 | 43011002 | G | A | 6346 | 47.90% |
| Mel.169.Rep.4 | chr1 | 25559063 | 25559063 | C | T | 5673 | 27.29% |
| Mel.169.Rep.4 | chr1 | 153276253 | 153276253 | G | A | 7428 | 3.68% |
| Mel.169.Rep.4 | chr1 | 155904250 | 155904250 | C | T | 5689 | 71.83% |
| Mel.169.Rep.4 | chr3 | 16306505 | 16306505 | C | T | 4567 | 80.29% |
| Mel.169.Rep.4 | chr7 | 39605969 | 39605969 | G | A | 5919 | 44.26% |
| Mel.169.Rep.4 | chr7 | 39605970 | 39605970 | G | A | 5911 | 44.61% |
| Mel.169.Rep.4 | chr7 | 56174183 | 56174183 | G | A | 4887 | 34.44% |
| Mel.169.Rep.4 | chr9 | 130700157 | 130700157 | C | T | 2917 | 39.09% |
| Mel.169.Rep.4 | chr11 | 62414150 | 62414150 | C | T | 192 | 90.10% |
| Mel.169.Rep.4 | chr13 | 41345346 | 41345346 | C | T | 4138 | 35.49% |
| Mel.169.Rep.4 | chr19 | 17970682 | 17970682 | C | T | 4472 | 53.06% |
| Mel.169.Rep.4 | chr20 | 34129792 | 34129792 | G | A | 3328 | 4.03% |
| Mel.169.Rep.4 | chr22 | 43011001 | 43011001 | G | A | 5430 | 59.96% |
| Mel.169.Rep.4 | chr22 | 43011002 | 43011002 | G | A | 5414 | 59.81% |
| Mel.170.Rep.1 | chr1 | 25559063 | 25559063 | C | T | 8600 | 11.55% |
| Mel.170.Rep.1 | chr1 | 25559064 | 25559064 | C | T | 8615 | 11.60% |
| Mel.170.Rep.1 | chr3 | 124449246 | 124449246 | C | T | 5116 | 14.13% |
| Mel.170.Rep.1 | chr4 | 152020703 | 152020703 | G | A | 8928 | 38.12% |
| Mel.170.Rep.1 | chr7 | 140453136 | 140453136 | A | T | 4187 | 40.15% |
| Mel.170.Rep.1 | chr8 | 30601668 | 30601668 | G | A | 5752 | 28.22% |
| Mel.170.Rep.1 | chr8 | 30601669 | 30601669 | G | A | 5746 | 28.25% |
| Mel.170.Rep.1 | chr8 | 125551345 | 125551345 | C | T | 435 | 20.69% |
| Mel.170.Rep.1 | chr17 | 30771480 | 30771480 | C | T | 3515 | 41.25% |
| Mel.170.Rep.1 | chr19 | 4247060 | 4247060 | G | A | 4049 | 20.42% |
| Mel.170.Rep.1 | chr22 | 43010992 | 43010992 | G | A | 13157 | 44.30% |
| Mel.170.Rep.2 | chr4 | 152020703 | 152020703 | G | A | 5053 | 12.86% |
| Mel.170.Rep.2 | chr7 | 140453136 | 140453136 | A | T | 7322 | 60.30% |
| Mel.170.Rep.2 | chr8 | 30601668 | 30601668 | G | A | 6666 | 23.75% |
| Mel.170.Rep.2 | chr8 | 30601669 | 30601669 | G | A | 6667 | 23.82% |
| Mel.170.Rep.2 | chr17 | 30771480 | 30771480 | C | T | 2112 | 36.74% |
| Mel.170.Rep.2 | chr19 | 4247060 | 4247060 | G | A | 3245 | 58.48% |
| Mel.170.Rep.2 | chr22 | 43010992 | 43010992 | G | A | 4011 | 20.64% |
| Mel.170.Rep.3 | chr4 | 152020703 | 152020703 | G | A | 8028 | 40.91% |
| Mel.170.Rep.3 | chr7 | 140453136 | 140453136 | A | T | 10441 | 47.25% |
| Mel.170.Rep.3 | chr8 | 30601668 | 30601668 | G | A | 9745 | 3.85% |
| Mel.170.Rep.3 | chr8 | 30601669 | 30601669 | G | A | 9734 | 3.85% |
| Mel.170.Rep.3 | chr17 | 30771480 | 30771480 | C | T | 5093 | 51.39% |
| Mel.170.Rep.3 | chr19 | 4247060 | 4247060 | G | A | 4794 | 43.97% |
| Mel.170.Rep.3 | chr22 | 43010992 | 43010992 | G | A | 6656 | 58.11% |
