# Supplementary material for: Clustered somatic mutations are frequent in transcription factor binding motifs within proximal promoter regions in melanoma and other cutaneous malignancies
Source: Oncotarget. 2016 Sep 7;7(41):66569–85. doi: 10.18632/oncotarget.11892 (PMC5341821; doi:10.18632/oncotarget.11892)
Supplement: Supplementary file 6 [file oncotarget-07-66569-s006.docx]

**Table S5. The frequency and percentage of hotspots in each of the putative chromatin states, for both melanocyte foreskin cell lines (E059 and E061), with enhancer states merged.**

| **Chromatin state** | **Frequency in E059 melanocyte line** | **Percentage in E059 melanocyte line** | **Frequency in E061 melanocyte line** | **Percentage in E061 melanocyte line** |
| --- | --- | --- | --- | --- |
| 1 Active TSS | 385 | 4.56 | 355 | 4.21 |
| 10 Bivalent/poised TSS | 7 | 0.08 | 23 | 0.27 |
| 11 Flanking bivalent TSS/Enh | 1 | 0.01 | 6 | 0.07 |
| ALL ENHANCERS | 75 | 0.89 | 114 | 1.35 |
| 13 Repressed Polycomb | 209 | 2.48 | 193 | 2.29 |
| 14 Weak repressed Polycomb | 1392 | 16.5 | 1752 | 20.77 |
| 15 Quiescent/low | 4937 | 58.54 | 4023 | 47.7 |
| 2 Flanking active TSS | 9 | 0.11 | 20 | 0.24 |
| 3 Transcr. at gene 5 prime and 3 prime | 3 | 0.04 | 3 | 0.04 |
| 4 Strong transcription | 87 | 1.03 | 215 | 2.55 |
| 5 Weak transcription | 670 | 7.94 | 646 | 7.66 |
| 8 ZNF genes + repeats | 41 | 0.49 | 37 | 0.44 |
| 9 Heterochromatin | 563 | 6.68 | 997 | 11.82 |
| MANY | 55 | 0.65 | 50 | 0.59 |
