# Supplementary material for: Clustered somatic mutations are frequent in transcription factor binding motifs within proximal promoter regions in melanoma and other cutaneous malignancies
Source: Oncotarget. 2016 Sep 7;7(41):66569–85. doi: 10.18632/oncotarget.11892 (PMC5341821; doi:10.18632/oncotarget.11892)
Supplement: Supplementary file 7 [file oncotarget-07-66569-s007.docx]

**Table S6. Table of all hotspots detected by the unbiased, background-corrected genome-wide cluster analysis**

**LEGEND**

chrom: chromosome

start: start position of hotspot (1-index)

end: end position of hotspot

number of variants: total number of SNVs in the hotspot

number of distinct samples: number of samples with SNVs in the hotspot

rmsk repeats, rmsk repeat status: which repeatmasker annotated regions are overlapped by hotspot, and TRUE/FALSE flag

tf binding sites, tf binding site overlaps: the name and number respectively of transcription factor binding sites from ENCODE that overlap hotspot

dnase flanking: whether a DNase I hypersensitivity site is within 50bp of the hotspot

restricted subset: whether the hotspot was present in the initial analysis which specifically looked for proximal promoters

| chrom | start | end | number of variants | number of distinct samples | rmsk repeats | rmsk repeat status | tf binding sites | tf binding site overlaps | uniq mappable 20 | dnase flanking | restricted subset |
| --- | --- | --- | --- | --- | --- | --- | --- | --- | --- | --- | --- |
| chr1 | 18528210 | 18528254 | 4 | 3 | NA | FALSE | NA | 0 | 1 | FALSE | FALSE |
| chr1 | 18564448 | 18564488 | 4 | 3 | Charlie4z | TRUE | NA | 0 | 1 | TRUE | FALSE |
| chr1 | 18581984 | 18582035 | 4 | 4 | NA | FALSE | NA | 0 | 1 | FALSE | FALSE |
| chr1 | 18598339 | 18598386 | 5 | 3 | NA | FALSE | NA | 0 | 1 | FALSE | FALSE |
| chr1 | 18757011 | 18757041 | 4 | 3 | LTR48B | TRUE | NA | 0 | 1 | FALSE | FALSE |
| chr1 | 18931259 | 18931307 | 6 | 4 | NA | FALSE | NA | 0 | 1 | FALSE | FALSE |
| chr1 | 19172028 | 19172066 | 4 | 3 | ERV3-16A3 I-int | TRUE | NA | 0 | 1 | FALSE | FALSE |
| chr1 | 19252313 | 19252370 | 8 | 6 | (TGGA)n | TRUE | NA | 0 | NA | FALSE | FALSE |
| chr1 | 20028476 | 20028531 | 4 | 2 | AluY | TRUE | NA | 0 | NA | FALSE | FALSE |
| chr1 | 20370295 | 20370295 | 4 | 4 | HERVH-int | TRUE | NA | 0 | NA | FALSE | FALSE |
| chr1 | 20641846 | 20641894 | 4 | 4 | NA | FALSE | NA | 0 | 1 | TRUE | FALSE |
| chr1 | 21738832 | 21738847 | 5 | 3 | NA | FALSE | NA | 0 | 1 | TRUE | FALSE |
| chr1 | 21794195 | 21794202 | 5 | 4 | NA | FALSE | NA | 0 | 1 | FALSE | FALSE |
| chr1 | 22162972 | 22162991 | 9 | 6 | AluSq2 | TRUE | NA | 0 | NA | FALSE | FALSE |
| chr1 | 22309965 | 22310017 | 4 | 2 | AluJo | TRUE | NA | 0 | 1 | FALSE | FALSE |
| chr1 | 22319191 | 22319207 | 4 | 4 | AluJb | TRUE | NA | 0 | NA | FALSE | FALSE |
| chr1 | 22332318 | 22332332 | 5 | 2 | NA | FALSE | NA | 0 | 1 | TRUE | FALSE |
| chr1 | 22664999 | 22665053 | 4 | 3 | NA | FALSE | NA | 0 | 1 | TRUE | FALSE |
| chr1 | 22804645 | 22804646 | 4 | 2 | MIR | TRUE | NA | 0 | 1 | FALSE | FALSE |
| chr1 | 23005872 | 23005911 | 4 | 2 | (CA)n | TRUE | SUZ12 | 1 | 1 | FALSE | FALSE |
| chr1 | 23678982 | 23679021 | 4 | 3 | NA | FALSE | NA | 0 | 1 | TRUE | FALSE |
| chr1 | 23999061 | 23999117 | 4 | 4 | MANY | TRUE | NA | 0 | 1 | FALSE | FALSE |
| chr1 | 24592604 | 24592688 | 4 | 4 | (TGGA)n | TRUE | NA | 0 | 1 | FALSE | FALSE |
| chr1 | 25559038 | 25559080 | 9 | 7 | NA | FALSE | MANY | 35 | 1 | TRUE | TRUE |
| chr1 | 25930479 | 25930541 | 8 | 6 | SVA D | TRUE | NA | 0 | NA | FALSE | FALSE |
| chr1 | 26075920 | 26076043 | 6 | 5 | MIR3 | TRUE | NA | 0 | 1 | TRUE | FALSE |
| chr1 | 26504882 | 26504919 | 4 | 2 | NA | FALSE | MANY | 4 | 1 | TRUE | TRUE |
| chr1 | 26671956 | 26672014 | 4 | 2 | NA | FALSE | NA | 0 | 1 | TRUE | FALSE |
| chr1 | 27130027 | 27130064 | 4 | 3 | SVA D | TRUE | NA | 0 | 1 | FALSE | FALSE |
| chr1 | 27952385 | 27952423 | 7 | 4 | AluSg4 | TRUE | NA | 0 | NA | FALSE | FALSE |
| chr1 | 28585892 | 28585922 | 10 | 6 | NA | FALSE | MANY | 35 | 1 | TRUE | TRUE |
| chr1 | 28934749 | 28934751 | 4 | 2 | AluY | TRUE | NA | 0 | NA | FALSE | FALSE |
| chr1 | 30101025 | 30101037 | 4 | 3 | MLT1J2 | TRUE | NA | 0 | 1 | TRUE | FALSE |
| chr1 | 30312317 | 30312319 | 4 | 2 | NA | FALSE | NA | 0 | NA | FALSE | FALSE |
| chr1 | 31012535 | 31012547 | 4 | 2 | L2b | TRUE | NA | 0 | 1 | TRUE | FALSE |
| chr1 | 31191318 | 31191352 | 4 | 3 | C-rich | TRUE | MANY | 2 | 1 | TRUE | FALSE |
| chr1 | 31334334 | 31334390 | 4 | 3 | L2c | TRUE | NA | 0 | 1 | FALSE | FALSE |
| chr1 | 31899287 | 31899288 | 4 | 2 | NA | FALSE | JunD | 1 | 1 | TRUE | FALSE |
| chr1 | 32110500 | 32110535 | 4 | 4 | NA | FALSE | MANY | 20 | 1 | TRUE | TRUE |
| chr1 | 32254208 | 32254209 | 4 | 3 | NA | FALSE | MANY | 25 | 1 | TRUE | TRUE |
| chr1 | 32898867 | 32898961 | 5 | 5 | HERVH-int | TRUE | NA | 0 | NA | FALSE | FALSE |
| chr1 | 32933461 | 32933510 | 4 | 3 | AluSx1 | TRUE | NA | 0 | NA | FALSE | FALSE |
| chr1 | 33529838 | 33529873 | 4 | 4 | LTR5 Hs | TRUE | MANY | 2 | NA | FALSE | FALSE |
| chr1 | 34995316 | 34995330 | 4 | 3 | L2c | TRUE | NA | 0 | 1 | FALSE | FALSE |
| chr1 | 35038135 | 35038157 | 4 | 3 | NA | FALSE | NA | 0 | 1 | TRUE | FALSE |
| chr1 | 35080986 | 35081008 | 4 | 3 | NA | FALSE | NA | 0 | 1 | FALSE | FALSE |
| chr1 | 35658768 | 35658803 | 4 | 3 | NA | FALSE | MANY | 43 | 1 | TRUE | TRUE |
| chr1 | 35875290 | 35875337 | 6 | 4 | AluSg | TRUE | NA | 0 | NA | FALSE | FALSE |
| chr1 | 36400471 | 36400490 | 5 | 3 | AluSx | TRUE | NA | 0 | NA | FALSE | FALSE |
| chr1 | 36709576 | 36709625 | 4 | 3 | SVA D | TRUE | NA | 0 | NA | FALSE | FALSE |
| chr1 | 37054738 | 37054789 | 5 | 4 | NA | FALSE | NA | 0 | 1 | TRUE | FALSE |
| chr1 | 37344412 | 37344450 | 4 | 3 | MIRb | TRUE | NA | 0 | 1 | TRUE | FALSE |
| chr1 | 38051497 | 38051503 | 4 | 3 | L1ME4a | TRUE | NA | 0 | 1 | FALSE | FALSE |
| chr1 | 38206127 | 38206159 | 4 | 2 | AluY | TRUE | NA | 0 | NA | FALSE | FALSE |
| chr1 | 38478321 | 38478323 | 6 | 6 | NA | FALSE | MANY | 45 | 1 | TRUE | TRUE |
| chr1 | 38765223 | 38765257 | 4 | 4 | NA | FALSE | NA | 0 | 1 | TRUE | FALSE |
| chr1 | 38811707 | 38811723 | 4 | 3 | NA | FALSE | NA | 0 | 1 | TRUE | FALSE |
| chr1 | 38815267 | 38815331 | 6 | 5 | MIR | TRUE | NA | 0 | 1 | TRUE | FALSE |
| chr1 | 38835259 | 38835297 | 4 | 3 | NA | FALSE | NA | 0 | 1 | FALSE | FALSE |
| chr1 | 39094109 | 39094112 | 5 | 5 | MIR | TRUE | NA | 0 | 1 | FALSE | FALSE |
| chr1 | 39095661 | 39095661 | 4 | 4 | L1PA6 | TRUE | NA | 0 | NA | FALSE | FALSE |
| chr1 | 39095717 | 39095717 | 5 | 5 | L1PA6 | TRUE | NA | 0 | NA | FALSE | FALSE |
| chr1 | 39651883 | 39651891 | 4 | 3 | MANY | TRUE | ZZZ3 | 1 | NA | FALSE | FALSE |
| chr1 | 40840293 | 40840297 | 4 | 4 | (TG)n | TRUE | MANY | 20 | NA | TRUE | FALSE |
| chr1 | 40937530 | 40937575 | 4 | 2 | LTR5A | TRUE | NA | 0 | NA | FALSE | FALSE |
| chr1 | 41023797 | 41023854 | 4 | 4 | LTR12C | TRUE | NA | 0 | NA | FALSE | FALSE |
| chr1 | 41051124 | 41051164 | 4 | 4 | MER5A | TRUE | NA | 0 | 1 | FALSE | FALSE |
| chr1 | 41057009 | 41057050 | 4 | 3 | MANY | TRUE | NA | 0 | 1 | FALSE | FALSE |
| chr1 | 41131377 | 41131452 | 4 | 3 | GC rich | TRUE | MANY | 10 | 1 | TRUE | TRUE |
| chr1 | 41362295 | 41362372 | 4 | 4 | NA | FALSE | HNF4A (H-171) | 1 | 1 | TRUE | FALSE |
| chr1 | 41394251 | 41394329 | 4 | 3 | L2b | TRUE | NA | 0 | 1 | FALSE | FALSE |
| chr1 | 41856644 | 41856683 | 4 | 3 | NA | FALSE | NA | 0 | 1 | FALSE | FALSE |
| chr1 | 42027419 | 42027419 | 8 | 8 | AluJb | TRUE | NA | 0 | NA | FALSE | FALSE |
| chr1 | 42626318 | 42626374 | 4 | 3 | L1MA8 | TRUE | SETDB1 | 1 | 1 | FALSE | FALSE |
| chr1 | 42825656 | 42825698 | 4 | 3 | NA | FALSE | NA | 0 | 1 | TRUE | FALSE |
| chr1 | 43824527 | 43824562 | 10 | 8 | NA | FALSE | MANY | 29 | 1 | TRUE | TRUE |
| chr1 | 44337301 | 44337327 | 4 | 4 | (GGATG)n | TRUE | NA | 0 | 1 | TRUE | FALSE |
| chr1 | 44435499 | 44435517 | 4 | 4 | NA | FALSE | MANY | 18 | 1 | TRUE | TRUE |
| chr1 | 45401264 | 45401318 | 4 | 2 | AluSz | TRUE | NA | 0 | NA | FALSE | FALSE |
| chr1 | 45538729 | 45538740 | 4 | 3 | L1MA2 | TRUE | NA | 0 | NA | FALSE | FALSE |
| chr1 | 45560060 | 45560133 | 4 | 4 | MANY | TRUE | NA | 0 | 1 | FALSE | FALSE |
| chr1 | 45769345 | 45769397 | 4 | 2 | NA | FALSE | MANY | 9 | 1 | TRUE | TRUE |
| chr1 | 46190722 | 46190769 | 4 | 3 | SVA E | TRUE | NA | 0 | NA | FALSE | FALSE |
| chr1 | 46205563 | 46205620 | 4 | 4 | SVA D | TRUE | NA | 0 | NA | TRUE | FALSE |
| chr1 | 46940775 | 46940818 | 4 | 3 | NA | FALSE | NA | 0 | 1 | TRUE | FALSE |
| chr1 | 47184849 | 47184865 | 4 | 3 | NA | FALSE | MANY | 37 | 1 | TRUE | TRUE |
| chr1 | 47379817 | 47379837 | 4 | 4 | L1PB2 | TRUE | NA | 0 | 1 | FALSE | FALSE |
| chr1 | 47390216 | 47390225 | 4 | 4 | L1PA7 | TRUE | NA | 0 | NA | FALSE | FALSE |
| chr1 | 47615371 | 47615371 | 4 | 4 | MLT1D | TRUE | NA | 0 | 1 | FALSE | FALSE |
| chr1 | 47751994 | 47752011 | 11 | 7 | NA | FALSE | NA | 0 | 1 | FALSE | FALSE |
| chr1 | 47780766 | 47780773 | 4 | 2 | AluY | TRUE | NA | 0 | NA | FALSE | FALSE |
| chr1 | 48312754 | 48312785 | 4 | 4 | NA | FALSE | NA | 0 | 1 | TRUE | FALSE |
| chr1 | 48459732 | 48459765 | 4 | 3 | NA | FALSE | NA | 0 | 1 | TRUE | FALSE |
| chr1 | 49014399 | 49014410 | 4 | 2 | NA | FALSE | NA | 0 | 1 | FALSE | FALSE |
| chr1 | 50004937 | 50004956 | 4 | 3 | HAL1b | TRUE | NA | 0 | 1 | FALSE | FALSE |
| chr1 | 50030779 | 50030782 | 4 | 4 | L1PA4 | TRUE | NA | 0 | 1 | FALSE | FALSE |
| chr1 | 50592878 | 50592912 | 4 | 3 | L2c | TRUE | GATA-2 | 1 | 1 | TRUE | FALSE |
| chr1 | 50747624 | 50747685 | 9 | 5 | (TAGA)n | TRUE | NA | 0 | NA | FALSE | FALSE |
| chr1 | 50777784 | 50777803 | 4 | 2 | MIRb | TRUE | NA | 0 | 1 | FALSE | FALSE |
| chr1 | 50786033 | 50786055 | 6 | 3 | L1P1 | TRUE | NA | 0 | 1 | FALSE | FALSE |
| chr1 | 50847502 | 50847552 | 6 | 3 | SVA D | TRUE | NA | 0 | NA | FALSE | FALSE |
| chr1 | 52396152 | 52396266 | 4 | 3 | MIR | TRUE | NA | 0 | 1 | FALSE | FALSE |
| chr1 | 52427241 | 52427285 | 4 | 2 | MIRc | TRUE | NA | 0 | 1 | FALSE | FALSE |
| chr1 | 53335525 | 53335560 | 5 | 3 | SVA D | TRUE | NA | 0 | 1 | FALSE | FALSE |
| chr1 | 53795917 | 53795927 | 7 | 4 | AluY | TRUE | NA | 0 | NA | FALSE | FALSE |
| chr1 | 54276646 | 54276684 | 4 | 3 | L1PB1 | TRUE | NA | 0 | 1 | FALSE | FALSE |
| chr1 | 54355505 | 54355512 | 4 | 3 | NA | FALSE | MANY | 21 | 1 | TRUE | TRUE |
| chr1 | 54519105 | 54519153 | 4 | 4 | NA | FALSE | MANY | 32 | 1 | TRUE | TRUE |
| chr1 | 55317149 | 55317202 | 4 | 4 | NA | FALSE | NA | 0 | 1 | TRUE | FALSE |
| chr1 | 56333341 | 56333362 | 4 | 3 | NA | FALSE | NA | 0 | 1 | TRUE | FALSE |
| chr1 | 56520249 | 56520270 | 4 | 3 | MIRb | TRUE | NA | 0 | 1 | FALSE | FALSE |
| chr1 | 57608303 | 57608309 | 5 | 4 | (CA)n | TRUE | NA | 0 | NA | TRUE | FALSE |
| chr1 | 57618168 | 57618187 | 4 | 4 | NA | FALSE | NA | 0 | 1 | FALSE | FALSE |
| chr1 | 59457062 | 59457089 | 4 | 4 | HERV16-int | TRUE | NA | 0 | 1 | FALSE | FALSE |
| chr1 | 59460103 | 59460107 | 4 | 2 | AluY | TRUE | NA | 0 | NA | FALSE | FALSE |
| chr1 | 59738676 | 59738714 | 4 | 4 | L1ME3F | TRUE | NA | 0 | 1 | FALSE | FALSE |
| chr1 | 61733143 | 61733148 | 4 | 3 | AluSx | TRUE | NA | 0 | NA | FALSE | FALSE |
| chr1 | 61858921 | 61858942 | 4 | 3 | (TC)n | TRUE | NA | 0 | NA | FALSE | FALSE |
| chr1 | 62383264 | 62383298 | 4 | 3 | L1M5 | TRUE | NA | 0 | 1 | FALSE | FALSE |
| chr1 | 62411276 | 62411326 | 6 | 3 | L1PA5 | TRUE | NA | 0 | NA | FALSE | FALSE |
| chr1 | 62604109 | 62604167 | 10 | 6 | AluSx | TRUE | NA | 0 | NA | FALSE | FALSE |
| chr1 | 62644660 | 62644665 | 4 | 3 | MANY | TRUE | NA | 0 | NA | FALSE | FALSE |
| chr1 | 63833262 | 63833266 | 4 | 3 | NA | FALSE | MANY | 17 | 1 | TRUE | TRUE |
| chr1 | 63938233 | 63938294 | 10 | 7 | SVA D | TRUE | PAX5-C20 | 1 | NA | FALSE | FALSE |
| chr1 | 64446554 | 64446601 | 4 | 3 | NA | FALSE | NA | 0 | 1 | TRUE | FALSE |
| chr1 | 64882472 | 64882514 | 8 | 7 | SVA D | TRUE | NA | 0 | NA | TRUE | FALSE |
| chr1 | 65484164 | 65484205 | 5 | 4 | MANY | TRUE | NA | 0 | 1 | TRUE | FALSE |
| chr1 | 65596988 | 65597050 | 4 | 3 | NA | FALSE | NA | 0 | 1 | TRUE | FALSE |
| chr1 | 65956978 | 65957029 | 4 | 3 | NA | FALSE | NA | 0 | 1 | FALSE | FALSE |
| chr1 | 66008611 | 66008675 | 10 | 6 | L1PA7 | TRUE | NA | 0 | NA | FALSE | FALSE |
| chr1 | 67288567 | 67288607 | 4 | 2 | MANY | TRUE | NA | 0 | 1 | FALSE | FALSE |
| chr1 | 67585609 | 67585653 | 4 | 3 | MLT1A0-int | TRUE | NA | 0 | 1 | FALSE | FALSE |
| chr1 | 67857542 | 67857595 | 5 | 4 | AluY | TRUE | NA | 0 | NA | FALSE | FALSE |
| chr1 | 69437110 | 69437137 | 4 | 4 | MLT1H | TRUE | NA | 0 | 1 | FALSE | FALSE |
| chr1 | 69496133 | 69496163 | 4 | 3 | L1MA4A | TRUE | SETDB1 | 1 | 1 | FALSE | FALSE |
| chr1 | 70757586 | 70757621 | 4 | 3 | AluSx1 | TRUE | NA | 0 | NA | FALSE | FALSE |
| chr1 | 71189892 | 71189921 | 4 | 4 | L1PA11 | TRUE | NA | 0 | 1 | FALSE | FALSE |
| chr1 | 71239762 | 71239790 | 4 | 3 | L1PA14 | TRUE | NA | 0 | 1 | FALSE | FALSE |
| chr1 | 71721282 | 71721300 | 4 | 2 | NA | FALSE | NA | 0 | 1 | FALSE | FALSE |
| chr1 | 72067984 | 72067993 | 4 | 4 | AluYa5 | TRUE | NA | 0 | NA | FALSE | FALSE |
| chr1 | 72092355 | 72092368 | 4 | 2 | L1PA4 | TRUE | NA | 0 | NA | FALSE | FALSE |
| chr1 | 72526058 | 72526088 | 4 | 4 | NA | FALSE | NA | 0 | 1 | FALSE | FALSE |
| chr1 | 73364079 | 73364089 | 4 | 3 | L1MEg | TRUE | NA | 0 | 1 | FALSE | FALSE |
| chr1 | 73377401 | 73377403 | 4 | 4 | NA | FALSE | NA | 0 | 1 | FALSE | FALSE |
| chr1 | 73401498 | 73401509 | 4 | 3 | NA | FALSE | NA | 0 | 1 | FALSE | FALSE |
| chr1 | 73649466 | 73649466 | 4 | 4 | LTR10F | TRUE | NA | 0 | NA | TRUE | FALSE |
| chr1 | 74132674 | 74132674 | 4 | 4 | THE1A | TRUE | NA | 0 | NA | FALSE | FALSE |
| chr1 | 74269794 | 74269807 | 4 | 3 | NA | FALSE | NA | 0 | 1 | FALSE | FALSE |
| chr1 | 74409680 | 74409685 | 4 | 3 | NA | FALSE | NA | 0 | 1 | FALSE | FALSE |
| chr1 | 75589336 | 75589340 | 4 | 3 | MANY | TRUE | NA | 0 | NA | FALSE | FALSE |
| chr1 | 75793642 | 75793664 | 4 | 3 | NA | FALSE | NA | 0 | 1 | TRUE | FALSE |
| chr1 | 75865413 | 75865413 | 4 | 4 | NA | FALSE | NA | 0 | 1 | FALSE | FALSE |
| chr1 | 76024480 | 76024508 | 4 | 4 | NA | FALSE | NA | 0 | 1 | TRUE | FALSE |
| chr1 | 76807342 | 76807386 | 4 | 3 | (TTTC)n | TRUE | NA | 0 | NA | FALSE | FALSE |
| chr1 | 77575511 | 77575550 | 5 | 5 | L1PA5 | TRUE | NA | 0 | NA | FALSE | FALSE |
| chr1 | 78696766 | 78696786 | 4 | 3 | Charlie1b | TRUE | NA | 0 | 1 | FALSE | FALSE |
| chr1 | 78733981 | 78733998 | 4 | 4 | NA | FALSE | NA | 0 | 1 | FALSE | FALSE |
| chr1 | 78788379 | 78788407 | 4 | 4 | NA | FALSE | NA | 0 | 1 | FALSE | FALSE |
| chr1 | 79324248 | 79324267 | 4 | 3 | NA | FALSE | NA | 0 | 1 | TRUE | FALSE |
| chr1 | 80109989 | 80110014 | 6 | 5 | LTR7 | TRUE | NA | 0 | 1 | TRUE | FALSE |
| chr1 | 80250999 | 80251017 | 4 | 4 | MIR | TRUE | NA | 0 | 1 | FALSE | FALSE |
| chr1 | 80444821 | 80444829 | 4 | 3 | NA | FALSE | NA | 0 | 1 | TRUE | FALSE |
| chr1 | 81221858 | 81221892 | 5 | 4 | L1ME1 | TRUE | MANY | 3 | 1 | FALSE | FALSE |
| chr1 | 81636760 | 81636788 | 4 | 4 | L1M2a | TRUE | NA | 0 | 1 | FALSE | FALSE |
| chr1 | 81801754 | 81801775 | 4 | 2 | NA | FALSE | NA | 0 | 1 | TRUE | FALSE |
| chr1 | 81868303 | 81868312 | 5 | 5 | NA | FALSE | NA | 0 | 1 | FALSE | FALSE |
| chr1 | 82154933 | 82154949 | 4 | 3 | NA | FALSE | NA | 0 | 1 | FALSE | FALSE |
| chr1 | 83065060 | 83065073 | 4 | 2 | NA | FALSE | NA | 0 | 1 | FALSE | FALSE |
| chr1 | 83205626 | 83205644 | 4 | 4 | NA | FALSE | NA | 0 | 1 | FALSE | FALSE |
| chr1 | 83602496 | 83602497 | 4 | 2 | L1PA15 | TRUE | NA | 0 | 1 | FALSE | FALSE |
| chr1 | 83714110 | 83714110 | 4 | 4 | NA | FALSE | NA | 0 | 1 | FALSE | FALSE |
| chr1 | 83786557 | 83786558 | 4 | 2 | L1ME3A | TRUE | NA | 0 | 1 | FALSE | FALSE |
| chr1 | 83895052 | 83895107 | 7 | 7 | NA | FALSE | NA | 0 | 1 | FALSE | FALSE |
| chr1 | 84074748 | 84074807 | 9 | 8 | AluSx3 | TRUE | NA | 0 | NA | FALSE | FALSE |
| chr1 | 85983695 | 85983699 | 5 | 4 | NA | FALSE | NA | 0 | 1 | FALSE | FALSE |
| chr1 | 87095914 | 87095958 | 6 | 6 | L1P1 | TRUE | NA | 0 | 1 | FALSE | FALSE |
| chr1 | 88358728 | 88358756 | 4 | 4 | MIRb | TRUE | NA | 0 | 1 | FALSE | FALSE |
| chr1 | 89516419 | 89516427 | 5 | 3 | L1M2b | TRUE | NA | 0 | 1 | TRUE | FALSE |
| chr1 | 89599036 | 89599069 | 7 | 4 | NA | FALSE | NA | 0 | 1 | FALSE | FALSE |
| chr1 | 89866569 | 89866628 | 4 | 4 | L1PA7 | TRUE | NA | 0 | 1 | FALSE | FALSE |
| chr1 | 91254222 | 91254262 | 4 | 3 | AluSc | TRUE | NA | 0 | 1 | FALSE | FALSE |
| chr1 | 92324548 | 92324565 | 5 | 2 | AluSz6 | TRUE | NA | 0 | NA | FALSE | FALSE |
| chr1 | 92528338 | 92528395 | 4 | 4 | MER106A | TRUE | NA | 0 | 1 | FALSE | FALSE |
| chr1 | 92848178 | 92848178 | 4 | 4 | L1PA4 | TRUE | NA | 0 | NA | FALSE | FALSE |
| chr1 | 93537863 | 93537935 | 4 | 4 | MSTA | TRUE | NA | 0 | 1 | TRUE | FALSE |
| chr1 | 93644759 | 93644834 | 5 | 3 | AluSx | TRUE | TFIIIC-110 | 1 | NA | FALSE | FALSE |
| chr1 | 96275816 | 96275828 | 4 | 4 | MER34A1 | TRUE | NA | 0 | 1 | TRUE | FALSE |
| chr1 | 96569065 | 96569085 | 4 | 3 | MIR | TRUE | NA | 0 | 1 | FALSE | FALSE |
| chr1 | 96739396 | 96739396 | 4 | 4 | L1MA9 | TRUE | NA | 0 | 1 | FALSE | FALSE |
| chr1 | 97545402 | 97545424 | 4 | 4 | (TCCC)n | TRUE | NA | 0 | 1 | FALSE | FALSE |
| chr1 | 97824513 | 97824515 | 4 | 4 | L1PA6 | TRUE | NA | 0 | NA | FALSE | FALSE |
| chr1 | 98136527 | 98136550 | 4 | 2 | NA | FALSE | NA | 0 | 1 | FALSE | FALSE |
| chr1 | 98261328 | 98261347 | 4 | 3 | NA | FALSE | NA | 0 | 1 | FALSE | FALSE |
| chr1 | 98280535 | 98280556 | 5 | 5 | A-rich | TRUE | NA | 0 | NA | FALSE | FALSE |
| chr1 | 100249469 | 100249552 | 4 | 3 | AluJo | TRUE | NA | 0 | 1 | FALSE | FALSE |
| chr1 | 100253631 | 100253653 | 4 | 3 | MANY | TRUE | NA | 0 | 1 | FALSE | FALSE |
| chr1 | 100322025 | 100322069 | 5 | 5 | SVA C | TRUE | NA | 0 | NA | FALSE | FALSE |
| chr1 | 100401588 | 100401703 | 6 | 5 | LTR27 | TRUE | NA | 0 | 1 | FALSE | FALSE |
| chr1 | 100598552 | 100598555 | 8 | 8 | NA | FALSE | MANY | 33 | 1 | TRUE | TRUE |
| chr1 | 101258165 | 101258228 | 4 | 4 | LTR33A | TRUE | NA | 0 | 1 | FALSE | FALSE |
| chr1 | 101778897 | 101778914 | 4 | 3 | NA | FALSE | NA | 0 | 1 | FALSE | FALSE |
| chr1 | 102087018 | 102087042 | 4 | 3 | AluSx | TRUE | NA | 0 | NA | FALSE | FALSE |
| chr1 | 102694262 | 102694275 | 4 | 4 | AluYf4 | TRUE | NA | 0 | NA | FALSE | FALSE |
| chr1 | 102715036 | 102715049 | 4 | 4 | NA | FALSE | NA | 0 | 1 | FALSE | FALSE |
| chr1 | 102880137 | 102880159 | 4 | 3 | MER21C | TRUE | NA | 0 | 1 | FALSE | FALSE |
| chr1 | 102906316 | 102906333 | 4 | 3 | THE1D | TRUE | NA | 0 | 1 | FALSE | FALSE |
| chr1 | 103121565 | 103121586 | 4 | 3 | L2a | TRUE | NA | 0 | 1 | TRUE | FALSE |
| chr1 | 103135912 | 103135934 | 4 | 3 | AluY | TRUE | NA | 0 | 1 | FALSE | FALSE |
| chr1 | 104136533 | 104136584 | 4 | 4 | MANY | TRUE | NA | 0 | 1 | FALSE | FALSE |
| chr1 | 104308520 | 104308563 | 6 | 5 | NA | FALSE | NA | 0 | 1 | FALSE | FALSE |
| chr1 | 104383280 | 104383311 | 4 | 3 | NA | FALSE | NA | 0 | 1 | FALSE | FALSE |
| chr1 | 104406718 | 104406754 | 6 | 5 | ALR/Alpha | TRUE | NA | 0 | 1 | FALSE | FALSE |
| chr1 | 104416338 | 104416366 | 4 | 4 | ALR/Alpha | TRUE | NA | 0 | 1 | FALSE | FALSE |
| chr1 | 104861335 | 104861338 | 4 | 4 | AT rich | TRUE | NA | 0 | NA | FALSE | FALSE |
| chr1 | 105130069 | 105130116 | 6 | 5 | L1PA7 | TRUE | NA | 0 | NA | FALSE | FALSE |
| chr1 | 106304259 | 106304277 | 4 | 4 | NA | FALSE | NA | 0 | 1 | TRUE | FALSE |
| chr1 | 106451849 | 106451849 | 4 | 4 | LTR1D | TRUE | NA | 0 | 1 | TRUE | FALSE |
| chr1 | 106776182 | 106776191 | 4 | 2 | MSTA-int | TRUE | NA | 0 | 1 | TRUE | FALSE |
| chr1 | 106899168 | 106899172 | 4 | 3 | L2a | TRUE | NA | 0 | 1 | FALSE | FALSE |
| chr1 | 107520983 | 107521003 | 4 | 4 | NA | FALSE | NA | 0 | 1 | FALSE | FALSE |
| chr1 | 108907279 | 108907290 | 4 | 2 | NA | FALSE | NA | 0 | 1 | TRUE | FALSE |
| chr1 | 109032268 | 109032417 | 5 | 5 | SVA F | TRUE | NA | 0 | NA | FALSE | FALSE |
| chr1 | 109217944 | 109218047 | 6 | 6 | SVA F | TRUE | NA | 0 | NA | FALSE | FALSE |
| chr1 | 109384968 | 109385018 | 4 | 2 | NA | FALSE | NA | 0 | 1 | FALSE | FALSE |
| chr1 | 109720154 | 109720158 | 7 | 4 | SVA D | TRUE | NA | 0 | NA | FALSE | FALSE |
| chr1 | 110252412 | 110252475 | 4 | 4 | NA | FALSE | NA | 0 | 1 | TRUE | FALSE |
| chr1 | 110254790 | 110254871 | 4 | 2 | NA | FALSE | NA | 0 | 1 | TRUE | FALSE |
| chr1 | 110443196 | 110443263 | 4 | 3 | MIRc | TRUE | CEBPB | 1 | 1 | TRUE | FALSE |
| chr1 | 111405502 | 111405514 | 4 | 4 | L1PA14 | TRUE | NA | 0 | 1 | FALSE | FALSE |
| chr1 | 111869481 | 111869515 | 4 | 4 | L1MA4A | TRUE | NA | 0 | 1 | FALSE | FALSE |
| chr1 | 112150437 | 112150478 | 5 | 4 | SVA D | TRUE | NA | 0 | NA | FALSE | FALSE |
| chr1 | 112718764 | 112718780 | 4 | 3 | MIRb | TRUE | NA | 0 | 1 | FALSE | FALSE |
| chr1 | 112729738 | 112729757 | 4 | 4 | NA | FALSE | NA | 0 | 1 | FALSE | FALSE |
| chr1 | 112807543 | 112807543 | 4 | 4 | NA | FALSE | NA | 0 | 1 | FALSE | FALSE |
| chr1 | 112816536 | 112816570 | 6 | 3 | AluSz | TRUE | NA | 0 | NA | FALSE | FALSE |
| chr1 | 112825874 | 112825886 | 4 | 3 | NA | FALSE | NA | 0 | 1 | FALSE | FALSE |
| chr1 | 113886273 | 113886317 | 4 | 3 | L1HS | TRUE | NA | 0 | 1 | FALSE | FALSE |
| chr1 | 114780530 | 114780556 | 4 | 4 | L2b | TRUE | NA | 0 | 1 | TRUE | FALSE |
| chr1 | 114889869 | 114889870 | 6 | 3 | NA | FALSE | NA | 0 | NA | TRUE | FALSE |
| chr1 | 115256528 | 115256529 | 8 | 7 | NA | FALSE | NA | 0 | 1 | TRUE | FALSE |
| chr1 | 115431986 | 115432005 | 4 | 3 | L1PA6 | TRUE | NA | 0 | NA | FALSE | FALSE |
| chr1 | 115457371 | 115457388 | 6 | 4 | NA | FALSE | NA | 0 | 1 | FALSE | FALSE |
| chr1 | 115540749 | 115540773 | 4 | 2 | MLT1J2 | TRUE | NA | 0 | 1 | FALSE | FALSE |
| chr1 | 115653601 | 115653621 | 4 | 3 | NA | FALSE | NA | 0 | 1 | FALSE | FALSE |
| chr1 | 115938685 | 115938695 | 4 | 3 | THE1A-int | TRUE | NA | 0 | 1 | FALSE | FALSE |
| chr1 | 117184658 | 117184725 | 6 | 6 | FRAM | TRUE | NA | 0 | 1 | TRUE | FALSE |
| chr1 | 117197520 | 117197563 | 5 | 4 | NA | FALSE | NA | 0 | 1 | FALSE | FALSE |
| chr1 | 117197873 | 117197900 | 4 | 3 | NA | FALSE | NA | 0 | 1 | FALSE | FALSE |
| chr1 | 117401007 | 117401058 | 4 | 2 | MANY | TRUE | NA | 0 | 1 | FALSE | FALSE |
| chr1 | 117732793 | 117732848 | 4 | 3 | L1MA9 | TRUE | NA | 0 | 1 | FALSE | FALSE |
| chr1 | 118431833 | 118431874 | 4 | 4 | L1MA8 | TRUE | NA | 0 | 1 | FALSE | FALSE |
| chr1 | 118855960 | 118855987 | 4 | 3 | L1PA5 | TRUE | NA | 0 | NA | FALSE | FALSE |
| chr1 | 118892396 | 118892440 | 7 | 4 | L1PA5 | TRUE | NA | 0 | NA | FALSE | FALSE |
| chr1 | 119203565 | 119203579 | 4 | 4 | NA | FALSE | NA | 0 | 1 | TRUE | FALSE |
| chr1 | 119360217 | 119360230 | 4 | 4 | NA | FALSE | NA | 0 | 1 | FALSE | FALSE |
| chr1 | 119611461 | 119611465 | 5 | 4 | MANY | TRUE | NA | 0 | NA | TRUE | FALSE |
| chr1 | 120023042 | 120023094 | 5 | 4 | (GGAA)n | TRUE | NA | 0 | NA | FALSE | FALSE |
| chr1 | 120030165 | 120030212 | 4 | 4 | LTR19-int | TRUE | NA | 0 | 1 | TRUE | FALSE |
| chr1 | 120093817 | 120093837 | 5 | 3 | NA | FALSE | NA | 0 | 1 | FALSE | FALSE |
| chr1 | 120153649 | 120153690 | 5 | 5 | NA | FALSE | NA | 0 | 1 | TRUE | FALSE |
| chr1 | 120387815 | 120387839 | 5 | 5 | NA | FALSE | NA | 0 | NA | FALSE | FALSE |
| chr1 | 120390367 | 120390420 | 5 | 4 | MER5A | TRUE | NA | 0 | 1 | TRUE | FALSE |
| chr1 | 120392867 | 120392890 | 7 | 4 | NA | FALSE | NA | 0 | 1 | FALSE | FALSE |
| chr1 | 120542578 | 120542595 | 8 | 5 | A-rich | TRUE | NA | 0 | NA | FALSE | FALSE |
| chr1 | 121137715 | 121137742 | 5 | 5 | NA | FALSE | MANY | 3 | 1 | TRUE | TRUE |
| chr1 | 121140512 | 121140523 | 4 | 4 | MER30B | TRUE | NA | 0 | NA | FALSE | FALSE |
| chr1 | 121142595 | 121142641 | 8 | 8 | (AAATG)n | TRUE | NA | 0 | NA | TRUE | FALSE |
| chr1 | 121351371 | 121351432 | 5 | 5 | CT-rich | TRUE | NA | 0 | 1 | TRUE | FALSE |
| chr1 | 142548081 | 142548107 | 4 | 4 | L1PA4 | TRUE | NA | 0 | NA | FALSE | FALSE |
| chr1 | 142554319 | 142554346 | 4 | 3 | NA | FALSE | NA | 0 | NA | FALSE | FALSE |
| chr1 | 142554440 | 142554481 | 6 | 5 | NA | FALSE | NA | 0 | 1 | FALSE | FALSE |
| chr1 | 142562781 | 142562806 | 6 | 6 | L1PA4 | TRUE | NA | 0 | NA | FALSE | FALSE |
| chr1 | 142574710 | 142574765 | 9 | 6 | AluY | TRUE | NA | 0 | NA | FALSE | FALSE |
| chr1 | 142582631 | 142582641 | 4 | 4 | L1MEf | TRUE | NA | 0 | 1 | FALSE | FALSE |
| chr1 | 142609513 | 142609540 | 4 | 4 | L1PA7 | TRUE | NA | 0 | NA | FALSE | FALSE |
| chr1 | 142616529 | 142616550 | 4 | 3 | L1M4 | TRUE | NA | 0 | 1 | TRUE | FALSE |
| chr1 | 142616719 | 142616731 | 4 | 3 | NA | FALSE | NA | 0 | 1 | FALSE | FALSE |
| chr1 | 142617575 | 142617593 | 4 | 3 | NA | FALSE | NA | 0 | 1 | TRUE | FALSE |
| chr1 | 142618339 | 142618358 | 4 | 4 | AluSg | TRUE | NA | 0 | NA | FALSE | FALSE |
| chr1 | 142647337 | 142647353 | 7 | 5 | (TG)n | TRUE | NA | 0 | 1 | FALSE | FALSE |
| chr1 | 142649391 | 142649408 | 4 | 4 | AluSx3 | TRUE | NA | 0 | NA | FALSE | FALSE |
| chr1 | 142662017 | 142662018 | 4 | 3 | NA | FALSE | NA | 0 | 1 | TRUE | FALSE |
| chr1 | 142701844 | 142701844 | 4 | 4 | L1MA5A | TRUE | NA | 0 | NA | FALSE | FALSE |
| chr1 | 142728834 | 142728852 | 7 | 7 | NA | FALSE | NA | 0 | NA | FALSE | FALSE |
| chr1 | 143122652 | 143122669 | 4 | 3 | NA | FALSE | NA | 0 | NA | FALSE | FALSE |
| chr1 | 143132718 | 143132727 | 4 | 3 | MER57-int | TRUE | NA | 0 | 1 | TRUE | FALSE |
| chr1 | 143141463 | 143141476 | 4 | 2 | NA | FALSE | NA | 0 | 1 | FALSE | FALSE |
| chr1 | 143145757 | 143145773 | 4 | 3 | AluSx | TRUE | NA | 0 | NA | FALSE | FALSE |
| chr1 | 143162941 | 143162943 | 4 | 3 | L1PA13 | TRUE | NA | 0 | NA | FALSE | FALSE |
| chr1 | 143412598 | 143412631 | 7 | 5 | MST-int | TRUE | NA | 0 | 1 | TRUE | FALSE |
| chr1 | 143414593 | 143414609 | 5 | 4 | MSTA-int | TRUE | NA | 0 | NA | FALSE | FALSE |
| chr1 | 143472957 | 143472983 | 4 | 3 | L1MA3 | TRUE | NA | 0 | 1 | FALSE | FALSE |
| chr1 | 143490415 | 143490416 | 4 | 4 | HERVK9-int | TRUE | NA | 0 | NA | FALSE | FALSE |
| chr1 | 143491311 | 143491338 | 5 | 5 | HERVK9-int | TRUE | NA | 0 | 1 | FALSE | FALSE |
| chr1 | 143502358 | 143502372 | 4 | 4 | L1MB1 | TRUE | NA | 0 | NA | FALSE | FALSE |
| chr1 | 143511765 | 143511767 | 4 | 2 | NA | FALSE | MANY | 3 | NA | FALSE | FALSE |
| chr1 | 143526824 | 143526849 | 6 | 6 | NA | FALSE | NA | 0 | 1 | TRUE | FALSE |
| chr1 | 143536565 | 143536575 | 5 | 5 | NA | FALSE | NA | 0 | NA | FALSE | FALSE |
| chr1 | 143536696 | 143536720 | 4 | 4 | AluSc | TRUE | TAF1 | 1 | NA | TRUE | FALSE |
| chr1 | 143880152 | 143880166 | 4 | 4 | AluSp | TRUE | NA | 0 | NA | FALSE | FALSE |
| chr1 | 143903447 | 143903502 | 5 | 4 | MANY | TRUE | NA | 0 | 1 | FALSE | FALSE |
| chr1 | 143941771 | 143941807 | 4 | 4 | NA | FALSE | NA | 0 | 1 | FALSE | FALSE |
| chr1 | 144083321 | 144083462 | 4 | 4 | (TTTC)n | TRUE | NA | 0 | NA | FALSE | FALSE |
| chr1 | 144531944 | 144531985 | 7 | 6 | AluY | TRUE | NA | 0 | NA | TRUE | FALSE |
| chr1 | 144586313 | 144586336 | 7 | 5 | LTR16A2 | TRUE | NA | 0 | 1 | FALSE | FALSE |
| chr1 | 144594307 | 144594372 | 4 | 3 | NA | FALSE | MANY | 6 | 1 | TRUE | TRUE |
| chr1 | 144706392 | 144706453 | 4 | 2 | L1PA7 | TRUE | NA | 0 | 1 | FALSE | FALSE |
| chr1 | 144812986 | 144813032 | 5 | 4 | MIR | TRUE | NA | 0 | 1 | FALSE | FALSE |
| chr1 | 144828307 | 144828352 | 5 | 5 | CT-rich | TRUE | NA | 0 | NA | FALSE | FALSE |
| chr1 | 144853455 | 144853461 | 6 | 4 | NA | FALSE | MANY | 2 | 1 | TRUE | FALSE |
| chr1 | 144861615 | 144861658 | 6 | 4 | NA | FALSE | NA | 0 | 1 | TRUE | FALSE |
| chr1 | 144861888 | 144861927 | 8 | 5 | NA | FALSE | NA | 0 | 1 | FALSE | FALSE |
| chr1 | 145241127 | 145241144 | 5 | 5 | NA | FALSE | NA | 0 | NA | TRUE | FALSE |
| chr1 | 145262772 | 145262820 | 4 | 4 | NA | FALSE | NA | 0 | 1 | TRUE | FALSE |
| chr1 | 145287967 | 145288007 | 4 | 3 | L1MB7 | TRUE | NA | 0 | 1 | TRUE | FALSE |
| chr1 | 145794979 | 145795103 | 6 | 5 | AluSx1 | TRUE | NA | 0 | 1 | FALSE | FALSE |
| chr1 | 145798865 | 145798875 | 5 | 2 | NA | FALSE | NA | 0 | 1 | FALSE | FALSE |
| chr1 | 145799048 | 145799173 | 13 | 10 | AluSz6 | TRUE | NA | 0 | 1 | FALSE | FALSE |
| chr1 | 146472288 | 146472378 | 5 | 4 | AluY | TRUE | NA | 0 | NA | FALSE | FALSE |
| chr1 | 146474239 | 146474309 | 4 | 3 | NA | FALSE | NA | 0 | 1 | FALSE | FALSE |
| chr1 | 146482219 | 146482289 | 13 | 7 | L1PA5 | TRUE | PU.1 | 1 | 1 | FALSE | FALSE |
| chr1 | 146482513 | 146482571 | 4 | 4 | L1PA5 | TRUE | NA | 0 | NA | FALSE | FALSE |
| chr1 | 146494370 | 146494510 | 5 | 5 | NA | FALSE | NA | 0 | 1 | FALSE | FALSE |
| chr1 | 146705020 | 146705039 | 5 | 5 | SVA D | TRUE | NA | 0 | NA | FALSE | FALSE |
| chr1 | 146942058 | 146942081 | 4 | 2 | L1PA12 | TRUE | NA | 0 | 1 | TRUE | FALSE |
| chr1 | 147746351 | 147746377 | 16 | 6 | L1PA16 | TRUE | NA | 0 | 1 | FALSE | FALSE |
| chr1 | 147823977 | 147824041 | 4 | 3 | NA | FALSE | NA | 0 | 1 | TRUE | FALSE |
| chr1 | 148186011 | 148186145 | 5 | 5 | L1PA6 | TRUE | NA | 0 | 1 | FALSE | FALSE |
| chr1 | 148349180 | 148349215 | 8 | 7 | NA | FALSE | NA | 0 | 1 | FALSE | FALSE |
| chr1 | 148512092 | 148512153 | 10 | 8 | L1M7 | TRUE | NA | 0 | 1 | FALSE | FALSE |
| chr1 | 148554729 | 148554771 | 9 | 7 | LTR71B | TRUE | NA | 0 | 1 | FALSE | FALSE |
| chr1 | 148562444 | 148562482 | 4 | 2 | CT-rich | TRUE | NA | 0 | NA | FALSE | FALSE |
| chr1 | 148633014 | 148633046 | 4 | 3 | AluJr | TRUE | NA | 0 | NA | FALSE | FALSE |
| chr1 | 148843997 | 148844039 | 10 | 7 | L1MEe | TRUE | NA | 0 | 1 | FALSE | FALSE |
| chr1 | 148923204 | 148923217 | 4 | 3 | L1PBa | TRUE | NA | 0 | 1 | FALSE | FALSE |
| chr1 | 148937171 | 148937189 | 5 | 3 | L1MEc | TRUE | NA | 0 | 1 | FALSE | FALSE |
| chr1 | 148950485 | 148950489 | 4 | 4 | L2c | TRUE | NA | 0 | 1 | FALSE | FALSE |
| chr1 | 149029261 | 149029300 | 7 | 7 | GA-rich | TRUE | NA | 0 | 1 | FALSE | FALSE |
| chr1 | 149033053 | 149033070 | 4 | 2 | L1M5 | TRUE | NA | 0 | NA | FALSE | FALSE |
| chr1 | 149040646 | 149040669 | 4 | 2 | NA | FALSE | NA | 0 | 1 | FALSE | FALSE |
| chr1 | 149052509 | 149052533 | 4 | 3 | NA | FALSE | NA | 0 | 1 | FALSE | FALSE |
| chr1 | 149088054 | 149088071 | 4 | 4 | NA | FALSE | NA | 0 | 1 | FALSE | FALSE |
| chr1 | 149096534 | 149096548 | 4 | 3 | NA | FALSE | NA | 0 | NA | FALSE | FALSE |
| chr1 | 149186565 | 149186579 | 4 | 3 | NA | FALSE | NA | 0 | 1 | TRUE | FALSE |
| chr1 | 149209119 | 149209122 | 6 | 6 | NA | FALSE | NA | 0 | NA | TRUE | FALSE |
| chr1 | 149214914 | 149214939 | 7 | 5 | LTR4 | TRUE | MANY | 13 | 1 | TRUE | TRUE |
| chr1 | 149240151 | 149240163 | 7 | 5 | NA | FALSE | NA | 0 | 1 | FALSE | FALSE |
| chr1 | 149343569 | 149343569 | 4 | 4 | NA | FALSE | NA | 0 | 1 | TRUE | FALSE |
| chr1 | 149346222 | 149346222 | 6 | 6 | L1MEg | TRUE | NA | 0 | 1 | FALSE | FALSE |
| chr1 | 151138465 | 151138524 | 4 | 4 | NA | FALSE | MANY | 68 | 1 | TRUE | TRUE |
| chr1 | 151913777 | 151913777 | 5 | 5 | (TC)n | TRUE | MANY | 3 | NA | TRUE | FALSE |
| chr1 | 152128192 | 152128231 | 4 | 4 | NA | FALSE | NA | 0 | 1 | FALSE | FALSE |
| chr1 | 152267624 | 152267654 | 4 | 4 | L1PA4 | TRUE | NA | 0 | NA | FALSE | FALSE |
| chr1 | 152302115 | 152302128 | 4 | 4 | (TC)n | TRUE | NA | 0 | 1 | FALSE | FALSE |
| chr1 | 152751078 | 152751092 | 4 | 3 | NA | FALSE | NA | 0 | 1 | TRUE | FALSE |
| chr1 | 153003158 | 153003179 | 4 | 4 | NA | FALSE | NA | 0 | 1 | FALSE | FALSE |
| chr1 | 153212845 | 153212862 | 4 | 2 | L2c | TRUE | NA | 0 | 1 | FALSE | FALSE |
| chr1 | 153357629 | 153357651 | 4 | 4 | NA | FALSE | STAT3 | 1 | 1 | TRUE | FALSE |
| chr1 | 153421491 | 153421519 | 5 | 4 | L1MC5 | TRUE | NA | 0 | 1 | TRUE | FALSE |
| chr1 | 153662579 | 153662618 | 6 | 6 | (TC)n | TRUE | NA | 0 | NA | FALSE | FALSE |
| chr1 | 153891198 | 153891203 | 4 | 4 | AluSq | TRUE | NA | 0 | NA | FALSE | FALSE |
| chr1 | 153963193 | 153963239 | 9 | 7 | NA | FALSE | MANY | 63 | 1 | TRUE | TRUE |
| chr1 | 154285808 | 154285871 | 4 | 3 | NA | FALSE | MANY | 6 | 1 | FALSE | FALSE |
| chr1 | 154650501 | 154650547 | 4 | 4 | LTR12C | TRUE | NA | 0 | 1 | FALSE | FALSE |
| chr1 | 154663418 | 154663473 | 4 | 3 | AluY | TRUE | NA | 0 | NA | FALSE | FALSE |
| chr1 | 156362091 | 156362095 | 4 | 4 | NA | FALSE | NA | 0 | 1 | TRUE | FALSE |
| chr1 | 157044716 | 157044716 | 4 | 4 | NA | FALSE | NA | 0 | NA | FALSE | FALSE |
| chr1 | 157134156 | 157134188 | 4 | 2 | NA | FALSE | NA | 0 | 1 | TRUE | FALSE |
| chr1 | 157268918 | 157268942 | 4 | 4 | L1PA7 | TRUE | NA | 0 | NA | FALSE | FALSE |
| chr1 | 157299874 | 157299886 | 4 | 2 | MER4-int | TRUE | KAP1 | 1 | NA | FALSE | FALSE |
| chr1 | 157882259 | 157882281 | 4 | 3 | L1MB3 | TRUE | NA | 0 | 1 | FALSE | FALSE |
| chr1 | 158177611 | 158177628 | 4 | 4 | L1M2 | TRUE | NA | 0 | 1 | FALSE | FALSE |
| chr1 | 159318204 | 159318223 | 4 | 4 | L1PA7 | TRUE | NA | 0 | NA | FALSE | FALSE |
| chr1 | 159333684 | 159333692 | 4 | 4 | NA | FALSE | NA | 0 | 1 | FALSE | FALSE |
| chr1 | 159491812 | 159491822 | 4 | 4 | MANY | TRUE | NA | 0 | 1 | FALSE | FALSE |
| chr1 | 159564727 | 159564729 | 4 | 3 | (GA)n | TRUE | NA | 0 | NA | FALSE | FALSE |
| chr1 | 159773528 | 159773540 | 4 | 3 | NA | FALSE | NA | 0 | 1 | TRUE | FALSE |
| chr1 | 159774185 | 159774207 | 4 | 4 | L3 | TRUE | CEBPB | 1 | 1 | FALSE | FALSE |
| chr1 | 160451778 | 160451815 | 4 | 4 | NA | FALSE | NA | 0 | 1 | TRUE | FALSE |
| chr1 | 160710084 | 160710120 | 5 | 5 | NA | FALSE | MANY | 2 | 1 | TRUE | FALSE |
| chr1 | 160726752 | 160726802 | 4 | 2 | L2b | TRUE | NA | 0 | 1 | TRUE | FALSE |
| chr1 | 161519385 | 161519423 | 5 | 4 | NA | FALSE | NA | 0 | 1 | FALSE | FALSE |
| chr1 | 161580152 | 161580156 | 5 | 5 | NA | FALSE | NA | 0 | 1 | TRUE | FALSE |
| chr1 | 162860516 | 162860517 | 4 | 3 | AluSc | TRUE | NA | 0 | NA | FALSE | FALSE |
| chr1 | 163455559 | 163455567 | 4 | 3 | L1MEc | TRUE | NA | 0 | 1 | FALSE | FALSE |
| chr1 | 163538446 | 163538457 | 4 | 4 | AluSq2 | TRUE | NA | 0 | NA | FALSE | FALSE |
| chr1 | 163660823 | 163660823 | 4 | 4 | MIRc | TRUE | NA | 0 | NA | FALSE | FALSE |
| chr1 | 163661691 | 163661691 | 4 | 4 | MIRc | TRUE | NA | 0 | 1 | FALSE | FALSE |
| chr1 | 163678009 | 163678014 | 4 | 4 | MSTA | TRUE | NA | 0 | 1 | FALSE | FALSE |
| chr1 | 163780621 | 163780631 | 4 | 3 | AluSq2 | TRUE | NA | 0 | NA | FALSE | FALSE |
| chr1 | 163848684 | 163848698 | 4 | 4 | NA | FALSE | NA | 0 | 1 | FALSE | FALSE |
| chr1 | 163988301 | 163988306 | 4 | 4 | L1M3e | TRUE | NA | 0 | 1 | FALSE | FALSE |
| chr1 | 164147700 | 164147711 | 4 | 3 | NA | FALSE | NA | 0 | 1 | FALSE | FALSE |
| chr1 | 164285865 | 164285882 | 4 | 4 | L2c | TRUE | NA | 0 | 1 | FALSE | FALSE |
| chr1 | 165083718 | 165083719 | 4 | 4 | NA | FALSE | MANY | 7 | 1 | TRUE | TRUE |
| chr1 | 166240415 | 166240435 | 4 | 4 | NA | FALSE | NA | 0 | 1 | FALSE | FALSE |
| chr1 | 166735665 | 166735682 | 4 | 4 | NA | FALSE | NA | 0 | 1 | FALSE | FALSE |
| chr1 | 167143329 | 167143355 | 4 | 2 | NA | FALSE | NA | 0 | 1 | TRUE | FALSE |
| chr1 | 167583398 | 167583427 | 4 | 3 | (TTCC)n | TRUE | NA | 0 | 1 | FALSE | FALSE |
| chr1 | 168383242 | 168383273 | 4 | 4 | FLAM C | TRUE | NA | 0 | 1 | FALSE | FALSE |
| chr1 | 170749504 | 170749538 | 4 | 4 | AluY | TRUE | NA | 0 | NA | FALSE | FALSE |
| chr1 | 170815798 | 170815822 | 4 | 4 | NA | FALSE | NA | 0 | 1 | FALSE | FALSE |
| chr1 | 172563371 | 172563375 | 4 | 3 | L1PA5 | TRUE | NA | 0 | NA | FALSE | FALSE |
| chr1 | 172721580 | 172721600 | 4 | 3 | MIRc | TRUE | NA | 0 | 1 | FALSE | FALSE |
| chr1 | 172961862 | 172961877 | 4 | 2 | L2b | TRUE | NA | 0 | 1 | FALSE | FALSE |
| chr1 | 172994658 | 172994679 | 4 | 4 | MSTA | TRUE | NA | 0 | 1 | FALSE | FALSE |
| chr1 | 173242855 | 173242859 | 4 | 3 | NA | FALSE | NA | 0 | 1 | FALSE | FALSE |
| chr1 | 175664965 | 175664992 | 4 | 3 | L2b | TRUE | NA | 0 | 1 | FALSE | FALSE |
| chr1 | 175758209 | 175758215 | 4 | 3 | NA | FALSE | NA | 0 | 1 | TRUE | FALSE |
| chr1 | 175764254 | 175764267 | 4 | 4 | NA | FALSE | NA | 0 | 1 | FALSE | FALSE |
| chr1 | 175961949 | 175961959 | 6 | 4 | L1PA3 | TRUE | NA | 0 | 1 | FALSE | FALSE |
| chr1 | 176327709 | 176327737 | 7 | 3 | L1PA6 | TRUE | NA | 0 | 1 | FALSE | FALSE |
| chr1 | 176422803 | 176422827 | 4 | 3 | L2c | TRUE | NA | 0 | 1 | FALSE | FALSE |
| chr1 | 176727407 | 176727408 | 4 | 4 | NA | FALSE | NA | 0 | 1 | FALSE | FALSE |
| chr1 | 176903173 | 176903186 | 6 | 5 | NA | FALSE | NA | 0 | 1 | FALSE | FALSE |
| chr1 | 176908945 | 176908960 | 5 | 4 | (GGAA)n | TRUE | NA | 0 | NA | FALSE | FALSE |
| chr1 | 177313549 | 177313567 | 4 | 2 | NA | FALSE | NA | 0 | 1 | FALSE | FALSE |
| chr1 | 177541773 | 177541773 | 4 | 4 | NA | FALSE | NA | 0 | 1 | FALSE | FALSE |
| chr1 | 178419004 | 178419034 | 4 | 3 | NA | FALSE | NA | 0 | 1 | FALSE | FALSE |
| chr1 | 179104924 | 179104964 | 4 | 3 | SVA D | TRUE | NA | 0 | NA | FALSE | FALSE |
| chr1 | 179236741 | 179236742 | 7 | 4 | AluY | TRUE | NA | 0 | NA | FALSE | FALSE |
| chr1 | 180628403 | 180628411 | 4 | 4 | (TTCC)n | TRUE | NA | 0 | 1 | FALSE | FALSE |
| chr1 | 181315544 | 181315545 | 4 | 3 | L2a | TRUE | NA | 0 | 1 | TRUE | FALSE |
| chr1 | 181354954 | 181354981 | 4 | 4 | CT-rich | TRUE | NA | 0 | 1 | FALSE | FALSE |
| chr1 | 181841474 | 181841480 | 5 | 5 | L1PB1 | TRUE | NA | 0 | 1 | FALSE | FALSE |
| chr1 | 182581036 | 182581066 | 4 | 3 | NA | FALSE | TCF12 | 1 | 1 | TRUE | FALSE |
| chr1 | 184202934 | 184202996 | 4 | 4 | L1PA4 | TRUE | NA | 0 | NA | FALSE | FALSE |
| chr1 | 184836240 | 184836303 | 5 | 5 | (TC)n | TRUE | MANY | 23 | 1 | TRUE | TRUE |
| chr1 | 184973214 | 184973234 | 4 | 2 | L1PA3 | TRUE | NA | 0 | NA | FALSE | FALSE |
| chr1 | 185571075 | 185571116 | 4 | 4 | (TCTA)n | TRUE | NA | 0 | 1 | FALSE | FALSE |
| chr1 | 186171703 | 186171768 | 4 | 4 | NA | FALSE | NA | 0 | 1 | TRUE | FALSE |
| chr1 | 187209707 | 187209741 | 4 | 4 | NA | FALSE | NA | 0 | 1 | FALSE | FALSE |
| chr1 | 187340257 | 187340286 | 4 | 3 | AluYf4 | TRUE | NA | 0 | NA | FALSE | FALSE |
| chr1 | 187402192 | 187402227 | 6 | 4 | AluY | TRUE | NA | 0 | NA | FALSE | FALSE |
| chr1 | 187923211 | 187923251 | 5 | 4 | NA | FALSE | NA | 0 | 1 | FALSE | FALSE |
| chr1 | 188157538 | 188157570 | 4 | 3 | NA | FALSE | MANY | 3 | 1 | TRUE | TRUE |
| chr1 | 189038732 | 189038756 | 4 | 4 | MER34-int | TRUE | NA | 0 | 1 | FALSE | FALSE |
| chr1 | 189415635 | 189415638 | 4 | 3 | L1PA5 | TRUE | NA | 0 | NA | FALSE | FALSE |
| chr1 | 189614452 | 189614459 | 4 | 2 | AluSc8 | TRUE | NA | 0 | NA | FALSE | FALSE |
| chr1 | 189894244 | 189894262 | 4 | 4 | L1PA15 | TRUE | NA | 0 | 1 | FALSE | FALSE |
| chr1 | 190078136 | 190078144 | 4 | 4 | NA | FALSE | NA | 0 | 1 | FALSE | FALSE |
| chr1 | 190153348 | 190153362 | 4 | 4 | NA | FALSE | NA | 0 | 1 | FALSE | FALSE |
| chr1 | 190764197 | 190764210 | 4 | 2 | NA | FALSE | NA | 0 | 1 | FALSE | FALSE |
| chr1 | 190810589 | 190810595 | 4 | 2 | NA | FALSE | NA | 0 | 1 | FALSE | FALSE |
| chr1 | 191296498 | 191296516 | 4 | 3 | NA | FALSE | NA | 0 | 1 | FALSE | FALSE |
| chr1 | 192241827 | 192241844 | 4 | 3 | NA | FALSE | NA | 0 | 1 | FALSE | FALSE |
| chr1 | 192378631 | 192378670 | 4 | 3 | MamGypLTR2c | TRUE | NA | 0 | 1 | FALSE | FALSE |
| chr1 | 193321871 | 193321874 | 6 | 2 | MLT2D | TRUE | NA | 0 | NA | FALSE | FALSE |
| chr1 | 193883222 | 193883245 | 4 | 4 | NA | FALSE | NA | 0 | 1 | FALSE | FALSE |
| chr1 | 194054109 | 194054110 | 4 | 4 | NA | FALSE | NA | 0 | NA | TRUE | FALSE |
| chr1 | 194318958 | 194318965 | 5 | 4 | THE1A | TRUE | NA | 0 | 1 | FALSE | FALSE |
| chr1 | 194656937 | 194656937 | 4 | 4 | L1PB | TRUE | NA | 0 | NA | FALSE | FALSE |
| chr1 | 195043544 | 195043544 | 4 | 4 | NA | FALSE | NA | 0 | NA | FALSE | FALSE |
| chr1 | 195758710 | 195758723 | 4 | 3 | L1MEg | TRUE | NA | 0 | 1 | TRUE | FALSE |
| chr1 | 196049059 | 196049060 | 4 | 4 | L1ME1 | TRUE | NA | 0 | 1 | TRUE | FALSE |
| chr1 | 196273568 | 196273592 | 6 | 3 | AluJo | TRUE | NA | 0 | 1 | FALSE | FALSE |
| chr1 | 196388940 | 196388962 | 5 | 4 | HAL1 | TRUE | NA | 0 | 1 | FALSE | FALSE |
| chr1 | 196701816 | 196701837 | 4 | 3 | L1M3de | TRUE | MANY | 3 | 1 | TRUE | TRUE |
| chr1 | 196822569 | 196822600 | 5 | 3 | NA | FALSE | NA | 0 | 1 | FALSE | FALSE |
| chr1 | 196925903 | 196925905 | 5 | 3 | L1MDa | TRUE | NA | 0 | 1 | FALSE | FALSE |
| chr1 | 196927544 | 196927546 | 5 | 5 | (TG)n | TRUE | NA | 0 | NA | FALSE | FALSE |
| chr1 | 197400676 | 197400701 | 4 | 4 | NA | FALSE | NA | 0 | 1 | FALSE | FALSE |
| chr1 | 197410835 | 197410837 | 4 | 2 | NA | FALSE | NA | 0 | 1 | FALSE | FALSE |
| chr1 | 197431581 | 197431607 | 4 | 2 | NA | FALSE | NA | 0 | 1 | FALSE | FALSE |
| chr1 | 197438135 | 197438163 | 4 | 3 | NA | FALSE | NA | 0 | 1 | TRUE | FALSE |
| chr1 | 197990498 | 197990536 | 4 | 4 | AluSc8 | TRUE | NA | 0 | NA | FALSE | FALSE |
| chr1 | 198009870 | 198009890 | 4 | 3 | LTR7 | TRUE | NA | 0 | 1 | TRUE | FALSE |
| chr1 | 198196861 | 198196882 | 4 | 2 | L1PA4 | TRUE | NA | 0 | NA | FALSE | FALSE |
| chr1 | 198537896 | 198537912 | 4 | 4 | NA | FALSE | NA | 0 | 1 | FALSE | FALSE |
| chr1 | 198602836 | 198602874 | 4 | 4 | MER1B | TRUE | MANY | 3 | 1 | TRUE | TRUE |
| chr1 | 198943115 | 198943158 | 4 | 2 | Tigger6a | TRUE | NA | 0 | 1 | FALSE | FALSE |
| chr1 | 198994707 | 198994709 | 4 | 3 | L1HS | TRUE | NA | 0 | NA | FALSE | FALSE |
| chr1 | 199874478 | 199874503 | 5 | 4 | SVA D | TRUE | NA | 0 | NA | FALSE | FALSE |
| chr1 | 200645461 | 200645468 | 4 | 4 | AluSq10 | TRUE | NA | 0 | NA | FALSE | FALSE |
| chr1 | 201023855 | 201023859 | 6 | 5 | NA | FALSE | NA | 0 | 1 | FALSE | FALSE |
| chr1 | 201178941 | 201178983 | 5 | 4 | NA | FALSE | NA | 0 | 1 | FALSE | FALSE |
| chr1 | 201225404 | 201225510 | 6 | 3 | MANY | TRUE | NA | 0 | 1 | FALSE | FALSE |
| chr1 | 202957729 | 202957781 | 4 | 3 | NA | FALSE | NA | 0 | 1 | TRUE | FALSE |
| chr1 | 203195389 | 203195447 | 4 | 3 | NA | FALSE | NA | 0 | 1 | FALSE | FALSE |
| chr1 | 203810850 | 203810915 | 4 | 2 | SVA B | TRUE | NA | 0 | 1 | FALSE | FALSE |
| chr1 | 204894225 | 204894271 | 4 | 3 | AluSx1 | TRUE | NA | 0 | 1 | FALSE | FALSE |
| chr1 | 205902014 | 205902072 | 4 | 3 | NA | FALSE | NA | 0 | 1 | TRUE | FALSE |
| chr1 | 206096214 | 206096242 | 4 | 4 | NA | FALSE | NA | 0 | 1 | TRUE | FALSE |
| chr1 | 206112483 | 206112541 | 4 | 4 | NA | FALSE | NA | 0 | NA | FALSE | FALSE |
| chr1 | 206179288 | 206179349 | 4 | 3 | L1MA9 | TRUE | NA | 0 | 1 | FALSE | FALSE |
| chr1 | 206185174 | 206185225 | 4 | 4 | AluJr4 | TRUE | NA | 0 | 1 | FALSE | FALSE |
| chr1 | 206294532 | 206294610 | 4 | 4 | MIRb | TRUE | NA | 0 | 1 | TRUE | FALSE |
| chr1 | 206519271 | 206519271 | 4 | 4 | AluJo | TRUE | NA | 0 | 1 | TRUE | FALSE |
| chr1 | 206709071 | 206709073 | 7 | 6 | (TG)n | TRUE | NA | 0 | NA | FALSE | FALSE |
| chr1 | 207665118 | 207665165 | 4 | 3 | NA | FALSE | NA | 0 | 1 | FALSE | FALSE |
| chr1 | 208103386 | 208103434 | 4 | 3 | Tigger1 | TRUE | NA | 0 | 1 | FALSE | FALSE |
| chr1 | 208481469 | 208481469 | 4 | 4 | NA | FALSE | NA | 0 | 1 | FALSE | FALSE |
| chr1 | 208707965 | 208707966 | 4 | 3 | NA | FALSE | NA | 0 | 1 | TRUE | FALSE |
| chr1 | 209170766 | 209170781 | 5 | 4 | L1PA5 | TRUE | NA | 0 | NA | FALSE | FALSE |
| chr1 | 209423061 | 209423077 | 4 | 4 | NA | FALSE | NA | 0 | 1 | FALSE | FALSE |
| chr1 | 209476182 | 209476200 | 5 | 5 | NA | FALSE | NA | 0 | 1 | FALSE | FALSE |
| chr1 | 209679365 | 209679385 | 4 | 4 | NA | FALSE | NA | 0 | 1 | FALSE | FALSE |
| chr1 | 210965479 | 210965513 | 4 | 3 | NA | FALSE | NA | 0 | 1 | TRUE | FALSE |
| chr1 | 211752640 | 211752707 | 5 | 4 | NA | FALSE | MANY | 16 | 1 | TRUE | TRUE |
| chr1 | 212142563 | 212142642 | 4 | 3 | AluY | TRUE | NA | 0 | 1 | FALSE | FALSE |
| chr1 | 212565312 | 212565392 | 5 | 4 | L1PA5 | TRUE | NA | 0 | NA | FALSE | FALSE |
| chr1 | 212732420 | 212732422 | 4 | 3 | NA | FALSE | MANY | 48 | 1 | TRUE | TRUE |
| chr1 | 212812956 | 212813027 | 5 | 5 | MANY | TRUE | NA | 0 | 1 | FALSE | FALSE |
| chr1 | 213901065 | 213901092 | 4 | 3 | X1 LINE | TRUE | NA | 0 | 1 | FALSE | FALSE |
| chr1 | 213942578 | 213942591 | 4 | 2 | NA | FALSE | NA | 0 | 1 | FALSE | FALSE |
| chr1 | 214286082 | 214286100 | 4 | 4 | MER33 | TRUE | NA | 0 | 1 | TRUE | FALSE |
| chr1 | 216648191 | 216648218 | 4 | 4 | NA | FALSE | NA | 0 | 1 | FALSE | FALSE |
| chr1 | 216860388 | 216860394 | 4 | 4 | (TG)n | TRUE | JunD | 1 | NA | TRUE | FALSE |
| chr1 | 220013431 | 220013441 | 4 | 2 | L1PA3 | TRUE | NA | 0 | NA | FALSE | FALSE |
| chr1 | 220666465 | 220666484 | 4 | 4 | MANY | TRUE | NA | 0 | 1 | FALSE | FALSE |
| chr1 | 220930423 | 220930450 | 4 | 2 | AluSq2 | TRUE | NA | 0 | NA | FALSE | FALSE |
| chr1 | 221162568 | 221162598 | 5 | 4 | L1MA5 | TRUE | PU.1 | 1 | 1 | FALSE | FALSE |
| chr1 | 221163306 | 221163330 | 4 | 2 | L1MA5 | TRUE | NA | 0 | NA | FALSE | FALSE |
| chr1 | 221350272 | 221350285 | 4 | 4 | NA | FALSE | NA | 0 | 1 | FALSE | FALSE |
| chr1 | 221398109 | 221398117 | 4 | 3 | L1ME2 | TRUE | NA | 0 | 1 | FALSE | FALSE |
| chr1 | 221444296 | 221444308 | 4 | 2 | AluY | TRUE | NA | 0 | 1 | FALSE | FALSE |
| chr1 | 221453947 | 221453968 | 4 | 4 | L1PA16 | TRUE | NA | 0 | 1 | FALSE | FALSE |
| chr1 | 221501237 | 221501238 | 4 | 3 | NA | FALSE | ZNF263 | 1 | NA | TRUE | FALSE |
| chr1 | 221566455 | 221566476 | 4 | 2 | NA | FALSE | NA | 0 | 1 | FALSE | FALSE |
| chr1 | 222647230 | 222647246 | 6 | 6 | NA | FALSE | NA | 0 | NA | TRUE | FALSE |
| chr1 | 222648161 | 222648214 | 6 | 3 | NA | FALSE | NA | 0 | 1 | FALSE | FALSE |
| chr1 | 223405089 | 223405105 | 4 | 3 | NA | FALSE | NA | 0 | 1 | TRUE | FALSE |
| chr1 | 223549804 | 223549840 | 4 | 4 | AluSc | TRUE | NA | 0 | 1 | FALSE | FALSE |
| chr1 | 223560661 | 223560709 | 4 | 4 | NA | FALSE | NA | 0 | 1 | FALSE | FALSE |
| chr1 | 224166641 | 224166697 | 5 | 4 | NA | FALSE | NA | 0 | NA | FALSE | FALSE |
| chr1 | 224172586 | 224172586 | 4 | 4 | MIRb | TRUE | NA | 0 | 1 | FALSE | FALSE |
| chr1 | 224172752 | 224172807 | 9 | 8 | NA | FALSE | NA | 0 | 1 | FALSE | FALSE |
| chr1 | 225026968 | 225027004 | 4 | 3 | AluY | TRUE | NA | 0 | NA | FALSE | FALSE |
| chr1 | 225266558 | 225266568 | 6 | 5 | (TC)n | TRUE | NA | 0 | NA | FALSE | FALSE |
| chr1 | 226142693 | 226142761 | 5 | 4 | NA | FALSE | NA | 0 | 1 | FALSE | FALSE |
| chr1 | 226244553 | 226244581 | 10 | 5 | AluSz6 | TRUE | NA | 0 | NA | FALSE | FALSE |
| chr1 | 226250284 | 226250318 | 5 | 5 | G-rich | TRUE | MANY | 16 | 1 | TRUE | TRUE |
| chr1 | 226803864 | 226803911 | 4 | 3 | MER61-int | TRUE | NA | 0 | 1 | FALSE | FALSE |
| chr1 | 226986162 | 226986227 | 4 | 2 | NA | FALSE | MANY | 5 | 1 | TRUE | TRUE |
| chr1 | 227143327 | 227143403 | 6 | 5 | C-rich | TRUE | NA | 0 | 1 | FALSE | FALSE |
| chr1 | 228422069 | 228422096 | 4 | 3 | AluSq2 | TRUE | NA | 0 | NA | FALSE | FALSE |
| chr1 | 228954096 | 228954146 | 4 | 2 | MER52C | TRUE | NA | 0 | 1 | TRUE | FALSE |
| chr1 | 229085699 | 229085749 | 4 | 2 | HERVH-int | TRUE | NA | 0 | 1 | FALSE | FALSE |
| chr1 | 229172735 | 229172784 | 4 | 3 | NA | FALSE | NA | 0 | 1 | FALSE | FALSE |
| chr1 | 229551877 | 229551888 | 4 | 3 | HERVH-int | TRUE | NA | 0 | 1 | FALSE | FALSE |
| chr1 | 229858198 | 229858291 | 6 | 6 | L1MB3 | TRUE | NA | 0 | 1 | FALSE | FALSE |
| chr1 | 230175682 | 230175754 | 6 | 4 | MANY | TRUE | NA | 0 | 1 | FALSE | FALSE |
| chr1 | 230737239 | 230737282 | 4 | 4 | LTR7 | TRUE | NA | 0 | NA | TRUE | FALSE |
| chr1 | 230756274 | 230756340 | 4 | 4 | MER4A | TRUE | NA | 0 | 1 | FALSE | FALSE |
| chr1 | 231114783 | 231114787 | 4 | 3 | NA | FALSE | MANY | 39 | 1 | TRUE | TRUE |
| chr1 | 231435616 | 231435692 | 4 | 3 | NA | FALSE | NA | 0 | 1 | TRUE | FALSE |
| chr1 | 231473663 | 231473745 | 6 | 4 | NA | FALSE | MANY | 20 | 1 | TRUE | TRUE |
| chr1 | 232370203 | 232370296 | 5 | 3 | MANY | TRUE | NA | 0 | 1 | FALSE | FALSE |
| chr1 | 232468004 | 232468053 | 4 | 3 | SVA D | TRUE | NA | 0 | NA | FALSE | FALSE |
| chr1 | 233006911 | 233006915 | 5 | 3 | LTR12C | TRUE | NA | 0 | 1 | FALSE | FALSE |
| chr1 | 233017245 | 233017284 | 4 | 2 | L2a | TRUE | NA | 0 | 1 | TRUE | FALSE |
| chr1 | 233615239 | 233615252 | 4 | 4 | NA | FALSE | MANY | 2 | 1 | TRUE | FALSE |
| chr1 | 233689228 | 233689235 | 4 | 3 | AluJr | TRUE | NA | 0 | 1 | FALSE | FALSE |
| chr1 | 233691568 | 233691568 | 4 | 4 | NA | FALSE | NA | 0 | 1 | FALSE | FALSE |
| chr1 | 233765505 | 233765513 | 4 | 3 | NA | FALSE | NA | 0 | 1 | FALSE | FALSE |
| chr1 | 233780722 | 233780730 | 9 | 5 | AluSc8 | TRUE | NA | 0 | NA | FALSE | FALSE |
| chr1 | 234822823 | 234822854 | 4 | 3 | L1ME1 | TRUE | NA | 0 | 1 | TRUE | FALSE |
| chr1 | 235049551 | 235049584 | 4 | 2 | NA | FALSE | YY1 | 1 | 1 | TRUE | FALSE |
| chr1 | 235304501 | 235304501 | 4 | 4 | LTR12C | TRUE | NA | 0 | NA | FALSE | FALSE |
| chr1 | 235472706 | 235472785 | 11 | 8 | SVA D | TRUE | NA | 0 | 1 | FALSE | FALSE |
| chr1 | 235548012 | 235548108 | 6 | 4 | L1PA3 | TRUE | NA | 0 | 1 | FALSE | FALSE |
| chr1 | 235548205 | 235548342 | 5 | 3 | L1PA3 | TRUE | NA | 0 | 1 | FALSE | FALSE |
| chr1 | 236832301 | 236832358 | 4 | 4 | MANY | TRUE | NA | 0 | 1 | TRUE | FALSE |
| chr1 | 236840467 | 236840501 | 4 | 4 | Arthur1A | TRUE | NA | 0 | 1 | FALSE | FALSE |
| chr1 | 236920486 | 236920540 | 7 | 3 | NA | FALSE | NA | 0 | NA | FALSE | FALSE |
| chr1 | 237146945 | 237146978 | 4 | 3 | AluJo | TRUE | NA | 0 | 1 | FALSE | FALSE |
| chr1 | 238047288 | 238047306 | 4 | 3 | (CA)n | TRUE | NA | 0 | NA | FALSE | FALSE |
| chr1 | 238380240 | 238380267 | 4 | 2 | NA | FALSE | NA | 0 | 1 | FALSE | FALSE |
| chr1 | 238496260 | 238496285 | 4 | 3 | MLT1E2 | TRUE | NA | 0 | 1 | FALSE | FALSE |
| chr1 | 239401632 | 239401632 | 4 | 4 | MER61A | TRUE | NA | 0 | 1 | FALSE | FALSE |
| chr1 | 239930363 | 239930364 | 4 | 2 | NA | FALSE | NA | 0 | 1 | FALSE | FALSE |
| chr1 | 240438746 | 240438761 | 4 | 3 | L1PA7 | TRUE | NA | 0 | NA | FALSE | FALSE |
| chr1 | 240947989 | 240948001 | 5 | 4 | NA | FALSE | NA | 0 | 1 | TRUE | FALSE |
| chr1 | 240977501 | 240977517 | 4 | 3 | NA | FALSE | NA | 0 | 1 | FALSE | FALSE |
| chr1 | 241044218 | 241044230 | 4 | 4 | NA | FALSE | NA | 0 | 1 | FALSE | FALSE |
| chr1 | 241380741 | 241380750 | 4 | 3 | NA | FALSE | NA | 0 | 1 | FALSE | FALSE |
| chr1 | 241452909 | 241452909 | 4 | 4 | NA | FALSE | NA | 0 | 1 | FALSE | FALSE |
| chr1 | 242011440 | 242011462 | 5 | 5 | NA | FALSE | MANY | 26 | 1 | TRUE | TRUE |
| chr1 | 242374099 | 242374099 | 4 | 4 | LTR7 | TRUE | NA | 0 | NA | FALSE | FALSE |
| chr1 | 242487962 | 242488012 | 8 | 5 | LTR8 | TRUE | NA | 0 | 1 | FALSE | FALSE |
| chr1 | 243018048 | 243018082 | 4 | 3 | L1PB1 | TRUE | NA | 0 | 1 | TRUE | FALSE |
| chr1 | 243205358 | 243205361 | 4 | 4 | NA | FALSE | NA | 0 | NA | FALSE | FALSE |
| chr1 | 243247580 | 243247582 | 4 | 3 | NA | FALSE | NA | 0 | NA | TRUE | FALSE |
| chr1 | 243326612 | 243326616 | 4 | 2 | NA | FALSE | NA | 0 | 1 | TRUE | FALSE |
| chr1 | 243525422 | 243525472 | 4 | 3 | NA | FALSE | NA | 0 | NA | FALSE | FALSE |
| chr1 | 243525542 | 243525586 | 6 | 5 | NA | FALSE | NA | 0 | 1 | FALSE | FALSE |
| chr1 | 243701988 | 243702051 | 4 | 1 | MANY | TRUE | NA | 0 | 1 | FALSE | FALSE |
| chr1 | 244650322 | 244650377 | 5 | 5 | NA | FALSE | NA | 0 | 1 | FALSE | FALSE |
| chr1 | 244674174 | 244674235 | 4 | 4 | AluSq | TRUE | NA | 0 | NA | FALSE | FALSE |
| chr1 | 245100395 | 245100431 | 4 | 4 | G-rich | TRUE | NA | 0 | NA | TRUE | FALSE |
| chr1 | 245410195 | 245410209 | 4 | 4 | (CA)n | TRUE | NA | 0 | NA | FALSE | FALSE |
| chr1 | 245636087 | 245636096 | 4 | 3 | L2b | TRUE | NA | 0 | 1 | FALSE | FALSE |
| chr1 | 246140085 | 246140200 | 7 | 5 | NA | FALSE | NA | 0 | NA | FALSE | FALSE |
| chr1 | 246145654 | 246145697 | 4 | 3 | AluJo | TRUE | NA | 0 | 1 | FALSE | FALSE |
| chr1 | 246188450 | 246188600 | 10 | 9 | NA | FALSE | NA | 0 | 1 | FALSE | FALSE |
| chr1 | 246395923 | 246395987 | 4 | 4 | NA | FALSE | NA | 0 | NA | FALSE | FALSE |
| chr1 | 246395992 | 246396041 | 8 | 7 | NA | FALSE | NA | 0 | NA | FALSE | FALSE |
| chr1 | 246396528 | 246396648 | 12 | 9 | NA | FALSE | NA | 0 | 1 | FALSE | FALSE |
| chr1 | 246440177 | 246440240 | 5 | 5 | MIR | TRUE | NA | 0 | NA | FALSE | FALSE |
| chr1 | 246488379 | 246488407 | 4 | 4 | G-rich | TRUE | NA | 0 | NA | TRUE | FALSE |
| chr1 | 246641829 | 246642024 | 10 | 8 | NA | FALSE | NA | 0 | 1 | FALSE | FALSE |
| chr1 | 246684167 | 246684257 | 8 | 6 | NA | FALSE | NA | 0 | NA | FALSE | FALSE |
| chr1 | 246768989 | 246768990 | 5 | 3 | MER48 | TRUE | NA | 0 | NA | TRUE | FALSE |
| chr1 | 246949056 | 246949090 | 5 | 4 | NA | FALSE | MANY | 2 | 1 | TRUE | FALSE |
| chr1 | 247162874 | 247162928 | 4 | 3 | NA | FALSE | NA | 0 | 1 | FALSE | FALSE |
| chr1 | 247790242 | 247790263 | 4 | 3 | MANY | TRUE | NA | 0 | 1 | FALSE | FALSE |
| chr1 | 247939508 | 247939522 | 4 | 4 | NA | FALSE | NA | 0 | 1 | FALSE | FALSE |
| chr1 | 248073903 | 248073920 | 4 | 4 | SATR1 | TRUE | NA | 0 | 1 | FALSE | FALSE |
| chr1 | 248231864 | 248231867 | 4 | 2 | L1PA3 | TRUE | NA | 0 | NA | FALSE | FALSE |
| chr1 | 248609433 | 248609476 | 6 | 6 | NA | FALSE | NA | 0 | 1 | TRUE | FALSE |
| chr1 | 248869943 | 248869981 | 5 | 4 | NA | FALSE | NA | 0 | 1 | FALSE | FALSE |
| chr1 | 248906013 | 248906052 | 4 | 3 | NA | FALSE | NA | 0 | NA | TRUE | FALSE |
| chr1 | 249071326 | 249071375 | 4 | 3 | THE1D-int | TRUE | NA | 0 | 1 | FALSE | FALSE |
| chr2 | 178088 | 178100 | 4 | 3 | NA | FALSE | NA | 0 | 1 | FALSE | FALSE |
| chr2 | 300943 | 300970 | 4 | 3 | NA | FALSE | NA | 0 | 1 | FALSE | FALSE |
| chr2 | 728645 | 728645 | 10 | 10 | (TG)n | TRUE | NA | 0 | NA | TRUE | FALSE |
| chr2 | 1045930 | 1045940 | 4 | 2 | L1ME3A | TRUE | NA | 0 | 1 | FALSE | FALSE |
| chr2 | 1110933 | 1110933 | 4 | 4 | NA | FALSE | NA | 0 | 1 | FALSE | FALSE |
| chr2 | 1160353 | 1160371 | 4 | 3 | NA | FALSE | NA | 0 | 1 | TRUE | FALSE |
| chr2 | 1239395 | 1239406 | 4 | 4 | NA | FALSE | NA | 0 | 1 | TRUE | FALSE |
| chr2 | 1414832 | 1414850 | 5 | 4 | NA | FALSE | NA | 0 | 1 | FALSE | FALSE |
| chr2 | 1461095 | 1461102 | 4 | 4 | NA | FALSE | NA | 0 | 1 | FALSE | FALSE |
| chr2 | 1600759 | 1600779 | 4 | 4 | (CA)n | TRUE | c-Myc | 1 | NA | FALSE | FALSE |
| chr2 | 1600910 | 1600916 | 7 | 5 | (CA)n | TRUE | c-Myc | 1 | 1 | FALSE | FALSE |
| chr2 | 2397983 | 2398003 | 4 | 3 | NA | FALSE | NA | 0 | 1 | FALSE | FALSE |
| chr2 | 2466029 | 2466050 | 4 | 4 | NA | FALSE | NA | 0 | 1 | FALSE | FALSE |
| chr2 | 2909866 | 2909882 | 4 | 3 | NA | FALSE | NA | 0 | 1 | FALSE | FALSE |
| chr2 | 3243876 | 3243927 | 10 | 8 | NA | FALSE | NA | 0 | NA | FALSE | FALSE |
| chr2 | 3244154 | 3244183 | 5 | 5 | NA | FALSE | NA | 0 | NA | FALSE | FALSE |
| chr2 | 3244917 | 3244917 | 4 | 4 | NA | FALSE | NA | 0 | 1 | FALSE | FALSE |
| chr2 | 3273145 | 3273166 | 4 | 4 | (CTCA)n | TRUE | MANY | 3 | NA | TRUE | FALSE |
| chr2 | 3303394 | 3303396 | 4 | 4 | (TC)n | TRUE | NA | 0 | NA | FALSE | FALSE |
| chr2 | 3332345 | 3332422 | 5 | 2 | NA | FALSE | NA | 0 | 1 | FALSE | FALSE |
| chr2 | 3441545 | 3441591 | 5 | 4 | NA | FALSE | NA | 0 | NA | FALSE | FALSE |
| chr2 | 3720054 | 3720085 | 4 | 3 | NA | FALSE | NA | 0 | 1 | FALSE | FALSE |
| chr2 | 4293503 | 4293533 | 5 | 5 | NA | FALSE | NA | 0 | 1 | FALSE | FALSE |
| chr2 | 4465217 | 4465243 | 5 | 4 | (CA)n | TRUE | NA | 0 | NA | FALSE | FALSE |
| chr2 | 4534222 | 4534223 | 5 | 4 | HERVL-int | TRUE | NA | 0 | 1 | FALSE | FALSE |
| chr2 | 4590567 | 4590578 | 4 | 3 | THE1B | TRUE | NA | 0 | 1 | FALSE | FALSE |
| chr2 | 4718787 | 4718804 | 4 | 3 | L1MEf | TRUE | NA | 0 | 1 | FALSE | FALSE |
| chr2 | 5628247 | 5628261 | 4 | 4 | (GA)n | TRUE | NA | 0 | NA | FALSE | FALSE |
| chr2 | 5780701 | 5780711 | 4 | 4 | NA | FALSE | NA | 0 | 1 | FALSE | FALSE |
| chr2 | 5782922 | 5782936 | 9 | 8 | L1M7 | TRUE | NA | 0 | 1 | FALSE | FALSE |
| chr2 | 5859743 | 5859760 | 4 | 3 | Tigger15a | TRUE | NA | 0 | 1 | FALSE | FALSE |
| chr2 | 5902733 | 5902743 | 4 | 4 | NA | FALSE | NA | 0 | 1 | FALSE | FALSE |
| chr2 | 5914828 | 5914830 | 4 | 4 | NA | FALSE | NA | 0 | 1 | FALSE | FALSE |
| chr2 | 6299819 | 6299834 | 5 | 4 | NA | FALSE | NA | 0 | 1 | TRUE | FALSE |
| chr2 | 6548491 | 6548508 | 4 | 4 | T-rich | TRUE | NA | 0 | NA | FALSE | FALSE |
| chr2 | 6673608 | 6673630 | 4 | 3 | NA | FALSE | NA | 0 | 1 | FALSE | FALSE |
| chr2 | 6717443 | 6717459 | 4 | 3 | NA | FALSE | NA | 0 | 1 | TRUE | FALSE |
| chr2 | 6865338 | 6865366 | 4 | 3 | NA | FALSE | NA | 0 | 1 | FALSE | FALSE |
| chr2 | 7388951 | 7388957 | 5 | 2 | MANY | TRUE | NA | 0 | NA | FALSE | FALSE |
| chr2 | 8118892 | 8118929 | 4 | 3 | NA | FALSE | NA | 0 | 1 | TRUE | FALSE |
| chr2 | 9280306 | 9280353 | 4 | 2 | NA | FALSE | NA | 0 | 1 | TRUE | FALSE |
| chr2 | 10628367 | 10628425 | 5 | 3 | NA | FALSE | NA | 0 | NA | FALSE | FALSE |
| chr2 | 10952937 | 10952973 | 4 | 3 | GC rich | TRUE | MANY | 27 | 1 | TRUE | TRUE |
| chr2 | 11063112 | 11063166 | 4 | 3 | L2b | TRUE | NA | 0 | 1 | TRUE | FALSE |
| chr2 | 11132925 | 11132941 | 4 | 3 | NA | FALSE | NA | 0 | 1 | FALSE | FALSE |
| chr2 | 11142642 | 11142698 | 4 | 3 | L2b | TRUE | NA | 0 | 1 | TRUE | FALSE |
| chr2 | 11899834 | 11899846 | 5 | 4 | SVA C | TRUE | NA | 0 | NA | FALSE | FALSE |
| chr2 | 12063670 | 12063699 | 5 | 4 | AluY | TRUE | NA | 0 | NA | FALSE | FALSE |
| chr2 | 12263186 | 12263232 | 5 | 5 | (TA)n | TRUE | NA | 0 | NA | FALSE | FALSE |
| chr2 | 12610833 | 12610869 | 4 | 3 | AluSp | TRUE | NA | 0 | NA | FALSE | FALSE |
| chr2 | 12822634 | 12822661 | 4 | 3 | GA-rich | TRUE | NA | 0 | 1 | FALSE | FALSE |
| chr2 | 13241635 | 13241653 | 4 | 4 | (TG)n | TRUE | NA | 0 | NA | TRUE | FALSE |
| chr2 | 13513996 | 13513996 | 4 | 4 | NA | FALSE | NA | 0 | 1 | FALSE | FALSE |
| chr2 | 13580811 | 13580821 | 4 | 3 | L1PA5 | TRUE | NA | 0 | 1 | FALSE | FALSE |
| chr2 | 13665872 | 13665873 | 4 | 3 | MLT1H | TRUE | NA | 0 | 1 | FALSE | FALSE |
| chr2 | 13836975 | 13836975 | 4 | 4 | NA | FALSE | NA | 0 | 1 | TRUE | FALSE |
| chr2 | 14190924 | 14190924 | 4 | 4 | NA | FALSE | NA | 0 | 1 | TRUE | FALSE |
| chr2 | 14655065 | 14655081 | 4 | 4 | NA | FALSE | NA | 0 | 1 | FALSE | FALSE |
| chr2 | 14768416 | 14768431 | 4 | 4 | NA | FALSE | NA | 0 | 1 | FALSE | FALSE |
| chr2 | 14837756 | 14837774 | 4 | 4 | NA | FALSE | NA | 0 | 1 | FALSE | FALSE |
| chr2 | 14861940 | 14861940 | 4 | 4 | NA | FALSE | NA | 0 | 1 | FALSE | FALSE |
| chr2 | 15247732 | 15247736 | 4 | 4 | NA | FALSE | NA | 0 | 1 | FALSE | FALSE |
| chr2 | 15820262 | 15820305 | 7 | 7 | SVA D | TRUE | NA | 0 | NA | FALSE | FALSE |
| chr2 | 15820358 | 15820364 | 4 | 3 | SVA D | TRUE | NA | 0 | NA | FALSE | FALSE |
| chr2 | 15946580 | 15946581 | 4 | 3 | NA | FALSE | NA | 0 | 1 | TRUE | FALSE |
| chr2 | 16001876 | 16001884 | 4 | 4 | NA | FALSE | NA | 0 | 1 | FALSE | FALSE |
| chr2 | 16095360 | 16095381 | 4 | 3 | AluSp | TRUE | NA | 0 | 1 | FALSE | FALSE |
| chr2 | 16179755 | 16179778 | 6 | 4 | CT-rich | TRUE | NA | 0 | 1 | FALSE | FALSE |
| chr2 | 16278804 | 16278825 | 4 | 4 | NA | FALSE | NA | 0 | 1 | FALSE | FALSE |
| chr2 | 16488098 | 16488101 | 4 | 3 | NA | FALSE | NA | 0 | 1 | FALSE | FALSE |
| chr2 | 16905173 | 16905191 | 4 | 4 | NA | FALSE | NA | 0 | 1 | FALSE | FALSE |
| chr2 | 17303984 | 17303995 | 4 | 4 | L2c | TRUE | NA | 0 | 1 | FALSE | FALSE |
| chr2 | 18053446 | 18053475 | 5 | 4 | T-rich | TRUE | NA | 0 | NA | FALSE | FALSE |
| chr2 | 18676703 | 18676722 | 4 | 3 | CT-rich | TRUE | NA | 0 | 1 | FALSE | FALSE |
| chr2 | 18839852 | 18839879 | 4 | 2 | NA | FALSE | NA | 0 | 1 | FALSE | FALSE |
| chr2 | 18876373 | 18876380 | 4 | 2 | L1PA4 | TRUE | NA | 0 | NA | FALSE | FALSE |
| chr2 | 19692250 | 19692274 | 4 | 4 | NA | FALSE | NA | 0 | 1 | FALSE | FALSE |
| chr2 | 19815716 | 19815733 | 6 | 5 | MER11A | TRUE | NA | 0 | NA | FALSE | FALSE |
| chr2 | 19842978 | 19843000 | 5 | 4 | NA | FALSE | PU.1 | 1 | 1 | TRUE | FALSE |
| chr2 | 19957508 | 19957510 | 4 | 3 | NA | FALSE | NA | 0 | 1 | TRUE | FALSE |
| chr2 | 21347390 | 21347424 | 8 | 4 | NA | FALSE | NA | 0 | 1 | FALSE | FALSE |
| chr2 | 21626218 | 21626234 | 4 | 3 | MLT1A0 | TRUE | GATA-2 | 1 | 1 | TRUE | FALSE |
| chr2 | 22109819 | 22109826 | 4 | 3 | NA | FALSE | NA | 0 | 1 | FALSE | FALSE |
| chr2 | 22351054 | 22351066 | 6 | 4 | PRIMA4 LTR | TRUE | NA | 0 | 1 | FALSE | FALSE |
| chr2 | 22422698 | 22422715 | 6 | 5 | MANY | TRUE | NA | 0 | NA | FALSE | FALSE |
| chr2 | 23335542 | 23335579 | 4 | 2 | NA | FALSE | MANY | 2 | 1 | FALSE | FALSE |
| chr2 | 24668315 | 24668352 | 4 | 3 | AluY | TRUE | NA | 0 | 1 | FALSE | FALSE |
| chr2 | 26101487 | 26101491 | 7 | 7 | NA | FALSE | MANY | 34 | 1 | TRUE | TRUE |
| chr2 | 27704320 | 27704322 | 4 | 3 | (CA)n | TRUE | NA | 0 | NA | FALSE | FALSE |
| chr2 | 27776652 | 27776695 | 4 | 2 | AluSq | TRUE | NA | 0 | NA | FALSE | FALSE |
| chr2 | 27972008 | 27972060 | 4 | 4 | NA | FALSE | MANY | 2 | 1 | TRUE | FALSE |
| chr2 | 28035051 | 28035130 | 6 | 6 | L1PA5 | TRUE | NA | 0 | 1 | FALSE | FALSE |
| chr2 | 28756831 | 28756897 | 4 | 3 | L1ME3C | TRUE | NA | 0 | 1 | FALSE | FALSE |
| chr2 | 30311982 | 30312012 | 4 | 2 | AluSp | TRUE | NA | 0 | NA | FALSE | FALSE |
| chr2 | 31229337 | 31229356 | 4 | 3 | MIR | TRUE | NA | 0 | 1 | FALSE | FALSE |
| chr2 | 32390871 | 32390942 | 6 | 6 | NA | FALSE | MANY | 61 | 1 | TRUE | TRUE |
| chr2 | 33175442 | 33175500 | 4 | 3 | AluSz6 | TRUE | MANY | 7 | NA | FALSE | FALSE |
| chr2 | 35042004 | 35042042 | 5 | 5 | (CA)n | TRUE | NA | 0 | NA | FALSE | FALSE |
| chr2 | 35048611 | 35048641 | 4 | 3 | L1ME3 | TRUE | NA | 0 | 1 | FALSE | FALSE |
| chr2 | 35499376 | 35499384 | 5 | 3 | L1MA6 | TRUE | NA | 0 | 1 | FALSE | FALSE |
| chr2 | 36077540 | 36077552 | 4 | 3 | NA | FALSE | NA | 0 | 1 | FALSE | FALSE |
| chr2 | 36922029 | 36922050 | 4 | 2 | AluSx1 | TRUE | NA | 0 | NA | FALSE | FALSE |
| chr2 | 37154060 | 37154209 | 6 | 6 | NA | FALSE | MANY | 2 | 1 | TRUE | FALSE |
| chr2 | 38198713 | 38198742 | 4 | 4 | L1M5 | TRUE | NA | 0 | 1 | FALSE | FALSE |
| chr2 | 38902660 | 38902684 | 4 | 3 | AluY | TRUE | NA | 0 | NA | TRUE | FALSE |
| chr2 | 38940636 | 38940712 | 5 | 3 | NA | FALSE | PU.1 | 1 | 1 | FALSE | FALSE |
| chr2 | 38988063 | 38988074 | 4 | 2 | FLAM C | TRUE | NA | 0 | 1 | FALSE | FALSE |
| chr2 | 39129596 | 39129644 | 4 | 3 | MIR3 | TRUE | NA | 0 | 1 | TRUE | FALSE |
| chr2 | 40056676 | 40056696 | 4 | 4 | L1PA7 | TRUE | NA | 0 | NA | FALSE | FALSE |
| chr2 | 40627247 | 40627275 | 4 | 3 | LTR2 | TRUE | NA | 0 | NA | FALSE | FALSE |
| chr2 | 41312707 | 41312734 | 5 | 3 | HSMAR2 | TRUE | NA | 0 | 1 | FALSE | FALSE |
| chr2 | 41924587 | 41924589 | 4 | 3 | NA | FALSE | NA | 0 | 1 | FALSE | FALSE |
| chr2 | 42002000 | 42002040 | 4 | 3 | AluSp | TRUE | NA | 0 | NA | FALSE | FALSE |
| chr2 | 42084730 | 42084740 | 4 | 2 | NA | FALSE | NA | 0 | 1 | TRUE | FALSE |
| chr2 | 42681767 | 42681775 | 6 | 3 | AluSx1 | TRUE | NA | 0 | NA | FALSE | FALSE |
| chr2 | 43274008 | 43274036 | 9 | 8 | (TC)n | TRUE | MANY | 5 | 1 | TRUE | TRUE |
| chr2 | 43422791 | 43422802 | 4 | 2 | (TTTC)n | TRUE | NA | 0 | NA | FALSE | FALSE |
| chr2 | 43524991 | 43525051 | 4 | 4 | SVA F | TRUE | NA | 0 | NA | FALSE | FALSE |
| chr2 | 43959478 | 43959535 | 5 | 3 | MANY | TRUE | NA | 0 | 1 | FALSE | FALSE |
| chr2 | 44964892 | 44964902 | 4 | 4 | (TC)n | TRUE | NA | 0 | NA | FALSE | FALSE |
| chr2 | 45257263 | 45257295 | 4 | 3 | MER53 | TRUE | NA | 0 | 1 | TRUE | FALSE |
| chr2 | 45537979 | 45538026 | 4 | 3 | NA | FALSE | NA | 0 | 1 | TRUE | FALSE |
| chr2 | 45538250 | 45538282 | 4 | 3 | NA | FALSE | NA | 0 | 1 | TRUE | FALSE |
| chr2 | 46442375 | 46442418 | 4 | 3 | L1M1 | TRUE | NA | 0 | NA | FALSE | FALSE |
| chr2 | 47016546 | 47016567 | 4 | 3 | Ricksha c | TRUE | NA | 0 | 1 | FALSE | FALSE |
| chr2 | 48133701 | 48133743 | 5 | 3 | (CGGGG)n | TRUE | MANY | 19 | 1 | FALSE | FALSE |
| chr2 | 50940803 | 50940808 | 4 | 2 | NA | FALSE | NA | 0 | 1 | FALSE | FALSE |
| chr2 | 50959011 | 50959025 | 4 | 4 | NA | FALSE | NA | 0 | 1 | FALSE | FALSE |
| chr2 | 51018803 | 51018803 | 4 | 4 | NA | FALSE | NA | 0 | 1 | FALSE | FALSE |
| chr2 | 51277409 | 51277427 | 4 | 4 | NA | FALSE | NA | 0 | 1 | FALSE | FALSE |
| chr2 | 51659495 | 51659512 | 4 | 3 | MANY | TRUE | NA | 0 | 1 | FALSE | FALSE |
| chr2 | 51672180 | 51672180 | 4 | 4 | NA | FALSE | NA | 0 | 1 | FALSE | FALSE |
| chr2 | 52250821 | 52250826 | 4 | 4 | HERVH-int | TRUE | NA | 0 | NA | FALSE | FALSE |
| chr2 | 52745799 | 52745833 | 4 | 4 | NA | FALSE | NA | 0 | 1 | TRUE | FALSE |
| chr2 | 52872089 | 52872124 | 4 | 2 | MLT1D-int | TRUE | NA | 0 | 1 | TRUE | FALSE |
| chr2 | 53276896 | 53276918 | 4 | 3 | L1PA4 | TRUE | NA | 0 | NA | FALSE | FALSE |
| chr2 | 53402699 | 53402750 | 4 | 3 | NA | FALSE | NA | 0 | 1 | FALSE | FALSE |
| chr2 | 54189276 | 54189344 | 7 | 5 | LTR10C | TRUE | NA | 0 | 1 | TRUE | FALSE |
| chr2 | 55459465 | 55459515 | 4 | 3 | NA | FALSE | MANY | 26 | 1 | TRUE | TRUE |
| chr2 | 55496363 | 55496433 | 4 | 3 | NA | FALSE | MANY | 73 | 1 | TRUE | TRUE |
| chr2 | 57000179 | 57000195 | 4 | 3 | L1PA13 | TRUE | NA | 0 | 1 | FALSE | FALSE |
| chr2 | 57065890 | 57065917 | 4 | 3 | L1M4 | TRUE | NA | 0 | 1 | FALSE | FALSE |
| chr2 | 57458954 | 57458960 | 4 | 2 | HERVH-int | TRUE | NA | 0 | NA | TRUE | FALSE |
| chr2 | 57569986 | 57570018 | 4 | 4 | NA | FALSE | NA | 0 | 1 | FALSE | FALSE |
| chr2 | 57806702 | 57806733 | 4 | 3 | NA | FALSE | NA | 0 | 1 | TRUE | FALSE |
| chr2 | 59134202 | 59134229 | 4 | 3 | NA | FALSE | NA | 0 | 1 | FALSE | FALSE |
| chr2 | 59206162 | 59206182 | 4 | 3 | NA | FALSE | NA | 0 | 1 | FALSE | FALSE |
| chr2 | 59310825 | 59310826 | 4 | 2 | NA | FALSE | GATA-1 | 1 | 1 | FALSE | FALSE |
| chr2 | 59402593 | 59402614 | 5 | 4 | NA | FALSE | NA | 0 | 1 | FALSE | FALSE |
| chr2 | 59680067 | 59680094 | 5 | 4 | AluY | TRUE | NA | 0 | NA | FALSE | FALSE |
| chr2 | 59909800 | 59909814 | 4 | 4 | L1PA5 | TRUE | NA | 0 | NA | FALSE | FALSE |
| chr2 | 60080662 | 60080692 | 4 | 4 | (TAGA)n | TRUE | NA | 0 | NA | FALSE | FALSE |
| chr2 | 60406004 | 60406036 | 4 | 4 | NA | FALSE | NA | 0 | 1 | FALSE | FALSE |
| chr2 | 60585440 | 60585460 | 4 | 4 | NA | FALSE | NA | 0 | 1 | FALSE | FALSE |
| chr2 | 60645675 | 60645726 | 6 | 5 | HERVH-int | TRUE | NA | 0 | NA | FALSE | FALSE |
| chr2 | 60874901 | 60874930 | 4 | 3 | NA | FALSE | NA | 0 | 1 | FALSE | FALSE |
| chr2 | 61264134 | 61264211 | 5 | 3 | L1PA6 | TRUE | NA | 0 | NA | FALSE | FALSE |
| chr2 | 61622814 | 61622907 | 4 | 4 | AluYd8 | TRUE | NA | 0 | NA | FALSE | FALSE |
| chr2 | 61746196 | 61746246 | 5 | 2 | AluSg | TRUE | NA | 0 | NA | FALSE | FALSE |
| chr2 | 61833906 | 61833978 | 4 | 3 | FAM | TRUE | NA | 0 | 1 | TRUE | FALSE |
| chr2 | 61852755 | 61852823 | 4 | 4 | MER33 | TRUE | NA | 0 | 1 | FALSE | FALSE |
| chr2 | 62662285 | 62662343 | 8 | 8 | (TG)n | TRUE | NA | 0 | 1 | FALSE | FALSE |
| chr2 | 62747992 | 62748050 | 4 | 2 | L1PA5 | TRUE | NA | 0 | NA | FALSE | FALSE |
| chr2 | 62798219 | 62798276 | 4 | 2 | NA | FALSE | Pol2 | 1 | 1 | TRUE | FALSE |
| chr2 | 62856855 | 62856902 | 6 | 4 | SVA C | TRUE | NA | 0 | 1 | FALSE | FALSE |
| chr2 | 63635344 | 63635413 | 4 | 3 | L1PB1 | TRUE | NA | 0 | 1 | FALSE | FALSE |
| chr2 | 64990069 | 64990130 | 4 | 4 | NA | FALSE | NA | 0 | 1 | TRUE | FALSE |
| chr2 | 65125471 | 65125494 | 4 | 2 | HERVE-int | TRUE | NA | 0 | NA | FALSE | FALSE |
| chr2 | 65566043 | 65566072 | 4 | 3 | SVA D | TRUE | NA | 0 | NA | FALSE | FALSE |
| chr2 | 65828031 | 65828062 | 5 | 5 | SVA D | TRUE | NA | 0 | NA | FALSE | FALSE |
| chr2 | 65877992 | 65878033 | 4 | 4 | NA | FALSE | NA | 0 | 1 | FALSE | FALSE |
| chr2 | 66990164 | 66990190 | 4 | 4 | (TATATG)n | TRUE | NA | 0 | 1 | FALSE | FALSE |
| chr2 | 67559714 | 67559747 | 4 | 3 | HERVH-int | TRUE | NA | 0 | 1 | FALSE | FALSE |
| chr2 | 68616629 | 68616695 | 4 | 4 | NA | FALSE | NA | 0 | 1 | FALSE | FALSE |
| chr2 | 68945682 | 68945689 | 4 | 2 | MANY | TRUE | NA | 0 | 1 | FALSE | FALSE |
| chr2 | 70056750 | 70056763 | 7 | 5 | NA | FALSE | MANY | 32 | 1 | TRUE | TRUE |
| chr2 | 70850391 | 70850416 | 10 | 6 | HERVH-int | TRUE | NA | 0 | NA | FALSE | FALSE |
| chr2 | 71254362 | 71254392 | 4 | 3 | SATR2 | TRUE | NA | 0 | 1 | FALSE | FALSE |
| chr2 | 72325584 | 72325608 | 4 | 4 | L1ME1 | TRUE | NA | 0 | 1 | FALSE | FALSE |
| chr2 | 72381900 | 72381902 | 4 | 3 | (TG)n | TRUE | NA | 0 | NA | TRUE | FALSE |
| chr2 | 72421725 | 72421725 | 4 | 4 | (TC)n | TRUE | NA | 0 | NA | FALSE | FALSE |
| chr2 | 72635648 | 72635689 | 4 | 3 | L1PA4 | TRUE | NA | 0 | NA | FALSE | FALSE |
| chr2 | 72739499 | 72739531 | 4 | 4 | (GGAA)n | TRUE | NA | 0 | NA | TRUE | FALSE |
| chr2 | 73771606 | 73771616 | 4 | 2 | L1PA4 | TRUE | NA | 0 | NA | FALSE | FALSE |
| chr2 | 73788358 | 73788472 | 8 | 7 | LTR76 | TRUE | NA | 0 | 1 | TRUE | FALSE |
| chr2 | 73948767 | 73948830 | 5 | 3 | L1PA3 | TRUE | NA | 0 | NA | FALSE | FALSE |
| chr2 | 73964560 | 73964606 | 4 | 3 | NA | FALSE | MANY | 46 | 1 | TRUE | TRUE |
| chr2 | 74788255 | 74788268 | 6 | 5 | (GGA)n | TRUE | ZNF263 | 1 | NA | FALSE | FALSE |
| chr2 | 75485229 | 75485263 | 4 | 4 | L1MB7 | TRUE | NA | 0 | 1 | FALSE | FALSE |
| chr2 | 76086031 | 76086055 | 4 | 3 | NA | FALSE | NA | 0 | 1 | FALSE | FALSE |
| chr2 | 76317827 | 76317843 | 4 | 3 | L1PA3 | TRUE | NA | 0 | NA | FALSE | FALSE |
| chr2 | 76609203 | 76609224 | 5 | 5 | NA | FALSE | NA | 0 | 1 | FALSE | FALSE |
| chr2 | 77033690 | 77033700 | 4 | 4 | AluSq2 | TRUE | NA | 0 | 1 | FALSE | FALSE |
| chr2 | 77051338 | 77051355 | 4 | 3 | LTR37A | TRUE | NA | 0 | 1 | FALSE | FALSE |
| chr2 | 77589244 | 77589274 | 4 | 2 | (GAAA)n | TRUE | NA | 0 | NA | FALSE | FALSE |
| chr2 | 78383997 | 78384041 | 4 | 3 | MLT2D | TRUE | NA | 0 | 1 | FALSE | FALSE |
| chr2 | 78783708 | 78783751 | 4 | 4 | L1PB | TRUE | NA | 0 | 1 | FALSE | FALSE |
| chr2 | 79956026 | 79956052 | 5 | 3 | L1PA3 | TRUE | NA | 0 | NA | FALSE | FALSE |
| chr2 | 80396412 | 80396442 | 4 | 4 | MER115 | TRUE | NA | 0 | 1 | FALSE | FALSE |
| chr2 | 80607277 | 80607304 | 5 | 2 | AluY | TRUE | NA | 0 | NA | FALSE | FALSE |
| chr2 | 80823658 | 80823674 | 4 | 4 | AluY | TRUE | NA | 0 | NA | FALSE | FALSE |
| chr2 | 81113580 | 81113587 | 4 | 3 | NA | FALSE | NA | 0 | 1 | FALSE | FALSE |
| chr2 | 81267784 | 81267784 | 5 | 5 | LTR12 | TRUE | NA | 0 | NA | FALSE | FALSE |
| chr2 | 81322182 | 81322203 | 5 | 5 | NA | FALSE | NA | 0 | 1 | FALSE | FALSE |
| chr2 | 81496818 | 81496824 | 4 | 4 | L1PA8 | TRUE | NA | 0 | NA | FALSE | FALSE |
| chr2 | 81647560 | 81647565 | 4 | 4 | THE1B | TRUE | NA | 0 | 1 | FALSE | FALSE |
| chr2 | 81702469 | 81702483 | 4 | 4 | L1M5 | TRUE | NA | 0 | 1 | FALSE | FALSE |
| chr2 | 81748102 | 81748108 | 4 | 2 | NA | FALSE | NA | 0 | 1 | FALSE | FALSE |
| chr2 | 81901154 | 81901164 | 4 | 4 | NA | FALSE | NA | 0 | 1 | FALSE | FALSE |
| chr2 | 82640315 | 82640317 | 5 | 5 | MANY | TRUE | NA | 0 | NA | FALSE | FALSE |
| chr2 | 82710104 | 82710114 | 4 | 4 | CT-rich | TRUE | NA | 0 | 1 | FALSE | FALSE |
| chr2 | 83114240 | 83114240 | 4 | 4 | (TA)n | TRUE | NA | 0 | NA | FALSE | FALSE |
| chr2 | 83393322 | 83393335 | 4 | 4 | MER33 | TRUE | NA | 0 | 1 | FALSE | FALSE |
| chr2 | 83490623 | 83490651 | 5 | 4 | CT-rich | TRUE | NA | 0 | NA | FALSE | FALSE |
| chr2 | 83775854 | 83775870 | 4 | 3 | MIRb | TRUE | NA | 0 | 1 | FALSE | FALSE |
| chr2 | 83967101 | 83967120 | 4 | 3 | THE1C-int | TRUE | NA | 0 | 1 | FALSE | FALSE |
| chr2 | 84276993 | 84277001 | 4 | 3 | NA | FALSE | NA | 0 | 1 | FALSE | FALSE |
| chr2 | 84323847 | 84323848 | 4 | 3 | L1MA8 | TRUE | NA | 0 | NA | FALSE | FALSE |
| chr2 | 85026081 | 85026083 | 4 | 4 | L1P4d | TRUE | NA | 0 | 1 | TRUE | FALSE |
| chr2 | 85026876 | 85026883 | 4 | 4 | L1P4d | TRUE | NA | 0 | 1 | FALSE | FALSE |
| chr2 | 85030272 | 85030278 | 4 | 3 | L1P4d | TRUE | NA | 0 | 1 | FALSE | FALSE |
| chr2 | 85055524 | 85055564 | 4 | 3 | NA | FALSE | NA | 0 | 1 | TRUE | FALSE |
| chr2 | 85796955 | 85797039 | 4 | 3 | AluSg7 | TRUE | NA | 0 | 1 | FALSE | FALSE |
| chr2 | 85904830 | 85904876 | 6 | 2 | AluSx1 | TRUE | NA | 0 | 1 | FALSE | FALSE |
| chr2 | 86499845 | 86499952 | 5 | 4 | L2 | TRUE | NA | 0 | 1 | TRUE | FALSE |
| chr2 | 86513323 | 86513396 | 4 | 2 | MER20 | TRUE | NA | 0 | 1 | FALSE | FALSE |
| chr2 | 86772584 | 86772585 | 4 | 2 | SVA A | TRUE | NA | 0 | NA | FALSE | FALSE |
| chr2 | 86868358 | 86868372 | 4 | 2 | L1ME1 | TRUE | NA | 0 | 1 | FALSE | FALSE |
| chr2 | 87369782 | 87369833 | 4 | 3 | NA | FALSE | NA | 0 | 1 | TRUE | FALSE |
| chr2 | 87592904 | 87592911 | 5 | 5 | (AAATG)n | TRUE | NA | 0 | 1 | FALSE | FALSE |
| chr2 | 87627367 | 87627407 | 6 | 6 | MANY | TRUE | NA | 0 | NA | FALSE | FALSE |
| chr2 | 87645960 | 87646000 | 4 | 4 | (CAGC)n | TRUE | NA | 0 | 1 | FALSE | FALSE |
| chr2 | 88046277 | 88046280 | 4 | 3 | NA | FALSE | NA | 0 | 1 | FALSE | FALSE |
| chr2 | 88451136 | 88451168 | 9 | 5 | L1PA4 | TRUE | NA | 0 | NA | FALSE | FALSE |
| chr2 | 88584989 | 88585000 | 4 | 2 | NA | FALSE | NA | 0 | 1 | TRUE | FALSE |
| chr2 | 89075971 | 89075979 | 5 | 5 | NA | FALSE | NA | 0 | NA | FALSE | FALSE |
| chr2 | 89298247 | 89298267 | 4 | 2 | MER70A | TRUE | NA | 0 | 1 | FALSE | FALSE |
| chr2 | 90413501 | 90413532 | 5 | 3 | NA | FALSE | Egr-1 | 1 | NA | TRUE | FALSE |
| chr2 | 90417161 | 90417187 | 4 | 4 | (AAATG)n | TRUE | NA | 0 | NA | FALSE | FALSE |
| chr2 | 90417313 | 90417328 | 5 | 3 | (AAATG)n | TRUE | NA | 0 | 1 | FALSE | FALSE |
| chr2 | 91629624 | 91629691 | 10 | 8 | AT rich | TRUE | NA | 0 | 1 | FALSE | FALSE |
| chr2 | 91740208 | 91740227 | 4 | 4 | (CATCT)n | TRUE | NA | 0 | NA | FALSE | FALSE |
| chr2 | 91763305 | 91763317 | 4 | 4 | NA | FALSE | NA | 0 | 1 | TRUE | FALSE |
| chr2 | 91764265 | 91764272 | 4 | 4 | NA | FALSE | NA | 0 | 1 | TRUE | FALSE |
| chr2 | 91765522 | 91765541 | 4 | 4 | AluJb | TRUE | NA | 0 | NA | TRUE | FALSE |
| chr2 | 91770578 | 91770599 | 4 | 4 | HSMAR2 | TRUE | MANY | 4 | 1 | TRUE | TRUE |
| chr2 | 91776932 | 91776951 | 4 | 4 | NA | FALSE | TAF1 | 1 | 1 | TRUE | FALSE |
| chr2 | 91780032 | 91780045 | 5 | 3 | CT-rich | TRUE | NA | 0 | 1 | FALSE | FALSE |
| chr2 | 91782461 | 91782476 | 4 | 3 | L2c | TRUE | NA | 0 | 1 | TRUE | FALSE |
| chr2 | 91790440 | 91790453 | 4 | 4 | L1MB3 | TRUE | NA | 0 | 1 | FALSE | FALSE |
| chr2 | 91792439 | 91792453 | 4 | 3 | MANY | TRUE | NA | 0 | NA | FALSE | FALSE |
| chr2 | 91801206 | 91801222 | 5 | 5 | L1PA16 | TRUE | NA | 0 | 1 | FALSE | FALSE |
| chr2 | 91809075 | 91809089 | 4 | 3 | NA | FALSE | NA | 0 | NA | TRUE | FALSE |
| chr2 | 91976393 | 91976413 | 6 | 4 | NA | FALSE | NA | 0 | NA | FALSE | FALSE |
| chr2 | 91987400 | 91987411 | 4 | 4 | NA | FALSE | NA | 0 | NA | FALSE | FALSE |
| chr2 | 92078383 | 92078383 | 5 | 5 | NA | FALSE | NA | 0 | NA | FALSE | FALSE |
| chr2 | 92091853 | 92091868 | 5 | 5 | L1PA5 | TRUE | NA | 0 | NA | FALSE | FALSE |
| chr2 | 92109086 | 92109103 | 4 | 4 | L1PB1 | TRUE | NA | 0 | NA | TRUE | FALSE |
| chr2 | 92123976 | 92123995 | 4 | 4 | L1MEd | TRUE | NA | 0 | NA | TRUE | FALSE |
| chr2 | 92132865 | 92132881 | 4 | 3 | MSTB2-int | TRUE | NA | 0 | 1 | FALSE | FALSE |
| chr2 | 92177821 | 92177822 | 5 | 5 | (AAATG)n | TRUE | NA | 0 | 1 | FALSE | FALSE |
| chr2 | 92178040 | 92178066 | 5 | 5 | (AAATG)n | TRUE | NA | 0 | NA | FALSE | FALSE |
| chr2 | 92191926 | 92191926 | 6 | 6 | NA | FALSE | NA | 0 | NA | FALSE | FALSE |
| chr2 | 92194104 | 92194146 | 11 | 8 | AluSq2 | TRUE | NA | 0 | NA | FALSE | FALSE |
| chr2 | 92194637 | 92194654 | 5 | 4 | NA | FALSE | NA | 0 | 1 | FALSE | FALSE |
| chr2 | 92241562 | 92241562 | 4 | 4 | CT-rich | TRUE | NA | 0 | NA | FALSE | FALSE |
| chr2 | 92259902 | 92259922 | 5 | 5 | L1M5 | TRUE | NA | 0 | NA | FALSE | FALSE |
| chr2 | 92263689 | 92263714 | 5 | 5 | GA-rich | TRUE | NA | 0 | 1 | TRUE | FALSE |
| chr2 | 92265890 | 92265902 | 4 | 3 | MER77 | TRUE | NA | 0 | NA | FALSE | FALSE |
| chr2 | 95362709 | 95362758 | 4 | 4 | L1MEg | TRUE | NA | 0 | 1 | FALSE | FALSE |
| chr2 | 95373420 | 95373463 | 4 | 4 | L1PREC2 | TRUE | NA | 0 | 1 | FALSE | FALSE |
| chr2 | 95383496 | 95383506 | 6 | 4 | AluSc8 | TRUE | NA | 0 | NA | FALSE | FALSE |
| chr2 | 95448123 | 95448215 | 10 | 8 | MSTA-int | TRUE | NA | 0 | 1 | TRUE | FALSE |
| chr2 | 95473501 | 95473503 | 4 | 2 | NA | FALSE | NA | 0 | 1 | FALSE | FALSE |
| chr2 | 95478982 | 95479027 | 4 | 2 | NA | FALSE | NA | 0 | 1 | FALSE | FALSE |
| chr2 | 95504609 | 95504627 | 4 | 3 | NA | FALSE | NA | 0 | 1 | FALSE | FALSE |
| chr2 | 95504947 | 95504968 | 4 | 3 | NA | FALSE | NA | 0 | 1 | FALSE | FALSE |
| chr2 | 95520873 | 95520895 | 4 | 3 | NA | FALSE | NA | 0 | NA | FALSE | FALSE |
| chr2 | 95525046 | 95525046 | 4 | 4 | L1ME2z | TRUE | NA | 0 | NA | FALSE | FALSE |
| chr2 | 95527977 | 95528004 | 4 | 4 | NA | FALSE | NA | 0 | 1 | TRUE | FALSE |
| chr2 | 95704347 | 95704408 | 8 | 6 | (TGAG)n | TRUE | MANY | 3 | 1 | FALSE | FALSE |
| chr2 | 96079165 | 96079208 | 4 | 3 | NA | FALSE | NA | 0 | 1 | TRUE | FALSE |
| chr2 | 96090330 | 96090330 | 4 | 4 | NA | FALSE | NA | 0 | 1 | FALSE | FALSE |
| chr2 | 96526978 | 96527016 | 4 | 4 | L1MEc | TRUE | NA | 0 | 1 | FALSE | FALSE |
| chr2 | 96622337 | 96622398 | 7 | 4 | MIRb | TRUE | NA | 0 | NA | FALSE | FALSE |
| chr2 | 96633695 | 96633740 | 4 | 3 | AluSx | TRUE | NA | 0 | 1 | FALSE | FALSE |
| chr2 | 96658898 | 96658899 | 4 | 4 | NA | FALSE | MANY | 3 | 1 | TRUE | TRUE |
| chr2 | 96659963 | 96659963 | 4 | 4 | L3 | TRUE | NA | 0 | 1 | FALSE | FALSE |
| chr2 | 96789921 | 96789974 | 4 | 2 | NA | FALSE | NA | 0 | 1 | TRUE | FALSE |
| chr2 | 96971291 | 96971308 | 5 | 5 | NA | FALSE | MANY | 40 | 1 | TRUE | TRUE |
| chr2 | 97117688 | 97117704 | 4 | 4 | NA | FALSE | NA | 0 | NA | FALSE | FALSE |
| chr2 | 97689211 | 97689221 | 6 | 4 | NA | FALSE | NA | 0 | 1 | TRUE | FALSE |
| chr2 | 97776065 | 97776120 | 6 | 6 | NA | FALSE | NA | 0 | 1 | FALSE | FALSE |
| chr2 | 97803165 | 97803231 | 4 | 2 | MANY | TRUE | NA | 0 | NA | TRUE | FALSE |
| chr2 | 98179564 | 98179594 | 6 | 3 | NA | FALSE | NA | 0 | 1 | TRUE | FALSE |
| chr2 | 98189306 | 98189368 | 5 | 4 | NA | FALSE | NA | 0 | 1 | FALSE | FALSE |
| chr2 | 98257006 | 98257028 | 5 | 4 | AluJo | TRUE | NA | 0 | 1 | FALSE | FALSE |
| chr2 | 98509518 | 98509581 | 4 | 3 | L1PA7 | TRUE | NA | 0 | 1 | FALSE | FALSE |
| chr2 | 98555773 | 98555782 | 8 | 4 | L1PA5 | TRUE | NA | 0 | NA | FALSE | FALSE |
| chr2 | 98690321 | 98690394 | 5 | 4 | MLT1A0 | TRUE | NA | 0 | 1 | FALSE | FALSE |
| chr2 | 99680350 | 99680368 | 4 | 2 | L1ME3C | TRUE | NA | 0 | 1 | FALSE | FALSE |
| chr2 | 100597534 | 100597566 | 5 | 2 | AluSp | TRUE | NA | 0 | NA | FALSE | FALSE |
| chr2 | 101098552 | 101098597 | 4 | 3 | NA | FALSE | NA | 0 | 1 | TRUE | FALSE |
| chr2 | 103769062 | 103769072 | 4 | 4 | NA | FALSE | NA | 0 | 1 | FALSE | FALSE |
| chr2 | 103806998 | 103807016 | 5 | 4 | L1PA13 | TRUE | NA | 0 | 1 | FALSE | FALSE |
| chr2 | 103963687 | 103963697 | 4 | 4 | L1MCa | TRUE | NA | 0 | 1 | FALSE | FALSE |
| chr2 | 104175161 | 104175161 | 4 | 4 | NA | FALSE | NA | 0 | 1 | FALSE | FALSE |
| chr2 | 104263044 | 104263053 | 4 | 3 | (GAAA)n | TRUE | NA | 0 | NA | FALSE | FALSE |
| chr2 | 104350583 | 104350591 | 4 | 3 | L1PA15 | TRUE | NA | 0 | 1 | FALSE | FALSE |
| chr2 | 104973825 | 104973859 | 4 | 3 | L1M5 | TRUE | NA | 0 | 1 | TRUE | FALSE |
| chr2 | 105346540 | 105346540 | 4 | 4 | NA | FALSE | NA | 0 | 1 | FALSE | FALSE |
| chr2 | 105549146 | 105549188 | 4 | 3 | NA | FALSE | NA | 0 | 1 | FALSE | FALSE |
| chr2 | 105618750 | 105618763 | 4 | 2 | NA | FALSE | NA | 0 | 1 | FALSE | FALSE |
| chr2 | 105636682 | 105636724 | 4 | 3 | L1MB3 | TRUE | NA | 0 | 1 | FALSE | FALSE |
| chr2 | 105864889 | 105864901 | 4 | 4 | NA | FALSE | CEBPB | 1 | 1 | TRUE | FALSE |
| chr2 | 106041982 | 106042036 | 5 | 3 | LTR5A | TRUE | NA | 0 | NA | FALSE | FALSE |
| chr2 | 107003043 | 107003054 | 5 | 3 | NA | FALSE | NA | 0 | 1 | FALSE | FALSE |
| chr2 | 107084158 | 107084179 | 4 | 3 | NA | FALSE | NA | 0 | NA | FALSE | FALSE |
| chr2 | 107389186 | 107389192 | 4 | 4 | NA | FALSE | NA | 0 | 1 | FALSE | FALSE |
| chr2 | 107424964 | 107424977 | 4 | 4 | NA | FALSE | NA | 0 | 1 | FALSE | FALSE |
| chr2 | 107521234 | 107521240 | 4 | 3 | L1MDa | TRUE | NA | 0 | 1 | FALSE | FALSE |
| chr2 | 107886942 | 107886942 | 5 | 5 | (TG)n | TRUE | NA | 0 | NA | FALSE | FALSE |
| chr2 | 108344750 | 108344778 | 4 | 4 | L1MD1 | TRUE | NA | 0 | 1 | FALSE | FALSE |
| chr2 | 108371978 | 108372005 | 4 | 4 | MLT1D | TRUE | NA | 0 | 1 | TRUE | FALSE |
| chr2 | 108426672 | 108426689 | 4 | 4 | NA | FALSE | NA | 0 | 1 | FALSE | FALSE |
| chr2 | 108444051 | 108444064 | 5 | 4 | NA | FALSE | NA | 0 | NA | FALSE | FALSE |
| chr2 | 108446596 | 108446606 | 4 | 4 | NA | FALSE | NA | 0 | 1 | TRUE | FALSE |
| chr2 | 108560745 | 108560749 | 4 | 2 | HERVK9-int | TRUE | NA | 0 | 1 | FALSE | FALSE |
| chr2 | 109360830 | 109360872 | 5 | 5 | AluSx1 | TRUE | NA | 0 | NA | FALSE | FALSE |
| chr2 | 109363461 | 109363489 | 6 | 3 | NA | FALSE | NA | 0 | 1 | TRUE | FALSE |
| chr2 | 109367218 | 109367285 | 6 | 4 | AluSx3 | TRUE | NA | 0 | 1 | TRUE | FALSE |
| chr2 | 109543591 | 109543660 | 4 | 4 | NA | FALSE | E2F6 (H-50) | 1 | 1 | TRUE | FALSE |
| chr2 | 109710115 | 109710182 | 4 | 4 | HUERS-P1-int | TRUE | NA | 0 | 1 | FALSE | FALSE |
| chr2 | 109884148 | 109884169 | 5 | 4 | NA | FALSE | NA | 0 | 1 | TRUE | FALSE |
| chr2 | 110078034 | 110078109 | 4 | 4 | ERVL-B4-int | TRUE | NA | 0 | NA | FALSE | FALSE |
| chr2 | 110428417 | 110428507 | 5 | 3 | NA | FALSE | Rad21 | 1 | 1 | TRUE | FALSE |
| chr2 | 110482386 | 110482492 | 4 | 3 | LTR81B | TRUE | NA | 0 | 1 | FALSE | FALSE |
| chr2 | 110910530 | 110910667 | 4 | 4 | L1MDa | TRUE | MANY | 2 | 1 | TRUE | FALSE |
| chr2 | 112172704 | 112172705 | 5 | 3 | MER58A | TRUE | NA | 0 | 1 | FALSE | FALSE |
| chr2 | 112175789 | 112175834 | 4 | 2 | NA | FALSE | MANY | 2 | 1 | TRUE | FALSE |
| chr2 | 112314887 | 112314943 | 5 | 4 | L1MC3 | TRUE | NA | 0 | 1 | FALSE | FALSE |
| chr2 | 112330596 | 112330648 | 4 | 4 | MANY | TRUE | NA | 0 | NA | FALSE | FALSE |
| chr2 | 112591195 | 112591238 | 6 | 5 | MIRc | TRUE | NA | 0 | 1 | FALSE | FALSE |
| chr2 | 112623171 | 112623219 | 7 | 3 | L1ME4a | TRUE | NA | 0 | 1 | FALSE | FALSE |
| chr2 | 112872845 | 112872896 | 4 | 3 | NA | FALSE | NA | 0 | NA | FALSE | FALSE |
| chr2 | 113116617 | 113116717 | 5 | 4 | AluSq2 | TRUE | NA | 0 | 1 | FALSE | FALSE |
| chr2 | 113195988 | 113196009 | 8 | 6 | MLT1D | TRUE | NA | 0 | 1 | FALSE | FALSE |
| chr2 | 113737227 | 113737257 | 5 | 3 | L3 | TRUE | NA | 0 | 1 | FALSE | FALSE |
| chr2 | 113753111 | 113753153 | 5 | 3 | NA | FALSE | NA | 0 | 1 | TRUE | FALSE |
| chr2 | 113783053 | 113783065 | 4 | 3 | NA | FALSE | NA | 0 | 1 | FALSE | FALSE |
| chr2 | 113816345 | 113816372 | 4 | 3 | GA-rich | TRUE | NA | 0 | 1 | TRUE | FALSE |
| chr2 | 113993582 | 113993596 | 4 | 4 | NA | FALSE | NA | 0 | NA | FALSE | FALSE |
| chr2 | 114177614 | 114177634 | 4 | 3 | HAL1 | TRUE | NA | 0 | 1 | FALSE | FALSE |
| chr2 | 114983516 | 114983537 | 4 | 4 | NA | FALSE | NA | 0 | 1 | FALSE | FALSE |
| chr2 | 116766656 | 116766658 | 5 | 4 | (TCTA)n | TRUE | NA | 0 | 1 | FALSE | FALSE |
| chr2 | 116899365 | 116899385 | 9 | 5 | LTR7 | TRUE | NA | 0 | NA | FALSE | FALSE |
| chr2 | 117013231 | 117013251 | 4 | 3 | MER9a2 | TRUE | NA | 0 | NA | FALSE | FALSE |
| chr2 | 117014159 | 117014177 | 4 | 3 | NA | FALSE | NA | 0 | 1 | FALSE | FALSE |
| chr2 | 117191473 | 117191492 | 5 | 5 | THE1B-int | TRUE | NA | 0 | NA | FALSE | FALSE |
| chr2 | 117595306 | 117595324 | 4 | 2 | (CATATA)n | TRUE | NA | 0 | 1 | FALSE | FALSE |
| chr2 | 117812089 | 117812107 | 5 | 4 | AluSq2 | TRUE | NA | 0 | NA | FALSE | FALSE |
| chr2 | 118530686 | 118530722 | 4 | 2 | MER106B | TRUE | NA | 0 | 1 | FALSE | FALSE |
| chr2 | 118617303 | 118617328 | 4 | 3 | NA | FALSE | E2F6 (H-50) | 1 | 1 | TRUE | FALSE |
| chr2 | 118657518 | 118657537 | 5 | 3 | L1PA3 | TRUE | NA | 0 | NA | FALSE | FALSE |
| chr2 | 118851970 | 118851993 | 4 | 3 | NA | FALSE | NA | 0 | 1 | FALSE | FALSE |
| chr2 | 119099147 | 119099163 | 4 | 4 | NA | FALSE | NA | 0 | 1 | TRUE | FALSE |
| chr2 | 119150418 | 119150424 | 4 | 4 | L2b | TRUE | NA | 0 | 1 | FALSE | FALSE |
| chr2 | 119478954 | 119478968 | 4 | 3 | NA | FALSE | NA | 0 | 1 | FALSE | FALSE |
| chr2 | 119490997 | 119491015 | 4 | 4 | MIRb | TRUE | NA | 0 | 1 | FALSE | FALSE |
| chr2 | 119817904 | 119817914 | 4 | 3 | MIRb | TRUE | NA | 0 | 1 | FALSE | FALSE |
| chr2 | 121168647 | 121168657 | 4 | 3 | L1PA10 | TRUE | NA | 0 | NA | FALSE | FALSE |
| chr2 | 122059769 | 122059826 | 5 | 4 | AluSz | TRUE | NA | 0 | NA | FALSE | FALSE |
| chr2 | 122434949 | 122435022 | 4 | 2 | AluY | TRUE | NA | 0 | NA | FALSE | FALSE |
| chr2 | 122458825 | 122458884 | 4 | 3 | AluSq2 | TRUE | NA | 0 | NA | FALSE | FALSE |
| chr2 | 122534536 | 122534578 | 6 | 6 | (TG)n | TRUE | NA | 0 | NA | TRUE | FALSE |
| chr2 | 123092140 | 123092187 | 4 | 4 | NA | FALSE | NA | 0 | 1 | FALSE | FALSE |
| chr2 | 123531921 | 123531928 | 6 | 3 | L1PA8 | TRUE | NA | 0 | NA | FALSE | FALSE |
| chr2 | 124753903 | 124753936 | 4 | 3 | L1PB1 | TRUE | NA | 0 | 1 | FALSE | FALSE |
| chr2 | 125618051 | 125618064 | 5 | 3 | (TTCC)n | TRUE | NA | 0 | NA | FALSE | FALSE |
| chr2 | 126033550 | 126033550 | 4 | 4 | AluY | TRUE | NA | 0 | 1 | FALSE | FALSE |
| chr2 | 126053839 | 126053858 | 4 | 4 | L1M1 | TRUE | NA | 0 | 1 | FALSE | FALSE |
| chr2 | 126096877 | 126096902 | 4 | 4 | LOR1a | TRUE | NA | 0 | 1 | FALSE | FALSE |
| chr2 | 126232833 | 126232852 | 4 | 2 | MLT2B3 | TRUE | NA | 0 | 1 | TRUE | FALSE |
| chr2 | 126302427 | 126302439 | 4 | 4 | NA | FALSE | NA | 0 | 1 | TRUE | FALSE |
| chr2 | 126591443 | 126591447 | 4 | 2 | MLT1C | TRUE | NA | 0 | 1 | FALSE | FALSE |
| chr2 | 127679702 | 127679723 | 4 | 4 | MLT1B | TRUE | NA | 0 | 1 | FALSE | FALSE |
| chr2 | 128615742 | 128615756 | 10 | 7 | NA | FALSE | MANY | 28 | 1 | TRUE | TRUE |
| chr2 | 128739238 | 128739279 | 6 | 5 | LTR21B | TRUE | NA | 0 | NA | TRUE | FALSE |
| chr2 | 129209778 | 129209778 | 4 | 4 | NA | FALSE | NA | 0 | NA | TRUE | FALSE |
| chr2 | 129703179 | 129703203 | 8 | 6 | L1PA5 | TRUE | NA | 0 | NA | FALSE | FALSE |
| chr2 | 130321321 | 130321323 | 4 | 3 | L1ME3A | TRUE | NA | 0 | 1 | FALSE | FALSE |
| chr2 | 130724867 | 130724878 | 4 | 3 | NA | FALSE | NA | 0 | 1 | FALSE | FALSE |
| chr2 | 131025923 | 131025933 | 4 | 4 | NA | FALSE | NA | 0 | NA | TRUE | FALSE |
| chr2 | 131394559 | 131394576 | 4 | 4 | AluYc | TRUE | NA | 0 | NA | FALSE | FALSE |
| chr2 | 131487716 | 131487757 | 4 | 3 | NA | FALSE | NA | 0 | 1 | FALSE | FALSE |
| chr2 | 131778159 | 131778162 | 4 | 3 | L1PA4 | TRUE | NA | 0 | NA | FALSE | FALSE |
| chr2 | 131944764 | 131944773 | 4 | 2 | L1PA4 | TRUE | NA | 0 | NA | FALSE | FALSE |
| chr2 | 131945029 | 131945040 | 4 | 3 | NA | FALSE | CTCF | 1 | 1 | TRUE | FALSE |
| chr2 | 131947688 | 131947707 | 5 | 2 | NA | FALSE | NA | 0 | 1 | FALSE | FALSE |
| chr2 | 131978544 | 131978556 | 4 | 2 | AluSx3 | TRUE | NA | 0 | NA | FALSE | FALSE |
| chr2 | 132046943 | 132046977 | 4 | 3 | NA | FALSE | NA | 0 | 1 | FALSE | FALSE |
| chr2 | 132063228 | 132063261 | 4 | 2 | AluSp | TRUE | NA | 0 | 1 | FALSE | FALSE |
| chr2 | 132068569 | 132068578 | 4 | 2 | NA | FALSE | NA | 0 | NA | FALSE | FALSE |
| chr2 | 132107870 | 132107906 | 4 | 3 | NA | FALSE | NA | 0 | 1 | FALSE | FALSE |
| chr2 | 132499936 | 132499937 | 4 | 4 | NA | FALSE | NA | 0 | NA | FALSE | FALSE |
| chr2 | 132509110 | 132509120 | 6 | 3 | NA | FALSE | NA | 0 | 1 | FALSE | FALSE |
| chr2 | 132618985 | 132619001 | 5 | 5 | NA | FALSE | NA | 0 | 1 | FALSE | FALSE |
| chr2 | 132711347 | 132711362 | 7 | 4 | L1MA4 | TRUE | NA | 0 | NA | FALSE | FALSE |
| chr2 | 132764813 | 132764814 | 6 | 3 | MER77 | TRUE | NA | 0 | 1 | FALSE | FALSE |
| chr2 | 132764934 | 132764951 | 5 | 5 | MER77 | TRUE | NA | 0 | 1 | FALSE | FALSE |
| chr2 | 132790369 | 132790388 | 4 | 3 | NA | FALSE | NA | 0 | NA | FALSE | FALSE |
| chr2 | 132794195 | 132794236 | 13 | 10 | NA | FALSE | NA | 0 | NA | FALSE | FALSE |
| chr2 | 132794460 | 132794464 | 4 | 4 | AluJr | TRUE | NA | 0 | NA | FALSE | FALSE |
| chr2 | 132804171 | 132804183 | 4 | 4 | NA | FALSE | NA | 0 | NA | FALSE | FALSE |
| chr2 | 132820019 | 132820033 | 4 | 4 | L1MEg | TRUE | NA | 0 | NA | FALSE | FALSE |
| chr2 | 132839843 | 132839861 | 4 | 3 | LTR70 | TRUE | NA | 0 | 1 | FALSE | FALSE |
| chr2 | 132858646 | 132858653 | 4 | 2 | AluJo | TRUE | NA | 0 | 1 | FALSE | FALSE |
| chr2 | 132877465 | 132877471 | 4 | 4 | NA | FALSE | NA | 0 | 1 | FALSE | FALSE |
| chr2 | 132902035 | 132902056 | 4 | 3 | HERVIP10F-int | TRUE | NA | 0 | 1 | FALSE | FALSE |
| chr2 | 132929847 | 132929852 | 6 | 4 | L1MB4 | TRUE | NA | 0 | 1 | FALSE | FALSE |
| chr2 | 132942741 | 132942741 | 4 | 4 | NA | FALSE | NA | 0 | NA | FALSE | FALSE |
| chr2 | 132942773 | 132942779 | 5 | 3 | NA | FALSE | NA | 0 | 1 | FALSE | FALSE |
| chr2 | 132951627 | 132951647 | 4 | 4 | MANY | TRUE | NA | 0 | NA | FALSE | FALSE |
| chr2 | 132960401 | 132960424 | 7 | 5 | NA | FALSE | NA | 0 | 1 | FALSE | FALSE |
| chr2 | 132960496 | 132960511 | 5 | 3 | NA | FALSE | MANY | 2 | 1 | FALSE | FALSE |
| chr2 | 133024555 | 133024555 | 4 | 4 | GA-rich | TRUE | NA | 0 | NA | TRUE | FALSE |
| chr2 | 133057124 | 133057132 | 4 | 4 | L1PB | TRUE | NA | 0 | 1 | FALSE | FALSE |
| chr2 | 133551882 | 133551901 | 4 | 2 | (GA)n | TRUE | NA | 0 | 1 | FALSE | FALSE |
| chr2 | 134552148 | 134552186 | 4 | 3 | L1MC3 | TRUE | NA | 0 | 1 | FALSE | FALSE |
| chr2 | 135755384 | 135755426 | 4 | 3 | MANY | TRUE | NA | 0 | 1 | FALSE | FALSE |
| chr2 | 135771748 | 135771780 | 4 | 2 | AluJb | TRUE | NA | 0 | 1 | FALSE | FALSE |
| chr2 | 135791000 | 135791045 | 4 | 3 | SVA D | TRUE | NA | 0 | NA | FALSE | FALSE |
| chr2 | 135792837 | 135792861 | 4 | 3 | AluJb | TRUE | NA | 0 | NA | FALSE | FALSE |
| chr2 | 136011289 | 136011310 | 4 | 2 | Tigger3a | TRUE | NA | 0 | 1 | FALSE | FALSE |
| chr2 | 136830167 | 136830198 | 5 | 4 | HERVK3-int | TRUE | NA | 0 | 1 | FALSE | FALSE |
| chr2 | 137313631 | 137313632 | 4 | 3 | L1MA7 | TRUE | NA | 0 | 1 | FALSE | FALSE |
| chr2 | 137434920 | 137434932 | 4 | 4 | NA | FALSE | NA | 0 | 1 | FALSE | FALSE |
| chr2 | 137438597 | 137438614 | 5 | 5 | L1PA10 | TRUE | NA | 0 | NA | FALSE | FALSE |
| chr2 | 137580271 | 137580272 | 4 | 3 | NA | FALSE | NA | 0 | 1 | FALSE | FALSE |
| chr2 | 138087401 | 138087413 | 4 | 3 | THE1C-int | TRUE | NA | 0 | 1 | FALSE | FALSE |
| chr2 | 138467389 | 138467397 | 4 | 4 | L1M1 | TRUE | NA | 0 | 1 | FALSE | FALSE |
| chr2 | 139749639 | 139749645 | 4 | 3 | MER41-int | TRUE | NA | 0 | NA | FALSE | FALSE |
| chr2 | 139906364 | 139906373 | 4 | 3 | L1MC4 | TRUE | NA | 0 | 1 | FALSE | FALSE |
| chr2 | 141227354 | 141227368 | 4 | 2 | MIRb | TRUE | NA | 0 | 1 | FALSE | FALSE |
| chr2 | 141576775 | 141576784 | 4 | 4 | NA | FALSE | NA | 0 | 1 | FALSE | FALSE |
| chr2 | 141723597 | 141723623 | 6 | 6 | L1PA5 | TRUE | NA | 0 | 1 | FALSE | FALSE |
| chr2 | 141824568 | 141824596 | 4 | 3 | NA | FALSE | NA | 0 | 1 | FALSE | FALSE |
| chr2 | 141855819 | 141855845 | 4 | 4 | NA | FALSE | NA | 0 | 1 | FALSE | FALSE |
| chr2 | 141962303 | 141962330 | 4 | 3 | AluSg4 | TRUE | NA | 0 | NA | FALSE | FALSE |
| chr2 | 143052529 | 143052551 | 4 | 2 | L1MC4a | TRUE | NA | 0 | NA | FALSE | FALSE |
| chr2 | 143111410 | 143111440 | 5 | 5 | (TC)n | TRUE | NA | 0 | NA | FALSE | FALSE |
| chr2 | 143438857 | 143438877 | 4 | 4 | MER34A | TRUE | NA | 0 | 1 | FALSE | FALSE |
| chr2 | 146975420 | 146975461 | 4 | 4 | L1MEf | TRUE | NA | 0 | 1 | FALSE | FALSE |
| chr2 | 147598152 | 147598161 | 4 | 4 | L1PB | TRUE | NA | 0 | NA | FALSE | FALSE |
| chr2 | 147863160 | 147863182 | 5 | 5 | (GA)n | TRUE | NA | 0 | NA | FALSE | FALSE |
| chr2 | 148024785 | 148024799 | 4 | 3 | L1M1 | TRUE | NA | 0 | 1 | FALSE | FALSE |
| chr2 | 148073296 | 148073314 | 4 | 4 | HERV9-int | TRUE | NA | 0 | 1 | FALSE | FALSE |
| chr2 | 148076223 | 148076248 | 4 | 3 | MANY | TRUE | NA | 0 | NA | FALSE | FALSE |
| chr2 | 148380806 | 148380838 | 4 | 2 | NA | FALSE | NA | 0 | 1 | FALSE | FALSE |
| chr2 | 149268394 | 149268464 | 4 | 4 | MANY | TRUE | NA | 0 | 1 | FALSE | FALSE |
| chr2 | 149434441 | 149434501 | 5 | 4 | L1ME4a | TRUE | NA | 0 | 1 | FALSE | FALSE |
| chr2 | 150705817 | 150705840 | 4 | 3 | NA | FALSE | NA | 0 | 1 | TRUE | FALSE |
| chr2 | 151317039 | 151317075 | 4 | 3 | NA | FALSE | NA | 0 | 1 | FALSE | FALSE |
| chr2 | 151748615 | 151748621 | 4 | 4 | (TC)n | TRUE | NA | 0 | NA | FALSE | FALSE |
| chr2 | 153861226 | 153861227 | 4 | 4 | NA | FALSE | NA | 0 | 1 | FALSE | FALSE |
| chr2 | 154099381 | 154099399 | 4 | 4 | HUERS-P1-int | TRUE | NA | 0 | 1 | FALSE | FALSE |
| chr2 | 154845914 | 154845916 | 4 | 3 | NA | FALSE | NA | 0 | 1 | FALSE | FALSE |
| chr2 | 155168295 | 155168311 | 5 | 5 | MER11C | TRUE | NA | 0 | NA | FALSE | FALSE |
| chr2 | 155317845 | 155317846 | 4 | 4 | NA | FALSE | NA | 0 | 1 | FALSE | FALSE |
| chr2 | 156189655 | 156189684 | 4 | 2 | AluSx | TRUE | NA | 0 | NA | FALSE | FALSE |
| chr2 | 157095279 | 157095279 | 4 | 4 | NA | FALSE | NA | 0 | 1 | FALSE | FALSE |
| chr2 | 157123438 | 157123497 | 4 | 3 | AluJb | TRUE | NA | 0 | 1 | FALSE | FALSE |
| chr2 | 157227045 | 157227103 | 6 | 4 | L1PA5 | TRUE | NA | 0 | NA | FALSE | FALSE |
| chr2 | 157729624 | 157729658 | 4 | 2 | L1PA7 | TRUE | NA | 0 | NA | FALSE | FALSE |
| chr2 | 158281565 | 158281605 | 4 | 4 | L1MEg | TRUE | NA | 0 | 1 | FALSE | FALSE |
| chr2 | 158291356 | 158291364 | 4 | 3 | MANY | TRUE | NA | 0 | NA | FALSE | FALSE |
| chr2 | 158526671 | 158526672 | 4 | 2 | AluY | TRUE | NA | 0 | NA | FALSE | FALSE |
| chr2 | 158994336 | 158994360 | 4 | 2 | NA | FALSE | NA | 0 | 1 | FALSE | FALSE |
| chr2 | 159560542 | 159560638 | 5 | 4 | NA | FALSE | NA | 0 | 1 | FALSE | FALSE |
| chr2 | 159614002 | 159614031 | 4 | 4 | NA | FALSE | CTCF | 1 | 1 | FALSE | FALSE |
| chr2 | 159705270 | 159705278 | 6 | 4 | HERVE-int | TRUE | NA | 0 | 1 | TRUE | FALSE |
| chr2 | 159727461 | 159727596 | 7 | 6 | SATR1 | TRUE | NA | 0 | 1 | FALSE | FALSE |
| chr2 | 159974448 | 159974480 | 4 | 2 | NA | FALSE | MANY | 3 | 1 | FALSE | FALSE |
| chr2 | 160273674 | 160273685 | 4 | 4 | AluSx1 | TRUE | NA | 0 | NA | FALSE | FALSE |
| chr2 | 162571990 | 162572018 | 4 | 4 | L1PA10 | TRUE | NA | 0 | 1 | FALSE | FALSE |
| chr2 | 163383623 | 163383635 | 4 | 3 | NA | FALSE | NA | 0 | 1 | FALSE | FALSE |
| chr2 | 164512294 | 164512315 | 7 | 7 | (CA)n | TRUE | NA | 0 | NA | TRUE | FALSE |
| chr2 | 165379403 | 165379452 | 4 | 3 | MLT1G3 | TRUE | c-Myc | 1 | 1 | TRUE | FALSE |
| chr2 | 167062263 | 167062299 | 4 | 3 | L1PA6 | TRUE | NA | 0 | NA | FALSE | FALSE |
| chr2 | 167599347 | 167599359 | 4 | 3 | HERV9-int | TRUE | NA | 0 | 1 | FALSE | FALSE |
| chr2 | 167784221 | 167784231 | 4 | 3 | MLT1D | TRUE | NA | 0 | 1 | FALSE | FALSE |
| chr2 | 167903431 | 167903458 | 4 | 3 | MANY | TRUE | NA | 0 | 1 | FALSE | FALSE |
| chr2 | 169307709 | 169307765 | 4 | 3 | NA | FALSE | NA | 0 | 1 | FALSE | FALSE |
| chr2 | 169723268 | 169723302 | 4 | 3 | L1P1 | TRUE | NA | 0 | NA | FALSE | FALSE |
| chr2 | 169925699 | 169925739 | 4 | 3 | NA | FALSE | NA | 0 | 1 | FALSE | FALSE |
| chr2 | 170183615 | 170183721 | 7 | 4 | SVA F | TRUE | NA | 0 | NA | FALSE | FALSE |
| chr2 | 170698230 | 170698235 | 4 | 3 | LTR12C | TRUE | NA | 0 | NA | FALSE | FALSE |
| chr2 | 171718464 | 171718483 | 4 | 3 | (TG)n | TRUE | NA | 0 | NA | FALSE | FALSE |
| chr2 | 171822315 | 171822365 | 4 | 2 | NA | FALSE | NA | 0 | 1 | TRUE | FALSE |
| chr2 | 172107470 | 172107532 | 4 | 4 | AluY | TRUE | NA | 0 | NA | FALSE | FALSE |
| chr2 | 172328990 | 172329059 | 4 | 2 | L2c | TRUE | NA | 0 | 1 | FALSE | FALSE |
| chr2 | 172396439 | 172396450 | 4 | 2 | AluSz | TRUE | NA | 0 | NA | FALSE | FALSE |
| chr2 | 173253013 | 173253033 | 4 | 3 | NA | FALSE | NA | 0 | 1 | TRUE | FALSE |
| chr2 | 175182957 | 175182976 | 4 | 3 | NA | FALSE | NA | 0 | 1 | FALSE | FALSE |
| chr2 | 175575378 | 175575420 | 4 | 3 | SATR1 | TRUE | NA | 0 | 1 | TRUE | FALSE |
| chr2 | 175673139 | 175673148 | 4 | 3 | AluSq2 | TRUE | NA | 0 | NA | FALSE | FALSE |
| chr2 | 176177839 | 176177884 | 4 | 4 | NA | FALSE | NA | 0 | 1 | FALSE | FALSE |
| chr2 | 176369186 | 176369197 | 4 | 3 | MIR | TRUE | NA | 0 | 1 | FALSE | FALSE |
| chr2 | 176447601 | 176447625 | 4 | 3 | NA | FALSE | NA | 0 | 1 | FALSE | FALSE |
| chr2 | 177883348 | 177883420 | 5 | 4 | NA | FALSE | NA | 0 | 1 | FALSE | FALSE |
| chr2 | 177886138 | 177886149 | 4 | 3 | NA | FALSE | NA | 0 | 1 | FALSE | FALSE |
| chr2 | 178443760 | 178443760 | 5 | 5 | NA | FALSE | NA | 0 | 1 | FALSE | FALSE |
| chr2 | 178472593 | 178472658 | 4 | 3 | MER31A | TRUE | NA | 0 | 1 | FALSE | FALSE |
| chr2 | 179579148 | 179579180 | 4 | 4 | NA | FALSE | NA | 0 | 1 | FALSE | FALSE |
| chr2 | 179746639 | 179746641 | 4 | 4 | (TC)n | TRUE | NA | 0 | NA | FALSE | FALSE |
| chr2 | 179873650 | 179873673 | 4 | 3 | L2a | TRUE | NA | 0 | 1 | FALSE | FALSE |
| chr2 | 179911724 | 179911735 | 4 | 3 | NA | FALSE | NA | 0 | 1 | FALSE | FALSE |
| chr2 | 179929287 | 179929304 | 4 | 2 | L1PA3 | TRUE | NA | 0 | 1 | FALSE | FALSE |
| chr2 | 181127490 | 181127518 | 4 | 4 | NA | FALSE | NA | 0 | 1 | FALSE | FALSE |
| chr2 | 182107414 | 182107417 | 4 | 3 | (CA)n | TRUE | NA | 0 | NA | TRUE | FALSE |
| chr2 | 182276375 | 182276409 | 5 | 4 | L1PA8A | TRUE | CTCF | 1 | 1 | FALSE | FALSE |
| chr2 | 183104192 | 183104220 | 4 | 2 | NA | FALSE | NA | 0 | 1 | FALSE | FALSE |
| chr2 | 183142108 | 183142139 | 4 | 4 | MER11A | TRUE | NA | 0 | 1 | FALSE | FALSE |
| chr2 | 184122434 | 184122468 | 4 | 3 | AluY | TRUE | NA | 0 | NA | FALSE | FALSE |
| chr2 | 184556199 | 184556223 | 4 | 2 | L1MA2 | TRUE | NA | 0 | 1 | FALSE | FALSE |
| chr2 | 184664816 | 184664840 | 4 | 2 | L1M4c | TRUE | NA | 0 | 1 | FALSE | FALSE |
| chr2 | 184993821 | 184993843 | 4 | 3 | (GA)n | TRUE | NA | 0 | NA | FALSE | FALSE |
| chr2 | 185019502 | 185019506 | 4 | 3 | MamSINE1 | TRUE | MANY | 2 | 1 | FALSE | FALSE |
| chr2 | 185100815 | 185100831 | 4 | 4 | L1MD2 | TRUE | NA | 0 | 1 | FALSE | FALSE |
| chr2 | 185407170 | 185407174 | 4 | 3 | L1PA3 | TRUE | NA | 0 | 1 | FALSE | FALSE |
| chr2 | 185781765 | 185781782 | 4 | 3 | MLT2D | TRUE | NA | 0 | 1 | FALSE | FALSE |
| chr2 | 186052072 | 186052072 | 4 | 4 | (TC)n | TRUE | NA | 0 | NA | FALSE | FALSE |
| chr2 | 187018659 | 187018713 | 4 | 3 | SVA F | TRUE | NA | 0 | NA | FALSE | FALSE |
| chr2 | 188129818 | 188129822 | 4 | 3 | NA | FALSE | NA | 0 | 1 | FALSE | FALSE |
| chr2 | 188528061 | 188528066 | 4 | 4 | NA | FALSE | NA | 0 | 1 | FALSE | FALSE |
| chr2 | 189110541 | 189110589 | 4 | 3 | NA | FALSE | NA | 0 | 1 | FALSE | FALSE |
| chr2 | 189721956 | 189721985 | 4 | 2 | THE1B | TRUE | NA | 0 | 1 | FALSE | FALSE |
| chr2 | 191471219 | 191471247 | 4 | 2 | AluSx | TRUE | NA | 0 | NA | TRUE | FALSE |
| chr2 | 191642088 | 191642101 | 4 | 3 | L1PA4 | TRUE | NA | 0 | NA | FALSE | FALSE |
| chr2 | 192606945 | 192606956 | 4 | 4 | SVA F | TRUE | NA | 0 | NA | FALSE | FALSE |
| chr2 | 192705577 | 192705605 | 4 | 3 | NA | FALSE | NA | 0 | 1 | TRUE | FALSE |
| chr2 | 193175648 | 193175655 | 4 | 4 | NA | FALSE | NA | 0 | 1 | FALSE | FALSE |
| chr2 | 193679429 | 193679442 | 4 | 4 | L1MCc | TRUE | NA | 0 | 1 | TRUE | FALSE |
| chr2 | 193763728 | 193763738 | 4 | 3 | MER21B | TRUE | NA | 0 | 1 | FALSE | FALSE |
| chr2 | 193807939 | 193807948 | 4 | 4 | L1M4b | TRUE | NA | 0 | 1 | FALSE | FALSE |
| chr2 | 193845904 | 193845904 | 4 | 4 | AluSq2 | TRUE | NA | 0 | NA | FALSE | FALSE |
| chr2 | 193910118 | 193910120 | 4 | 2 | NA | FALSE | NA | 0 | 1 | FALSE | FALSE |
| chr2 | 194314188 | 194314188 | 4 | 4 | NA | FALSE | NA | 0 | 1 | FALSE | FALSE |
| chr2 | 195433022 | 195433022 | 4 | 4 | NA | FALSE | NA | 0 | 1 | TRUE | FALSE |
| chr2 | 195471556 | 195471572 | 4 | 4 | HUERS-P3b-int | TRUE | NA | 0 | 1 | FALSE | FALSE |
| chr2 | 195499302 | 195499303 | 4 | 3 | NA | FALSE | NA | 0 | 1 | FALSE | FALSE |
| chr2 | 195864355 | 195864361 | 4 | 3 | L2 | TRUE | NA | 0 | 1 | FALSE | FALSE |
| chr2 | 195874551 | 195874563 | 5 | 4 | NA | FALSE | NA | 0 | 1 | FALSE | FALSE |
| chr2 | 195978335 | 195978345 | 4 | 4 | THE1D | TRUE | NA | 0 | 1 | FALSE | FALSE |
| chr2 | 196174809 | 196174826 | 5 | 4 | (TC)n | TRUE | NA | 0 | NA | FALSE | FALSE |
| chr2 | 196717636 | 196717640 | 5 | 5 | (CA)n | TRUE | NA | 0 | NA | TRUE | FALSE |
| chr2 | 198363500 | 198363500 | 5 | 5 | NA | FALSE | MANY | 2 | NA | FALSE | FALSE |
| chr2 | 198564163 | 198564224 | 5 | 5 | L1PA5 | TRUE | NA | 0 | NA | TRUE | FALSE |
| chr2 | 199142382 | 199142390 | 4 | 3 | (TC)n | TRUE | NA | 0 | NA | FALSE | FALSE |
| chr2 | 199363516 | 199363532 | 4 | 4 | NA | FALSE | NA | 0 | 1 | FALSE | FALSE |
| chr2 | 199385502 | 199385532 | 4 | 3 | AluJb | TRUE | NA | 0 | 1 | FALSE | FALSE |
| chr2 | 199918872 | 199918883 | 4 | 4 | NA | FALSE | NA | 0 | 1 | FALSE | FALSE |
| chr2 | 200112830 | 200112847 | 4 | 3 | HERVH-int | TRUE | NA | 0 | 1 | FALSE | FALSE |
| chr2 | 200902111 | 200902133 | 4 | 4 | (TG)n | TRUE | NA | 0 | NA | FALSE | FALSE |
| chr2 | 201083150 | 201083179 | 4 | 4 | AluYf4 | TRUE | NA | 0 | NA | FALSE | FALSE |
| chr2 | 201528292 | 201528305 | 4 | 4 | MANY | TRUE | NA | 0 | NA | FALSE | FALSE |
| chr2 | 202447528 | 202447600 | 5 | 3 | NA | FALSE | NA | 0 | 1 | FALSE | FALSE |
| chr2 | 202982020 | 202982095 | 4 | 4 | NA | FALSE | NA | 0 | 1 | FALSE | FALSE |
| chr2 | 203367016 | 203367082 | 4 | 2 | MANY | TRUE | NA | 0 | 1 | FALSE | FALSE |
| chr2 | 203369165 | 203369183 | 8 | 7 | LTR12C | TRUE | NA | 0 | NA | TRUE | FALSE |
| chr2 | 203598393 | 203598425 | 4 | 3 | MER11A | TRUE | NA | 0 | 1 | FALSE | FALSE |
| chr2 | 204375306 | 204375331 | 4 | 2 | MER102c | TRUE | NA | 0 | 1 | TRUE | FALSE |
| chr2 | 204911700 | 204911745 | 7 | 6 | MANY | TRUE | NA | 0 | NA | FALSE | FALSE |
| chr2 | 207859924 | 207859979 | 4 | 3 | MSTB1 | TRUE | NA | 0 | 1 | TRUE | FALSE |
| chr2 | 208576078 | 208576131 | 4 | 4 | NA | FALSE | MANY | 27 | 1 | TRUE | TRUE |
| chr2 | 209082503 | 209082507 | 4 | 2 | HERVH-int | TRUE | NA | 0 | NA | FALSE | FALSE |
| chr2 | 209302274 | 209302319 | 6 | 3 | NA | FALSE | NA | 0 | 1 | FALSE | FALSE |
| chr2 | 209377391 | 209377408 | 4 | 3 | NA | FALSE | NA | 0 | 1 | FALSE | FALSE |
| chr2 | 209423507 | 209423520 | 4 | 3 | NA | FALSE | NA | 0 | 1 | FALSE | FALSE |
| chr2 | 209869331 | 209869343 | 4 | 3 | MER61-int | TRUE | NA | 0 | 1 | FALSE | FALSE |
| chr2 | 209906375 | 209906401 | 4 | 2 | MER82 | TRUE | NA | 0 | 1 | FALSE | FALSE |
| chr2 | 210663658 | 210663716 | 5 | 4 | MANY | TRUE | NA | 0 | 1 | FALSE | FALSE |
| chr2 | 210867228 | 210867280 | 4 | 3 | NA | FALSE | MANY | 23 | 1 | TRUE | TRUE |
| chr2 | 210934576 | 210934612 | 8 | 6 | AluSx1 | TRUE | NA | 0 | 1 | FALSE | FALSE |
| chr2 | 211035502 | 211035555 | 5 | 4 | NA | FALSE | MANY | 16 | 1 | TRUE | TRUE |
| chr2 | 211161515 | 211161561 | 4 | 4 | L1MA5 | TRUE | NA | 0 | 1 | FALSE | FALSE |
| chr2 | 211884416 | 211884433 | 4 | 4 | L1PB1 | TRUE | NA | 0 | NA | FALSE | FALSE |
| chr2 | 212384911 | 212384924 | 4 | 3 | NA | FALSE | NA | 0 | 1 | TRUE | FALSE |
| chr2 | 212696256 | 212696275 | 4 | 3 | NA | FALSE | NA | 0 | 1 | TRUE | FALSE |
| chr2 | 212923107 | 212923132 | 4 | 4 | L1PA5 | TRUE | NA | 0 | 1 | FALSE | FALSE |
| chr2 | 213065499 | 213065522 | 6 | 6 | NA | FALSE | NA | 0 | 1 | FALSE | FALSE |
| chr2 | 213534231 | 213534233 | 4 | 2 | MIRb | TRUE | MafK (ab50322) | 1 | 1 | FALSE | FALSE |
| chr2 | 213629893 | 213629894 | 4 | 3 | NA | FALSE | NA | 0 | 1 | FALSE | FALSE |
| chr2 | 213641108 | 213641124 | 5 | 4 | NA | FALSE | NA | 0 | 1 | TRUE | FALSE |
| chr2 | 213656211 | 213656231 | 4 | 4 | NA | FALSE | NA | 0 | 1 | FALSE | FALSE |
| chr2 | 213672944 | 213672945 | 5 | 4 | L2c | TRUE | NA | 0 | 1 | FALSE | FALSE |
| chr2 | 214412474 | 214412498 | 4 | 4 | NA | FALSE | NA | 0 | 1 | FALSE | FALSE |
| chr2 | 214501549 | 214501558 | 4 | 3 | L1PBa | TRUE | NA | 0 | 1 | FALSE | FALSE |
| chr2 | 214508839 | 214508855 | 4 | 4 | L1M1 | TRUE | NA | 0 | 1 | FALSE | FALSE |
| chr2 | 215279839 | 215279849 | 4 | 2 | NA | FALSE | NA | 0 | 1 | FALSE | FALSE |
| chr2 | 215335767 | 215335806 | 4 | 3 | NA | FALSE | NA | 0 | 1 | TRUE | FALSE |
| chr2 | 215572321 | 215572329 | 6 | 3 | (A)n | TRUE | NA | 0 | NA | FALSE | FALSE |
| chr2 | 216053788 | 216053792 | 5 | 4 | (TC)n | TRUE | NA | 0 | NA | TRUE | FALSE |
| chr2 | 216241778 | 216241789 | 8 | 4 | (TG)n | TRUE | NA | 0 | NA | FALSE | FALSE |
| chr2 | 216529075 | 216529144 | 4 | 3 | (TG)n | TRUE | NA | 0 | 1 | TRUE | FALSE |
| chr2 | 217166971 | 217167044 | 4 | 4 | AluY | TRUE | NA | 0 | NA | FALSE | FALSE |
| chr2 | 218674045 | 218674086 | 4 | 4 | (TGGA)n | TRUE | NA | 0 | NA | FALSE | FALSE |
| chr2 | 218811691 | 218811693 | 5 | 5 | (TC)n | TRUE | NA | 0 | NA | FALSE | FALSE |
| chr2 | 218825957 | 218825984 | 7 | 6 | (CA)n | TRUE | NA | 0 | NA | FALSE | FALSE |
| chr2 | 219134963 | 219135029 | 5 | 5 | NA | FALSE | MANY | 36 | 1 | TRUE | TRUE |
| chr2 | 219711610 | 219711669 | 10 | 9 | SVA F | TRUE | NA | 0 | NA | FALSE | FALSE |
| chr2 | 220723097 | 220723118 | 4 | 3 | NA | FALSE | NA | 0 | 1 | FALSE | FALSE |
| chr2 | 220746434 | 220746451 | 4 | 4 | MER31A | TRUE | NA | 0 | 1 | FALSE | FALSE |
| chr2 | 220812834 | 220812845 | 4 | 3 | NA | FALSE | NA | 0 | 1 | FALSE | FALSE |
| chr2 | 221338837 | 221338841 | 4 | 4 | (TC)n | TRUE | NA | 0 | NA | FALSE | FALSE |
| chr2 | 223939222 | 223939272 | 4 | 3 | NA | FALSE | NA | 0 | 1 | FALSE | FALSE |
| chr2 | 225090206 | 225090272 | 4 | 4 | LTR7 | TRUE | NA | 0 | NA | TRUE | FALSE |
| chr2 | 225139133 | 225139164 | 4 | 4 | L2c | TRUE | NA | 0 | 1 | FALSE | FALSE |
| chr2 | 225209787 | 225209842 | 4 | 3 | MSTA-int | TRUE | NA | 0 | 1 | FALSE | FALSE |
| chr2 | 225730571 | 225730591 | 6 | 6 | CT-rich | TRUE | NA | 0 | NA | FALSE | FALSE |
| chr2 | 226786746 | 226786746 | 7 | 7 | (TG)n | TRUE | NA | 0 | NA | FALSE | FALSE |
| chr2 | 228036664 | 228036677 | 4 | 4 | MER50 | TRUE | Nrf1 | 1 | NA | FALSE | FALSE |
| chr2 | 228436925 | 228436939 | 4 | 3 | MANY | TRUE | NA | 0 | 1 | FALSE | FALSE |
| chr2 | 228696787 | 228696812 | 4 | 3 | LTR67B | TRUE | NA | 0 | 1 | FALSE | FALSE |
| chr2 | 229557182 | 229557182 | 4 | 4 | NA | FALSE | NA | 0 | 1 | FALSE | FALSE |
| chr2 | 229573160 | 229573179 | 4 | 3 | L1ME2z | TRUE | NA | 0 | 1 | FALSE | FALSE |
| chr2 | 229628979 | 229628989 | 4 | 3 | AluSz6 | TRUE | NA | 0 | NA | FALSE | FALSE |
| chr2 | 230596458 | 230596480 | 4 | 2 | NA | FALSE | NA | 0 | 1 | FALSE | FALSE |
| chr2 | 230909437 | 230909465 | 4 | 3 | (TG)n | TRUE | NA | 0 | NA | FALSE | FALSE |
| chr2 | 232372799 | 232372809 | 4 | 4 | AluSg | TRUE | NA | 0 | NA | FALSE | FALSE |
| chr2 | 232694819 | 232694881 | 4 | 2 | MER20 | TRUE | NA | 0 | NA | FALSE | FALSE |
| chr2 | 232697952 | 232697983 | 5 | 2 | MER20 | TRUE | NA | 0 | NA | FALSE | FALSE |
| chr2 | 232699022 | 232699094 | 4 | 4 | MER20 | TRUE | NA | 0 | NA | FALSE | FALSE |
| chr2 | 232700914 | 232701081 | 11 | 9 | MER20 | TRUE | NA | 0 | 1 | FALSE | FALSE |
| chr2 | 233754942 | 233754984 | 8 | 5 | NA | FALSE | NA | 0 | 1 | TRUE | FALSE |
| chr2 | 233764939 | 233764939 | 4 | 4 | (CA)n | TRUE | BCL3 | 1 | NA | FALSE | FALSE |
| chr2 | 234095391 | 234095419 | 4 | 3 | AluSz | TRUE | NA | 0 | NA | FALSE | FALSE |
| chr2 | 234134490 | 234134500 | 4 | 4 | (GA)n | TRUE | MafK (ab50322) | 1 | 1 | TRUE | FALSE |
| chr2 | 234400261 | 234400277 | 5 | 4 | (TG)n | TRUE | NA | 0 | NA | FALSE | FALSE |
| chr2 | 234763214 | 234763234 | 6 | 6 | NA | FALSE | MANY | 24 | 1 | TRUE | TRUE |
| chr2 | 237010837 | 237010900 | 6 | 4 | AluY | TRUE | NA | 0 | NA | FALSE | FALSE |
| chr2 | 237668978 | 237669018 | 4 | 3 | GA-rich | TRUE | NA | 0 | 1 | FALSE | FALSE |
| chr2 | 238252135 | 238252156 | 14 | 10 | AluSg | TRUE | NA | 0 | NA | FALSE | FALSE |
| chr2 | 238276768 | 238276775 | 4 | 2 | NA | FALSE | NA | 0 | 1 | TRUE | FALSE |
| chr2 | 238431120 | 238431147 | 4 | 4 | NA | FALSE | NA | 0 | NA | FALSE | FALSE |
| chr2 | 239686078 | 239686146 | 4 | 4 | NA | FALSE | MANY | 3 | 1 | TRUE | TRUE |
| chr2 | 239755281 | 239755301 | 4 | 2 | NA | FALSE | CTCF | 1 | 1 | TRUE | FALSE |
| chr2 | 240072360 | 240072421 | 4 | 4 | NA | FALSE | NA | 0 | 1 | TRUE | FALSE |
| chr2 | 240352381 | 240352435 | 4 | 2 | NA | FALSE | NA | 0 | NA | FALSE | FALSE |
| chr2 | 240398419 | 240398481 | 4 | 4 | NA | FALSE | NA | 0 | 1 | TRUE | FALSE |
| chr2 | 240427795 | 240427853 | 4 | 2 | NA | FALSE | BCL3 | 1 | 1 | TRUE | FALSE |
| chr2 | 241293887 | 241293931 | 4 | 4 | NA | FALSE | NA | 0 | NA | TRUE | FALSE |
| chr2 | 241324981 | 241325014 | 5 | 4 | (GGCTG)n | TRUE | NA | 0 | 1 | FALSE | FALSE |
| chr2 | 241395126 | 241395166 | 4 | 2 | NA | FALSE | NA | 0 | 1 | TRUE | FALSE |
| chr2 | 241500537 | 241500555 | 4 | 4 | NA | FALSE | MANY | 17 | 1 | TRUE | TRUE |
| chr2 | 241579473 | 241579494 | 4 | 3 | (TTCC)n | TRUE | NA | 0 | NA | TRUE | FALSE |
| chr2 | 241800433 | 241800513 | 6 | 6 | MER84-int | TRUE | NA | 0 | 1 | TRUE | FALSE |
| chr2 | 241873598 | 241873722 | 5 | 3 | NA | FALSE | NA | 0 | 1 | TRUE | FALSE |
| chr2 | 241925093 | 241925105 | 5 | 5 | (CA)n | TRUE | NA | 0 | NA | FALSE | FALSE |
| chr2 | 242416603 | 242416646 | 4 | 2 | AluSc | TRUE | NA | 0 | NA | FALSE | FALSE |
| chr2 | 242680975 | 242681111 | 9 | 9 | NA | FALSE | NA | 0 | NA | FALSE | FALSE |
| chr2 | 242771070 | 242771138 | 4 | 3 | MANY | TRUE | NA | 0 | 1 | FALSE | FALSE |
| chr2 | 242927469 | 242927595 | 5 | 3 | NA | FALSE | USF-1 | 1 | 1 | TRUE | FALSE |
| chr2 | 242937902 | 242937961 | 4 | 3 | AluSz | TRUE | NA | 0 | 1 | TRUE | FALSE |
| chr2 | 242946059 | 242946064 | 4 | 3 | NA | FALSE | NA | 0 | NA | FALSE | FALSE |
| chr2 | 242988858 | 242988934 | 5 | 3 | NA | FALSE | SUZ12 | 1 | NA | TRUE | FALSE |
| chr2 | 243062604 | 243062639 | 7 | 6 | AluSq2 | TRUE | NA | 0 | NA | FALSE | FALSE |
| chr3 | 177208 | 177294 | 7 | 5 | NA | FALSE | NA | 0 | NA | FALSE | FALSE |
| chr3 | 573503 | 573510 | 4 | 3 | L1MB3 | TRUE | NA | 0 | 1 | FALSE | FALSE |
| chr3 | 634849 | 634853 | 10 | 9 | NA | FALSE | NA | 0 | 1 | FALSE | FALSE |
| chr3 | 635429 | 635430 | 4 | 2 | MER58A | TRUE | NA | 0 | 1 | FALSE | FALSE |
| chr3 | 651036 | 651060 | 5 | 4 | NA | FALSE | NA | 0 | 1 | FALSE | FALSE |
| chr3 | 656753 | 656753 | 4 | 4 | NA | FALSE | NA | 0 | 1 | FALSE | FALSE |
| chr3 | 663099 | 663099 | 4 | 4 | (TATATG)n | TRUE | NA | 0 | NA | FALSE | FALSE |
| chr3 | 767987 | 768007 | 4 | 3 | NA | FALSE | NA | 0 | 1 | FALSE | FALSE |
| chr3 | 1000035 | 1000047 | 4 | 3 | NA | FALSE | NA | 0 | 1 | FALSE | FALSE |
| chr3 | 1481653 | 1481688 | 4 | 4 | NA | FALSE | NA | 0 | 1 | FALSE | FALSE |
| chr3 | 2196102 | 2196103 | 4 | 3 | NA | FALSE | NA | 0 | 1 | FALSE | FALSE |
| chr3 | 2581158 | 2581175 | 5 | 4 | NA | FALSE | NA | 0 | 1 | FALSE | FALSE |
| chr3 | 2789049 | 2789049 | 4 | 4 | AluY | TRUE | NA | 0 | NA | FALSE | FALSE |
| chr3 | 3067210 | 3067230 | 4 | 4 | L1ME4a | TRUE | NA | 0 | 1 | FALSE | FALSE |
| chr3 | 3667569 | 3667588 | 4 | 4 | MIRb | TRUE | NA | 0 | 1 | TRUE | FALSE |
| chr3 | 3744562 | 3744586 | 4 | 4 | MIRb | TRUE | NA | 0 | 1 | FALSE | FALSE |
| chr3 | 5136318 | 5136325 | 4 | 3 | AluSg | TRUE | NA | 0 | 1 | TRUE | FALSE |
| chr3 | 5611400 | 5611417 | 4 | 4 | L1MEd | TRUE | NA | 0 | 1 | FALSE | FALSE |
| chr3 | 6314485 | 6314491 | 4 | 2 | L1PA3 | TRUE | NA | 0 | NA | FALSE | FALSE |
| chr3 | 8184387 | 8184411 | 4 | 2 | NA | FALSE | NA | 0 | 1 | FALSE | FALSE |
| chr3 | 8384054 | 8384075 | 4 | 3 | NA | FALSE | NA | 0 | 1 | FALSE | FALSE |
| chr3 | 8767580 | 8767638 | 4 | 3 | NA | FALSE | NA | 0 | 1 | TRUE | FALSE |
| chr3 | 9772919 | 9772953 | 5 | 3 | C-rich | TRUE | MANY | 2 | 1 | TRUE | FALSE |
| chr3 | 10115370 | 10115428 | 7 | 5 | NA | FALSE | NA | 0 | 1 | FALSE | FALSE |
| chr3 | 10583787 | 10583795 | 4 | 3 | L1MD2 | TRUE | NA | 0 | 1 | FALSE | FALSE |
| chr3 | 11266037 | 11266078 | 4 | 3 | NA | FALSE | NA | 0 | 1 | TRUE | FALSE |
| chr3 | 11309689 | 11309695 | 5 | 4 | MANY | TRUE | NA | 0 | NA | FALSE | FALSE |
| chr3 | 11632157 | 11632161 | 4 | 4 | (TG)n | TRUE | NA | 0 | NA | TRUE | FALSE |
| chr3 | 12799995 | 12800060 | 4 | 3 | NA | FALSE | NA | 0 | 1 | TRUE | FALSE |
| chr3 | 12883296 | 12883307 | 4 | 4 | NA | FALSE | MANY | 30 | 1 | TRUE | TRUE |
| chr3 | 13493377 | 13493415 | 4 | 4 | MER66A | TRUE | MANY | 29 | 1 | TRUE | TRUE |
| chr3 | 13618846 | 13618854 | 4 | 2 | L2a | TRUE | NA | 0 | 1 | TRUE | FALSE |
| chr3 | 13858226 | 13858273 | 4 | 3 | (TCCA)n | TRUE | NA | 0 | 1 | TRUE | FALSE |
| chr3 | 13898410 | 13898465 | 5 | 5 | (GAA)n | TRUE | NA | 0 | NA | FALSE | FALSE |
| chr3 | 14060316 | 14060339 | 4 | 3 | NA | FALSE | NA | 0 | 1 | TRUE | FALSE |
| chr3 | 14105954 | 14106090 | 11 | 7 | (CAG)n | TRUE | NA | 0 | 1 | TRUE | FALSE |
| chr3 | 14693049 | 14693089 | 6 | 4 | NA | FALSE | MANY | 51 | 1 | TRUE | TRUE |
| chr3 | 15187628 | 15187628 | 5 | 5 | LTR5A | TRUE | NA | 0 | NA | FALSE | FALSE |
| chr3 | 16306453 | 16306507 | 9 | 8 | NA | FALSE | MANY | 37 | 1 | TRUE | TRUE |
| chr3 | 16822878 | 16822893 | 6 | 2 | L1PA5 | TRUE | NA | 0 | NA | FALSE | FALSE |
| chr3 | 16857027 | 16857049 | 4 | 4 | NA | FALSE | NA | 0 | 1 | FALSE | FALSE |
| chr3 | 16965272 | 16965316 | 5 | 3 | L1M4 | TRUE | NA | 0 | 1 | FALSE | FALSE |
| chr3 | 17184400 | 17184436 | 4 | 2 | NA | FALSE | NA | 0 | 1 | TRUE | FALSE |
| chr3 | 18865586 | 18865598 | 4 | 3 | MLT1B-int | TRUE | NA | 0 | 1 | FALSE | FALSE |
| chr3 | 18947641 | 18947641 | 6 | 6 | NA | FALSE | NA | 0 | 1 | TRUE | FALSE |
| chr3 | 19680548 | 19680552 | 4 | 4 | MLT1D | TRUE | NA | 0 | NA | TRUE | FALSE |
| chr3 | 19706280 | 19706311 | 4 | 4 | THE1D | TRUE | NA | 0 | 1 | FALSE | FALSE |
| chr3 | 20021946 | 20021947 | 4 | 3 | NA | FALSE | NA | 0 | 1 | FALSE | FALSE |
| chr3 | 20408773 | 20408792 | 4 | 3 | Harlequin-int | TRUE | NA | 0 | 1 | FALSE | FALSE |
| chr3 | 20453758 | 20453786 | 4 | 2 | NA | FALSE | NA | 0 | 1 | FALSE | FALSE |
| chr3 | 20455329 | 20455348 | 4 | 4 | L4 | TRUE | NA | 0 | 1 | FALSE | FALSE |
| chr3 | 20508836 | 20508836 | 4 | 4 | L1MC1 | TRUE | NA | 0 | 1 | FALSE | FALSE |
| chr3 | 20510263 | 20510271 | 4 | 4 | L1MC1 | TRUE | NA | 0 | 1 | FALSE | FALSE |
| chr3 | 20813738 | 20813745 | 4 | 3 | MER46C | TRUE | NA | 0 | 1 | FALSE | FALSE |
| chr3 | 20852190 | 20852190 | 4 | 4 | NA | FALSE | NA | 0 | 1 | FALSE | FALSE |
| chr3 | 21589663 | 21589685 | 5 | 5 | MER61A | TRUE | NA | 0 | 1 | FALSE | FALSE |
| chr3 | 22071042 | 22071060 | 4 | 3 | HERVL-int | TRUE | NA | 0 | 1 | FALSE | FALSE |
| chr3 | 22230004 | 22230025 | 4 | 3 | AluY | TRUE | NA | 0 | NA | FALSE | FALSE |
| chr3 | 22268969 | 22268980 | 4 | 4 | MER21A | TRUE | NA | 0 | 1 | FALSE | FALSE |
| chr3 | 22439962 | 22439963 | 4 | 4 | L1MB8 | TRUE | NA | 0 | 1 | FALSE | FALSE |
| chr3 | 22494169 | 22494192 | 4 | 4 | HAL1 | TRUE | NA | 0 | 1 | TRUE | FALSE |
| chr3 | 23134350 | 23134394 | 4 | 3 | L1PA4 | TRUE | NA | 0 | NA | FALSE | FALSE |
| chr3 | 23719140 | 23719185 | 4 | 1 | NA | FALSE | NA | 0 | 1 | FALSE | FALSE |
| chr3 | 24250972 | 24250998 | 6 | 2 | L1PA5 | TRUE | NA | 0 | NA | FALSE | FALSE |
| chr3 | 25802306 | 25802352 | 15 | 12 | AluSp | TRUE | NA | 0 | NA | FALSE | FALSE |
| chr3 | 26622110 | 26622119 | 4 | 3 | LTR16E1 | TRUE | NA | 0 | 1 | FALSE | FALSE |
| chr3 | 26645130 | 26645140 | 4 | 3 | THE1B-int | TRUE | NA | 0 | NA | FALSE | FALSE |
| chr3 | 28409529 | 28409553 | 4 | 4 | L1P1 | TRUE | NA | 0 | 1 | FALSE | FALSE |
| chr3 | 29214136 | 29214138 | 6 | 3 | AluY | TRUE | NA | 0 | NA | FALSE | FALSE |
| chr3 | 29788863 | 29788868 | 7 | 4 | MANY | TRUE | NA | 0 | 1 | FALSE | FALSE |
| chr3 | 30603553 | 30603561 | 5 | 5 | (TG)n | TRUE | NA | 0 | NA | TRUE | FALSE |
| chr3 | 30770695 | 30770714 | 4 | 3 | NA | FALSE | NA | 0 | 1 | FALSE | FALSE |
| chr3 | 31519099 | 31519154 | 4 | 2 | NA | FALSE | NA | 0 | 1 | FALSE | FALSE |
| chr3 | 31940002 | 31940016 | 6 | 3 | FLAM C | TRUE | NA | 0 | NA | FALSE | FALSE |
| chr3 | 33036252 | 33036305 | 9 | 5 | AluSx | TRUE | NA | 0 | NA | FALSE | FALSE |
| chr3 | 33067124 | 33067184 | 4 | 1 | MER52D | TRUE | NA | 0 | NA | FALSE | FALSE |
| chr3 | 33877348 | 33877361 | 4 | 2 | L1MC4a | TRUE | NA | 0 | 1 | FALSE | FALSE |
| chr3 | 34026510 | 34026524 | 4 | 4 | L1PA8A | TRUE | NA | 0 | NA | FALSE | FALSE |
| chr3 | 34108774 | 34108798 | 5 | 3 | NA | FALSE | NA | 0 | 1 | FALSE | FALSE |
| chr3 | 34238386 | 34238405 | 4 | 4 | AluSx1 | TRUE | NA | 0 | NA | FALSE | FALSE |
| chr3 | 34384171 | 34384185 | 4 | 4 | NA | FALSE | NA | 0 | 1 | FALSE | FALSE |
| chr3 | 34465581 | 34465591 | 4 | 4 | L2 | TRUE | NA | 0 | 1 | FALSE | FALSE |
| chr3 | 34939751 | 34939761 | 5 | 3 | NA | FALSE | NA | 0 | 1 | FALSE | FALSE |
| chr3 | 35039443 | 35039465 | 4 | 2 | MLT1I | TRUE | USF-1 | 1 | 1 | FALSE | FALSE |
| chr3 | 35504309 | 35504313 | 4 | 2 | NA | FALSE | NA | 0 | 1 | FALSE | FALSE |
| chr3 | 35638607 | 35638607 | 4 | 4 | MLT1H | TRUE | NA | 0 | 1 | FALSE | FALSE |
| chr3 | 35770830 | 35770851 | 4 | 3 | NA | FALSE | NA | 0 | 1 | FALSE | FALSE |
| chr3 | 35860111 | 35860121 | 4 | 3 | L1MC3 | TRUE | NA | 0 | NA | FALSE | FALSE |
| chr3 | 36082465 | 36082475 | 4 | 4 | L1MA2 | TRUE | NA | 0 | 1 | FALSE | FALSE |
| chr3 | 36128850 | 36128859 | 5 | 3 | L1PA4 | TRUE | NA | 0 | NA | FALSE | FALSE |
| chr3 | 36135982 | 36135999 | 4 | 3 | NA | FALSE | NA | 0 | 1 | FALSE | FALSE |
| chr3 | 36363248 | 36363258 | 4 | 3 | L1PA4 | TRUE | NA | 0 | NA | FALSE | FALSE |
| chr3 | 36694034 | 36694052 | 4 | 4 | NA | FALSE | NA | 0 | 1 | FALSE | FALSE |
| chr3 | 37380540 | 37380569 | 4 | 4 | AluY | TRUE | NA | 0 | NA | FALSE | FALSE |
| chr3 | 38348009 | 38348030 | 4 | 4 | NA | FALSE | NA | 0 | 1 | TRUE | FALSE |
| chr3 | 38780431 | 38780464 | 4 | 4 | NA | FALSE | MANY | 3 | 1 | TRUE | TRUE |
| chr3 | 39621789 | 39621789 | 4 | 4 | HERVH-int | TRUE | NA | 0 | NA | FALSE | FALSE |
| chr3 | 40125943 | 40125975 | 4 | 4 | (TA)n | TRUE | NA | 0 | NA | FALSE | FALSE |
| chr3 | 40471314 | 40471316 | 4 | 4 | (TC)n | TRUE | NA | 0 | NA | TRUE | FALSE |
| chr3 | 41322319 | 41322320 | 5 | 3 | L1PA6 | TRUE | NA | 0 | NA | FALSE | FALSE |
| chr3 | 41689767 | 41689828 | 4 | 4 | L1PA4 | TRUE | YY1 | 1 | 1 | TRUE | FALSE |
| chr3 | 41819297 | 41819351 | 4 | 4 | L1HS | TRUE | NA | 0 | 1 | FALSE | FALSE |
| chr3 | 42012490 | 42012542 | 4 | 4 | AluSp | TRUE | NA | 0 | NA | FALSE | FALSE |
| chr3 | 42326773 | 42326801 | 12 | 8 | L1PA3 | TRUE | NA | 0 | NA | FALSE | FALSE |
| chr3 | 43095330 | 43095340 | 4 | 4 | Charlie15a | TRUE | NA | 0 | 1 | FALSE | FALSE |
| chr3 | 43559929 | 43559969 | 4 | 3 | MER11B | TRUE | NA | 0 | NA | FALSE | FALSE |
| chr3 | 45017263 | 45017265 | 4 | 4 | (TC)n | TRUE | NA | 0 | NA | TRUE | FALSE |
| chr3 | 45223971 | 45223984 | 5 | 3 | AluSx | TRUE | NA | 0 | NA | FALSE | FALSE |
| chr3 | 46952110 | 46952172 | 4 | 3 | NA | FALSE | NA | 0 | 1 | FALSE | FALSE |
| chr3 | 47358086 | 47358160 | 6 | 5 | (TA)n | TRUE | NA | 0 | NA | FALSE | FALSE |
| chr3 | 47598129 | 47598232 | 4 | 3 | MamRep605 | TRUE | NA | 0 | 1 | TRUE | FALSE |
| chr3 | 48525103 | 48525173 | 5 | 3 | SVA D | TRUE | NA | 0 | 1 | FALSE | FALSE |
| chr3 | 49469453 | 49469512 | 4 | 2 | AluSc8 | TRUE | MANY | 2 | 1 | FALSE | FALSE |
| chr3 | 49823984 | 49824103 | 6 | 5 | NA | FALSE | MANY | 44 | 1 | TRUE | TRUE |
| chr3 | 49893997 | 49894114 | 8 | 7 | NA | FALSE | MANY | 30 | 1 | TRUE | TRUE |
| chr3 | 50122220 | 50122290 | 4 | 4 | SVA D | TRUE | NA | 0 | NA | FALSE | FALSE |
| chr3 | 50349266 | 50349270 | 4 | 4 | SVA D | TRUE | NA | 0 | NA | FALSE | FALSE |
| chr3 | 50349524 | 50349573 | 7 | 6 | SVA D | TRUE | NA | 0 | NA | FALSE | FALSE |
| chr3 | 50910504 | 50910511 | 5 | 3 | L1MA2 | TRUE | NA | 0 | 1 | FALSE | FALSE |
| chr3 | 51286549 | 51286572 | 4 | 3 | SVA D | TRUE | NA | 0 | NA | FALSE | FALSE |
| chr3 | 51809901 | 51809958 | 4 | 4 | NA | FALSE | NA | 0 | 1 | FALSE | FALSE |
| chr3 | 52029908 | 52029959 | 5 | 5 | NA | FALSE | MANY | 54 | 1 | TRUE | TRUE |
| chr3 | 52145212 | 52145213 | 4 | 3 | NA | FALSE | NA | 0 | 1 | FALSE | FALSE |
| chr3 | 52322051 | 52322051 | 4 | 4 | NA | FALSE | MANY | 23 | 1 | TRUE | TRUE |
| chr3 | 52827646 | 52827722 | 4 | 2 | NA | FALSE | NA | 0 | 1 | TRUE | FALSE |
| chr3 | 54113820 | 54113821 | 4 | 3 | NA | FALSE | NA | 0 | 1 | FALSE | FALSE |
| chr3 | 54299681 | 54299682 | 4 | 2 | (TA)n | TRUE | NA | 0 | NA | FALSE | FALSE |
| chr3 | 54565504 | 54565515 | 4 | 3 | NA | FALSE | NA | 0 | 1 | FALSE | FALSE |
| chr3 | 54635379 | 54635388 | 4 | 2 | AluJo | TRUE | NA | 0 | 1 | FALSE | FALSE |
| chr3 | 55814318 | 55814342 | 4 | 3 | NA | FALSE | NA | 0 | 1 | FALSE | FALSE |
| chr3 | 56047724 | 56047742 | 4 | 4 | MIR3 | TRUE | NA | 0 | 1 | FALSE | FALSE |
| chr3 | 56617754 | 56617792 | 5 | 5 | SVA C | TRUE | NA | 0 | 1 | FALSE | FALSE |
| chr3 | 58652699 | 58652766 | 4 | 2 | NA | FALSE | c-Fos | 1 | 1 | TRUE | FALSE |
| chr3 | 58751217 | 58751240 | 4 | 4 | (A)n | TRUE | NA | 0 | 1 | FALSE | FALSE |
| chr3 | 61179728 | 61179728 | 4 | 4 | MER39 | TRUE | NA | 0 | 1 | FALSE | FALSE |
| chr3 | 61264348 | 61264365 | 4 | 2 | (CA)n | TRUE | NA | 0 | NA | FALSE | FALSE |
| chr3 | 63184460 | 63184518 | 4 | 4 | (TA)n | TRUE | NA | 0 | NA | FALSE | FALSE |
| chr3 | 63473657 | 63473703 | 4 | 2 | NA | FALSE | NA | 0 | 1 | FALSE | FALSE |
| chr3 | 64334920 | 64334922 | 4 | 4 | (TG)n | TRUE | NA | 0 | NA | TRUE | FALSE |
| chr3 | 64475972 | 64476011 | 4 | 3 | MER45C | TRUE | GATA-1 | 1 | 1 | TRUE | FALSE |
| chr3 | 65272857 | 65272894 | 4 | 4 | MIRc | TRUE | NA | 0 | 1 | FALSE | FALSE |
| chr3 | 65798119 | 65798144 | 4 | 4 | (TC)n | TRUE | NA | 0 | NA | FALSE | FALSE |
| chr3 | 65850265 | 65850292 | 4 | 4 | NA | FALSE | NA | 0 | 1 | FALSE | FALSE |
| chr3 | 66687050 | 66687077 | 4 | 4 | NA | FALSE | NA | 0 | 1 | TRUE | FALSE |
| chr3 | 66800719 | 66800746 | 4 | 3 | NA | FALSE | NA | 0 | 1 | FALSE | FALSE |
| chr3 | 66943413 | 66943435 | 4 | 3 | L1PA8 | TRUE | NA | 0 | NA | FALSE | FALSE |
| chr3 | 67170932 | 67170949 | 4 | 3 | NA | FALSE | NA | 0 | 1 | FALSE | FALSE |
| chr3 | 68044871 | 68044872 | 4 | 4 | NA | FALSE | NA | 0 | 1 | FALSE | FALSE |
| chr3 | 68407292 | 68407298 | 4 | 3 | NA | FALSE | NA | 0 | 1 | FALSE | FALSE |
| chr3 | 68425455 | 68425456 | 4 | 4 | L1PA13 | TRUE | NA | 0 | 1 | FALSE | FALSE |
| chr3 | 68484731 | 68484732 | 4 | 4 | NA | FALSE | NA | 0 | 1 | FALSE | FALSE |
| chr3 | 68584978 | 68584995 | 5 | 3 | LTR56 | TRUE | NA | 0 | 1 | TRUE | FALSE |
| chr3 | 68611909 | 68611915 | 4 | 4 | GA-rich | TRUE | NA | 0 | 1 | FALSE | FALSE |
| chr3 | 68684755 | 68684772 | 4 | 4 | NA | FALSE | NA | 0 | 1 | FALSE | FALSE |
| chr3 | 68738584 | 68738585 | 4 | 3 | L1MB4 | TRUE | NA | 0 | 1 | FALSE | FALSE |
| chr3 | 69606518 | 69606604 | 4 | 4 | NA | FALSE | NA | 0 | 1 | FALSE | FALSE |
| chr3 | 69684056 | 69684134 | 4 | 3 | L1PA6 | TRUE | NA | 0 | NA | FALSE | FALSE |
| chr3 | 70153720 | 70153758 | 4 | 3 | NA | FALSE | NA | 0 | 1 | FALSE | FALSE |
| chr3 | 71555628 | 71555648 | 4 | 2 | NA | FALSE | NA | 0 | 1 | TRUE | FALSE |
| chr3 | 71793630 | 71793646 | 4 | 3 | AluSg | TRUE | NA | 0 | NA | FALSE | FALSE |
| chr3 | 72081830 | 72081879 | 4 | 4 | (TGGGGG)n | TRUE | USF-1 | 1 | 1 | TRUE | FALSE |
| chr3 | 73272119 | 73272171 | 4 | 4 | LTR50 | TRUE | NA | 0 | 1 | FALSE | FALSE |
| chr3 | 73854958 | 73854969 | 4 | 3 | NA | FALSE | NA | 0 | 1 | TRUE | FALSE |
| chr3 | 73861580 | 73861589 | 4 | 4 | MLT1J | TRUE | NA | 0 | 1 | FALSE | FALSE |
| chr3 | 73954333 | 73954369 | 4 | 2 | NA | FALSE | NA | 0 | 1 | FALSE | FALSE |
| chr3 | 73978796 | 73978837 | 4 | 4 | NA | FALSE | NA | 0 | 1 | FALSE | FALSE |
| chr3 | 74180331 | 74180364 | 4 | 4 | THE1B-int | TRUE | NA | 0 | 1 | FALSE | FALSE |
| chr3 | 74211509 | 74211541 | 4 | 3 | L1PA4 | TRUE | NA | 0 | 1 | FALSE | FALSE |
| chr3 | 74823309 | 74823326 | 4 | 3 | NA | FALSE | NA | 0 | 1 | FALSE | FALSE |
| chr3 | 75022632 | 75022653 | 4 | 4 | L1PA8 | TRUE | NA | 0 | NA | FALSE | FALSE |
| chr3 | 75032273 | 75032273 | 5 | 5 | L1PA10 | TRUE | NA | 0 | 1 | FALSE | FALSE |
| chr3 | 75118609 | 75118633 | 4 | 3 | L1PB1 | TRUE | NA | 0 | 1 | FALSE | FALSE |
| chr3 | 75155723 | 75155729 | 4 | 4 | NA | FALSE | NA | 0 | 1 | FALSE | FALSE |
| chr3 | 75446587 | 75446597 | 4 | 2 | AluSz6 | TRUE | NA | 0 | 1 | FALSE | FALSE |
| chr3 | 75510843 | 75510862 | 5 | 4 | NA | FALSE | NA | 0 | 1 | TRUE | FALSE |
| chr3 | 75608233 | 75608238 | 4 | 2 | LTR5A | TRUE | NA | 0 | 1 | FALSE | FALSE |
| chr3 | 75739649 | 75739659 | 4 | 3 | NA | FALSE | NA | 0 | 1 | TRUE | FALSE |
| chr3 | 75805176 | 75805186 | 4 | 3 | Tigger1 | TRUE | NA | 0 | NA | FALSE | FALSE |
| chr3 | 75811935 | 75811944 | 4 | 2 | L1MB2 | TRUE | NA | 0 | NA | FALSE | FALSE |
| chr3 | 75818186 | 75818186 | 6 | 6 | MLT1H1-int | TRUE | NA | 0 | NA | TRUE | FALSE |
| chr3 | 75837885 | 75837892 | 4 | 4 | L1MA3 | TRUE | NA | 0 | NA | FALSE | FALSE |
| chr3 | 75838759 | 75838794 | 6 | 6 | L1MA3 | TRUE | NA | 0 | NA | FALSE | FALSE |
| chr3 | 75840276 | 75840302 | 4 | 4 | L1MA3 | TRUE | NA | 0 | 1 | FALSE | FALSE |
| chr3 | 75842409 | 75842430 | 6 | 5 | NA | FALSE | NA | 0 | NA | FALSE | FALSE |
| chr3 | 75842551 | 75842561 | 4 | 4 | NA | FALSE | NA | 0 | NA | FALSE | FALSE |
| chr3 | 75842582 | 75842588 | 6 | 4 | NA | FALSE | NA | 0 | NA | FALSE | FALSE |
| chr3 | 75843762 | 75843801 | 7 | 3 | L1PA7 | TRUE | NA | 0 | 1 | FALSE | FALSE |
| chr3 | 75845734 | 75845735 | 10 | 10 | L1PA7 | TRUE | NA | 0 | NA | FALSE | FALSE |
| chr3 | 75852510 | 75852510 | 4 | 4 | LTR49-int | TRUE | NA | 0 | NA | FALSE | FALSE |
| chr3 | 75874259 | 75874279 | 5 | 5 | NA | FALSE | NA | 0 | NA | FALSE | FALSE |
| chr3 | 75875739 | 75875781 | 5 | 4 | L1PA4 | TRUE | NA | 0 | NA | FALSE | FALSE |
| chr3 | 75876129 | 75876137 | 6 | 5 | NA | FALSE | NA | 0 | 1 | FALSE | FALSE |
| chr3 | 75877099 | 75877123 | 4 | 3 | NA | FALSE | NA | 0 | 1 | TRUE | FALSE |
| chr3 | 75877159 | 75877183 | 5 | 5 | NA | FALSE | NA | 0 | 1 | TRUE | FALSE |
| chr3 | 75908329 | 75908342 | 4 | 4 | MER5A | TRUE | NA | 0 | NA | FALSE | FALSE |
| chr3 | 75909271 | 75909294 | 5 | 5 | NA | FALSE | NA | 0 | 1 | TRUE | FALSE |
| chr3 | 75909374 | 75909406 | 6 | 6 | NA | FALSE | NA | 0 | 1 | TRUE | FALSE |
| chr3 | 75909555 | 75909560 | 4 | 4 | NA | FALSE | NA | 0 | NA | FALSE | FALSE |
| chr3 | 75911302 | 75911339 | 6 | 5 | NA | FALSE | NA | 0 | 1 | FALSE | FALSE |
| chr3 | 75911651 | 75911667 | 5 | 5 | NA | FALSE | NA | 0 | 1 | FALSE | FALSE |
| chr3 | 75975674 | 75975674 | 4 | 4 | NA | FALSE | NA | 0 | 1 | FALSE | FALSE |
| chr3 | 76082232 | 76082241 | 4 | 4 | NA | FALSE | NA | 0 | 1 | FALSE | FALSE |
| chr3 | 76199097 | 76199117 | 4 | 3 | NA | FALSE | NA | 0 | NA | FALSE | FALSE |
| chr3 | 77213717 | 77213750 | 5 | 3 | SVA F | TRUE | NA | 0 | NA | FALSE | FALSE |
| chr3 | 77714742 | 77714763 | 4 | 3 | L1PA11 | TRUE | NA | 0 | NA | FALSE | FALSE |
| chr3 | 77758429 | 77758435 | 4 | 4 | MSTD | TRUE | NA | 0 | 1 | FALSE | FALSE |
| chr3 | 77833194 | 77833211 | 4 | 3 | NA | FALSE | NA | 0 | 1 | FALSE | FALSE |
| chr3 | 77833587 | 77833614 | 9 | 4 | NA | FALSE | NA | 0 | 1 | TRUE | FALSE |
| chr3 | 78127588 | 78127606 | 4 | 4 | NA | FALSE | NA | 0 | 1 | FALSE | FALSE |
| chr3 | 78272171 | 78272186 | 4 | 2 | MLT1H1 | TRUE | NA | 0 | 1 | FALSE | FALSE |
| chr3 | 78373926 | 78373946 | 4 | 3 | HERVH-int | TRUE | NA | 0 | 1 | TRUE | FALSE |
| chr3 | 78481927 | 78481952 | 4 | 4 | NA | FALSE | NA | 0 | 1 | FALSE | FALSE |
| chr3 | 78557862 | 78557894 | 5 | 2 | MER39 | TRUE | NA | 0 | 1 | FALSE | FALSE |
| chr3 | 80435928 | 80435941 | 4 | 4 | HERVH-int | TRUE | NA | 0 | NA | FALSE | FALSE |
| chr3 | 80929127 | 80929141 | 4 | 3 | AluSz | TRUE | NA | 0 | 1 | FALSE | FALSE |
| chr3 | 80938554 | 80938564 | 4 | 3 | AluSq | TRUE | NA | 0 | NA | FALSE | FALSE |
| chr3 | 81232234 | 81232237 | 4 | 4 | MLT1F2-int | TRUE | NA | 0 | 1 | FALSE | FALSE |
| chr3 | 81236378 | 81236399 | 4 | 4 | MSTB-int | TRUE | NA | 0 | 1 | TRUE | FALSE |
| chr3 | 82163758 | 82163781 | 4 | 3 | L1PA8A | TRUE | NA | 0 | 1 | TRUE | FALSE |
| chr3 | 82644205 | 82644240 | 4 | 3 | THE1B-int | TRUE | NA | 0 | 1 | FALSE | FALSE |
| chr3 | 82764467 | 82764496 | 5 | 5 | LTR12C | TRUE | NA | 0 | 1 | FALSE | FALSE |
| chr3 | 83690673 | 83690673 | 4 | 4 | L1P2 | TRUE | NA | 0 | NA | FALSE | FALSE |
| chr3 | 84215622 | 84215622 | 4 | 4 | L1MB3 | TRUE | NA | 0 | 1 | FALSE | FALSE |
| chr3 | 84649538 | 84649545 | 4 | 4 | NA | FALSE | NA | 0 | 1 | FALSE | FALSE |
| chr3 | 84704471 | 84704493 | 4 | 3 | L1PB4 | TRUE | NA | 0 | 1 | FALSE | FALSE |
| chr3 | 86046231 | 86046247 | 4 | 4 | NA | FALSE | NA | 0 | 1 | FALSE | FALSE |
| chr3 | 86116276 | 86116289 | 4 | 4 | NA | FALSE | NA | 0 | 1 | FALSE | FALSE |
| chr3 | 86166587 | 86166600 | 4 | 3 | LOR1-int | TRUE | NA | 0 | 1 | FALSE | FALSE |
| chr3 | 86214910 | 86214910 | 4 | 4 | NA | FALSE | NA | 0 | 1 | FALSE | FALSE |
| chr3 | 86393091 | 86393101 | 4 | 4 | AluSp | TRUE | NA | 0 | NA | FALSE | FALSE |
| chr3 | 86547787 | 86547806 | 4 | 3 | NA | FALSE | NA | 0 | 1 | FALSE | FALSE |
| chr3 | 86586712 | 86586724 | 4 | 4 | HERVH-int | TRUE | NA | 0 | 1 | FALSE | FALSE |
| chr3 | 87393663 | 87393665 | 4 | 2 | L1PA3 | TRUE | NA | 0 | NA | FALSE | FALSE |
| chr3 | 87473645 | 87473666 | 4 | 3 | LTR16A | TRUE | NA | 0 | 1 | FALSE | FALSE |
| chr3 | 87601224 | 87601248 | 4 | 3 | NA | FALSE | NA | 0 | 1 | FALSE | FALSE |
| chr3 | 88108325 | 88108350 | 4 | 4 | NA | FALSE | MANY | 26 | 1 | TRUE | TRUE |
| chr3 | 88352342 | 88352349 | 4 | 3 | SVA A | TRUE | NA | 0 | NA | TRUE | FALSE |
| chr3 | 88374427 | 88374439 | 4 | 3 | NA | FALSE | NA | 0 | 1 | FALSE | FALSE |
| chr3 | 88641847 | 88641875 | 4 | 2 | L2 | TRUE | NA | 0 | 1 | FALSE | FALSE |
| chr3 | 89453927 | 89453956 | 4 | 4 | NA | FALSE | NA | 0 | 1 | FALSE | FALSE |
| chr3 | 93553918 | 93553924 | 4 | 3 | L1PA4 | TRUE | NA | 0 | NA | FALSE | FALSE |
| chr3 | 93663411 | 93663460 | 5 | 5 | L1P1 | TRUE | NA | 0 | NA | FALSE | FALSE |
| chr3 | 94013188 | 94013209 | 4 | 4 | NA | FALSE | NA | 0 | 1 | TRUE | FALSE |
| chr3 | 94227988 | 94227998 | 4 | 4 | NA | FALSE | NA | 0 | 1 | FALSE | FALSE |
| chr3 | 94395484 | 94395485 | 4 | 3 | L1PA10 | TRUE | NA | 0 | NA | FALSE | FALSE |
| chr3 | 94449413 | 94449430 | 4 | 3 | HERVL-int | TRUE | NA | 0 | 1 | TRUE | FALSE |
| chr3 | 94609718 | 94609735 | 4 | 3 | PRIMAX-int | TRUE | NA | 0 | 1 | FALSE | FALSE |
| chr3 | 95650577 | 95650578 | 4 | 3 | AluSz6 | TRUE | NA | 0 | NA | FALSE | FALSE |
| chr3 | 95829462 | 95829464 | 4 | 4 | NA | FALSE | NA | 0 | 1 | FALSE | FALSE |
| chr3 | 95935691 | 95935692 | 4 | 2 | MER66-int | TRUE | NA | 0 | 1 | FALSE | FALSE |
| chr3 | 96047117 | 96047119 | 5 | 5 | (TG)n | TRUE | NA | 0 | NA | FALSE | FALSE |
| chr3 | 96269007 | 96269013 | 5 | 3 | L1PA7 | TRUE | NA | 0 | NA | FALSE | FALSE |
| chr3 | 96825712 | 96825718 | 5 | 5 | LTR1 | TRUE | NA | 0 | 1 | FALSE | FALSE |
| chr3 | 97222628 | 97222649 | 4 | 3 | NA | FALSE | NA | 0 | 1 | FALSE | FALSE |
| chr3 | 97381430 | 97381440 | 4 | 3 | NA | FALSE | NA | 0 | 1 | TRUE | FALSE |
| chr3 | 97427467 | 97427476 | 4 | 4 | THE1D-int | TRUE | NA | 0 | NA | FALSE | FALSE |
| chr3 | 97834668 | 97834700 | 4 | 4 | L1M4c | TRUE | NA | 0 | 1 | FALSE | FALSE |
| chr3 | 97881316 | 97881316 | 4 | 4 | MLT1A1-int | TRUE | NA | 0 | 1 | FALSE | FALSE |
| chr3 | 97881991 | 97882020 | 4 | 3 | MLT1A1-int | TRUE | NA | 0 | NA | FALSE | FALSE |
| chr3 | 97921904 | 97921907 | 4 | 4 | MLT1A1 | TRUE | NA | 0 | 1 | FALSE | FALSE |
| chr3 | 98622413 | 98622433 | 4 | 2 | AluSp | TRUE | NA | 0 | NA | FALSE | FALSE |
| chr3 | 99289760 | 99289773 | 4 | 3 | NA | FALSE | NA | 0 | 1 | FALSE | FALSE |
| chr3 | 99834428 | 99834450 | 4 | 4 | (TC)n | TRUE | NA | 0 | NA | FALSE | FALSE |
| chr3 | 99948207 | 99948285 | 4 | 4 | GA-rich | TRUE | NA | 0 | 1 | FALSE | FALSE |
| chr3 | 101181221 | 101181221 | 6 | 6 | AluSp | TRUE | NA | 0 | NA | FALSE | FALSE |
| chr3 | 101280669 | 101280680 | 11 | 8 | NA | FALSE | MANY | 43 | 1 | TRUE | TRUE |
| chr3 | 102086675 | 102086675 | 5 | 5 | L1PA7 | TRUE | NA | 0 | NA | FALSE | FALSE |
| chr3 | 102086707 | 102086707 | 6 | 6 | L1PA7 | TRUE | NA | 0 | 1 | FALSE | FALSE |
| chr3 | 102224555 | 102224568 | 4 | 4 | THE1C-int | TRUE | NA | 0 | NA | FALSE | FALSE |
| chr3 | 102631903 | 102631912 | 4 | 3 | AluSx3 | TRUE | NA | 0 | 1 | FALSE | FALSE |
| chr3 | 102904084 | 102904095 | 4 | 3 | L1M4c | TRUE | NA | 0 | 1 | FALSE | FALSE |
| chr3 | 103182174 | 103182191 | 4 | 4 | NA | FALSE | NA | 0 | 1 | FALSE | FALSE |
| chr3 | 103343263 | 103343264 | 4 | 3 | L1MA4 | TRUE | p300 | 1 | 1 | TRUE | FALSE |
| chr3 | 103912513 | 103912532 | 4 | 3 | MER21C | TRUE | NA | 0 | 1 | FALSE | FALSE |
| chr3 | 104911874 | 104911926 | 6 | 5 | L1PA8 | TRUE | NA | 0 | 1 | TRUE | FALSE |
| chr3 | 105473340 | 105473380 | 4 | 3 | (GA)n | TRUE | ZZZ3 | 1 | NA | FALSE | FALSE |
| chr3 | 106073523 | 106073554 | 4 | 4 | MSTA | TRUE | NA | 0 | 1 | FALSE | FALSE |
| chr3 | 106317668 | 106317674 | 4 | 3 | L1MA6 | TRUE | NA | 0 | 1 | FALSE | FALSE |
| chr3 | 106383266 | 106383285 | 4 | 4 | L1M1 | TRUE | NA | 0 | 1 | FALSE | FALSE |
| chr3 | 106575078 | 106575085 | 6 | 6 | L1PA8 | TRUE | NA | 0 | 1 | FALSE | FALSE |
| chr3 | 106725584 | 106725603 | 4 | 4 | NA | FALSE | NA | 0 | 1 | FALSE | FALSE |
| chr3 | 106795120 | 106795120 | 4 | 4 | (A)n | TRUE | MANY | 2 | NA | TRUE | FALSE |
| chr3 | 107323589 | 107323589 | 4 | 4 | NA | FALSE | NA | 0 | 1 | TRUE | FALSE |
| chr3 | 107970819 | 107970820 | 4 | 3 | L1MD1 | TRUE | NA | 0 | 1 | FALSE | FALSE |
| chr3 | 108625688 | 108625699 | 4 | 4 | NA | FALSE | NA | 0 | 1 | FALSE | FALSE |
| chr3 | 108815406 | 108815425 | 4 | 4 | MER41B | TRUE | MANY | 2 | 1 | FALSE | FALSE |
| chr3 | 108890820 | 108890841 | 6 | 4 | AluSc | TRUE | NA | 0 | NA | FALSE | FALSE |
| chr3 | 108967775 | 108967777 | 4 | 4 | AluSx3 | TRUE | NA | 0 | NA | FALSE | FALSE |
| chr3 | 109197395 | 109197402 | 4 | 2 | NA | FALSE | NA | 0 | 1 | FALSE | FALSE |
| chr3 | 109515330 | 109515360 | 4 | 4 | L1PA7 | TRUE | NA | 0 | NA | FALSE | FALSE |
| chr3 | 109756992 | 109757025 | 5 | 3 | NA | FALSE | NA | 0 | 1 | FALSE | FALSE |
| chr3 | 110159126 | 110159158 | 4 | 4 | L1PA2 | TRUE | NA | 0 | NA | TRUE | FALSE |
| chr3 | 110636180 | 110636180 | 4 | 4 | L1PA3 | TRUE | NA | 0 | NA | FALSE | FALSE |
| chr3 | 112385234 | 112385288 | 4 | 4 | NA | FALSE | NA | 0 | 1 | FALSE | FALSE |
| chr3 | 112435126 | 112435183 | 4 | 3 | AluSx1 | TRUE | NA | 0 | NA | FALSE | FALSE |
| chr3 | 112594718 | 112594769 | 4 | 4 | L1MB5 | TRUE | NA | 0 | 1 | TRUE | FALSE |
| chr3 | 112667160 | 112667171 | 4 | 2 | NA | FALSE | NA | 0 | 1 | FALSE | FALSE |
| chr3 | 113171789 | 113171803 | 5 | 5 | AluSg | TRUE | NA | 0 | NA | FALSE | FALSE |
| chr3 | 113433125 | 113433201 | 5 | 2 | AluY | TRUE | NA | 0 | 1 | FALSE | FALSE |
| chr3 | 113922715 | 113922764 | 4 | 3 | L1MEf | TRUE | NA | 0 | 1 | FALSE | FALSE |
| chr3 | 113964306 | 113964372 | 4 | 4 | MSTA-int | TRUE | NA | 0 | 1 | TRUE | FALSE |
| chr3 | 114242707 | 114242753 | 4 | 2 | L2a | TRUE | NA | 0 | 1 | FALSE | FALSE |
| chr3 | 114327300 | 114327336 | 4 | 3 | MER5A | TRUE | MANY | 6 | 1 | TRUE | TRUE |
| chr3 | 114717886 | 114717956 | 5 | 3 | L2 | TRUE | NA | 0 | 1 | FALSE | FALSE |
| chr3 | 115009294 | 115009327 | 4 | 4 | Plat L3 | TRUE | NA | 0 | 1 | FALSE | FALSE |
| chr3 | 115085077 | 115085078 | 4 | 3 | L1PA11 | TRUE | NA | 0 | NA | FALSE | FALSE |
| chr3 | 115287099 | 115287117 | 4 | 2 | AluSq4 | TRUE | NA | 0 | NA | FALSE | FALSE |
| chr3 | 115450399 | 115450443 | 4 | 2 | L1PA10 | TRUE | NA | 0 | 1 | FALSE | FALSE |
| chr3 | 115659229 | 115659254 | 5 | 3 | LTR12 | TRUE | NA | 0 | NA | FALSE | FALSE |
| chr3 | 116303654 | 116303700 | 4 | 4 | NA | FALSE | NA | 0 | 1 | FALSE | FALSE |
| chr3 | 118062251 | 118062251 | 4 | 4 | L1ME4a | TRUE | NA | 0 | 1 | FALSE | FALSE |
| chr3 | 118103400 | 118103401 | 4 | 2 | THE1A-int | TRUE | NA | 0 | NA | FALSE | FALSE |
| chr3 | 118423667 | 118423690 | 4 | 4 | C-rich | TRUE | NA | 0 | 1 | FALSE | FALSE |
| chr3 | 118510912 | 118510922 | 4 | 4 | NA | FALSE | NA | 0 | 1 | FALSE | FALSE |
| chr3 | 118796447 | 118796451 | 4 | 2 | L1PA15 | TRUE | NA | 0 | NA | FALSE | FALSE |
| chr3 | 120200377 | 120200393 | 5 | 5 | (CA)n | TRUE | NA | 0 | NA | FALSE | FALSE |
| chr3 | 120759994 | 120760006 | 4 | 2 | L1M5 | TRUE | NA | 0 | 1 | FALSE | FALSE |
| chr3 | 120956219 | 120956242 | 5 | 4 | MSTB1-int | TRUE | NA | 0 | 1 | FALSE | FALSE |
| chr3 | 121004866 | 121004866 | 4 | 4 | NA | FALSE | NA | 0 | 1 | FALSE | FALSE |
| chr3 | 121074625 | 121074641 | 4 | 4 | L1PA6 | TRUE | NA | 0 | NA | FALSE | FALSE |
| chr3 | 121793487 | 121793509 | 4 | 3 | NA | FALSE | MANY | 3 | 1 | TRUE | TRUE |
| chr3 | 121858838 | 121858838 | 5 | 5 | L1PA4 | TRUE | NA | 0 | NA | FALSE | FALSE |
| chr3 | 121877174 | 121877195 | 5 | 3 | AluSq2 | TRUE | NA | 0 | NA | FALSE | FALSE |
| chr3 | 123174262 | 123174311 | 4 | 2 | NA | FALSE | NA | 0 | 1 | TRUE | FALSE |
| chr3 | 123544289 | 123544333 | 4 | 3 | L1MA5A | TRUE | NA | 0 | 1 | FALSE | FALSE |
| chr3 | 124392378 | 124392468 | 7 | 5 | (TA)n | TRUE | NA | 0 | NA | FALSE | FALSE |
| chr3 | 124473358 | 124473417 | 4 | 2 | HERVH-int | TRUE | NA | 0 | NA | FALSE | FALSE |
| chr3 | 124638199 | 124638202 | 4 | 2 | AluSg | TRUE | NA | 0 | NA | FALSE | FALSE |
| chr3 | 124689555 | 124689608 | 4 | 2 | NA | FALSE | NA | 0 | 1 | TRUE | FALSE |
| chr3 | 125314437 | 125314438 | 6 | 5 | NA | FALSE | MANY | 18 | 1 | TRUE | TRUE |
| chr3 | 125450138 | 125450162 | 4 | 4 | LTR5A | TRUE | NA | 0 | NA | FALSE | FALSE |
| chr3 | 125455603 | 125455638 | 4 | 4 | SATR2 | TRUE | NA | 0 | 1 | FALSE | FALSE |
| chr3 | 125483483 | 125483531 | 4 | 4 | ERVL-B4-int | TRUE | NA | 0 | 1 | TRUE | FALSE |
| chr3 | 125483602 | 125483610 | 5 | 3 | ERVL-B4-int | TRUE | NA | 0 | 1 | TRUE | FALSE |
| chr3 | 125511133 | 125511188 | 4 | 3 | Charlie18a | TRUE | NA | 0 | NA | FALSE | FALSE |
| chr3 | 125512122 | 125512175 | 4 | 4 | NA | FALSE | NA | 0 | NA | FALSE | FALSE |
| chr3 | 125541043 | 125541083 | 6 | 5 | MLT1F2 | TRUE | NA | 0 | 1 | FALSE | FALSE |
| chr3 | 125560929 | 125560980 | 5 | 3 | NA | FALSE | NA | 0 | 1 | FALSE | FALSE |
| chr3 | 125612031 | 125612086 | 4 | 3 | HERVK-int | TRUE | NA | 0 | 1 | FALSE | FALSE |
| chr3 | 126580998 | 126581036 | 4 | 3 | (CCA)n | TRUE | NA | 0 | NA | FALSE | FALSE |
| chr3 | 126582110 | 126582157 | 4 | 4 | MANY | TRUE | NA | 0 | NA | FALSE | FALSE |
| chr3 | 127281445 | 127281447 | 4 | 2 | NA | FALSE | NA | 0 | 1 | TRUE | FALSE |
| chr3 | 127696513 | 127696572 | 4 | 3 | L1PA15 | TRUE | NA | 0 | 1 | FALSE | FALSE |
| chr3 | 127699364 | 127699368 | 6 | 3 | AluSz | TRUE | NA | 0 | NA | FALSE | FALSE |
| chr3 | 127705512 | 127705597 | 6 | 4 | AluSc | TRUE | NA | 0 | NA | TRUE | FALSE |
| chr3 | 128418082 | 128418098 | 4 | 3 | AluSx | TRUE | NA | 0 | NA | TRUE | FALSE |
| chr3 | 128880190 | 128880220 | 4 | 3 | NA | FALSE | MANY | 41 | 1 | TRUE | TRUE |
| chr3 | 129196385 | 129196456 | 4 | 2 | NA | FALSE | NA | 0 | 1 | TRUE | FALSE |
| chr3 | 129734958 | 129734962 | 5 | 5 | LTR5A | TRUE | NA | 0 | NA | FALSE | FALSE |
| chr3 | 129779497 | 129779504 | 4 | 4 | HERVH-int | TRUE | NA | 0 | NA | FALSE | FALSE |
| chr3 | 129852749 | 129852760 | 10 | 7 | (TC)n | TRUE | NA | 0 | NA | FALSE | FALSE |
| chr3 | 129890916 | 129890924 | 8 | 7 | NA | FALSE | NA | 0 | 1 | FALSE | FALSE |
| chr3 | 129893375 | 129893419 | 4 | 4 | AluSx1 | TRUE | NA | 0 | NA | FALSE | FALSE |
| chr3 | 130292940 | 130292985 | 4 | 3 | NA | FALSE | NA | 0 | 1 | FALSE | FALSE |
| chr3 | 130928410 | 130928436 | 4 | 3 | L1PA6 | TRUE | NA | 0 | NA | FALSE | FALSE |
| chr3 | 131810942 | 131810971 | 4 | 3 | L2c | TRUE | NA | 0 | 1 | FALSE | FALSE |
| chr3 | 131963962 | 131963997 | 4 | 3 | AT rich | TRUE | NA | 0 | 1 | FALSE | FALSE |
| chr3 | 132516662 | 132516663 | 4 | 2 | L1PA4 | TRUE | NA | 0 | NA | FALSE | FALSE |
| chr3 | 132677161 | 132677201 | 5 | 4 | L1PA7 | TRUE | NA | 0 | NA | FALSE | FALSE |
| chr3 | 132731667 | 132731704 | 4 | 4 | THE1A | TRUE | NA | 0 | 1 | FALSE | FALSE |
| chr3 | 133788611 | 133788637 | 4 | 2 | LTR12C | TRUE | NF-YA | 1 | NA | FALSE | FALSE |
| chr3 | 134465246 | 134465246 | 4 | 4 | L1PA6 | TRUE | NA | 0 | NA | FALSE | FALSE |
| chr3 | 134630673 | 134630711 | 4 | 3 | NA | FALSE | NA | 0 | 1 | TRUE | FALSE |
| chr3 | 134781031 | 134781056 | 4 | 3 | L1PB1 | TRUE | NA | 0 | 1 | FALSE | FALSE |
| chr3 | 135422046 | 135422058 | 4 | 3 | NA | FALSE | NA | 0 | 1 | TRUE | FALSE |
| chr3 | 136622371 | 136622425 | 6 | 2 | AluSq2 | TRUE | NA | 0 | NA | FALSE | FALSE |
| chr3 | 136710685 | 136710705 | 6 | 3 | AluSx1 | TRUE | NA | 0 | NA | FALSE | FALSE |
| chr3 | 136904604 | 136904641 | 4 | 4 | NA | FALSE | NA | 0 | 1 | TRUE | FALSE |
| chr3 | 138205626 | 138205654 | 4 | 2 | MER11A | TRUE | MANY | 2 | NA | FALSE | FALSE |
| chr3 | 138723906 | 138723948 | 4 | 2 | NA | FALSE | NA | 0 | 1 | TRUE | FALSE |
| chr3 | 138750233 | 138750266 | 5 | 5 | L1M3 | TRUE | NA | 0 | 1 | FALSE | FALSE |
| chr3 | 138774603 | 138774620 | 4 | 3 | L1M2 | TRUE | NA | 0 | 1 | FALSE | FALSE |
| chr3 | 139603927 | 139603947 | 5 | 3 | L1P3 | TRUE | NA | 0 | NA | FALSE | FALSE |
| chr3 | 139691003 | 139691019 | 6 | 3 | AluSx1 | TRUE | NA | 0 | NA | FALSE | FALSE |
| chr3 | 140028560 | 140028588 | 4 | 3 | NA | FALSE | NA | 0 | 1 | FALSE | FALSE |
| chr3 | 140129045 | 140129067 | 4 | 2 | L1MA9 | TRUE | NA | 0 | 1 | FALSE | FALSE |
| chr3 | 140204053 | 140204085 | 5 | 5 | L1PA4 | TRUE | NA | 0 | NA | FALSE | FALSE |
| chr3 | 140204143 | 140204155 | 5 | 5 | L1PA4 | TRUE | NA | 0 | NA | FALSE | FALSE |
| chr3 | 140218688 | 140218717 | 4 | 3 | AluY | TRUE | NA | 0 | NA | FALSE | FALSE |
| chr3 | 140738294 | 140738341 | 4 | 3 | NA | FALSE | NA | 0 | 1 | TRUE | FALSE |
| chr3 | 140923957 | 140923996 | 4 | 3 | LTR7 | TRUE | NA | 0 | NA | FALSE | FALSE |
| chr3 | 141504528 | 141504590 | 4 | 4 | AluSx1 | TRUE | NA | 0 | 1 | FALSE | FALSE |
| chr3 | 141987135 | 141987230 | 4 | 3 | L1MC4 | TRUE | NA | 0 | 1 | FALSE | FALSE |
| chr3 | 142556026 | 142556066 | 4 | 1 | MLT1D | TRUE | NA | 0 | 1 | FALSE | FALSE |
| chr3 | 144224004 | 144224004 | 4 | 4 | HERVL-int | TRUE | NA | 0 | NA | FALSE | FALSE |
| chr3 | 144432721 | 144432733 | 4 | 3 | L1MA8 | TRUE | NA | 0 | 1 | FALSE | FALSE |
| chr3 | 144434438 | 144434458 | 4 | 3 | THE1A-int | TRUE | NA | 0 | 1 | FALSE | FALSE |
| chr3 | 144517042 | 144517044 | 4 | 3 | THE1B | TRUE | NA | 0 | NA | FALSE | FALSE |
| chr3 | 144689747 | 144689752 | 4 | 4 | NA | FALSE | NA | 0 | 1 | FALSE | FALSE |
| chr3 | 145028606 | 145028606 | 4 | 4 | AluSx | TRUE | NA | 0 | 1 | FALSE | FALSE |
| chr3 | 147075951 | 147075978 | 4 | 3 | NA | FALSE | NA | 0 | 1 | TRUE | FALSE |
| chr3 | 147256760 | 147256784 | 7 | 3 | MLT2A2 | TRUE | NA | 0 | NA | FALSE | FALSE |
| chr3 | 147724762 | 147724796 | 5 | 5 | CT-rich | TRUE | NA | 0 | NA | FALSE | FALSE |
| chr3 | 147985736 | 147985742 | 4 | 4 | MANY | TRUE | NA | 0 | NA | TRUE | FALSE |
| chr3 | 148236864 | 148236886 | 4 | 3 | L1M1 | TRUE | NA | 0 | 1 | FALSE | FALSE |
| chr3 | 149083224 | 149083224 | 5 | 5 | (TG)n | TRUE | NA | 0 | NA | FALSE | FALSE |
| chr3 | 150549529 | 150549529 | 4 | 4 | L2a | TRUE | NA | 0 | NA | FALSE | FALSE |
| chr3 | 151909418 | 151909424 | 4 | 2 | (TA)n | TRUE | NA | 0 | NA | FALSE | FALSE |
| chr3 | 152431193 | 152431211 | 4 | 3 | LTR2 | TRUE | NA | 0 | NA | FALSE | FALSE |
| chr3 | 153226939 | 153226970 | 6 | 5 | L1PA2 | TRUE | NA | 0 | 1 | FALSE | FALSE |
| chr3 | 154156454 | 154156476 | 4 | 4 | L1PA4 | TRUE | NA | 0 | NA | FALSE | FALSE |
| chr3 | 154262380 | 154262397 | 4 | 3 | NA | FALSE | NA | 0 | 1 | FALSE | FALSE |
| chr3 | 154445378 | 154445389 | 4 | 4 | NA | FALSE | NA | 0 | 1 | FALSE | FALSE |
| chr3 | 155018766 | 155018780 | 4 | 2 | NA | FALSE | NA | 0 | 1 | FALSE | FALSE |
| chr3 | 155968693 | 155968733 | 4 | 3 | NA | FALSE | NA | 0 | 1 | FALSE | FALSE |
| chr3 | 155975228 | 155975265 | 4 | 4 | NA | FALSE | NA | 0 | 1 | FALSE | FALSE |
| chr3 | 155989456 | 155989496 | 4 | 2 | L1MA2 | TRUE | MANY | 2 | 1 | FALSE | FALSE |
| chr3 | 156493525 | 156493571 | 4 | 3 | AluSx3 | TRUE | NA | 0 | NA | FALSE | FALSE |
| chr3 | 157299630 | 157299630 | 4 | 4 | HERVE a-int | TRUE | CTCF | 1 | 1 | TRUE | FALSE |
| chr3 | 158688299 | 158688306 | 4 | 3 | NA | FALSE | NA | 0 | 1 | FALSE | FALSE |
| chr3 | 158724166 | 158724171 | 4 | 3 | NA | FALSE | NA | 0 | 1 | TRUE | FALSE |
| chr3 | 158742774 | 158742787 | 4 | 4 | L1PA16 | TRUE | NA | 0 | NA | FALSE | FALSE |
| chr3 | 158777905 | 158777922 | 4 | 4 | L2a | TRUE | NA | 0 | 1 | FALSE | FALSE |
| chr3 | 159410655 | 159410660 | 4 | 4 | NA | FALSE | NA | 0 | 1 | FALSE | FALSE |
| chr3 | 159619280 | 159619290 | 4 | 2 | NA | FALSE | NA | 0 | 1 | FALSE | FALSE |
| chr3 | 159701659 | 159701663 | 4 | 3 | L1ME5 | TRUE | NA | 0 | 1 | FALSE | FALSE |
| chr3 | 159719276 | 159719293 | 4 | 4 | Charlie15a | TRUE | NA | 0 | 1 | FALSE | FALSE |
| chr3 | 160372306 | 160372351 | 4 | 2 | L1M1 | TRUE | NA | 0 | 1 | FALSE | FALSE |
| chr3 | 161190888 | 161190901 | 4 | 4 | MER34C | TRUE | NA | 0 | 1 | FALSE | FALSE |
| chr3 | 161262579 | 161262596 | 4 | 4 | L1PA4 | TRUE | NA | 0 | NA | FALSE | FALSE |
| chr3 | 161361063 | 161361083 | 4 | 4 | LTR48 | TRUE | NA | 0 | 1 | FALSE | FALSE |
| chr3 | 161655255 | 161655269 | 4 | 3 | HERVE a-int | TRUE | NA | 0 | 1 | FALSE | FALSE |
| chr3 | 161809118 | 161809130 | 4 | 4 | L1MA1 | TRUE | NA | 0 | 1 | FALSE | FALSE |
| chr3 | 162080483 | 162080483 | 4 | 4 | AT rich | TRUE | NA | 0 | 1 | FALSE | FALSE |
| chr3 | 162680549 | 162680570 | 6 | 2 | L1PA5 | TRUE | NA | 0 | NA | FALSE | FALSE |
| chr3 | 162897973 | 162897988 | 4 | 3 | NA | FALSE | NA | 0 | 1 | FALSE | FALSE |
| chr3 | 163105344 | 163105351 | 4 | 3 | L1MA8 | TRUE | NA | 0 | 1 | FALSE | FALSE |
| chr3 | 163440255 | 163440278 | 4 | 4 | MER52A | TRUE | NA | 0 | 1 | TRUE | FALSE |
| chr3 | 163551943 | 163551951 | 4 | 4 | NA | FALSE | NA | 0 | 1 | FALSE | FALSE |
| chr3 | 164130650 | 164130684 | 5 | 5 | L1HS | TRUE | NA | 0 | 1 | FALSE | FALSE |
| chr3 | 164790991 | 164790999 | 6 | 4 | (TC)n | TRUE | NA | 0 | NA | FALSE | FALSE |
| chr3 | 165847789 | 165847797 | 4 | 4 | L1MA1 | TRUE | NA | 0 | 1 | FALSE | FALSE |
| chr3 | 165961404 | 165961415 | 4 | 3 | NA | FALSE | NA | 0 | 1 | FALSE | FALSE |
| chr3 | 166004558 | 166004581 | 4 | 3 | AluSx1 | TRUE | NA | 0 | NA | FALSE | FALSE |
| chr3 | 166062673 | 166062689 | 4 | 4 | (GGAA)n | TRUE | NA | 0 | NA | TRUE | FALSE |
| chr3 | 167008432 | 167008440 | 4 | 3 | L1PA7 | TRUE | NA | 0 | NA | FALSE | FALSE |
| chr3 | 167159893 | 167159918 | 4 | 3 | NA | FALSE | NA | 0 | 1 | FALSE | FALSE |
| chr3 | 167180276 | 167180290 | 4 | 4 | MLT1B | TRUE | NA | 0 | 1 | FALSE | FALSE |
| chr3 | 168103848 | 168103856 | 5 | 5 | NA | FALSE | NA | 0 | 1 | FALSE | FALSE |
| chr3 | 168249500 | 168249517 | 4 | 3 | NA | FALSE | NA | 0 | 1 | FALSE | FALSE |
| chr3 | 168308348 | 168308360 | 4 | 3 | NA | FALSE | NA | 0 | 1 | FALSE | FALSE |
| chr3 | 168509474 | 168509495 | 4 | 4 | L1MA4 | TRUE | NA | 0 | 1 | FALSE | FALSE |
| chr3 | 169190475 | 169190495 | 4 | 4 | A-rich | TRUE | NA | 0 | NA | FALSE | FALSE |
| chr3 | 169354079 | 169354105 | 4 | 3 | L3 | TRUE | NA | 0 | 1 | FALSE | FALSE |
| chr3 | 169616061 | 169616072 | 4 | 2 | AluY | TRUE | NA | 0 | NA | FALSE | FALSE |
| chr3 | 171110530 | 171110530 | 4 | 4 | AluSx1 | TRUE | NA | 0 | NA | FALSE | FALSE |
| chr3 | 171634595 | 171634677 | 4 | 3 | L1ME3 | TRUE | NA | 0 | 1 | FALSE | FALSE |
| chr3 | 172126041 | 172126082 | 4 | 3 | NA | FALSE | FOSL2 | 1 | 1 | FALSE | FALSE |
| chr3 | 172208414 | 172208478 | 9 | 6 | L1PA8 | TRUE | NA | 0 | NA | FALSE | FALSE |
| chr3 | 172839363 | 172839392 | 4 | 3 | L2c | TRUE | NA | 0 | 1 | FALSE | FALSE |
| chr3 | 174067964 | 174068033 | 4 | 3 | NA | FALSE | NA | 0 | 1 | FALSE | FALSE |
| chr3 | 174778624 | 174778624 | 4 | 4 | AluSp | TRUE | NA | 0 | NA | FALSE | FALSE |
| chr3 | 174849730 | 174849768 | 4 | 3 | SVA D | TRUE | NA | 0 | 1 | FALSE | FALSE |
| chr3 | 175637409 | 175637435 | 4 | 4 | THE1B-int | TRUE | NA | 0 | 1 | FALSE | FALSE |
| chr3 | 175919343 | 175919379 | 6 | 4 | NA | FALSE | NA | 0 | 1 | FALSE | FALSE |
| chr3 | 176041149 | 176041163 | 4 | 4 | HERVH-int | TRUE | NA | 0 | NA | FALSE | FALSE |
| chr3 | 176041210 | 176041219 | 7 | 5 | HERVH-int | TRUE | NA | 0 | 1 | FALSE | FALSE |
| chr3 | 176099479 | 176099479 | 4 | 4 | MLT2B5 | TRUE | NA | 0 | NA | FALSE | FALSE |
| chr3 | 176251636 | 176251661 | 4 | 4 | L1M1 | TRUE | NA | 0 | 1 | FALSE | FALSE |
| chr3 | 176579309 | 176579343 | 4 | 3 | L1PA5 | TRUE | NA | 0 | 1 | FALSE | FALSE |
| chr3 | 178310600 | 178310625 | 4 | 4 | MER5A | TRUE | NA | 0 | 1 | FALSE | FALSE |
| chr3 | 178540108 | 178540138 | 5 | 3 | L2c | TRUE | NA | 0 | 1 | FALSE | FALSE |
| chr3 | 179185100 | 179185132 | 4 | 3 | NA | FALSE | NA | 0 | 1 | FALSE | FALSE |
| chr3 | 180810243 | 180810270 | 4 | 4 | NA | FALSE | NA | 0 | 1 | FALSE | FALSE |
| chr3 | 181737409 | 181737456 | 4 | 3 | NA | FALSE | NA | 0 | 1 | TRUE | FALSE |
| chr3 | 183251844 | 183251874 | 4 | 2 | NA | FALSE | MANY | 12 | 1 | TRUE | TRUE |
| chr3 | 183807068 | 183807105 | 4 | 4 | AluSz | TRUE | NA | 0 | 1 | FALSE | FALSE |
| chr3 | 184522565 | 184522642 | 5 | 4 | AluJr4 | TRUE | NA | 0 | NA | TRUE | FALSE |
| chr3 | 185000640 | 185000731 | 4 | 4 | NA | FALSE | MANY | 34 | 1 | TRUE | TRUE |
| chr3 | 185150177 | 185150188 | 5 | 4 | AluSx | TRUE | NA | 0 | NA | FALSE | FALSE |
| chr3 | 185172740 | 185172837 | 6 | 2 | (TA)n | TRUE | NA | 0 | NA | FALSE | FALSE |
| chr3 | 188773827 | 188773835 | 4 | 3 | (TTCC)n | TRUE | NA | 0 | NA | TRUE | FALSE |
| chr3 | 189862645 | 189862662 | 4 | 3 | LTR7 | TRUE | NA | 0 | NA | FALSE | FALSE |
| chr3 | 190516018 | 190516050 | 4 | 4 | L1M1 | TRUE | NA | 0 | 1 | FALSE | FALSE |
| chr3 | 190638654 | 190638659 | 4 | 4 | HERVH-int | TRUE | NA | 0 | NA | FALSE | FALSE |
| chr3 | 191329561 | 191329561 | 4 | 4 | NA | FALSE | NA | 0 | 1 | FALSE | FALSE |
| chr3 | 191461057 | 191461076 | 4 | 4 | HERVP71A-int | TRUE | NA | 0 | 1 | FALSE | FALSE |
| chr3 | 191566543 | 191566561 | 4 | 3 | L2a | TRUE | NA | 0 | 1 | FALSE | FALSE |
| chr3 | 191984971 | 191984991 | 5 | 5 | NA | FALSE | NA | 0 | 1 | FALSE | FALSE |
| chr3 | 192222757 | 192222761 | 4 | 3 | NA | FALSE | NA | 0 | 1 | FALSE | FALSE |
| chr3 | 192830392 | 192830395 | 4 | 2 | NA | FALSE | NA | 0 | 1 | FALSE | FALSE |
| chr3 | 192994390 | 192994399 | 4 | 3 | NA | FALSE | NA | 0 | 1 | FALSE | FALSE |
| chr3 | 193644219 | 193644248 | 4 | 4 | HERVL-int | TRUE | MANY | 7 | 1 | TRUE | TRUE |
| chr3 | 193699120 | 193699152 | 4 | 2 | NA | FALSE | NA | 0 | 1 | TRUE | FALSE |
| chr3 | 194514825 | 194514896 | 4 | 4 | AluY | TRUE | NA | 0 | 1 | FALSE | FALSE |
| chr3 | 195163894 | 195163951 | 4 | 4 | NA | FALSE | MANY | 33 | 1 | TRUE | TRUE |
| chr3 | 195217984 | 195218029 | 4 | 4 | L1MC1 | TRUE | NA | 0 | NA | FALSE | FALSE |
| chr3 | 195326823 | 195326866 | 5 | 2 | SVA C | TRUE | NA | 0 | NA | FALSE | FALSE |
| chr3 | 195414038 | 195414046 | 4 | 3 | NA | FALSE | NA | 0 | 1 | TRUE | FALSE |
| chr3 | 195437511 | 195437540 | 5 | 5 | NA | FALSE | NA | 0 | 1 | TRUE | FALSE |
| chr3 | 195502683 | 195502730 | 4 | 3 | NA | FALSE | NA | 0 | 1 | FALSE | FALSE |
| chr3 | 195502788 | 195502851 | 5 | 4 | (CATAC)n | TRUE | NA | 0 | 1 | FALSE | FALSE |
| chr3 | 195598832 | 195598833 | 4 | 2 | NA | FALSE | NA | 0 | NA | TRUE | FALSE |
| chr3 | 195665803 | 195665809 | 10 | 5 | NA | FALSE | NA | 0 | NA | FALSE | FALSE |
| chr3 | 195669197 | 195669244 | 4 | 4 | MANY | TRUE | NA | 0 | 1 | FALSE | FALSE |
| chr3 | 195675127 | 195675140 | 5 | 3 | LTR78B | TRUE | NA | 0 | 1 | FALSE | FALSE |
| chr3 | 195677407 | 195677408 | 6 | 4 | L4 | TRUE | NA | 0 | 1 | FALSE | FALSE |
| chr3 | 195677465 | 195677504 | 9 | 8 | L4 | TRUE | NA | 0 | 1 | FALSE | FALSE |
| chr3 | 195682183 | 195682226 | 5 | 4 | AluSp | TRUE | NA | 0 | NA | FALSE | FALSE |
| chr3 | 195687624 | 195687669 | 4 | 4 | MER1B | TRUE | NA | 0 | 1 | TRUE | FALSE |
| chr3 | 195688781 | 195688810 | 6 | 6 | MER1B | TRUE | NA | 0 | NA | TRUE | FALSE |
| chr3 | 195722156 | 195722163 | 5 | 3 | L1M5 | TRUE | NA | 0 | 1 | TRUE | FALSE |
| chr3 | 195872113 | 195872158 | 6 | 6 | NA | FALSE | Pbx3 | 1 | NA | FALSE | FALSE |
| chr3 | 195880862 | 195880900 | 4 | 3 | L1P4 | TRUE | NA | 0 | NA | FALSE | FALSE |
| chr3 | 195963683 | 195963697 | 4 | 2 | AluSx1 | TRUE | NA | 0 | NA | FALSE | FALSE |
| chr3 | 196274412 | 196274481 | 4 | 2 | THE1B | TRUE | NA | 0 | 1 | FALSE | FALSE |
| chr3 | 196509521 | 196509576 | 4 | 2 | NA | FALSE | NA | 0 | 1 | TRUE | FALSE |
| chr3 | 196761537 | 196761619 | 4 | 4 | NA | FALSE | NA | 0 | NA | FALSE | FALSE |
| chr3 | 197164102 | 197164123 | 4 | 3 | NA | FALSE | NA | 0 | NA | FALSE | FALSE |
| chr3 | 197308838 | 197308921 | 5 | 5 | NA | FALSE | NA | 0 | 1 | TRUE | FALSE |
| chr3 | 197345478 | 197345537 | 4 | 4 | L1MA9 | TRUE | NA | 0 | 1 | TRUE | FALSE |
| chr3 | 197385836 | 197385902 | 4 | 3 | NA | FALSE | NA | 0 | 1 | TRUE | FALSE |
| chr3 | 197648641 | 197648714 | 5 | 4 | NA | FALSE | NFKB | 1 | 1 | TRUE | FALSE |
| chr3 | 197739967 | 197740043 | 4 | 3 | NA | FALSE | NA | 0 | NA | FALSE | FALSE |
| chr3 | 197741680 | 197741744 | 4 | 4 | NA | FALSE | NA | 0 | 1 | FALSE | FALSE |
| chr3 | 197843745 | 197843845 | 10 | 8 | NA | FALSE | NA | 0 | 1 | TRUE | FALSE |
| chr3 | 197851425 | 197851495 | 4 | 4 | NA | FALSE | NA | 0 | 1 | FALSE | FALSE |
| chr3 | 197857057 | 197857097 | 4 | 3 | AluSp | TRUE | NA | 0 | NA | FALSE | FALSE |
| chr4 | 18077 | 18131 | 4 | 3 | L1MC3 | TRUE | NA | 0 | 1 | FALSE | FALSE |
| chr4 | 27750 | 27823 | 4 | 4 | NA | FALSE | NA | 0 | 1 | FALSE | FALSE |
| chr4 | 29678 | 29732 | 6 | 5 | NA | FALSE | NA | 0 | 1 | FALSE | FALSE |
| chr4 | 36115 | 36206 | 5 | 4 | NA | FALSE | NA | 0 | 1 | TRUE | FALSE |
| chr4 | 38620 | 38667 | 4 | 3 | L1P2 | TRUE | NA | 0 | 1 | FALSE | FALSE |
| chr4 | 40697 | 40795 | 10 | 9 | LTR5B | TRUE | NA | 0 | 1 | FALSE | FALSE |
| chr4 | 41929 | 42017 | 5 | 4 | L1P2 | TRUE | NA | 0 | 1 | FALSE | FALSE |
| chr4 | 43244 | 43310 | 4 | 4 | MARNA | TRUE | NA | 0 | 1 | TRUE | FALSE |
| chr4 | 44071 | 44140 | 5 | 5 | MIRc | TRUE | NA | 0 | 1 | TRUE | FALSE |
| chr4 | 46022 | 46145 | 6 | 5 | AluSq2 | TRUE | NA | 0 | 1 | TRUE | FALSE |
| chr4 | 48052 | 48117 | 4 | 4 | NA | FALSE | ZZZ3 | 1 | 1 | TRUE | FALSE |
| chr4 | 48204 | 48318 | 7 | 6 | NA | FALSE | BRF1 | 1 | 1 | TRUE | FALSE |
| chr4 | 50169 | 50236 | 5 | 5 | AluY | TRUE | NA | 0 | 1 | TRUE | FALSE |
| chr4 | 50430 | 50459 | 4 | 4 | AluY | TRUE | NA | 0 | NA | TRUE | FALSE |
| chr4 | 50955 | 51003 | 4 | 4 | MER51C | TRUE | ZZZ3 | 1 | 1 | TRUE | FALSE |
| chr4 | 52346 | 52402 | 4 | 4 | NA | FALSE | NA | 0 | 1 | TRUE | FALSE |
| chr4 | 53200 | 53228 | 4 | 4 | NA | FALSE | MANY | 5 | 1 | TRUE | TRUE |
| chr4 | 54357 | 54417 | 6 | 6 | NA | FALSE | NA | 0 | 1 | TRUE | FALSE |
| chr4 | 57997 | 58077 | 5 | 4 | NA | FALSE | PU.1 | 1 | 1 | TRUE | FALSE |
| chr4 | 62604 | 62677 | 4 | 3 | L1MA9 | TRUE | NA | 0 | 1 | TRUE | FALSE |
| chr4 | 65639 | 65691 | 5 | 5 | MSTA | TRUE | NA | 0 | 1 | TRUE | FALSE |
| chr4 | 301145 | 301184 | 4 | 3 | MER51C | TRUE | NA | 0 | 1 | FALSE | FALSE |
| chr4 | 544206 | 544208 | 5 | 3 | SVA D | TRUE | NA | 0 | NA | FALSE | FALSE |
| chr4 | 584328 | 584402 | 5 | 5 | MLT1E | TRUE | NA | 0 | 1 | TRUE | FALSE |
| chr4 | 710062 | 710093 | 6 | 6 | NA | FALSE | NA | 0 | NA | FALSE | FALSE |
| chr4 | 768226 | 768249 | 4 | 2 | LTR12C | TRUE | HEY1 | 1 | NA | FALSE | FALSE |
| chr4 | 1141362 | 1141408 | 4 | 2 | NA | FALSE | NA | 0 | NA | FALSE | FALSE |
| chr4 | 1159134 | 1159134 | 4 | 4 | NA | FALSE | NA | 0 | NA | FALSE | FALSE |
| chr4 | 1283555 | 1283558 | 4 | 4 | NA | FALSE | MANY | 31 | 1 | TRUE | TRUE |
| chr4 | 1499088 | 1499105 | 4 | 2 | (TGGA)n | TRUE | NA | 0 | 1 | FALSE | FALSE |
| chr4 | 1672829 | 1672865 | 4 | 4 | (CCCCAG)n | TRUE | NA | 0 | 1 | FALSE | FALSE |
| chr4 | 1673027 | 1673077 | 4 | 3 | (CCCCAG)n | TRUE | NA | 0 | 1 | FALSE | FALSE |
| chr4 | 1689739 | 1689773 | 4 | 3 | AluY | TRUE | NA | 0 | NA | FALSE | FALSE |
| chr4 | 1829510 | 1829610 | 12 | 8 | SVA F | TRUE | NA | 0 | NA | FALSE | FALSE |
| chr4 | 2003781 | 2003812 | 5 | 4 | AluY | TRUE | NA | 0 | NA | FALSE | FALSE |
| chr4 | 2936582 | 2936630 | 7 | 6 | NA | FALSE | MANY | 32 | 1 | TRUE | TRUE |
| chr4 | 3294615 | 3294653 | 4 | 3 | (CCCCG)n | TRUE | MANY | 7 | 1 | TRUE | TRUE |
| chr4 | 3356201 | 3356259 | 4 | 3 | L1ME3B | TRUE | MANY | 18 | 1 | TRUE | TRUE |
| chr4 | 3549186 | 3549225 | 5 | 5 | MER74B | TRUE | NA | 0 | 1 | FALSE | FALSE |
| chr4 | 3990368 | 3990406 | 4 | 3 | NA | FALSE | NA | 0 | 1 | FALSE | FALSE |
| chr4 | 4038291 | 4038332 | 4 | 4 | HERVS71-int | TRUE | NA | 0 | NA | FALSE | FALSE |
| chr4 | 4062526 | 4062551 | 4 | 2 | L1MA4 | TRUE | NA | 0 | NA | FALSE | FALSE |
| chr4 | 4157130 | 4157196 | 5 | 4 | AluSp | TRUE | NA | 0 | NA | FALSE | FALSE |
| chr4 | 4185482 | 4185517 | 7 | 4 | NA | FALSE | NA | 0 | 1 | TRUE | FALSE |
| chr4 | 4216829 | 4216862 | 5 | 3 | AT rich | TRUE | NA | 0 | 1 | FALSE | FALSE |
| chr4 | 4217100 | 4217130 | 5 | 5 | AluSp | TRUE | NA | 0 | NA | FALSE | FALSE |
| chr4 | 4236064 | 4236088 | 6 | 4 | HSMAR2 | TRUE | NA | 0 | 1 | FALSE | FALSE |
| chr4 | 4846632 | 4846704 | 5 | 4 | MLT1C | TRUE | NA | 0 | 1 | TRUE | FALSE |
| chr4 | 5295856 | 5295928 | 5 | 3 | L1PA8 | TRUE | NA | 0 | NA | FALSE | FALSE |
| chr4 | 5495500 | 5495554 | 7 | 5 | L1PA3 | TRUE | NA | 0 | NA | FALSE | FALSE |
| chr4 | 6006131 | 6006146 | 4 | 4 | HAL1-2a MD | TRUE | NA | 0 | NA | FALSE | FALSE |
| chr4 | 6514109 | 6514145 | 6 | 4 | (TGG)n | TRUE | NA | 0 | NA | FALSE | FALSE |
| chr4 | 6576887 | 6576901 | 4 | 4 | GC rich | TRUE | MANY | 16 | 1 | TRUE | TRUE |
| chr4 | 7545699 | 7545722 | 4 | 4 | (TCCA)n | TRUE | NA | 0 | NA | FALSE | FALSE |
| chr4 | 8296286 | 8296316 | 4 | 3 | (TCCA)n | TRUE | NA | 0 | 1 | FALSE | FALSE |
| chr4 | 8847638 | 8847660 | 4 | 4 | G-rich | TRUE | NA | 0 | 1 | FALSE | FALSE |
| chr4 | 8903215 | 8903233 | 4 | 4 | NA | FALSE | MANY | 2 | 1 | TRUE | FALSE |
| chr4 | 8948171 | 8948171 | 4 | 4 | SATR1 | TRUE | NA | 0 | NA | FALSE | FALSE |
| chr4 | 9035601 | 9035629 | 4 | 4 | NA | FALSE | NA | 0 | 1 | FALSE | FALSE |
| chr4 | 9037161 | 9037180 | 4 | 3 | MANY | TRUE | NA | 0 | 1 | FALSE | FALSE |
| chr4 | 9082127 | 9082140 | 4 | 4 | L2b | TRUE | NA | 0 | 1 | FALSE | FALSE |
| chr4 | 9126371 | 9126372 | 4 | 2 | HERVK-int | TRUE | NA | 0 | NA | TRUE | FALSE |
| chr4 | 9129741 | 9129741 | 4 | 4 | HERVK-int | TRUE | NA | 0 | NA | FALSE | FALSE |
| chr4 | 9404146 | 9404150 | 5 | 5 | NA | FALSE | NA | 0 | 1 | FALSE | FALSE |
| chr4 | 9404196 | 9404234 | 9 | 8 | NA | FALSE | NA | 0 | 1 | FALSE | FALSE |
| chr4 | 9440273 | 9440298 | 5 | 4 | AluY | TRUE | NA | 0 | NA | FALSE | FALSE |
| chr4 | 9499433 | 9499439 | 4 | 2 | NA | FALSE | NA | 0 | NA | TRUE | FALSE |
| chr4 | 9504914 | 9504934 | 6 | 5 | SATR1 | TRUE | NA | 0 | NA | TRUE | FALSE |
| chr4 | 9539949 | 9539975 | 5 | 3 | NA | FALSE | NA | 0 | 1 | FALSE | FALSE |
| chr4 | 9548651 | 9548701 | 7 | 3 | NA | FALSE | NA | 0 | 1 | FALSE | FALSE |
| chr4 | 9570574 | 9570611 | 7 | 6 | HERVK-int | TRUE | NA | 0 | 1 | TRUE | FALSE |
| chr4 | 9634185 | 9634210 | 4 | 4 | MLT1E | TRUE | NA | 0 | 1 | FALSE | FALSE |
| chr4 | 9643377 | 9643408 | 4 | 3 | HERVH-int | TRUE | NA | 0 | 1 | FALSE | FALSE |
| chr4 | 9666566 | 9666610 | 5 | 4 | HERVK-int | TRUE | NA | 0 | 1 | FALSE | FALSE |
| chr4 | 9682061 | 9682066 | 7 | 4 | LTR12C | TRUE | NF-YA | 1 | 1 | FALSE | FALSE |
| chr4 | 9729702 | 9729703 | 4 | 2 | AluSx3 | TRUE | NA | 0 | NA | FALSE | FALSE |
| chr4 | 9735902 | 9735915 | 5 | 4 | AluY | TRUE | NA | 0 | NA | FALSE | FALSE |
| chr4 | 9786860 | 9786863 | 4 | 3 | MIR3 | TRUE | NA | 0 | 1 | FALSE | FALSE |
| chr4 | 10666814 | 10666858 | 4 | 4 | NA | FALSE | NA | 0 | 1 | FALSE | FALSE |
| chr4 | 11168170 | 11168181 | 4 | 4 | Charlie15a | TRUE | NA | 0 | 1 | FALSE | FALSE |
| chr4 | 11206366 | 11206389 | 4 | 4 | L1ME3 | TRUE | NA | 0 | 1 | FALSE | FALSE |
| chr4 | 11681026 | 11681038 | 4 | 3 | NA | FALSE | NA | 0 | 1 | FALSE | FALSE |
| chr4 | 12065610 | 12065625 | 4 | 3 | NA | FALSE | NA | 0 | 1 | FALSE | FALSE |
| chr4 | 12365647 | 12365648 | 4 | 3 | LTR7 | TRUE | NA | 0 | NA | TRUE | FALSE |
| chr4 | 12402667 | 12402671 | 4 | 3 | A-rich | TRUE | NA | 0 | 1 | FALSE | FALSE |
| chr4 | 13579823 | 13579895 | 4 | 3 | AluSx1 | TRUE | NA | 0 | 1 | FALSE | FALSE |
| chr4 | 14092384 | 14092460 | 4 | 3 | MLT1G1 | TRUE | NA | 0 | 1 | FALSE | FALSE |
| chr4 | 14474032 | 14474083 | 5 | 4 | L1PA4 | TRUE | NA | 0 | NA | FALSE | FALSE |
| chr4 | 15200682 | 15200755 | 4 | 3 | L1ME3A | TRUE | NA | 0 | 1 | FALSE | FALSE |
| chr4 | 15889543 | 15889596 | 4 | 2 | MANY | TRUE | NA | 0 | NA | FALSE | FALSE |
| chr4 | 17031078 | 17031096 | 4 | 4 | NA | FALSE | NA | 0 | 1 | FALSE | FALSE |
| chr4 | 17144007 | 17144032 | 4 | 4 | L2 | TRUE | MANY | 3 | 1 | TRUE | TRUE |
| chr4 | 17180820 | 17180840 | 4 | 3 | NA | FALSE | NA | 0 | 1 | FALSE | FALSE |
| chr4 | 18497277 | 18497284 | 4 | 2 | L1PA4 | TRUE | NA | 0 | NA | FALSE | FALSE |
| chr4 | 19240211 | 19240231 | 4 | 4 | NA | FALSE | NA | 0 | 1 | FALSE | FALSE |
| chr4 | 19428666 | 19428667 | 4 | 4 | L3 | TRUE | NA | 0 | 1 | FALSE | FALSE |
| chr4 | 19668784 | 19668809 | 4 | 3 | NA | FALSE | NA | 0 | 1 | FALSE | FALSE |
| chr4 | 19741589 | 19741609 | 4 | 3 | L2c | TRUE | NA | 0 | 1 | FALSE | FALSE |
| chr4 | 20098531 | 20098548 | 4 | 2 | NA | FALSE | NA | 0 | 1 | FALSE | FALSE |
| chr4 | 20208965 | 20208973 | 4 | 3 | NA | FALSE | NA | 0 | 1 | FALSE | FALSE |
| chr4 | 21063856 | 21063873 | 5 | 4 | L2b | TRUE | NA | 0 | 1 | TRUE | FALSE |
| chr4 | 21200689 | 21200689 | 4 | 4 | NA | FALSE | NA | 0 | NA | FALSE | FALSE |
| chr4 | 21309037 | 21309050 | 4 | 3 | L2a | TRUE | NA | 0 | 1 | FALSE | FALSE |
| chr4 | 21351421 | 21351440 | 4 | 3 | NA | FALSE | NA | 0 | 1 | FALSE | FALSE |
| chr4 | 22088773 | 22088803 | 4 | 3 | MANY | TRUE | NA | 0 | 1 | FALSE | FALSE |
| chr4 | 22759275 | 22759299 | 4 | 4 | NA | FALSE | NA | 0 | 1 | FALSE | FALSE |
| chr4 | 23575797 | 23575811 | 4 | 3 | MER61-int | TRUE | NA | 0 | 1 | FALSE | FALSE |
| chr4 | 25536654 | 25536694 | 4 | 2 | NA | FALSE | NA | 0 | 1 | FALSE | FALSE |
| chr4 | 25915722 | 25915777 | 7 | 7 | C-rich | TRUE | MANY | 13 | 1 | TRUE | TRUE |
| chr4 | 26149205 | 26149212 | 4 | 3 | MLT1N2 | TRUE | NA | 0 | 1 | FALSE | FALSE |
| chr4 | 26546360 | 26546413 | 5 | 3 | L1PA10 | TRUE | NA | 0 | 1 | FALSE | FALSE |
| chr4 | 27239044 | 27239075 | 4 | 3 | THE1B | TRUE | NA | 0 | 1 | FALSE | FALSE |
| chr4 | 27326319 | 27326332 | 5 | 4 | MER67C | TRUE | NA | 0 | 1 | FALSE | FALSE |
| chr4 | 27385660 | 27385668 | 4 | 4 | AluY | TRUE | NA | 0 | 1 | FALSE | FALSE |
| chr4 | 27394409 | 27394421 | 4 | 3 | L1ME3F | TRUE | NA | 0 | 1 | FALSE | FALSE |
| chr4 | 27412757 | 27412769 | 4 | 3 | NA | FALSE | NA | 0 | 1 | FALSE | FALSE |
| chr4 | 27752767 | 27752796 | 4 | 4 | (TTATA)n | TRUE | NA | 0 | 1 | TRUE | FALSE |
| chr4 | 28019488 | 28019511 | 4 | 3 | L2a | TRUE | NA | 0 | 1 | FALSE | FALSE |
| chr4 | 28871403 | 28871403 | 4 | 4 | NA | FALSE | NA | 0 | 1 | FALSE | FALSE |
| chr4 | 29870473 | 29870511 | 4 | 2 | L1P2 | TRUE | NA | 0 | 1 | FALSE | FALSE |
| chr4 | 29902951 | 29902981 | 4 | 3 | NA | FALSE | NA | 0 | 1 | FALSE | FALSE |
| chr4 | 30439750 | 30439801 | 4 | 3 | MIRc | TRUE | NA | 0 | 1 | FALSE | FALSE |
| chr4 | 32367916 | 32367938 | 4 | 3 | L1PA2 | TRUE | NA | 0 | NA | FALSE | FALSE |
| chr4 | 32999113 | 32999118 | 4 | 3 | L2 | TRUE | NA | 0 | 1 | FALSE | FALSE |
| chr4 | 33316791 | 33316798 | 5 | 5 | THE1B-int | TRUE | NA | 0 | 1 | FALSE | FALSE |
| chr4 | 33379903 | 33379933 | 4 | 3 | AluSq2 | TRUE | NA | 0 | NA | FALSE | FALSE |
| chr4 | 33705705 | 33705728 | 4 | 4 | L1MB1 | TRUE | NA | 0 | NA | FALSE | FALSE |
| chr4 | 33861658 | 33861678 | 4 | 2 | NA | FALSE | NA | 0 | 1 | FALSE | FALSE |
| chr4 | 34006448 | 34006463 | 5 | 3 | MamRep605 | TRUE | NA | 0 | 1 | FALSE | FALSE |
| chr4 | 34008033 | 34008040 | 4 | 3 | NA | FALSE | NA | 0 | NA | FALSE | FALSE |
| chr4 | 34161719 | 34161737 | 4 | 3 | L1MA2 | TRUE | NA | 0 | 1 | FALSE | FALSE |
| chr4 | 34432033 | 34432060 | 6 | 4 | NA | FALSE | NA | 0 | 1 | FALSE | FALSE |
| chr4 | 34879387 | 34879405 | 4 | 4 | L1PA8 | TRUE | NA | 0 | NA | FALSE | FALSE |
| chr4 | 35162068 | 35162087 | 4 | 4 | NA | FALSE | NA | 0 | 1 | FALSE | FALSE |
| chr4 | 35638053 | 35638078 | 4 | 4 | L1PREC2 | TRUE | NA | 0 | 1 | FALSE | FALSE |
| chr4 | 36442119 | 36442147 | 4 | 4 | NA | FALSE | NA | 0 | 1 | FALSE | FALSE |
| chr4 | 36468438 | 36468443 | 4 | 4 | NA | FALSE | NA | 0 | 1 | FALSE | FALSE |
| chr4 | 36853362 | 36853382 | 4 | 4 | NA | FALSE | NA | 0 | 1 | FALSE | FALSE |
| chr4 | 37670752 | 37670797 | 4 | 3 | SVA D | TRUE | NA | 0 | NA | FALSE | FALSE |
| chr4 | 37918956 | 37919003 | 5 | 3 | AluSx1 | TRUE | TCF4 | 1 | NA | FALSE | FALSE |
| chr4 | 39396428 | 39396527 | 4 | 3 | MLT1A0 | TRUE | NA | 0 | 1 | TRUE | FALSE |
| chr4 | 40149227 | 40149290 | 6 | 4 | SVA D | TRUE | NA | 0 | NA | FALSE | FALSE |
| chr4 | 40445483 | 40445532 | 4 | 4 | AluSp | TRUE | NA | 0 | NA | FALSE | FALSE |
| chr4 | 40584585 | 40584634 | 4 | 2 | L2c | TRUE | NA | 0 | 1 | TRUE | FALSE |
| chr4 | 40706625 | 40706677 | 4 | 3 | Charlie4a | TRUE | NA | 0 | 1 | TRUE | FALSE |
| chr4 | 41432611 | 41432633 | 4 | 4 | NA | FALSE | NA | 0 | 1 | FALSE | FALSE |
| chr4 | 41870889 | 41870905 | 4 | 4 | (TA)n | TRUE | NA | 0 | NA | FALSE | FALSE |
| chr4 | 42142228 | 42142246 | 4 | 2 | NA | FALSE | NA | 0 | 1 | TRUE | FALSE |
| chr4 | 42185033 | 42185041 | 4 | 4 | L1M5 | TRUE | NA | 0 | 1 | FALSE | FALSE |
| chr4 | 42199261 | 42199277 | 4 | 2 | NA | FALSE | NA | 0 | 1 | FALSE | FALSE |
| chr4 | 42269467 | 42269480 | 4 | 4 | MER4B | TRUE | NA | 0 | 1 | TRUE | FALSE |
| chr4 | 42793335 | 42793345 | 4 | 3 | L1PA5 | TRUE | NA | 0 | NA | FALSE | FALSE |
| chr4 | 43168666 | 43168666 | 4 | 4 | NA | FALSE | NA | 0 | 1 | FALSE | FALSE |
| chr4 | 43378627 | 43378638 | 4 | 3 | L1PA10 | TRUE | NA | 0 | NA | FALSE | FALSE |
| chr4 | 43384847 | 43384855 | 4 | 4 | NA | FALSE | NA | 0 | 1 | FALSE | FALSE |
| chr4 | 43429843 | 43429854 | 4 | 3 | L1PA7 | TRUE | NA | 0 | NA | FALSE | FALSE |
| chr4 | 43882508 | 43882524 | 4 | 3 | (GGAAA)n | TRUE | NA | 0 | NA | FALSE | FALSE |
| chr4 | 44103857 | 44103871 | 4 | 4 | L1PB | TRUE | NA | 0 | 1 | FALSE | FALSE |
| chr4 | 44115951 | 44115962 | 4 | 4 | L1MA3 | TRUE | NA | 0 | NA | FALSE | FALSE |
| chr4 | 44168239 | 44168253 | 4 | 3 | NA | FALSE | NA | 0 | 1 | TRUE | FALSE |
| chr4 | 44180641 | 44180655 | 4 | 3 | NA | FALSE | NA | 0 | 1 | FALSE | FALSE |
| chr4 | 44250317 | 44250330 | 4 | 3 | MER53 | TRUE | NA | 0 | 1 | FALSE | FALSE |
| chr4 | 44300670 | 44300688 | 4 | 3 | NA | FALSE | NA | 0 | 1 | FALSE | FALSE |
| chr4 | 44613427 | 44613444 | 4 | 4 | NA | FALSE | NA | 0 | 1 | FALSE | FALSE |
| chr4 | 44993846 | 44993864 | 4 | 3 | MER50-int | TRUE | NA | 0 | 1 | FALSE | FALSE |
| chr4 | 45191006 | 45191006 | 5 | 5 | (TCTA)n | TRUE | NA | 0 | 1 | FALSE | FALSE |
| chr4 | 45318587 | 45318601 | 4 | 4 | G-rich | TRUE | NA | 0 | 1 | TRUE | FALSE |
| chr4 | 45425349 | 45425359 | 4 | 4 | L1PB1 | TRUE | NA | 0 | 1 | FALSE | FALSE |
| chr4 | 45787285 | 45787293 | 4 | 3 | NA | FALSE | NA | 0 | 1 | FALSE | FALSE |
| chr4 | 45942869 | 45942882 | 4 | 4 | AluY | TRUE | NA | 0 | NA | FALSE | FALSE |
| chr4 | 46067724 | 46067724 | 4 | 4 | NA | FALSE | CEBPB | 1 | NA | TRUE | FALSE |
| chr4 | 46198430 | 46198445 | 4 | 3 | MER61A | TRUE | NA | 0 | 1 | FALSE | FALSE |
| chr4 | 46201226 | 46201232 | 4 | 4 | NA | FALSE | NA | 0 | 1 | FALSE | FALSE |
| chr4 | 46400315 | 46400326 | 4 | 4 | MLT1I | TRUE | NA | 0 | 1 | FALSE | FALSE |
| chr4 | 46524625 | 46524634 | 4 | 3 | HERVL-int | TRUE | NA | 0 | 1 | FALSE | FALSE |
| chr4 | 47742344 | 47742386 | 4 | 3 | L1M4 | TRUE | NA | 0 | 1 | FALSE | FALSE |
| chr4 | 47789093 | 47789134 | 4 | 3 | NA | FALSE | MANY | 12 | 1 | TRUE | TRUE |
| chr4 | 48186449 | 48186496 | 4 | 1 | NA | FALSE | NA | 0 | 1 | FALSE | FALSE |
| chr4 | 48294688 | 48294734 | 4 | 3 | SVA C | TRUE | NA | 0 | 1 | FALSE | FALSE |
| chr4 | 48343327 | 48343377 | 6 | 4 | NA | FALSE | MANY | 10 | 1 | TRUE | TRUE |
| chr4 | 48930932 | 48930932 | 4 | 4 | AluSx | TRUE | NA | 0 | NA | FALSE | FALSE |
| chr4 | 49017213 | 49017244 | 4 | 3 | L1MEg | TRUE | NA | 0 | 1 | FALSE | FALSE |
| chr4 | 49058901 | 49058907 | 4 | 4 | L1MA8 | TRUE | NA | 0 | NA | FALSE | FALSE |
| chr4 | 52924786 | 52924821 | 4 | 2 | L2b | TRUE | ERalpha a | 1 | 1 | FALSE | FALSE |
| chr4 | 53324619 | 53324661 | 5 | 5 | L1P2 | TRUE | NA | 0 | 1 | FALSE | FALSE |
| chr4 | 54103930 | 54103954 | 5 | 5 | SVA D | TRUE | NA | 0 | NA | FALSE | FALSE |
| chr4 | 54122913 | 54122992 | 7 | 4 | SVA D | TRUE | NA | 0 | 1 | FALSE | FALSE |
| chr4 | 54716455 | 54716485 | 4 | 4 | MANY | TRUE | CTCF | 1 | NA | TRUE | FALSE |
| chr4 | 54767401 | 54767415 | 4 | 2 | MIRb | TRUE | NA | 0 | 1 | FALSE | FALSE |
| chr4 | 55331483 | 55331504 | 9 | 4 | L1PA6 | TRUE | NA | 0 | NA | FALSE | FALSE |
| chr4 | 56057912 | 56057933 | 6 | 3 | L1PA8 | TRUE | NA | 0 | NA | FALSE | FALSE |
| chr4 | 56123636 | 56123654 | 5 | 2 | L1PA7 | TRUE | NA | 0 | NA | FALSE | FALSE |
| chr4 | 56783322 | 56783384 | 4 | 2 | MANY | TRUE | NA | 0 | 1 | FALSE | FALSE |
| chr4 | 57199134 | 57199187 | 4 | 3 | L1PA3 | TRUE | NA | 0 | NA | FALSE | FALSE |
| chr4 | 57560200 | 57560260 | 5 | 3 | SVA D | TRUE | NA | 0 | NA | FALSE | FALSE |
| chr4 | 57727037 | 57727037 | 5 | 5 | AluSx | TRUE | MANY | 4 | NA | TRUE | FALSE |
| chr4 | 58019877 | 58019907 | 4 | 2 | NA | FALSE | NA | 0 | 1 | FALSE | FALSE |
| chr4 | 58039160 | 58039194 | 4 | 4 | SVA D | TRUE | NA | 0 | NA | FALSE | FALSE |
| chr4 | 58557720 | 58557725 | 4 | 3 | NA | FALSE | MANY | 2 | 1 | TRUE | FALSE |
| chr4 | 59138750 | 59138762 | 4 | 4 | MER5B | TRUE | NA | 0 | 1 | FALSE | FALSE |
| chr4 | 59683241 | 59683243 | 5 | 5 | LTR33C | TRUE | NA | 0 | 1 | FALSE | FALSE |
| chr4 | 59723613 | 59723625 | 4 | 2 | L1PA7 | TRUE | NA | 0 | 1 | FALSE | FALSE |
| chr4 | 59840632 | 59840654 | 4 | 2 | AT rich | TRUE | NA | 0 | 1 | FALSE | FALSE |
| chr4 | 59870602 | 59870639 | 5 | 4 | L2 | TRUE | NA | 0 | 1 | FALSE | FALSE |
| chr4 | 60026445 | 60026454 | 4 | 2 | L1PA16 | TRUE | NA | 0 | 1 | FALSE | FALSE |
| chr4 | 60296144 | 60296166 | 5 | 4 | L1PA5 | TRUE | NA | 0 | 1 | FALSE | FALSE |
| chr4 | 60839627 | 60839629 | 4 | 3 | NA | FALSE | NA | 0 | 1 | FALSE | FALSE |
| chr4 | 61285154 | 61285182 | 4 | 4 | L1PA7 | TRUE | NA | 0 | 1 | FALSE | FALSE |
| chr4 | 61464846 | 61464855 | 4 | 4 | L1PA11 | TRUE | NA | 0 | NA | FALSE | FALSE |
| chr4 | 61493090 | 61493116 | 4 | 4 | L1PA7 | TRUE | NA | 0 | NA | FALSE | FALSE |
| chr4 | 61729960 | 61729978 | 4 | 3 | NA | FALSE | NA | 0 | 1 | TRUE | FALSE |
| chr4 | 63198206 | 63198236 | 4 | 3 | L1PB1 | TRUE | NA | 0 | NA | FALSE | FALSE |
| chr4 | 63405747 | 63405784 | 6 | 5 | L1MC1 | TRUE | NA | 0 | 1 | FALSE | FALSE |
| chr4 | 64429395 | 64429408 | 4 | 3 | MER52A | TRUE | CTCF | 1 | 1 | TRUE | FALSE |
| chr4 | 65541899 | 65541924 | 4 | 4 | HERVL-int | TRUE | NA | 0 | 1 | FALSE | FALSE |
| chr4 | 66923994 | 66924015 | 4 | 2 | MLT1A | TRUE | NA | 0 | 1 | FALSE | FALSE |
| chr4 | 67601194 | 67601225 | 6 | 6 | MLT2D | TRUE | NA | 0 | 1 | TRUE | FALSE |
| chr4 | 67636931 | 67636931 | 4 | 4 | L1PREC2 | TRUE | NA | 0 | 1 | FALSE | FALSE |
| chr4 | 68121010 | 68121036 | 5 | 5 | L1PA10 | TRUE | NA | 0 | 1 | FALSE | FALSE |
| chr4 | 68142069 | 68142107 | 4 | 3 | HERV9-int | TRUE | NA | 0 | 1 | FALSE | FALSE |
| chr4 | 68229063 | 68229079 | 4 | 3 | L1M4b | TRUE | NA | 0 | 1 | FALSE | FALSE |
| chr4 | 68502909 | 68502912 | 4 | 2 | SVA C | TRUE | NA | 0 | NA | FALSE | FALSE |
| chr4 | 69154332 | 69154356 | 4 | 4 | L1M4b | TRUE | NA | 0 | 1 | TRUE | FALSE |
| chr4 | 69424165 | 69424184 | 4 | 4 | MER4-int | TRUE | NA | 0 | NA | FALSE | FALSE |
| chr4 | 69504093 | 69504095 | 4 | 3 | NA | FALSE | NA | 0 | 1 | FALSE | FALSE |
| chr4 | 69793481 | 69793493 | 4 | 4 | NA | FALSE | NA | 0 | 1 | FALSE | FALSE |
| chr4 | 70172118 | 70172134 | 4 | 3 | AT rich | TRUE | NA | 0 | 1 | FALSE | FALSE |
| chr4 | 70254072 | 70254080 | 4 | 4 | NA | FALSE | NA | 0 | 1 | FALSE | FALSE |
| chr4 | 71239654 | 71239674 | 4 | 4 | L1PB1 | TRUE | NA | 0 | NA | FALSE | FALSE |
| chr4 | 71275417 | 71275445 | 4 | 3 | NA | FALSE | NA | 0 | 1 | FALSE | FALSE |
| chr4 | 71284940 | 71284984 | 5 | 2 | L1PA4 | TRUE | NA | 0 | NA | FALSE | FALSE |
| chr4 | 72221545 | 72221589 | 4 | 4 | NA | FALSE | NA | 0 | 1 | FALSE | FALSE |
| chr4 | 72726573 | 72726614 | 4 | 4 | MER4B-int | TRUE | MANY | 2 | 1 | TRUE | FALSE |
| chr4 | 72812911 | 72812913 | 5 | 3 | (TG)n | TRUE | NA | 0 | NA | FALSE | FALSE |
| chr4 | 73652646 | 73652702 | 4 | 3 | NA | FALSE | NA | 0 | 1 | FALSE | FALSE |
| chr4 | 73812319 | 73812361 | 4 | 4 | NA | FALSE | NA | 0 | 1 | FALSE | FALSE |
| chr4 | 74354994 | 74355032 | 4 | 3 | T-rich | TRUE | NA | 0 | 1 | FALSE | FALSE |
| chr4 | 75358329 | 75358345 | 4 | 4 | NA | FALSE | NA | 0 | 1 | FALSE | FALSE |
| chr4 | 75662657 | 75662684 | 4 | 4 | NA | FALSE | NA | 0 | 1 | FALSE | FALSE |
| chr4 | 76344411 | 76344424 | 4 | 4 | L1PA7 | TRUE | NA | 0 | NA | FALSE | FALSE |
| chr4 | 76972131 | 76972220 | 4 | 4 | LTR12C | TRUE | NA | 0 | 1 | FALSE | FALSE |
| chr4 | 77578333 | 77578342 | 6 | 6 | (TC)n | TRUE | NA | 0 | NA | FALSE | FALSE |
| chr4 | 77581830 | 77581869 | 10 | 4 | AluSx1 | TRUE | NA | 0 | NA | FALSE | FALSE |
| chr4 | 78262643 | 78262643 | 4 | 4 | L1PA4 | TRUE | NA | 0 | NA | FALSE | FALSE |
| chr4 | 78420267 | 78420282 | 4 | 4 | NA | FALSE | NA | 0 | 1 | FALSE | FALSE |
| chr4 | 79008584 | 79008640 | 5 | 2 | L1PA4 | TRUE | NA | 0 | NA | FALSE | FALSE |
| chr4 | 79924672 | 79924677 | 4 | 2 | L1PA10 | TRUE | NA | 0 | NA | FALSE | FALSE |
| chr4 | 81295150 | 81295162 | 4 | 4 | ERV3-16A3 I-int | TRUE | NA | 0 | 1 | FALSE | FALSE |
| chr4 | 81331916 | 81331917 | 4 | 3 | MIRb | TRUE | NA | 0 | 1 | FALSE | FALSE |
| chr4 | 81338746 | 81338762 | 4 | 4 | NA | FALSE | NA | 0 | 1 | FALSE | FALSE |
| chr4 | 81385005 | 81385006 | 4 | 4 | NA | FALSE | NA | 0 | 1 | FALSE | FALSE |
| chr4 | 81432678 | 81432688 | 4 | 3 | NA | FALSE | NA | 0 | 1 | FALSE | FALSE |
| chr4 | 81582819 | 81582824 | 4 | 4 | MER11C | TRUE | NA | 0 | NA | FALSE | FALSE |
| chr4 | 81864331 | 81864352 | 5 | 5 | NA | FALSE | NA | 0 | 1 | FALSE | FALSE |
| chr4 | 81961756 | 81961778 | 4 | 1 | L3 | TRUE | NA | 0 | 1 | TRUE | FALSE |
| chr4 | 82218452 | 82218460 | 4 | 4 | THE1D-int | TRUE | NA | 0 | NA | FALSE | FALSE |
| chr4 | 84095607 | 84095672 | 4 | 4 | NA | FALSE | NA | 0 | 1 | FALSE | FALSE |
| chr4 | 84544338 | 84544411 | 7 | 5 | THE1C-int | TRUE | NA | 0 | 1 | FALSE | FALSE |
| chr4 | 85015244 | 85015254 | 4 | 3 | NA | FALSE | NA | 0 | 1 | FALSE | FALSE |
| chr4 | 85059722 | 85059757 | 4 | 2 | L1MD3 | TRUE | NA | 0 | 1 | TRUE | FALSE |
| chr4 | 85395035 | 85395075 | 4 | 4 | NA | FALSE | NA | 0 | 1 | FALSE | FALSE |
| chr4 | 86108510 | 86108516 | 4 | 2 | L1M1 | TRUE | NA | 0 | 1 | FALSE | FALSE |
| chr4 | 86246043 | 86246071 | 5 | 4 | L1PA4 | TRUE | NA | 0 | NA | FALSE | FALSE |
| chr4 | 86706101 | 86706105 | 5 | 4 | (TCTA)n | TRUE | NA | 0 | NA | FALSE | FALSE |
| chr4 | 87194481 | 87194545 | 4 | 3 | NA | FALSE | NA | 0 | 1 | FALSE | FALSE |
| chr4 | 91694337 | 91694366 | 4 | 2 | AluSp | TRUE | NA | 0 | 1 | FALSE | FALSE |
| chr4 | 93661874 | 93661888 | 4 | 3 | (TAGA)n | TRUE | NA | 0 | NA | FALSE | FALSE |
| chr4 | 94101163 | 94101184 | 4 | 4 | NA | FALSE | NA | 0 | 1 | FALSE | FALSE |
| chr4 | 94400787 | 94400812 | 4 | 3 | MLT2B3 | TRUE | NA | 0 | 1 | FALSE | FALSE |
| chr4 | 94803401 | 94803419 | 4 | 3 | NA | FALSE | NA | 0 | 1 | FALSE | FALSE |
| chr4 | 94853802 | 94853823 | 4 | 3 | L1PA7 | TRUE | NA | 0 | 1 | FALSE | FALSE |
| chr4 | 94972533 | 94972563 | 5 | 4 | L1MB2 | TRUE | NA | 0 | 1 | FALSE | FALSE |
| chr4 | 97352713 | 97352731 | 4 | 4 | NA | FALSE | NA | 0 | 1 | FALSE | FALSE |
| chr4 | 97940846 | 97940872 | 4 | 2 | NA | FALSE | NA | 0 | 1 | FALSE | FALSE |
| chr4 | 98101475 | 98101501 | 4 | 3 | MLT1J2 | TRUE | NA | 0 | 1 | FALSE | FALSE |
| chr4 | 98145720 | 98145735 | 4 | 4 | NA | FALSE | NA | 0 | 1 | FALSE | FALSE |
| chr4 | 98680029 | 98680050 | 5 | 5 | NA | FALSE | NA | 0 | 1 | FALSE | FALSE |
| chr4 | 98913751 | 98913770 | 4 | 4 | L1PB1 | TRUE | NA | 0 | 1 | FALSE | FALSE |
| chr4 | 99754854 | 99754887 | 4 | 3 | NA | FALSE | NA | 0 | 1 | TRUE | FALSE |
| chr4 | 100185042 | 100185077 | 5 | 4 | L1PA6 | TRUE | NA | 0 | NA | TRUE | FALSE |
| chr4 | 100371052 | 100371075 | 4 | 3 | L1PA14 | TRUE | NA | 0 | NA | FALSE | FALSE |
| chr4 | 101218786 | 101218811 | 4 | 4 | L2c | TRUE | NA | 0 | 1 | FALSE | FALSE |
| chr4 | 101221381 | 101221400 | 4 | 4 | NA | FALSE | NA | 0 | 1 | FALSE | FALSE |
| chr4 | 101470157 | 101470177 | 4 | 3 | MSTB2 | TRUE | NA | 0 | 1 | FALSE | FALSE |
| chr4 | 101799070 | 101799087 | 4 | 4 | L2c | TRUE | NA | 0 | 1 | FALSE | FALSE |
| chr4 | 102433702 | 102433718 | 4 | 4 | L2a | TRUE | NA | 0 | 1 | TRUE | FALSE |
| chr4 | 102556242 | 102556253 | 5 | 3 | HERV9-int | TRUE | NA | 0 | 1 | FALSE | FALSE |
| chr4 | 102966616 | 102966636 | 4 | 4 | L1PA3 | TRUE | NA | 0 | 1 | FALSE | FALSE |
| chr4 | 103159613 | 103159639 | 4 | 4 | HERV9-int | TRUE | NA | 0 | NA | FALSE | FALSE |
| chr4 | 103297560 | 103297627 | 4 | 4 | L1PA4 | TRUE | NA | 0 | NA | FALSE | FALSE |
| chr4 | 103829458 | 103829499 | 4 | 4 | NA | FALSE | NA | 0 | 1 | FALSE | FALSE |
| chr4 | 103830340 | 103830340 | 4 | 4 | FLAM C | TRUE | NA | 0 | NA | FALSE | FALSE |
| chr4 | 103831098 | 103831143 | 5 | 2 | AluSq | TRUE | NA | 0 | 1 | FALSE | FALSE |
| chr4 | 103838787 | 103838851 | 4 | 2 | L1M4c | TRUE | NA | 0 | 1 | FALSE | FALSE |
| chr4 | 103849809 | 103849840 | 9 | 6 | MLT2A2 | TRUE | NA | 0 | NA | FALSE | FALSE |
| chr4 | 103862348 | 103862381 | 4 | 2 | L1PA15 | TRUE | NA | 0 | 1 | FALSE | FALSE |
| chr4 | 103864864 | 103864882 | 5 | 4 | L1PB1 | TRUE | NA | 0 | NA | FALSE | FALSE |
| chr4 | 104436913 | 104436934 | 4 | 3 | NA | FALSE | NA | 0 | 1 | FALSE | FALSE |
| chr4 | 105111468 | 105111481 | 4 | 4 | AluSx | TRUE | NA | 0 | NA | FALSE | FALSE |
| chr4 | 105429276 | 105429295 | 4 | 3 | L2a | TRUE | NA | 0 | 1 | FALSE | FALSE |
| chr4 | 105461608 | 105461626 | 8 | 5 | L1PA7 | TRUE | NA | 0 | NA | FALSE | FALSE |
| chr4 | 105637369 | 105637388 | 4 | 4 | NA | FALSE | NA | 0 | 1 | FALSE | FALSE |
| chr4 | 105704464 | 105704474 | 4 | 3 | NA | FALSE | STAT3 | 1 | 1 | TRUE | FALSE |
| chr4 | 105999089 | 105999113 | 4 | 4 | MSTD | TRUE | NA | 0 | 1 | FALSE | FALSE |
| chr4 | 106892600 | 106892650 | 4 | 4 | NA | FALSE | NA | 0 | 1 | FALSE | FALSE |
| chr4 | 107260401 | 107260425 | 7 | 4 | THE1B-int | TRUE | NA | 0 | NA | FALSE | FALSE |
| chr4 | 107903318 | 107903327 | 4 | 4 | L2a | TRUE | NA | 0 | 1 | FALSE | FALSE |
| chr4 | 108037709 | 108037727 | 4 | 4 | THE1B | TRUE | NA | 0 | 1 | FALSE | FALSE |
| chr4 | 108724190 | 108724194 | 4 | 4 | (TC)n | TRUE | NA | 0 | NA | FALSE | FALSE |
| chr4 | 109541655 | 109541734 | 11 | 7 | NA | FALSE | MANY | 45 | 1 | TRUE | TRUE |
| chr4 | 111141843 | 111141884 | 4 | 3 | LTR1B | TRUE | NA | 0 | 1 | FALSE | FALSE |
| chr4 | 111370305 | 111370307 | 4 | 2 | L1MC2 | TRUE | NA | 0 | 1 | FALSE | FALSE |
| chr4 | 111387033 | 111387056 | 4 | 4 | NA | FALSE | NA | 0 | 1 | FALSE | FALSE |
| chr4 | 112106480 | 112106489 | 4 | 2 | NA | FALSE | NA | 0 | 1 | FALSE | FALSE |
| chr4 | 112874673 | 112874675 | 4 | 2 | NA | FALSE | NA | 0 | 1 | FALSE | FALSE |
| chr4 | 113035199 | 113035211 | 5 | 4 | NA | FALSE | NA | 0 | 1 | FALSE | FALSE |
| chr4 | 113471152 | 113471152 | 4 | 4 | AluY | TRUE | NA | 0 | NA | FALSE | FALSE |
| chr4 | 113486302 | 113486335 | 5 | 3 | NA | FALSE | MANY | 2 | 1 | TRUE | FALSE |
| chr4 | 113652207 | 113652265 | 4 | 3 | AluSc | TRUE | NA | 0 | 1 | FALSE | FALSE |
| chr4 | 113801185 | 113801231 | 4 | 3 | (CA)n | TRUE | NA | 0 | 1 | TRUE | FALSE |
| chr4 | 113864904 | 113864990 | 4 | 1 | NA | FALSE | NA | 0 | 1 | FALSE | FALSE |
| chr4 | 114330242 | 114330292 | 4 | 2 | AluSx1 | TRUE | NA | 0 | NA | FALSE | FALSE |
| chr4 | 114797763 | 114797823 | 4 | 2 | AluSq2 | TRUE | NA | 0 | 1 | FALSE | FALSE |
| chr4 | 114968194 | 114968275 | 9 | 5 | HERV17-int | TRUE | NA | 0 | 1 | FALSE | FALSE |
| chr4 | 115095826 | 115095897 | 6 | 4 | MANY | TRUE | NA | 0 | NA | FALSE | FALSE |
| chr4 | 115603945 | 115603968 | 5 | 4 | AluY | TRUE | NA | 0 | NA | FALSE | FALSE |
| chr4 | 115696014 | 115696061 | 7 | 7 | (TAGA)n | TRUE | NA | 0 | NA | FALSE | FALSE |
| chr4 | 116951901 | 116951907 | 4 | 4 | MLT1F-int | TRUE | NA | 0 | 1 | FALSE | FALSE |
| chr4 | 117100353 | 117100362 | 4 | 3 | AluJo | TRUE | NA | 0 | 1 | FALSE | FALSE |
| chr4 | 117167941 | 117167948 | 5 | 4 | NA | FALSE | NA | 0 | 1 | FALSE | FALSE |
| chr4 | 117464386 | 117464387 | 4 | 2 | NA | FALSE | NA | 0 | 1 | FALSE | FALSE |
| chr4 | 117486484 | 117486505 | 4 | 4 | L1PA11 | TRUE | NA | 0 | 1 | FALSE | FALSE |
| chr4 | 117771081 | 117771097 | 4 | 4 | L1MA8 | TRUE | CTCF | 1 | 1 | TRUE | FALSE |
| chr4 | 117825080 | 117825099 | 4 | 2 | NA | FALSE | NA | 0 | 1 | FALSE | FALSE |
| chr4 | 119308544 | 119308575 | 4 | 4 | NA | FALSE | NA | 0 | 1 | FALSE | FALSE |
| chr4 | 119368091 | 119368141 | 7 | 4 | MIR | TRUE | NA | 0 | 1 | FALSE | FALSE |
| chr4 | 119368332 | 119368344 | 4 | 3 | AluJb | TRUE | NA | 0 | NA | FALSE | FALSE |
| chr4 | 119380041 | 119380089 | 4 | 4 | NA | FALSE | NA | 0 | 1 | FALSE | FALSE |
| chr4 | 119387810 | 119387867 | 7 | 7 | (TC)n | TRUE | NA | 0 | NA | TRUE | FALSE |
| chr4 | 119443408 | 119443434 | 4 | 3 | L2a | TRUE | NA | 0 | 1 | FALSE | FALSE |
| chr4 | 119499369 | 119499372 | 5 | 4 | AluSx | TRUE | NA | 0 | NA | FALSE | FALSE |
| chr4 | 119811889 | 119811891 | 5 | 4 | MANY | TRUE | ZZZ3 | 1 | NA | TRUE | FALSE |
| chr4 | 120195245 | 120195273 | 6 | 5 | MANY | TRUE | NA | 0 | NA | FALSE | FALSE |
| chr4 | 120328092 | 120328111 | 4 | 3 | NA | FALSE | NA | 0 | NA | FALSE | FALSE |
| chr4 | 120367864 | 120367882 | 5 | 4 | NA | FALSE | NA | 0 | 1 | FALSE | FALSE |
| chr4 | 120625165 | 120625177 | 4 | 4 | L1MEf | TRUE | NA | 0 | 1 | FALSE | FALSE |
| chr4 | 120642151 | 120642178 | 4 | 2 | L1PB1 | TRUE | NA | 0 | NA | FALSE | FALSE |
| chr4 | 122360252 | 122360284 | 4 | 4 | L1MA3 | TRUE | NA | 0 | 1 | FALSE | FALSE |
| chr4 | 123471685 | 123471719 | 4 | 2 | L1PA17 | TRUE | NA | 0 | 1 | FALSE | FALSE |
| chr4 | 125216507 | 125216562 | 4 | 3 | MLT1A0-int | TRUE | NA | 0 | 1 | FALSE | FALSE |
| chr4 | 125340232 | 125340293 | 4 | 2 | L1M5 | TRUE | NA | 0 | 1 | FALSE | FALSE |
| chr4 | 126524565 | 126524573 | 4 | 3 | HAL1-3A ME | TRUE | NA | 0 | 1 | FALSE | FALSE |
| chr4 | 127377811 | 127377830 | 4 | 2 | NA | FALSE | NA | 0 | 1 | FALSE | FALSE |
| chr4 | 127515906 | 127515935 | 4 | 3 | NA | FALSE | NA | 0 | 1 | FALSE | FALSE |
| chr4 | 127734273 | 127734287 | 4 | 2 | L1PA7 | TRUE | NA | 0 | NA | FALSE | FALSE |
| chr4 | 127839985 | 127840013 | 4 | 3 | NA | FALSE | NA | 0 | 1 | FALSE | FALSE |
| chr4 | 128394064 | 128394093 | 4 | 4 | L1PA4 | TRUE | NA | 0 | NA | FALSE | FALSE |
| chr4 | 130580185 | 130580235 | 5 | 5 | THE1B-int | TRUE | NA | 0 | 1 | FALSE | FALSE |
| chr4 | 130730044 | 130730070 | 5 | 2 | L1PB1 | TRUE | NA | 0 | NA | FALSE | FALSE |
| chr4 | 131283194 | 131283214 | 4 | 4 | UCON28a | TRUE | NA | 0 | 1 | FALSE | FALSE |
| chr4 | 131297160 | 131297171 | 4 | 4 | MER31-int | TRUE | NA | 0 | 1 | FALSE | FALSE |
| chr4 | 131769259 | 131769272 | 4 | 3 | HERV9-int | TRUE | NA | 0 | NA | FALSE | FALSE |
| chr4 | 132582264 | 132582303 | 5 | 5 | L3 | TRUE | NA | 0 | 1 | FALSE | FALSE |
| chr4 | 132594858 | 132594875 | 4 | 3 | NA | FALSE | NA | 0 | 1 | FALSE | FALSE |
| chr4 | 132609273 | 132609298 | 4 | 3 | Charlie2a | TRUE | CEBPB | 1 | 1 | TRUE | FALSE |
| chr4 | 132622040 | 132622060 | 7 | 5 | MANY | TRUE | NA | 0 | NA | FALSE | FALSE |
| chr4 | 132627541 | 132627550 | 4 | 3 | Tigger1 | TRUE | NA | 0 | 1 | TRUE | FALSE |
| chr4 | 132670576 | 132670578 | 4 | 3 | SST1 | TRUE | NA | 0 | 1 | FALSE | FALSE |
| chr4 | 132700928 | 132700960 | 6 | 5 | NA | FALSE | NA | 0 | 1 | TRUE | FALSE |
| chr4 | 132710704 | 132710720 | 5 | 3 | L1MD | TRUE | NA | 0 | 1 | FALSE | FALSE |
| chr4 | 132722207 | 132722238 | 4 | 4 | NA | FALSE | NA | 0 | 1 | FALSE | FALSE |
| chr4 | 132725878 | 132725883 | 4 | 2 | LTR25-int | TRUE | NA | 0 | 1 | FALSE | FALSE |
| chr4 | 132735805 | 132735845 | 6 | 4 | L1PB3 | TRUE | NA | 0 | 1 | FALSE | FALSE |
| chr4 | 132753539 | 132753573 | 5 | 3 | LTR1D | TRUE | NA | 0 | 1 | FALSE | FALSE |
| chr4 | 132782745 | 132782745 | 4 | 4 | NA | FALSE | NA | 0 | NA | FALSE | FALSE |
| chr4 | 132782878 | 132782887 | 6 | 5 | NA | FALSE | NA | 0 | 1 | FALSE | FALSE |
| chr4 | 132813914 | 132813951 | 6 | 2 | NA | FALSE | NA | 0 | NA | FALSE | FALSE |
| chr4 | 132878853 | 132878853 | 4 | 4 | THE1B-int | TRUE | NA | 0 | NA | FALSE | FALSE |
| chr4 | 132894265 | 132894280 | 7 | 4 | L1ME3B | TRUE | NA | 0 | 1 | FALSE | FALSE |
| chr4 | 133002567 | 133002595 | 4 | 3 | L1MC5 | TRUE | NA | 0 | 1 | FALSE | FALSE |
| chr4 | 133113669 | 133113701 | 4 | 2 | NA | FALSE | NA | 0 | 1 | FALSE | FALSE |
| chr4 | 133825046 | 133825077 | 4 | 2 | NA | FALSE | MafK (ab50322) | 1 | 1 | FALSE | FALSE |
| chr4 | 134662069 | 134662097 | 4 | 4 | NA | FALSE | NA | 0 | 1 | FALSE | FALSE |
| chr4 | 136126664 | 136126674 | 4 | 4 | L1M5 | TRUE | NA | 0 | 1 | FALSE | FALSE |
| chr4 | 136529226 | 136529234 | 4 | 3 | L1ME4a | TRUE | NA | 0 | 1 | FALSE | FALSE |
| chr4 | 136768666 | 136768684 | 5 | 3 | L1PA7 | TRUE | NA | 0 | NA | FALSE | FALSE |
| chr4 | 136903589 | 136903589 | 4 | 4 | NA | FALSE | NA | 0 | 1 | FALSE | FALSE |
| chr4 | 138440296 | 138440303 | 4 | 3 | NA | FALSE | NA | 0 | 1 | TRUE | FALSE |
| chr4 | 138473853 | 138473853 | 4 | 4 | NA | FALSE | NA | 0 | 1 | FALSE | FALSE |
| chr4 | 138492382 | 138492402 | 4 | 4 | L1PA8A | TRUE | NA | 0 | 1 | FALSE | FALSE |
| chr4 | 138706291 | 138706317 | 4 | 3 | L1PA7 | TRUE | NA | 0 | 1 | FALSE | FALSE |
| chr4 | 138760965 | 138760983 | 4 | 4 | L1MA5 | TRUE | NA | 0 | 1 | FALSE | FALSE |
| chr4 | 138836674 | 138836687 | 4 | 3 | L1PA5 | TRUE | NA | 0 | NA | FALSE | FALSE |
| chr4 | 139572202 | 139572238 | 4 | 4 | MER21B | TRUE | MANY | 4 | 1 | TRUE | TRUE |
| chr4 | 139618356 | 139618376 | 4 | 3 | NA | FALSE | NA | 0 | 1 | TRUE | FALSE |
| chr4 | 140956893 | 140956913 | 6 | 3 | AluSc8 | TRUE | NA | 0 | NA | FALSE | FALSE |
| chr4 | 141658618 | 141658631 | 4 | 3 | MANY | TRUE | NA | 0 | NA | FALSE | FALSE |
| chr4 | 142504841 | 142504873 | 8 | 3 | L1PBa | TRUE | NA | 0 | NA | FALSE | FALSE |
| chr4 | 142871148 | 142871170 | 4 | 4 | MSTB | TRUE | NA | 0 | 1 | FALSE | FALSE |
| chr4 | 144554268 | 144554296 | 5 | 5 | MLT1N2 | TRUE | NA | 0 | 1 | FALSE | FALSE |
| chr4 | 144710688 | 144710714 | 4 | 4 | NA | FALSE | NA | 0 | 1 | FALSE | FALSE |
| chr4 | 144749298 | 144749308 | 4 | 4 | NA | FALSE | NA | 0 | 1 | FALSE | FALSE |
| chr4 | 144764205 | 144764213 | 5 | 2 | NA | FALSE | CEBPB | 1 | 1 | TRUE | FALSE |
| chr4 | 144765655 | 144765656 | 4 | 2 | MLT1H | TRUE | NA | 0 | 1 | FALSE | FALSE |
| chr4 | 144793374 | 144793383 | 4 | 2 | LTR78B | TRUE | NA | 0 | 1 | FALSE | FALSE |
| chr4 | 144824533 | 144824547 | 5 | 4 | NA | FALSE | NA | 0 | 1 | FALSE | FALSE |
| chr4 | 144851225 | 144851225 | 4 | 4 | MSTA-int | TRUE | NA | 0 | NA | TRUE | FALSE |
| chr4 | 144978867 | 144978890 | 7 | 4 | NA | FALSE | NA | 0 | 1 | FALSE | FALSE |
| chr4 | 145046759 | 145046778 | 6 | 5 | NA | FALSE | GATA-1 | 1 | 1 | FALSE | FALSE |
| chr4 | 145059009 | 145059009 | 4 | 4 | L2a | TRUE | NA | 0 | NA | FALSE | FALSE |
| chr4 | 145513054 | 145513078 | 4 | 3 | MIR3 | TRUE | GATA-2 | 1 | 1 | TRUE | FALSE |
| chr4 | 145726964 | 145726966 | 4 | 3 | NA | FALSE | NA | 0 | 1 | FALSE | FALSE |
| chr4 | 146323449 | 146323489 | 4 | 3 | L1PA6 | TRUE | NA | 0 | NA | FALSE | FALSE |
| chr4 | 146338728 | 146338781 | 4 | 4 | A-rich | TRUE | NA | 0 | 1 | FALSE | FALSE |
| chr4 | 146382537 | 146382540 | 4 | 2 | L1PA5 | TRUE | NA | 0 | NA | FALSE | FALSE |
| chr4 | 146518363 | 146518390 | 5 | 4 | L1PA5 | TRUE | NA | 0 | 1 | FALSE | FALSE |
| chr4 | 146799753 | 146799786 | 5 | 3 | A-rich | TRUE | NA | 0 | NA | TRUE | FALSE |
| chr4 | 147413140 | 147413158 | 4 | 3 | AluY | TRUE | NA | 0 | NA | FALSE | FALSE |
| chr4 | 147533614 | 147533635 | 4 | 3 | L1PREC2 | TRUE | NA | 0 | 1 | FALSE | FALSE |
| chr4 | 147573383 | 147573397 | 4 | 3 | NA | FALSE | NA | 0 | 1 | TRUE | FALSE |
| chr4 | 147715697 | 147715698 | 4 | 3 | NA | FALSE | NA | 0 | NA | FALSE | FALSE |
| chr4 | 148171427 | 148171429 | 4 | 4 | L1M4 | TRUE | NA | 0 | 1 | FALSE | FALSE |
| chr4 | 148326963 | 148326995 | 5 | 5 | L1PA8A | TRUE | NA | 0 | 1 | FALSE | FALSE |
| chr4 | 148622316 | 148622335 | 4 | 4 | L1M2c | TRUE | NA | 0 | 1 | FALSE | FALSE |
| chr4 | 148840726 | 148840727 | 6 | 3 | AluSz | TRUE | NA | 0 | NA | FALSE | FALSE |
| chr4 | 149429328 | 149429342 | 4 | 2 | NA | FALSE | BCL3 | 1 | NA | FALSE | FALSE |
| chr4 | 149624098 | 149624142 | 4 | 4 | L1MEg | TRUE | NA | 0 | 1 | FALSE | FALSE |
| chr4 | 149666039 | 149666051 | 4 | 4 | L1PA3 | TRUE | NA | 0 | NA | FALSE | FALSE |
| chr4 | 150249741 | 150249771 | 4 | 3 | NA | FALSE | NA | 0 | 1 | FALSE | FALSE |
| chr4 | 150861152 | 150861199 | 5 | 2 | L1PA13 | TRUE | NA | 0 | 1 | FALSE | FALSE |
| chr4 | 151613178 | 151613208 | 4 | 3 | L1ME2 | TRUE | NA | 0 | 1 | TRUE | FALSE |
| chr4 | 152762027 | 152762077 | 4 | 3 | NA | FALSE | NA | 0 | 1 | FALSE | FALSE |
| chr4 | 153044740 | 153044805 | 4 | 4 | L1PB1 | TRUE | NA | 0 | 1 | FALSE | FALSE |
| chr4 | 154018835 | 154018842 | 4 | 3 | L1ME3A | TRUE | NA | 0 | 1 | FALSE | FALSE |
| chr4 | 154421188 | 154421213 | 4 | 4 | L3 | TRUE | NA | 0 | 1 | FALSE | FALSE |
| chr4 | 155310436 | 155310479 | 4 | 3 | MER58B | TRUE | NA | 0 | 1 | TRUE | FALSE |
| chr4 | 155844602 | 155844603 | 4 | 4 | HERVH-int | TRUE | NA | 0 | NA | FALSE | FALSE |
| chr4 | 155899497 | 155899506 | 4 | 3 | LTR16A | TRUE | NA | 0 | 1 | FALSE | FALSE |
| chr4 | 157226774 | 157226827 | 7 | 4 | LTR5 Hs | TRUE | NA | 0 | NA | FALSE | FALSE |
| chr4 | 157232813 | 157232874 | 5 | 5 | NA | FALSE | NA | 0 | 1 | FALSE | FALSE |
| chr4 | 157308176 | 157308176 | 4 | 4 | LTR5A | TRUE | NA | 0 | NA | FALSE | FALSE |
| chr4 | 157308240 | 157308241 | 7 | 4 | LTR5A | TRUE | NA | 0 | NA | FALSE | FALSE |
| chr4 | 157570206 | 157570234 | 4 | 3 | NA | FALSE | NA | 0 | 1 | FALSE | FALSE |
| chr4 | 157844947 | 157844973 | 5 | 5 | L2c | TRUE | NA | 0 | 1 | TRUE | FALSE |
| chr4 | 157983788 | 157983811 | 4 | 3 | NA | FALSE | NA | 0 | 1 | FALSE | FALSE |
| chr4 | 158011844 | 158011880 | 4 | 4 | Charlie1a | TRUE | NA | 0 | 1 | FALSE | FALSE |
| chr4 | 158447388 | 158447410 | 4 | 3 | LTR25-int | TRUE | NA | 0 | 1 | FALSE | FALSE |
| chr4 | 158894399 | 158894407 | 4 | 2 | AluSp | TRUE | NA | 0 | NA | FALSE | FALSE |
| chr4 | 159241106 | 159241136 | 4 | 4 | LTR1D | TRUE | NA | 0 | 1 | FALSE | FALSE |
| chr4 | 159287166 | 159287206 | 4 | 4 | L1PA3 | TRUE | NA | 0 | NA | FALSE | FALSE |
| chr4 | 159355447 | 159355464 | 4 | 2 | L1PA3 | TRUE | NA | 0 | NA | FALSE | FALSE |
| chr4 | 159418682 | 159418720 | 4 | 4 | Charlie4z | TRUE | NA | 0 | 1 | FALSE | FALSE |
| chr4 | 160623261 | 160623288 | 4 | 3 | L1M3b | TRUE | NA | 0 | 1 | FALSE | FALSE |
| chr4 | 160721004 | 160721031 | 4 | 3 | NA | FALSE | NA | 0 | 1 | FALSE | FALSE |
| chr4 | 160762190 | 160762213 | 5 | 4 | L1PB3 | TRUE | NA | 0 | 1 | FALSE | FALSE |
| chr4 | 162370465 | 162370482 | 4 | 3 | NA | FALSE | NA | 0 | 1 | FALSE | FALSE |
| chr4 | 162658163 | 162658187 | 4 | 4 | NA | FALSE | NA | 0 | 1 | FALSE | FALSE |
| chr4 | 162658214 | 162658237 | 4 | 1 | NA | FALSE | NA | 0 | 1 | FALSE | FALSE |
| chr4 | 162710620 | 162710641 | 4 | 4 | LTR37A | TRUE | NA | 0 | 1 | FALSE | FALSE |
| chr4 | 163121569 | 163121573 | 4 | 4 | MER21A | TRUE | NA | 0 | 1 | TRUE | FALSE |
| chr4 | 163328790 | 163328810 | 4 | 2 | NA | FALSE | NA | 0 | 1 | FALSE | FALSE |
| chr4 | 163373997 | 163374018 | 4 | 2 | (TATATG)n | TRUE | NA | 0 | 1 | FALSE | FALSE |
| chr4 | 163451431 | 163451444 | 4 | 4 | NA | FALSE | NA | 0 | 1 | FALSE | FALSE |
| chr4 | 163479184 | 163479200 | 4 | 4 | PABL A-int | TRUE | NA | 0 | 1 | FALSE | FALSE |
| chr4 | 163716012 | 163716017 | 4 | 3 | AluY | TRUE | NA | 0 | NA | FALSE | FALSE |
| chr4 | 163722061 | 163722076 | 4 | 4 | L1PA7 | TRUE | NA | 0 | NA | FALSE | FALSE |
| chr4 | 164232703 | 164232734 | 5 | 5 | NA | FALSE | NA | 0 | 1 | FALSE | FALSE |
| chr4 | 164896347 | 164896349 | 4 | 4 | (TC)n | TRUE | NA | 0 | NA | TRUE | FALSE |
| chr4 | 165003661 | 165003661 | 4 | 4 | NA | FALSE | NA | 0 | 1 | FALSE | FALSE |
| chr4 | 165006074 | 165006084 | 4 | 4 | NA | FALSE | NA | 0 | 1 | TRUE | FALSE |
| chr4 | 165381807 | 165381825 | 4 | 3 | THE1B | TRUE | NA | 0 | 1 | FALSE | FALSE |
| chr4 | 165773927 | 165773952 | 4 | 3 | G-rich | TRUE | NA | 0 | 1 | FALSE | FALSE |
| chr4 | 165804188 | 165804188 | 4 | 4 | NA | FALSE | NA | 0 | 1 | FALSE | FALSE |
| chr4 | 165941511 | 165941518 | 4 | 2 | SVA D | TRUE | NA | 0 | NA | FALSE | FALSE |
| chr4 | 165960492 | 165960517 | 4 | 3 | L1MC4 | TRUE | NA | 0 | 1 | FALSE | FALSE |
| chr4 | 165994769 | 165994792 | 6 | 4 | LTR5B | TRUE | NA | 0 | NA | TRUE | FALSE |
| chr4 | 166165983 | 166165990 | 4 | 4 | NA | FALSE | NA | 0 | 1 | FALSE | FALSE |
| chr4 | 166523213 | 166523232 | 4 | 4 | NA | FALSE | NA | 0 | 1 | FALSE | FALSE |
| chr4 | 166580470 | 166580481 | 4 | 3 | AluSz | TRUE | NA | 0 | NA | FALSE | FALSE |
| chr4 | 166662093 | 166662099 | 4 | 4 | NA | FALSE | STAT3 | 1 | 1 | FALSE | FALSE |
| chr4 | 166702923 | 166702923 | 4 | 4 | NA | FALSE | NA | 0 | 1 | FALSE | FALSE |
| chr4 | 166869919 | 166869927 | 4 | 3 | MSTC | TRUE | NA | 0 | 1 | FALSE | FALSE |
| chr4 | 168297021 | 168297022 | 5 | 3 | AluSq2 | TRUE | NA | 0 | NA | FALSE | FALSE |
| chr4 | 168437499 | 168437510 | 5 | 5 | L2 | TRUE | NA | 0 | 1 | FALSE | FALSE |
| chr4 | 168472209 | 168472210 | 4 | 3 | MLT1D | TRUE | NA | 0 | NA | FALSE | FALSE |
| chr4 | 168635557 | 168635574 | 4 | 2 | NA | FALSE | NA | 0 | 1 | FALSE | FALSE |
| chr4 | 168938176 | 168938185 | 4 | 4 | ERV3-16A3 I-int | TRUE | NA | 0 | 1 | TRUE | FALSE |
| chr4 | 169009016 | 169009041 | 4 | 3 | L1MA7 | TRUE | KAP1 | 1 | 1 | FALSE | FALSE |
| chr4 | 169378431 | 169378445 | 6 | 5 | (CA)n | TRUE | NA | 0 | NA | FALSE | FALSE |
| chr4 | 170868119 | 170868149 | 4 | 2 | AluY | TRUE | NA | 0 | NA | FALSE | FALSE |
| chr4 | 171233580 | 171233589 | 5 | 4 | MER31A | TRUE | NA | 0 | NA | FALSE | FALSE |
| chr4 | 171432165 | 171432189 | 4 | 3 | L1PA5 | TRUE | NA | 0 | NA | FALSE | FALSE |
| chr4 | 171599019 | 171599020 | 4 | 3 | LTR7 | TRUE | NA | 0 | NA | FALSE | FALSE |
| chr4 | 171860579 | 171860596 | 4 | 2 | NA | FALSE | NA | 0 | 1 | FALSE | FALSE |
| chr4 | 172240420 | 172240468 | 4 | 3 | L2 | TRUE | NA | 0 | 1 | FALSE | FALSE |
| chr4 | 172707423 | 172707474 | 4 | 4 | NA | FALSE | NA | 0 | 1 | FALSE | FALSE |
| chr4 | 173099772 | 173099793 | 6 | 5 | L1PA7 | TRUE | NA | 0 | NA | FALSE | FALSE |
| chr4 | 173469461 | 173469485 | 4 | 3 | NA | FALSE | NA | 0 | 1 | TRUE | FALSE |
| chr4 | 173633595 | 173633630 | 4 | 3 | L1PA4 | TRUE | NA | 0 | 1 | FALSE | FALSE |
| chr4 | 173943188 | 173943208 | 4 | 4 | (GGAA)n | TRUE | NA | 0 | NA | TRUE | FALSE |
| chr4 | 174529411 | 174529428 | 5 | 5 | L1PA4 | TRUE | NA | 0 | NA | FALSE | FALSE |
| chr4 | 174560218 | 174560247 | 4 | 3 | L1PA4 | TRUE | NA | 0 | NA | FALSE | FALSE |
| chr4 | 174654261 | 174654289 | 4 | 3 | L2c | TRUE | NA | 0 | 1 | FALSE | FALSE |
| chr4 | 174711531 | 174711536 | 4 | 2 | L1PA4 | TRUE | NA | 0 | NA | FALSE | FALSE |
| chr4 | 174713963 | 174713967 | 4 | 3 | MamGypLTR1c | TRUE | NA | 0 | 1 | FALSE | FALSE |
| chr4 | 174745516 | 174745539 | 4 | 3 | NA | FALSE | NA | 0 | 1 | FALSE | FALSE |
| chr4 | 174792104 | 174792112 | 4 | 4 | NA | FALSE | NA | 0 | 1 | FALSE | FALSE |
| chr4 | 174848137 | 174848138 | 4 | 3 | AluSz | TRUE | NA | 0 | NA | FALSE | FALSE |
| chr4 | 175894074 | 175894087 | 4 | 4 | L1M3d | TRUE | NA | 0 | 1 | FALSE | FALSE |
| chr4 | 176174517 | 176174538 | 4 | 3 | HUERS-P2-int | TRUE | NA | 0 | 1 | FALSE | FALSE |
| chr4 | 176767157 | 176767157 | 4 | 4 | NA | FALSE | NA | 0 | 1 | FALSE | FALSE |
| chr4 | 177385496 | 177385516 | 4 | 3 | MER1B | TRUE | NA | 0 | 1 | FALSE | FALSE |
| chr4 | 178029798 | 178029829 | 4 | 3 | NA | FALSE | NA | 0 | 1 | TRUE | FALSE |
| chr4 | 178030830 | 178030832 | 4 | 3 | ERV3-16A3 I-int | TRUE | NA | 0 | 1 | FALSE | FALSE |
| chr4 | 178802775 | 178802801 | 4 | 3 | NA | FALSE | NA | 0 | 1 | FALSE | FALSE |
| chr4 | 179001050 | 179001061 | 4 | 3 | NA | FALSE | NA | 0 | 1 | FALSE | FALSE |
| chr4 | 179841767 | 179841793 | 4 | 3 | (TCCCC)n | TRUE | NA | 0 | NA | FALSE | FALSE |
| chr4 | 180091362 | 180091376 | 5 | 3 | LTR7 | TRUE | TAF1 | 1 | 1 | TRUE | FALSE |
| chr4 | 180467637 | 180467647 | 4 | 3 | MER50 | TRUE | NA | 0 | 1 | FALSE | FALSE |
| chr4 | 180646672 | 180646692 | 10 | 7 | HERVH-int | TRUE | NA | 0 | NA | FALSE | FALSE |
| chr4 | 181655535 | 181655583 | 4 | 3 | Tigger9b | TRUE | NA | 0 | 1 | FALSE | FALSE |
| chr4 | 181865647 | 181865687 | 4 | 4 | SVA F | TRUE | NA | 0 | NA | FALSE | FALSE |
| chr4 | 182056325 | 182056371 | 5 | 2 | NA | FALSE | NA | 0 | 1 | FALSE | FALSE |
| chr4 | 184772740 | 184772824 | 6 | 6 | MANY | TRUE | NA | 0 | NA | FALSE | FALSE |
| chr4 | 184773484 | 184773532 | 4 | 4 | G-rich | TRUE | NA | 0 | NA | FALSE | FALSE |
| chr4 | 184773640 | 184773693 | 5 | 5 | G-rich | TRUE | NA | 0 | NA | FALSE | FALSE |
| chr4 | 185192259 | 185192326 | 4 | 2 | (TCTA)n | TRUE | NA | 0 | 1 | FALSE | FALSE |
| chr4 | 185445279 | 185445322 | 4 | 2 | L1PA11 | TRUE | NA | 0 | NA | FALSE | FALSE |
| chr4 | 186246425 | 186246461 | 5 | 2 | AluSx3 | TRUE | NA | 0 | NA | FALSE | FALSE |
| chr4 | 186463228 | 186463281 | 4 | 3 | MANY | TRUE | NA | 0 | 1 | TRUE | FALSE |
| chr4 | 187058115 | 187058171 | 9 | 8 | (TC)n | TRUE | NA | 0 | 1 | TRUE | FALSE |
| chr4 | 187129236 | 187129278 | 4 | 3 | NA | FALSE | NA | 0 | 1 | FALSE | FALSE |
| chr4 | 187234699 | 187234722 | 4 | 3 | NA | FALSE | NA | 0 | 1 | FALSE | FALSE |
| chr4 | 187357620 | 187357645 | 4 | 3 | NA | FALSE | NA | 0 | NA | TRUE | FALSE |
| chr4 | 187407152 | 187407192 | 4 | 3 | (CA)n | TRUE | NA | 0 | NA | TRUE | FALSE |
| chr4 | 187973081 | 187973111 | 4 | 2 | L1PA3 | TRUE | NA | 0 | NA | FALSE | FALSE |
| chr4 | 188218482 | 188218482 | 4 | 4 | THE1A | TRUE | NA | 0 | NA | FALSE | FALSE |
| chr4 | 188554205 | 188554232 | 4 | 3 | NA | FALSE | NA | 0 | 1 | FALSE | FALSE |
| chr4 | 188821287 | 188821296 | 4 | 3 | L2 | TRUE | NA | 0 | 1 | FALSE | FALSE |
| chr4 | 189374768 | 189374791 | 4 | 3 | L2b | TRUE | NA | 0 | 1 | FALSE | FALSE |
| chr4 | 189375671 | 189375685 | 4 | 2 | (CA)n | TRUE | NA | 0 | NA | FALSE | FALSE |
| chr4 | 189429715 | 189429746 | 4 | 4 | NA | FALSE | NA | 0 | 1 | FALSE | FALSE |
| chr4 | 189552189 | 189552218 | 4 | 2 | NA | FALSE | NA | 0 | 1 | FALSE | FALSE |
| chr4 | 189713112 | 189713137 | 4 | 4 | NA | FALSE | NA | 0 | 1 | FALSE | FALSE |
| chr4 | 190139321 | 190139341 | 4 | 3 | NA | FALSE | NA | 0 | 1 | FALSE | FALSE |
| chr4 | 190396826 | 190396839 | 4 | 3 | NA | FALSE | NA | 0 | 1 | FALSE | FALSE |
| chr4 | 190508464 | 190508464 | 4 | 4 | NA | FALSE | NA | 0 | 1 | FALSE | FALSE |
| chr4 | 190512032 | 190512039 | 4 | 3 | NA | FALSE | NA | 0 | 1 | TRUE | FALSE |
| chr4 | 190522605 | 190522605 | 4 | 4 | NA | FALSE | NA | 0 | 1 | TRUE | FALSE |
| chr4 | 190530200 | 190530225 | 5 | 3 | NA | FALSE | IRF3 | 1 | 1 | TRUE | FALSE |
| chr4 | 190539339 | 190539339 | 6 | 6 | NA | FALSE | NA | 0 | 1 | TRUE | FALSE |
| chr4 | 190640370 | 190640374 | 4 | 4 | HAL1-3A ME | TRUE | CEBPB | 1 | 1 | FALSE | FALSE |
| chr4 | 190657389 | 190657397 | 5 | 4 | NA | FALSE | NA | 0 | NA | FALSE | FALSE |
| chr4 | 190807563 | 190807583 | 4 | 3 | L2a | TRUE | NA | 0 | 1 | TRUE | FALSE |
| chr4 | 190811159 | 190811183 | 4 | 4 | Ricksha c | TRUE | NA | 0 | 1 | TRUE | FALSE |
| chr4 | 190812883 | 190812900 | 4 | 4 | Ricksha | TRUE | NA | 0 | 1 | TRUE | FALSE |
| chr4 | 190814001 | 190814020 | 6 | 5 | NA | FALSE | NA | 0 | 1 | TRUE | FALSE |
| chr4 | 190821630 | 190821657 | 7 | 5 | NA | FALSE | NA | 0 | 1 | TRUE | FALSE |
| chr4 | 190824895 | 190824910 | 5 | 5 | NA | FALSE | NA | 0 | 1 | TRUE | FALSE |
| chr4 | 190824952 | 190824964 | 4 | 3 | NA | FALSE | NA | 0 | NA | TRUE | FALSE |
| chr4 | 190832292 | 190832320 | 4 | 4 | NA | FALSE | NA | 0 | NA | FALSE | FALSE |
| chr4 | 190834510 | 190834511 | 4 | 3 | NA | FALSE | NA | 0 | 1 | FALSE | FALSE |
| chr4 | 190834714 | 190834734 | 4 | 4 | NA | FALSE | NA | 0 | 1 | TRUE | FALSE |
| chr4 | 190843600 | 190843621 | 4 | 4 | L1M1 | TRUE | NA | 0 | NA | FALSE | FALSE |
| chr4 | 190844085 | 190844139 | 6 | 4 | L1M1 | TRUE | NA | 0 | 1 | TRUE | FALSE |
| chr4 | 190844376 | 190844376 | 5 | 5 | L1M1 | TRUE | NA | 0 | NA | TRUE | FALSE |
| chr4 | 190844514 | 190844542 | 9 | 5 | L1M1 | TRUE | NA | 0 | NA | TRUE | FALSE |
| chr4 | 190846365 | 190846381 | 5 | 4 | L1M1 | TRUE | NA | 0 | 1 | FALSE | FALSE |
| chr4 | 190865191 | 190865197 | 5 | 3 | L1MEc | TRUE | NA | 0 | 1 | TRUE | FALSE |
| chr4 | 190871586 | 190871586 | 4 | 4 | L1M4 | TRUE | NA | 0 | NA | FALSE | FALSE |
| chr4 | 190877313 | 190877325 | 6 | 4 | MER112 | TRUE | NA | 0 | 1 | FALSE | FALSE |
| chr4 | 190877928 | 190877929 | 5 | 4 | NA | FALSE | NA | 0 | 1 | FALSE | FALSE |
| chr4 | 190878158 | 190878173 | 4 | 4 | NA | FALSE | NA | 0 | NA | FALSE | FALSE |
| chr4 | 190878418 | 190878448 | 4 | 4 | L1ME4a | TRUE | NA | 0 | NA | TRUE | FALSE |
| chr4 | 190881701 | 190881701 | 4 | 4 | NA | FALSE | NA | 0 | NA | FALSE | FALSE |
| chr4 | 190882429 | 190882491 | 6 | 4 | NA | FALSE | NA | 0 | 1 | TRUE | FALSE |
| chr4 | 190883474 | 190883505 | 4 | 4 | NA | FALSE | NA | 0 | 1 | FALSE | FALSE |
| chr4 | 190883569 | 190883596 | 4 | 2 | NA | FALSE | NA | 0 | 1 | TRUE | FALSE |
| chr4 | 190884071 | 190884157 | 10 | 8 | NA | FALSE | NA | 0 | 1 | FALSE | FALSE |
| chr4 | 190925760 | 190925808 | 9 | 9 | L1PA14 | TRUE | NA | 0 | NA | FALSE | FALSE |
| chr5 | 29973 | 30073 | 6 | 4 | NA | FALSE | NA | 0 | 1 | FALSE | FALSE |
| chr5 | 112465 | 112472 | 4 | 3 | NA | FALSE | NA | 0 | 1 | FALSE | FALSE |
| chr5 | 165431 | 165496 | 4 | 4 | NA | FALSE | NA | 0 | NA | TRUE | FALSE |
| chr5 | 165592 | 165659 | 4 | 3 | NA | FALSE | NA | 0 | NA | FALSE | FALSE |
| chr5 | 230414 | 230441 | 5 | 4 | NA | FALSE | NA | 0 | 1 | FALSE | FALSE |
| chr5 | 252035 | 252096 | 5 | 5 | NA | FALSE | NA | 0 | NA | FALSE | FALSE |
| chr5 | 258853 | 258885 | 4 | 3 | NA | FALSE | NA | 0 | NA | FALSE | FALSE |
| chr5 | 262349 | 262413 | 6 | 6 | MER1B | TRUE | NA | 0 | NA | TRUE | FALSE |
| chr5 | 265509 | 265547 | 6 | 4 | L1MC4a | TRUE | NA | 0 | 1 | TRUE | FALSE |
| chr5 | 398311 | 398355 | 4 | 3 | NA | FALSE | NA | 0 | NA | TRUE | FALSE |
| chr5 | 584683 | 584730 | 5 | 4 | (TGGG)n | TRUE | NA | 0 | NA | TRUE | FALSE |
| chr5 | 584770 | 584882 | 12 | 11 | (TGGG)n | TRUE | Egr-1 | 1 | 1 | FALSE | FALSE |
| chr5 | 649669 | 649707 | 5 | 5 | NA | FALSE | NA | 0 | 1 | FALSE | FALSE |
| chr5 | 792931 | 792932 | 5 | 3 | NA | FALSE | NA | 0 | 1 | TRUE | FALSE |
| chr5 | 1098324 | 1098363 | 4 | 4 | NA | FALSE | NA | 0 | NA | TRUE | FALSE |
| chr5 | 1136879 | 1136880 | 4 | 3 | (CA)n | TRUE | NA | 0 | NA | FALSE | FALSE |
| chr5 | 1157868 | 1157912 | 8 | 3 | NA | FALSE | ZEB1 (SC-25388) | 1 | 1 | TRUE | FALSE |
| chr5 | 1295227 | 1295252 | 37 | 29 | (CGGGG)n | TRUE | MANY | 6 | 1 | TRUE | TRUE |
| chr5 | 1606721 | 1606731 | 4 | 2 | NA | FALSE | NA | 0 | 1 | TRUE | FALSE |
| chr5 | 1621942 | 1621957 | 4 | 2 | MLT2B4 | TRUE | NA | 0 | 1 | FALSE | FALSE |
| chr5 | 1628030 | 1628030 | 5 | 5 | LTR78B | TRUE | NA | 0 | NA | FALSE | FALSE |
| chr5 | 1641142 | 1641142 | 4 | 4 | NA | FALSE | NA | 0 | 1 | TRUE | FALSE |
| chr5 | 1935475 | 1935496 | 4 | 3 | L1ME2z | TRUE | NA | 0 | 1 | TRUE | FALSE |
| chr5 | 2045062 | 2045081 | 4 | 4 | L2 | TRUE | NA | 0 | 1 | FALSE | FALSE |
| chr5 | 2115889 | 2115904 | 4 | 4 | THE1B | TRUE | NA | 0 | NA | FALSE | FALSE |
| chr5 | 2867833 | 2867845 | 4 | 4 | NA | FALSE | NA | 0 | 1 | TRUE | FALSE |
| chr5 | 2873907 | 2873922 | 4 | 4 | MSTA | TRUE | NA | 0 | NA | FALSE | FALSE |
| chr5 | 2930888 | 2930903 | 4 | 2 | NA | FALSE | NA | 0 | 1 | FALSE | FALSE |
| chr5 | 2960374 | 2960387 | 4 | 4 | NA | FALSE | NA | 0 | 1 | FALSE | FALSE |
| chr5 | 3761713 | 3761719 | 4 | 3 | NA | FALSE | NA | 0 | NA | FALSE | FALSE |
| chr5 | 3858723 | 3858725 | 4 | 2 | NA | FALSE | NA | 0 | 1 | TRUE | FALSE |
| chr5 | 3884727 | 3884728 | 4 | 2 | NA | FALSE | NA | 0 | 1 | TRUE | FALSE |
| chr5 | 3901759 | 3901781 | 4 | 4 | NA | FALSE | NA | 0 | 1 | FALSE | FALSE |
| chr5 | 4554725 | 4554736 | 4 | 4 | NA | FALSE | NA | 0 | 1 | TRUE | FALSE |
| chr5 | 4831852 | 4831862 | 4 | 3 | NA | FALSE | NA | 0 | 1 | FALSE | FALSE |
| chr5 | 5058523 | 5058537 | 4 | 3 | MLT1A0 | TRUE | NA | 0 | 1 | FALSE | FALSE |
| chr5 | 5506893 | 5506921 | 6 | 5 | L1ME1 | TRUE | NA | 0 | 1 | FALSE | FALSE |
| chr5 | 5896377 | 5896394 | 4 | 4 | MER1A | TRUE | NA | 0 | 1 | FALSE | FALSE |
| chr5 | 5983729 | 5983735 | 4 | 4 | NA | FALSE | NA | 0 | 1 | TRUE | FALSE |
| chr5 | 6019804 | 6019805 | 4 | 3 | NA | FALSE | MANY | 2 | 1 | FALSE | FALSE |
| chr5 | 6879601 | 6879610 | 4 | 2 | NA | FALSE | NA | 0 | 1 | FALSE | FALSE |
| chr5 | 7074138 | 7074168 | 4 | 3 | AluSx | TRUE | NA | 0 | NA | FALSE | FALSE |
| chr5 | 7136157 | 7136194 | 4 | 3 | NA | FALSE | NA | 0 | 1 | FALSE | FALSE |
| chr5 | 7301918 | 7301954 | 4 | 4 | NA | FALSE | NA | 0 | NA | FALSE | FALSE |
| chr5 | 7513216 | 7513244 | 4 | 3 | MANY | TRUE | NA | 0 | NA | FALSE | FALSE |
| chr5 | 8285424 | 8285446 | 4 | 4 | L1PA6 | TRUE | NA | 0 | NA | FALSE | FALSE |
| chr5 | 8543238 | 8543263 | 4 | 4 | NA | FALSE | NA | 0 | 1 | FALSE | FALSE |
| chr5 | 9737218 | 9737232 | 4 | 4 | NA | FALSE | NA | 0 | 1 | FALSE | FALSE |
| chr5 | 9754694 | 9754713 | 4 | 4 | L1PA4 | TRUE | NA | 0 | 1 | FALSE | FALSE |
| chr5 | 9902773 | 9902789 | 4 | 4 | L1MCa | TRUE | NA | 0 | 1 | FALSE | FALSE |
| chr5 | 9965025 | 9965044 | 4 | 4 | NA | FALSE | NA | 0 | 1 | FALSE | FALSE |
| chr5 | 10430528 | 10430561 | 5 | 2 | AluSc | TRUE | NA | 0 | NA | FALSE | FALSE |
| chr5 | 10898244 | 10898264 | 4 | 3 | Tigger2a | TRUE | NA | 0 | 1 | FALSE | FALSE |
| chr5 | 11120424 | 11120437 | 6 | 4 | NA | FALSE | NA | 0 | 1 | TRUE | FALSE |
| chr5 | 11183670 | 11183672 | 4 | 2 | AluSq | TRUE | NA | 0 | NA | FALSE | FALSE |
| chr5 | 11783030 | 11783031 | 4 | 3 | LTR79 | TRUE | NA | 0 | 1 | FALSE | FALSE |
| chr5 | 11813117 | 11813118 | 4 | 4 | NA | FALSE | NA | 0 | 1 | FALSE | FALSE |
| chr5 | 12650580 | 12650591 | 4 | 3 | NA | FALSE | NA | 0 | 1 | FALSE | FALSE |
| chr5 | 13147507 | 13147513 | 4 | 3 | LTR16A | TRUE | NA | 0 | 1 | FALSE | FALSE |
| chr5 | 13579190 | 13579202 | 4 | 4 | LTR12C | TRUE | NA | 0 | NA | FALSE | FALSE |
| chr5 | 13674710 | 13674719 | 4 | 4 | NA | FALSE | NA | 0 | 1 | FALSE | FALSE |
| chr5 | 13854769 | 13854777 | 4 | 4 | L1PA7 | TRUE | NA | 0 | NA | FALSE | FALSE |
| chr5 | 14573900 | 14573935 | 4 | 3 | AluY | TRUE | NA | 0 | NA | FALSE | FALSE |
| chr5 | 15036912 | 15036917 | 4 | 3 | NA | FALSE | NA | 0 | 1 | FALSE | FALSE |
| chr5 | 15091956 | 15091991 | 4 | 3 | NA | FALSE | NA | 0 | 1 | FALSE | FALSE |
| chr5 | 15987631 | 15987657 | 4 | 4 | L1MA9 | TRUE | NA | 0 | 1 | FALSE | FALSE |
| chr5 | 16300029 | 16300034 | 4 | 3 | NA | FALSE | NA | 0 | 1 | FALSE | FALSE |
| chr5 | 16378407 | 16378434 | 4 | 4 | MIRb | TRUE | NA | 0 | 1 | FALSE | FALSE |
| chr5 | 16392084 | 16392095 | 4 | 2 | NA | FALSE | NA | 0 | 1 | TRUE | FALSE |
| chr5 | 16649296 | 16649338 | 4 | 3 | L1P1 | TRUE | NA | 0 | NA | FALSE | FALSE |
| chr5 | 17646934 | 17646970 | 4 | 4 | L1PBa | TRUE | NA | 0 | NA | FALSE | FALSE |
| chr5 | 17900773 | 17900799 | 4 | 2 | (TA)n | TRUE | NA | 0 | NA | FALSE | FALSE |
| chr5 | 17956079 | 17956116 | 4 | 3 | NA | FALSE | NA | 0 | 1 | FALSE | FALSE |
| chr5 | 18145693 | 18145710 | 4 | 2 | NA | FALSE | NA | 0 | 1 | FALSE | FALSE |
| chr5 | 18198376 | 18198399 | 4 | 2 | MST-int | TRUE | NA | 0 | 1 | FALSE | FALSE |
| chr5 | 20344181 | 20344182 | 4 | 2 | MER52-int | TRUE | NA | 0 | 1 | FALSE | FALSE |
| chr5 | 20816592 | 20816607 | 7 | 5 | L1P3 | TRUE | NA | 0 | 1 | FALSE | FALSE |
| chr5 | 21324482 | 21324528 | 8 | 4 | NA | FALSE | NA | 0 | 1 | FALSE | FALSE |
| chr5 | 21350197 | 21350210 | 5 | 3 | AluSq | TRUE | NA | 0 | 1 | FALSE | FALSE |
| chr5 | 21520075 | 21520082 | 5 | 4 | NA | FALSE | NA | 0 | 1 | FALSE | FALSE |
| chr5 | 21776049 | 21776074 | 4 | 4 | MLT1F2 | TRUE | NA | 0 | 1 | FALSE | FALSE |
| chr5 | 21940470 | 21940474 | 4 | 3 | NA | FALSE | NA | 0 | 1 | FALSE | FALSE |
| chr5 | 21940966 | 21940966 | 5 | 5 | (TC)n | TRUE | NA | 0 | NA | FALSE | FALSE |
| chr5 | 21946002 | 21946008 | 4 | 4 | L1MDa | TRUE | NA | 0 | 1 | FALSE | FALSE |
| chr5 | 22004154 | 22004170 | 8 | 6 | NA | FALSE | NA | 0 | 1 | FALSE | FALSE |
| chr5 | 22005997 | 22005997 | 5 | 5 | NA | FALSE | NA | 0 | 1 | FALSE | FALSE |
| chr5 | 22059426 | 22059434 | 4 | 3 | (TCTA)n | TRUE | NA | 0 | 1 | FALSE | FALSE |
| chr5 | 22932226 | 22932242 | 4 | 3 | HERV9-int | TRUE | NA | 0 | 1 | FALSE | FALSE |
| chr5 | 23052410 | 23052415 | 4 | 4 | L1ME3B | TRUE | NA | 0 | 1 | FALSE | FALSE |
| chr5 | 23416737 | 23416738 | 4 | 3 | NA | FALSE | NA | 0 | 1 | FALSE | FALSE |
| chr5 | 23544618 | 23544624 | 4 | 4 | L1PREC2 | TRUE | NA | 0 | 1 | FALSE | FALSE |
| chr5 | 23620620 | 23620636 | 4 | 3 | NA | FALSE | NA | 0 | 1 | FALSE | FALSE |
| chr5 | 23864473 | 23864478 | 4 | 3 | MER61-int | TRUE | NA | 0 | 1 | FALSE | FALSE |
| chr5 | 24030303 | 24030303 | 4 | 4 | L1PB | TRUE | NA | 0 | NA | FALSE | FALSE |
| chr5 | 24258299 | 24258305 | 5 | 3 | NA | FALSE | NA | 0 | 1 | FALSE | FALSE |
| chr5 | 24305786 | 24305795 | 4 | 4 | L1PA7 | TRUE | NA | 0 | NA | FALSE | FALSE |
| chr5 | 24517507 | 24517515 | 5 | 3 | L1MC4 | TRUE | NA | 0 | 1 | FALSE | FALSE |
| chr5 | 25401781 | 25401796 | 4 | 4 | NA | FALSE | NA | 0 | 1 | FALSE | FALSE |
| chr5 | 25587534 | 25587547 | 4 | 4 | L1ME3A | TRUE | NA | 0 | 1 | FALSE | FALSE |
| chr5 | 25668407 | 25668422 | 4 | 3 | NA | FALSE | NA | 0 | 1 | FALSE | FALSE |
| chr5 | 25754002 | 25754019 | 4 | 4 | NA | FALSE | NA | 0 | 1 | FALSE | FALSE |
| chr5 | 26325558 | 26325582 | 7 | 5 | LTR12C | TRUE | NA | 0 | NA | FALSE | FALSE |
| chr5 | 27173730 | 27173740 | 4 | 3 | Charlie3 | TRUE | NA | 0 | 1 | FALSE | FALSE |
| chr5 | 27308632 | 27308640 | 4 | 4 | L1ME1 | TRUE | NA | 0 | 1 | FALSE | FALSE |
| chr5 | 27860409 | 27860415 | 4 | 4 | MER4-int | TRUE | NA | 0 | 1 | FALSE | FALSE |
| chr5 | 28260392 | 28260400 | 5 | 3 | NA | FALSE | NA | 0 | 1 | FALSE | FALSE |
| chr5 | 28385582 | 28385590 | 5 | 4 | AluSq2 | TRUE | NA | 0 | NA | FALSE | FALSE |
| chr5 | 28610332 | 28610335 | 4 | 4 | AluSc | TRUE | NA | 0 | NA | FALSE | FALSE |
| chr5 | 28651398 | 28651403 | 4 | 4 | L1MA7 | TRUE | NA | 0 | NA | FALSE | FALSE |
| chr5 | 28708029 | 28708029 | 4 | 4 | NA | FALSE | NA | 0 | 1 | TRUE | FALSE |
| chr5 | 28834122 | 28834130 | 4 | 4 | NA | FALSE | NA | 0 | 1 | FALSE | FALSE |
| chr5 | 28966475 | 28966479 | 4 | 4 | NA | FALSE | NA | 0 | 1 | FALSE | FALSE |
| chr5 | 29387619 | 29387634 | 4 | 4 | NA | FALSE | NA | 0 | 1 | FALSE | FALSE |
| chr5 | 29435644 | 29435656 | 4 | 2 | Tigger7 | TRUE | NA | 0 | 1 | FALSE | FALSE |
| chr5 | 29435830 | 29435857 | 5 | 5 | NA | FALSE | NA | 0 | 1 | FALSE | FALSE |
| chr5 | 29452285 | 29452302 | 4 | 4 | L2a | TRUE | NA | 0 | 1 | FALSE | FALSE |
| chr5 | 29523354 | 29523369 | 4 | 4 | NA | FALSE | NA | 0 | 1 | FALSE | FALSE |
| chr5 | 29766784 | 29766784 | 4 | 4 | L1PA16 | TRUE | NA | 0 | 1 | FALSE | FALSE |
| chr5 | 29837379 | 29837384 | 4 | 4 | (TTCC)n | TRUE | NA | 0 | NA | FALSE | FALSE |
| chr5 | 30017591 | 30017606 | 4 | 4 | NA | FALSE | NA | 0 | 1 | FALSE | FALSE |
| chr5 | 30150062 | 30150073 | 4 | 3 | NA | FALSE | NA | 0 | 1 | FALSE | FALSE |
| chr5 | 30235101 | 30235113 | 4 | 4 | THE1B | TRUE | NA | 0 | 1 | FALSE | FALSE |
| chr5 | 30235531 | 30235531 | 4 | 4 | MER57-int | TRUE | NA | 0 | 1 | FALSE | FALSE |
| chr5 | 30501015 | 30501025 | 4 | 4 | NA | FALSE | NA | 0 | 1 | FALSE | FALSE |
| chr5 | 30526015 | 30526032 | 4 | 4 | NA | FALSE | NA | 0 | 1 | TRUE | FALSE |
| chr5 | 31655330 | 31655369 | 11 | 6 | AluSx3 | TRUE | NA | 0 | NA | FALSE | FALSE |
| chr5 | 31706849 | 31706883 | 6 | 4 | AluSc | TRUE | NA | 0 | NA | FALSE | FALSE |
| chr5 | 31983413 | 31983491 | 4 | 4 | NA | FALSE | NA | 0 | 1 | TRUE | FALSE |
| chr5 | 32175697 | 32175762 | 4 | 3 | AluY | TRUE | NA | 0 | NA | FALSE | FALSE |
| chr5 | 32585649 | 32585656 | 4 | 2 | NA | FALSE | MANY | 47 | 1 | TRUE | TRUE |
| chr5 | 32674451 | 32674476 | 4 | 2 | HERV9-int | TRUE | NA | 0 | NA | FALSE | FALSE |
| chr5 | 33181231 | 33181248 | 4 | 3 | L1MC4a | TRUE | MANY | 5 | 1 | TRUE | TRUE |
| chr5 | 34397526 | 34397579 | 4 | 3 | Tigger1 | TRUE | NA | 0 | 1 | FALSE | FALSE |
| chr5 | 34643843 | 34643887 | 4 | 2 | L1PA6 | TRUE | NA | 0 | NA | FALSE | FALSE |
| chr5 | 34972923 | 34972926 | 5 | 3 | L1PB1 | TRUE | NA | 0 | NA | FALSE | FALSE |
| chr5 | 35055546 | 35055555 | 4 | 4 | NA | FALSE | NA | 0 | 1 | TRUE | FALSE |
| chr5 | 35119980 | 35120006 | 4 | 2 | NA | FALSE | NA | 0 | 1 | TRUE | FALSE |
| chr5 | 35740999 | 35741006 | 4 | 3 | NA | FALSE | NA | 0 | 1 | FALSE | FALSE |
| chr5 | 35990704 | 35990712 | 4 | 4 | NA | FALSE | NA | 0 | 1 | FALSE | FALSE |
| chr5 | 35993620 | 35993620 | 4 | 4 | PRIMA4 LTR | TRUE | NA | 0 | 1 | FALSE | FALSE |
| chr5 | 36343174 | 36343194 | 4 | 4 | L1MEc | TRUE | NA | 0 | 1 | FALSE | FALSE |
| chr5 | 36827054 | 36827079 | 4 | 4 | MANY | TRUE | NA | 0 | 1 | FALSE | FALSE |
| chr5 | 37261598 | 37261618 | 4 | 2 | L1PA5 | TRUE | NA | 0 | NA | FALSE | FALSE |
| chr5 | 37564598 | 37564639 | 5 | 3 | SVA D | TRUE | NA | 0 | 1 | FALSE | FALSE |
| chr5 | 37797371 | 37797376 | 4 | 3 | HERVL-int | TRUE | NA | 0 | 1 | FALSE | FALSE |
| chr5 | 38018587 | 38018607 | 4 | 4 | NA | FALSE | NA | 0 | 1 | FALSE | FALSE |
| chr5 | 38024379 | 38024405 | 4 | 4 | L1ME3C | TRUE | NA | 0 | 1 | FALSE | FALSE |
| chr5 | 38206951 | 38206977 | 4 | 4 | CT-rich | TRUE | NA | 0 | 1 | FALSE | FALSE |
| chr5 | 38381616 | 38381624 | 4 | 4 | LTR33 | TRUE | NA | 0 | 1 | TRUE | FALSE |
| chr5 | 38672073 | 38672100 | 4 | 3 | NA | FALSE | NA | 0 | 1 | FALSE | FALSE |
| chr5 | 38893358 | 38893386 | 4 | 4 | L1PA15-16 | TRUE | NA | 0 | 1 | FALSE | FALSE |
| chr5 | 39331382 | 39331401 | 4 | 4 | NA | FALSE | NA | 0 | 1 | FALSE | FALSE |
| chr5 | 39875088 | 39875110 | 4 | 4 | NA | FALSE | NA | 0 | 1 | FALSE | FALSE |
| chr5 | 39897728 | 39897744 | 4 | 4 | NA | FALSE | NA | 0 | 1 | FALSE | FALSE |
| chr5 | 40112828 | 40112838 | 4 | 3 | MER11C | TRUE | NA | 0 | NA | FALSE | FALSE |
| chr5 | 41036901 | 41036907 | 4 | 4 | NA | FALSE | NA | 0 | 1 | FALSE | FALSE |
| chr5 | 41039001 | 41039014 | 4 | 4 | NA | FALSE | NA | 0 | 1 | FALSE | FALSE |
| chr5 | 41357655 | 41357656 | 4 | 4 | NA | FALSE | NA | 0 | 1 | FALSE | FALSE |
| chr5 | 41429313 | 41429320 | 4 | 3 | NA | FALSE | NA | 0 | 1 | TRUE | FALSE |
| chr5 | 41446081 | 41446094 | 4 | 2 | AluY | TRUE | NA | 0 | NA | FALSE | FALSE |
| chr5 | 41470621 | 41470645 | 5 | 4 | HAL1 | TRUE | NA | 0 | 1 | TRUE | FALSE |
| chr5 | 41475574 | 41475584 | 4 | 4 | NA | FALSE | NA | 0 | 1 | TRUE | FALSE |
| chr5 | 41524091 | 41524099 | 4 | 3 | LTR50 | TRUE | NA | 0 | 1 | FALSE | FALSE |
| chr5 | 41566136 | 41566157 | 4 | 4 | L1PA3 | TRUE | NA | 0 | NA | FALSE | FALSE |
| chr5 | 42159045 | 42159054 | 4 | 2 | NA | FALSE | NA | 0 | 1 | FALSE | FALSE |
| chr5 | 42204939 | 42204947 | 5 | 4 | L1MEc | TRUE | NA | 0 | 1 | FALSE | FALSE |
| chr5 | 42492350 | 42492370 | 4 | 4 | NA | FALSE | NA | 0 | 1 | FALSE | FALSE |
| chr5 | 43263646 | 43263668 | 4 | 3 | L1PA5 | TRUE | NA | 0 | NA | FALSE | FALSE |
| chr5 | 44580036 | 44580036 | 4 | 4 | L1PA3 | TRUE | NA | 0 | NA | FALSE | FALSE |
| chr5 | 45120419 | 45120433 | 5 | 5 | L1PA5 | TRUE | NA | 0 | NA | FALSE | FALSE |
| chr5 | 45147240 | 45147260 | 4 | 4 | L1MC3 | TRUE | NA | 0 | 1 | FALSE | FALSE |
| chr5 | 49693750 | 49693769 | 4 | 3 | NA | FALSE | NA | 0 | 1 | FALSE | FALSE |
| chr5 | 49771176 | 49771190 | 5 | 4 | THE1B-int | TRUE | NA | 0 | NA | FALSE | FALSE |
| chr5 | 49790924 | 49790936 | 4 | 3 | L1PA4 | TRUE | NA | 0 | NA | FALSE | FALSE |
| chr5 | 49808824 | 49808834 | 5 | 3 | MIR | TRUE | NA | 0 | 1 | FALSE | FALSE |
| chr5 | 49875537 | 49875539 | 4 | 3 | L1PB1 | TRUE | NA | 0 | 1 | FALSE | FALSE |
| chr5 | 50441290 | 50441311 | 4 | 4 | AluJo | TRUE | NA | 0 | 1 | FALSE | FALSE |
| chr5 | 50511428 | 50511448 | 4 | 3 | NA | FALSE | NA | 0 | 1 | FALSE | FALSE |
| chr5 | 50598786 | 50598802 | 4 | 4 | NA | FALSE | NA | 0 | 1 | FALSE | FALSE |
| chr5 | 51575966 | 51575974 | 4 | 3 | L1PA4 | TRUE | NA | 0 | NA | FALSE | FALSE |
| chr5 | 51938276 | 51938316 | 4 | 3 | L1P1 | TRUE | NA | 0 | 1 | FALSE | FALSE |
| chr5 | 52200573 | 52200609 | 5 | 5 | AluY | TRUE | NA | 0 | NA | FALSE | FALSE |
| chr5 | 54561137 | 54561145 | 4 | 3 | NA | FALSE | NA | 0 | 1 | FALSE | FALSE |
| chr5 | 54909384 | 54909429 | 4 | 3 | LTR12C | TRUE | NA | 0 | 1 | TRUE | FALSE |
| chr5 | 55034252 | 55034325 | 4 | 3 | NA | FALSE | NA | 0 | 1 | TRUE | FALSE |
| chr5 | 56405492 | 56405502 | 6 | 4 | AluSc | TRUE | NA | 0 | 1 | FALSE | FALSE |
| chr5 | 57265145 | 57265239 | 11 | 8 | L1PA4 | TRUE | NA | 0 | 1 | FALSE | FALSE |
| chr5 | 57678315 | 57678356 | 4 | 3 | MER41B | TRUE | STAT1 | 1 | 1 | FALSE | FALSE |
| chr5 | 58816342 | 58816445 | 4 | 3 | NA | FALSE | NA | 0 | 1 | FALSE | FALSE |
| chr5 | 60010832 | 60010892 | 4 | 4 | L1PA7 | TRUE | NA | 0 | 1 | FALSE | FALSE |
| chr5 | 60627917 | 60627955 | 4 | 4 | NA | FALSE | MANY | 16 | 1 | TRUE | TRUE |
| chr5 | 61542664 | 61542678 | 4 | 2 | NA | FALSE | NA | 0 | 1 | FALSE | FALSE |
| chr5 | 61679967 | 61679967 | 5 | 5 | AluSx | TRUE | NA | 0 | NA | FALSE | FALSE |
| chr5 | 61809160 | 61809163 | 4 | 3 | SVA D | TRUE | NA | 0 | NA | FALSE | FALSE |
| chr5 | 62277524 | 62277542 | 4 | 4 | LTR78B | TRUE | NA | 0 | 1 | TRUE | FALSE |
| chr5 | 62288545 | 62288545 | 4 | 4 | L1MC1 | TRUE | NA | 0 | 1 | FALSE | FALSE |
| chr5 | 62329528 | 62329541 | 5 | 4 | THE1D | TRUE | NA | 0 | NA | FALSE | FALSE |
| chr5 | 62557436 | 62557436 | 4 | 4 | AluSq2 | TRUE | NA | 0 | NA | FALSE | FALSE |
| chr5 | 62598515 | 62598523 | 4 | 3 | L2b | TRUE | NA | 0 | 1 | FALSE | FALSE |
| chr5 | 63218351 | 63218380 | 4 | 3 | NA | FALSE | NA | 0 | 1 | FALSE | FALSE |
| chr5 | 63347976 | 63347999 | 4 | 3 | NA | FALSE | NA | 0 | 1 | FALSE | FALSE |
| chr5 | 63409141 | 63409165 | 4 | 2 | NA | FALSE | NA | 0 | 1 | FALSE | FALSE |
| chr5 | 63728313 | 63728321 | 4 | 3 | L1MA4 | TRUE | NA | 0 | NA | FALSE | FALSE |
| chr5 | 63755477 | 63755498 | 4 | 4 | L1M2 | TRUE | NA | 0 | 1 | FALSE | FALSE |
| chr5 | 63809230 | 63809263 | 4 | 3 | NA | FALSE | NA | 0 | 1 | TRUE | FALSE |
| chr5 | 63972192 | 63972192 | 4 | 4 | (TTTC)n | TRUE | NA | 0 | NA | FALSE | FALSE |
| chr5 | 64511801 | 64511825 | 4 | 3 | L1M5 | TRUE | NA | 0 | 1 | FALSE | FALSE |
| chr5 | 64689120 | 64689167 | 4 | 2 | NA | FALSE | NA | 0 | 1 | FALSE | FALSE |
| chr5 | 64717282 | 64717292 | 4 | 4 | L1PBa | TRUE | NA | 0 | 1 | FALSE | FALSE |
| chr5 | 64721020 | 64721028 | 4 | 3 | AluSq2 | TRUE | NA | 0 | 1 | FALSE | FALSE |
| chr5 | 65181885 | 65181891 | 6 | 6 | (TC)n | TRUE | MANY | 10 | NA | TRUE | FALSE |
| chr5 | 65272988 | 65273038 | 5 | 5 | SVA D | TRUE | NA | 0 | NA | FALSE | FALSE |
| chr5 | 65679886 | 65679918 | 5 | 4 | L1PA3 | TRUE | NA | 0 | NA | FALSE | FALSE |
| chr5 | 66100440 | 66100451 | 4 | 3 | MLT1H | TRUE | NA | 0 | 1 | TRUE | FALSE |
| chr5 | 68485334 | 68485355 | 5 | 5 | NA | FALSE | MANY | 24 | 1 | TRUE | TRUE |
| chr5 | 68612968 | 68613027 | 7 | 4 | AluSg4 | TRUE | NA | 0 | NA | FALSE | FALSE |
| chr5 | 70821165 | 70821283 | 5 | 4 | SVA D | TRUE | NA | 0 | NA | FALSE | FALSE |
| chr5 | 70821470 | 70821527 | 7 | 5 | SVA D | TRUE | NA | 0 | NA | FALSE | FALSE |
| chr5 | 71222048 | 71222092 | 4 | 3 | MER20 | TRUE | NA | 0 | 1 | TRUE | FALSE |
| chr5 | 72643997 | 72644052 | 4 | 1 | Ricksha c | TRUE | NA | 0 | 1 | FALSE | FALSE |
| chr5 | 73695613 | 73695651 | 4 | 3 | MamGypLTR1c | TRUE | NA | 0 | 1 | FALSE | FALSE |
| chr5 | 73956854 | 73956856 | 4 | 3 | (GA)n | TRUE | NA | 0 | NA | TRUE | FALSE |
| chr5 | 74101976 | 74102013 | 4 | 2 | NA | FALSE | NA | 0 | 1 | FALSE | FALSE |
| chr5 | 75280389 | 75280395 | 6 | 3 | (TCTA)n | TRUE | NA | 0 | NA | FALSE | FALSE |
| chr5 | 75658792 | 75658823 | 4 | 2 | LTR7 | TRUE | NA | 0 | NA | FALSE | FALSE |
| chr5 | 76332462 | 76332504 | 4 | 4 | NA | FALSE | NA | 0 | 1 | FALSE | FALSE |
| chr5 | 76338354 | 76338424 | 5 | 5 | SVA F | TRUE | NA | 0 | NA | FALSE | FALSE |
| chr5 | 76339319 | 76339367 | 5 | 5 | SVA F | TRUE | NA | 0 | NA | FALSE | FALSE |
| chr5 | 76787254 | 76787254 | 4 | 4 | LTR7 | TRUE | NA | 0 | NA | FALSE | FALSE |
| chr5 | 77090793 | 77090848 | 4 | 4 | SVA F | TRUE | NA | 0 | NA | FALSE | FALSE |
| chr5 | 77293642 | 77293697 | 6 | 3 | AluSx1 | TRUE | NA | 0 | NA | FALSE | FALSE |
| chr5 | 77620263 | 77620326 | 4 | 3 | L2 | TRUE | NA | 0 | 1 | FALSE | FALSE |
| chr5 | 77735325 | 77735326 | 6 | 3 | L1PA6 | TRUE | NA | 0 | NA | FALSE | FALSE |
| chr5 | 79268260 | 79268262 | 4 | 2 | NA | FALSE | NA | 0 | 1 | FALSE | FALSE |
| chr5 | 79322038 | 79322086 | 4 | 4 | NA | FALSE | NA | 0 | 1 | FALSE | FALSE |
| chr5 | 80232420 | 80232491 | 4 | 2 | AluSx | TRUE | NA | 0 | NA | FALSE | FALSE |
| chr5 | 81344246 | 81344266 | 5 | 3 | L1PA3 | TRUE | NA | 0 | NA | FALSE | FALSE |
| chr5 | 81735504 | 81735504 | 4 | 4 | AluSg7 | TRUE | NA | 0 | 1 | FALSE | FALSE |
| chr5 | 82628086 | 82628090 | 6 | 5 | (CA)n | TRUE | NA | 0 | NA | FALSE | FALSE |
| chr5 | 82733327 | 82733396 | 10 | 7 | AluSc8 | TRUE | NA | 0 | NA | FALSE | FALSE |
| chr5 | 83784944 | 83784954 | 4 | 4 | MER63B | TRUE | NA | 0 | 1 | FALSE | FALSE |
| chr5 | 84461418 | 84461455 | 7 | 6 | LTR17 | TRUE | NA | 0 | NA | FALSE | FALSE |
| chr5 | 84728287 | 84728311 | 4 | 4 | L1PA5 | TRUE | NA | 0 | NA | FALSE | FALSE |
| chr5 | 85475696 | 85475719 | 4 | 3 | NA | FALSE | NA | 0 | 1 | FALSE | FALSE |
| chr5 | 85527749 | 85527777 | 4 | 2 | L1ME2z | TRUE | NA | 0 | 1 | FALSE | FALSE |
| chr5 | 86861167 | 86861167 | 4 | 4 | L1PA7 | TRUE | NA | 0 | 1 | FALSE | FALSE |
| chr5 | 86869518 | 86869555 | 6 | 6 | MLT1J2-int | TRUE | NA | 0 | 1 | FALSE | FALSE |
| chr5 | 86896365 | 86896385 | 4 | 4 | L1PREC2 | TRUE | NA | 0 | 1 | TRUE | FALSE |
| chr5 | 87103757 | 87103779 | 4 | 4 | L1MD3 | TRUE | PU.1 | 1 | 1 | FALSE | FALSE |
| chr5 | 87858963 | 87858984 | 4 | 4 | NA | FALSE | ZNF263 | 1 | 1 | TRUE | FALSE |
| chr5 | 87907405 | 87907447 | 4 | 3 | L1MDa | TRUE | NA | 0 | 1 | TRUE | FALSE |
| chr5 | 88441819 | 88441860 | 4 | 4 | L1MC3 | TRUE | NA | 0 | 1 | FALSE | FALSE |
| chr5 | 89445650 | 89445705 | 6 | 3 | L1PA5 | TRUE | NA | 0 | NA | FALSE | FALSE |
| chr5 | 89705710 | 89705745 | 4 | 4 | NA | FALSE | MANY | 12 | 1 | TRUE | TRUE |
| chr5 | 90349887 | 90349922 | 4 | 4 | MANY | TRUE | ZZZ3 | 1 | NA | TRUE | FALSE |
| chr5 | 90979467 | 90979496 | 4 | 3 | NA | FALSE | NA | 0 | 1 | TRUE | FALSE |
| chr5 | 91308144 | 91308172 | 4 | 4 | THE1D-int | TRUE | NA | 0 | 1 | FALSE | FALSE |
| chr5 | 92435139 | 92435141 | 6 | 4 | (TG)n | TRUE | NA | 0 | NA | FALSE | FALSE |
| chr5 | 92823414 | 92823458 | 6 | 3 | L1PA7 | TRUE | NA | 0 | NA | FALSE | FALSE |
| chr5 | 92918491 | 92918493 | 4 | 3 | (TG)n | TRUE | MANY | 14 | NA | TRUE | FALSE |
| chr5 | 93028916 | 93028931 | 4 | 2 | L1PA7 | TRUE | NA | 0 | NA | FALSE | FALSE |
| chr5 | 93101601 | 93101661 | 4 | 3 | L1PA4 | TRUE | NA | 0 | NA | FALSE | FALSE |
| chr5 | 93577875 | 93577922 | 4 | 2 | NA | FALSE | NA | 0 | 1 | FALSE | FALSE |
| chr5 | 94484278 | 94484297 | 4 | 4 | L1PA16 | TRUE | NA | 0 | 1 | TRUE | FALSE |
| chr5 | 94537144 | 94537159 | 5 | 5 | NA | FALSE | NA | 0 | 1 | FALSE | FALSE |
| chr5 | 94675525 | 94675537 | 5 | 3 | L1P2 | TRUE | NA | 0 | NA | FALSE | FALSE |
| chr5 | 95043403 | 95043446 | 9 | 4 | LTR6B | TRUE | TAL1 (SC-12984) | 1 | 1 | TRUE | FALSE |
| chr5 | 95701518 | 95701533 | 4 | 2 | L1PA5 | TRUE | NA | 0 | NA | FALSE | FALSE |
| chr5 | 95846941 | 95846971 | 6 | 6 | AluSc | TRUE | NA | 0 | NA | FALSE | FALSE |
| chr5 | 96719335 | 96719351 | 4 | 2 | L1PA3 | TRUE | NA | 0 | NA | FALSE | FALSE |
| chr5 | 97229636 | 97229652 | 5 | 4 | AluY | TRUE | NA | 0 | NA | FALSE | FALSE |
| chr5 | 97619628 | 97619673 | 4 | 4 | MANY | TRUE | NA | 0 | 1 | FALSE | FALSE |
| chr5 | 97724292 | 97724331 | 4 | 4 | NA | FALSE | NA | 0 | 1 | FALSE | FALSE |
| chr5 | 98712455 | 98712474 | 4 | 4 | NA | FALSE | NA | 0 | 1 | FALSE | FALSE |
| chr5 | 99043887 | 99043917 | 4 | 4 | THE1B-int | TRUE | NA | 0 | 1 | FALSE | FALSE |
| chr5 | 99115250 | 99115264 | 4 | 4 | NA | FALSE | NA | 0 | 1 | FALSE | FALSE |
| chr5 | 99364072 | 99364098 | 4 | 3 | L1MA3 | TRUE | NA | 0 | 1 | FALSE | FALSE |
| chr5 | 99409795 | 99409807 | 7 | 7 | L1MC4 | TRUE | NA | 0 | NA | FALSE | FALSE |
| chr5 | 99581446 | 99581477 | 4 | 3 | NA | FALSE | NA | 0 | 1 | FALSE | FALSE |
| chr5 | 99803113 | 99803147 | 4 | 4 | NA | FALSE | NA | 0 | 1 | FALSE | FALSE |
| chr5 | 99929695 | 99929706 | 4 | 3 | (TA)n | TRUE | NA | 0 | NA | FALSE | FALSE |
| chr5 | 100568769 | 100568788 | 6 | 5 | L1PA8 | TRUE | NA | 0 | 1 | FALSE | FALSE |
| chr5 | 100588384 | 100588396 | 4 | 4 | NA | FALSE | NA | 0 | 1 | FALSE | FALSE |
| chr5 | 100821317 | 100821320 | 4 | 3 | ERV3-16A3 I-int | TRUE | NA | 0 | 1 | FALSE | FALSE |
| chr5 | 101030452 | 101030482 | 4 | 3 | AluSz | TRUE | NA | 0 | 1 | FALSE | FALSE |
| chr5 | 101682496 | 101682513 | 4 | 3 | L1PBb | TRUE | NA | 0 | 1 | TRUE | FALSE |
| chr5 | 101854180 | 101854208 | 4 | 3 | NA | FALSE | NA | 0 | 1 | FALSE | FALSE |
| chr5 | 101952130 | 101952136 | 4 | 2 | NA | FALSE | NA | 0 | 1 | FALSE | FALSE |
| chr5 | 103020459 | 103020482 | 4 | 4 | NA | FALSE | NA | 0 | 1 | FALSE | FALSE |
| chr5 | 103322479 | 103322513 | 4 | 3 | NA | FALSE | NA | 0 | 1 | FALSE | FALSE |
| chr5 | 104164578 | 104164607 | 6 | 2 | L1PA6 | TRUE | NA | 0 | NA | FALSE | FALSE |
| chr5 | 104330211 | 104330225 | 4 | 3 | L2a | TRUE | NA | 0 | 1 | FALSE | FALSE |
| chr5 | 106315384 | 106315402 | 4 | 2 | HERVH-int | TRUE | NA | 0 | 1 | FALSE | FALSE |
| chr5 | 107234553 | 107234608 | 4 | 3 | L1PA7 | TRUE | NA | 0 | NA | FALSE | FALSE |
| chr5 | 107894851 | 107894879 | 4 | 3 | SVA D | TRUE | NA | 0 | NA | FALSE | FALSE |
| chr5 | 108191216 | 108191235 | 4 | 3 | AluYc | TRUE | NA | 0 | NA | FALSE | FALSE |
| chr5 | 108689771 | 108689795 | 4 | 4 | NA | FALSE | NA | 0 | 1 | FALSE | FALSE |
| chr5 | 108888053 | 108888110 | 4 | 3 | MANY | TRUE | NA | 0 | 1 | TRUE | FALSE |
| chr5 | 108918138 | 108918152 | 4 | 4 | Tigger2 | TRUE | NA | 0 | 1 | TRUE | FALSE |
| chr5 | 109375915 | 109375915 | 4 | 4 | L1PA11 | TRUE | CTCF | 1 | 1 | FALSE | FALSE |
| chr5 | 109527433 | 109527470 | 4 | 2 | AluY | TRUE | NA | 0 | NA | FALSE | FALSE |
| chr5 | 110074660 | 110074686 | 4 | 4 | NA | FALSE | MANY | 32 | 1 | TRUE | TRUE |
| chr5 | 110160119 | 110160136 | 4 | 4 | MER52-int | TRUE | NA | 0 | 1 | FALSE | FALSE |
| chr5 | 110208529 | 110208544 | 4 | 4 | NA | FALSE | NA | 0 | 1 | FALSE | FALSE |
| chr5 | 110271242 | 110271263 | 4 | 4 | L1PA4 | TRUE | NA | 0 | NA | FALSE | FALSE |
| chr5 | 111267048 | 111267055 | 4 | 2 | MLT1F1 | TRUE | NA | 0 | 1 | TRUE | FALSE |
| chr5 | 111324146 | 111324166 | 5 | 3 | L1PA3 | TRUE | NA | 0 | NA | FALSE | FALSE |
| chr5 | 111386615 | 111386622 | 4 | 2 | L1PA14 | TRUE | NA | 0 | 1 | TRUE | FALSE |
| chr5 | 111548796 | 111548810 | 4 | 4 | NA | FALSE | NA | 0 | 1 | FALSE | FALSE |
| chr5 | 111895276 | 111895311 | 4 | 3 | MER50 | TRUE | NA | 0 | NA | TRUE | FALSE |
| chr5 | 113041134 | 113041150 | 4 | 3 | MSTA-int | TRUE | NA | 0 | 1 | FALSE | FALSE |
| chr5 | 113223739 | 113223752 | 4 | 4 | NA | FALSE | NA | 0 | 1 | FALSE | FALSE |
| chr5 | 113230564 | 113230583 | 4 | 3 | L1M5 | TRUE | NA | 0 | 1 | FALSE | FALSE |
| chr5 | 113362522 | 113362541 | 4 | 2 | L1PA7 | TRUE | NA | 0 | 1 | FALSE | FALSE |
| chr5 | 114036263 | 114036279 | 4 | 3 | NA | FALSE | NA | 0 | 1 | FALSE | FALSE |
| chr5 | 114189384 | 114189413 | 4 | 3 | L1PB1 | TRUE | NA | 0 | NA | FALSE | FALSE |
| chr5 | 117618603 | 117618644 | 7 | 5 | SVA C | TRUE | NA | 0 | NA | FALSE | FALSE |
| chr5 | 117657439 | 117657484 | 4 | 4 | THE1D | TRUE | NA | 0 | NA | FALSE | FALSE |
| chr5 | 119123284 | 119123308 | 4 | 3 | NA | FALSE | NA | 0 | 1 | FALSE | FALSE |
| chr5 | 119315021 | 119315027 | 5 | 2 | L1PA17 | TRUE | NA | 0 | 1 | FALSE | FALSE |
| chr5 | 119315534 | 119315550 | 4 | 3 | L2a | TRUE | NA | 0 | 1 | FALSE | FALSE |
| chr5 | 119384526 | 119384536 | 4 | 2 | (TATATG)n | TRUE | NA | 0 | 1 | FALSE | FALSE |
| chr5 | 120984131 | 120984198 | 5 | 4 | L1PA6 | TRUE | NA | 0 | NA | FALSE | FALSE |
| chr5 | 121689564 | 121689584 | 4 | 4 | NA | FALSE | NA | 0 | 1 | FALSE | FALSE |
| chr5 | 121843832 | 121843845 | 4 | 3 | L2a | TRUE | NA | 0 | 1 | FALSE | FALSE |
| chr5 | 122249885 | 122249886 | 4 | 2 | L1PA3 | TRUE | NA | 0 | 1 | FALSE | FALSE |
| chr5 | 122577028 | 122577045 | 4 | 4 | L1PA2 | TRUE | NA | 0 | NA | FALSE | FALSE |
| chr5 | 122626672 | 122626709 | 5 | 4 | LTR16A | TRUE | NA | 0 | 1 | FALSE | FALSE |
| chr5 | 123367726 | 123367788 | 4 | 3 | NA | FALSE | NA | 0 | 1 | FALSE | FALSE |
| chr5 | 123644583 | 123644657 | 5 | 3 | HERVH-int | TRUE | NA | 0 | NA | FALSE | FALSE |
| chr5 | 124304991 | 124304996 | 4 | 2 | HERVH-int | TRUE | NA | 0 | NA | TRUE | FALSE |
| chr5 | 124396497 | 124396562 | 4 | 2 | (TATATG)n | TRUE | NA | 0 | 1 | FALSE | FALSE |
| chr5 | 125936586 | 125936599 | 4 | 3 | NA | FALSE | MANY | 37 | 1 | TRUE | TRUE |
| chr5 | 126179731 | 126179830 | 7 | 4 | HERVH-int | TRUE | NA | 0 | NA | FALSE | FALSE |
| chr5 | 126336476 | 126336547 | 4 | 2 | AluY | TRUE | NA | 0 | NA | FALSE | FALSE |
| chr5 | 127362828 | 127362888 | 4 | 2 | NA | FALSE | NA | 0 | 1 | FALSE | FALSE |
| chr5 | 128222466 | 128222490 | 5 | 5 | L1M4c | TRUE | NA | 0 | 1 | FALSE | FALSE |
| chr5 | 129932490 | 129932546 | 8 | 5 | (TA)n | TRUE | NA | 0 | NA | FALSE | FALSE |
| chr5 | 130082817 | 130082846 | 4 | 3 | THE1A-int | TRUE | NA | 0 | 1 | FALSE | FALSE |
| chr5 | 130393520 | 130393550 | 5 | 4 | L1MA9 | TRUE | NA | 0 | 1 | FALSE | FALSE |
| chr5 | 131444264 | 131444308 | 4 | 2 | L1PA4 | TRUE | NA | 0 | NA | FALSE | FALSE |
| chr5 | 132193384 | 132193426 | 10 | 5 | AluSc8 | TRUE | NA | 0 | NA | FALSE | FALSE |
| chr5 | 132252830 | 132252859 | 5 | 5 | AluSp | TRUE | NA | 0 | NA | FALSE | FALSE |
| chr5 | 132310248 | 132310304 | 4 | 3 | AluSc | TRUE | NA | 0 | NA | TRUE | FALSE |
| chr5 | 132926877 | 132926929 | 4 | 3 | NA | FALSE | NA | 0 | 1 | TRUE | FALSE |
| chr5 | 133272095 | 133272129 | 4 | 3 | NA | FALSE | ZNF263 | 1 | 1 | FALSE | FALSE |
| chr5 | 133492907 | 133492915 | 4 | 3 | NA | FALSE | NA | 0 | 1 | TRUE | FALSE |
| chr5 | 133620696 | 133620741 | 8 | 6 | MER11C | TRUE | NA | 0 | NA | FALSE | FALSE |
| chr5 | 133702908 | 133702909 | 7 | 5 | NA | FALSE | MANY | 43 | 1 | TRUE | TRUE |
| chr5 | 134448638 | 134448693 | 4 | 4 | AluY | TRUE | NA | 0 | NA | FALSE | FALSE |
| chr5 | 134623407 | 134623452 | 4 | 4 | L1MA9 | TRUE | SETDB1 | 1 | 1 | FALSE | FALSE |
| chr5 | 135951108 | 135951127 | 4 | 3 | NA | FALSE | MANY | 2 | 1 | TRUE | FALSE |
| chr5 | 136517546 | 136517564 | 4 | 3 | NA | FALSE | MANY | 3 | 1 | TRUE | TRUE |
| chr5 | 137549124 | 137549195 | 4 | 4 | NA | FALSE | MANY | 24 | 1 | TRUE | TRUE |
| chr5 | 137800743 | 137800840 | 8 | 6 | NA | FALSE | MANY | 15 | 1 | TRUE | TRUE |
| chr5 | 137878969 | 137878970 | 5 | 4 | NA | FALSE | MANY | 30 | 1 | TRUE | TRUE |
| chr5 | 139043970 | 139044019 | 4 | 2 | NA | FALSE | MANY | 6 | 1 | TRUE | TRUE |
| chr5 | 139313006 | 139313077 | 4 | 3 | L1M5 | TRUE | NA | 0 | 1 | FALSE | FALSE |
| chr5 | 139395093 | 139395133 | 4 | 3 | L2c | TRUE | NA | 0 | 1 | FALSE | FALSE |
| chr5 | 139443941 | 139443993 | 4 | 3 | NA | FALSE | NA | 0 | 1 | FALSE | FALSE |
| chr5 | 139895181 | 139895213 | 4 | 3 | L1PA3 | TRUE | NA | 0 | 1 | FALSE | FALSE |
| chr5 | 140052406 | 140052429 | 6 | 3 | NA | FALSE | NA | 0 | 1 | TRUE | FALSE |
| chr5 | 140196806 | 140196843 | 4 | 4 | L1M3d | TRUE | NA | 0 | 1 | TRUE | FALSE |
| chr5 | 141454816 | 141454920 | 8 | 5 | SVA F | TRUE | NA | 0 | NA | FALSE | FALSE |
| chr5 | 141455200 | 141455349 | 8 | 7 | SVA F | TRUE | NA | 0 | 1 | FALSE | FALSE |
| chr5 | 141582508 | 141582545 | 5 | 5 | (GA)n | TRUE | CTCF | 1 | 1 | TRUE | FALSE |
| chr5 | 141771596 | 141771664 | 4 | 3 | (TTCC)n | TRUE | NA | 0 | 1 | FALSE | FALSE |
| chr5 | 141975989 | 141976054 | 4 | 4 | MANY | TRUE | NA | 0 | 1 | FALSE | FALSE |
| chr5 | 142369275 | 142369351 | 6 | 4 | L1PA5 | TRUE | NA | 0 | 1 | FALSE | FALSE |
| chr5 | 142930315 | 142930315 | 4 | 4 | NA | FALSE | NA | 0 | NA | FALSE | FALSE |
| chr5 | 142976606 | 142976657 | 5 | 4 | NA | FALSE | NA | 0 | 1 | FALSE | FALSE |
| chr5 | 143651733 | 143651752 | 4 | 3 | AluSq2 | TRUE | NA | 0 | NA | FALSE | FALSE |
| chr5 | 143842553 | 143842558 | 4 | 2 | NA | FALSE | NA | 0 | 1 | FALSE | FALSE |
| chr5 | 144088682 | 144088694 | 5 | 3 | LTR12 | TRUE | NF-YA | 1 | NA | FALSE | FALSE |
| chr5 | 144495827 | 144495851 | 5 | 4 | (TC)n | TRUE | NA | 0 | NA | FALSE | FALSE |
| chr5 | 145332313 | 145332350 | 4 | 3 | LTR16C | TRUE | NA | 0 | 1 | FALSE | FALSE |
| chr5 | 145562310 | 145562356 | 7 | 6 | NA | FALSE | MANY | 45 | 1 | TRUE | TRUE |
| chr5 | 146931979 | 146931979 | 4 | 4 | AluY | TRUE | NA | 0 | NA | FALSE | FALSE |
| chr5 | 146958841 | 146958875 | 5 | 2 | AluSp | TRUE | NA | 0 | NA | FALSE | FALSE |
| chr5 | 147035285 | 147035304 | 4 | 2 | L1PA8 | TRUE | NA | 0 | NA | FALSE | FALSE |
| chr5 | 147158354 | 147158382 | 4 | 2 | NA | FALSE | NA | 0 | 1 | FALSE | FALSE |
| chr5 | 147251193 | 147251216 | 4 | 2 | HERVH-int | TRUE | NA | 0 | 1 | FALSE | FALSE |
| chr5 | 147955627 | 147955660 | 4 | 4 | AluYc | TRUE | NA | 0 | NA | FALSE | FALSE |
| chr5 | 148130216 | 148130228 | 4 | 3 | NA | FALSE | NA | 0 | 1 | FALSE | FALSE |
| chr5 | 148813434 | 148813470 | 4 | 3 | NA | FALSE | NA | 0 | 1 | TRUE | FALSE |
| chr5 | 149257782 | 149257844 | 4 | 1 | Charlie2b | TRUE | ERalpha a | 1 | 1 | TRUE | FALSE |
| chr5 | 149263862 | 149263898 | 4 | 2 | (TA)n | TRUE | NA | 0 | NA | FALSE | FALSE |
| chr5 | 149746641 | 149746718 | 4 | 4 | AluY | TRUE | NA | 0 | NA | FALSE | FALSE |
| chr5 | 149829297 | 149829353 | 4 | 4 | NA | FALSE | MANY | 36 | 1 | TRUE | TRUE |
| chr5 | 150764547 | 150764599 | 6 | 6 | MANY | TRUE | MANY | 2 | NA | TRUE | FALSE |
| chr5 | 150829503 | 150829557 | 4 | 2 | NA | FALSE | NA | 0 | 1 | TRUE | FALSE |
| chr5 | 151554868 | 151554883 | 4 | 4 | L1PA7 | TRUE | NA | 0 | NA | FALSE | FALSE |
| chr5 | 151992787 | 151992808 | 4 | 1 | MLT1G3 | TRUE | NA | 0 | 1 | FALSE | FALSE |
| chr5 | 152426950 | 152426961 | 4 | 3 | THE1B | TRUE | NA | 0 | NA | FALSE | FALSE |
| chr5 | 152970349 | 152970368 | 4 | 4 | NA | FALSE | NA | 0 | 1 | FALSE | FALSE |
| chr5 | 153289481 | 153289514 | 4 | 3 | NA | FALSE | NA | 0 | 1 | FALSE | FALSE |
| chr5 | 153297634 | 153297634 | 4 | 4 | Charlie7 | TRUE | NA | 0 | 1 | FALSE | FALSE |
| chr5 | 153299499 | 153299531 | 4 | 4 | NA | FALSE | NA | 0 | 1 | FALSE | FALSE |
| chr5 | 153553297 | 153553301 | 6 | 5 | MLT1K | TRUE | NA | 0 | NA | TRUE | FALSE |
| chr5 | 153928450 | 153928556 | 5 | 4 | AluJo | TRUE | NA | 0 | NA | FALSE | FALSE |
| chr5 | 154571676 | 154571682 | 4 | 3 | LTR81B | TRUE | NA | 0 | 1 | FALSE | FALSE |
| chr5 | 154743386 | 154743390 | 4 | 2 | NA | FALSE | NA | 0 | 1 | FALSE | FALSE |
| chr5 | 155924653 | 155924655 | 4 | 4 | (TC)n | TRUE | NA | 0 | NA | FALSE | FALSE |
| chr5 | 156754751 | 156754771 | 4 | 3 | NA | FALSE | NA | 0 | 1 | FALSE | FALSE |
| chr5 | 157082393 | 157082439 | 4 | 3 | PABL A-int | TRUE | NA | 0 | 1 | FALSE | FALSE |
| chr5 | 157772278 | 157772300 | 4 | 3 | L1MC4a | TRUE | NA | 0 | 1 | FALSE | FALSE |
| chr5 | 158514470 | 158514476 | 7 | 4 | (CA)n | TRUE | NA | 0 | NA | FALSE | FALSE |
| chr5 | 159308315 | 159308321 | 5 | 5 | (TC)n | TRUE | NA | 0 | NA | FALSE | FALSE |
| chr5 | 159409816 | 159409873 | 5 | 4 | MamGypLTR1d | TRUE | STAT3 | 1 | 1 | TRUE | FALSE |
| chr5 | 159925681 | 159925725 | 6 | 6 | NA | FALSE | NA | 0 | NA | FALSE | FALSE |
| chr5 | 161063479 | 161063496 | 4 | 3 | NA | FALSE | NA | 0 | 1 | FALSE | FALSE |
| chr5 | 161178795 | 161178798 | 4 | 3 | NA | FALSE | NA | 0 | NA | FALSE | FALSE |
| chr5 | 161196012 | 161196014 | 4 | 3 | L1PA4 | TRUE | NA | 0 | NA | FALSE | FALSE |
| chr5 | 161448505 | 161448516 | 4 | 3 | L1M1 | TRUE | NA | 0 | 1 | FALSE | FALSE |
| chr5 | 161890740 | 161890761 | 4 | 4 | L1PA4 | TRUE | NA | 0 | NA | FALSE | FALSE |
| chr5 | 162237047 | 162237047 | 4 | 4 | MER11B | TRUE | NA | 0 | NA | FALSE | FALSE |
| chr5 | 162307391 | 162307414 | 4 | 3 | LOR1-int | TRUE | NA | 0 | 1 | FALSE | FALSE |
| chr5 | 162701977 | 162702007 | 4 | 3 | NA | FALSE | NA | 0 | 1 | FALSE | FALSE |
| chr5 | 162739444 | 162739471 | 4 | 3 | MLT2A2 | TRUE | NA | 0 | 1 | FALSE | FALSE |
| chr5 | 162740390 | 162740413 | 5 | 3 | LTR2B | TRUE | NA | 0 | NA | FALSE | FALSE |
| chr5 | 163075968 | 163075982 | 4 | 4 | L1MB7 | TRUE | NA | 0 | 1 | FALSE | FALSE |
| chr5 | 163554109 | 163554156 | 5 | 3 | THE1A-int | TRUE | NA | 0 | 1 | FALSE | FALSE |
| chr5 | 164034983 | 164034983 | 4 | 4 | L1PB1 | TRUE | NA | 0 | NA | FALSE | FALSE |
| chr5 | 165880717 | 165880750 | 4 | 2 | L1MB8 | TRUE | NA | 0 | 1 | FALSE | FALSE |
| chr5 | 166091646 | 166091667 | 4 | 3 | NA | FALSE | NA | 0 | 1 | FALSE | FALSE |
| chr5 | 166778097 | 166778115 | 4 | 4 | (TA)n | TRUE | NA | 0 | NA | FALSE | FALSE |
| chr5 | 167495703 | 167495715 | 4 | 2 | NA | FALSE | NA | 0 | 1 | FALSE | FALSE |
| chr5 | 168178570 | 168178602 | 4 | 4 | MIR | TRUE | NA | 0 | 1 | TRUE | FALSE |
| chr5 | 168655649 | 168655649 | 4 | 4 | NA | FALSE | NA | 0 | 1 | FALSE | FALSE |
| chr5 | 168890848 | 168890870 | 4 | 4 | MLT1G3 | TRUE | NA | 0 | 1 | FALSE | FALSE |
| chr5 | 169452776 | 169452797 | 5 | 3 | (TTCC)n | TRUE | NA | 0 | NA | FALSE | FALSE |
| chr5 | 171433907 | 171433908 | 4 | 3 | NA | FALSE | MANY | 22 | 1 | TRUE | TRUE |
| chr5 | 172007264 | 172007312 | 4 | 2 | L2b | TRUE | NA | 0 | 1 | FALSE | FALSE |
| chr5 | 172078048 | 172078098 | 5 | 2 | LTR10F | TRUE | NA | 0 | 1 | FALSE | FALSE |
| chr5 | 172190204 | 172190213 | 4 | 2 | NA | FALSE | NA | 0 | 1 | TRUE | FALSE |
| chr5 | 172499115 | 172499115 | 4 | 4 | AluJb | TRUE | NA | 0 | NA | TRUE | FALSE |
| chr5 | 172990194 | 172990251 | 4 | 3 | L2a | TRUE | NA | 0 | 1 | FALSE | FALSE |
| chr5 | 173495882 | 173495887 | 5 | 5 | NA | FALSE | NA | 0 | 1 | FALSE | FALSE |
| chr5 | 173532784 | 173532802 | 4 | 2 | AluSx | TRUE | NA | 0 | NA | FALSE | FALSE |
| chr5 | 174151455 | 174151458 | 4 | 4 | NA | FALSE | MANY | 3 | 1 | TRUE | TRUE |
| chr5 | 174488770 | 174488786 | 4 | 2 | AluSx1 | TRUE | NA | 0 | NA | FALSE | FALSE |
| chr5 | 174659249 | 174659249 | 4 | 4 | (GGA)n | TRUE | NA | 0 | NA | FALSE | FALSE |
| chr5 | 175483831 | 175483836 | 5 | 3 | L1PA5 | TRUE | NA | 0 | NA | FALSE | FALSE |
| chr5 | 175571880 | 175571900 | 4 | 3 | L4 | TRUE | NA | 0 | 1 | FALSE | FALSE |
| chr5 | 175713091 | 175713096 | 4 | 4 | L1PA5 | TRUE | NA | 0 | NA | FALSE | FALSE |
| chr5 | 175713429 | 175713435 | 4 | 3 | L1PA5 | TRUE | NA | 0 | 1 | FALSE | FALSE |
| chr5 | 176286800 | 176286817 | 4 | 4 | NA | FALSE | NA | 0 | 1 | FALSE | FALSE |
| chr5 | 176299873 | 176299889 | 4 | 3 | NA | FALSE | NA | 0 | NA | FALSE | FALSE |
| chr5 | 176523869 | 176523928 | 4 | 4 | (TCC)n | TRUE | ZNF263 | 1 | NA | TRUE | FALSE |
| chr5 | 176768656 | 176768704 | 4 | 1 | L1MB4 | TRUE | NA | 0 | 1 | FALSE | FALSE |
| chr5 | 177469541 | 177469563 | 4 | 2 | AluSp | TRUE | NA | 0 | NA | FALSE | FALSE |
| chr5 | 177626550 | 177626564 | 4 | 3 | AluY | TRUE | NA | 0 | NA | FALSE | FALSE |
| chr5 | 178437148 | 178437159 | 4 | 3 | L1ME3E | TRUE | NA | 0 | 1 | FALSE | FALSE |
| chr5 | 178440276 | 178440316 | 4 | 3 | LTR37A | TRUE | NA | 0 | 1 | FALSE | FALSE |
| chr5 | 178740863 | 178740876 | 4 | 4 | NA | FALSE | NA | 0 | NA | FALSE | FALSE |
| chr5 | 178881312 | 178881364 | 4 | 2 | L2a | TRUE | NA | 0 | 1 | FALSE | FALSE |
| chr5 | 178944722 | 178944736 | 5 | 3 | NA | FALSE | NA | 0 | 1 | FALSE | FALSE |
| chr5 | 178979124 | 178979138 | 6 | 3 | AluSc8 | TRUE | NA | 0 | NA | FALSE | FALSE |
| chr5 | 179299086 | 179299139 | 4 | 3 | NA | FALSE | NA | 0 | NA | TRUE | FALSE |
| chr5 | 179364106 | 179364154 | 4 | 3 | L1MB5 | TRUE | NA | 0 | NA | FALSE | FALSE |
| chr5 | 179539980 | 179540043 | 6 | 5 | (CGGA)n | TRUE | NA | 0 | NA | TRUE | FALSE |
| chr5 | 180042637 | 180042639 | 4 | 4 | NA | FALSE | NA | 0 | NA | FALSE | FALSE |
| chr5 | 180182041 | 180182091 | 4 | 4 | L1M2a | TRUE | NA | 0 | 1 | FALSE | FALSE |
| chr5 | 180198293 | 180198333 | 6 | 4 | SVA D | TRUE | NA | 0 | NA | FALSE | FALSE |
| chr5 | 180254326 | 180254341 | 4 | 2 | LTR5 Hs | TRUE | NA | 0 | 1 | FALSE | FALSE |
| chr5 | 180588146 | 180588166 | 5 | 3 | (CTGGGG)n | TRUE | Pol2 | 1 | NA | FALSE | FALSE |
| chr5 | 180629667 | 180629694 | 6 | 4 | AluY | TRUE | NA | 0 | NA | FALSE | FALSE |
| chr5 | 180700597 | 180700686 | 4 | 2 | NA | FALSE | MANY | 3 | 1 | TRUE | TRUE |
| chr5 | 180703323 | 180703394 | 5 | 5 | AluSx1 | TRUE | NA | 0 | NA | FALSE | FALSE |
| chr5 | 180709849 | 180709886 | 4 | 3 | NA | FALSE | NA | 0 | NA | TRUE | FALSE |
| chr5 | 180730610 | 180730735 | 8 | 6 | AluJo | TRUE | NA | 0 | NA | FALSE | FALSE |
| chr6 | 267472 | 267535 | 4 | 3 | L1MC3 | TRUE | NA | 0 | 1 | TRUE | FALSE |
| chr6 | 281717 | 281770 | 4 | 4 | L2c | TRUE | NA | 0 | 1 | TRUE | FALSE |
| chr6 | 317283 | 317330 | 4 | 4 | NA | FALSE | MANY | 7 | 1 | TRUE | TRUE |
| chr6 | 675904 | 675954 | 4 | 2 | NA | FALSE | NA | 0 | NA | FALSE | FALSE |
| chr6 | 676653 | 676674 | 4 | 3 | NA | FALSE | NA | 0 | 1 | FALSE | FALSE |
| chr6 | 676824 | 676903 | 10 | 8 | NA | FALSE | NA | 0 | NA | FALSE | FALSE |
| chr6 | 1005320 | 1005324 | 7 | 5 | (CA)n | TRUE | MANY | 2 | NA | TRUE | FALSE |
| chr6 | 1478325 | 1478390 | 4 | 4 | (TGG)n | TRUE | NA | 0 | NA | FALSE | FALSE |
| chr6 | 1534993 | 1535049 | 4 | 4 | NA | FALSE | NA | 0 | NA | FALSE | FALSE |
| chr6 | 1932894 | 1932919 | 4 | 2 | AluY | TRUE | NA | 0 | NA | FALSE | FALSE |
| chr6 | 2466396 | 2466458 | 4 | 4 | L1ME4a | TRUE | NA | 0 | 1 | TRUE | FALSE |
| chr6 | 2907930 | 2907996 | 5 | 4 | (TG)n | TRUE | NA | 0 | NA | FALSE | FALSE |
| chr6 | 3094242 | 3094280 | 4 | 4 | NA | FALSE | NA | 0 | NA | FALSE | FALSE |
| chr6 | 3157800 | 3157855 | 6 | 5 | NA | FALSE | MANY | 44 | 1 | TRUE | TRUE |
| chr6 | 3259135 | 3259136 | 4 | 3 | NA | FALSE | MANY | 27 | 1 | TRUE | TRUE |
| chr6 | 3550458 | 3550507 | 7 | 7 | (TTC)n | TRUE | NA | 0 | NA | FALSE | FALSE |
| chr6 | 4199801 | 4199832 | 5 | 4 | L1PA4 | TRUE | NA | 0 | NA | FALSE | FALSE |
| chr6 | 4810066 | 4810134 | 4 | 3 | NA | FALSE | MANY | 2 | NA | FALSE | FALSE |
| chr6 | 4978743 | 4978784 | 5 | 5 | MANY | TRUE | NA | 0 | NA | FALSE | FALSE |
| chr6 | 5001145 | 5001187 | 7 | 7 | (CAAG)n | TRUE | NA | 0 | 1 | FALSE | FALSE |
| chr6 | 6753727 | 6753754 | 4 | 3 | MER21C | TRUE | NA | 0 | 1 | FALSE | FALSE |
| chr6 | 6806099 | 6806132 | 4 | 3 | LTR16E2 | TRUE | NA | 0 | 1 | TRUE | FALSE |
| chr6 | 7033821 | 7033852 | 4 | 2 | NA | FALSE | NA | 0 | 1 | FALSE | FALSE |
| chr6 | 8521973 | 8522027 | 4 | 2 | L1PA8A | TRUE | NA | 0 | NA | FALSE | FALSE |
| chr6 | 8828197 | 8828263 | 5 | 3 | HAL1-2a MD | TRUE | NA | 0 | NA | FALSE | FALSE |
| chr6 | 10787244 | 10787314 | 4 | 4 | NA | FALSE | NA | 0 | 1 | FALSE | FALSE |
| chr6 | 12287918 | 12287944 | 4 | 4 | (TC)n | TRUE | MANY | 2 | NA | TRUE | FALSE |
| chr6 | 12661622 | 12661650 | 4 | 3 | NA | FALSE | NA | 0 | 1 | FALSE | FALSE |
| chr6 | 12772116 | 12772138 | 4 | 4 | NA | FALSE | NA | 0 | 1 | FALSE | FALSE |
| chr6 | 12802816 | 12802824 | 4 | 3 | Kanga1 | TRUE | CEBPB | 1 | 1 | FALSE | FALSE |
| chr6 | 13541772 | 13541830 | 4 | 4 | LTR12C | TRUE | NA | 0 | 1 | TRUE | FALSE |
| chr6 | 15264870 | 15264885 | 5 | 5 | MANY | TRUE | NA | 0 | NA | TRUE | FALSE |
| chr6 | 15415882 | 15415946 | 5 | 5 | SVA D | TRUE | NA | 0 | NA | FALSE | FALSE |
| chr6 | 15743972 | 15744010 | 4 | 3 | L1ME4a | TRUE | NA | 0 | 1 | FALSE | FALSE |
| chr6 | 16063940 | 16064005 | 4 | 3 | GA-rich | TRUE | NA | 0 | 1 | TRUE | FALSE |
| chr6 | 16574367 | 16574415 | 4 | 2 | AluSx | TRUE | NA | 0 | NA | FALSE | FALSE |
| chr6 | 17197552 | 17197593 | 4 | 4 | MLT1H2 | TRUE | NA | 0 | 1 | TRUE | FALSE |
| chr6 | 17329035 | 17329091 | 7 | 4 | AluSp | TRUE | NA | 0 | NA | FALSE | FALSE |
| chr6 | 17338801 | 17338843 | 4 | 4 | AluY | TRUE | NA | 0 | NA | FALSE | FALSE |
| chr6 | 17587130 | 17587130 | 4 | 4 | (CA)n | TRUE | NA | 0 | NA | FALSE | FALSE |
| chr6 | 18155421 | 18155458 | 4 | 4 | C-rich | TRUE | MANY | 9 | 1 | TRUE | TRUE |
| chr6 | 18327778 | 18327784 | 4 | 3 | (GA)n | TRUE | ZZZ3 | 1 | NA | FALSE | FALSE |
| chr6 | 19637216 | 19637253 | 4 | 4 | (GAAA)n | TRUE | NA | 0 | NA | FALSE | FALSE |
| chr6 | 19939998 | 19940051 | 14 | 11 | SVA D | TRUE | NA | 0 | NA | FALSE | FALSE |
| chr6 | 19940226 | 19940263 | 6 | 5 | SVA D | TRUE | NA | 0 | NA | FALSE | FALSE |
| chr6 | 19940723 | 19940741 | 5 | 4 | SVA D | TRUE | NA | 0 | NA | FALSE | FALSE |
| chr6 | 20401770 | 20401837 | 4 | 3 | GC rich | TRUE | MANY | 19 | 1 | TRUE | TRUE |
| chr6 | 21299418 | 21299441 | 7 | 6 | CT-rich | TRUE | NA | 0 | NA | FALSE | FALSE |
| chr6 | 21718670 | 21718704 | 5 | 5 | MANY | TRUE | NA | 0 | NA | FALSE | FALSE |
| chr6 | 21765117 | 21765144 | 5 | 4 | NA | FALSE | NA | 0 | 1 | TRUE | FALSE |
| chr6 | 21914187 | 21914245 | 4 | 4 | NA | FALSE | NA | 0 | 1 | TRUE | FALSE |
| chr6 | 21925115 | 21925149 | 7 | 6 | MANY | TRUE | TR4 | 1 | NA | FALSE | FALSE |
| chr6 | 22243336 | 22243336 | 4 | 4 | L1PB1 | TRUE | NA | 0 | 1 | FALSE | FALSE |
| chr6 | 24567420 | 24567421 | 6 | 4 | NA | FALSE | NA | 0 | 1 | FALSE | FALSE |
| chr6 | 24571718 | 24571770 | 4 | 3 | NA | FALSE | RXRA | 1 | 1 | TRUE | FALSE |
| chr6 | 24721411 | 24721439 | 6 | 5 | NA | FALSE | MANY | 22 | 1 | TRUE | TRUE |
| chr6 | 25809266 | 25809321 | 4 | 3 | L1PB4 | TRUE | NA | 0 | 1 | FALSE | FALSE |
| chr6 | 26521055 | 26521105 | 5 | 3 | (CCCCG)n | TRUE | MANY | 9 | 1 | TRUE | TRUE |
| chr6 | 26751653 | 26751691 | 4 | 4 | NA | FALSE | NA | 0 | 1 | TRUE | FALSE |
| chr6 | 26757054 | 26757062 | 8 | 5 | LTR5 Hs | TRUE | NA | 0 | NA | TRUE | FALSE |
| chr6 | 26761306 | 26761352 | 5 | 2 | (TATG)n | TRUE | NA | 0 | 1 | TRUE | FALSE |
| chr6 | 26799041 | 26799146 | 10 | 7 | (TATG)n | TRUE | NA | 0 | 1 | FALSE | FALSE |
| chr6 | 26859201 | 26859250 | 6 | 5 | AluSz | TRUE | NA | 0 | NA | FALSE | FALSE |
| chr6 | 27100690 | 27100711 | 4 | 4 | NA | FALSE | MANY | 26 | 1 | TRUE | TRUE |
| chr6 | 27236323 | 27236362 | 4 | 3 | NA | FALSE | NA | 0 | 1 | TRUE | FALSE |
| chr6 | 28048713 | 28048718 | 4 | 3 | NA | FALSE | MANY | 54 | 1 | TRUE | TRUE |
| chr6 | 29116080 | 29116100 | 4 | 2 | L1MC2 | TRUE | NA | 0 | 1 | FALSE | FALSE |
| chr6 | 29281976 | 29281997 | 4 | 3 | MANY | TRUE | NA | 0 | 1 | FALSE | FALSE |
| chr6 | 29379844 | 29379871 | 4 | 4 | L1PREC2 | TRUE | NA | 0 | 1 | FALSE | FALSE |
| chr6 | 29536164 | 29536206 | 6 | 5 | (TTC)n | TRUE | NA | 0 | NA | FALSE | FALSE |
| chr6 | 29758258 | 29758298 | 7 | 5 | MLT1E3 | TRUE | NA | 0 | 1 | FALSE | FALSE |
| chr6 | 29784325 | 29784329 | 4 | 2 | ERV3-16A3 I-int | TRUE | NA | 0 | 1 | FALSE | FALSE |
| chr6 | 29896456 | 29896466 | 4 | 2 | NA | FALSE | NA | 0 | NA | TRUE | FALSE |
| chr6 | 30226067 | 30226116 | 4 | 4 | NA | FALSE | MANY | 3 | 1 | TRUE | TRUE |
| chr6 | 30377441 | 30377461 | 4 | 4 | LTR10B | TRUE | NA | 0 | 1 | FALSE | FALSE |
| chr6 | 30483876 | 30483936 | 4 | 2 | NA | FALSE | MANY | 21 | 1 | TRUE | TRUE |
| chr6 | 30585199 | 30585221 | 5 | 5 | NA | FALSE | MANY | 29 | 1 | TRUE | TRUE |
| chr6 | 30640794 | 30640795 | 8 | 7 | NA | FALSE | MANY | 40 | 1 | TRUE | TRUE |
| chr6 | 30954522 | 30954533 | 4 | 2 | NA | FALSE | NA | 0 | 1 | FALSE | FALSE |
| chr6 | 31153643 | 31153647 | 4 | 2 | Harlequin-int | TRUE | BATF | 1 | 1 | TRUE | FALSE |
| chr6 | 31192215 | 31192228 | 8 | 4 | NA | FALSE | NA | 0 | 1 | TRUE | FALSE |
| chr6 | 31359320 | 31359320 | 4 | 4 | NA | FALSE | NA | 0 | 1 | TRUE | FALSE |
| chr6 | 31396444 | 31396481 | 5 | 3 | (TA)n | TRUE | NA | 0 | NA | FALSE | FALSE |
| chr6 | 31940081 | 31940122 | 4 | 4 | NA | FALSE | MANY | 29 | 1 | TRUE | TRUE |
| chr6 | 32038519 | 32038571 | 4 | 4 | NA | FALSE | NA | 0 | 1 | TRUE | FALSE |
| chr6 | 32054345 | 32054375 | 4 | 3 | NA | FALSE | NA | 0 | 1 | TRUE | FALSE |
| chr6 | 32352833 | 32352903 | 8 | 3 | SVA D | TRUE | NA | 0 | 1 | FALSE | FALSE |
| chr6 | 32449523 | 32449602 | 8 | 4 | NA | FALSE | NA | 0 | 1 | FALSE | FALSE |
| chr6 | 32548516 | 32548562 | 4 | 4 | NA | FALSE | Pol2-4H8 | 1 | 1 | TRUE | FALSE |
| chr6 | 32732681 | 32732722 | 4 | 4 | NA | FALSE | NA | 0 | 1 | FALSE | FALSE |
| chr6 | 33185015 | 33185045 | 4 | 3 | NA | FALSE | NA | 0 | 1 | FALSE | FALSE |
| chr6 | 33239777 | 33239846 | 8 | 5 | NA | FALSE | MANY | 53 | 1 | TRUE | TRUE |
| chr6 | 33461499 | 33461515 | 4 | 3 | (GAAA)n | TRUE | NA | 0 | NA | FALSE | FALSE |
| chr6 | 33747200 | 33747241 | 4 | 2 | NA | FALSE | MANY | 3 | 1 | TRUE | TRUE |
| chr6 | 33989810 | 33989819 | 7 | 5 | (CAGG)n | TRUE | NA | 0 | 1 | TRUE | FALSE |
| chr6 | 33999745 | 33999763 | 5 | 5 | (CA)n | TRUE | NA | 0 | NA | TRUE | FALSE |
| chr6 | 34634083 | 34634089 | 7 | 3 | AluSp | TRUE | NA | 0 | NA | FALSE | FALSE |
| chr6 | 34855823 | 34855824 | 4 | 4 | NA | FALSE | MANY | 58 | 1 | TRUE | TRUE |
| chr6 | 35156383 | 35156455 | 4 | 3 | AluY | TRUE | NA | 0 | NA | FALSE | FALSE |
| chr6 | 35660585 | 35660634 | 4 | 4 | AluSx | TRUE | NA | 0 | 1 | TRUE | FALSE |
| chr6 | 35978365 | 35978405 | 5 | 2 | AluSz | TRUE | NA | 0 | NA | FALSE | FALSE |
| chr6 | 36910984 | 36910994 | 4 | 3 | L1ME3E | TRUE | NA | 0 | NA | TRUE | FALSE |
| chr6 | 36999109 | 36999164 | 4 | 3 | AluJo | TRUE | NA | 0 | 1 | FALSE | FALSE |
| chr6 | 37237052 | 37237090 | 4 | 2 | MIRb | TRUE | NA | 0 | 1 | FALSE | FALSE |
| chr6 | 37692755 | 37692787 | 4 | 3 | L2c | TRUE | ELF1 (SC-631) | 1 | 1 | TRUE | FALSE |
| chr6 | 38764429 | 38764463 | 4 | 3 | LTR2 | TRUE | MANY | 11 | 1 | TRUE | TRUE |
| chr6 | 38792906 | 38792942 | 4 | 3 | NA | FALSE | NA | 0 | 1 | FALSE | FALSE |
| chr6 | 38909485 | 38909520 | 4 | 3 | NA | FALSE | NA | 0 | 1 | FALSE | FALSE |
| chr6 | 38915319 | 38915342 | 4 | 3 | NA | FALSE | NA | 0 | 1 | TRUE | FALSE |
| chr6 | 39052260 | 39052271 | 5 | 2 | NA | FALSE | NA | 0 | 1 | FALSE | FALSE |
| chr6 | 39693259 | 39693297 | 6 | 5 | NA | FALSE | MANY | 12 | 1 | TRUE | TRUE |
| chr6 | 40073451 | 40073478 | 5 | 4 | L1PA3 | TRUE | NA | 0 | NA | FALSE | FALSE |
| chr6 | 40102340 | 40102365 | 4 | 4 | NA | FALSE | NA | 0 | 1 | TRUE | FALSE |
| chr6 | 40147869 | 40147895 | 4 | 3 | NA | FALSE | NA | 0 | 1 | FALSE | FALSE |
| chr6 | 40177444 | 40177462 | 4 | 3 | (TA)n | TRUE | NA | 0 | NA | FALSE | FALSE |
| chr6 | 40942885 | 40942907 | 4 | 4 | L1PREC2 | TRUE | NA | 0 | 1 | FALSE | FALSE |
| chr6 | 41076861 | 41076865 | 6 | 6 | (TC)n | TRUE | ZZZ3 | 1 | NA | FALSE | FALSE |
| chr6 | 42337855 | 42337871 | 5 | 3 | NA | FALSE | NA | 0 | 1 | FALSE | FALSE |
| chr6 | 42679338 | 42679396 | 4 | 4 | NA | FALSE | NA | 0 | 1 | TRUE | FALSE |
| chr6 | 43266907 | 43266983 | 4 | 3 | NA | FALSE | MANY | 2 | 1 | TRUE | FALSE |
| chr6 | 44095260 | 44095282 | 6 | 5 | (CCCCCT)n | TRUE | MANY | 14 | 1 | TRUE | TRUE |
| chr6 | 44679230 | 44679231 | 4 | 2 | NA | FALSE | NA | 0 | NA | FALSE | FALSE |
| chr6 | 44914110 | 44914131 | 4 | 3 | Tigger1 | TRUE | NA | 0 | 1 | FALSE | FALSE |
| chr6 | 46496580 | 46496590 | 5 | 5 | (GA)n | TRUE | NA | 0 | NA | FALSE | FALSE |
| chr6 | 46999025 | 46999051 | 5 | 5 | (TC)n | TRUE | NA | 0 | 1 | TRUE | FALSE |
| chr6 | 47622873 | 47622912 | 4 | 4 | MANY | TRUE | NA | 0 | NA | FALSE | FALSE |
| chr6 | 49040590 | 49040621 | 5 | 4 | (GGAA)n | TRUE | NA | 0 | NA | TRUE | FALSE |
| chr6 | 49051914 | 49051927 | 5 | 5 | L1M1 | TRUE | NA | 0 | 1 | FALSE | FALSE |
| chr6 | 49054681 | 49054703 | 4 | 4 | NA | FALSE | NA | 0 | 1 | FALSE | FALSE |
| chr6 | 49057948 | 49057966 | 4 | 4 | AluSc | TRUE | NA | 0 | NA | FALSE | FALSE |
| chr6 | 49367402 | 49367409 | 4 | 3 | NA | FALSE | NA | 0 | 1 | FALSE | FALSE |
| chr6 | 49662514 | 49662531 | 5 | 4 | AluSg | TRUE | NA | 0 | NA | FALSE | FALSE |
| chr6 | 49982272 | 49982293 | 4 | 4 | NA | FALSE | NA | 0 | 1 | FALSE | FALSE |
| chr6 | 49989727 | 49989744 | 4 | 3 | NA | FALSE | NA | 0 | 1 | FALSE | FALSE |
| chr6 | 50081235 | 50081256 | 4 | 2 | NA | FALSE | GATA-2 | 1 | 1 | TRUE | FALSE |
| chr6 | 50347319 | 50347345 | 4 | 3 | NA | FALSE | NA | 0 | 1 | FALSE | FALSE |
| chr6 | 50587575 | 50587604 | 4 | 4 | L1PA4 | TRUE | NA | 0 | NA | FALSE | FALSE |
| chr6 | 50896207 | 50896236 | 4 | 3 | NA | FALSE | NA | 0 | 1 | FALSE | FALSE |
| chr6 | 52021940 | 52021961 | 4 | 3 | NA | FALSE | NA | 0 | 1 | FALSE | FALSE |
| chr6 | 52278849 | 52278855 | 4 | 4 | (TG)n | TRUE | NA | 0 | NA | FALSE | FALSE |
| chr6 | 52616490 | 52616618 | 9 | 5 | NA | FALSE | NA | 0 | 1 | FALSE | FALSE |
| chr6 | 52659750 | 52659756 | 9 | 6 | L1MB7 | TRUE | NA | 0 | 1 | FALSE | FALSE |
| chr6 | 52671198 | 52671256 | 4 | 3 | NA | FALSE | NA | 0 | 1 | TRUE | FALSE |
| chr6 | 53065550 | 53065594 | 6 | 4 | AluY | TRUE | NA | 0 | NA | FALSE | FALSE |
| chr6 | 54039084 | 54039105 | 4 | 2 | L1MA2 | TRUE | NA | 0 | 1 | FALSE | FALSE |
| chr6 | 54198237 | 54198255 | 4 | 3 | L1PA7 | TRUE | NA | 0 | NA | FALSE | FALSE |
| chr6 | 54333365 | 54333386 | 4 | 4 | L1PA13 | TRUE | NA | 0 | 1 | FALSE | FALSE |
| chr6 | 54424891 | 54424905 | 4 | 4 | NA | FALSE | NA | 0 | 1 | FALSE | FALSE |
| chr6 | 55008939 | 55008941 | 4 | 4 | AluSp | TRUE | NA | 0 | NA | FALSE | FALSE |
| chr6 | 55018841 | 55018857 | 4 | 3 | L1M3 | TRUE | NA | 0 | 1 | FALSE | FALSE |
| chr6 | 55149293 | 55149311 | 4 | 4 | MANY | TRUE | NA | 0 | 1 | FALSE | FALSE |
| chr6 | 55295195 | 55295207 | 4 | 4 | NA | FALSE | NA | 0 | 1 | FALSE | FALSE |
| chr6 | 56023017 | 56023044 | 4 | 3 | NA | FALSE | NA | 0 | 1 | FALSE | FALSE |
| chr6 | 57319968 | 57319974 | 4 | 3 | L1PA4 | TRUE | NA | 0 | NA | FALSE | FALSE |
| chr6 | 57449135 | 57449137 | 4 | 4 | L1PA3 | TRUE | NA | 0 | NA | FALSE | FALSE |
| chr6 | 57459060 | 57459066 | 4 | 2 | AluSx1 | TRUE | NA | 0 | NA | FALSE | FALSE |
| chr6 | 57547350 | 57547352 | 4 | 4 | L1PA7 | TRUE | NA | 0 | NA | FALSE | FALSE |
| chr6 | 57580111 | 57580125 | 4 | 4 | MLT1A | TRUE | NA | 0 | 1 | FALSE | FALSE |
| chr6 | 57810953 | 57810954 | 5 | 3 | NA | FALSE | NA | 0 | 1 | FALSE | FALSE |
| chr6 | 57849291 | 57849291 | 5 | 5 | NA | FALSE | NA | 0 | 1 | FALSE | FALSE |
| chr6 | 57911505 | 57911507 | 7 | 4 | NA | FALSE | NA | 0 | 1 | FALSE | FALSE |
| chr6 | 57959922 | 57959934 | 4 | 3 | L1PA16 | TRUE | NA | 0 | NA | FALSE | FALSE |
| chr6 | 58044196 | 58044196 | 4 | 4 | L1MA8 | TRUE | NA | 0 | NA | FALSE | FALSE |
| chr6 | 58144374 | 58144383 | 6 | 5 | L1M5 | TRUE | NA | 0 | 1 | FALSE | FALSE |
| chr6 | 58145210 | 58145212 | 5 | 4 | ERV3-16A3 I-int | TRUE | NA | 0 | 1 | FALSE | FALSE |
| chr6 | 58161277 | 58161287 | 5 | 3 | (TTTTA)n | TRUE | NA | 0 | 1 | FALSE | FALSE |
| chr6 | 58421372 | 58421373 | 6 | 4 | L1MB3 | TRUE | NA | 0 | 1 | FALSE | FALSE |
| chr6 | 58429885 | 58429888 | 4 | 2 | L1MCa | TRUE | NA | 0 | 1 | FALSE | FALSE |
| chr6 | 58533732 | 58533733 | 4 | 3 | NA | FALSE | NA | 0 | 1 | FALSE | FALSE |
| chr6 | 58655852 | 58655876 | 4 | 3 | MIRc | TRUE | NA | 0 | 1 | FALSE | FALSE |
| chr6 | 58673542 | 58673572 | 4 | 2 | NA | FALSE | NA | 0 | 1 | FALSE | FALSE |
| chr6 | 58692921 | 58692952 | 4 | 4 | NA | FALSE | NA | 0 | 1 | FALSE | FALSE |
| chr6 | 58720295 | 58720321 | 4 | 4 | L1PA4 | TRUE | NA | 0 | NA | FALSE | FALSE |
| chr6 | 58720387 | 58720416 | 4 | 4 | L1PA4 | TRUE | NA | 0 | NA | FALSE | FALSE |
| chr6 | 58739041 | 58739063 | 4 | 4 | MER9a1 | TRUE | NA | 0 | NA | FALSE | FALSE |
| chr6 | 62041922 | 62041946 | 4 | 3 | MER52-int | TRUE | NA | 0 | 1 | FALSE | FALSE |
| chr6 | 62055149 | 62055170 | 4 | 4 | Tigger1 | TRUE | NA | 0 | 1 | TRUE | FALSE |
| chr6 | 62290569 | 62290583 | 4 | 4 | LTR12 | TRUE | NA | 0 | 1 | FALSE | FALSE |
| chr6 | 62329931 | 62329943 | 4 | 3 | MIR | TRUE | NA | 0 | 1 | FALSE | FALSE |
| chr6 | 62364922 | 62364934 | 4 | 4 | LTR33A | TRUE | NA | 0 | 1 | FALSE | FALSE |
| chr6 | 62798491 | 62798493 | 4 | 2 | L1P3 | TRUE | NA | 0 | NA | FALSE | FALSE |
| chr6 | 62828361 | 62828364 | 4 | 3 | L1MA4 | TRUE | NA | 0 | 1 | FALSE | FALSE |
| chr6 | 63720038 | 63720058 | 4 | 3 | HERVH-int | TRUE | NA | 0 | NA | FALSE | FALSE |
| chr6 | 63871066 | 63871077 | 4 | 4 | MLT1L | TRUE | NA | 0 | 1 | FALSE | FALSE |
| chr6 | 63934918 | 63934919 | 4 | 4 | NA | FALSE | NA | 0 | 1 | FALSE | FALSE |
| chr6 | 64012631 | 64012631 | 4 | 4 | MER57A-int | TRUE | NA | 0 | 1 | FALSE | FALSE |
| chr6 | 64869306 | 64869326 | 5 | 3 | AluY | TRUE | NA | 0 | NA | FALSE | FALSE |
| chr6 | 66457577 | 66457629 | 4 | 3 | L1PA13 | TRUE | NA | 0 | 1 | FALSE | FALSE |
| chr6 | 67201971 | 67201991 | 4 | 2 | L1PA7 | TRUE | NA | 0 | 1 | FALSE | FALSE |
| chr6 | 67631195 | 67631234 | 4 | 4 | MANY | TRUE | NA | 0 | 1 | FALSE | FALSE |
| chr6 | 68923027 | 68923058 | 4 | 3 | L1PA7 | TRUE | NA | 0 | 1 | FALSE | FALSE |
| chr6 | 69788677 | 69788701 | 5 | 4 | L2a | TRUE | NA | 0 | 1 | FALSE | FALSE |
| chr6 | 70083995 | 70084016 | 4 | 2 | NA | FALSE | NA | 0 | 1 | FALSE | FALSE |
| chr6 | 70189395 | 70189431 | 5 | 5 | NA | FALSE | NA | 0 | 1 | FALSE | FALSE |
| chr6 | 72338821 | 72338840 | 4 | 3 | L1MA6 | TRUE | NA | 0 | 1 | FALSE | FALSE |
| chr6 | 72350423 | 72350432 | 4 | 4 | NA | FALSE | NA | 0 | 1 | FALSE | FALSE |
| chr6 | 72384226 | 72384236 | 4 | 3 | NA | FALSE | NA | 0 | 1 | FALSE | FALSE |
| chr6 | 72476106 | 72476126 | 4 | 4 | NA | FALSE | NA | 0 | 1 | FALSE | FALSE |
| chr6 | 72719866 | 72719869 | 5 | 3 | L1PA4 | TRUE | NA | 0 | NA | FALSE | FALSE |
| chr6 | 72766741 | 72766749 | 4 | 3 | L1PA13 | TRUE | NA | 0 | NA | FALSE | FALSE |
| chr6 | 72802001 | 72802018 | 4 | 3 | (TTCC)n | TRUE | NA | 0 | 1 | TRUE | FALSE |
| chr6 | 72912002 | 72912023 | 4 | 4 | MLT1B | TRUE | NA | 0 | 1 | FALSE | FALSE |
| chr6 | 73161664 | 73161673 | 4 | 3 | L3b | TRUE | NA | 0 | 1 | FALSE | FALSE |
| chr6 | 73748243 | 73748261 | 4 | 3 | L1MD2 | TRUE | NA | 0 | 1 | FALSE | FALSE |
| chr6 | 73773424 | 73773471 | 6 | 3 | (TAGA)n | TRUE | NA | 0 | NA | FALSE | FALSE |
| chr6 | 73799550 | 73799562 | 4 | 2 | L1PB1 | TRUE | NA | 0 | NA | FALSE | FALSE |
| chr6 | 74020399 | 74020425 | 6 | 2 | L1PA5 | TRUE | PU.1 | 1 | 1 | FALSE | FALSE |
| chr6 | 74043021 | 74043049 | 7 | 5 | HERVK-int | TRUE | NA | 0 | 1 | FALSE | FALSE |
| chr6 | 74230296 | 74230336 | 6 | 3 | NA | FALSE | MANY | 12 | 1 | TRUE | TRUE |
| chr6 | 74745346 | 74745387 | 5 | 5 | L1P2 | TRUE | NA | 0 | 1 | FALSE | FALSE |
| chr6 | 75201726 | 75201727 | 4 | 3 | L1MCa | TRUE | NA | 0 | 1 | FALSE | FALSE |
| chr6 | 75312165 | 75312183 | 4 | 3 | NA | FALSE | NA | 0 | 1 | TRUE | FALSE |
| chr6 | 75512164 | 75512188 | 4 | 2 | L2a | TRUE | NA | 0 | 1 | TRUE | FALSE |
| chr6 | 76860249 | 76860261 | 5 | 2 | L1PA5 | TRUE | NA | 0 | NA | FALSE | FALSE |
| chr6 | 77081720 | 77081743 | 4 | 4 | L1PA4 | TRUE | NA | 0 | NA | FALSE | FALSE |
| chr6 | 77828600 | 77828613 | 4 | 3 | NA | FALSE | NA | 0 | 1 | FALSE | FALSE |
| chr6 | 78254594 | 78254600 | 4 | 4 | NA | FALSE | NA | 0 | 1 | FALSE | FALSE |
| chr6 | 78672095 | 78672109 | 5 | 4 | THE1A-int | TRUE | NA | 0 | 1 | TRUE | FALSE |
| chr6 | 78901920 | 78901923 | 5 | 4 | MSTA-int | TRUE | MANY | 2 | 1 | TRUE | FALSE |
| chr6 | 79065936 | 79065948 | 4 | 4 | NA | FALSE | NA | 0 | 1 | FALSE | FALSE |
| chr6 | 79172838 | 79172838 | 4 | 4 | AluY | TRUE | NA | 0 | NA | FALSE | FALSE |
| chr6 | 79212714 | 79212731 | 4 | 3 | L1PA15 | TRUE | NA | 0 | 1 | FALSE | FALSE |
| chr6 | 79301241 | 79301257 | 4 | 4 | NA | FALSE | NA | 0 | 1 | FALSE | FALSE |
| chr6 | 80010095 | 80010153 | 4 | 3 | L1MA10 | TRUE | NA | 0 | 1 | FALSE | FALSE |
| chr6 | 82699479 | 82699482 | 4 | 3 | L1M6 | TRUE | NA | 0 | 1 | FALSE | FALSE |
| chr6 | 83184179 | 83184212 | 4 | 2 | L1PA4 | TRUE | NA | 0 | NA | FALSE | FALSE |
| chr6 | 83221454 | 83221468 | 4 | 3 | NA | FALSE | NA | 0 | 1 | TRUE | FALSE |
| chr6 | 83331134 | 83331161 | 4 | 2 | AluY | TRUE | NA | 0 | NA | FALSE | FALSE |
| chr6 | 83338292 | 83338328 | 4 | 4 | MER102b | TRUE | NA | 0 | 1 | FALSE | FALSE |
| chr6 | 84020673 | 84020687 | 4 | 3 | ERV3-16A3 I-int | TRUE | NA | 0 | 1 | FALSE | FALSE |
| chr6 | 84030429 | 84030453 | 4 | 2 | NA | FALSE | NA | 0 | 1 | TRUE | FALSE |
| chr6 | 84157038 | 84157048 | 4 | 2 | HERV17-int | TRUE | NA | 0 | 1 | FALSE | FALSE |
| chr6 | 84294325 | 84294342 | 4 | 3 | MADE2 | TRUE | NA | 0 | 1 | FALSE | FALSE |
| chr6 | 84518422 | 84518443 | 4 | 2 | AluJo | TRUE | NA | 0 | NA | FALSE | FALSE |
| chr6 | 84749969 | 84749992 | 4 | 3 | THE1C | TRUE | NA | 0 | 1 | FALSE | FALSE |
| chr6 | 85050461 | 85050479 | 4 | 4 | L1PB1 | TRUE | NA | 0 | 1 | TRUE | FALSE |
| chr6 | 85144763 | 85144787 | 4 | 4 | NA | FALSE | NA | 0 | 1 | FALSE | FALSE |
| chr6 | 85215827 | 85215849 | 4 | 4 | AluSc | TRUE | NA | 0 | NA | FALSE | FALSE |
| chr6 | 85225473 | 85225482 | 4 | 4 | NA | FALSE | NA | 0 | 1 | FALSE | FALSE |
| chr6 | 85369607 | 85369622 | 4 | 4 | NA | FALSE | NA | 0 | 1 | FALSE | FALSE |
| chr6 | 85404803 | 85404812 | 4 | 4 | NA | FALSE | NA | 0 | 1 | FALSE | FALSE |
| chr6 | 85873425 | 85873439 | 4 | 3 | NA | FALSE | NA | 0 | 1 | TRUE | FALSE |
| chr6 | 86499481 | 86499490 | 4 | 4 | AluJr | TRUE | NA | 0 | 1 | FALSE | FALSE |
| chr6 | 86554133 | 86554163 | 4 | 3 | L1PA6 | TRUE | NA | 0 | NA | FALSE | FALSE |
| chr6 | 86995094 | 86995094 | 4 | 4 | L1PA4 | TRUE | NA | 0 | NA | FALSE | FALSE |
| chr6 | 86995149 | 86995150 | 8 | 5 | L1PA4 | TRUE | NA | 0 | NA | FALSE | FALSE |
| chr6 | 87585943 | 87585963 | 4 | 2 | L1PA3 | TRUE | NA | 0 | NA | FALSE | FALSE |
| chr6 | 87719402 | 87719425 | 4 | 2 | SVA F | TRUE | NA | 0 | NA | FALSE | FALSE |
| chr6 | 88765583 | 88765615 | 4 | 3 | AluSc | TRUE | NA | 0 | NA | FALSE | FALSE |
| chr6 | 88836537 | 88836549 | 6 | 4 | AluSz | TRUE | NA | 0 | NA | FALSE | FALSE |
| chr6 | 88934712 | 88934730 | 4 | 4 | AluJr4 | TRUE | NA | 0 | NA | FALSE | FALSE |
| chr6 | 88999544 | 88999560 | 4 | 3 | NA | FALSE | NA | 0 | 1 | FALSE | FALSE |
| chr6 | 89102906 | 89102916 | 5 | 3 | L1PA6 | TRUE | NA | 0 | NA | FALSE | FALSE |
| chr6 | 89224860 | 89224901 | 4 | 3 | AluY | TRUE | NA | 0 | NA | FALSE | FALSE |
| chr6 | 89677839 | 89677840 | 4 | 2 | MER61-int | TRUE | MANY | 2 | 1 | FALSE | FALSE |
| chr6 | 90810420 | 90810446 | 6 | 6 | (CA)n | TRUE | NA | 0 | NA | FALSE | FALSE |
| chr6 | 91582757 | 91582772 | 4 | 4 | NA | FALSE | NA | 0 | 1 | TRUE | FALSE |
| chr6 | 91616545 | 91616554 | 4 | 3 | L1M1 | TRUE | NA | 0 | 1 | FALSE | FALSE |
| chr6 | 92262269 | 92262271 | 4 | 2 | THE1D-int | TRUE | NA | 0 | NA | FALSE | FALSE |
| chr6 | 93023963 | 93023972 | 4 | 4 | NA | FALSE | NA | 0 | 1 | FALSE | FALSE |
| chr6 | 93234604 | 93234604 | 4 | 4 | NA | FALSE | NA | 0 | 1 | FALSE | FALSE |
| chr6 | 93303557 | 93303568 | 4 | 4 | NA | FALSE | NA | 0 | 1 | FALSE | FALSE |
| chr6 | 94138125 | 94138132 | 4 | 4 | MLT1F2 | TRUE | NA | 0 | 1 | TRUE | FALSE |
| chr6 | 94210383 | 94210402 | 5 | 4 | L1ME3A | TRUE | NA | 0 | 1 | FALSE | FALSE |
| chr6 | 94318904 | 94318920 | 4 | 4 | NA | FALSE | NA | 0 | 1 | FALSE | FALSE |
| chr6 | 94566521 | 94566534 | 5 | 4 | L1PB3 | TRUE | NA | 0 | 1 | FALSE | FALSE |
| chr6 | 94613415 | 94613422 | 4 | 4 | MLT1B | TRUE | NA | 0 | 1 | FALSE | FALSE |
| chr6 | 94846930 | 94846943 | 4 | 4 | NA | FALSE | NA | 0 | 1 | FALSE | FALSE |
| chr6 | 94949435 | 94949441 | 4 | 4 | L1PA7 | TRUE | NA | 0 | NA | FALSE | FALSE |
| chr6 | 94951104 | 94951118 | 4 | 4 | L1PA7 | TRUE | NA | 0 | NA | FALSE | FALSE |
| chr6 | 94968288 | 94968301 | 4 | 3 | NA | FALSE | NA | 0 | 1 | FALSE | FALSE |
| chr6 | 94979677 | 94979694 | 4 | 4 | NA | FALSE | NA | 0 | 1 | FALSE | FALSE |
| chr6 | 95629469 | 95629491 | 4 | 4 | THE1C-int | TRUE | NA | 0 | 1 | FALSE | FALSE |
| chr6 | 95896636 | 95896637 | 4 | 3 | MANY | TRUE | NA | 0 | 1 | FALSE | FALSE |
| chr6 | 96155971 | 96155987 | 4 | 3 | NA | FALSE | NA | 0 | 1 | FALSE | FALSE |
| chr6 | 96356081 | 96356095 | 4 | 4 | NA | FALSE | NA | 0 | 1 | FALSE | FALSE |
| chr6 | 96591356 | 96591368 | 5 | 3 | CT-rich | TRUE | NA | 0 | 1 | FALSE | FALSE |
| chr6 | 96726116 | 96726130 | 4 | 4 | L1M6 | TRUE | NA | 0 | 1 | FALSE | FALSE |
| chr6 | 97039584 | 97039606 | 4 | 4 | MANY | TRUE | NA | 0 | 1 | TRUE | FALSE |
| chr6 | 97927887 | 97927917 | 4 | 4 | L1MB2 | TRUE | NA | 0 | 1 | FALSE | FALSE |
| chr6 | 98054937 | 98054968 | 4 | 4 | L1PB1 | TRUE | NA | 0 | NA | FALSE | FALSE |
| chr6 | 98510926 | 98510950 | 5 | 5 | NA | FALSE | NA | 0 | 1 | FALSE | FALSE |
| chr6 | 98642362 | 98642389 | 4 | 3 | L1MB2 | TRUE | NA | 0 | 1 | FALSE | FALSE |
| chr6 | 98845667 | 98845694 | 4 | 3 | NA | FALSE | NA | 0 | 1 | FALSE | FALSE |
| chr6 | 99466184 | 99466191 | 4 | 3 | NA | FALSE | NA | 0 | 1 | TRUE | FALSE |
| chr6 | 100258084 | 100258087 | 4 | 3 | (TG)n | TRUE | NA | 0 | NA | FALSE | FALSE |
| chr6 | 100497893 | 100497907 | 4 | 4 | L1PA8 | TRUE | NA | 0 | NA | FALSE | FALSE |
| chr6 | 100498406 | 100498432 | 4 | 2 | NA | FALSE | FOXA1 (C-20) | 1 | 1 | FALSE | FALSE |
| chr6 | 100517919 | 100517919 | 5 | 5 | L1PA3 | TRUE | NA | 0 | 1 | FALSE | FALSE |
| chr6 | 100604180 | 100604218 | 5 | 4 | (TAGA)n | TRUE | NA | 0 | NA | FALSE | FALSE |
| chr6 | 100652971 | 100652993 | 4 | 3 | L1MC4 | TRUE | NA | 0 | 1 | FALSE | FALSE |
| chr6 | 101696223 | 101696258 | 4 | 2 | L1PA4 | TRUE | NA | 0 | NA | FALSE | FALSE |
| chr6 | 102257295 | 102257320 | 4 | 3 | NA | FALSE | NA | 0 | 1 | FALSE | FALSE |
| chr6 | 102455775 | 102455790 | 4 | 3 | NA | FALSE | NA | 0 | 1 | FALSE | FALSE |
| chr6 | 103034297 | 103034312 | 4 | 3 | AluJr | TRUE | NA | 0 | 1 | FALSE | FALSE |
| chr6 | 103043986 | 103044001 | 4 | 4 | NA | FALSE | NA | 0 | 1 | FALSE | FALSE |
| chr6 | 103122906 | 103122920 | 4 | 1 | NA | FALSE | NA | 0 | 1 | FALSE | FALSE |
| chr6 | 103871831 | 103871832 | 4 | 4 | NA | FALSE | NA | 0 | 1 | FALSE | FALSE |
| chr6 | 103876476 | 103876476 | 4 | 4 | L1PA16 | TRUE | NA | 0 | 1 | FALSE | FALSE |
| chr6 | 104198653 | 104198671 | 4 | 4 | MER61A | TRUE | NA | 0 | 1 | TRUE | FALSE |
| chr6 | 104305759 | 104305759 | 4 | 4 | L1ME3B | TRUE | NA | 0 | 1 | FALSE | FALSE |
| chr6 | 104457611 | 104457621 | 4 | 4 | NA | FALSE | NA | 0 | 1 | TRUE | FALSE |
| chr6 | 104710726 | 104710726 | 5 | 5 | NA | FALSE | NA | 0 | 1 | FALSE | FALSE |
| chr6 | 104872205 | 104872230 | 5 | 5 | GA-rich | TRUE | NA | 0 | NA | FALSE | FALSE |
| chr6 | 104894896 | 104894896 | 5 | 5 | LTR12C | TRUE | NA | 0 | NA | FALSE | FALSE |
| chr6 | 105249658 | 105249687 | 6 | 3 | L1PA6 | TRUE | NA | 0 | 1 | FALSE | FALSE |
| chr6 | 105249784 | 105249796 | 8 | 4 | L1PA6 | TRUE | NA | 0 | NA | FALSE | FALSE |
| chr6 | 107262608 | 107262677 | 4 | 3 | MANY | TRUE | NA | 0 | 1 | TRUE | FALSE |
| chr6 | 107454740 | 107454841 | 9 | 5 | MANY | TRUE | NA | 0 | 1 | FALSE | FALSE |
| chr6 | 108169700 | 108169756 | 4 | 3 | NA | FALSE | MANY | 22 | 1 | TRUE | TRUE |
| chr6 | 110074255 | 110074301 | 4 | 4 | L1PA3 | TRUE | NA | 0 | NA | FALSE | FALSE |
| chr6 | 110485692 | 110485710 | 4 | 2 | L1MC1 | TRUE | NA | 0 | 1 | FALSE | FALSE |
| chr6 | 110808584 | 110808624 | 6 | 4 | AluSx3 | TRUE | NA | 0 | NA | FALSE | FALSE |
| chr6 | 111488746 | 111488866 | 4 | 2 | MANY | TRUE | NA | 0 | 1 | TRUE | FALSE |
| chr6 | 112490550 | 112490565 | 4 | 4 | NA | FALSE | NA | 0 | 1 | FALSE | FALSE |
| chr6 | 116131554 | 116131574 | 4 | 4 | L2 | TRUE | NA | 0 | 1 | FALSE | FALSE |
| chr6 | 116236091 | 116236118 | 4 | 2 | NA | FALSE | NA | 0 | 1 | FALSE | FALSE |
| chr6 | 116261474 | 116261504 | 4 | 2 | L1PA7 | TRUE | NA | 0 | NA | FALSE | FALSE |
| chr6 | 116810634 | 116810685 | 4 | 2 | L1PA7 | TRUE | NA | 0 | 1 | FALSE | FALSE |
| chr6 | 117005032 | 117005057 | 5 | 3 | AluY | TRUE | NA | 0 | NA | FALSE | FALSE |
| chr6 | 117357266 | 117357267 | 4 | 4 | L2a | TRUE | NA | 0 | 1 | FALSE | FALSE |
| chr6 | 118376636 | 118376680 | 4 | 3 | NA | FALSE | NA | 0 | 1 | FALSE | FALSE |
| chr6 | 119271724 | 119271824 | 7 | 4 | MANY | TRUE | NA | 0 | NA | FALSE | FALSE |
| chr6 | 119726708 | 119726715 | 4 | 4 | L1PA4 | TRUE | NA | 0 | NA | FALSE | FALSE |
| chr6 | 120085959 | 120085968 | 4 | 4 | LTR10D | TRUE | NA | 0 | NA | FALSE | FALSE |
| chr6 | 120128178 | 120128192 | 4 | 4 | HAL1 | TRUE | NA | 0 | 1 | FALSE | FALSE |
| chr6 | 121246428 | 121246444 | 4 | 1 | HERVH-int | TRUE | NA | 0 | NA | FALSE | FALSE |
| chr6 | 121308568 | 121308580 | 4 | 4 | MER74B | TRUE | NA | 0 | 1 | FALSE | FALSE |
| chr6 | 121772203 | 121772204 | 4 | 2 | AluY | TRUE | NA | 0 | NA | FALSE | FALSE |
| chr6 | 122589406 | 122589431 | 4 | 2 | L1ME1 | TRUE | NA | 0 | 1 | FALSE | FALSE |
| chr6 | 122592015 | 122592056 | 4 | 3 | NA | FALSE | NA | 0 | 1 | FALSE | FALSE |
| chr6 | 123307305 | 123307344 | 5 | 5 | L1P3 | TRUE | NA | 0 | 1 | FALSE | FALSE |
| chr6 | 123321797 | 123321831 | 4 | 2 | NA | FALSE | NA | 0 | 1 | FALSE | FALSE |
| chr6 | 123395513 | 123395538 | 4 | 4 | NA | FALSE | NA | 0 | 1 | FALSE | FALSE |
| chr6 | 123482129 | 123482165 | 5 | 4 | L2 | TRUE | NA | 0 | 1 | FALSE | FALSE |
| chr6 | 124317239 | 124317257 | 4 | 4 | NA | FALSE | NA | 0 | 1 | FALSE | FALSE |
| chr6 | 125103102 | 125103131 | 4 | 3 | NA | FALSE | NA | 0 | 1 | TRUE | FALSE |
| chr6 | 125861848 | 125861870 | 4 | 2 | AluJb | TRUE | NA | 0 | NA | FALSE | FALSE |
| chr6 | 127094248 | 127094255 | 4 | 4 | NA | FALSE | NA | 0 | 1 | FALSE | FALSE |
| chr6 | 127924605 | 127924613 | 4 | 4 | L1M1 | TRUE | NA | 0 | 1 | FALSE | FALSE |
| chr6 | 128103585 | 128103614 | 5 | 4 | L1P2 | TRUE | NA | 0 | 1 | FALSE | FALSE |
| chr6 | 129080511 | 129080541 | 5 | 4 | L1M2c | TRUE | NA | 0 | 1 | TRUE | FALSE |
| chr6 | 129881408 | 129881417 | 7 | 4 | L1PA4 | TRUE | NA | 0 | NA | FALSE | FALSE |
| chr6 | 130174098 | 130174130 | 4 | 3 | NA | FALSE | Pol2 | 1 | 1 | FALSE | FALSE |
| chr6 | 130703976 | 130703984 | 4 | 4 | NA | FALSE | NA | 0 | 1 | FALSE | FALSE |
| chr6 | 131018637 | 131018652 | 4 | 3 | NA | FALSE | NA | 0 | 1 | TRUE | FALSE |
| chr6 | 131829353 | 131829366 | 5 | 3 | HERVH-int | TRUE | NA | 0 | 1 | TRUE | FALSE |
| chr6 | 133434502 | 133434549 | 5 | 2 | LTR33B | TRUE | STAT1 | 1 | 1 | FALSE | FALSE |
| chr6 | 134086276 | 134086308 | 4 | 3 | NA | FALSE | NA | 0 | 1 | TRUE | FALSE |
| chr6 | 134093135 | 134093153 | 4 | 3 | NA | FALSE | NA | 0 | 1 | FALSE | FALSE |
| chr6 | 134120202 | 134120233 | 4 | 4 | L1PREC2 | TRUE | NA | 0 | 1 | TRUE | FALSE |
| chr6 | 135051668 | 135051736 | 5 | 3 | AluY | TRUE | NA | 0 | NA | FALSE | FALSE |
| chr6 | 135973012 | 135973033 | 4 | 4 | L1MCa | TRUE | NA | 0 | 1 | FALSE | FALSE |
| chr6 | 137317275 | 137317328 | 5 | 5 | MIR | TRUE | NA | 0 | 1 | FALSE | FALSE |
| chr6 | 139713710 | 139713757 | 6 | 3 | AluY | TRUE | NA | 0 | NA | FALSE | FALSE |
| chr6 | 140245729 | 140245779 | 4 | 3 | (TTTC)n | TRUE | NA | 0 | 1 | FALSE | FALSE |
| chr6 | 140582465 | 140582490 | 5 | 2 | HERVH-int | TRUE | NA | 0 | NA | FALSE | FALSE |
| chr6 | 140695794 | 140695856 | 6 | 3 | SVA C | TRUE | NA | 0 | NA | FALSE | FALSE |
| chr6 | 141187102 | 141187131 | 4 | 3 | NA | FALSE | NA | 0 | 1 | FALSE | FALSE |
| chr6 | 141884146 | 141884166 | 4 | 4 | NA | FALSE | MANY | 13 | 1 | TRUE | TRUE |
| chr6 | 142921756 | 142921818 | 4 | 4 | L2c | TRUE | NA | 0 | 1 | FALSE | FALSE |
| chr6 | 143132338 | 143132347 | 7 | 4 | (GA)n | TRUE | NFKB | 1 | NA | TRUE | FALSE |
| chr6 | 143356557 | 143356599 | 4 | 3 | L1PA6 | TRUE | NA | 0 | 1 | FALSE | FALSE |
| chr6 | 145427368 | 145427382 | 4 | 2 | HERVH-int | TRUE | NA | 0 | NA | FALSE | FALSE |
| chr6 | 146538806 | 146538827 | 4 | 3 | L2c | TRUE | NA | 0 | 1 | FALSE | FALSE |
| chr6 | 146841279 | 146841284 | 4 | 2 | L1PA5 | TRUE | NA | 0 | NA | FALSE | FALSE |
| chr6 | 147744333 | 147744424 | 4 | 4 | LTR28 | TRUE | NA | 0 | 1 | FALSE | FALSE |
| chr6 | 148500222 | 148500284 | 4 | 3 | SVA D | TRUE | NA | 0 | NA | FALSE | FALSE |
| chr6 | 148961115 | 148961184 | 4 | 2 | LTR7 | TRUE | NA | 0 | NA | TRUE | FALSE |
| chr6 | 149867284 | 149867285 | 4 | 3 | NA | FALSE | MANY | 52 | 1 | TRUE | TRUE |
| chr6 | 150264141 | 150264201 | 5 | 3 | MER58B | TRUE | NA | 0 | 1 | FALSE | FALSE |
| chr6 | 150519151 | 150519222 | 4 | 3 | NA | FALSE | NA | 0 | 1 | TRUE | FALSE |
| chr6 | 151355923 | 151356143 | 12 | 7 | NA | FALSE | NA | 0 | 1 | TRUE | FALSE |
| chr6 | 151357532 | 151357784 | 14 | 9 | NA | FALSE | MANY | 27 | 1 | TRUE | TRUE |
| chr6 | 151458230 | 151458316 | 4 | 3 | AluSz6 | TRUE | NA | 0 | 1 | TRUE | FALSE |
| chr6 | 152220275 | 152220320 | 4 | 3 | SVA D | TRUE | NA | 0 | NA | FALSE | FALSE |
| chr6 | 152333182 | 152333188 | 4 | 2 | (CA)n | TRUE | NA | 0 | NA | FALSE | FALSE |
| chr6 | 152398162 | 152398194 | 4 | 3 | THE1A-int | TRUE | NA | 0 | NA | TRUE | FALSE |
| chr6 | 153097879 | 153097916 | 4 | 4 | LTR41B | TRUE | NA | 0 | 1 | FALSE | FALSE |
| chr6 | 153171416 | 153171441 | 4 | 3 | NA | FALSE | NA | 0 | 1 | FALSE | FALSE |
| chr6 | 155008633 | 155008716 | 5 | 4 | SVA D | TRUE | NA | 0 | NA | FALSE | FALSE |
| chr6 | 155749195 | 155749235 | 4 | 3 | AluSz6 | TRUE | NA | 0 | NA | FALSE | FALSE |
| chr6 | 155949052 | 155949119 | 6 | 4 | (GGAA)n | TRUE | NA | 0 | NA | FALSE | FALSE |
| chr6 | 157638727 | 157638767 | 4 | 2 | NA | FALSE | MANY | 10 | 1 | TRUE | TRUE |
| chr6 | 157849555 | 157849668 | 5 | 3 | NA | FALSE | NA | 0 | 1 | TRUE | FALSE |
| chr6 | 158456215 | 158456243 | 4 | 3 | L1PA13 | TRUE | NA | 0 | NA | FALSE | FALSE |
| chr6 | 159225733 | 159225810 | 4 | 3 | L1PA2 | TRUE | NA | 0 | 1 | FALSE | FALSE |
| chr6 | 159787713 | 159787753 | 4 | 3 | HERVL-int | TRUE | NA | 0 | 1 | FALSE | FALSE |
| chr6 | 160288194 | 160288237 | 4 | 3 | NA | FALSE | NA | 0 | 1 | FALSE | FALSE |
| chr6 | 161030770 | 161030785 | 5 | 4 | L1MEc | TRUE | NA | 0 | 1 | FALSE | FALSE |
| chr6 | 161104560 | 161104597 | 4 | 4 | L1PA3 | TRUE | NA | 0 | 1 | FALSE | FALSE |
| chr6 | 161138808 | 161138823 | 4 | 4 | NA | FALSE | NA | 0 | 1 | FALSE | FALSE |
| chr6 | 161833050 | 161833052 | 4 | 2 | (TTCA)n | TRUE | NA | 0 | NA | FALSE | FALSE |
| chr6 | 162014323 | 162014360 | 4 | 3 | MIR | TRUE | NA | 0 | 1 | FALSE | FALSE |
| chr6 | 163405215 | 163405227 | 4 | 2 | L1PB1 | TRUE | NA | 0 | 1 | FALSE | FALSE |
| chr6 | 165198887 | 165198910 | 4 | 3 | L1PA5 | TRUE | NA | 0 | NA | FALSE | FALSE |
| chr6 | 165875595 | 165875612 | 4 | 3 | HERVK9-int | TRUE | NA | 0 | 1 | FALSE | FALSE |
| chr6 | 166601456 | 166601494 | 4 | 3 | AluSx1 | TRUE | NA | 0 | NA | FALSE | FALSE |
| chr6 | 167800769 | 167800826 | 4 | 3 | (TG)n | TRUE | NA | 0 | NA | FALSE | FALSE |
| chr6 | 168047366 | 168047372 | 4 | 2 | NA | FALSE | NA | 0 | 1 | FALSE | FALSE |
| chr6 | 168099563 | 168099645 | 6 | 3 | NA | FALSE | NA | 0 | 1 | TRUE | FALSE |
| chr6 | 168486460 | 168486504 | 5 | 5 | NA | FALSE | NA | 0 | 1 | TRUE | FALSE |
| chr6 | 168637538 | 168637562 | 4 | 4 | NA | FALSE | NA | 0 | NA | FALSE | FALSE |
| chr6 | 168637597 | 168637644 | 4 | 3 | NA | FALSE | NA | 0 | 1 | FALSE | FALSE |
| chr6 | 168866714 | 168866752 | 4 | 4 | NA | FALSE | NA | 0 | NA | TRUE | FALSE |
| chr6 | 168994991 | 168995025 | 5 | 5 | NA | FALSE | NA | 0 | NA | FALSE | FALSE |
| chr6 | 169014384 | 169014406 | 4 | 4 | TAR1 | TRUE | NA | 0 | NA | FALSE | FALSE |
| chr6 | 169015203 | 169015225 | 4 | 4 | TAR1 | TRUE | NA | 0 | NA | FALSE | FALSE |
| chr6 | 169249986 | 169250044 | 6 | 5 | NA | FALSE | NA | 0 | 1 | TRUE | FALSE |
| chr6 | 169256795 | 169256811 | 4 | 3 | NA | FALSE | NA | 0 | 1 | TRUE | FALSE |
| chr6 | 169298121 | 169298121 | 4 | 4 | (TC)n | TRUE | NA | 0 | NA | FALSE | FALSE |
| chr6 | 169342158 | 169342192 | 4 | 2 | NA | FALSE | NA | 0 | NA | FALSE | FALSE |
| chr6 | 169531329 | 169531361 | 4 | 2 | L1MA2 | TRUE | NA | 0 | 1 | FALSE | FALSE |
| chr6 | 169592457 | 169592605 | 10 | 6 | NA | FALSE | NA | 0 | 1 | FALSE | FALSE |
| chr6 | 169825750 | 169825798 | 4 | 3 | NA | FALSE | NA | 0 | NA | TRUE | FALSE |
| chr6 | 170465095 | 170465117 | 4 | 3 | NA | FALSE | NA | 0 | 1 | FALSE | FALSE |
| chr6 | 170469595 | 170469653 | 4 | 4 | NA | FALSE | NA | 0 | 1 | TRUE | FALSE |
| chr6 | 170805008 | 170805057 | 4 | 3 | NA | FALSE | NA | 0 | 1 | TRUE | FALSE |
| chr6 | 170816882 | 170816975 | 5 | 5 | MER82 | TRUE | NA | 0 | NA | FALSE | FALSE |
| chr6 | 170893741 | 170893741 | 4 | 4 | NA | FALSE | MANY | 37 | 1 | TRUE | TRUE |
| chr7 | 376457 | 376500 | 4 | 4 | NA | FALSE | NA | 0 | 1 | FALSE | FALSE |
| chr7 | 451144 | 451165 | 5 | 3 | NA | FALSE | NA | 0 | NA | FALSE | FALSE |
| chr7 | 669143 | 669180 | 4 | 3 | NA | FALSE | NA | 0 | 1 | FALSE | FALSE |
| chr7 | 895538 | 895571 | 4 | 3 | NA | FALSE | NA | 0 | 1 | TRUE | FALSE |
| chr7 | 916027 | 916064 | 4 | 3 | NA | FALSE | MANY | 35 | 1 | TRUE | TRUE |
| chr7 | 945630 | 945674 | 8 | 5 | GA-rich | TRUE | NA | 0 | NA | FALSE | FALSE |
| chr7 | 977207 | 977253 | 6 | 5 | NA | FALSE | NA | 0 | NA | FALSE | FALSE |
| chr7 | 1211125 | 1211198 | 13 | 8 | LTR12F | TRUE | MANY | 5 | NA | TRUE | FALSE |
| chr7 | 1211342 | 1211343 | 4 | 3 | LTR12F | TRUE | MANY | 5 | NA | TRUE | FALSE |
| chr7 | 1269637 | 1269641 | 5 | 3 | NA | FALSE | Pol2 | 1 | 1 | TRUE | FALSE |
| chr7 | 1333728 | 1333750 | 4 | 2 | NA | FALSE | NA | 0 | 1 | FALSE | FALSE |
| chr7 | 1346110 | 1346114 | 6 | 4 | (CCCA)n | TRUE | Egr-1 | 1 | NA | FALSE | FALSE |
| chr7 | 1544047 | 1544063 | 9 | 7 | NA | FALSE | MANY | 52 | 1 | TRUE | TRUE |
| chr7 | 1639617 | 1639643 | 4 | 4 | NA | FALSE | NA | 0 | 1 | TRUE | FALSE |
| chr7 | 1735810 | 1735812 | 4 | 4 | MANY | TRUE | NA | 0 | NA | FALSE | FALSE |
| chr7 | 1909927 | 1910016 | 7 | 6 | NA | FALSE | NA | 0 | 1 | FALSE | FALSE |
| chr7 | 2342519 | 2342573 | 6 | 2 | SVA D | TRUE | NA | 0 | NA | TRUE | FALSE |
| chr7 | 2394147 | 2394225 | 4 | 3 | NA | FALSE | MANY | 16 | 1 | TRUE | TRUE |
| chr7 | 2623413 | 2623420 | 4 | 3 | (CGGGG)n | TRUE | NA | 0 | NA | FALSE | FALSE |
| chr7 | 2671396 | 2671445 | 6 | 5 | NA | FALSE | MANY | 13 | 1 | TRUE | TRUE |
| chr7 | 2917160 | 2917180 | 4 | 3 | NA | FALSE | NA | 0 | 1 | TRUE | FALSE |
| chr7 | 2983802 | 2983811 | 5 | 4 | NA | FALSE | NA | 0 | 1 | FALSE | FALSE |
| chr7 | 3052326 | 3052337 | 4 | 2 | MIR | TRUE | NA | 0 | 1 | TRUE | FALSE |
| chr7 | 3094732 | 3094768 | 7 | 4 | AluY | TRUE | NA | 0 | 1 | FALSE | FALSE |
| chr7 | 3882610 | 3882646 | 4 | 2 | NA | FALSE | NA | 0 | 1 | TRUE | FALSE |
| chr7 | 4220978 | 4221001 | 4 | 3 | L1MB3 | TRUE | NA | 0 | 1 | FALSE | FALSE |
| chr7 | 4602808 | 4602840 | 4 | 3 | AluJo | TRUE | NA | 0 | NA | FALSE | FALSE |
| chr7 | 5009455 | 5009501 | 6 | 3 | AluJr | TRUE | NA | 0 | 1 | FALSE | FALSE |
| chr7 | 5170338 | 5170372 | 5 | 4 | AluJr | TRUE | NA | 0 | 1 | TRUE | FALSE |
| chr7 | 5303481 | 5303501 | 10 | 5 | NA | FALSE | NA | 0 | 1 | FALSE | FALSE |
| chr7 | 5318687 | 5318687 | 5 | 5 | NA | FALSE | NA | 0 | 1 | TRUE | FALSE |
| chr7 | 5337863 | 5337889 | 6 | 5 | NA | FALSE | NA | 0 | 1 | TRUE | FALSE |
| chr7 | 5553438 | 5553461 | 5 | 5 | NA | FALSE | MANY | 27 | 1 | TRUE | TRUE |
| chr7 | 5609895 | 5609917 | 4 | 4 | NA | FALSE | MANY | 17 | 1 | TRUE | TRUE |
| chr7 | 5821277 | 5821324 | 4 | 2 | NA | FALSE | MANY | 38 | 1 | TRUE | TRUE |
| chr7 | 5941581 | 5941599 | 4 | 3 | NA | FALSE | NA | 0 | NA | FALSE | FALSE |
| chr7 | 6048787 | 6048880 | 9 | 4 | NA | FALSE | MANY | 34 | 1 | TRUE | TRUE |
| chr7 | 6093135 | 6093172 | 5 | 5 | AluSc | TRUE | NA | 0 | NA | FALSE | FALSE |
| chr7 | 6647472 | 6647495 | 4 | 2 | NA | FALSE | NA | 0 | 1 | TRUE | FALSE |
| chr7 | 6650481 | 6650490 | 4 | 2 | NA | FALSE | NA | 0 | 1 | FALSE | FALSE |
| chr7 | 6950310 | 6950336 | 7 | 5 | L1MC1 | TRUE | NA | 0 | 1 | FALSE | FALSE |
| chr7 | 7111993 | 7111995 | 4 | 4 | SATR1 | TRUE | NA | 0 | 1 | FALSE | FALSE |
| chr7 | 8192292 | 8192333 | 4 | 4 | (CCA)n | TRUE | c-Myc | 1 | 1 | TRUE | FALSE |
| chr7 | 8685610 | 8685611 | 4 | 4 | NA | FALSE | NA | 0 | 1 | FALSE | FALSE |
| chr7 | 8886312 | 8886320 | 4 | 3 | L1M5 | TRUE | NA | 0 | 1 | FALSE | FALSE |
| chr7 | 9356688 | 9356712 | 4 | 2 | L3 | TRUE | NA | 0 | 1 | FALSE | FALSE |
| chr7 | 9575089 | 9575113 | 4 | 4 | L1PA16 | TRUE | NA | 0 | 1 | FALSE | FALSE |
| chr7 | 10244825 | 10244870 | 5 | 3 | (GGGAA)n | TRUE | NA | 0 | NA | FALSE | FALSE |
| chr7 | 10535536 | 10535536 | 4 | 4 | L1PA10 | TRUE | NA | 0 | 1 | FALSE | FALSE |
| chr7 | 10778106 | 10778109 | 4 | 4 | NA | FALSE | NA | 0 | 1 | FALSE | FALSE |
| chr7 | 10917990 | 10918023 | 4 | 3 | L1PA11 | TRUE | NA | 0 | 1 | FALSE | FALSE |
| chr7 | 11920242 | 11920256 | 7 | 4 | (CATATA)n | TRUE | NA | 0 | NA | FALSE | FALSE |
| chr7 | 12566407 | 12566441 | 4 | 3 | NA | FALSE | NA | 0 | NA | FALSE | FALSE |
| chr7 | 12903938 | 12903984 | 4 | 3 | MER52D | TRUE | NA | 0 | 1 | TRUE | FALSE |
| chr7 | 13625938 | 13625973 | 4 | 3 | L1PA10 | TRUE | NA | 0 | 1 | FALSE | FALSE |
| chr7 | 13710445 | 13710471 | 4 | 4 | L1PA7 | TRUE | NA | 0 | 1 | FALSE | FALSE |
| chr7 | 14510891 | 14510922 | 5 | 3 | (TA)n | TRUE | NA | 0 | NA | FALSE | FALSE |
| chr7 | 14532108 | 14532128 | 4 | 3 | NA | FALSE | NA | 0 | 1 | FALSE | FALSE |
| chr7 | 14628209 | 14628232 | 4 | 3 | CT-rich | TRUE | NA | 0 | 1 | FALSE | FALSE |
| chr7 | 14634976 | 14634997 | 4 | 3 | NA | FALSE | NA | 0 | 1 | FALSE | FALSE |
| chr7 | 15112745 | 15112764 | 4 | 4 | NA | FALSE | NA | 0 | 1 | FALSE | FALSE |
| chr7 | 15287079 | 15287114 | 6 | 6 | MANY | TRUE | NA | 0 | 1 | FALSE | FALSE |
| chr7 | 15629065 | 15629085 | 4 | 4 | (CA)n | TRUE | NA | 0 | NA | FALSE | FALSE |
| chr7 | 16685697 | 16685724 | 4 | 4 | NA | FALSE | MANY | 31 | 1 | TRUE | TRUE |
| chr7 | 17083132 | 17083209 | 5 | 4 | (TTCC)n | TRUE | GATA-2 | 1 | NA | TRUE | FALSE |
| chr7 | 18667275 | 18667286 | 4 | 3 | NA | FALSE | NA | 0 | 1 | FALSE | FALSE |
| chr7 | 18891488 | 18891511 | 4 | 4 | NA | FALSE | NA | 0 | 1 | FALSE | FALSE |
| chr7 | 19069756 | 19069767 | 4 | 4 | MLT1L | TRUE | NA | 0 | 1 | FALSE | FALSE |
| chr7 | 19557802 | 19557858 | 8 | 5 | L1PA5 | TRUE | NA | 0 | NA | FALSE | FALSE |
| chr7 | 20570966 | 20570968 | 4 | 3 | NA | FALSE | NA | 0 | 1 | FALSE | FALSE |
| chr7 | 20747458 | 20747489 | 4 | 4 | AluY | TRUE | NA | 0 | NA | FALSE | FALSE |
| chr7 | 21132058 | 21132058 | 4 | 4 | NA | FALSE | NA | 0 | 1 | FALSE | FALSE |
| chr7 | 21260459 | 21260513 | 6 | 5 | NA | FALSE | NA | 0 | 1 | FALSE | FALSE |
| chr7 | 21274876 | 21274877 | 5 | 3 | L1PA5 | TRUE | NA | 0 | NA | FALSE | FALSE |
| chr7 | 21440466 | 21440472 | 4 | 4 | (TG)n | TRUE | NA | 0 | NA | TRUE | FALSE |
| chr7 | 22068996 | 22068998 | 4 | 3 | LTR12C | TRUE | NA | 0 | NA | FALSE | FALSE |
| chr7 | 22375350 | 22375350 | 4 | 4 | (CAA)n | TRUE | NA | 0 | 1 | TRUE | FALSE |
| chr7 | 22564172 | 22564217 | 8 | 8 | ERV3-16A3 LTR | TRUE | NA | 0 | 1 | TRUE | FALSE |
| chr7 | 23053837 | 23053909 | 4 | 3 | NA | FALSE | MANY | 29 | 1 | TRUE | TRUE |
| chr7 | 23591856 | 23591862 | 4 | 3 | NA | FALSE | NA | 0 | 1 | FALSE | FALSE |
| chr7 | 23996781 | 23996803 | 4 | 4 | MamGypLTR1b | TRUE | NA | 0 | 1 | TRUE | FALSE |
| chr7 | 24198394 | 24198401 | 5 | 5 | AluSp | TRUE | NA | 0 | NA | FALSE | FALSE |
| chr7 | 24279361 | 24279373 | 4 | 4 | AluY | TRUE | NA | 0 | NA | FALSE | FALSE |
| chr7 | 24318534 | 24318546 | 4 | 3 | L1PA6 | TRUE | NA | 0 | NA | TRUE | FALSE |
| chr7 | 27079723 | 27079757 | 4 | 2 | L1MB4 | TRUE | NA | 0 | 1 | FALSE | FALSE |
| chr7 | 27202350 | 27202401 | 4 | 4 | NA | FALSE | NA | 0 | 1 | TRUE | FALSE |
| chr7 | 27539176 | 27539189 | 4 | 2 | NA | FALSE | NA | 0 | 1 | TRUE | FALSE |
| chr7 | 27779714 | 27779717 | 4 | 4 | NA | FALSE | MANY | 33 | 1 | TRUE | TRUE |
| chr7 | 27949106 | 27949158 | 4 | 2 | AluSp | TRUE | NA | 0 | NA | FALSE | FALSE |
| chr7 | 28197786 | 28197825 | 4 | 4 | MANY | TRUE | MANY | 2 | NA | TRUE | FALSE |
| chr7 | 28887943 | 28888000 | 4 | 4 | NA | FALSE | NA | 0 | 1 | TRUE | FALSE |
| chr7 | 29672694 | 29672738 | 6 | 5 | NA | FALSE | NA | 0 | 1 | FALSE | FALSE |
| chr7 | 29728720 | 29728756 | 5 | 3 | L2b | TRUE | NA | 0 | 1 | FALSE | FALSE |
| chr7 | 29740103 | 29740191 | 5 | 3 | L1PA3 | TRUE | NA | 0 | NA | FALSE | FALSE |
| chr7 | 29774465 | 29774552 | 4 | 2 | L1ME1 | TRUE | NA | 0 | 1 | FALSE | FALSE |
| chr7 | 29824956 | 29825016 | 5 | 3 | MANY | TRUE | NA | 0 | NA | FALSE | FALSE |
| chr7 | 31546476 | 31546499 | 4 | 3 | L1PA3 | TRUE | NA | 0 | NA | FALSE | FALSE |
| chr7 | 31903675 | 31903683 | 4 | 4 | L3 | TRUE | NA | 0 | 1 | FALSE | FALSE |
| chr7 | 31996011 | 31996013 | 4 | 2 | NA | FALSE | NA | 0 | 1 | TRUE | FALSE |
| chr7 | 32043747 | 32043755 | 4 | 3 | NA | FALSE | NA | 0 | 1 | TRUE | FALSE |
| chr7 | 32075259 | 32075282 | 4 | 4 | NA | FALSE | NA | 0 | 1 | FALSE | FALSE |
| chr7 | 32311991 | 32312003 | 4 | 3 | NA | FALSE | NA | 0 | 1 | FALSE | FALSE |
| chr7 | 32423700 | 32423719 | 4 | 4 | NA | FALSE | NA | 0 | 1 | FALSE | FALSE |
| chr7 | 32469807 | 32469834 | 4 | 4 | NA | FALSE | NA | 0 | 1 | FALSE | FALSE |
| chr7 | 32728916 | 32728945 | 4 | 2 | AluY | TRUE | NA | 0 | 1 | FALSE | FALSE |
| chr7 | 33357800 | 33357879 | 4 | 3 | L2c | TRUE | NA | 0 | 1 | FALSE | FALSE |
| chr7 | 34255267 | 34255275 | 4 | 4 | Arthur1C | TRUE | NA | 0 | 1 | TRUE | FALSE |
| chr7 | 34560345 | 34560347 | 4 | 3 | MER45C | TRUE | NA | 0 | 1 | FALSE | FALSE |
| chr7 | 34629803 | 34629815 | 5 | 5 | NA | FALSE | NA | 0 | 1 | FALSE | FALSE |
| chr7 | 34671984 | 34671998 | 4 | 2 | L1PA4 | TRUE | NA | 0 | NA | FALSE | FALSE |
| chr7 | 34734154 | 34734170 | 4 | 3 | L1PB4 | TRUE | NA | 0 | 1 | FALSE | FALSE |
| chr7 | 34776867 | 34776882 | 4 | 4 | L2a | TRUE | NA | 0 | 1 | FALSE | FALSE |
| chr7 | 35142099 | 35142119 | 5 | 4 | NA | FALSE | NA | 0 | 1 | FALSE | FALSE |
| chr7 | 35161636 | 35161636 | 5 | 5 | NA | FALSE | NA | 0 | NA | FALSE | FALSE |
| chr7 | 35398201 | 35398223 | 4 | 4 | L1M5 | TRUE | NA | 0 | 1 | FALSE | FALSE |
| chr7 | 35543886 | 35543894 | 4 | 2 | MER39B | TRUE | NA | 0 | NA | TRUE | FALSE |
| chr7 | 35974068 | 35974101 | 4 | 3 | NA | FALSE | NA | 0 | 1 | FALSE | FALSE |
| chr7 | 36787941 | 36787984 | 4 | 4 | NA | FALSE | MANY | 2 | 1 | TRUE | FALSE |
| chr7 | 37331440 | 37331467 | 4 | 4 | AluSq | TRUE | NA | 0 | NA | FALSE | FALSE |
| chr7 | 38049093 | 38049110 | 4 | 3 | AluY | TRUE | NA | 0 | NA | FALSE | FALSE |
| chr7 | 38297613 | 38297636 | 6 | 5 | L1M5 | TRUE | NA | 0 | 1 | FALSE | FALSE |
| chr7 | 39253919 | 39253939 | 4 | 3 | L2b | TRUE | NA | 0 | 1 | FALSE | FALSE |
| chr7 | 39605965 | 39605969 | 6 | 5 | NA | FALSE | MANY | 30 | 1 | TRUE | TRUE |
| chr7 | 39840571 | 39840571 | 4 | 4 | AluSz6 | TRUE | NA | 0 | NA | FALSE | FALSE |
| chr7 | 40004227 | 40004251 | 4 | 3 | L1PA4 | TRUE | NA | 0 | NA | FALSE | FALSE |
| chr7 | 40488518 | 40488583 | 13 | 11 | MANY | TRUE | NA | 0 | NA | FALSE | FALSE |
| chr7 | 40958362 | 40958382 | 4 | 3 | NA | FALSE | MANY | 4 | 1 | TRUE | TRUE |
| chr7 | 41121795 | 41121825 | 4 | 2 | NA | FALSE | NA | 0 | 1 | FALSE | FALSE |
| chr7 | 41464622 | 41464628 | 4 | 4 | (TC)n | TRUE | NA | 0 | NA | TRUE | FALSE |
| chr7 | 41861327 | 41861348 | 4 | 4 | (GGAA)n | TRUE | NA | 0 | 1 | TRUE | FALSE |
| chr7 | 41894149 | 41894155 | 4 | 4 | LTR78 | TRUE | NA | 0 | 1 | FALSE | FALSE |
| chr7 | 42311466 | 42311494 | 4 | 3 | NA | FALSE | NA | 0 | 1 | FALSE | FALSE |
| chr7 | 43125512 | 43125536 | 4 | 4 | L1MEc | TRUE | NA | 0 | 1 | FALSE | FALSE |
| chr7 | 43461439 | 43461482 | 4 | 2 | L2 | TRUE | NA | 0 | 1 | TRUE | FALSE |
| chr7 | 43893871 | 43893914 | 4 | 2 | FLAM C | TRUE | NA | 0 | 1 | FALSE | FALSE |
| chr7 | 44003044 | 44003078 | 5 | 5 | AluSg7 | TRUE | NA | 0 | NA | FALSE | FALSE |
| chr7 | 44006934 | 44007030 | 13 | 10 | AluJr | TRUE | NA | 0 | 1 | FALSE | FALSE |
| chr7 | 44010637 | 44010685 | 4 | 4 | MIRc | TRUE | NA | 0 | 1 | TRUE | FALSE |
| chr7 | 44015780 | 44015833 | 5 | 5 | AluJo | TRUE | NA | 0 | 1 | TRUE | FALSE |
| chr7 | 44032914 | 44032930 | 4 | 3 | AluSx | TRUE | NA | 0 | 1 | FALSE | FALSE |
| chr7 | 44033277 | 44033339 | 4 | 4 | AluJr | TRUE | NA | 0 | NA | FALSE | FALSE |
| chr7 | 44062554 | 44062615 | 5 | 3 | AluJo | TRUE | STAT3 | 1 | NA | FALSE | FALSE |
| chr7 | 44073364 | 44073428 | 4 | 4 | AluSx1 | TRUE | NA | 0 | NA | FALSE | FALSE |
| chr7 | 44158192 | 44158235 | 4 | 3 | AluY | TRUE | NA | 0 | NA | FALSE | FALSE |
| chr7 | 44269040 | 44269086 | 4 | 4 | NA | FALSE | NA | 0 | 1 | TRUE | FALSE |
| chr7 | 44301560 | 44301586 | 4 | 3 | NA | FALSE | NA | 0 | 1 | FALSE | FALSE |
| chr7 | 44346634 | 44346664 | 4 | 3 | GA-rich | TRUE | ZNF263 | 1 | 1 | TRUE | FALSE |
| chr7 | 45063397 | 45063424 | 5 | 5 | AluSp | TRUE | NA | 0 | NA | FALSE | FALSE |
| chr7 | 45157592 | 45157637 | 4 | 3 | NA | FALSE | NA | 0 | 1 | FALSE | FALSE |
| chr7 | 45310941 | 45310967 | 4 | 2 | NA | FALSE | NA | 0 | 1 | FALSE | FALSE |
| chr7 | 45472946 | 45472975 | 4 | 3 | C-rich | TRUE | ZNF263 | 1 | 1 | FALSE | FALSE |
| chr7 | 46260447 | 46260488 | 4 | 2 | NA | FALSE | NA | 0 | 1 | TRUE | FALSE |
| chr7 | 48884693 | 48884693 | 5 | 5 | NA | FALSE | NA | 0 | NA | TRUE | FALSE |
| chr7 | 49755832 | 49755832 | 4 | 4 | NA | FALSE | NA | 0 | NA | TRUE | FALSE |
| chr7 | 49876522 | 49876533 | 4 | 3 | NA | FALSE | NA | 0 | 1 | FALSE | FALSE |
| chr7 | 50188137 | 50188165 | 4 | 3 | NA | FALSE | MANY | 6 | 1 | TRUE | TRUE |
| chr7 | 50253166 | 50253199 | 4 | 4 | NA | FALSE | MANY | 4 | 1 | TRUE | TRUE |
| chr7 | 51624549 | 51624553 | 4 | 3 | L1PA6 | TRUE | NA | 0 | NA | FALSE | FALSE |
| chr7 | 51761878 | 51761895 | 4 | 3 | NA | FALSE | NA | 0 | 1 | FALSE | FALSE |
| chr7 | 51913017 | 51913018 | 9 | 5 | MER11A | TRUE | NA | 0 | 1 | FALSE | FALSE |
| chr7 | 51913099 | 51913106 | 4 | 4 | MER11A | TRUE | NA | 0 | NA | TRUE | FALSE |
| chr7 | 51992075 | 51992085 | 4 | 4 | L1PA7 | TRUE | NA | 0 | NA | FALSE | FALSE |
| chr7 | 52098279 | 52098287 | 4 | 3 | L1M5 | TRUE | NA | 0 | 1 | FALSE | FALSE |
| chr7 | 52193736 | 52193736 | 4 | 4 | NA | FALSE | NA | 0 | 1 | FALSE | FALSE |
| chr7 | 52885112 | 52885113 | 4 | 4 | L1MA5 | TRUE | NA | 0 | NA | FALSE | FALSE |
| chr7 | 53092981 | 53092986 | 4 | 2 | (CA)n | TRUE | NA | 0 | NA | FALSE | FALSE |
| chr7 | 53166464 | 53166469 | 4 | 4 | NA | FALSE | NA | 0 | 1 | FALSE | FALSE |
| chr7 | 53265564 | 53265569 | 4 | 3 | NA | FALSE | NA | 0 | 1 | FALSE | FALSE |
| chr7 | 53387025 | 53387026 | 4 | 4 | NA | FALSE | NA | 0 | 1 | FALSE | FALSE |
| chr7 | 53465786 | 53465798 | 4 | 3 | MLT1F2 | TRUE | NA | 0 | 1 | TRUE | FALSE |
| chr7 | 53499302 | 53499302 | 4 | 4 | L1MEf | TRUE | NA | 0 | 1 | FALSE | FALSE |
| chr7 | 53552228 | 53552228 | 4 | 4 | NA | FALSE | NA | 0 | 1 | FALSE | FALSE |
| chr7 | 53991352 | 53991358 | 5 | 5 | NA | FALSE | NA | 0 | 1 | FALSE | FALSE |
| chr7 | 54459947 | 54459948 | 4 | 3 | PABL A-int | TRUE | NA | 0 | 1 | FALSE | FALSE |
| chr7 | 54612811 | 54612816 | 4 | 3 | NA | FALSE | NA | 0 | 1 | TRUE | FALSE |
| chr7 | 54929298 | 54929306 | 4 | 4 | L1M5 | TRUE | NA | 0 | 1 | TRUE | FALSE |
| chr7 | 55399368 | 55399406 | 4 | 4 | MSTD | TRUE | NA | 0 | 1 | FALSE | FALSE |
| chr7 | 55605527 | 55605573 | 5 | 2 | NA | FALSE | NA | 0 | 1 | TRUE | FALSE |
| chr7 | 55678193 | 55678225 | 12 | 6 | L1MA9 | TRUE | NA | 0 | 1 | FALSE | FALSE |
| chr7 | 55686746 | 55686790 | 4 | 4 | THE1C-int | TRUE | NA | 0 | 1 | FALSE | FALSE |
| chr7 | 55741608 | 55741628 | 5 | 3 | L2 | TRUE | NA | 0 | 1 | FALSE | FALSE |
| chr7 | 55809630 | 55809672 | 4 | 3 | NA | FALSE | ERalpha a | 1 | 1 | TRUE | FALSE |
| chr7 | 55821724 | 55821768 | 6 | 3 | MANY | TRUE | NA | 0 | 1 | FALSE | FALSE |
| chr7 | 56174179 | 56174186 | 4 | 3 | NA | FALSE | MANY | 36 | 1 | TRUE | TRUE |
| chr7 | 56258436 | 56258449 | 4 | 3 | NA | FALSE | NA | 0 | 1 | FALSE | FALSE |
| chr7 | 56396319 | 56396338 | 4 | 2 | L2 | TRUE | JunD | 1 | 1 | TRUE | FALSE |
| chr7 | 56679391 | 56679395 | 4 | 2 | BSR/Beta | TRUE | NA | 0 | 1 | FALSE | FALSE |
| chr7 | 57274886 | 57274895 | 4 | 3 | C-rich | TRUE | NA | 0 | NA | FALSE | FALSE |
| chr7 | 57355191 | 57355195 | 4 | 3 | NA | FALSE | NA | 0 | NA | FALSE | FALSE |
| chr7 | 57744315 | 57744328 | 5 | 4 | NA | FALSE | NA | 0 | NA | FALSE | FALSE |
| chr7 | 57745355 | 57745366 | 5 | 3 | MER52A | TRUE | NA | 0 | 1 | FALSE | FALSE |
| chr7 | 57786354 | 57786361 | 4 | 3 | NA | FALSE | NA | 0 | NA | FALSE | FALSE |
| chr7 | 57794454 | 57794455 | 4 | 2 | L1ME3A | TRUE | NA | 0 | 1 | FALSE | FALSE |
| chr7 | 57795281 | 57795281 | 4 | 4 | L1ME3A | TRUE | NA | 0 | NA | FALSE | FALSE |
| chr7 | 57806137 | 57806153 | 6 | 5 | L1ME3B | TRUE | NA | 0 | NA | FALSE | FALSE |
| chr7 | 57810437 | 57810443 | 7 | 3 | L1MC2 | TRUE | NA | 0 | 1 | FALSE | FALSE |
| chr7 | 57847499 | 57847514 | 5 | 3 | AluJb | TRUE | NA | 0 | NA | FALSE | FALSE |
| chr7 | 57874290 | 57874290 | 6 | 6 | THE1A-int | TRUE | NA | 0 | NA | FALSE | FALSE |
| chr7 | 57936907 | 57936915 | 5 | 5 | NA | FALSE | NA | 0 | NA | FALSE | FALSE |
| chr7 | 62624718 | 62624731 | 4 | 2 | AluSc8 | TRUE | NA | 0 | NA | FALSE | FALSE |
| chr7 | 62709799 | 62709806 | 4 | 4 | NA | FALSE | NA | 0 | 1 | FALSE | FALSE |
| chr7 | 62726989 | 62727004 | 6 | 4 | AluY | TRUE | NA | 0 | NA | FALSE | FALSE |
| chr7 | 62932954 | 62932969 | 4 | 4 | AluY | TRUE | NA | 0 | NA | FALSE | FALSE |
| chr7 | 63120895 | 63120898 | 12 | 7 | AluSz | TRUE | NA | 0 | NA | FALSE | FALSE |
| chr7 | 63147867 | 63147867 | 4 | 4 | AluJo | TRUE | NA | 0 | NA | TRUE | FALSE |
| chr7 | 63263454 | 63263474 | 4 | 4 | L1M5 | TRUE | NA | 0 | NA | FALSE | FALSE |
| chr7 | 63295316 | 63295328 | 4 | 4 | NA | FALSE | NA | 0 | 1 | FALSE | FALSE |
| chr7 | 63304437 | 63304457 | 4 | 3 | AluY | TRUE | NA | 0 | NA | FALSE | FALSE |
| chr7 | 63319344 | 63319358 | 4 | 3 | NA | FALSE | NA | 0 | 1 | FALSE | FALSE |
| chr7 | 63386784 | 63386797 | 4 | 3 | NA | FALSE | MANY | 8 | 1 | TRUE | TRUE |
| chr7 | 63418179 | 63418184 | 4 | 3 | AluSg | TRUE | NA | 0 | NA | FALSE | FALSE |
| chr7 | 63504029 | 63504046 | 4 | 3 | NA | FALSE | NA | 0 | 1 | TRUE | FALSE |
| chr7 | 63524226 | 63524236 | 4 | 4 | L1M1 | TRUE | NA | 0 | NA | FALSE | FALSE |
| chr7 | 63667997 | 63668008 | 4 | 3 | MER57E3 | TRUE | NA | 0 | 1 | FALSE | FALSE |
| chr7 | 63719557 | 63719559 | 4 | 3 | AluSx | TRUE | NA | 0 | NA | FALSE | FALSE |
| chr7 | 64188148 | 64188189 | 4 | 4 | L1PB1 | TRUE | NA | 0 | 1 | FALSE | FALSE |
| chr7 | 64350568 | 64350600 | 5 | 4 | (TG)n | TRUE | NA | 0 | NA | FALSE | FALSE |
| chr7 | 64570369 | 64570381 | 5 | 4 | NA | FALSE | NA | 0 | NA | TRUE | FALSE |
| chr7 | 64572288 | 64572309 | 7 | 6 | MLT1D | TRUE | NA | 0 | NA | FALSE | FALSE |
| chr7 | 64718071 | 64718107 | 5 | 4 | SVA C | TRUE | NA | 0 | 1 | FALSE | FALSE |
| chr7 | 64956523 | 64956527 | 6 | 3 | MER77B | TRUE | NA | 0 | NA | FALSE | FALSE |
| chr7 | 64961525 | 64961581 | 7 | 5 | NA | FALSE | NA | 0 | NA | FALSE | FALSE |
| chr7 | 64962931 | 64962973 | 6 | 5 | L1MEe | TRUE | NA | 0 | NA | FALSE | FALSE |
| chr7 | 64963478 | 64963524 | 4 | 3 | L1MEe | TRUE | NA | 0 | 1 | FALSE | FALSE |
| chr7 | 64968091 | 64968111 | 5 | 3 | Tigger2a | TRUE | NA | 0 | 1 | FALSE | FALSE |
| chr7 | 65054248 | 65054255 | 4 | 4 | L1MC | TRUE | NA | 0 | NA | FALSE | FALSE |
| chr7 | 65054624 | 65054656 | 4 | 3 | LTR49 | TRUE | NA | 0 | NA | FALSE | FALSE |
| chr7 | 65054761 | 65054796 | 4 | 4 | LTR49 | TRUE | NA | 0 | 1 | FALSE | FALSE |
| chr7 | 65151398 | 65151487 | 7 | 6 | MIR3 | TRUE | NA | 0 | 1 | FALSE | FALSE |
| chr7 | 65332415 | 65332438 | 4 | 4 | MLT1G3 | TRUE | NA | 0 | 1 | FALSE | FALSE |
| chr7 | 65378728 | 65378745 | 5 | 4 | AluSx1 | TRUE | NA | 0 | 1 | FALSE | FALSE |
| chr7 | 66461639 | 66461702 | 5 | 5 | NA | FALSE | MANY | 14 | 1 | TRUE | TRUE |
| chr7 | 66490318 | 66490402 | 5 | 4 | MER30 | TRUE | NA | 0 | 1 | TRUE | FALSE |
| chr7 | 66670555 | 66670559 | 5 | 5 | L1MDa | TRUE | NA | 0 | 1 | FALSE | FALSE |
| chr7 | 67605151 | 67605169 | 5 | 3 | (GGAA)n | TRUE | NA | 0 | NA | FALSE | FALSE |
| chr7 | 68605936 | 68605959 | 5 | 5 | L1MA9 | TRUE | NA | 0 | 1 | FALSE | FALSE |
| chr7 | 70294320 | 70294330 | 4 | 4 | NA | FALSE | MANY | 15 | 1 | TRUE | TRUE |
| chr7 | 70349128 | 70349133 | 5 | 4 | NA | FALSE | NA | 0 | 1 | FALSE | FALSE |
| chr7 | 70398474 | 70398483 | 4 | 3 | L1MD2 | TRUE | NA | 0 | 1 | FALSE | FALSE |
| chr7 | 70439965 | 70439995 | 7 | 7 | MSTA | TRUE | CHD2 (N-1250) | 1 | 1 | FALSE | FALSE |
| chr7 | 70600499 | 70600519 | 5 | 4 | MIR | TRUE | NA | 0 | 1 | TRUE | FALSE |
| chr7 | 70630678 | 70630678 | 4 | 4 | NA | FALSE | NA | 0 | 1 | FALSE | FALSE |
| chr7 | 70864251 | 70864252 | 4 | 4 | HAL1 | TRUE | NA | 0 | 1 | FALSE | FALSE |
| chr7 | 70980625 | 70980638 | 5 | 3 | NA | FALSE | NA | 0 | 1 | TRUE | FALSE |
| chr7 | 71031605 | 71031613 | 4 | 3 | NA | FALSE | NA | 0 | 1 | TRUE | FALSE |
| chr7 | 71513631 | 71513649 | 5 | 4 | CT-rich | TRUE | NA | 0 | 1 | FALSE | FALSE |
| chr7 | 71601783 | 71601810 | 4 | 4 | Tigger1 | TRUE | NA | 0 | 1 | FALSE | FALSE |
| chr7 | 71872940 | 71872959 | 4 | 3 | NA | FALSE | NA | 0 | 1 | FALSE | FALSE |
| chr7 | 72093250 | 72093254 | 4 | 2 | AluJr | TRUE | NA | 0 | 1 | FALSE | FALSE |
| chr7 | 72113783 | 72113829 | 4 | 3 | NA | FALSE | NA | 0 | 1 | TRUE | FALSE |
| chr7 | 72267019 | 72267104 | 8 | 6 | MER30 | TRUE | NA | 0 | 1 | FALSE | FALSE |
| chr7 | 72298914 | 72299007 | 15 | 9 | NA | FALSE | MANY | 18 | 1 | TRUE | TRUE |
| chr7 | 72695680 | 72695699 | 5 | 5 | AluSx1 | TRUE | NA | 0 | 1 | FALSE | FALSE |
| chr7 | 72737701 | 72737718 | 4 | 3 | L2a | TRUE | MANY | 2 | 1 | FALSE | FALSE |
| chr7 | 73577819 | 73577852 | 4 | 2 | AluJo | TRUE | NA | 0 | 1 | FALSE | FALSE |
| chr7 | 73708333 | 73708392 | 4 | 2 | AluSq | TRUE | NA | 0 | NA | FALSE | FALSE |
| chr7 | 74051223 | 74051314 | 5 | 3 | (TTTA)n | TRUE | NA | 0 | 1 | TRUE | FALSE |
| chr7 | 74326711 | 74326794 | 4 | 3 | AluSx | TRUE | NA | 0 | 1 | FALSE | FALSE |
| chr7 | 74379989 | 74380026 | 4 | 4 | (TC)n | TRUE | NA | 0 | NA | FALSE | FALSE |
| chr7 | 74566675 | 74566839 | 7 | 7 | MANY | TRUE | NA | 0 | 1 | FALSE | FALSE |
| chr7 | 74579271 | 74579351 | 4 | 4 | MANY | TRUE | NA | 0 | NA | FALSE | FALSE |
| chr7 | 75069405 | 75069483 | 5 | 5 | AluSx1 | TRUE | NA | 0 | 1 | FALSE | FALSE |
| chr7 | 75395358 | 75395360 | 4 | 3 | L1MA9 | TRUE | NA | 0 | NA | FALSE | FALSE |
| chr7 | 75496570 | 75496606 | 4 | 4 | (TC)n | TRUE | MANY | 3 | NA | TRUE | FALSE |
| chr7 | 75623974 | 75624038 | 4 | 4 | NA | FALSE | MANY | 41 | 1 | TRUE | TRUE |
| chr7 | 75642637 | 75642644 | 4 | 2 | AluY | TRUE | NA | 0 | NA | FALSE | FALSE |
| chr7 | 75795924 | 75795947 | 6 | 4 | NA | FALSE | MANY | 39 | 1 | TRUE | TRUE |
| chr7 | 75889709 | 75889763 | 4 | 4 | NA | FALSE | MANY | 2 | 1 | TRUE | FALSE |
| chr7 | 76005628 | 76005661 | 4 | 3 | NA | FALSE | NA | 0 | 1 | FALSE | FALSE |
| chr7 | 76559620 | 76559622 | 4 | 3 | L1MC | TRUE | NA | 0 | 1 | FALSE | FALSE |
| chr7 | 76628575 | 76628626 | 4 | 3 | NA | FALSE | NA | 0 | 1 | TRUE | FALSE |
| chr7 | 76637708 | 76637753 | 4 | 4 | AluSq | TRUE | NA | 0 | NA | FALSE | FALSE |
| chr7 | 76655188 | 76655188 | 4 | 4 | AluJb | TRUE | NA | 0 | NA | FALSE | FALSE |
| chr7 | 76733549 | 76733579 | 4 | 2 | AluSq10 | TRUE | NA | 0 | 1 | FALSE | FALSE |
| chr7 | 76771451 | 76771508 | 5 | 4 | L1P3 | TRUE | NA | 0 | 1 | TRUE | FALSE |
| chr7 | 76970082 | 76970083 | 4 | 2 | HERVH-int | TRUE | NA | 0 | NA | FALSE | FALSE |
| chr7 | 77163093 | 77163169 | 4 | 3 | SVA D | TRUE | NA | 0 | NA | FALSE | FALSE |
| chr7 | 77957847 | 77957850 | 4 | 3 | (CA)n | TRUE | NA | 0 | NA | FALSE | FALSE |
| chr7 | 78298950 | 78299009 | 8 | 6 | L1PA3 | TRUE | NA | 0 | NA | FALSE | FALSE |
| chr7 | 78933508 | 78933528 | 4 | 4 | (TG)n | TRUE | NA | 0 | NA | FALSE | FALSE |
| chr7 | 80889895 | 80889904 | 4 | 3 | L1MCa | TRUE | NA | 0 | 1 | FALSE | FALSE |
| chr7 | 81139107 | 81139130 | 5 | 2 | THE1A-int | TRUE | NA | 0 | 1 | FALSE | FALSE |
| chr7 | 82719112 | 82719135 | 4 | 3 | NA | FALSE | NA | 0 | 1 | FALSE | FALSE |
| chr7 | 82891470 | 82891471 | 4 | 3 | NA | FALSE | NA | 0 | 1 | FALSE | FALSE |
| chr7 | 82980155 | 82980177 | 4 | 3 | NA | FALSE | NA | 0 | 1 | FALSE | FALSE |
| chr7 | 83523992 | 83524038 | 4 | 3 | NA | FALSE | NA | 0 | 1 | FALSE | FALSE |
| chr7 | 85563094 | 85563109 | 5 | 4 | THE1D | TRUE | NA | 0 | NA | FALSE | FALSE |
| chr7 | 85628180 | 85628187 | 4 | 3 | L1MA3 | TRUE | NA | 0 | 1 | TRUE | FALSE |
| chr7 | 85702159 | 85702166 | 4 | 4 | L1MA9 | TRUE | NA | 0 | 1 | FALSE | FALSE |
| chr7 | 85721735 | 85721749 | 4 | 3 | HERVL-int | TRUE | NA | 0 | NA | FALSE | FALSE |
| chr7 | 85781738 | 85781752 | 4 | 3 | THE1C-int | TRUE | NA | 0 | 1 | FALSE | FALSE |
| chr7 | 85791390 | 85791402 | 4 | 4 | L1PA8A | TRUE | NA | 0 | NA | FALSE | FALSE |
| chr7 | 85846132 | 85846148 | 4 | 3 | NA | FALSE | NA | 0 | 1 | FALSE | FALSE |
| chr7 | 86021135 | 86021144 | 4 | 4 | THE1B-int | TRUE | NA | 0 | 1 | FALSE | FALSE |
| chr7 | 86025944 | 86025944 | 4 | 4 | NA | FALSE | NA | 0 | 1 | FALSE | FALSE |
| chr7 | 86055483 | 86055503 | 5 | 4 | NA | FALSE | NA | 0 | 1 | FALSE | FALSE |
| chr7 | 86076194 | 86076205 | 4 | 3 | AluJb | TRUE | NA | 0 | 1 | FALSE | FALSE |
| chr7 | 86165833 | 86165849 | 4 | 4 | MER50 | TRUE | NA | 0 | 1 | FALSE | FALSE |
| chr7 | 86386075 | 86386091 | 5 | 4 | L1MEf | TRUE | NA | 0 | 1 | FALSE | FALSE |
| chr7 | 86874901 | 86874939 | 5 | 5 | THE1A-int | TRUE | NA | 0 | 1 | FALSE | FALSE |
| chr7 | 86928999 | 86929026 | 5 | 2 | THE1B-int | TRUE | NA | 0 | 1 | FALSE | FALSE |
| chr7 | 87283030 | 87283047 | 4 | 4 | L3 | TRUE | NA | 0 | 1 | FALSE | FALSE |
| chr7 | 87367034 | 87367039 | 4 | 4 | NA | FALSE | NA | 0 | 1 | FALSE | FALSE |
| chr7 | 87792310 | 87792331 | 4 | 2 | NA | FALSE | NA | 0 | 1 | TRUE | FALSE |
| chr7 | 89072545 | 89072546 | 4 | 2 | L1PA4 | TRUE | NA | 0 | NA | FALSE | FALSE |
| chr7 | 89162190 | 89162190 | 7 | 7 | (TG)n | TRUE | NA | 0 | NA | FALSE | FALSE |
| chr7 | 89254956 | 89254985 | 4 | 3 | HERVIP10FH-int | TRUE | NA | 0 | NA | FALSE | FALSE |
| chr7 | 89579312 | 89579312 | 4 | 4 | NA | FALSE | NA | 0 | 1 | FALSE | FALSE |
| chr7 | 89707183 | 89707214 | 4 | 2 | (TC)n | TRUE | NA | 0 | NA | FALSE | FALSE |
| chr7 | 90507407 | 90507428 | 9 | 6 | MANY | TRUE | NA | 0 | NA | FALSE | FALSE |
| chr7 | 90733156 | 90733183 | 4 | 4 | (GA)n | TRUE | NA | 0 | 1 | FALSE | FALSE |
| chr7 | 91071612 | 91071635 | 5 | 4 | MANY | TRUE | NA | 0 | 1 | FALSE | FALSE |
| chr7 | 92091022 | 92091051 | 4 | 3 | MLT1F | TRUE | MANY | 9 | 1 | TRUE | TRUE |
| chr7 | 93071420 | 93071430 | 4 | 4 | NA | FALSE | NA | 0 | 1 | FALSE | FALSE |
| chr7 | 93079992 | 93080017 | 4 | 3 | L1MEb | TRUE | NA | 0 | 1 | FALSE | FALSE |
| chr7 | 93144515 | 93144542 | 4 | 3 | NA | FALSE | RFX5 (N-494) | 1 | 1 | TRUE | FALSE |
| chr7 | 93790983 | 93790984 | 4 | 2 | NA | FALSE | NA | 0 | 1 | TRUE | FALSE |
| chr7 | 94795400 | 94795426 | 4 | 3 | AluSq2 | TRUE | NA | 0 | NA | FALSE | FALSE |
| chr7 | 94856128 | 94856149 | 4 | 2 | NA | FALSE | NA | 0 | 1 | FALSE | FALSE |
| chr7 | 95692410 | 95692420 | 5 | 5 | (TG)n | TRUE | NA | 0 | NA | FALSE | FALSE |
| chr7 | 96212343 | 96212397 | 4 | 4 | L1PA3 | TRUE | NA | 0 | NA | FALSE | FALSE |
| chr7 | 96956806 | 96956824 | 5 | 4 | LTR1B | TRUE | NA | 0 | NA | TRUE | FALSE |
| chr7 | 97083751 | 97083775 | 4 | 1 | MLT1A0 | TRUE | NA | 0 | 1 | TRUE | FALSE |
| chr7 | 97334295 | 97334307 | 5 | 5 | L1PREC2 | TRUE | NA | 0 | 1 | FALSE | FALSE |
| chr7 | 97586562 | 97586587 | 4 | 3 | SATR1 | TRUE | NA | 0 | 1 | FALSE | FALSE |
| chr7 | 97602345 | 97602347 | 4 | 3 | MANY | TRUE | NA | 0 | NA | FALSE | FALSE |
| chr7 | 98232357 | 98232383 | 8 | 8 | MANY | TRUE | NA | 0 | NA | FALSE | FALSE |
| chr7 | 98320430 | 98320456 | 5 | 4 | (TTCC)n | TRUE | NA | 0 | NA | TRUE | FALSE |
| chr7 | 98363505 | 98363546 | 4 | 2 | NA | FALSE | NA | 0 | 1 | TRUE | FALSE |
| chr7 | 98778515 | 98778537 | 4 | 4 | CT-rich | TRUE | NA | 0 | 1 | FALSE | FALSE |
| chr7 | 99550410 | 99550479 | 4 | 3 | AluYc | TRUE | NA | 0 | NA | FALSE | FALSE |
| chr7 | 99756350 | 99756351 | 5 | 4 | NA | FALSE | MANY | 21 | 1 | TRUE | TRUE |
| chr7 | 99795227 | 99795239 | 12 | 9 | NA | FALSE | NA | 0 | 1 | TRUE | FALSE |
| chr7 | 99845418 | 99845449 | 4 | 2 | NA | FALSE | TFIIIC-110 | 1 | 1 | FALSE | FALSE |
| chr7 | 99856475 | 99856487 | 8 | 6 | NA | FALSE | NA | 0 | 1 | FALSE | FALSE |
| chr7 | 99883258 | 99883308 | 4 | 3 | NA | FALSE | NA | 0 | 1 | FALSE | FALSE |
| chr7 | 99901846 | 99901884 | 5 | 5 | AluSq2 | TRUE | NA | 0 | NA | TRUE | FALSE |
| chr7 | 99924812 | 99924849 | 8 | 7 | LTR45B | TRUE | NA | 0 | 1 | TRUE | FALSE |
| chr7 | 99932102 | 99932168 | 5 | 4 | L2c | TRUE | NA | 0 | 1 | FALSE | FALSE |
| chr7 | 100056314 | 100056314 | 6 | 6 | AluSq | TRUE | NA | 0 | NA | FALSE | FALSE |
| chr7 | 100121075 | 100121142 | 5 | 4 | NA | FALSE | NA | 0 | 1 | TRUE | FALSE |
| chr7 | 100210059 | 100210060 | 4 | 4 | NA | FALSE | MANY | 23 | 1 | TRUE | TRUE |
| chr7 | 100661308 | 100661308 | 4 | 4 | C-rich | TRUE | NA | 0 | NA | FALSE | FALSE |
| chr7 | 100686969 | 100687013 | 6 | 3 | NA | FALSE | NA | 0 | 1 | FALSE | FALSE |
| chr7 | 100908752 | 100908778 | 5 | 2 | NA | FALSE | NA | 0 | NA | TRUE | FALSE |
| chr7 | 101003660 | 101003677 | 4 | 4 | SVA D | TRUE | NA | 0 | NA | FALSE | FALSE |
| chr7 | 101067853 | 101067907 | 4 | 3 | AluSx | TRUE | NA | 0 | 1 | TRUE | FALSE |
| chr7 | 101981616 | 101981732 | 10 | 7 | MANY | TRUE | NA | 0 | 1 | TRUE | FALSE |
| chr7 | 101998831 | 101998910 | 4 | 4 | L3 | TRUE | NA | 0 | 1 | FALSE | FALSE |
| chr7 | 102105379 | 102105458 | 7 | 5 | NA | FALSE | MANY | 44 | 1 | TRUE | TRUE |
| chr7 | 102213039 | 102213106 | 8 | 7 | NA | FALSE | MANY | 21 | NA | TRUE | FALSE |
| chr7 | 102325348 | 102325374 | 4 | 4 | MANY | TRUE | NA | 0 | NA | FALSE | FALSE |
| chr7 | 102403098 | 102403132 | 4 | 4 | NA | FALSE | NA | 0 | 1 | TRUE | FALSE |
| chr7 | 102895893 | 102895977 | 5 | 3 | MIR | TRUE | NA | 0 | 1 | FALSE | FALSE |
| chr7 | 103803593 | 103803620 | 4 | 2 | MANY | TRUE | NA | 0 | 1 | TRUE | FALSE |
| chr7 | 103922213 | 103922215 | 4 | 4 | L1PA3 | TRUE | NA | 0 | NA | FALSE | FALSE |
| chr7 | 103997994 | 103998026 | 4 | 3 | MLT2C1 | TRUE | NA | 0 | 1 | FALSE | FALSE |
| chr7 | 105084954 | 105085004 | 4 | 4 | L1MB8 | TRUE | PU.1 | 1 | 1 | TRUE | FALSE |
| chr7 | 106371385 | 106371444 | 4 | 4 | LTR67B | TRUE | NA | 0 | 1 | TRUE | FALSE |
| chr7 | 107804549 | 107804614 | 4 | 4 | (GGGTG)n | TRUE | NA | 0 | NA | FALSE | FALSE |
| chr7 | 107896329 | 107896406 | 7 | 5 | SVA B | TRUE | NA | 0 | NA | FALSE | FALSE |
| chr7 | 108356852 | 108356889 | 5 | 4 | NA | FALSE | NA | 0 | 1 | TRUE | FALSE |
| chr7 | 108370168 | 108370205 | 9 | 7 | L1PA4 | TRUE | NA | 0 | NA | FALSE | FALSE |
| chr7 | 108395662 | 108395692 | 4 | 2 | L1PA13 | TRUE | NA | 0 | 1 | FALSE | FALSE |
| chr7 | 109016101 | 109016109 | 4 | 3 | NA | FALSE | NA | 0 | 1 | FALSE | FALSE |
| chr7 | 109047327 | 109047343 | 4 | 3 | NA | FALSE | NA | 0 | 1 | FALSE | FALSE |
| chr7 | 109653482 | 109653498 | 4 | 3 | NA | FALSE | NA | 0 | 1 | FALSE | FALSE |
| chr7 | 111250190 | 111250253 | 4 | 4 | SVA D | TRUE | NA | 0 | NA | FALSE | FALSE |
| chr7 | 111846577 | 111846617 | 5 | 3 | NA | FALSE | MANY | 26 | 1 | TRUE | TRUE |
| chr7 | 112090436 | 112090486 | 5 | 5 | NA | FALSE | MANY | 50 | 1 | TRUE | TRUE |
| chr7 | 112185473 | 112185507 | 4 | 2 | L1PA10 | TRUE | NA | 0 | NA | FALSE | FALSE |
| chr7 | 112189474 | 112189525 | 4 | 3 | AT rich | TRUE | NA | 0 | 1 | FALSE | FALSE |
| chr7 | 113007354 | 113007369 | 4 | 4 | NA | FALSE | NA | 0 | 1 | TRUE | FALSE |
| chr7 | 113159103 | 113159121 | 4 | 3 | NA | FALSE | NA | 0 | 1 | FALSE | FALSE |
| chr7 | 113258342 | 113258358 | 4 | 4 | MER4-int | TRUE | NA | 0 | NA | FALSE | FALSE |
| chr7 | 113304305 | 113304317 | 4 | 4 | NA | FALSE | NA | 0 | 1 | FALSE | FALSE |
| chr7 | 114529253 | 114529281 | 5 | 5 | (TTCC)n | TRUE | NA | 0 | NA | TRUE | FALSE |
| chr7 | 115281559 | 115281560 | 4 | 3 | NA | FALSE | NA | 0 | 1 | FALSE | FALSE |
| chr7 | 115539196 | 115539222 | 5 | 4 | L1PA15 | TRUE | NA | 0 | NA | FALSE | FALSE |
| chr7 | 115611002 | 115611029 | 4 | 3 | MSTB1 | TRUE | NA | 0 | 1 | FALSE | FALSE |
| chr7 | 115654384 | 115654394 | 4 | 4 | NA | FALSE | NA | 0 | 1 | FALSE | FALSE |
| chr7 | 115688598 | 115688622 | 4 | 2 | L1PA5 | TRUE | NA | 0 | NA | FALSE | FALSE |
| chr7 | 115932351 | 115932368 | 4 | 4 | (GA)n | TRUE | NA | 0 | NA | FALSE | FALSE |
| chr7 | 116297668 | 116297734 | 4 | 2 | NA | FALSE | NA | 0 | 1 | FALSE | FALSE |
| chr7 | 116491653 | 116491711 | 4 | 4 | AluJr | TRUE | NA | 0 | 1 | FALSE | FALSE |
| chr7 | 117003504 | 117003534 | 4 | 4 | NA | FALSE | NA | 0 | 1 | FALSE | FALSE |
| chr7 | 117200816 | 117200853 | 4 | 4 | NA | FALSE | NA | 0 | 1 | FALSE | FALSE |
| chr7 | 118180318 | 118180344 | 4 | 3 | MER67C | TRUE | NA | 0 | 1 | FALSE | FALSE |
| chr7 | 118280016 | 118280024 | 4 | 3 | (T)n | TRUE | NA | 0 | NA | FALSE | FALSE |
| chr7 | 118571851 | 118571866 | 4 | 4 | NA | FALSE | NA | 0 | 1 | FALSE | FALSE |
| chr7 | 119036143 | 119036155 | 4 | 4 | L1MD2 | TRUE | NA | 0 | 1 | TRUE | FALSE |
| chr7 | 119147414 | 119147423 | 4 | 3 | L1MA3 | TRUE | NA | 0 | 1 | FALSE | FALSE |
| chr7 | 119246998 | 119247010 | 4 | 4 | L1MCc | TRUE | NA | 0 | 1 | FALSE | FALSE |
| chr7 | 119608338 | 119608350 | 4 | 3 | NA | FALSE | NA | 0 | 1 | FALSE | FALSE |
| chr7 | 119637417 | 119637430 | 4 | 4 | NA | FALSE | NA | 0 | 1 | FALSE | FALSE |
| chr7 | 119796061 | 119796074 | 5 | 5 | L1MC | TRUE | NA | 0 | 1 | FALSE | FALSE |
| chr7 | 120130960 | 120130960 | 4 | 4 | NA | FALSE | NA | 0 | 1 | FALSE | FALSE |
| chr7 | 120196266 | 120196271 | 4 | 3 | NA | FALSE | NA | 0 | 1 | FALSE | FALSE |
| chr7 | 120215583 | 120215601 | 4 | 4 | MLT2A2 | TRUE | NA | 0 | 1 | FALSE | FALSE |
| chr7 | 120590715 | 120590743 | 6 | 6 | NA | FALSE | MANY | 30 | 1 | TRUE | TRUE |
| chr7 | 120591171 | 120591192 | 4 | 3 | NA | FALSE | MANY | 42 | 1 | TRUE | TRUE |
| chr7 | 121452963 | 121452988 | 4 | 4 | (TG)n | TRUE | NA | 0 | 1 | FALSE | FALSE |
| chr7 | 121926271 | 121926313 | 4 | 2 | Tigger3a | TRUE | NA | 0 | 1 | FALSE | FALSE |
| chr7 | 121928631 | 121928675 | 4 | 4 | MANY | TRUE | NA | 0 | 1 | FALSE | FALSE |
| chr7 | 122689069 | 122689101 | 4 | 3 | THE1B | TRUE | NA | 0 | NA | FALSE | FALSE |
| chr7 | 122962993 | 122963013 | 7 | 5 | MANY | TRUE | NA | 0 | NA | FALSE | FALSE |
| chr7 | 123879083 | 123879088 | 4 | 2 | L4 | TRUE | NA | 0 | 1 | FALSE | FALSE |
| chr7 | 125696379 | 125696396 | 4 | 4 | L1MA3 | TRUE | NA | 0 | 1 | FALSE | FALSE |
| chr7 | 125932421 | 125932425 | 4 | 3 | L1PA8A | TRUE | NA | 0 | 1 | TRUE | FALSE |
| chr7 | 125973144 | 125973157 | 4 | 4 | NA | FALSE | NA | 0 | 1 | FALSE | FALSE |
| chr7 | 126058729 | 126058730 | 4 | 3 | NA | FALSE | NA | 0 | 1 | FALSE | FALSE |
| chr7 | 126112814 | 126112826 | 4 | 3 | THE1C-int | TRUE | NA | 0 | 1 | FALSE | FALSE |
| chr7 | 126252114 | 126252114 | 4 | 4 | MIR | TRUE | NA | 0 | 1 | FALSE | FALSE |
| chr7 | 126573436 | 126573436 | 4 | 4 | NA | FALSE | NA | 0 | 1 | FALSE | FALSE |
| chr7 | 126669600 | 126669610 | 7 | 4 | L1PA6 | TRUE | NA | 0 | NA | FALSE | FALSE |
| chr7 | 127018785 | 127018785 | 7 | 7 | NA | FALSE | NA | 0 | NA | TRUE | FALSE |
| chr7 | 127843591 | 127843670 | 5 | 3 | C-rich | TRUE | NA | 0 | 1 | TRUE | FALSE |
| chr7 | 127968557 | 127968562 | 4 | 2 | CR1 Mam | TRUE | NA | 0 | 1 | FALSE | FALSE |
| chr7 | 128062367 | 128062386 | 4 | 4 | NA | FALSE | NA | 0 | 1 | TRUE | FALSE |
| chr7 | 128216670 | 128216714 | 5 | 4 | SVA D | TRUE | NA | 0 | NA | FALSE | FALSE |
| chr7 | 128258533 | 128258601 | 7 | 5 | NA | FALSE | NA | 0 | 1 | FALSE | FALSE |
| chr7 | 128262390 | 128262566 | 12 | 8 | MANY | TRUE | NA | 0 | 1 | FALSE | FALSE |
| chr7 | 128882923 | 128882956 | 4 | 3 | AluSg4 | TRUE | NA | 0 | NA | FALSE | FALSE |
| chr7 | 129089992 | 129090046 | 4 | 2 | HERVL-int | TRUE | NA | 0 | 1 | FALSE | FALSE |
| chr7 | 129195284 | 129195320 | 4 | 2 | AluY | TRUE | NA | 0 | NA | FALSE | FALSE |
| chr7 | 129195423 | 129195486 | 4 | 3 | AluY | TRUE | NA | 0 | NA | FALSE | FALSE |
| chr7 | 129672556 | 129672564 | 4 | 3 | GA-rich | TRUE | NA | 0 | NA | FALSE | FALSE |
| chr7 | 130275753 | 130275819 | 4 | 3 | SVA F | TRUE | NA | 0 | NA | FALSE | FALSE |
| chr7 | 130375207 | 130375235 | 4 | 4 | (TTTC)n | TRUE | NA | 0 | NA | FALSE | FALSE |
| chr7 | 131419294 | 131419317 | 4 | 4 | (CA)n | TRUE | NA | 0 | NA | TRUE | FALSE |
| chr7 | 131479772 | 131479777 | 4 | 3 | NA | FALSE | NA | 0 | 1 | TRUE | FALSE |
| chr7 | 131976025 | 131976044 | 4 | 3 | L2a | TRUE | NA | 0 | 1 | FALSE | FALSE |
| chr7 | 132379873 | 132379905 | 4 | 3 | MIRb | TRUE | NA | 0 | 1 | FALSE | FALSE |
| chr7 | 132937628 | 132937708 | 4 | 4 | NA | FALSE | MANY | 27 | 1 | TRUE | TRUE |
| chr7 | 133368991 | 133369035 | 4 | 3 | NA | FALSE | NA | 0 | 1 | FALSE | FALSE |
| chr7 | 133916328 | 133916474 | 11 | 10 | MANY | TRUE | ZNF263 | 1 | 1 | FALSE | FALSE |
| chr7 | 133943124 | 133943150 | 4 | 2 | NA | FALSE | NA | 0 | 1 | FALSE | FALSE |
| chr7 | 134264747 | 134264776 | 4 | 3 | MIR | TRUE | NA | 0 | 1 | TRUE | FALSE |
| chr7 | 134982040 | 134982090 | 4 | 3 | L1PA8 | TRUE | NA | 0 | 1 | FALSE | FALSE |
| chr7 | 135032017 | 135032019 | 5 | 3 | (CA)n | TRUE | NA | 0 | NA | FALSE | FALSE |
| chr7 | 135753655 | 135753687 | 4 | 4 | NA | FALSE | NA | 0 | 1 | TRUE | FALSE |
| chr7 | 135812772 | 135812798 | 5 | 4 | MIR3 | TRUE | NA | 0 | 1 | FALSE | FALSE |
| chr7 | 135919381 | 135919392 | 4 | 3 | NA | FALSE | NA | 0 | 1 | FALSE | FALSE |
| chr7 | 136124371 | 136124389 | 5 | 4 | L1PA10 | TRUE | NA | 0 | 1 | FALSE | FALSE |
| chr7 | 136780301 | 136780326 | 4 | 4 | L1PA15 | TRUE | NA | 0 | 1 | FALSE | FALSE |
| chr7 | 137114854 | 137114862 | 4 | 2 | L1PA3 | TRUE | NA | 0 | NA | FALSE | FALSE |
| chr7 | 137794359 | 137794388 | 4 | 4 | NA | FALSE | NA | 0 | 1 | FALSE | FALSE |
| chr7 | 138144801 | 138144808 | 4 | 3 | NA | FALSE | MANY | 13 | 1 | TRUE | TRUE |
| chr7 | 139172517 | 139172553 | 5 | 4 | G-rich | TRUE | NA | 0 | 1 | TRUE | FALSE |
| chr7 | 140181734 | 140181739 | 4 | 3 | AluSc | TRUE | NA | 0 | NA | FALSE | FALSE |
| chr7 | 140358938 | 140358956 | 4 | 3 | HERV3-int | TRUE | NA | 0 | 1 | FALSE | FALSE |
| chr7 | 140453133 | 140453144 | 23 | 19 | NA | FALSE | NA | 0 | 1 | FALSE | FALSE |
| chr7 | 140696747 | 140696766 | 4 | 2 | L1M4c | TRUE | NA | 0 | 1 | FALSE | FALSE |
| chr7 | 140714738 | 140714752 | 8 | 5 | NA | FALSE | MANY | 49 | 1 | TRUE | TRUE |
| chr7 | 141451447 | 141451469 | 7 | 4 | LTR5 Hs | TRUE | NA | 0 | NA | FALSE | FALSE |
| chr7 | 141731800 | 141731819 | 4 | 3 | NA | FALSE | NA | 0 | 1 | FALSE | FALSE |
| chr7 | 141935477 | 141935485 | 5 | 5 | L1PA13 | TRUE | NA | 0 | 1 | FALSE | FALSE |
| chr7 | 142101517 | 142101518 | 4 | 3 | NA | FALSE | NA | 0 | 1 | FALSE | FALSE |
| chr7 | 142161587 | 142161603 | 4 | 4 | NA | FALSE | NA | 0 | 1 | TRUE | FALSE |
| chr7 | 142205659 | 142205660 | 4 | 3 | NA | FALSE | NA | 0 | 1 | FALSE | FALSE |
| chr7 | 142213210 | 142213216 | 4 | 4 | NA | FALSE | NA | 0 | 1 | TRUE | FALSE |
| chr7 | 142650808 | 142650828 | 4 | 3 | NA | FALSE | NA | 0 | 1 | FALSE | FALSE |
| chr7 | 142775676 | 142775683 | 4 | 3 | L1PB1 | TRUE | NA | 0 | NA | FALSE | FALSE |
| chr7 | 143721208 | 143721211 | 4 | 2 | NA | FALSE | NA | 0 | 1 | FALSE | FALSE |
| chr7 | 143755810 | 143755847 | 4 | 3 | AluSx4 | TRUE | NA | 0 | 1 | FALSE | FALSE |
| chr7 | 144617182 | 144617194 | 5 | 3 | L1PA5 | TRUE | NA | 0 | 1 | FALSE | FALSE |
| chr7 | 144835562 | 144835581 | 4 | 3 | NA | FALSE | NA | 0 | 1 | FALSE | FALSE |
| chr7 | 145193983 | 145194000 | 4 | 3 | NA | FALSE | NA | 0 | 1 | FALSE | FALSE |
| chr7 | 145243779 | 145243781 | 4 | 4 | NA | FALSE | NA | 0 | 1 | FALSE | FALSE |
| chr7 | 145334241 | 145334254 | 5 | 5 | NA | FALSE | NA | 0 | 1 | FALSE | FALSE |
| chr7 | 145488821 | 145488833 | 4 | 4 | L1MC3 | TRUE | NA | 0 | 1 | FALSE | FALSE |
| chr7 | 145741828 | 145741841 | 4 | 4 | L1PA4 | TRUE | NA | 0 | NA | FALSE | FALSE |
| chr7 | 145970909 | 145970916 | 4 | 4 | MIRc | TRUE | NA | 0 | 1 | FALSE | FALSE |
| chr7 | 146167162 | 146167162 | 4 | 4 | AluY | TRUE | NA | 0 | NA | FALSE | FALSE |
| chr7 | 146190681 | 146190682 | 4 | 4 | NA | FALSE | NA | 0 | 1 | FALSE | FALSE |
| chr7 | 146409112 | 146409112 | 4 | 4 | (TA)n | TRUE | NA | 0 | NA | FALSE | FALSE |
| chr7 | 146409139 | 146409139 | 6 | 6 | (TA)n | TRUE | NA | 0 | NA | FALSE | FALSE |
| chr7 | 146409166 | 146409170 | 6 | 6 | (TA)n | TRUE | NA | 0 | NA | FALSE | FALSE |
| chr7 | 146526213 | 146526223 | 4 | 3 | NA | FALSE | NA | 0 | 1 | FALSE | FALSE |
| chr7 | 146600313 | 146600323 | 4 | 3 | NA | FALSE | NA | 0 | 1 | FALSE | FALSE |
| chr7 | 146707371 | 146707371 | 4 | 4 | AluSx | TRUE | NA | 0 | NA | FALSE | FALSE |
| chr7 | 146834421 | 146834433 | 5 | 5 | NA | FALSE | NA | 0 | 1 | FALSE | FALSE |
| chr7 | 146849082 | 146849083 | 4 | 4 | NA | FALSE | NA | 0 | 1 | FALSE | FALSE |
| chr7 | 146922885 | 146922890 | 4 | 3 | (GGAA)n | TRUE | NA | 0 | NA | FALSE | FALSE |
| chr7 | 148214599 | 148214626 | 4 | 3 | L1PA8 | TRUE | NA | 0 | 1 | FALSE | FALSE |
| chr7 | 148697442 | 148697482 | 4 | 2 | NA | FALSE | NA | 0 | 1 | TRUE | FALSE |
| chr7 | 149646614 | 149646624 | 5 | 5 | NA | FALSE | NA | 0 | 1 | TRUE | FALSE |
| chr7 | 149714152 | 149714179 | 5 | 3 | NA | FALSE | NA | 0 | 1 | TRUE | FALSE |
| chr7 | 149728819 | 149728832 | 4 | 2 | NA | FALSE | NA | 0 | 1 | TRUE | FALSE |
| chr7 | 149733125 | 149733127 | 4 | 3 | NA | FALSE | NA | 0 | 1 | FALSE | FALSE |
| chr7 | 149739473 | 149739506 | 4 | 4 | NA | FALSE | NA | 0 | 1 | TRUE | FALSE |
| chr7 | 150239137 | 150239145 | 5 | 4 | NA | FALSE | NA | 0 | 1 | FALSE | FALSE |
| chr7 | 150300939 | 150300965 | 4 | 4 | NA | FALSE | NA | 0 | 1 | TRUE | FALSE |
| chr7 | 150676012 | 150676026 | 4 | 4 | (CA)n | TRUE | MANY | 3 | NA | TRUE | FALSE |
| chr7 | 151038718 | 151038779 | 5 | 5 | (CCCCG)n | TRUE | MANY | 14 | 1 | TRUE | TRUE |
| chr7 | 151916243 | 151916286 | 5 | 2 | AluSz | TRUE | NA | 0 | 1 | FALSE | FALSE |
| chr7 | 151932996 | 151933013 | 4 | 3 | NA | FALSE | NA | 0 | 1 | FALSE | FALSE |
| chr7 | 151935310 | 151935361 | 4 | 4 | NA | FALSE | NA | 0 | NA | FALSE | FALSE |
| chr7 | 151945827 | 151945866 | 6 | 5 | NA | FALSE | NA | 0 | 1 | FALSE | FALSE |
| chr7 | 151961455 | 151961464 | 9 | 6 | NA | FALSE | NA | 0 | NA | TRUE | FALSE |
| chr7 | 151965237 | 151965266 | 4 | 4 | AluSz | TRUE | NA | 0 | NA | FALSE | FALSE |
| chr7 | 151988115 | 151988207 | 7 | 6 | Cheshire | TRUE | NA | 0 | 1 | TRUE | FALSE |
| chr7 | 152073958 | 152073996 | 4 | 4 | Tigger1 | TRUE | MANY | 3 | NA | TRUE | FALSE |
| chr7 | 152078725 | 152078765 | 6 | 6 | NA | FALSE | NA | 0 | 1 | TRUE | FALSE |
| chr7 | 152079848 | 152079899 | 5 | 4 | L1M5 | TRUE | NA | 0 | 1 | TRUE | FALSE |
| chr7 | 152081329 | 152081401 | 16 | 9 | L2c | TRUE | CTCF | 1 | 1 | TRUE | FALSE |
| chr7 | 152106908 | 152106931 | 4 | 4 | NA | FALSE | NA | 0 | 1 | TRUE | FALSE |
| chr7 | 152109161 | 152109183 | 4 | 4 | NA | FALSE | NA | 0 | 1 | TRUE | FALSE |
| chr7 | 152109622 | 152109671 | 4 | 4 | NA | FALSE | NA | 0 | 1 | TRUE | FALSE |
| chr7 | 152880934 | 152880957 | 4 | 4 | NA | FALSE | NA | 0 | 1 | FALSE | FALSE |
| chr7 | 153012122 | 153012140 | 4 | 4 | SVA D | TRUE | NA | 0 | NA | FALSE | FALSE |
| chr7 | 153114143 | 153114143 | 4 | 4 | ERVL-E-int | TRUE | NA | 0 | 1 | FALSE | FALSE |
| chr7 | 153148017 | 153148033 | 4 | 3 | NA | FALSE | NA | 0 | 1 | FALSE | FALSE |
| chr7 | 153684497 | 153684505 | 4 | 3 | NA | FALSE | NA | 0 | 1 | TRUE | FALSE |
| chr7 | 153757758 | 153757784 | 6 | 5 | SATR1 | TRUE | NA | 0 | NA | FALSE | FALSE |
| chr7 | 153961816 | 153961843 | 4 | 3 | HAL1-2a MD | TRUE | NA | 0 | 1 | TRUE | FALSE |
| chr7 | 154452112 | 154452126 | 6 | 6 | NA | FALSE | NA | 0 | NA | TRUE | FALSE |
| chr7 | 154455759 | 154455849 | 8 | 6 | NA | FALSE | NA | 0 | NA | FALSE | FALSE |
| chr7 | 154827722 | 154827737 | 4 | 3 | (TTC)n | TRUE | NA | 0 | NA | TRUE | FALSE |
| chr7 | 154847832 | 154847860 | 4 | 4 | ERVL-E-int | TRUE | NA | 0 | 1 | TRUE | FALSE |
| chr7 | 154944736 | 154944736 | 4 | 4 | NA | FALSE | ZNF263 | 1 | 1 | TRUE | FALSE |
| chr7 | 155409727 | 155409761 | 6 | 5 | NA | FALSE | NA | 0 | 1 | FALSE | FALSE |
| chr7 | 155410230 | 155410235 | 5 | 5 | NA | FALSE | NA | 0 | NA | FALSE | FALSE |
| chr7 | 155466220 | 155466226 | 4 | 2 | (TA)n | TRUE | NA | 0 | NA | FALSE | FALSE |
| chr7 | 155662119 | 155662141 | 5 | 4 | NA | FALSE | NA | 0 | 1 | FALSE | FALSE |
| chr7 | 155817948 | 155817979 | 8 | 7 | (TG)n | TRUE | NA | 0 | 1 | TRUE | FALSE |
| chr7 | 155888929 | 155888954 | 4 | 4 | NA | FALSE | NA | 0 | 1 | TRUE | FALSE |
| chr7 | 155896420 | 155896464 | 6 | 4 | NA | FALSE | NA | 0 | 1 | FALSE | FALSE |
| chr7 | 156058692 | 156058724 | 4 | 4 | NA | FALSE | NA | 0 | NA | FALSE | FALSE |
| chr7 | 156059269 | 156059294 | 4 | 3 | NA | FALSE | NA | 0 | NA | FALSE | FALSE |
| chr7 | 156700990 | 156701027 | 4 | 2 | AluSg | TRUE | NA | 0 | NA | TRUE | FALSE |
| chr7 | 156923797 | 156923839 | 4 | 4 | (TC)n | TRUE | NA | 0 | NA | FALSE | FALSE |
| chr7 | 157275140 | 157275141 | 4 | 2 | (TG)n | TRUE | NA | 0 | 1 | FALSE | FALSE |
| chr7 | 157280645 | 157280672 | 5 | 5 | G-rich | TRUE | NA | 0 | 1 | TRUE | FALSE |
| chr7 | 157373399 | 157373418 | 4 | 4 | NA | FALSE | NA | 0 | 1 | FALSE | FALSE |
| chr7 | 157373489 | 157373537 | 9 | 5 | (CAGCC)n | TRUE | NA | 0 | NA | FALSE | FALSE |
| chr7 | 157518553 | 157518564 | 5 | 3 | L1M2 | TRUE | NA | 0 | 1 | FALSE | FALSE |
| chr7 | 157671685 | 157671706 | 4 | 4 | NA | FALSE | NA | 0 | NA | FALSE | FALSE |
| chr7 | 157709703 | 157709716 | 5 | 4 | NA | FALSE | NA | 0 | 1 | FALSE | FALSE |
| chr7 | 157726454 | 157726465 | 6 | 5 | NA | FALSE | MANY | 4 | NA | FALSE | FALSE |
| chr7 | 157891760 | 157891771 | 4 | 4 | C-rich | TRUE | NA | 0 | NA | FALSE | FALSE |
| chr7 | 157945510 | 157945516 | 5 | 4 | LTR1 | TRUE | NA | 0 | 1 | FALSE | FALSE |
| chr7 | 157957850 | 157957853 | 4 | 4 | NA | FALSE | NA | 0 | NA | TRUE | FALSE |
| chr7 | 158051274 | 158051295 | 4 | 2 | NA | FALSE | NA | 0 | 1 | TRUE | FALSE |
| chr7 | 158112583 | 158112605 | 4 | 3 | (CA)n | TRUE | NA | 0 | NA | FALSE | FALSE |
| chr7 | 158127033 | 158127043 | 5 | 3 | NA | FALSE | NA | 0 | NA | FALSE | FALSE |
| chr7 | 158135170 | 158135195 | 5 | 3 | NA | FALSE | NA | 0 | NA | TRUE | FALSE |
| chr7 | 158142512 | 158142533 | 4 | 4 | NA | FALSE | NA | 0 | NA | FALSE | FALSE |
| chr7 | 158192395 | 158192410 | 4 | 3 | NA | FALSE | NA | 0 | 1 | FALSE | FALSE |
| chr7 | 158210379 | 158210401 | 4 | 4 | NA | FALSE | NA | 0 | NA | FALSE | FALSE |
| chr7 | 158220651 | 158220653 | 5 | 3 | NA | FALSE | NA | 0 | NA | FALSE | FALSE |
| chr7 | 158316743 | 158316756 | 4 | 4 | (GAGTG)n | TRUE | MANY | 2 | NA | TRUE | FALSE |
| chr7 | 158326944 | 158326953 | 5 | 4 | NA | FALSE | NA | 0 | NA | TRUE | FALSE |
| chr7 | 158327003 | 158327020 | 5 | 5 | NA | FALSE | NA | 0 | NA | TRUE | FALSE |
| chr7 | 158752706 | 158752714 | 4 | 3 | NA | FALSE | NA | 0 | NA | TRUE | FALSE |
| chr7 | 158790973 | 158791001 | 4 | 4 | C-rich | TRUE | NA | 0 | NA | FALSE | FALSE |
| chr7 | 158893848 | 158893948 | 6 | 3 | MANY | TRUE | NA | 0 | NA | FALSE | FALSE |
| chr7 | 158968059 | 158968153 | 5 | 4 | NA | FALSE | PU.1 | 1 | 1 | TRUE | FALSE |
| chr7 | 158986619 | 158986678 | 4 | 3 | MER4C | TRUE | NA | 0 | 1 | TRUE | FALSE |
| chr7 | 159054597 | 159054646 | 4 | 3 | L2a | TRUE | NA | 0 | 1 | FALSE | FALSE |
| chr8 | 79903 | 79926 | 4 | 3 | L1M4 | TRUE | NA | 0 | NA | FALSE | FALSE |
| chr8 | 163186 | 163214 | 9 | 4 | NA | FALSE | CEBPB | 1 | 1 | FALSE | FALSE |
| chr8 | 266562 | 266606 | 5 | 3 | NA | FALSE | NA | 0 | 1 | TRUE | FALSE |
| chr8 | 701896 | 701896 | 4 | 4 | NA | FALSE | NA | 0 | NA | TRUE | FALSE |
| chr8 | 745971 | 746006 | 5 | 4 | NA | FALSE | NA | 0 | 1 | FALSE | FALSE |
| chr8 | 928653 | 928688 | 4 | 3 | NA | FALSE | NA | 0 | 1 | TRUE | FALSE |
| chr8 | 1039049 | 1039079 | 4 | 2 | NA | FALSE | NA | 0 | 1 | FALSE | FALSE |
| chr8 | 1298893 | 1298934 | 5 | 4 | NA | FALSE | NA | 0 | 1 | FALSE | FALSE |
| chr8 | 1333899 | 1333924 | 5 | 5 | NA | FALSE | NA | 0 | NA | FALSE | FALSE |
| chr8 | 1439533 | 1439553 | 7 | 7 | NA | FALSE | NA | 0 | 1 | TRUE | FALSE |
| chr8 | 2003180 | 2003194 | 4 | 3 | NA | FALSE | NA | 0 | NA | FALSE | FALSE |
| chr8 | 2021698 | 2021720 | 4 | 3 | NA | FALSE | NA | 0 | 1 | TRUE | FALSE |
| chr8 | 2201469 | 2201492 | 4 | 4 | NA | FALSE | NA | 0 | 1 | FALSE | FALSE |
| chr8 | 2224301 | 2224318 | 4 | 4 | NA | FALSE | NA | 0 | 1 | TRUE | FALSE |
| chr8 | 2262703 | 2262722 | 4 | 4 | NA | FALSE | NA | 0 | 1 | TRUE | FALSE |
| chr8 | 2512619 | 2512632 | 4 | 3 | LTR82A | TRUE | MANY | 4 | 1 | FALSE | FALSE |
| chr8 | 3120451 | 3120459 | 4 | 3 | NA | FALSE | NA | 0 | 1 | FALSE | FALSE |
| chr8 | 4128299 | 4128313 | 4 | 3 | NA | FALSE | NA | 0 | 1 | FALSE | FALSE |
| chr8 | 4526778 | 4526796 | 4 | 4 | NA | FALSE | NA | 0 | 1 | FALSE | FALSE |
| chr8 | 5015433 | 5015437 | 4 | 3 | NA | FALSE | NA | 0 | 1 | TRUE | FALSE |
| chr8 | 5117289 | 5117292 | 4 | 3 | L1PA13 | TRUE | NA | 0 | NA | FALSE | FALSE |
| chr8 | 5335457 | 5335458 | 4 | 4 | AluSx | TRUE | NA | 0 | NA | FALSE | FALSE |
| chr8 | 5372293 | 5372301 | 4 | 3 | ERVL-E-int | TRUE | NA | 0 | 1 | FALSE | FALSE |
| chr8 | 5401042 | 5401042 | 4 | 4 | MLT1L | TRUE | NA | 0 | 1 | FALSE | FALSE |
| chr8 | 5505719 | 5505733 | 4 | 3 | NA | FALSE | NA | 0 | 1 | FALSE | FALSE |
| chr8 | 5539367 | 5539373 | 4 | 4 | NA | FALSE | NA | 0 | 1 | FALSE | FALSE |
| chr8 | 5815324 | 5815338 | 4 | 3 | NA | FALSE | NA | 0 | 1 | FALSE | FALSE |
| chr8 | 6000466 | 6000466 | 4 | 4 | (TC)n | TRUE | NA | 0 | NA | FALSE | FALSE |
| chr8 | 6730680 | 6730708 | 4 | 3 | MIR3 | TRUE | MANY | 2 | 1 | TRUE | FALSE |
| chr8 | 6782398 | 6782415 | 4 | 2 | NA | FALSE | NA | 0 | 1 | TRUE | FALSE |
| chr8 | 6947612 | 6947640 | 5 | 3 | ERVL-B4-int | TRUE | NA | 0 | 1 | FALSE | FALSE |
| chr8 | 6957801 | 6957854 | 14 | 10 | NA | FALSE | NA | 0 | 1 | FALSE | FALSE |
| chr8 | 7463846 | 7463888 | 6 | 5 | L2c | TRUE | NA | 0 | NA | FALSE | FALSE |
| chr8 | 7885729 | 7885814 | 4 | 4 | NA | FALSE | NA | 0 | 1 | TRUE | FALSE |
| chr8 | 7996861 | 7996876 | 6 | 3 | HERVS71-int | TRUE | NA | 0 | NA | FALSE | FALSE |
| chr8 | 9140796 | 9140909 | 8 | 7 | (TTTCC)n | TRUE | NA | 0 | NA | FALSE | FALSE |
| chr8 | 9303003 | 9303004 | 4 | 2 | L1PA5 | TRUE | NA | 0 | NA | FALSE | FALSE |
| chr8 | 9838071 | 9838103 | 4 | 4 | C-rich | TRUE | NA | 0 | 1 | FALSE | FALSE |
| chr8 | 10663206 | 10663239 | 5 | 3 | NA | FALSE | NA | 0 | NA | TRUE | FALSE |
| chr8 | 10860412 | 10860448 | 4 | 4 | NA | FALSE | NA | 0 | 1 | FALSE | FALSE |
| chr8 | 11468122 | 11468154 | 4 | 4 | L1MA4A | TRUE | NA | 0 | 1 | FALSE | FALSE |
| chr8 | 11866530 | 11866578 | 9 | 4 | NA | FALSE | NA | 0 | 1 | FALSE | FALSE |
| chr8 | 11873823 | 11873861 | 4 | 2 | G-rich | TRUE | NA | 0 | NA | TRUE | FALSE |
| chr8 | 11996712 | 11996718 | 4 | 4 | (TG)n | TRUE | NA | 0 | NA | FALSE | FALSE |
| chr8 | 11997239 | 11997245 | 4 | 4 | NA | FALSE | NA | 0 | 1 | FALSE | FALSE |
| chr8 | 12306634 | 12306686 | 4 | 3 | (TA)n | TRUE | NA | 0 | 1 | FALSE | FALSE |
| chr8 | 12401001 | 12401008 | 6 | 5 | NA | FALSE | NA | 0 | 1 | TRUE | FALSE |
| chr8 | 12416764 | 12416765 | 4 | 2 | L1M2 | TRUE | NA | 0 | 1 | TRUE | FALSE |
| chr8 | 12425775 | 12425828 | 4 | 2 | NA | FALSE | NA | 0 | 1 | FALSE | FALSE |
| chr8 | 12477154 | 12477185 | 6 | 2 | MANY | TRUE | NA | 0 | 1 | FALSE | FALSE |
| chr8 | 12482113 | 12482175 | 4 | 4 | LTR5A | TRUE | NA | 0 | 1 | FALSE | FALSE |
| chr8 | 12500512 | 12500546 | 4 | 4 | AluSz6 | TRUE | NA | 0 | NA | FALSE | FALSE |
| chr8 | 12502426 | 12502438 | 6 | 3 | NA | FALSE | NA | 0 | 1 | FALSE | FALSE |
| chr8 | 12511127 | 12511133 | 6 | 4 | AluSz | TRUE | NA | 0 | NA | FALSE | FALSE |
| chr8 | 12514653 | 12514682 | 6 | 3 | AluJb | TRUE | NA | 0 | NA | FALSE | FALSE |
| chr8 | 12522820 | 12522884 | 5 | 3 | NA | FALSE | NA | 0 | NA | FALSE | FALSE |
| chr8 | 12525507 | 12525583 | 4 | 3 | NA | FALSE | NA | 0 | 1 | TRUE | FALSE |
| chr8 | 13619230 | 13619231 | 4 | 4 | L1MA9 | TRUE | NA | 0 | 1 | FALSE | FALSE |
| chr8 | 13636078 | 13636097 | 4 | 4 | L1M5 | TRUE | NA | 0 | 1 | FALSE | FALSE |
| chr8 | 13646981 | 13647005 | 4 | 4 | NA | FALSE | NA | 0 | 1 | FALSE | FALSE |
| chr8 | 13955956 | 13955957 | 4 | 2 | LTR79 | TRUE | NA | 0 | 1 | FALSE | FALSE |
| chr8 | 14123347 | 14123347 | 4 | 4 | L1M1 | TRUE | NA | 0 | 1 | FALSE | FALSE |
| chr8 | 14191870 | 14191875 | 4 | 4 | MIRc | TRUE | NA | 0 | 1 | FALSE | FALSE |
| chr8 | 14302448 | 14302448 | 4 | 4 | MER101-int | TRUE | NA | 0 | NA | FALSE | FALSE |
| chr8 | 14370246 | 14370250 | 4 | 3 | AluJo | TRUE | NA | 0 | 1 | FALSE | FALSE |
| chr8 | 14596654 | 14596661 | 4 | 2 | NA | FALSE | NA | 0 | 1 | FALSE | FALSE |
| chr8 | 14901077 | 14901077 | 4 | 4 | NA | FALSE | NA | 0 | 1 | FALSE | FALSE |
| chr8 | 15048814 | 15048814 | 4 | 4 | NA | FALSE | NA | 0 | 1 | FALSE | FALSE |
| chr8 | 15293789 | 15293804 | 4 | 4 | MLT1F | TRUE | NA | 0 | 1 | FALSE | FALSE |
| chr8 | 15331431 | 15331441 | 4 | 3 | MER11A | TRUE | NA | 0 | 1 | TRUE | FALSE |
| chr8 | 15492575 | 15492585 | 4 | 3 | HERVL40-int | TRUE | NA | 0 | 1 | FALSE | FALSE |
| chr8 | 15670628 | 15670629 | 4 | 4 | AluSx4 | TRUE | NA | 0 | 1 | FALSE | FALSE |
| chr8 | 15680126 | 15680144 | 5 | 4 | L2 | TRUE | NA | 0 | 1 | FALSE | FALSE |
| chr8 | 15818031 | 15818031 | 4 | 4 | L1PA15 | TRUE | NA | 0 | 1 | FALSE | FALSE |
| chr8 | 16237594 | 16237605 | 4 | 4 | NA | FALSE | NA | 0 | 1 | FALSE | FALSE |
| chr8 | 16760594 | 16760595 | 4 | 4 | AluYg6 | TRUE | NA | 0 | NA | FALSE | FALSE |
| chr8 | 16777959 | 16778000 | 4 | 4 | AluY | TRUE | NA | 0 | NA | FALSE | FALSE |
| chr8 | 18365052 | 18365101 | 4 | 2 | MLT1D | TRUE | NA | 0 | 1 | TRUE | FALSE |
| chr8 | 18967156 | 18967229 | 4 | 3 | CT-rich | TRUE | NA | 0 | 1 | TRUE | FALSE |
| chr8 | 20007113 | 20007150 | 4 | 4 | (TC)n | TRUE | NA | 0 | 1 | FALSE | FALSE |
| chr8 | 21153083 | 21153102 | 4 | 3 | CT-rich | TRUE | NA | 0 | 1 | TRUE | FALSE |
| chr8 | 21195415 | 21195426 | 4 | 4 | AluSx | TRUE | NA | 0 | 1 | FALSE | FALSE |
| chr8 | 21468857 | 21468857 | 5 | 5 | (TG)n | TRUE | EBF | 1 | NA | TRUE | FALSE |
| chr8 | 24350347 | 24350357 | 4 | 4 | NA | FALSE | NA | 0 | 1 | FALSE | FALSE |
| chr8 | 24529760 | 24529775 | 4 | 3 | MER41A | TRUE | NA | 0 | 1 | TRUE | FALSE |
| chr8 | 25516980 | 25517005 | 6 | 3 | L1PA3 | TRUE | NA | 0 | NA | FALSE | FALSE |
| chr8 | 25564673 | 25564681 | 4 | 4 | NA | FALSE | NA | 0 | 1 | FALSE | FALSE |
| chr8 | 26540822 | 26540857 | 5 | 5 | SVA A | TRUE | MANY | 2 | NA | TRUE | FALSE |
| chr8 | 26759853 | 26759918 | 5 | 4 | L2c | TRUE | NA | 0 | 1 | TRUE | FALSE |
| chr8 | 26841145 | 26841186 | 5 | 4 | MER4C | TRUE | NA | 0 | 1 | FALSE | FALSE |
| chr8 | 26862512 | 26862552 | 4 | 4 | ERVL-E-int | TRUE | NA | 0 | 1 | FALSE | FALSE |
| chr8 | 27168914 | 27168969 | 6 | 5 | NA | FALSE | MANY | 26 | 1 | TRUE | TRUE |
| chr8 | 28151685 | 28151730 | 4 | 3 | MER83 | TRUE | SETDB1 | 1 | 1 | FALSE | FALSE |
| chr8 | 28480088 | 28480135 | 6 | 3 | G-rich | TRUE | MANY | 4 | 1 | TRUE | TRUE |
| chr8 | 29307361 | 29307425 | 4 | 2 | L1PA5 | TRUE | NA | 0 | NA | FALSE | FALSE |
| chr8 | 30518664 | 30518684 | 4 | 4 | (CA)n | TRUE | NA | 0 | NA | TRUE | FALSE |
| chr8 | 30601667 | 30601698 | 9 | 6 | NA | FALSE | MANY | 29 | 1 | TRUE | TRUE |
| chr8 | 31603876 | 31603878 | 4 | 3 | NA | FALSE | NA | 0 | 1 | FALSE | FALSE |
| chr8 | 31633918 | 31633938 | 6 | 4 | NA | FALSE | NA | 0 | 1 | FALSE | FALSE |
| chr8 | 32769978 | 32770000 | 4 | 3 | NA | FALSE | NA | 0 | 1 | FALSE | FALSE |
| chr8 | 33535119 | 33535148 | 6 | 5 | NA | FALSE | NA | 0 | 1 | FALSE | FALSE |
| chr8 | 34393126 | 34393140 | 4 | 4 | L1P2 | TRUE | NA | 0 | 1 | FALSE | FALSE |
| chr8 | 34632747 | 34632771 | 6 | 4 | L2a | TRUE | NA | 0 | 1 | FALSE | FALSE |
| chr8 | 34764918 | 34764934 | 4 | 3 | NA | FALSE | TCF12 | 1 | 1 | TRUE | FALSE |
| chr8 | 34802103 | 34802123 | 4 | 4 | NA | FALSE | NA | 0 | 1 | TRUE | FALSE |
| chr8 | 35189259 | 35189278 | 4 | 2 | MIRb | TRUE | NA | 0 | 1 | FALSE | FALSE |
| chr8 | 35260392 | 35260412 | 4 | 4 | NA | FALSE | NA | 0 | 1 | FALSE | FALSE |
| chr8 | 36036677 | 36036678 | 4 | 4 | L1MEc | TRUE | NA | 0 | 1 | FALSE | FALSE |
| chr8 | 36062294 | 36062295 | 5 | 4 | NA | FALSE | NA | 0 | 1 | FALSE | FALSE |
| chr8 | 36201740 | 36201742 | 4 | 4 | (GA)n | TRUE | NA | 0 | NA | FALSE | FALSE |
| chr8 | 36728923 | 36728927 | 5 | 2 | NA | FALSE | NA | 0 | NA | FALSE | FALSE |
| chr8 | 36882847 | 36882882 | 4 | 4 | (TC)n | TRUE | NA | 0 | 1 | FALSE | FALSE |
| chr8 | 36925857 | 36925858 | 4 | 3 | NA | FALSE | NA | 0 | 1 | TRUE | FALSE |
| chr8 | 37594057 | 37594117 | 6 | 4 | NA | FALSE | MANY | 56 | 1 | TRUE | TRUE |
| chr8 | 37644240 | 37644276 | 6 | 6 | MANY | TRUE | NA | 0 | NA | TRUE | FALSE |
| chr8 | 37777293 | 37777356 | 4 | 3 | NA | FALSE | NA | 0 | 1 | FALSE | FALSE |
| chr8 | 37784683 | 37784716 | 13 | 10 | SVA D | TRUE | NA | 0 | NA | FALSE | FALSE |
| chr8 | 38012832 | 38012894 | 6 | 3 | AluSp | TRUE | NA | 0 | NA | FALSE | FALSE |
| chr8 | 39545775 | 39545778 | 4 | 3 | L1PA13 | TRUE | NA | 0 | NA | FALSE | FALSE |
| chr8 | 40233153 | 40233155 | 4 | 3 | (CA)n | TRUE | NA | 0 | NA | FALSE | FALSE |
| chr8 | 40318388 | 40318404 | 4 | 3 | MIR3 | TRUE | NA | 0 | 1 | FALSE | FALSE |
| chr8 | 40898891 | 40898894 | 4 | 2 | L1PB1 | TRUE | NA | 0 | NA | FALSE | FALSE |
| chr8 | 41026497 | 41026501 | 4 | 2 | AluSq | TRUE | NA | 0 | NA | FALSE | FALSE |
| chr8 | 41063719 | 41063726 | 4 | 4 | MLT1B | TRUE | NA | 0 | 1 | TRUE | FALSE |
| chr8 | 41759318 | 41759351 | 4 | 3 | NA | FALSE | NA | 0 | 1 | FALSE | FALSE |
| chr8 | 42110470 | 42110536 | 7 | 4 | MER4E1 | TRUE | c-Fos | 1 | NA | FALSE | FALSE |
| chr8 | 42421032 | 42421106 | 4 | 3 | HERV9-int | TRUE | NA | 0 | 1 | FALSE | FALSE |
| chr8 | 43185949 | 43185979 | 5 | 5 | L1PA5 | TRUE | NA | 0 | NA | FALSE | FALSE |
| chr8 | 43355842 | 43355852 | 4 | 3 | MLT1G1 | TRUE | NA | 0 | 1 | TRUE | FALSE |
| chr8 | 47545441 | 47545451 | 4 | 4 | L1PA3 | TRUE | NA | 0 | NA | FALSE | FALSE |
| chr8 | 47638494 | 47638505 | 4 | 4 | L1MC4a | TRUE | NA | 0 | 1 | FALSE | FALSE |
| chr8 | 49907397 | 49907404 | 6 | 4 | L1PA4 | TRUE | NA | 0 | NA | FALSE | FALSE |
| chr8 | 50341164 | 50341182 | 4 | 3 | HERV9-int | TRUE | NA | 0 | 1 | FALSE | FALSE |
| chr8 | 50425603 | 50425620 | 4 | 3 | NA | FALSE | NA | 0 | 1 | FALSE | FALSE |
| chr8 | 50457644 | 50457662 | 4 | 3 | MER21A | TRUE | NA | 0 | 1 | FALSE | FALSE |
| chr8 | 50479687 | 50479699 | 4 | 4 | NA | FALSE | NA | 0 | 1 | FALSE | FALSE |
| chr8 | 50509669 | 50509687 | 4 | 2 | L1MC2 | TRUE | NA | 0 | 1 | FALSE | FALSE |
| chr8 | 50987415 | 50987428 | 4 | 2 | LTR12C | TRUE | NA | 0 | 1 | TRUE | FALSE |
| chr8 | 51438650 | 51438674 | 4 | 3 | NA | FALSE | NA | 0 | 1 | TRUE | FALSE |
| chr8 | 51933206 | 51933224 | 4 | 3 | NA | FALSE | NA | 0 | 1 | FALSE | FALSE |
| chr8 | 52555291 | 52555313 | 4 | 4 | L1P1 | TRUE | NA | 0 | 1 | FALSE | FALSE |
| chr8 | 52732957 | 52732964 | 6 | 5 | NA | FALSE | MafK (ab50322) | 1 | 1 | TRUE | FALSE |
| chr8 | 52733208 | 52733226 | 6 | 5 | NA | FALSE | NA | 0 | 1 | TRUE | FALSE |
| chr8 | 53432587 | 53432588 | 4 | 3 | NA | FALSE | NA | 0 | 1 | TRUE | FALSE |
| chr8 | 53986767 | 53986778 | 4 | 3 | HERV9-int | TRUE | NA | 0 | NA | TRUE | FALSE |
| chr8 | 53987545 | 53987578 | 6 | 5 | HERV9-int | TRUE | NA | 0 | 1 | FALSE | FALSE |
| chr8 | 54077353 | 54077373 | 4 | 3 | MSTB2 | TRUE | NA | 0 | 1 | FALSE | FALSE |
| chr8 | 54157637 | 54157638 | 4 | 3 | MER2 | TRUE | NA | 0 | 1 | FALSE | FALSE |
| chr8 | 54353138 | 54353150 | 5 | 3 | HERV9-int | TRUE | NA | 0 | NA | FALSE | FALSE |
| chr8 | 54374017 | 54374037 | 5 | 4 | HERV9-int | TRUE | NA | 0 | NA | FALSE | FALSE |
| chr8 | 54376508 | 54376531 | 4 | 2 | HERV9-int | TRUE | NA | 0 | 1 | FALSE | FALSE |
| chr8 | 54504071 | 54504091 | 4 | 3 | NA | FALSE | NA | 0 | 1 | FALSE | FALSE |
| chr8 | 54518767 | 54518786 | 4 | 4 | L1PA16 | TRUE | NA | 0 | 1 | FALSE | FALSE |
| chr8 | 55282368 | 55282381 | 4 | 3 | C-rich | TRUE | NA | 0 | NA | FALSE | FALSE |
| chr8 | 55425984 | 55425998 | 4 | 4 | MLT1D | TRUE | NA | 0 | 1 | FALSE | FALSE |
| chr8 | 55657375 | 55657389 | 4 | 3 | NA | FALSE | NA | 0 | 1 | FALSE | FALSE |
| chr8 | 56529525 | 56529545 | 4 | 3 | L1PA4 | TRUE | NA | 0 | NA | FALSE | FALSE |
| chr8 | 56987133 | 56987140 | 6 | 5 | NA | FALSE | MANY | 67 | 1 | TRUE | TRUE |
| chr8 | 57231155 | 57231178 | 4 | 2 | NA | FALSE | NA | 0 | 1 | FALSE | FALSE |
| chr8 | 57251223 | 57251246 | 4 | 4 | L1P2 | TRUE | NA | 0 | 1 | FALSE | FALSE |
| chr8 | 57441955 | 57441975 | 4 | 2 | LTR1 | TRUE | NA | 0 | 1 | TRUE | FALSE |
| chr8 | 58122230 | 58122263 | 5 | 4 | MER50-int | TRUE | MANY | 8 | 1 | TRUE | TRUE |
| chr8 | 58122553 | 58122571 | 4 | 4 | MER50-int | TRUE | MANY | 2 | NA | TRUE | FALSE |
| chr8 | 58122957 | 58122981 | 4 | 3 | MER50 | TRUE | Egr-1 | 1 | 1 | TRUE | FALSE |
| chr8 | 59207302 | 59207348 | 4 | 2 | L1M5 | TRUE | MANY | 3 | 1 | FALSE | FALSE |
| chr8 | 61240729 | 61240767 | 5 | 4 | L1MA9 | TRUE | NA | 0 | 1 | FALSE | FALSE |
| chr8 | 62076103 | 62076116 | 4 | 2 | LTR17 | TRUE | NA | 0 | NA | FALSE | FALSE |
| chr8 | 62227395 | 62227432 | 5 | 4 | Tigger15a | TRUE | NA | 0 | 1 | FALSE | FALSE |
| chr8 | 62233982 | 62234015 | 4 | 4 | L1M6 | TRUE | NA | 0 | 1 | FALSE | FALSE |
| chr8 | 62312590 | 62312611 | 4 | 3 | L1PA11 | TRUE | NA | 0 | NA | FALSE | FALSE |
| chr8 | 62817764 | 62817780 | 4 | 3 | MER5A | TRUE | NA | 0 | 1 | FALSE | FALSE |
| chr8 | 63154124 | 63154153 | 5 | 4 | L1PA7 | TRUE | NA | 0 | NA | FALSE | FALSE |
| chr8 | 63773622 | 63773629 | 4 | 2 | MER117 | TRUE | FOXA1 (C-20) | 1 | 1 | TRUE | FALSE |
| chr8 | 64483439 | 64483445 | 4 | 3 | MLT1D | TRUE | NA | 0 | 1 | FALSE | FALSE |
| chr8 | 64509246 | 64509255 | 4 | 3 | NA | FALSE | NA | 0 | 1 | FALSE | FALSE |
| chr8 | 64566264 | 64566280 | 4 | 4 | NA | FALSE | NA | 0 | 1 | FALSE | FALSE |
| chr8 | 64668887 | 64668893 | 4 | 2 | L1PA11 | TRUE | NA | 0 | 1 | FALSE | FALSE |
| chr8 | 65094862 | 65094863 | 4 | 3 | L1PA13 | TRUE | NA | 0 | 1 | FALSE | FALSE |
| chr8 | 65172641 | 65172651 | 4 | 3 | NA | FALSE | NA | 0 | 1 | FALSE | FALSE |
| chr8 | 65258650 | 65258689 | 5 | 4 | NA | FALSE | NA | 0 | 1 | FALSE | FALSE |
| chr8 | 65408859 | 65408875 | 4 | 4 | L1MA3 | TRUE | NA | 0 | 1 | FALSE | FALSE |
| chr8 | 65434619 | 65434631 | 4 | 3 | NA | FALSE | NA | 0 | 1 | FALSE | FALSE |
| chr8 | 65453390 | 65453405 | 4 | 4 | A-rich | TRUE | NA | 0 | NA | FALSE | FALSE |
| chr8 | 65804518 | 65804538 | 4 | 2 | (TTCC)n | TRUE | NA | 0 | NA | TRUE | FALSE |
| chr8 | 65972145 | 65972152 | 4 | 3 | LTR1D | TRUE | NA | 0 | 1 | FALSE | FALSE |
| chr8 | 66247787 | 66247811 | 4 | 4 | NA | FALSE | NA | 0 | 1 | FALSE | FALSE |
| chr8 | 66332591 | 66332607 | 4 | 1 | (GGAA)n | TRUE | NA | 0 | NA | FALSE | FALSE |
| chr8 | 66418797 | 66418814 | 7 | 4 | L1PB1 | TRUE | NA | 0 | NA | FALSE | FALSE |
| chr8 | 66662748 | 66662748 | 4 | 4 | (TG)n | TRUE | NA | 0 | NA | FALSE | FALSE |
| chr8 | 66794665 | 66794704 | 5 | 5 | MER1B | TRUE | NA | 0 | 1 | FALSE | FALSE |
| chr8 | 67180108 | 67180118 | 4 | 3 | NA | FALSE | NA | 0 | 1 | FALSE | FALSE |
| chr8 | 67434086 | 67434090 | 5 | 4 | (GA)n | TRUE | MANY | 4 | NA | FALSE | FALSE |
| chr8 | 67458171 | 67458191 | 4 | 4 | (GGAA)n | TRUE | NA | 0 | NA | TRUE | FALSE |
| chr8 | 67579516 | 67579582 | 7 | 4 | NA | FALSE | MANY | 33 | 1 | TRUE | TRUE |
| chr8 | 67837793 | 67837825 | 7 | 7 | NA | FALSE | MANY | 39 | 1 | TRUE | TRUE |
| chr8 | 68079720 | 68079769 | 5 | 4 | SVA C | TRUE | NA | 0 | NA | FALSE | FALSE |
| chr8 | 68579208 | 68579224 | 4 | 3 | NA | FALSE | NA | 0 | 1 | FALSE | FALSE |
| chr8 | 68989693 | 68989709 | 4 | 3 | NA | FALSE | MANY | 2 | 1 | TRUE | FALSE |
| chr8 | 69509818 | 69509822 | 4 | 4 | (TG)n | TRUE | NA | 0 | NA | FALSE | FALSE |
| chr8 | 69573079 | 69573087 | 4 | 4 | MER61-int | TRUE | NA | 0 | 1 | FALSE | FALSE |
| chr8 | 69618015 | 69618028 | 4 | 3 | L2c | TRUE | NA | 0 | 1 | FALSE | FALSE |
| chr8 | 69757664 | 69757673 | 4 | 3 | HERV35I-int | TRUE | NA | 0 | 1 | FALSE | FALSE |
| chr8 | 69887178 | 69887197 | 5 | 4 | HERVH-int | TRUE | NA | 0 | NA | FALSE | FALSE |
| chr8 | 70628993 | 70629040 | 4 | 2 | MANY | TRUE | NA | 0 | 1 | FALSE | FALSE |
| chr8 | 71409905 | 71409947 | 5 | 5 | MANY | TRUE | NA | 0 | 1 | FALSE | FALSE |
| chr8 | 71458823 | 71458863 | 4 | 4 | NA | FALSE | NA | 0 | 1 | FALSE | FALSE |
| chr8 | 71652749 | 71652771 | 4 | 3 | L1PA3 | TRUE | NA | 0 | NA | FALSE | FALSE |
| chr8 | 71694731 | 71694731 | 4 | 4 | L1MA7 | TRUE | NA | 0 | 1 | FALSE | FALSE |
| chr8 | 73181114 | 73181131 | 4 | 3 | MER66-int | TRUE | NA | 0 | 1 | FALSE | FALSE |
| chr8 | 73310420 | 73310439 | 4 | 4 | MANY | TRUE | NA | 0 | 1 | FALSE | FALSE |
| chr8 | 73330709 | 73330729 | 6 | 6 | (TC)n | TRUE | ZZZ3 | 1 | NA | FALSE | FALSE |
| chr8 | 73388352 | 73388366 | 4 | 2 | L1PA17 | TRUE | NA | 0 | 1 | FALSE | FALSE |
| chr8 | 73389592 | 73389610 | 4 | 2 | L1PA17 | TRUE | NA | 0 | 1 | FALSE | FALSE |
| chr8 | 73827824 | 73827831 | 4 | 4 | NA | FALSE | NA | 0 | 1 | FALSE | FALSE |
| chr8 | 73867195 | 73867210 | 4 | 3 | NA | FALSE | NA | 0 | 1 | FALSE | FALSE |
| chr8 | 74092662 | 74092675 | 4 | 4 | MANY | TRUE | ZZZ3 | 1 | NA | FALSE | FALSE |
| chr8 | 74791171 | 74791208 | 4 | 3 | NA | FALSE | MANY | 29 | 1 | TRUE | TRUE |
| chr8 | 75054453 | 75054492 | 4 | 3 | NA | FALSE | NA | 0 | 1 | FALSE | FALSE |
| chr8 | 75193321 | 75193366 | 4 | 3 | NA | FALSE | MANY | 3 | 1 | TRUE | TRUE |
| chr8 | 75262589 | 75262590 | 7 | 6 | NA | FALSE | MANY | 19 | 1 | TRUE | TRUE |
| chr8 | 76129443 | 76129460 | 4 | 4 | LTR7 | TRUE | NA | 0 | 1 | TRUE | FALSE |
| chr8 | 76188550 | 76188576 | 4 | 4 | L1M1 | TRUE | NA | 0 | NA | FALSE | FALSE |
| chr8 | 76369605 | 76369625 | 4 | 4 | NA | FALSE | NA | 0 | 1 | TRUE | FALSE |
| chr8 | 76493487 | 76493514 | 4 | 3 | L1MC1 | TRUE | NA | 0 | 1 | FALSE | FALSE |
| chr8 | 77114703 | 77114704 | 4 | 3 | NA | FALSE | NA | 0 | 1 | FALSE | FALSE |
| chr8 | 77406704 | 77406722 | 4 | 3 | L1MA3 | TRUE | NA | 0 | NA | FALSE | FALSE |
| chr8 | 78196569 | 78196576 | 4 | 4 | NA | FALSE | NA | 0 | 1 | FALSE | FALSE |
| chr8 | 78659694 | 78659710 | 4 | 3 | L1M1 | TRUE | NA | 0 | 1 | FALSE | FALSE |
| chr8 | 79239045 | 79239051 | 4 | 3 | NA | FALSE | NA | 0 | 1 | FALSE | FALSE |
| chr8 | 79755941 | 79755948 | 5 | 2 | AluSq2 | TRUE | NA | 0 | 1 | FALSE | FALSE |
| chr8 | 79858667 | 79858686 | 4 | 3 | NA | FALSE | NA | 0 | 1 | FALSE | FALSE |
| chr8 | 79947546 | 79947550 | 4 | 4 | L1PA11 | TRUE | NA | 0 | NA | FALSE | FALSE |
| chr8 | 81206399 | 81206417 | 4 | 3 | AluSq | TRUE | NA | 0 | NA | FALSE | FALSE |
| chr8 | 82514802 | 82514844 | 4 | 3 | MER5B | TRUE | NA | 0 | 1 | TRUE | FALSE |
| chr8 | 83252512 | 83252527 | 4 | 3 | MIRc | TRUE | NA | 0 | 1 | TRUE | FALSE |
| chr8 | 83904675 | 83904688 | 4 | 4 | FLAM A | TRUE | NA | 0 | 1 | FALSE | FALSE |
| chr8 | 84085168 | 84085181 | 4 | 3 | NA | FALSE | NA | 0 | 1 | FALSE | FALSE |
| chr8 | 84124945 | 84124951 | 4 | 3 | NA | FALSE | NA | 0 | 1 | FALSE | FALSE |
| chr8 | 84143579 | 84143586 | 4 | 2 | L1PA4 | TRUE | NA | 0 | NA | FALSE | FALSE |
| chr8 | 84161112 | 84161125 | 4 | 4 | L1MC1 | TRUE | NA | 0 | 1 | FALSE | FALSE |
| chr8 | 84166620 | 84166629 | 4 | 3 | NA | FALSE | NA | 0 | 1 | FALSE | FALSE |
| chr8 | 84236813 | 84236814 | 5 | 4 | L1PA7 | TRUE | NA | 0 | NA | FALSE | FALSE |
| chr8 | 84722578 | 84722596 | 4 | 4 | LTR8 | TRUE | NA | 0 | NA | FALSE | FALSE |
| chr8 | 85135356 | 85135371 | 4 | 1 | (TTTCC)n | TRUE | NA | 0 | NA | FALSE | FALSE |
| chr8 | 85774283 | 85774305 | 4 | 3 | NA | FALSE | NA | 0 | 1 | FALSE | FALSE |
| chr8 | 85865183 | 85865207 | 4 | 4 | L1PA6 | TRUE | NA | 0 | NA | FALSE | FALSE |
| chr8 | 85881041 | 85881066 | 4 | 4 | L2 | TRUE | NA | 0 | 1 | FALSE | FALSE |
| chr8 | 86348826 | 86348859 | 4 | 3 | NA | FALSE | STAT3 | 1 | 1 | TRUE | FALSE |
| chr8 | 87183957 | 87184000 | 4 | 3 | L1PA6 | TRUE | NA | 0 | NA | FALSE | FALSE |
| chr8 | 87203632 | 87203674 | 4 | 3 | THE1C | TRUE | NA | 0 | 1 | FALSE | FALSE |
| chr8 | 87219185 | 87219224 | 4 | 4 | L2 | TRUE | NA | 0 | 1 | FALSE | FALSE |
| chr8 | 87251362 | 87251383 | 4 | 4 | L1MC2 | TRUE | NA | 0 | 1 | FALSE | FALSE |
| chr8 | 87969006 | 87969028 | 4 | 3 | L1PA5 | TRUE | NA | 0 | NA | FALSE | FALSE |
| chr8 | 88226776 | 88226798 | 4 | 3 | THE1B-int | TRUE | NA | 0 | 1 | FALSE | FALSE |
| chr8 | 88483428 | 88483430 | 4 | 3 | NA | FALSE | NA | 0 | 1 | FALSE | FALSE |
| chr8 | 88581409 | 88581421 | 4 | 3 | THE1C-int | TRUE | NA | 0 | NA | FALSE | FALSE |
| chr8 | 88790706 | 88790728 | 4 | 4 | L1M2 | TRUE | NA | 0 | 1 | FALSE | FALSE |
| chr8 | 89468623 | 89468645 | 4 | 3 | L1PA12 | TRUE | NA | 0 | 1 | FALSE | FALSE |
| chr8 | 89469873 | 89469884 | 4 | 3 | L1PA12 | TRUE | NA | 0 | 1 | FALSE | FALSE |
| chr8 | 89756963 | 89756974 | 4 | 3 | HERVH-int | TRUE | NA | 0 | NA | FALSE | FALSE |
| chr8 | 89757092 | 89757092 | 4 | 4 | HERVH-int | TRUE | NA | 0 | NA | FALSE | FALSE |
| chr8 | 89893678 | 89893678 | 4 | 4 | (GA)n | TRUE | NA | 0 | NA | FALSE | FALSE |
| chr8 | 91270415 | 91270415 | 4 | 4 | L1MEf | TRUE | NA | 0 | 1 | FALSE | FALSE |
| chr8 | 92269313 | 92269323 | 4 | 4 | NA | FALSE | NA | 0 | 1 | FALSE | FALSE |
| chr8 | 92618988 | 92619003 | 4 | 3 | LTR16A1 | TRUE | NA | 0 | 1 | FALSE | FALSE |
| chr8 | 93365593 | 93365606 | 5 | 4 | L1PA8 | TRUE | NA | 0 | 1 | FALSE | FALSE |
| chr8 | 93455315 | 93455323 | 4 | 3 | L2c | TRUE | NA | 0 | 1 | FALSE | FALSE |
| chr8 | 94478898 | 94478909 | 4 | 3 | L1P2 | TRUE | NA | 0 | NA | FALSE | FALSE |
| chr8 | 94513019 | 94513043 | 4 | 2 | NA | FALSE | NA | 0 | 1 | FALSE | FALSE |
| chr8 | 94575431 | 94575446 | 4 | 3 | MLT2B1 | TRUE | NA | 0 | 1 | FALSE | FALSE |
| chr8 | 95203909 | 95203963 | 4 | 4 | AluSq | TRUE | NA | 0 | NA | FALSE | FALSE |
| chr8 | 95350115 | 95350152 | 4 | 3 | L1PA3 | TRUE | NA | 0 | NA | FALSE | FALSE |
| chr8 | 95816592 | 95816679 | 4 | 3 | AluY | TRUE | NA | 0 | NA | FALSE | FALSE |
| chr8 | 96009572 | 96009574 | 5 | 5 | MANY | TRUE | NA | 0 | NA | FALSE | FALSE |
| chr8 | 96681043 | 96681059 | 4 | 4 | NA | FALSE | NA | 0 | 1 | FALSE | FALSE |
| chr8 | 96817011 | 96817019 | 4 | 3 | MANY | TRUE | NA | 0 | 1 | FALSE | FALSE |
| chr8 | 97002082 | 97002093 | 4 | 4 | (TA)n | TRUE | NA | 0 | 1 | FALSE | FALSE |
| chr8 | 97682553 | 97682623 | 4 | 4 | MER82 | TRUE | FOXA1 (C-20) | 1 | 1 | TRUE | FALSE |
| chr8 | 97938849 | 97938861 | 4 | 4 | (TTC)n | TRUE | NA | 0 | NA | FALSE | FALSE |
| chr8 | 98175191 | 98175218 | 4 | 2 | MLT1J | TRUE | NA | 0 | 1 | FALSE | FALSE |
| chr8 | 98344309 | 98344376 | 4 | 4 | L1PA7 | TRUE | NA | 0 | 1 | FALSE | FALSE |
| chr8 | 98350171 | 98350240 | 4 | 2 | L1MEg | TRUE | NA | 0 | 1 | FALSE | FALSE |
| chr8 | 98935782 | 98935832 | 4 | 3 | MANY | TRUE | NA | 0 | 1 | TRUE | FALSE |
| chr8 | 98980414 | 98980441 | 4 | 3 | MSTB-int | TRUE | NA | 0 | 1 | FALSE | FALSE |
| chr8 | 99887376 | 99887424 | 4 | 3 | NA | FALSE | NA | 0 | 1 | FALSE | FALSE |
| chr8 | 100078065 | 100078139 | 11 | 7 | L1PA6 | TRUE | NA | 0 | NA | FALSE | FALSE |
| chr8 | 100961031 | 100961043 | 4 | 2 | HERVH-int | TRUE | NA | 0 | NA | FALSE | FALSE |
| chr8 | 101986270 | 101986314 | 4 | 3 | AluSg | TRUE | NA | 0 | NA | TRUE | FALSE |
| chr8 | 102636744 | 102636753 | 6 | 2 | (CAGAA)n | TRUE | NA | 0 | 1 | FALSE | FALSE |
| chr8 | 102902668 | 102902686 | 4 | 3 | MLT1A1 | TRUE | NA | 0 | 1 | TRUE | FALSE |
| chr8 | 103094000 | 103094041 | 4 | 3 | NA | FALSE | NA | 0 | 1 | TRUE | FALSE |
| chr8 | 103535020 | 103535064 | 4 | 4 | MANY | TRUE | NA | 0 | 1 | TRUE | FALSE |
| chr8 | 103671625 | 103671663 | 4 | 3 | AluYc | TRUE | NA | 0 | 1 | TRUE | FALSE |
| chr8 | 103954713 | 103954728 | 6 | 4 | (CATCC)n | TRUE | NA | 0 | NA | TRUE | FALSE |
| chr8 | 104480740 | 104480783 | 5 | 3 | AluY | TRUE | NA | 0 | NA | FALSE | FALSE |
| chr8 | 104682184 | 104682202 | 5 | 4 | THE1B-int | TRUE | NA | 0 | NA | FALSE | FALSE |
| chr8 | 104732426 | 104732432 | 4 | 2 | NA | FALSE | NA | 0 | 1 | FALSE | FALSE |
| chr8 | 105278928 | 105278934 | 4 | 3 | NA | FALSE | NA | 0 | 1 | FALSE | FALSE |
| chr8 | 105999386 | 105999402 | 4 | 2 | NA | FALSE | NA | 0 | 1 | FALSE | FALSE |
| chr8 | 106127495 | 106127504 | 4 | 4 | NA | FALSE | NA | 0 | 1 | FALSE | FALSE |
| chr8 | 106212843 | 106212851 | 4 | 3 | L1PA10 | TRUE | NA | 0 | NA | FALSE | FALSE |
| chr8 | 106221274 | 106221283 | 4 | 3 | NA | FALSE | GATA-1 | 1 | 1 | FALSE | FALSE |
| chr8 | 106261789 | 106261798 | 4 | 4 | L1M4 | TRUE | NA | 0 | 1 | FALSE | FALSE |
| chr8 | 106699135 | 106699141 | 4 | 2 | NA | FALSE | NA | 0 | 1 | FALSE | FALSE |
| chr8 | 106883776 | 106883780 | 5 | 4 | (CA)n | TRUE | NA | 0 | NA | TRUE | FALSE |
| chr8 | 107158265 | 107158273 | 5 | 3 | (TG)n | TRUE | NA | 0 | NA | FALSE | FALSE |
| chr8 | 107356794 | 107356822 | 4 | 2 | AluSc8 | TRUE | NA | 0 | NA | FALSE | FALSE |
| chr8 | 107963423 | 107963435 | 4 | 4 | L2a | TRUE | NA | 0 | 1 | FALSE | FALSE |
| chr8 | 108155430 | 108155438 | 4 | 3 | NA | FALSE | NA | 0 | 1 | FALSE | FALSE |
| chr8 | 108559440 | 108559455 | 4 | 4 | L1PA10 | TRUE | NA | 0 | NA | FALSE | FALSE |
| chr8 | 108616514 | 108616522 | 4 | 4 | HERV30-int | TRUE | NA | 0 | NA | FALSE | FALSE |
| chr8 | 108745042 | 108745055 | 4 | 4 | NA | FALSE | NA | 0 | 1 | FALSE | FALSE |
| chr8 | 108869662 | 108869664 | 4 | 4 | (GA)n | TRUE | NA | 0 | NA | TRUE | FALSE |
| chr8 | 108904249 | 108904262 | 7 | 7 | L1PA3 | TRUE | NA | 0 | NA | FALSE | FALSE |
| chr8 | 109782149 | 109782167 | 4 | 4 | NA | FALSE | NA | 0 | 1 | TRUE | FALSE |
| chr8 | 109984099 | 109984115 | 4 | 3 | L1MA4A | TRUE | NA | 0 | 1 | FALSE | FALSE |
| chr8 | 109992480 | 109992494 | 5 | 5 | NA | FALSE | NA | 0 | 1 | FALSE | FALSE |
| chr8 | 110047593 | 110047607 | 5 | 5 | L1ME3F | TRUE | NA | 0 | 1 | FALSE | FALSE |
| chr8 | 110067381 | 110067388 | 4 | 4 | NA | FALSE | NA | 0 | 1 | FALSE | FALSE |
| chr8 | 110077409 | 110077435 | 4 | 2 | MER103C | TRUE | NA | 0 | 1 | TRUE | FALSE |
| chr8 | 110109427 | 110109458 | 6 | 4 | NA | FALSE | NA | 0 | 1 | FALSE | FALSE |
| chr8 | 110169303 | 110169326 | 4 | 2 | NA | FALSE | NA | 0 | 1 | FALSE | FALSE |
| chr8 | 110452473 | 110452497 | 4 | 3 | NA | FALSE | NA | 0 | 1 | FALSE | FALSE |
| chr8 | 110767146 | 110767159 | 4 | 4 | AluY | TRUE | NA | 0 | NA | FALSE | FALSE |
| chr8 | 110781212 | 110781232 | 4 | 4 | NA | FALSE | NA | 0 | 1 | FALSE | FALSE |
| chr8 | 110806843 | 110806860 | 4 | 4 | NA | FALSE | NA | 0 | 1 | FALSE | FALSE |
| chr8 | 110893792 | 110893810 | 4 | 3 | LTR26B | TRUE | NA | 0 | 1 | FALSE | FALSE |
| chr8 | 110943016 | 110943034 | 4 | 4 | L1M5 | TRUE | NA | 0 | 1 | FALSE | FALSE |
| chr8 | 110959023 | 110959023 | 4 | 4 | THE1A-int | TRUE | NA | 0 | 1 | FALSE | FALSE |
| chr8 | 111184397 | 111184404 | 4 | 4 | THE1B-int | TRUE | NA | 0 | NA | FALSE | FALSE |
| chr8 | 111269264 | 111269271 | 4 | 2 | MER11C | TRUE | NA | 0 | NA | FALSE | FALSE |
| chr8 | 111275060 | 111275068 | 4 | 4 | MANY | TRUE | NA | 0 | NA | FALSE | FALSE |
| chr8 | 111644187 | 111644206 | 4 | 3 | NA | FALSE | NA | 0 | 1 | FALSE | FALSE |
| chr8 | 111804465 | 111804478 | 4 | 3 | MER21-int | TRUE | NA | 0 | 1 | TRUE | FALSE |
| chr8 | 111806992 | 111807005 | 4 | 3 | AluY | TRUE | NA | 0 | NA | FALSE | FALSE |
| chr8 | 111987994 | 111988020 | 4 | 3 | L2a | TRUE | NA | 0 | 1 | FALSE | FALSE |
| chr8 | 112324241 | 112324243 | 4 | 4 | (TATATG)n | TRUE | NA | 0 | NA | FALSE | FALSE |
| chr8 | 112360987 | 112361018 | 4 | 4 | G-rich | TRUE | NA | 0 | 1 | FALSE | FALSE |
| chr8 | 112756166 | 112756178 | 4 | 4 | (TC)n | TRUE | NA | 0 | NA | FALSE | FALSE |
| chr8 | 113194368 | 113194386 | 4 | 4 | NA | FALSE | NA | 0 | 1 | FALSE | FALSE |
| chr8 | 113831720 | 113831725 | 4 | 4 | L3 | TRUE | NA | 0 | 1 | FALSE | FALSE |
| chr8 | 113944337 | 113944347 | 4 | 4 | L1MA4A | TRUE | NA | 0 | 1 | FALSE | FALSE |
| chr8 | 114121559 | 114121569 | 4 | 2 | MLT-int | TRUE | NA | 0 | 1 | FALSE | FALSE |
| chr8 | 114312571 | 114312578 | 4 | 3 | L1PA16 | TRUE | NA | 0 | 1 | FALSE | FALSE |
| chr8 | 114791042 | 114791052 | 4 | 2 | AluSx1 | TRUE | NA | 0 | NA | FALSE | FALSE |
| chr8 | 114954202 | 114954209 | 4 | 3 | NA | FALSE | NA | 0 | 1 | FALSE | FALSE |
| chr8 | 115149659 | 115149660 | 4 | 3 | NA | FALSE | NA | 0 | 1 | FALSE | FALSE |
| chr8 | 115309589 | 115309598 | 4 | 4 | L1ME1 | TRUE | NA | 0 | 1 | FALSE | FALSE |
| chr8 | 115311261 | 115311261 | 4 | 4 | AluSz | TRUE | NA | 0 | NA | FALSE | FALSE |
| chr8 | 115349214 | 115349231 | 4 | 3 | L2a | TRUE | NA | 0 | 1 | FALSE | FALSE |
| chr8 | 115390146 | 115390147 | 5 | 5 | L1PA16 | TRUE | NA | 0 | NA | FALSE | FALSE |
| chr8 | 115729408 | 115729409 | 4 | 2 | NA | FALSE | NA | 0 | 1 | FALSE | FALSE |
| chr8 | 115887450 | 115887473 | 4 | 4 | MANY | TRUE | NA | 0 | 1 | FALSE | FALSE |
| chr8 | 117612051 | 117612089 | 5 | 4 | L1PA13 | TRUE | NA | 0 | NA | FALSE | FALSE |
| chr8 | 117768124 | 117768142 | 4 | 4 | NA | FALSE | MANY | 37 | 1 | TRUE | TRUE |
| chr8 | 117809348 | 117809368 | 4 | 3 | AluSx | TRUE | NA | 0 | NA | FALSE | FALSE |
| chr8 | 118097805 | 118097828 | 5 | 5 | NA | FALSE | NA | 0 | 1 | TRUE | FALSE |
| chr8 | 118749966 | 118749993 | 4 | 3 | NA | FALSE | NA | 0 | 1 | FALSE | FALSE |
| chr8 | 119389726 | 119389742 | 4 | 3 | NA | FALSE | NA | 0 | 1 | TRUE | FALSE |
| chr8 | 120019075 | 120019076 | 4 | 3 | NA | FALSE | NA | 0 | 1 | FALSE | FALSE |
| chr8 | 120177160 | 120177169 | 4 | 2 | L1PA2 | TRUE | NA | 0 | NA | FALSE | FALSE |
| chr8 | 120185710 | 120185734 | 4 | 4 | NA | FALSE | NA | 0 | 1 | FALSE | FALSE |
| chr8 | 120251987 | 120252026 | 6 | 5 | L1PA3 | TRUE | NA | 0 | NA | FALSE | FALSE |
| chr8 | 120300184 | 120300189 | 4 | 2 | L1PA6 | TRUE | NA | 0 | 1 | FALSE | FALSE |
| chr8 | 120511409 | 120511423 | 6 | 5 | NA | FALSE | NA | 0 | NA | TRUE | FALSE |
| chr8 | 121151815 | 121151867 | 4 | 3 | NA | FALSE | NA | 0 | 1 | FALSE | FALSE |
| chr8 | 122571219 | 122571240 | 7 | 7 | (TG)n | TRUE | NA | 0 | NA | FALSE | FALSE |
| chr8 | 122955198 | 122955235 | 4 | 3 | NA | FALSE | NA | 0 | 1 | TRUE | FALSE |
| chr8 | 124050899 | 124050931 | 4 | 3 | NA | FALSE | NA | 0 | 1 | TRUE | FALSE |
| chr8 | 124054556 | 124054557 | 9 | 6 | NA | FALSE | MANY | 22 | 1 | TRUE | TRUE |
| chr8 | 124428695 | 124428798 | 5 | 5 | NA | FALSE | MANY | 24 | 1 | TRUE | TRUE |
| chr8 | 125191902 | 125191946 | 4 | 4 | (TG)n | TRUE | NA | 0 | NA | FALSE | FALSE |
| chr8 | 125441488 | 125441503 | 7 | 4 | A-rich | TRUE | NA | 0 | NA | FALSE | FALSE |
| chr8 | 125551343 | 125551344 | 4 | 3 | NA | FALSE | MANY | 42 | 1 | TRUE | TRUE |
| chr8 | 125809328 | 125809331 | 5 | 3 | (CA)n | TRUE | CEBPB | 1 | NA | FALSE | FALSE |
| chr8 | 125985493 | 125985546 | 4 | 4 | NA | FALSE | MANY | 23 | 1 | TRUE | TRUE |
| chr8 | 126294653 | 126294661 | 4 | 2 | MANY | TRUE | NA | 0 | 1 | FALSE | FALSE |
| chr8 | 126574874 | 126574876 | 5 | 4 | (CA)n | TRUE | STAT3 | 1 | NA | TRUE | FALSE |
| chr8 | 127178261 | 127178298 | 4 | 4 | (TTCC)n | TRUE | NA | 0 | 1 | FALSE | FALSE |
| chr8 | 127791698 | 127791739 | 4 | 2 | LTR19B | TRUE | MANY | 2 | 1 | TRUE | FALSE |
| chr8 | 129295997 | 129296001 | 5 | 4 | MANY | TRUE | NA | 0 | NA | TRUE | FALSE |
| chr8 | 129614227 | 129614227 | 4 | 4 | (CA)n | TRUE | NA | 0 | 1 | FALSE | FALSE |
| chr8 | 129906838 | 129906840 | 4 | 4 | L1PA2 | TRUE | NA | 0 | NA | FALSE | FALSE |
| chr8 | 130509858 | 130509902 | 5 | 3 | (TA)n | TRUE | NA | 0 | 1 | FALSE | FALSE |
| chr8 | 131288971 | 131288979 | 4 | 4 | MANY | TRUE | NA | 0 | NA | TRUE | FALSE |
| chr8 | 131559146 | 131559177 | 5 | 4 | NA | FALSE | NA | 0 | 1 | FALSE | FALSE |
| chr8 | 132249806 | 132249812 | 4 | 3 | L1PA4 | TRUE | NA | 0 | NA | FALSE | FALSE |
| chr8 | 132326372 | 132326395 | 5 | 4 | HERVH-int | TRUE | NA | 0 | 1 | FALSE | FALSE |
| chr8 | 132593362 | 132593379 | 4 | 4 | NA | FALSE | NA | 0 | 1 | FALSE | FALSE |
| chr8 | 132626051 | 132626061 | 4 | 3 | NA | FALSE | NA | 0 | 1 | FALSE | FALSE |
| chr8 | 132659343 | 132659354 | 4 | 4 | NA | FALSE | NA | 0 | 1 | FALSE | FALSE |
| chr8 | 132756379 | 132756400 | 4 | 4 | HERVH-int | TRUE | NA | 0 | NA | FALSE | FALSE |
| chr8 | 132756437 | 132756445 | 4 | 2 | HERVH-int | TRUE | NA | 0 | 1 | FALSE | FALSE |
| chr8 | 133176682 | 133176699 | 5 | 4 | NA | FALSE | NA | 0 | 1 | FALSE | FALSE |
| chr8 | 133191108 | 133191129 | 4 | 4 | LTR16A1 | TRUE | NA | 0 | 1 | FALSE | FALSE |
| chr8 | 133286161 | 133286173 | 4 | 4 | THE1B-int | TRUE | NA | 0 | 1 | FALSE | FALSE |
| chr8 | 133356785 | 133356823 | 5 | 5 | MIR | TRUE | NA | 0 | 1 | FALSE | FALSE |
| chr8 | 133365409 | 133365426 | 4 | 4 | AluSq | TRUE | NA | 0 | NA | FALSE | FALSE |
| chr8 | 133554507 | 133554525 | 4 | 2 | AluSz | TRUE | NA | 0 | NA | FALSE | FALSE |
| chr8 | 134264840 | 134264875 | 4 | 4 | NA | FALSE | NA | 0 | 1 | TRUE | FALSE |
| chr8 | 135068759 | 135068788 | 4 | 3 | NA | FALSE | NA | 0 | 1 | FALSE | FALSE |
| chr8 | 135257054 | 135257073 | 5 | 4 | L1PA17 | TRUE | NA | 0 | 1 | FALSE | FALSE |
| chr8 | 135691556 | 135691581 | 5 | 2 | L1PA7 | TRUE | NA | 0 | NA | FALSE | FALSE |
| chr8 | 135844682 | 135844689 | 4 | 4 | NA | FALSE | MANY | 34 | 1 | TRUE | TRUE |
| chr8 | 136788865 | 136788905 | 6 | 3 | LTR16A | TRUE | NA | 0 | 1 | FALSE | FALSE |
| chr8 | 136872767 | 136872800 | 4 | 3 | AluSp | TRUE | NA | 0 | NA | FALSE | FALSE |
| chr8 | 137385134 | 137385158 | 4 | 2 | NA | FALSE | NA | 0 | 1 | FALSE | FALSE |
| chr8 | 137479896 | 137479902 | 4 | 3 | L2 | TRUE | MANY | 4 | 1 | TRUE | TRUE |
| chr8 | 137827008 | 137827017 | 4 | 3 | NA | FALSE | NA | 0 | 1 | FALSE | FALSE |
| chr8 | 138375602 | 138375606 | 4 | 3 | NA | FALSE | NA | 0 | 1 | FALSE | FALSE |
| chr8 | 138743973 | 138743985 | 4 | 3 | MLT1A1 | TRUE | NA | 0 | 1 | FALSE | FALSE |
| chr8 | 138826854 | 138826864 | 4 | 2 | L1PA4 | TRUE | NA | 0 | NA | FALSE | FALSE |
| chr8 | 138837279 | 138837287 | 4 | 3 | MANY | TRUE | NA | 0 | NA | FALSE | FALSE |
| chr8 | 138859586 | 138859594 | 4 | 3 | NA | FALSE | MANY | 3 | 1 | TRUE | TRUE |
| chr8 | 138965300 | 138965308 | 4 | 4 | NA | FALSE | NA | 0 | 1 | FALSE | FALSE |
| chr8 | 139141588 | 139141603 | 4 | 4 | L2b | TRUE | NA | 0 | 1 | FALSE | FALSE |
| chr8 | 140131925 | 140131946 | 4 | 3 | NA | FALSE | NA | 0 | 1 | FALSE | FALSE |
| chr8 | 140147321 | 140147322 | 4 | 2 | NA | FALSE | NA | 0 | 1 | FALSE | FALSE |
| chr8 | 140232185 | 140232202 | 4 | 3 | L1MC3 | TRUE | NA | 0 | 1 | FALSE | FALSE |
| chr8 | 140423940 | 140423962 | 4 | 4 | AluSg4 | TRUE | NA | 0 | NA | FALSE | FALSE |
| chr8 | 141282218 | 141282233 | 5 | 3 | HAL1-2a MD | TRUE | NA | 0 | NA | FALSE | FALSE |
| chr8 | 141585333 | 141585369 | 4 | 3 | NA | FALSE | NA | 0 | 1 | TRUE | FALSE |
| chr8 | 142044897 | 142044902 | 4 | 2 | NA | FALSE | MANY | 3 | 1 | TRUE | TRUE |
| chr8 | 142083611 | 142083619 | 4 | 4 | MANY | TRUE | MANY | 10 | NA | TRUE | FALSE |
| chr8 | 142212393 | 142212458 | 7 | 5 | SVA D | TRUE | NA | 0 | 1 | FALSE | FALSE |
| chr8 | 142368662 | 142368699 | 4 | 1 | NA | FALSE | NA | 0 | 1 | TRUE | FALSE |
| chr8 | 142491940 | 142491963 | 4 | 2 | NA | FALSE | NA | 0 | 1 | TRUE | FALSE |
| chr8 | 142538176 | 142538186 | 4 | 3 | NA | FALSE | NA | 0 | 1 | TRUE | FALSE |
| chr8 | 143527207 | 143527240 | 4 | 4 | NA | FALSE | NA | 0 | NA | FALSE | FALSE |
| chr8 | 143775327 | 143775339 | 5 | 4 | MANY | TRUE | CTCF | 1 | 1 | TRUE | FALSE |
| chr8 | 143836431 | 143836433 | 4 | 2 | NA | FALSE | NA | 0 | 1 | FALSE | FALSE |
| chr8 | 143968837 | 143968841 | 4 | 3 | (GA)n | TRUE | NA | 0 | NA | FALSE | FALSE |
| chr8 | 144025793 | 144025821 | 4 | 4 | NA | FALSE | NRSF | 1 | 1 | FALSE | FALSE |
| chr8 | 144096916 | 144096938 | 6 | 5 | NA | FALSE | ZNF263 | 1 | NA | FALSE | FALSE |
| chr8 | 144122163 | 144122185 | 6 | 4 | MER11C | TRUE | NA | 0 | NA | FALSE | FALSE |
| chr8 | 144286330 | 144286347 | 4 | 3 | NA | FALSE | NA | 0 | 1 | TRUE | FALSE |
| chr8 | 144342172 | 144342201 | 4 | 4 | NA | FALSE | NA | 0 | 1 | TRUE | FALSE |
| chr8 | 144390005 | 144390054 | 6 | 5 | NA | FALSE | NA | 0 | NA | FALSE | FALSE |
| chr8 | 144424398 | 144424398 | 5 | 5 | NA | FALSE | NA | 0 | NA | FALSE | FALSE |
| chr8 | 144490249 | 144490264 | 6 | 3 | (CCCCG)n | TRUE | MANY | 7 | 1 | TRUE | TRUE |
| chr8 | 144743757 | 144743810 | 4 | 3 | NA | FALSE | NA | 0 | 1 | FALSE | FALSE |
| chr8 | 144748995 | 144749004 | 4 | 4 | NA | FALSE | NA | 0 | NA | FALSE | FALSE |
| chr8 | 144856674 | 144856736 | 4 | 4 | L1MD2 | TRUE | NA | 0 | 1 | TRUE | FALSE |
| chr8 | 145064155 | 145064186 | 4 | 4 | (CCCCG)n | TRUE | MANY | 9 | 1 | TRUE | TRUE |
| chr8 | 145601494 | 145601579 | 4 | 3 | NA | FALSE | NA | 0 | 1 | TRUE | FALSE |
| chr8 | 145787783 | 145787871 | 4 | 3 | NA | FALSE | MANY | 2 | NA | TRUE | FALSE |
| chr8 | 145912566 | 145912616 | 4 | 3 | NA | FALSE | Pol2 | 1 | NA | TRUE | FALSE |
| chr8 | 146017833 | 146017919 | 4 | 4 | NA | FALSE | MANY | 43 | 1 | TRUE | TRUE |
| chr8 | 146037336 | 146037476 | 7 | 6 | NA | FALSE | NA | 0 | NA | FALSE | FALSE |
| chr8 | 146297347 | 146297351 | 4 | 2 | HERVE-int | TRUE | NA | 0 | NA | FALSE | FALSE |
| chr8 | 146297816 | 146298025 | 7 | 4 | AluSp | TRUE | NA | 0 | 1 | FALSE | FALSE |
| chr9 | 46431 | 46500 | 4 | 4 | MER45B | TRUE | Egr-1 | 1 | 1 | TRUE | FALSE |
| chr9 | 72793 | 72867 | 4 | 3 | NA | FALSE | NA | 0 | 1 | FALSE | FALSE |
| chr9 | 105285 | 105285 | 6 | 6 | NA | FALSE | NA | 0 | NA | FALSE | FALSE |
| chr9 | 118265 | 118448 | 8 | 5 | NA | FALSE | NA | 0 | 1 | TRUE | FALSE |
| chr9 | 157255 | 157274 | 5 | 4 | NA | FALSE | NA | 0 | NA | FALSE | FALSE |
| chr9 | 400167 | 400265 | 7 | 5 | (CCA)n | TRUE | NA | 0 | 1 | FALSE | FALSE |
| chr9 | 1290981 | 1290989 | 4 | 4 | NA | FALSE | NA | 0 | 1 | FALSE | FALSE |
| chr9 | 1319967 | 1319979 | 4 | 3 | NA | FALSE | NA | 0 | 1 | FALSE | FALSE |
| chr9 | 1423167 | 1423181 | 6 | 3 | L1PA4 | TRUE | NA | 0 | 1 | FALSE | FALSE |
| chr9 | 1732438 | 1732459 | 4 | 4 | NA | FALSE | NA | 0 | 1 | FALSE | FALSE |
| chr9 | 3041153 | 3041204 | 4 | 3 | NA | FALSE | NA | 0 | 1 | FALSE | FALSE |
| chr9 | 3091267 | 3091287 | 4 | 4 | L2a | TRUE | NA | 0 | 1 | FALSE | FALSE |
| chr9 | 3102932 | 3102962 | 4 | 2 | L1ME2z | TRUE | NA | 0 | 1 | FALSE | FALSE |
| chr9 | 3107795 | 3107828 | 4 | 4 | L1PA5 | TRUE | NA | 0 | NA | FALSE | FALSE |
| chr9 | 3526038 | 3526039 | 4 | 3 | NA | FALSE | MANY | 14 | 1 | TRUE | TRUE |
| chr9 | 3668437 | 3668485 | 4 | 3 | NA | FALSE | NA | 0 | 1 | TRUE | FALSE |
| chr9 | 4855065 | 4855065 | 5 | 5 | AluSg | TRUE | MANY | 4 | NA | TRUE | FALSE |
| chr9 | 5255033 | 5255092 | 4 | 3 | L1PA6 | TRUE | NA | 0 | NA | TRUE | FALSE |
| chr9 | 5625877 | 5625884 | 4 | 2 | AluSz6 | TRUE | NA | 0 | NA | FALSE | FALSE |
| chr9 | 6102526 | 6102572 | 4 | 2 | HERVL-int | TRUE | NA | 0 | 1 | TRUE | FALSE |
| chr9 | 6603735 | 6603736 | 4 | 2 | AluSg | TRUE | NA | 0 | NA | FALSE | FALSE |
| chr9 | 6633308 | 6633350 | 4 | 1 | NA | FALSE | NA | 0 | 1 | TRUE | FALSE |
| chr9 | 6801638 | 6801642 | 4 | 3 | (TC)n | TRUE | NA | 0 | NA | FALSE | FALSE |
| chr9 | 7038601 | 7038648 | 4 | 3 | NA | FALSE | NA | 0 | 1 | FALSE | FALSE |
| chr9 | 8289057 | 8289084 | 4 | 3 | NA | FALSE | NA | 0 | 1 | FALSE | FALSE |
| chr9 | 8969127 | 8969149 | 4 | 4 | L1M5 | TRUE | NA | 0 | 1 | FALSE | FALSE |
| chr9 | 9646317 | 9646343 | 4 | 3 | (TG)n | TRUE | NA | 0 | NA | FALSE | FALSE |
| chr9 | 10517003 | 10517035 | 4 | 3 | L1MB1 | TRUE | NA | 0 | 1 | FALSE | FALSE |
| chr9 | 10630330 | 10630360 | 4 | 2 | MIR3 | TRUE | NA | 0 | 1 | FALSE | FALSE |
| chr9 | 12949423 | 12949473 | 4 | 2 | HERVH-int | TRUE | NA | 0 | 1 | FALSE | FALSE |
| chr9 | 14861265 | 14861301 | 7 | 5 | (TA)n | TRUE | NA | 0 | NA | FALSE | FALSE |
| chr9 | 15976328 | 15976352 | 4 | 3 | NA | FALSE | NA | 0 | 1 | FALSE | FALSE |
| chr9 | 18561309 | 18561360 | 4 | 3 | L1PA7 | TRUE | NA | 0 | NA | FALSE | FALSE |
| chr9 | 19102939 | 19103000 | 5 | 3 | NA | FALSE | MANY | 40 | 1 | TRUE | TRUE |
| chr9 | 19122085 | 19122106 | 4 | 3 | AluSx | TRUE | NA | 0 | NA | FALSE | FALSE |
| chr9 | 19608745 | 19608790 | 4 | 4 | MANY | TRUE | NA | 0 | 1 | FALSE | FALSE |
| chr9 | 19797419 | 19797454 | 4 | 3 | HAL1 | TRUE | NA | 0 | 1 | FALSE | FALSE |
| chr9 | 20068645 | 20068675 | 4 | 3 | NA | FALSE | NA | 0 | 1 | FALSE | FALSE |
| chr9 | 20195440 | 20195448 | 4 | 4 | L1ME3B | TRUE | NA | 0 | 1 | FALSE | FALSE |
| chr9 | 20220166 | 20220185 | 4 | 3 | NA | FALSE | NA | 0 | 1 | FALSE | FALSE |
| chr9 | 20283684 | 20283730 | 4 | 3 | MER50B | TRUE | NA | 0 | 1 | FALSE | FALSE |
| chr9 | 21124253 | 21124299 | 4 | 2 | MANY | TRUE | NA | 0 | 1 | FALSE | FALSE |
| chr9 | 21202039 | 21202104 | 4 | 2 | NA | FALSE | NA | 0 | 1 | FALSE | FALSE |
| chr9 | 21211474 | 21211522 | 4 | 2 | L1PREC2 | TRUE | NA | 0 | 1 | FALSE | FALSE |
| chr9 | 21490884 | 21490898 | 4 | 4 | HERVH-int | TRUE | NA | 0 | 1 | FALSE | FALSE |
| chr9 | 23341999 | 23342017 | 4 | 3 | NA | FALSE | NA | 0 | 1 | FALSE | FALSE |
| chr9 | 24125821 | 24125822 | 4 | 3 | NA | FALSE | NA | 0 | 1 | FALSE | FALSE |
| chr9 | 24291845 | 24291857 | 4 | 4 | THE1B-int | TRUE | NA | 0 | NA | FALSE | FALSE |
| chr9 | 26156653 | 26156653 | 4 | 4 | NA | FALSE | NA | 0 | 1 | FALSE | FALSE |
| chr9 | 26165188 | 26165189 | 4 | 3 | NA | FALSE | NA | 0 | 1 | FALSE | FALSE |
| chr9 | 26835726 | 26835768 | 4 | 4 | L1PA5 | TRUE | NA | 0 | NA | FALSE | FALSE |
| chr9 | 29259438 | 29259471 | 5 | 5 | MANY | TRUE | NA | 0 | NA | FALSE | FALSE |
| chr9 | 29418558 | 29418612 | 4 | 4 | Charlie2b | TRUE | NA | 0 | 1 | FALSE | FALSE |
| chr9 | 31947881 | 31947883 | 4 | 2 | L1PA5 | TRUE | NA | 0 | NA | FALSE | FALSE |
| chr9 | 32057098 | 32057099 | 4 | 3 | NA | FALSE | NA | 0 | 1 | FALSE | FALSE |
| chr9 | 33183503 | 33183525 | 5 | 4 | L1PA3 | TRUE | NA | 0 | NA | FALSE | FALSE |
| chr9 | 33383031 | 33383109 | 5 | 4 | NA | FALSE | NA | 0 | 1 | FALSE | FALSE |
| chr9 | 33383772 | 33383798 | 6 | 5 | NA | FALSE | NA | 0 | 1 | FALSE | FALSE |
| chr9 | 33388178 | 33388215 | 4 | 4 | NA | FALSE | NA | 0 | 1 | TRUE | FALSE |
| chr9 | 33390692 | 33390725 | 4 | 4 | NA | FALSE | NA | 0 | 1 | TRUE | FALSE |
| chr9 | 33391521 | 33391550 | 5 | 5 | NA | FALSE | MANY | 13 | NA | TRUE | FALSE |
| chr9 | 33391833 | 33391899 | 4 | 2 | MIRb | TRUE | MANY | 3 | 1 | TRUE | TRUE |
| chr9 | 33393089 | 33393119 | 5 | 2 | AluSx | TRUE | NA | 0 | NA | FALSE | FALSE |
| chr9 | 33394076 | 33394122 | 6 | 3 | L2b | TRUE | NA | 0 | 1 | TRUE | FALSE |
| chr9 | 33424156 | 33424221 | 6 | 5 | SVA E | TRUE | NA | 0 | NA | FALSE | FALSE |
| chr9 | 33473996 | 33474044 | 5 | 4 | NA | FALSE | MANY | 34 | 1 | TRUE | TRUE |
| chr9 | 33573083 | 33573136 | 4 | 2 | L1MC4 | TRUE | NA | 0 | 1 | TRUE | FALSE |
| chr9 | 33597080 | 33597082 | 4 | 2 | MSTA | TRUE | NA | 0 | NA | FALSE | FALSE |
| chr9 | 33793587 | 33793605 | 7 | 3 | NA | FALSE | NA | 0 | 1 | TRUE | FALSE |
| chr9 | 34416270 | 34416319 | 4 | 3 | GA-rich | TRUE | NA | 0 | 1 | TRUE | FALSE |
| chr9 | 35116157 | 35116238 | 4 | 3 | NA | FALSE | MANY | 8 | 1 | TRUE | TRUE |
| chr9 | 36617482 | 36617561 | 4 | 2 | AluJr | TRUE | NA | 0 | 1 | FALSE | FALSE |
| chr9 | 36869870 | 36869935 | 4 | 3 | (TGGA)n | TRUE | NA | 0 | NA | FALSE | FALSE |
| chr9 | 36913772 | 36913851 | 4 | 3 | NA | FALSE | CTCF | 1 | 1 | TRUE | FALSE |
| chr9 | 37061795 | 37061850 | 4 | 3 | L2b | TRUE | NA | 0 | 1 | TRUE | FALSE |
| chr9 | 37120366 | 37120418 | 4 | 4 | NA | FALSE | MANY | 16 | 1 | TRUE | TRUE |
| chr9 | 37384160 | 37384210 | 4 | 2 | (TTCC)n | TRUE | NA | 0 | NA | TRUE | FALSE |
| chr9 | 37609151 | 37609221 | 4 | 3 | AluSp | TRUE | NA | 0 | 1 | FALSE | FALSE |
| chr9 | 37612193 | 37612286 | 4 | 3 | NA | FALSE | NA | 0 | 1 | TRUE | FALSE |
| chr9 | 37695798 | 37695822 | 4 | 4 | L2a | TRUE | NA | 0 | 1 | FALSE | FALSE |
| chr9 | 38242682 | 38242688 | 5 | 2 | SVA F | TRUE | NA | 0 | NA | FALSE | FALSE |
| chr9 | 38280737 | 38280759 | 4 | 3 | NA | FALSE | NA | 0 | 1 | FALSE | FALSE |
| chr9 | 38462894 | 38462915 | 4 | 4 | L1ME1 | TRUE | NA | 0 | 1 | FALSE | FALSE |
| chr9 | 38564618 | 38564641 | 5 | 3 | MSTA-int | TRUE | NA | 0 | 1 | TRUE | FALSE |
| chr9 | 39004600 | 39004634 | 4 | 3 | L2a | TRUE | NA | 0 | 1 | FALSE | FALSE |
| chr9 | 39008645 | 39008692 | 4 | 4 | NA | FALSE | NA | 0 | 1 | FALSE | FALSE |
| chr9 | 39060167 | 39060209 | 5 | 5 | NA | FALSE | NA | 0 | 1 | FALSE | FALSE |
| chr9 | 39142301 | 39142340 | 5 | 4 | NA | FALSE | NA | 0 | 1 | FALSE | FALSE |
| chr9 | 39145813 | 39145874 | 4 | 2 | NA | FALSE | NA | 0 | 1 | FALSE | FALSE |
| chr9 | 39152881 | 39152925 | 5 | 3 | AluY | TRUE | NA | 0 | NA | FALSE | FALSE |
| chr9 | 39770640 | 39770882 | 5 | 5 | NA | FALSE | NA | 0 | 1 | FALSE | FALSE |
| chr9 | 39782167 | 39782571 | 6 | 5 | MANY | TRUE | NA | 0 | 1 | FALSE | FALSE |
| chr9 | 39829416 | 39829672 | 4 | 4 | L1M4c | TRUE | NA | 0 | NA | FALSE | FALSE |
| chr9 | 40319857 | 40319901 | 5 | 3 | L1MB8 | TRUE | NA | 0 | 1 | FALSE | FALSE |
| chr9 | 40415269 | 40415373 | 4 | 3 | MANY | TRUE | NA | 0 | NA | TRUE | FALSE |
| chr9 | 40495941 | 40496055 | 4 | 3 | NA | FALSE | NA | 0 | 1 | FALSE | FALSE |
| chr9 | 40633751 | 40633810 | 6 | 6 | NA | FALSE | MANY | 2 | 1 | TRUE | FALSE |
| chr9 | 40804409 | 40804443 | 5 | 4 | MER3 | TRUE | NA | 0 | 1 | FALSE | FALSE |
| chr9 | 41918322 | 41918597 | 4 | 3 | (TA)n | TRUE | NA | 0 | 1 | FALSE | FALSE |
| chr9 | 41966843 | 41967047 | 4 | 4 | NA | FALSE | NA | 0 | 1 | FALSE | FALSE |
| chr9 | 41973998 | 41974355 | 6 | 5 | MANY | TRUE | NA | 0 | 1 | FALSE | FALSE |
| chr9 | 42251171 | 42251339 | 4 | 4 | MANY | TRUE | MANY | 2 | 1 | TRUE | FALSE |
| chr9 | 42972909 | 42972952 | 4 | 4 | AluYc | TRUE | NA | 0 | NA | FALSE | FALSE |
| chr9 | 43141677 | 43141744 | 5 | 4 | AluSg7 | TRUE | NA | 0 | NA | FALSE | FALSE |
| chr9 | 43316209 | 43316259 | 6 | 3 | NA | FALSE | NA | 0 | 1 | FALSE | FALSE |
| chr9 | 43447866 | 43447908 | 4 | 4 | AluSx1 | TRUE | NA | 0 | 1 | TRUE | FALSE |
| chr9 | 43526513 | 43526533 | 5 | 4 | NA | FALSE | NA | 0 | 1 | FALSE | FALSE |
| chr9 | 43583454 | 43583477 | 5 | 3 | L1PA13 | TRUE | NA | 0 | 1 | FALSE | FALSE |
| chr9 | 43599114 | 43599190 | 6 | 5 | AluY | TRUE | NA | 0 | NA | FALSE | FALSE |
| chr9 | 43648528 | 43648570 | 5 | 5 | L1ME3D | TRUE | NA | 0 | 1 | FALSE | FALSE |
| chr9 | 43653197 | 43653253 | 4 | 3 | MANY | TRUE | NA | 0 | NA | FALSE | FALSE |
| chr9 | 44058952 | 44059007 | 5 | 5 | NA | FALSE | NA | 0 | 1 | FALSE | FALSE |
| chr9 | 44069917 | 44069963 | 4 | 4 | NA | FALSE | NA | 0 | 1 | FALSE | FALSE |
| chr9 | 44100312 | 44100360 | 4 | 2 | (TTTTTG)n | TRUE | NA | 0 | 1 | FALSE | FALSE |
| chr9 | 44176825 | 44176890 | 4 | 4 | NA | FALSE | NA | 0 | 1 | TRUE | FALSE |
| chr9 | 44181605 | 44181683 | 4 | 4 | L4 | TRUE | NA | 0 | 1 | FALSE | FALSE |
| chr9 | 44192939 | 44192971 | 4 | 4 | L1PREC2 | TRUE | NA | 0 | NA | FALSE | FALSE |
| chr9 | 44214750 | 44214828 | 5 | 5 | L1MA5 | TRUE | NA | 0 | 1 | FALSE | FALSE |
| chr9 | 44227724 | 44227790 | 4 | 4 | NA | FALSE | NA | 0 | 1 | FALSE | FALSE |
| chr9 | 44436805 | 44436831 | 4 | 3 | NA | FALSE | NA | 0 | 1 | FALSE | FALSE |
| chr9 | 44729058 | 44729139 | 4 | 2 | MANY | TRUE | NA | 0 | 1 | FALSE | FALSE |
| chr9 | 44774819 | 44774898 | 4 | 3 | AluSc8 | TRUE | NA | 0 | 1 | FALSE | FALSE |
| chr9 | 44796519 | 44796564 | 4 | 2 | NA | FALSE | NA | 0 | 1 | TRUE | FALSE |
| chr9 | 44798436 | 44798530 | 4 | 4 | NA | FALSE | NA | 0 | 1 | FALSE | FALSE |
| chr9 | 44798617 | 44798700 | 5 | 5 | (CATATA)n | TRUE | NA | 0 | 1 | FALSE | FALSE |
| chr9 | 44806290 | 44806321 | 5 | 2 | L1ME3B | TRUE | NA | 0 | NA | FALSE | FALSE |
| chr9 | 44810000 | 44810058 | 5 | 3 | NA | FALSE | NA | 0 | NA | FALSE | FALSE |
| chr9 | 44827025 | 44827087 | 6 | 4 | NA | FALSE | NA | 0 | 1 | FALSE | FALSE |
| chr9 | 44829644 | 44829702 | 6 | 3 | NA | FALSE | NA | 0 | 1 | FALSE | FALSE |
| chr9 | 44830691 | 44830776 | 6 | 6 | NA | FALSE | NA | 0 | 1 | FALSE | FALSE |
| chr9 | 44834658 | 44834717 | 5 | 3 | MIRb | TRUE | NA | 0 | NA | FALSE | FALSE |
| chr9 | 44838292 | 44838328 | 4 | 4 | L2a | TRUE | NA | 0 | 1 | FALSE | FALSE |
| chr9 | 44839678 | 44839705 | 5 | 5 | NA | FALSE | NA | 0 | 1 | FALSE | FALSE |
| chr9 | 44853967 | 44854057 | 6 | 4 | AluSg4 | TRUE | NA | 0 | NA | FALSE | FALSE |
| chr9 | 44855856 | 44855918 | 5 | 5 | NA | FALSE | NA | 0 | 1 | FALSE | FALSE |
| chr9 | 44861906 | 44861967 | 5 | 5 | NA | FALSE | NA | 0 | NA | FALSE | FALSE |
| chr9 | 44862937 | 44863028 | 6 | 5 | NA | FALSE | NA | 0 | 1 | FALSE | FALSE |
| chr9 | 44868933 | 44869017 | 5 | 5 | NA | FALSE | NA | 0 | 1 | FALSE | FALSE |
| chr9 | 45367161 | 45367278 | 5 | 5 | AluJr4 | TRUE | NA | 0 | 1 | FALSE | FALSE |
| chr9 | 45378701 | 45378830 | 6 | 5 | L1PA5 | TRUE | NA | 0 | NA | FALSE | FALSE |
| chr9 | 45418088 | 45418250 | 5 | 5 | NA | FALSE | NA | 0 | NA | FALSE | FALSE |
| chr9 | 45708376 | 45708400 | 5 | 3 | NA | FALSE | NA | 0 | 1 | FALSE | FALSE |
| chr9 | 45709320 | 45709369 | 5 | 5 | NA | FALSE | NA | 0 | NA | FALSE | FALSE |
| chr9 | 46204432 | 46204784 | 7 | 6 | AluSx1 | TRUE | NA | 0 | 1 | FALSE | FALSE |
| chr9 | 46751024 | 46751237 | 4 | 4 | MER5B | TRUE | NA | 0 | 1 | FALSE | FALSE |
| chr9 | 46773642 | 46773730 | 4 | 4 | NA | FALSE | NA | 0 | 1 | FALSE | FALSE |
| chr9 | 46846188 | 46846580 | 6 | 5 | MANY | TRUE | NA | 0 | 1 | FALSE | FALSE |
| chr9 | 46874335 | 46874551 | 7 | 4 | (CA)n | TRUE | NA | 0 | 1 | FALSE | FALSE |
| chr9 | 47171484 | 47171588 | 4 | 3 | MANY | TRUE | NA | 0 | NA | FALSE | FALSE |
| chr9 | 47187260 | 47187360 | 4 | 4 | (CA)n | TRUE | NA | 0 | 1 | FALSE | FALSE |
| chr9 | 47206372 | 47206596 | 4 | 4 | MANY | TRUE | NA | 0 | 1 | FALSE | FALSE |
| chr9 | 47207314 | 47207502 | 4 | 4 | MANY | TRUE | NA | 0 | NA | FALSE | FALSE |
| chr9 | 47207637 | 47207751 | 4 | 4 | NA | FALSE | NA | 0 | 1 | FALSE | FALSE |
| chr9 | 47207929 | 47208167 | 6 | 4 | AluY | TRUE | NA | 0 | 1 | FALSE | FALSE |
| chr9 | 47213462 | 47213506 | 6 | 4 | NA | FALSE | NA | 0 | 1 | FALSE | FALSE |
| chr9 | 65595901 | 65596234 | 4 | 4 | AluSc | TRUE | NA | 0 | 1 | FALSE | FALSE |
| chr9 | 65599498 | 65599805 | 4 | 4 | MANY | TRUE | NA | 0 | NA | FALSE | FALSE |
| chr9 | 65607898 | 65608258 | 5 | 4 | MANY | TRUE | NA | 0 | 1 | FALSE | FALSE |
| chr9 | 66246758 | 66246767 | 6 | 3 | NA | FALSE | NA | 0 | 1 | FALSE | FALSE |
| chr9 | 66260049 | 66260103 | 4 | 3 | NA | FALSE | NA | 0 | 1 | FALSE | FALSE |
| chr9 | 66267797 | 66267827 | 8 | 6 | NA | FALSE | NA | 0 | NA | FALSE | FALSE |
| chr9 | 66302597 | 66302659 | 7 | 4 | L1MEg | TRUE | NA | 0 | 1 | FALSE | FALSE |
| chr9 | 66480690 | 66480736 | 4 | 3 | L1PA6 | TRUE | NA | 0 | NA | FALSE | FALSE |
| chr9 | 66714593 | 66714629 | 4 | 3 | NA | FALSE | NA | 0 | NA | FALSE | FALSE |
| chr9 | 66725049 | 66725097 | 7 | 4 | AluSx1 | TRUE | NA | 0 | NA | FALSE | FALSE |
| chr9 | 67219904 | 67219979 | 4 | 1 | AluJo | TRUE | NA | 0 | NA | FALSE | FALSE |
| chr9 | 67269436 | 67269522 | 5 | 5 | AluSx1 | TRUE | NA | 0 | 1 | FALSE | FALSE |
| chr9 | 67306978 | 67307076 | 5 | 4 | L1PA3 | TRUE | NA | 0 | 1 | FALSE | FALSE |
| chr9 | 67308919 | 67308999 | 4 | 2 | AT rich | TRUE | NA | 0 | NA | FALSE | FALSE |
| chr9 | 67340064 | 67340159 | 4 | 4 | TAR1 | TRUE | MANY | 2 | 1 | TRUE | FALSE |
| chr9 | 67340586 | 67340649 | 4 | 4 | TAR1 | TRUE | NA | 0 | 1 | FALSE | FALSE |
| chr9 | 68242811 | 68242830 | 7 | 6 | NA | FALSE | NA | 0 | 1 | FALSE | FALSE |
| chr9 | 68309819 | 68309845 | 4 | 2 | NA | FALSE | NA | 0 | 1 | FALSE | FALSE |
| chr9 | 68326390 | 68326401 | 6 | 3 | L1MA3 | TRUE | NA | 0 | 1 | FALSE | FALSE |
| chr9 | 68330632 | 68330653 | 4 | 3 | L1PA15-16 | TRUE | NA | 0 | 1 | FALSE | FALSE |
| chr9 | 68345961 | 68346052 | 10 | 8 | NA | FALSE | NA | 0 | 1 | FALSE | FALSE |
| chr9 | 68347597 | 68347626 | 4 | 3 | NA | FALSE | NA | 0 | 1 | FALSE | FALSE |
| chr9 | 68348781 | 68348788 | 5 | 4 | AluY | TRUE | NA | 0 | NA | FALSE | FALSE |
| chr9 | 68352672 | 68352682 | 4 | 4 | MLT1C | TRUE | NA | 0 | 1 | FALSE | FALSE |
| chr9 | 68353313 | 68353338 | 4 | 4 | NA | FALSE | NA | 0 | 1 | FALSE | FALSE |
| chr9 | 68355725 | 68355749 | 9 | 9 | L1PA5 | TRUE | NA | 0 | NA | FALSE | FALSE |
| chr9 | 68377316 | 68377335 | 4 | 4 | TAR1 | TRUE | ZBTB33 | 1 | 1 | TRUE | FALSE |
| chr9 | 68385938 | 68385977 | 4 | 4 | L1PA6 | TRUE | NA | 0 | 1 | FALSE | FALSE |
| chr9 | 68388100 | 68388102 | 6 | 2 | L1PA6 | TRUE | NA | 0 | NA | FALSE | FALSE |
| chr9 | 68476810 | 68476839 | 4 | 2 | NA | FALSE | NA | 0 | 1 | FALSE | FALSE |
| chr9 | 68490709 | 68490738 | 4 | 3 | MIRc | TRUE | NA | 0 | NA | FALSE | FALSE |
| chr9 | 68500092 | 68500124 | 4 | 4 | Ricksha c | TRUE | NA | 0 | 1 | TRUE | FALSE |
| chr9 | 68506372 | 68506374 | 5 | 4 | NA | FALSE | NA | 0 | NA | FALSE | FALSE |
| chr9 | 68513874 | 68513902 | 4 | 4 | CER | TRUE | NA | 0 | 1 | FALSE | FALSE |
| chr9 | 68993079 | 68993154 | 4 | 3 | L1PA3 | TRUE | NA | 0 | 1 | FALSE | FALSE |
| chr9 | 69167087 | 69167158 | 6 | 4 | MANY | TRUE | NA | 0 | NA | FALSE | FALSE |
| chr9 | 69786319 | 69786379 | 4 | 3 | GC rich | TRUE | MANY | 2 | 1 | TRUE | FALSE |
| chr9 | 69795587 | 69795636 | 6 | 4 | L1M5 | TRUE | NA | 0 | 1 | FALSE | FALSE |
| chr9 | 69803991 | 69804047 | 4 | 4 | NA | FALSE | NA | 0 | NA | FALSE | FALSE |
| chr9 | 69811829 | 69811888 | 5 | 5 | L1PA3 | TRUE | NA | 0 | NA | FALSE | FALSE |
| chr9 | 69812936 | 69812953 | 4 | 3 | L1PA3 | TRUE | NA | 0 | NA | FALSE | FALSE |
| chr9 | 69816230 | 69816253 | 4 | 4 | L1PA3 | TRUE | NA | 0 | NA | FALSE | FALSE |
| chr9 | 69819755 | 69819809 | 4 | 4 | CER | TRUE | NA | 0 | 1 | FALSE | FALSE |
| chr9 | 69825349 | 69825400 | 4 | 3 | LTR70 | TRUE | NA | 0 | 1 | FALSE | FALSE |
| chr9 | 69825863 | 69825918 | 4 | 3 | NA | FALSE | TCF4 | 1 | NA | FALSE | FALSE |
| chr9 | 69836674 | 69836729 | 6 | 4 | L1MB2 | TRUE | NA | 0 | 1 | FALSE | FALSE |
| chr9 | 69841118 | 69841187 | 5 | 4 | AluSp | TRUE | NA | 0 | 1 | FALSE | FALSE |
| chr9 | 69846302 | 69846355 | 6 | 4 | NA | FALSE | NA | 0 | 1 | FALSE | FALSE |
| chr9 | 69923785 | 69923828 | 4 | 4 | AluSx1 | TRUE | NA | 0 | NA | FALSE | FALSE |
| chr9 | 69924341 | 69924384 | 4 | 3 | NA | FALSE | NA | 0 | NA | FALSE | FALSE |
| chr9 | 70174806 | 70174857 | 10 | 8 | L1MEb | TRUE | NA | 0 | 1 | FALSE | FALSE |
| chr9 | 70179377 | 70179433 | 4 | 3 | AluY | TRUE | NA | 0 | NA | FALSE | FALSE |
| chr9 | 70484900 | 70485018 | 8 | 8 | AluY | TRUE | NA | 0 | 1 | FALSE | FALSE |
| chr9 | 70499160 | 70499212 | 6 | 5 | AluSq2 | TRUE | NA | 0 | NA | FALSE | FALSE |
| chr9 | 70598283 | 70598353 | 5 | 5 | NA | FALSE | NA | 0 | NA | FALSE | FALSE |
| chr9 | 70954263 | 70954269 | 4 | 3 | AluJb | TRUE | NA | 0 | 1 | FALSE | FALSE |
| chr9 | 70996533 | 70996584 | 4 | 3 | MANY | TRUE | NA | 0 | 1 | FALSE | FALSE |
| chr9 | 71458475 | 71458525 | 4 | 2 | NA | FALSE | NA | 0 | 1 | TRUE | FALSE |
| chr9 | 71641307 | 71641342 | 4 | 3 | AluSx | TRUE | NA | 0 | 1 | FALSE | FALSE |
| chr9 | 74012449 | 74012481 | 4 | 4 | NA | FALSE | NA | 0 | 1 | FALSE | FALSE |
| chr9 | 74090981 | 74090988 | 4 | 4 | NA | FALSE | NA | 0 | 1 | FALSE | FALSE |
| chr9 | 74142955 | 74142975 | 4 | 4 | NA | FALSE | NA | 0 | 1 | FALSE | FALSE |
| chr9 | 74191765 | 74191817 | 4 | 3 | NA | FALSE | NA | 0 | 1 | FALSE | FALSE |
| chr9 | 74719681 | 74719753 | 4 | 4 | AluY | TRUE | NA | 0 | NA | FALSE | FALSE |
| chr9 | 74799436 | 74799479 | 4 | 3 | L1MC1 | TRUE | NA | 0 | 1 | FALSE | FALSE |
| chr9 | 78176259 | 78176278 | 4 | 3 | L1PA15 | TRUE | NA | 0 | 1 | FALSE | FALSE |
| chr9 | 78232801 | 78232819 | 4 | 2 | MLT1C | TRUE | NA | 0 | 1 | FALSE | FALSE |
| chr9 | 78917178 | 78917221 | 5 | 4 | NA | FALSE | NA | 0 | 1 | FALSE | FALSE |
| chr9 | 78934252 | 78934272 | 5 | 4 | LTR13A | TRUE | NA | 0 | NA | FALSE | FALSE |
| chr9 | 80695850 | 80695889 | 4 | 3 | L1PA4 | TRUE | NA | 0 | NA | FALSE | FALSE |
| chr9 | 81028001 | 81028047 | 4 | 3 | (TA)n | TRUE | NA | 0 | NA | FALSE | FALSE |
| chr9 | 81723519 | 81723544 | 4 | 2 | THE1A-int | TRUE | SETDB1 | 1 | 1 | FALSE | FALSE |
| chr9 | 81793776 | 81793796 | 4 | 4 | L1PA3 | TRUE | NA | 0 | NA | FALSE | FALSE |
| chr9 | 82159732 | 82159767 | 10 | 7 | L1PA4 | TRUE | NA | 0 | NA | FALSE | FALSE |
| chr9 | 82366874 | 82366875 | 4 | 2 | THE1B-int | TRUE | KAP1 | 1 | 1 | FALSE | FALSE |
| chr9 | 82398527 | 82398540 | 14 | 7 | AluSc | TRUE | NA | 0 | NA | FALSE | FALSE |
| chr9 | 82707263 | 82707306 | 4 | 3 | NA | FALSE | NA | 0 | 1 | FALSE | FALSE |
| chr9 | 83832384 | 83832423 | 5 | 3 | L1PA4 | TRUE | NA | 0 | 1 | FALSE | FALSE |
| chr9 | 83961762 | 83961819 | 4 | 2 | NA | FALSE | NA | 0 | 1 | FALSE | FALSE |
| chr9 | 84048312 | 84048326 | 6 | 4 | L1PA3 | TRUE | NA | 0 | NA | FALSE | FALSE |
| chr9 | 84575390 | 84575433 | 4 | 4 | L1MCa | TRUE | NA | 0 | 1 | FALSE | FALSE |
| chr9 | 87497635 | 87497637 | 4 | 4 | (TG)n | TRUE | NA | 0 | NA | FALSE | FALSE |
| chr9 | 87559056 | 87559129 | 4 | 4 | (TC)n | TRUE | NA | 0 | 1 | FALSE | FALSE |
| chr9 | 88455882 | 88455894 | 4 | 2 | NA | FALSE | NA | 0 | 1 | FALSE | FALSE |
| chr9 | 88555775 | 88555858 | 5 | 4 | NA | FALSE | MANY | 40 | 1 | TRUE | TRUE |
| chr9 | 88995339 | 88995399 | 5 | 4 | L1PA4 | TRUE | NA | 0 | 1 | FALSE | FALSE |
| chr9 | 90373473 | 90373516 | 4 | 3 | SVA F | TRUE | NA | 0 | NA | FALSE | FALSE |
| chr9 | 90373597 | 90373638 | 4 | 3 | SVA F | TRUE | NA | 0 | 1 | FALSE | FALSE |
| chr9 | 90460082 | 90460082 | 4 | 4 | NA | FALSE | NA | 0 | 1 | FALSE | FALSE |
| chr9 | 90513856 | 90513861 | 4 | 4 | L1M4c | TRUE | NA | 0 | 1 | FALSE | FALSE |
| chr9 | 90539565 | 90539568 | 5 | 3 | NA | FALSE | NA | 0 | NA | TRUE | FALSE |
| chr9 | 91241198 | 91241253 | 5 | 3 | SVA F | TRUE | NA | 0 | NA | FALSE | FALSE |
| chr9 | 91933356 | 91933356 | 5 | 5 | NA | FALSE | MANY | 43 | 1 | TRUE | TRUE |
| chr9 | 93350540 | 93350572 | 5 | 2 | L1MC4 | TRUE | NA | 0 | 1 | FALSE | FALSE |
| chr9 | 95593122 | 95593309 | 7 | 6 | SVA D | TRUE | NA | 0 | NA | FALSE | FALSE |
| chr9 | 97305007 | 97305061 | 4 | 3 | HERVH-int | TRUE | NA | 0 | 1 | FALSE | FALSE |
| chr9 | 97707901 | 97707984 | 4 | 2 | NA | FALSE | MANY | 12 | 1 | TRUE | TRUE |
| chr9 | 98138182 | 98138196 | 5 | 2 | AluSx3 | TRUE | NA | 0 | NA | FALSE | FALSE |
| chr9 | 99477471 | 99477471 | 4 | 4 | SVA B | TRUE | NA | 0 | NA | TRUE | FALSE |
| chr9 | 99907416 | 99907505 | 5 | 5 | MSTA-int | TRUE | NA | 0 | 1 | FALSE | FALSE |
| chr9 | 100015564 | 100015656 | 5 | 3 | MANY | TRUE | NA | 0 | 1 | FALSE | FALSE |
| chr9 | 100790132 | 100790215 | 6 | 6 | AluY | TRUE | NA | 0 | NA | FALSE | FALSE |
| chr9 | 101035693 | 101035719 | 5 | 4 | NA | FALSE | NA | 0 | 1 | TRUE | FALSE |
| chr9 | 101481662 | 101481699 | 8 | 3 | NA | FALSE | NA | 0 | 1 | TRUE | FALSE |
| chr9 | 101627829 | 101627883 | 4 | 3 | NA | FALSE | NA | 0 | 1 | FALSE | FALSE |
| chr9 | 102394683 | 102394726 | 5 | 4 | (TA)n | TRUE | MafK (ab50322) | 1 | NA | FALSE | FALSE |
| chr9 | 103549552 | 103549584 | 5 | 5 | AluSq2 | TRUE | NA | 0 | NA | FALSE | FALSE |
| chr9 | 103648817 | 103648820 | 4 | 4 | L2a | TRUE | NA | 0 | 1 | FALSE | FALSE |
| chr9 | 104412316 | 104412340 | 4 | 4 | NA | FALSE | NA | 0 | 1 | FALSE | FALSE |
| chr9 | 104505786 | 104505794 | 4 | 4 | NA | FALSE | NA | 0 | 1 | FALSE | FALSE |
| chr9 | 104570455 | 104570474 | 9 | 6 | THE1A-int | TRUE | NA | 0 | NA | FALSE | FALSE |
| chr9 | 104761575 | 104761583 | 4 | 4 | NA | FALSE | NA | 0 | 1 | FALSE | FALSE |
| chr9 | 104850218 | 104850226 | 4 | 4 | NA | FALSE | NA | 0 | 1 | FALSE | FALSE |
| chr9 | 104881951 | 104881951 | 5 | 5 | L1PA7 | TRUE | NA | 0 | NA | FALSE | FALSE |
| chr9 | 104881981 | 104881981 | 5 | 5 | L1PA7 | TRUE | NA | 0 | NA | FALSE | FALSE |
| chr9 | 105421993 | 105422011 | 5 | 5 | L1MA1 | TRUE | NA | 0 | 1 | FALSE | FALSE |
| chr9 | 105654212 | 105654231 | 4 | 3 | MER34 | TRUE | NA | 0 | 1 | FALSE | FALSE |
| chr9 | 105699549 | 105699555 | 4 | 3 | MER11C | TRUE | NA | 0 | 1 | FALSE | FALSE |
| chr9 | 105795534 | 105795538 | 4 | 3 | L1MC3 | TRUE | NA | 0 | 1 | FALSE | FALSE |
| chr9 | 106216293 | 106216307 | 5 | 3 | L1P1 | TRUE | NA | 0 | NA | FALSE | FALSE |
| chr9 | 106325406 | 106325427 | 4 | 4 | NA | FALSE | NA | 0 | 1 | FALSE | FALSE |
| chr9 | 106443860 | 106443861 | 4 | 4 | MSTA | TRUE | NA | 0 | 1 | TRUE | FALSE |
| chr9 | 106742406 | 106742426 | 5 | 4 | ERV3-16A3 I-int | TRUE | NA | 0 | 1 | FALSE | FALSE |
| chr9 | 106972866 | 106972885 | 4 | 2 | MSTC | TRUE | NA | 0 | 1 | FALSE | FALSE |
| chr9 | 107072345 | 107072358 | 4 | 2 | L1PA8A | TRUE | NA | 0 | 1 | FALSE | FALSE |
| chr9 | 107167416 | 107167433 | 4 | 3 | NA | FALSE | NA | 0 | 1 | FALSE | FALSE |
| chr9 | 107404969 | 107404969 | 4 | 4 | L1PA13 | TRUE | NA | 0 | 1 | FALSE | FALSE |
| chr9 | 108710638 | 108710725 | 4 | 4 | L1PREC2 | TRUE | NA | 0 | 1 | FALSE | FALSE |
| chr9 | 109553009 | 109553096 | 4 | 3 | HERVH-int | TRUE | NA | 0 | 1 | FALSE | FALSE |
| chr9 | 109664954 | 109664965 | 4 | 2 | L1PB1 | TRUE | NA | 0 | NA | FALSE | FALSE |
| chr9 | 109788917 | 109789006 | 5 | 5 | (TA)n | TRUE | NA | 0 | NA | FALSE | FALSE |
| chr9 | 110289755 | 110289782 | 4 | 2 | NA | FALSE | NA | 0 | 1 | TRUE | FALSE |
| chr9 | 110993306 | 110993372 | 4 | 2 | AluSc | TRUE | NA | 0 | NA | FALSE | FALSE |
| chr9 | 112360509 | 112360517 | 4 | 2 | AluSg | TRUE | NA | 0 | NA | FALSE | FALSE |
| chr9 | 113592841 | 113592860 | 4 | 4 | LTR1 | TRUE | NA | 0 | NA | TRUE | FALSE |
| chr9 | 114149615 | 114149623 | 4 | 4 | NA | FALSE | NA | 0 | NA | FALSE | FALSE |
| chr9 | 115162215 | 115162245 | 6 | 2 | MER11A | TRUE | NA | 0 | NA | FALSE | FALSE |
| chr9 | 115737680 | 115737718 | 4 | 4 | AluY | TRUE | NA | 0 | NA | FALSE | FALSE |
| chr9 | 116163597 | 116163672 | 5 | 4 | NA | FALSE | MANY | 22 | 1 | TRUE | TRUE |
| chr9 | 117665513 | 117665553 | 4 | 4 | MER54B | TRUE | NA | 0 | 1 | FALSE | FALSE |
| chr9 | 117939702 | 117939736 | 4 | 3 | HERVH-int | TRUE | NA | 0 | NA | FALSE | FALSE |
| chr9 | 118253954 | 118253967 | 4 | 2 | NA | FALSE | NA | 0 | 1 | FALSE | FALSE |
| chr9 | 118780802 | 118780839 | 6 | 4 | L1MA9 | TRUE | NA | 0 | 1 | FALSE | FALSE |
| chr9 | 120140967 | 120140978 | 4 | 2 | NA | FALSE | NA | 0 | 1 | FALSE | FALSE |
| chr9 | 120231697 | 120231697 | 5 | 5 | MLT2B1 | TRUE | NA | 0 | NA | FALSE | FALSE |
| chr9 | 120669695 | 120669695 | 4 | 4 | NA | FALSE | NA | 0 | 1 | FALSE | FALSE |
| chr9 | 121441311 | 121441330 | 4 | 3 | SVA B | TRUE | NA | 0 | NA | FALSE | FALSE |
| chr9 | 121561983 | 121561984 | 4 | 4 | NA | FALSE | NA | 0 | 1 | FALSE | FALSE |
| chr9 | 121650907 | 121650922 | 4 | 3 | MANY | TRUE | NA | 0 | 1 | FALSE | FALSE |
| chr9 | 122910851 | 122910881 | 4 | 3 | MLT1C | TRUE | NA | 0 | 1 | FALSE | FALSE |
| chr9 | 123023145 | 123023171 | 5 | 4 | NA | FALSE | NA | 0 | NA | FALSE | FALSE |
| chr9 | 123023356 | 123023363 | 4 | 3 | NA | FALSE | NA | 0 | 1 | FALSE | FALSE |
| chr9 | 123089815 | 123089848 | 4 | 4 | NA | FALSE | NA | 0 | 1 | FALSE | FALSE |
| chr9 | 123650207 | 123650208 | 4 | 2 | NA | FALSE | NA | 0 | 1 | FALSE | FALSE |
| chr9 | 123880124 | 123880161 | 4 | 3 | AluY | TRUE | NA | 0 | NA | FALSE | FALSE |
| chr9 | 124558582 | 124558873 | 12 | 11 | MANY | TRUE | NA | 0 | 1 | TRUE | FALSE |
| chr9 | 125473260 | 125473266 | 6 | 4 | SVA D | TRUE | NA | 0 | NA | FALSE | FALSE |
| chr9 | 126899076 | 126899110 | 5 | 2 | AluSx1 | TRUE | NA | 0 | NA | FALSE | FALSE |
| chr9 | 127533913 | 127533961 | 4 | 4 | NA | FALSE | MANY | 24 | 1 | TRUE | TRUE |
| chr9 | 127713616 | 127713621 | 6 | 3 | AluSq | TRUE | NA | 0 | NA | FALSE | FALSE |
| chr9 | 127962769 | 127962820 | 4 | 3 | NA | FALSE | MANY | 21 | 1 | TRUE | TRUE |
| chr9 | 128153586 | 128153675 | 4 | 2 | MANY | TRUE | NA | 0 | 1 | FALSE | FALSE |
| chr9 | 128342934 | 128342936 | 4 | 2 | L1PA6 | TRUE | NA | 0 | NA | TRUE | FALSE |
| chr9 | 128542658 | 128542776 | 7 | 7 | SVA A | TRUE | NA | 0 | NA | FALSE | FALSE |
| chr9 | 128542903 | 128542958 | 5 | 4 | MANY | TRUE | NA | 0 | NA | FALSE | FALSE |
| chr9 | 128710998 | 128711021 | 4 | 4 | MANY | TRUE | NA | 0 | 1 | TRUE | FALSE |
| chr9 | 128750956 | 128750997 | 4 | 2 | L2a | TRUE | NA | 0 | 1 | TRUE | FALSE |
| chr9 | 128827495 | 128827525 | 4 | 3 | NA | FALSE | NA | 0 | 1 | TRUE | FALSE |
| chr9 | 128858310 | 128858371 | 4 | 3 | NA | FALSE | NA | 0 | 1 | TRUE | FALSE |
| chr9 | 129129650 | 129129710 | 4 | 3 | NA | FALSE | NA | 0 | 1 | TRUE | FALSE |
| chr9 | 129307749 | 129307858 | 5 | 3 | NA | FALSE | NA | 0 | 1 | TRUE | FALSE |
| chr9 | 129484836 | 129484917 | 4 | 2 | NA | FALSE | MANY | 11 | 1 | TRUE | TRUE |
| chr9 | 129944626 | 129944646 | 4 | 3 | (TTCC)n | TRUE | NA | 0 | NA | FALSE | FALSE |
| chr9 | 130176069 | 130176121 | 4 | 2 | HERVH-int | TRUE | NA | 0 | 1 | FALSE | FALSE |
| chr9 | 130820781 | 130820785 | 5 | 3 | AluSc5 | TRUE | NA | 0 | NA | FALSE | FALSE |
| chr9 | 130954113 | 130954114 | 4 | 2 | NA | FALSE | MANY | 12 | 1 | TRUE | TRUE |
| chr9 | 131038338 | 131038413 | 10 | 9 | NA | FALSE | MANY | 24 | 1 | TRUE | TRUE |
| chr9 | 131343025 | 131343140 | 4 | 2 | NA | FALSE | NA | 0 | 1 | TRUE | FALSE |
| chr9 | 131920994 | 131920995 | 5 | 3 | AluY | TRUE | NA | 0 | NA | FALSE | FALSE |
| chr9 | 132565389 | 132565433 | 6 | 6 | NA | FALSE | MANY | 41 | 1 | TRUE | TRUE |
| chr9 | 133300572 | 133300574 | 4 | 2 | NA | FALSE | NA | 0 | 1 | TRUE | FALSE |
| chr9 | 133454936 | 133454937 | 5 | 5 | NA | FALSE | MANY | 34 | 1 | TRUE | TRUE |
| chr9 | 134462260 | 134462379 | 5 | 5 | (CA)n | TRUE | NA | 0 | 1 | FALSE | FALSE |
| chr9 | 134570284 | 134570358 | 7 | 6 | L1PA4 | TRUE | NA | 0 | 1 | FALSE | FALSE |
| chr9 | 134646247 | 134646327 | 4 | 3 | L2b | TRUE | NA | 0 | 1 | TRUE | FALSE |
| chr9 | 135820131 | 135820204 | 4 | 4 | NA | FALSE | MANY | 29 | 1 | TRUE | TRUE |
| chr9 | 135905909 | 135906052 | 6 | 5 | NA | FALSE | MANY | 17 | 1 | TRUE | TRUE |
| chr9 | 135946891 | 135946953 | 5 | 5 | GC rich | TRUE | Egr-1 | 1 | 1 | FALSE | FALSE |
| chr9 | 136130720 | 136130754 | 5 | 3 | (TG)n | TRUE | NA | 0 | NA | FALSE | FALSE |
| chr9 | 136257021 | 136257068 | 4 | 2 | SVA D | TRUE | NA | 0 | 1 | FALSE | FALSE |
| chr9 | 137154776 | 137154811 | 4 | 2 | (CA)n | TRUE | NA | 0 | NA | FALSE | FALSE |
| chr9 | 137240082 | 137240102 | 4 | 2 | NA | FALSE | NA | 0 | 1 | TRUE | FALSE |
| chr9 | 137415650 | 137415660 | 4 | 4 | NA | FALSE | NA | 0 | NA | FALSE | FALSE |
| chr9 | 137443631 | 137443633 | 4 | 3 | NA | FALSE | MafK (ab50322) | 1 | 1 | TRUE | FALSE |
| chr9 | 137738393 | 137738436 | 4 | 4 | NA | FALSE | NA | 0 | 1 | TRUE | FALSE |
| chr9 | 138088435 | 138088466 | 4 | 3 | C-rich | TRUE | NA | 0 | 1 | TRUE | FALSE |
| chr9 | 138092802 | 138092803 | 4 | 2 | NA | FALSE | NA | 0 | NA | FALSE | FALSE |
| chr9 | 138329577 | 138329616 | 5 | 3 | HERVE-int | TRUE | NA | 0 | 1 | FALSE | FALSE |
| chr9 | 139017235 | 139017257 | 4 | 3 | (TTGG)n | TRUE | NA | 0 | 1 | FALSE | FALSE |
| chr9 | 139066579 | 139066641 | 4 | 3 | NA | FALSE | NA | 0 | 1 | FALSE | FALSE |
| chr9 | 139430258 | 139430293 | 4 | 3 | NA | FALSE | ZNF263 | 1 | 1 | FALSE | FALSE |
| chr9 | 139886221 | 139886260 | 16 | 8 | AluSx | TRUE | MANY | 13 | NA | TRUE | FALSE |
| chr9 | 139997531 | 139997615 | 6 | 4 | NA | FALSE | NA | 0 | 1 | FALSE | FALSE |
| chr9 | 139998108 | 139998126 | 4 | 2 | NA | FALSE | NA | 0 | NA | FALSE | FALSE |
| chr9 | 140273607 | 140273690 | 5 | 3 | NA | FALSE | NA | 0 | NA | TRUE | FALSE |
| chr9 | 140403896 | 140403934 | 6 | 5 | NA | FALSE | HA-E2F1 | 1 | 1 | FALSE | FALSE |
| chr9 | 140412589 | 140412709 | 6 | 4 | (CTCA)n | TRUE | NA | 0 | 1 | FALSE | FALSE |
| chr9 | 140675594 | 140675615 | 5 | 4 | NA | FALSE | NA | 0 | NA | FALSE | FALSE |
| chr9 | 140783002 | 140783075 | 5 | 4 | NA | FALSE | NA | 0 | 1 | TRUE | FALSE |
| chr9 | 140849330 | 140849358 | 5 | 4 | MANY | TRUE | NA | 0 | NA | TRUE | FALSE |
| chr9 | 141045222 | 141045301 | 4 | 4 | AluSp | TRUE | NA | 0 | 1 | TRUE | FALSE |
| chr9 | 141092104 | 141092121 | 5 | 3 | NA | FALSE | NA | 0 | 1 | TRUE | FALSE |
| chr10 | 98350 | 98453 | 6 | 4 | MER41D | TRUE | NA | 0 | 1 | TRUE | FALSE |
| chr10 | 98792 | 98916 | 11 | 5 | NA | FALSE | NA | 0 | 1 | FALSE | FALSE |
| chr10 | 110096 | 110143 | 6 | 5 | L1MA2 | TRUE | NA | 0 | 1 | TRUE | FALSE |
| chr10 | 115209 | 115369 | 10 | 5 | NA | FALSE | NA | 0 | 1 | TRUE | FALSE |
| chr10 | 117057 | 117185 | 4 | 2 | AluSz | TRUE | NA | 0 | 1 | FALSE | FALSE |
| chr10 | 127292 | 127300 | 4 | 3 | (CA)n | TRUE | NA | 0 | NA | FALSE | FALSE |
| chr10 | 129399 | 129508 | 5 | 3 | NA | FALSE | NA | 0 | 1 | FALSE | FALSE |
| chr10 | 383238 | 383240 | 4 | 3 | NA | FALSE | NA | 0 | NA | FALSE | FALSE |
| chr10 | 387322 | 387343 | 4 | 3 | NA | FALSE | NA | 0 | 1 | TRUE | FALSE |
| chr10 | 447184 | 447184 | 4 | 4 | NA | FALSE | NA | 0 | 1 | FALSE | FALSE |
| chr10 | 634096 | 634164 | 4 | 4 | (CCCCAG)n | TRUE | MANY | 2 | NA | TRUE | FALSE |
| chr10 | 749754 | 749833 | 4 | 4 | L1MEf | TRUE | NA | 0 | 1 | FALSE | FALSE |
| chr10 | 840129 | 840177 | 4 | 3 | NA | FALSE | NA | 0 | 1 | FALSE | FALSE |
| chr10 | 1114282 | 1114332 | 5 | 2 | AluY | TRUE | NA | 0 | 1 | FALSE | FALSE |
| chr10 | 1681159 | 1681191 | 4 | 3 | NA | FALSE | NA | 0 | 1 | FALSE | FALSE |
| chr10 | 1717387 | 1717420 | 4 | 4 | NA | FALSE | NA | 0 | NA | FALSE | FALSE |
| chr10 | 2565188 | 2565221 | 5 | 4 | NA | FALSE | NA | 0 | NA | FALSE | FALSE |
| chr10 | 2779181 | 2779203 | 4 | 4 | A-rich | TRUE | NA | 0 | 1 | FALSE | FALSE |
| chr10 | 2789597 | 2789632 | 4 | 4 | NA | FALSE | NA | 0 | 1 | FALSE | FALSE |
| chr10 | 2924187 | 2924230 | 4 | 3 | NA | FALSE | NA | 0 | 1 | FALSE | FALSE |
| chr10 | 2991540 | 2991566 | 4 | 3 | GA-rich | TRUE | NA | 0 | NA | FALSE | FALSE |
| chr10 | 3868174 | 3868256 | 4 | 3 | NA | FALSE | NA | 0 | 1 | TRUE | FALSE |
| chr10 | 3897745 | 3897749 | 4 | 2 | MLT1A | TRUE | NA | 0 | 1 | FALSE | FALSE |
| chr10 | 4191646 | 4191750 | 6 | 4 | LTR1 | TRUE | GABP | 1 | NA | FALSE | FALSE |
| chr10 | 4999198 | 4999198 | 4 | 4 | NA | FALSE | FOSL2 | 1 | NA | TRUE | FALSE |
| chr10 | 5011450 | 5011499 | 6 | 5 | NA | FALSE | Pol2 | 1 | 1 | FALSE | FALSE |
| chr10 | 5097319 | 5097326 | 4 | 2 | HERV9-int | TRUE | GATA-1 | 1 | NA | FALSE | FALSE |
| chr10 | 5355533 | 5355568 | 4 | 2 | NA | FALSE | NA | 0 | 1 | FALSE | FALSE |
| chr10 | 5724670 | 5724681 | 4 | 3 | L1ME3B | TRUE | NA | 0 | 1 | TRUE | FALSE |
| chr10 | 5855549 | 5855601 | 4 | 2 | NA | FALSE | MANY | 31 | 1 | TRUE | TRUE |
| chr10 | 5932065 | 5932086 | 4 | 4 | NA | FALSE | MANY | 24 | 1 | TRUE | TRUE |
| chr10 | 6360855 | 6360891 | 4 | 3 | NA | FALSE | NA | 0 | 1 | FALSE | FALSE |
| chr10 | 7563371 | 7563378 | 4 | 2 | (TTCC)n | TRUE | NA | 0 | 1 | FALSE | FALSE |
| chr10 | 7830001 | 7830002 | 5 | 5 | NA | FALSE | MANY | 16 | 1 | TRUE | TRUE |
| chr10 | 8530287 | 8530302 | 4 | 3 | NA | FALSE | NA | 0 | 1 | FALSE | FALSE |
| chr10 | 8694771 | 8694790 | 4 | 4 | NA | FALSE | NA | 0 | 1 | FALSE | FALSE |
| chr10 | 9477228 | 9477229 | 4 | 3 | NA | FALSE | NA | 0 | 1 | FALSE | FALSE |
| chr10 | 9486326 | 9486341 | 4 | 4 | NA | FALSE | NA | 0 | 1 | FALSE | FALSE |
| chr10 | 9523415 | 9523430 | 4 | 4 | NA | FALSE | NA | 0 | 1 | FALSE | FALSE |
| chr10 | 10353345 | 10353347 | 5 | 4 | (TC)n | TRUE | NA | 0 | NA | FALSE | FALSE |
| chr10 | 10451267 | 10451271 | 4 | 2 | NA | FALSE | NA | 0 | NA | FALSE | FALSE |
| chr10 | 12110915 | 12110948 | 4 | 3 | NA | FALSE | MANY | 30 | 1 | TRUE | TRUE |
| chr10 | 12288996 | 12289040 | 6 | 3 | NA | FALSE | GR | 1 | 1 | FALSE | FALSE |
| chr10 | 12677581 | 12677610 | 4 | 4 | NA | FALSE | YY1 | 1 | 1 | TRUE | FALSE |
| chr10 | 12931392 | 12931433 | 5 | 4 | MLT1A | TRUE | NA | 0 | 1 | FALSE | FALSE |
| chr10 | 13561276 | 13561335 | 5 | 2 | AluY | TRUE | NA | 0 | NA | FALSE | FALSE |
| chr10 | 14425666 | 14425692 | 4 | 4 | AluSx | TRUE | NA | 0 | 1 | FALSE | FALSE |
| chr10 | 15815763 | 15815794 | 4 | 3 | L1PA3 | TRUE | NA | 0 | NA | FALSE | FALSE |
| chr10 | 16319404 | 16319413 | 4 | 4 | MANY | TRUE | NA | 0 | 1 | FALSE | FALSE |
| chr10 | 16350396 | 16350431 | 4 | 4 | AluJb | TRUE | NA | 0 | 1 | FALSE | FALSE |
| chr10 | 17265112 | 17265128 | 4 | 3 | L1PA17 | TRUE | NA | 0 | NA | FALSE | FALSE |
| chr10 | 18233637 | 18233715 | 4 | 2 | L1P3 | TRUE | NA | 0 | 1 | FALSE | FALSE |
| chr10 | 18270239 | 18270306 | 4 | 3 | NA | FALSE | NA | 0 | 1 | TRUE | FALSE |
| chr10 | 18361741 | 18361783 | 4 | 4 | AluSg4 | TRUE | NA | 0 | 1 | FALSE | FALSE |
| chr10 | 18373369 | 18373418 | 4 | 4 | NA | FALSE | NA | 0 | 1 | FALSE | FALSE |
| chr10 | 18795884 | 18795915 | 4 | 2 | AluJb | TRUE | NA | 0 | NA | FALSE | FALSE |
| chr10 | 19031656 | 19031681 | 4 | 3 | L1ME1 | TRUE | NA | 0 | 1 | FALSE | FALSE |
| chr10 | 19361130 | 19361148 | 4 | 4 | NA | FALSE | NA | 0 | 1 | FALSE | FALSE |
| chr10 | 19442946 | 19442964 | 4 | 3 | NA | FALSE | NA | 0 | 1 | FALSE | FALSE |
| chr10 | 20679782 | 20679790 | 5 | 3 | L1MEg | TRUE | NA | 0 | 1 | FALSE | FALSE |
| chr10 | 20887294 | 20887303 | 4 | 3 | NA | FALSE | NA | 0 | 1 | FALSE | FALSE |
| chr10 | 20958200 | 20958224 | 5 | 3 | NA | FALSE | NA | 0 | 1 | FALSE | FALSE |
| chr10 | 21423192 | 21423203 | 4 | 3 | NA | FALSE | NA | 0 | 1 | FALSE | FALSE |
| chr10 | 21471100 | 21471120 | 4 | 4 | AluJr | TRUE | NA | 0 | 1 | FALSE | FALSE |
| chr10 | 21945074 | 21945107 | 4 | 3 | NA | FALSE | eGFP-GATA2 | 1 | 1 | TRUE | FALSE |
| chr10 | 23562835 | 23562867 | 4 | 3 | MER11B | TRUE | NA | 0 | 1 | FALSE | FALSE |
| chr10 | 24125600 | 24125625 | 4 | 3 | AluJb | TRUE | NA | 0 | 1 | FALSE | FALSE |
| chr10 | 25013718 | 25013724 | 4 | 4 | NA | FALSE | MANY | 18 | 1 | TRUE | TRUE |
| chr10 | 25654279 | 25654294 | 4 | 3 | NA | FALSE | NA | 0 | 1 | FALSE | FALSE |
| chr10 | 26484728 | 26484757 | 4 | 4 | L2c | TRUE | NA | 0 | 1 | FALSE | FALSE |
| chr10 | 26544536 | 26544560 | 5 | 5 | L2b | TRUE | NA | 0 | 1 | FALSE | FALSE |
| chr10 | 26663164 | 26663165 | 4 | 4 | NA | FALSE | NA | 0 | 1 | FALSE | FALSE |
| chr10 | 26863602 | 26863623 | 5 | 4 | NA | FALSE | NA | 0 | 1 | FALSE | FALSE |
| chr10 | 27443327 | 27443328 | 6 | 5 | NA | FALSE | MANY | 34 | 1 | TRUE | TRUE |
| chr10 | 27472332 | 27472372 | 5 | 2 | AluSc | TRUE | NA | 0 | NA | FALSE | FALSE |
| chr10 | 27731963 | 27731965 | 4 | 3 | (TATATG)n | TRUE | NA | 0 | 1 | FALSE | FALSE |
| chr10 | 27732684 | 27732716 | 6 | 6 | (CATA)n | TRUE | NA | 0 | NA | FALSE | FALSE |
| chr10 | 27758910 | 27758947 | 4 | 4 | HERVH-int | TRUE | NA | 0 | NA | FALSE | FALSE |
| chr10 | 28001436 | 28001452 | 4 | 3 | NA | FALSE | NA | 0 | 1 | FALSE | FALSE |
| chr10 | 28082601 | 28082615 | 4 | 4 | NA | FALSE | NA | 0 | 1 | FALSE | FALSE |
| chr10 | 28254437 | 28254452 | 5 | 4 | NA | FALSE | NA | 0 | 1 | FALSE | FALSE |
| chr10 | 28364228 | 28364247 | 4 | 4 | NA | FALSE | NA | 0 | 1 | FALSE | FALSE |
| chr10 | 29039863 | 29039926 | 4 | 2 | AluSq2 | TRUE | NA | 0 | NA | FALSE | FALSE |
| chr10 | 29402113 | 29402189 | 4 | 4 | MANY | TRUE | NA | 0 | 1 | TRUE | FALSE |
| chr10 | 29625509 | 29625530 | 8 | 3 | L1PA6 | TRUE | NA | 0 | NA | FALSE | FALSE |
| chr10 | 29649289 | 29649289 | 4 | 4 | Tigger3c | TRUE | NA | 0 | NA | FALSE | FALSE |
| chr10 | 30171862 | 30171877 | 5 | 3 | NA | FALSE | NA | 0 | 1 | TRUE | FALSE |
| chr10 | 32049174 | 32049188 | 6 | 3 | NA | FALSE | MANY | 10 | 1 | TRUE | TRUE |
| chr10 | 32404443 | 32404500 | 5 | 5 | (TTC)n | TRUE | NA | 0 | NA | FALSE | FALSE |
| chr10 | 32425330 | 32425404 | 4 | 3 | HERVH-int | TRUE | NA | 0 | 1 | FALSE | FALSE |
| chr10 | 33042787 | 33042814 | 4 | 4 | NA | FALSE | NA | 0 | 1 | FALSE | FALSE |
| chr10 | 34017160 | 34017189 | 4 | 3 | LTR8 | TRUE | NA | 0 | 1 | FALSE | FALSE |
| chr10 | 35263883 | 35263963 | 4 | 4 | HERVE-int | TRUE | NA | 0 | 1 | TRUE | FALSE |
| chr10 | 35307860 | 35307917 | 4 | 4 | NA | FALSE | NA | 0 | 1 | FALSE | FALSE |
| chr10 | 35754867 | 35754888 | 4 | 4 | MANY | TRUE | NA | 0 | NA | TRUE | FALSE |
| chr10 | 36145063 | 36145093 | 4 | 2 | C-rich | TRUE | NA | 0 | 1 | FALSE | FALSE |
| chr10 | 36668233 | 36668249 | 4 | 3 | MER2 | TRUE | NA | 0 | 1 | FALSE | FALSE |
| chr10 | 36883907 | 36883926 | 4 | 4 | NA | FALSE | NA | 0 | 1 | FALSE | FALSE |
| chr10 | 37018052 | 37018056 | 4 | 3 | (TG)n | TRUE | NA | 0 | NA | TRUE | FALSE |
| chr10 | 37454973 | 37454979 | 4 | 4 | NA | FALSE | NA | 0 | 1 | FALSE | FALSE |
| chr10 | 38009731 | 38009750 | 4 | 3 | NA | FALSE | NA | 0 | 1 | FALSE | FALSE |
| chr10 | 38456413 | 38456414 | 4 | 4 | L1MC1 | TRUE | NA | 0 | 1 | FALSE | FALSE |
| chr10 | 38585464 | 38585464 | 5 | 5 | Tigger3a | TRUE | NA | 0 | NA | FALSE | FALSE |
| chr10 | 38637968 | 38637969 | 4 | 3 | NA | FALSE | NA | 0 | 1 | FALSE | FALSE |
| chr10 | 38690683 | 38690691 | 6 | 4 | AluJb | TRUE | CEBPB | 1 | NA | TRUE | FALSE |
| chr10 | 38711403 | 38711404 | 6 | 4 | AluJr | TRUE | NA | 0 | NA | FALSE | FALSE |
| chr10 | 38734909 | 38734920 | 5 | 2 | AluJr | TRUE | NA | 0 | 1 | FALSE | FALSE |
| chr10 | 38735918 | 38735918 | 4 | 4 | LTR41 | TRUE | NA | 0 | 1 | FALSE | FALSE |
| chr10 | 38735965 | 38735982 | 4 | 4 | LTR41 | TRUE | NA | 0 | 1 | FALSE | FALSE |
| chr10 | 38736007 | 38736015 | 5 | 3 | LTR41 | TRUE | NA | 0 | 1 | TRUE | FALSE |
| chr10 | 38765592 | 38765600 | 9 | 5 | L1PA14 | TRUE | NA | 0 | 1 | FALSE | FALSE |
| chr10 | 38889862 | 38889878 | 4 | 4 | L1MB4 | TRUE | NA | 0 | NA | FALSE | FALSE |
| chr10 | 38900828 | 38900842 | 4 | 3 | NA | FALSE | NA | 0 | NA | FALSE | FALSE |
| chr10 | 38907990 | 38907990 | 4 | 4 | L1PBa | TRUE | NA | 0 | NA | FALSE | FALSE |
| chr10 | 38919622 | 38919633 | 4 | 4 | L1M5 | TRUE | NA | 0 | 1 | FALSE | FALSE |
| chr10 | 38928599 | 38928617 | 4 | 4 | NA | FALSE | NA | 0 | 1 | FALSE | FALSE |
| chr10 | 38935646 | 38935646 | 4 | 4 | MIR | TRUE | NA | 0 | 1 | FALSE | FALSE |
| chr10 | 39038207 | 39038207 | 4 | 4 | (AGATG)n | TRUE | NA | 0 | NA | FALSE | FALSE |
| chr10 | 39038232 | 39038232 | 4 | 4 | NA | FALSE | NA | 0 | 1 | FALSE | FALSE |
| chr10 | 39061827 | 39061828 | 6 | 5 | NA | FALSE | NA | 0 | 1 | FALSE | FALSE |
| chr10 | 42602225 | 42602245 | 5 | 5 | L1M5 | TRUE | NA | 0 | 1 | FALSE | FALSE |
| chr10 | 42605406 | 42605429 | 5 | 4 | L1MCa | TRUE | NA | 0 | NA | FALSE | FALSE |
[truncated: 307,212 more chars]
